# The Reverse Transcriptases Associated with CRISPR-Cas Systems

**DOI:** 10.1038/s41598-017-07828-y

**Published:** 2017-08-02

**Authors:** Nicolás Toro, Francisco Martínez-Abarca, Alejandro González-Delgado

**Affiliations:** Structure, Dynamics and Function of Rhizobacterial Genomes, Grupo de Ecología Genética de la Rizosfera, Department of Soil Microbiology and Symbiotic Systems, Estación Experimental del Zaidín, Consejo Superior de Investigaciones Científicas, C/Profesor Albareda 1, 18008 Granada, Spain

## Abstract

CRISPR (clustered regularly interspaced short palindromic repeats) and associated proteins (Cas) act as adaptive immune systems in bacteria and archaea. Some CRISPR-Cas systems have been found to be associated with putative reverse transcriptases (RT), and an RT-Cas1 fusion associated with a type III-B system has been shown to acquire RNA spacers *in vivo*. Nevertheless, the origin and evolutionary relationships of these RTs and associated CRISPR-Cas systems remain largely unknown. We performed a comprehensive phylogenetic analysis of these RTs and associated Cas1 proteins, and classified their CRISPR-Cas modules. These systems were found predominantly in bacteria, and their presence in archaea may be due to a horizontal gene transfer event. These RTs cluster into 12 major clades essentially restricted to particular phyla, suggesting host-dependent functioning. The RTs and associated Cas1 proteins may have largely coevolved. They are, therefore, subject to the same selection pressures, which may have led to coadaptation within particular protein complexes. Furthermore, our results indicate that the association of an RT with a CRISPR-Cas system has occurred on multiple occasions during evolution.

## Introduction

The reverse transcriptase enzyme^1, 2^ responsible for converting RNA into cDNA is required for genome invasion by mobile retroelements and the spread of these elements through the reverse transcription of RNA transposition intermediates^3, 4^. The first bacterial RT to be discovered was found in a retroelement known as a retron^5, 6^, but more than 50% of the bacterial RTs identified to date are encoded by group II introns^7^, which are catalytic RNAs and mobile retroelements. Phylogenetic analyses have shown that bacterial RTs can be classified into 17 main groups^7^. Some of these RTs appear to be more closely related to those encoded by mobile group II introns and have been found to be associated with clustered regularly interspaced short palindromic repeats (CRISPR) and CRISPR-associated protein genes (by *cas* genes), with these genes either separate or resulting in a natural fusion, at the C-terminus, to Cas1^7–10^.

CRISPR-Cas modules are adaptive immune systems present in most archaea and about 40% of bacteria^11, 12^. They provide sequence-specific protection against foreign viruses and plasmids^13^. CRISPR-Cas immunity can be broken down into three stages: adaptation, expression, and interference^14–16^. CRISPR arrays consist of repeated sequences (repeats) separated by variable sequences (spacers). Cas1 and Cas2 proteins are required for the acquisition of DNA spacers^17, 18^ by the CRISPR locus (adaptation), and they display polarity towards the leader sequence end of the array. The CRISPR array provides a precursor transcript (precursor crRNA) that is processed into short (mature crRNA) structured RNAs (expression), leading to the formation of crRNA-Cas effector complexes that recognize and bind complementary nucleic acids, resulting in degradation of the target molecule (interference). CRISPR-Cas systems are highly diverse^19, 20^, and it has been suggested that this diversity reflects a rapid evolutionary process and extensive horizontal transfer. These immunogenic systems are currently classified into two broad classes on the basis of the crRNA-effector complexes they form. Class 1 systems have multisubunit effector complexes and are of types I, III and IV, whereas class 2 systems have a single protein and are of types II, V or VI. The six types can be broken down into 19 subtypes^21–23^. Interestingly, RTs seem to be associated exclusively with type III CRISPR-Cas systems^10^.

A particular feature of the associated multisubunit effector complexes of type III systems is the targeting of both single-stranded RNA and transcriptionally active DNA. The effector complexes of type III-A and type III-B systems (Csm/Cmr complexes, respectively) have been found to have a common mechanism of RNA-dependent DNA degradation^24–27^. In a type III-B system present in the marine bacterium *Marinomonas mediterranea* (MMB-1), the associated RT-Cas1 fusion was recently shown to facilitate the RT-dependent acquisition of RNA spacers *in vivo* through a mechanism displaying several similarities to group II intron retrohoming^10^. It is, thus, possible that the association of an RT with a type III-CRISPR-Cas system expands immunity to parasitic RNA sequences and, possibly, to highly transcribed regions of phage and plasmid DNA^10^. It has been suggested that this association has a small number of common origins, but no comprehensive analysis of the origin and evolutionary relationships of these RTs (separate or fused to Cas1) associated with CRISPR-Cas systems and their *cas* gene architectures has ever been carried out. We addressed these issues in this work.

## Results and Discussion

**Figure 1:**
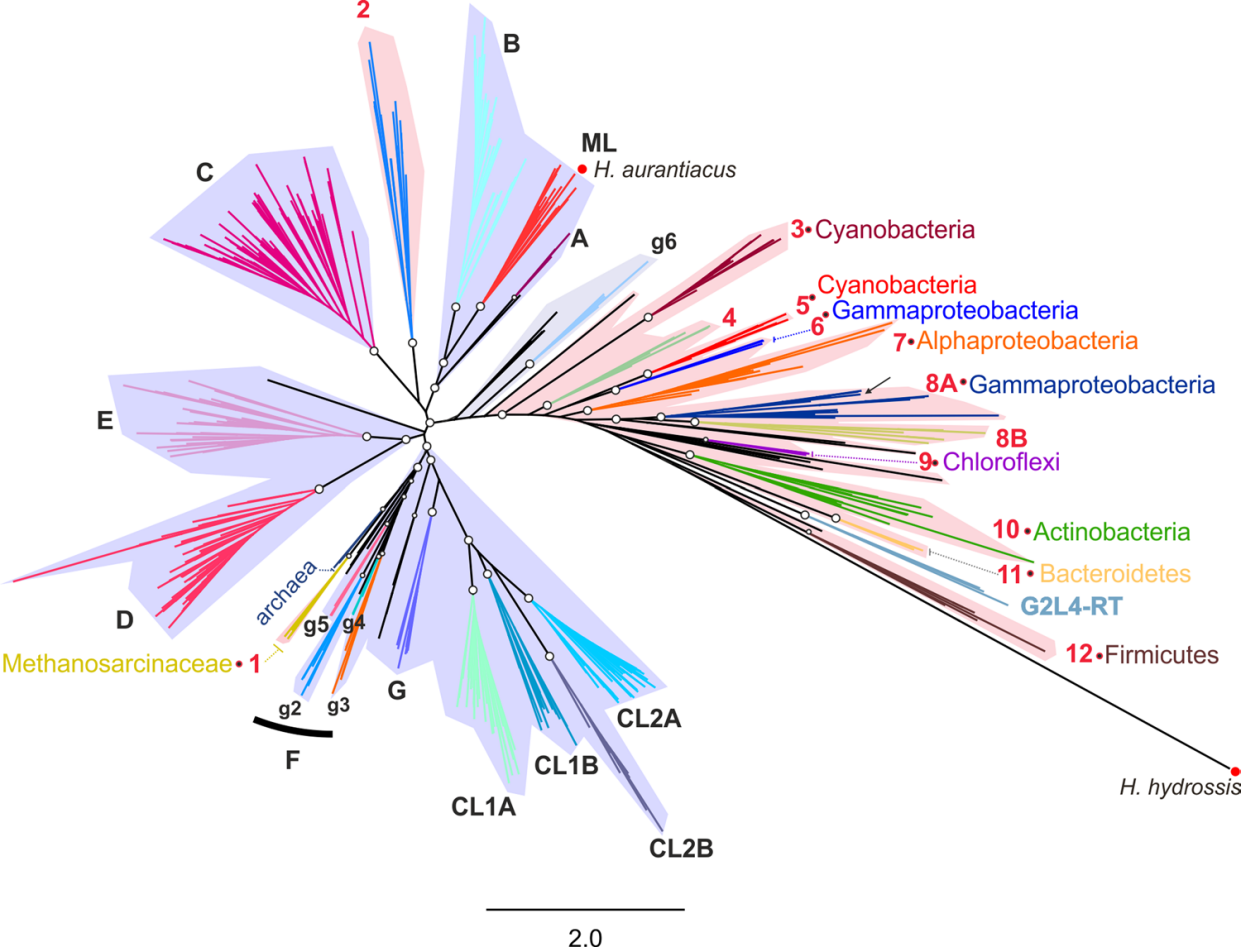
Unrooted phylogenetic tree encompassing the diversity of RTs associated with CRISPR-Cas systems. The tree includes 118 RT sequences associated with CRISPR-Cas systems and 419 closely related RT sequences (Methods). Note that the RTs associated with CRISPR-Cas^21^ (highlighted with red dots) from *Herpetosiphon aurantiacus* (GI: 159898445) and *Haliscomenobacter hydrossis* (GI: 332661943) correspond to a group II intron and a retron/retron-like RT, respectively. The arrow indicates the position of the *M*. *mediterranea* (MMB-1) RT. Group II intron classes and varieties are highlighted in color and their names are indicated in black. All group II introns RTs are shadowed in light purple. The RT clades associated with CRISPR-*cas* loci are highlighted in color and their names are indicated in red. All RTs associated with CRISPR-Cas systems are shadowed in light pink. Open circles at the nodes indicate that the node concerned has a FastTree support value ≥0.92. The phyla restricted to particular RT-CRISPR clades are indicated.

A phylogeny of RTs associated with CRISPR-Cas systems was generated (Fig. 1) by constructing a phylogenetic tree consisting of 537 sequences based on RTs associated with CRISPR-Cas systems described in previous studies, protein sequences carrying both Cas1 and RT domains present in the NCBI database, annotated RTs in complete archaeal genome sequences shown here to be associated with CRISPR-*cas* loci, together with group II intron RTs and more closely related RT-like sequences (see Methods). This phylogenetic clustering identified 12 major clades. By contrast to the extensive horizontal transfer observed for CRISPR-*cas* loci, most of the clades identified for RT sequences were limited to particular phyla (Fig. 1 and Table 1) suggesting host-dependent functioning.

**Table 1:** Distribution of RTs associated with CRISPR-cas systems.

	Clade	Taxonomic adscription	Records^a^	Associated Effector complex			Type of RT		
				C mr (B-C)	C sm(A-D)	(−)^b^	RT	RT-Cas1	Cas6-RT-Cas1
Archaea	All	Taxonomic adscription	110	26	43	41	14	76	20^c^
	1	Euryarcheota (Methanosarcinaceae)	5	2	3	0	5	0	0
Bacteria	2	Planctomycetia, Bacteroidetes, (Delta, Epsilon) Proteobacteria	13	2	4	7	0	13	0
	3	Cyanobacteria	15	4	6	5	0	15	0
	4	Planctomycetia, Chlorobi, (Gamma, Delta) Proteobacteria, no-rank phyla	6	0	4	2	2	4	0
	5	Cyanobacteria	8	1	2	5	3	5	0
	6	Gammaproteobacteria	3	0	2	1	0	3	0
	7	(Alpha, Delta) proteobacteria^d^	13	2	4	7	1	12	0
	8								
	8A	Gammaproteobacteria	23	6	9	8	0	7	16^c^
	8B	Planctomycetia, (Beta, Gamma, Delta) Proteobacteria							
	9	Chloroflexi	3	0	0	3	3	0	0
	10	Actinobacteria	12	5	7	0	0	12	0
	11	Bacteroidetes	4	3	0	1	0	0	4
	12	Firmicutes	5	1	2	3	0	5	0

^(a)^Number of representative RTs described in this study (≤85% identity) corresponding to Tables S1–S12.

^(b)^N^o^ records with partial or unknown effector complex associated.

^(c)^One of the records corresponds to a Cas6-RT fusion gene without a recognizable Cas1 domain.

^(d)^92% of the records belong to Alphaproteobacteria (12/13).

Various studies have shown that CRISPR-Cas systems are much more prevalent in archaea than in bacteria^11, 12^. By contrast, RTs are associated with CRISPR-*cas* loci more frequently in bacterial phyla than in archaea. Indeed, all the RT sequences associated with CRISPR-Cas systems from 262 complete archaeal genome sequences clustered into a single clade (RT-CRISPR-1). These RTs remained separate from Cas1, as for members of the bacterial RT-CRISPR-9 (Chloroflexi) group (Table 1), suggesting that the two clades may have a more recent evolutionary history. A BLAST search of 3043 annotated RTs from 6291 complete bacterial genome sequences available from the PATRIC (Pathogenic Resource Integration Center) platform^28^ against a local database containing RT consensus (Supplementary Figure 1) sequences from the various RT-CRISPR groups and phylogenetic analyses detected no bacterial sequences within the RT-CRISPR-1 clade. Remarkably, these archaeal RTs are present in only two genera from the Methanosarcinaceae family (*Methanosarcina* and *Methanomethylovorans*), and they share a common ancestor with two RT sequences from uncultured archaea that do not seem to be associated with either CRISPR arrays or *cas* genes.

Group II introns, which are known to be present in taxonomically identified archaea, are present in only a few methanogenic species, such as *Methanosarcina* spp., and their presence in these species is thought to result from horizontal transmission from bacteria^29–31^. Furthermore, RT-CRISPR-1 seems to have split off from a node common to class F group II intron RTs (Fig. 1). Together, these observations suggest that this association of an RT with a CRISPR-Cas system may have been laterally transferred from bacteria into *Methanosarcina* spp., in which this association is more widespread. It is, therefore, tempting to speculate that there may have been an earlier group II intron invasion underlying the emergence of the RT-CRISPR-1 clade.

**Figure 2:**
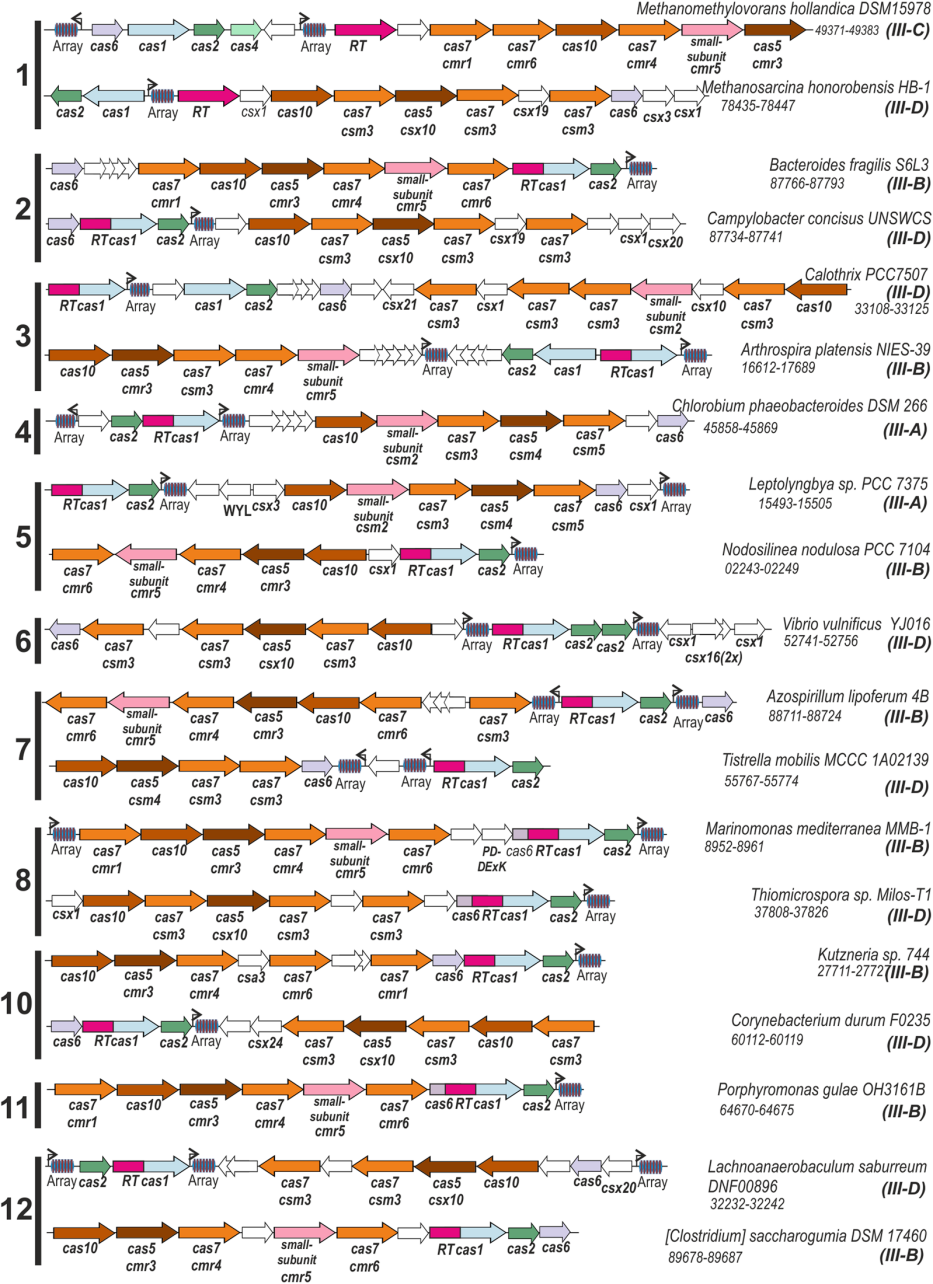
Architectures of the genomic loci for the subtypes of CRISPR-Cas systems associated with RTs. A representative operon is shown for types IIIB/C and III-A/D for 11 of the 12 RT phylogenetic clades including CRISPR-array (*Array*) sites. For each representative genome, the corresponding gene locus tag (final digits) is indicated. Homologous genes are color-coded and identified by family (based on the findings of Makarova *et al*.)^21^. Warm colors correspond to effector genes and cold colors correspond to adaptive genes. RT function is indicated by a fuchsia color. Ancillary and unknown functions are not color-coded. Gene names follow reported classifications and assignments^21^. When available, both a systematic (above) and a ‘legacy’^11^ (below) name are indicated. Only complete loci are shown (no complete operons are available for clade 9). The diagrams are not drawn to scale.

The current classification of CRISPR-Cas systems combines signature genes with elements of the architecture of *cas* loci. Using the reported library of 395 profiles representing all 93 protein families known to be associated with CRISPR-Cas systems^21^, we analyzed the genes located close to the RT gene in the prokaryotic genomes for each clade, identified the putative *cas* genes, and determined the proximity to a CRISPR array and its putative orientation (see Methods). The identified combination of signature genes and distinctive operon organization features of these RT genes associated with CRISPR-*cas* loci is shown in Fig. 2. The CRISPR array data (Supplementary Table 1) will be useful for future CRISPR-based experimental studies on these particular systems. All the RTs associated with complete CRISPR-Cas systems were associated with type III systems (Supplementary Table 1) classified as subtypes III-A/D and III-B/C, with a predominance of Csm (A/D) complexes (Table 1). Interestingly, subtypes III-C and III-D have been reported to lack *cas1* and *cas2* ^21^, but the subtypes associated with RTs harbored both adaptation and effector loci. Moreover, The RT-Cas1 fusions of the RT-CRISPR-8 and RT-CRISPR-11 clades have acquired a Cas6 domain at their N-termini, and this event has clearly occurred independently on at least two occasions during evolution.

**Figure 3:**
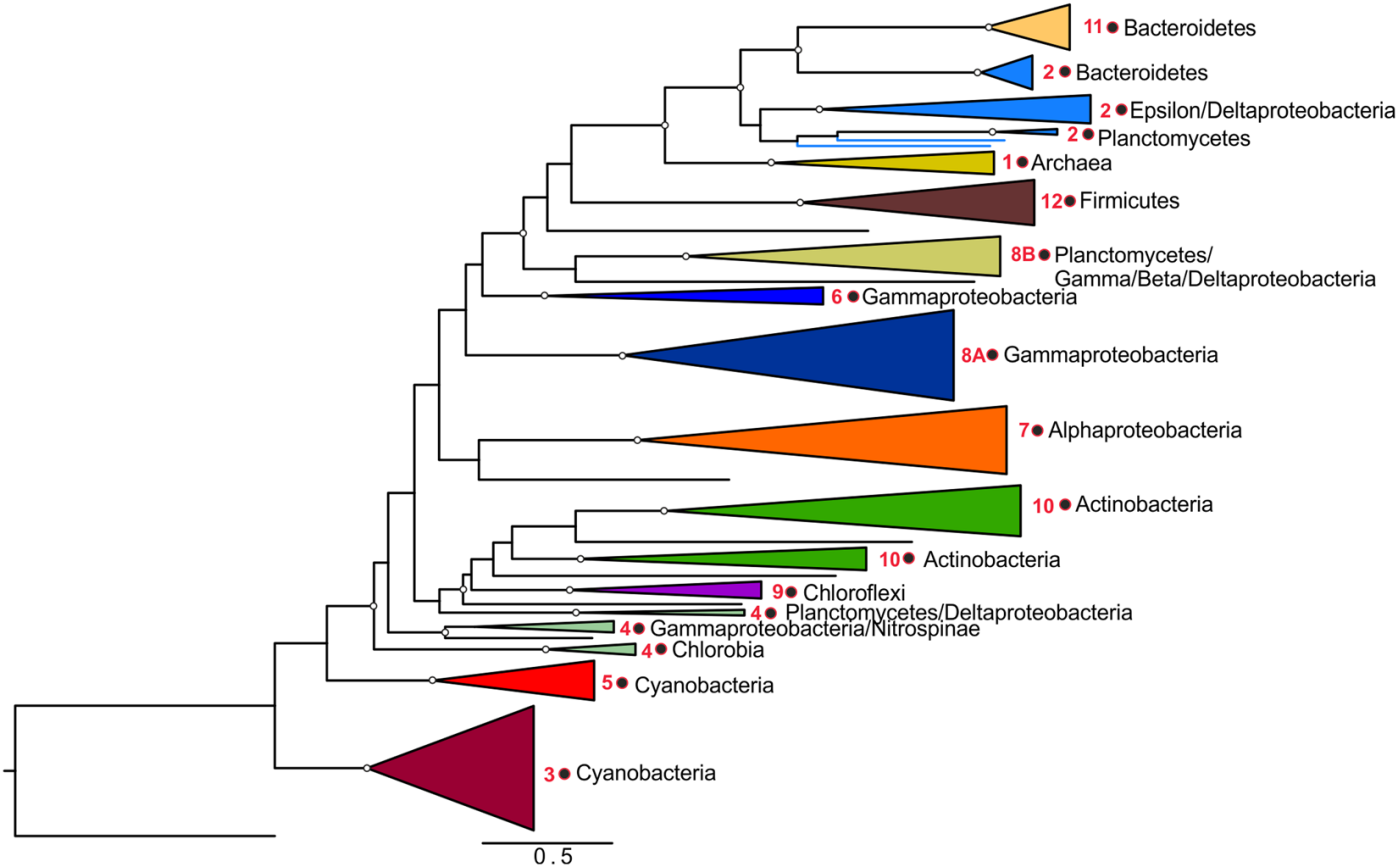
Phylogenetic tree of Cas1 associated with RTs. The phylogenetic reconstruction was carried out with 148 Cas1 proteins. The identified clades were named and colored according to the RT-associated clade. FastTree support values ≥0.92 are indicated at the nodes. The Cas1 protein (unknown subtype) from *Arthropira platensis* (GI:479129287)^21^ was used as an outgroup.

The phylogeny of Cas1 proteins (the most conserved Cas protein) shows that those of the type III system are not monophyletic, instead being scattered throughout the Cas1 phylogenetic tree^21^. We investigated the phylogenetic relationships of this particular group of Cas1 proteins associated with RTs in CRISPR-Cas modules, looking for signatures of coevolution, by constructing a phylogenetic tree (Fig. 3) for 148 unique Cas1 sequences. The Cas1 phylogeny essentially matched that of the associated nearby or fused RT (clades 1, 3, 5, 6, 7, 8A, 8B, 9, 11 and 12), suggesting extensive coevolution. These two protein domains (RT and Cas1), thus, appear to have a common evolutionary history that may have led to coadaptation through a direct relationship between these two proteins (e.g. a physical interaction) within particular protein complexes, which need to be further investigated. Nevertheless, the Cas1 sequences corresponding to RT-CRISPR clades 2, 4, 8 and 10 are polyphyletic, and some of these clades (clades 2 and 4) are subdivided according to particular phyla. This implies that either the association of the RT and Cas1 is a more recent evolutionary event or that it has occurred on multiple occasions in these CRISPR-Cas modules.

The Cas1 proteins associated with RTs (fused or separate) clustered into two main lineages, one of which contained most of the sequences including the Cas1 from archaeal CRISPR-Cas modules carrying an RT, providing further support for its acquisition from bacteria. The other lineage contained Cas1 proteins from the CRISPR-Cas systems identified in the RT-CRISPR-3 clade, which is restricted to cyanobacteria. In this particular clade, in addition to the RT-Cas1 fusion, the CRISPR-Cas modules used to include a more distant *cas1* locus (Supplementary Table 1), but their phylogeny suggests that these two genes have no common ancestor (Supplementary Figure 2). Given the diversity of *cas1* loci within type III systems^21^, the existence of only two major lineages of Cas1 proteins associated with RTs suggests that these particular CRISPR-Cas systems may also be subject to functional constraints dependent on unknown features of the associated Cas1 protein subtype. Contrary to previous assumptions of a small number of origins for the RT and Cas1 association^10^, a comparison of the RT and Cas1 phylogenetic trees suggests that this association has occurred independently on a number of occasions.

The RT-CRISPR-3 and RT-CRISPR-5 clades, which are found only in cyanobacteria, have two independent evolutionary origins (Figs 1 and 3 and Supplementary Figure 2). The phylogenetic relationships between the various cyanobacterial clades and their morphological complexity remain largely unknown^32^. Cyanobacteria are classified into five subsections on the basis of their morphological complexity. The presence of representatives of all (subsection I, Chroococcales; subsection III; Oscillatoriales, subsection IV; Notococales, and subsection V, Stigonematales) but one (subsection II, Pleurocapsales) of the cyanobacterial subsections in both the RT-CRISPR-3 and RT-CRISPR-5 clades suggests that these immunogenic systems are widespread among cyanobacteria of various developmental patterns and complexities. However, none of the five full genome sequences available (*Stanieria cyanosphera*, *Myxosarcina* sp. Gl1, *Pleurocapsa* sp. PCC 7327, *Pleurocapsa* sp. 7319 and *Xenococcus* sp. PCC7305) from subsection II (Pleurocapsales), corresponding to unicellular coccoids reproducing by multiple fission events to generate small cells (baeocytes), harbor genes encoding RTs associated with CRISPR-Cas systems. The significance of these findings is currently uncertain.

There is increasing evidence that type III CRISPR-Cas systems both act as RNases and target RNA-activated DNA nucleases, and that some of these systems have acquired RTs during evolution, but little else is known about the evolution and developmental mechanisms of these CRISPR-Cas systems. Indeed, only one representative of clade 8A, as defined here, has been studied^10^. The comprehensive phylogenetic analysis of these immunogenic systems and their classification, as presented here, extend our understanding of the association of RTs with type III CRIPSR-Cas systems and provide a basis for further studies.

## Methods

### Compilation of RTs associated to CRISPR-Cas systems

A flow-chart picture showing the different steps performed for the compilation of RTs associated to CRISPR-Cas systems and the generation of the data set is shown in Supplementary Figure 3. Makarova and coworkers21 annotated 38 sequences corresponding to RTs in the neighborhood of CRISPR-Cas loci. We found that the RT associated with a CRISPR-Cas system in *Herpetosiphon aurantiacus* (GI: 159898445) had a secondary structure typical of group II introns (not shown), and phylogenetic analyses indicated that this RT belonged to the group II intron ORF ML class (Fig. 1), whereas that of *Haliscomenobacter hydrossis* (GI: 332661943) was clearly identified in phylogenetic analyses as a retron/retron-like RT (not shown) with close relatives not associated with CRISPR-Cas systems bearing the retron-specific conserved motif (VTG) within the RT domain 7^7^. These RT sequences may therefore correspond to recent retrotranspositions of mobile elements. We also considered the only RT associated with a CRISPR-Cas system to have been identified to date in archaea (*Methanomethylovorans hollandica* DSM15978)^21^. We searched for close relatives of this archaeal RT, by searching the 262 complete archaeal genome sequences available from the PATRIC platform^28^. We retrieved 120 sequences annotated as retron-type RNA-directed DNA polymerases (EC2.7.7.49), and, after the elimination of duplicates, the unique archaeal RTs (46 sequences) with ≥200 amino-acid residues displaying ≤85% identity^7^ (to remove closer relatives) were aligned with a RT dataset (RT 0–7 domains) of 742 sequences described in a previous study^7^. This preliminary phylogenetic analysis (not shown) identified seven sequences, including the *M*. *hollandica* RT described above, clustered in a well-supported clade. These RTs did not have a recognizable group II intron secondary structure, and the CRISPR recognition tool implemented in Geneious Pro software (Biomatters Ltd.)^33, 34^ showed that five of them were associated with CRISPR arrays. We searched for possible bacterial members of the RT-CRISPR-1 archaeal clade by performing a BLAST search of 3043 annotated RTs with ≥200 amino-acid residues from 6291 complete bacterial genome sequences available from the PATRIC platform^28^ against a local database of RT consensus sequences from the various RT-CRISPR clades (Supplementary Figure 1). The significant *e*-values for archaeal RTs within the abovementioned clade were in the range of 9.22e-97 to 1.39e-127. None of the 3043 sequences analyzed had an *e*-value ≤ e-75.

A comprehensive dataset of RTs associated with CRISPR-Cas systems was then generated from the 38 RTs associated with CRISPR-Cas systems identified by Makarova and coworkers^21^, the seven archaeal RTs, 14 RTs associated with CRISPR-Cas systems reported in our previous RT survey^7^, and 157 protein sequences obtained from the National Center for Biotechnology Information (NCBI) Conserved Domain Architecture Retrieval Tool (CDART; 24^th^-Oct 2016) on the basis of the presence of both a Cas1 domain (pfam01867) and a RT domain of any origin (pfam00087). Using the indicated size and percent identity cutoffs (≥200 amino acid residues displaying ≤85% identity), we reduced the dataset to 118 unique RT sequences associated with CRISPR-Cas systems (Supplementary Table 2).

### RT sequences phylogenetic analysis

The above 118 sequences encompassing RT domains (RT 0–7 domains) were aligned (250 positions), with MUSCLE^35^ software, against 414 RT sequences representative of group II introns, three RT-like sequences from the closely related G2L4 group^7^ and two RT sequences from archaea related to the archaeal RTs associated with CRISPR-Cas systems (Supplementary Data 1). A phylogenetic tree was constructed with the FastTree program^36^ and the WAG evolutionary model, using pseudocounts (recommended for sequences containing large numbers of gaps) and a discrete gamma model with 20 rate categories. The clades were assigned to the inner nodes showing a high local support value (≥0.92), and subclades were assigned when a large number of sequences were restricted to particular phyla.

### Identification of CRISPR-*cas* loci

For CRISPR-*cas* loci identification, we retrieved the genomic neighborhoods (up to 50 kb in some cases) of the RTs included in our final dataset. In most cases, there was a CRISPR array in close proximity (less than 1 kb) to the RT gene identified. CRISPR array sites were identified and classified, and their orientation and properties were determined with the CRISPRDetect^37^ and CRISPRstrand^38^ algorithms. The correct orientation of the array was determined on the basis of the following criteria: (*i*) orientation predicted by the CRISPR-detect algorithm with a score of H or M if the flanking region of the array was available (>200 nt), (*ii*) for scores of L or NA, orientation was determined on the basis of the presence of direct repeats (DR) in the CRISPRstrand database, and (*iii*) DR similarities between arrays of other members of the group with a predicted orientation.

*Cas* genes in the neighborhood were identified as encoding proteins belonging to one of the 93 distinct Cas protein families, by BLAST searches with a consensus sequence from 395 profiles described in a previous study^21^. We annotated all the protein-coding genes present in the genomic region containing the RT gene and flanked by annotated coding sequences displaying sequence identity to characterized protein-coding sequences other than *cas* genes. An *e*-value threshold of 0.01 was used, except for subtype specificity, for which an *e*-value threshold of 10^−6^ was used. The genomic regions containing all the identified *cas* genes and CRISPR arrays were extracted and the region carrying the RT was trimmed to the first and last *cas* gene and/or the CRISPR array carrying intervening sequences of less than 5 kb in length (Supplementary Table 1).

### Cas1 sequences phylogenetic analysis

For generation of the RT-associated Cas1 protein phylogenetic tree, unique Cas 1 sequences that were either separate or fused to a RT (148 sequences) were aligned (329 positions), with MUSCLE (Supplementary Data 2), against the Cas1 protein (unknown subtype) from *Arthropira platensis* (GI:479129287)^21^ used as an outgroup. The phylogenetic tree was reconstructed with FastTree, as described above. Similarly, a phylogenetic tree was constructed by adding to the above alignment nine Cas1 sequences separate from the RT-Cas1 fusion in CRISPR-Cas modules from the RT-CRISPR-3 clade (Supplementary Figure 2).

## Electronic supplementary material

Supplementary information
