# Supplementary material for: The Reverse Transcriptases Associated with CRISPR-Cas Systems
Source: Sci Rep. 2017 Aug 2;7:7089. doi: 10.1038/s41598-017-07828-y (PMC5541045; doi:10.1038/s41598-017-07828-y)
Supplement: Supplementary file 1 — Supplementary information [file 41598_2017_7828_MOESM1_ESM.pdf]

The Reverse Transcriptases Associated with CRISPR-Cas Systems

Nicolás Toro*, Francisco Martínez-Abarca and Alejandro González-Delgado

Structure, Dynamics and Function of Rhizobacterial Genomes, Grupo de Ecología Genética de la Rizosfera, Department of Soil Microbiology and Symbiotic Systems, Estación Experimental del Zaidín, Consejo Superior de Investigaciones Científicas, C/ Profesor Albareda 1, 18008 Granada, Spain.

Correspondence and requests for materials should be addressed to N.T. (e-mail: nicolas.toro@eez.csic.es)

Supplementary information:

1. Supplementary Figure 1
2. Supplementary Figure 2
3. Supplementary Figure 3
4. Supplementary Table 1
5. Supplementary Table 2
6. Supplementary Data 1
7. Supplementary Data 2

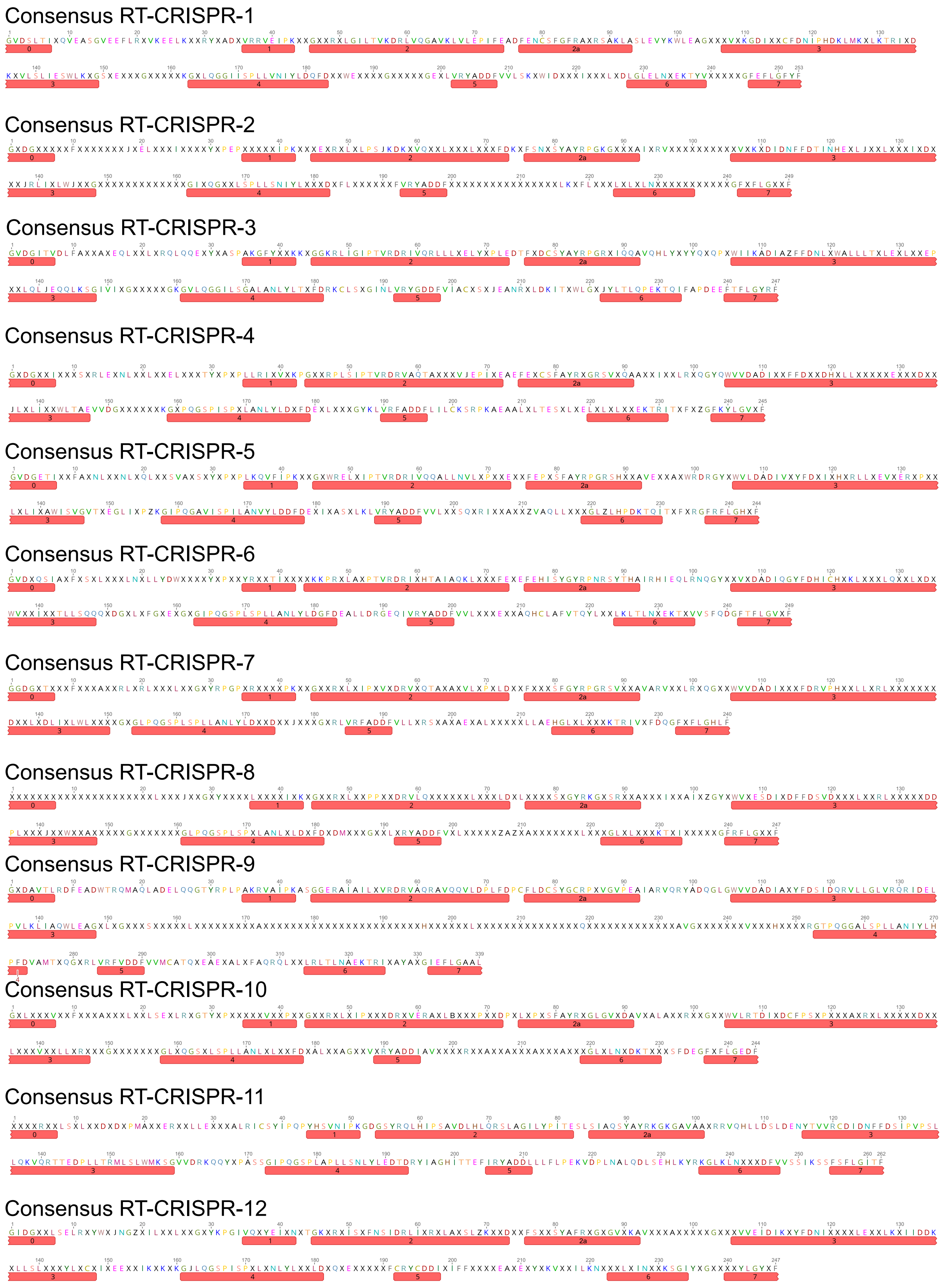

Supplementary Figure 1. RT consensus sequences for each of the RT-CRISPR clades. the amino acids encompassing the RT 0-7 domain are indicated.

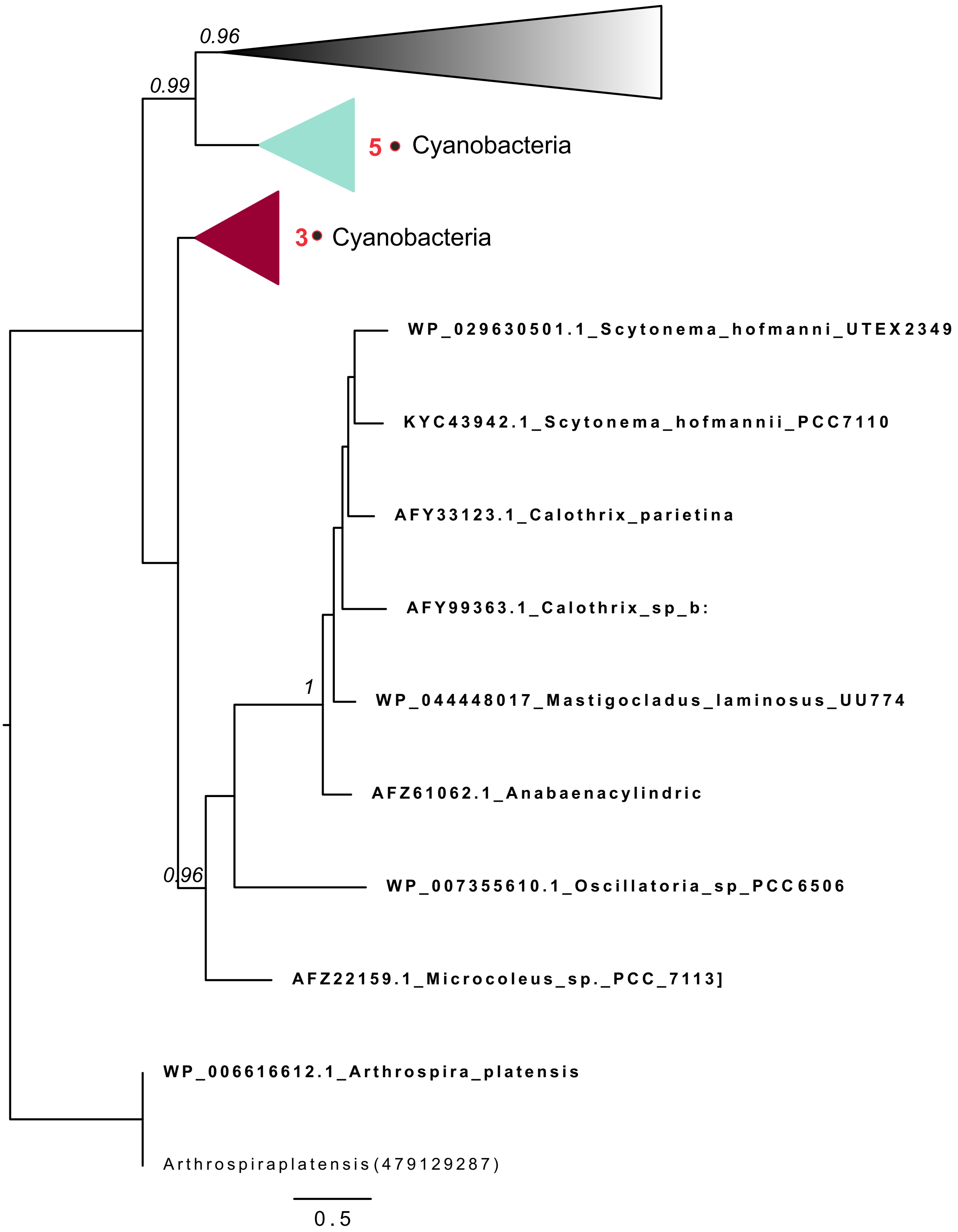

Supplementary Figure 2. Cas1 phylogenetic tree inferred by addition of 9 Cas1 sequences present separately from the RT-Cas1 fusion in the CRISPR-Cas modules from the RT-CRISPR-3 clade to the alignment used to infer the Cas1 phylogenetic tree shown in Fig. 3. FastTree support values ≥ 0.92 are indicated at the nodes. For the sake of symplicity, Cas1 clades were collapsed.

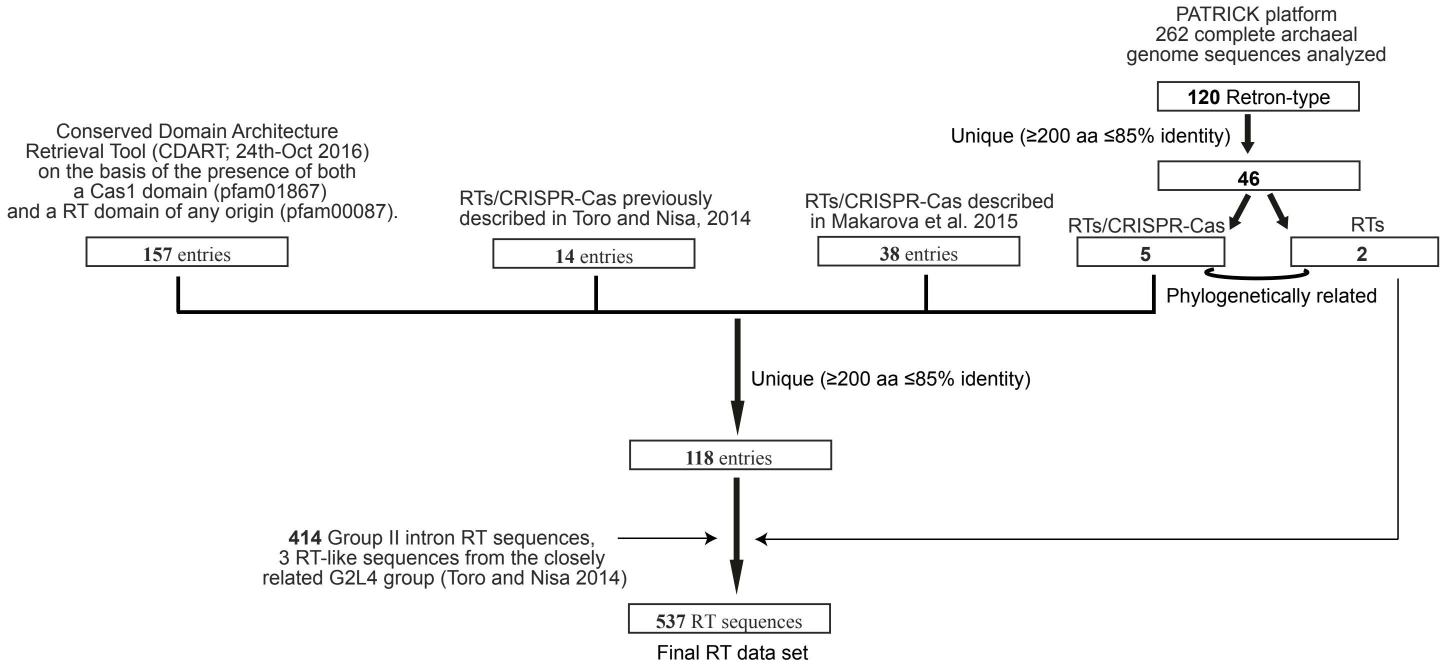

Supplementary Figure 3. Flow-chart picture showing the different steps performed for the compilation of RTs associated to CRISPR-Cas systems and the generation of the data set.

Supplementary Table 1. The list of all cas and RT loci, CRISPR arrays, profiles and *cas* gene names assigned to each gene in the locus corresponding to each RT-CRISPR clade

(a) Different CRISPR Loci/ III subtypes (shaded in green) are defined by a Profile-based CRISP-*cas* loci classification as previously described[21]. Partial is indicated when loci do not contain a full complement of effector module genes. Unknown is indicated when the sequence available comes from a single contig.
(b) Genomic coordinates of the genes and CRISPR-arrays.
(c) Current annotation of the corresponding CDS. (H) Hipothetical protein.
(d) Definition of the genes present in CRISPR loci using the Makarova consensus profiles[21]. E-value threshold of 0.01 are considered. E-value threshold of 10-6 are indicated in bold. Only genes in bold are considered to define the subtype-specifity.
(e) Identification of the CRISPR array using Geneious. Final orientation has been defined as specified in Methods section.
(f) Structural classification of CRISPR array based on tools previously reported[38].
(g) Orientation confidence (H, high; M; medium and L, low) and characteristics of repeat units were identified using http://brownlabtools.otago.ac.nz/CRISPRDetect/predict_crispr_array.html

RT-CRISPR-1 (CAS-III-A/D, CAS-III-C)
Archaea (Methanosarcinales)

[a](Sub) Type/[b]Coordinates	Strand	Protein_id	Genome Partition ID	Strain	Domain	Phylum	Lineage (class)	Order	Family	Genus	[c]Cas gene (annotated)	[d]Cas gene/[e]Array (identified)	[d]Profiles	[d](Sub)Type-specificity	[e]Spacers (length)	[e]Repeats (length)	[f]Str.motif/Seq.family/Superclass	[f]Seq Repeats	[g]Orientation Confidence
CAS-III-C																			
1145732 -> 1144393	-		CP003362	Methanomethylovorans hollandica DSM15978	Archaea	Euryarcheota	Methanomicrobia	Methanosarcinales	Methanosarcinaceae	Methanomethylovorans		CRISPR array			17 (34-66)	18 (37)	M9/F10/E	CTTTCAGAGCATGATCCACTAAAACAAGGATTGAAAC	H
1146077 -> 1146202	+	AGB49371.1	CP003362	Methanomethylovorans hollandica DSM15978	Archaea	Euryarcheota	Methanomicrobia	Methanosarcinales	Methanosarcinaceae	Methanomethylovorans	H								
1146204 -> 1146857	+	AGB49372.1	CP003362	Methanomethylovorans hollandica DSM15978	Archaea	Euryarcheota	Methanomicrobia	Methanosarcinales	Methanosarcinaceae	Methanomethylovorans	H	**Cas6**	cls000004	CAS-I, CAS-III					
1146848 -> 1147816	+	AGB49373.1	CP003362	Methanomethylovorans hollandica DSM15978	Archaea	Euryarcheota	Methanomicrobia	Methanosarcinales	Methanosarcinaceae	Methanomethylovorans	CRISPR-associated endonuclease Cas1	**Cas1**	cd09634	CAS-I, CAS-II, CAS-III, CASV					
1147829 -> 1148119	+	AGB49374.1	CP003362	Methanomethylovorans hollandica DSM15978	Archaea	Euryarcheota	Methanomicrobia	Methanosarcinales	Methanosarcinaceae	Methanomethylovorans	CRISPR-associated endonuclease Cas2	**Cas2**	mkCas0128	CAS-I, CAS-II, CAS-III					
1148128 -> 1148739	+	AGB49375.1	CP003362	Methanomethylovorans hollandica DSM15978	Archaea	Euryarcheota	Methanomicrobia	Methanosarcinales	Methanosarcinaceae	Methanomethylovorans	CRISPR-associated endonuclease Cas4	**Cas4**	cd09637	CAS-I, CAS-II, CASV					
1149180 -> 1148923	-	AGB49376.1	CP003362	Methanomethylovorans hollandica DSM15978	Archaea	Euryarcheota	Methanomicrobia	Methanosarcinales	Methanosarcinaceae	Methanomethylovorans	H								
1149272 -> 1149838	+		CP003362	Methanomethylovorans hollandica DSM15978	Archaea	Euryarcheota	Methanomicrobia	Methanosarcinales	Methanosarcinaceae	Methanomethylovorans		CRISPR array			7 (34-52)	8 (37)	M9/F10/E	ATTCGAGAGCAAGATCCACTATAACAAGGATTGAAAC	H
1149913 -> 1151103	+	AGB49377.1	CP003362	Methanomethylovorans hollandica DSM15978	Archaea	Euryarcheota	Methanomicrobia	Methanosarcinales	Methanosarcinaceae	Methanomethylovorans	Retron-type reverse transcriptase	**RT**	pfam00078	CAS-III					
1151201 -> 1151485	+	AGB49378.1	CP003362	Methanomethylovorans hollandica DSM15978	Archaea	Euryarcheota	Methanomicrobia	Methanosarcinales	Methanosarcinaceae	Methanomethylovorans	H								
1151568 -> 1152536	+	AGB49379.1	CP003362	Methanomethylovorans hollandica DSM15978	Archaea	Euryarcheota	Methanomicrobia	Methanosarcinales	Methanosarcinaceae	Methanomethylovorans	CRISPR type III-B_RAMP module RAMP protein Cmr1	Cmr1gr7	cd09657						
1152533 -> 1153417	+	AGB49380.1	CP003362	Methanomethylovorans hollandica DSM15978	Archaea	Euryarcheota	Methanomicrobia	Methanosarcinales	Methanosarcinaceae	Methanomethylovorans	uncharacterized protein predicted to be involved in DNA repair (RAMP superfamily)	**Cmr6gr7**	mkCas0142	CAS-III-C					
1153428 -> 1156391	+	AGB49381.1	CP003362	Methanomethylovorans hollandica DSM15978	Archaea	Euryarcheota	Methanomicrobia	Methanosarcinales	Methanosarcinaceae	Methanomethylovorans	CRISPR-associated protein, Csx11 family	**Cas10**	cd09701, cd09701	CAS-III-C; CAS-III-C					
1156394 -> 1157389	+	AGB49382.1	CP003362	Methanomethylovorans hollandica DSM15978	Archaea	Euryarcheota	Methanomicrobia	Methanosarcinales	Methanosarcinaceae	Methanomethylovorans	CRISPR type III-B_RAMP module RAMP protein Cmr4	**Cmr4gr7**	COG1336	CAS-III-C					
1157392 -> 1157760	+	AGB49383.1	CP003362	Methanomethylovorans hollandica DSM15978	Archaea	Euryarcheota	Methanomicrobia	Methanosarcinales	Methanosarcinaceae	Methanomethylovorans	H	**Cmr5gr11**	mkCas0060	CAS-III-C					
1157757 -> 1158647	+	AGB49383.1	CP003362	Methanomethylovorans hollandica DSM15978	Archaea	Euryarcheota	Methanomicrobia	Methanosarcinales	Methanosarcinaceae	Methanomethylovorans	H	**Cmr3gr5**	mkCas0067	CAS-III-C					
CAS-III-C																			
2759740 -> 2758832	-	AKB36818.1	CP009508	Methanosarcina siciliae C2J	Archaea	Euryarcheota	Methanomicrobia	Methanosarcinales	Methanosarcinaceae	Methanosarcina	DUF324 domain containing Cmr6-like protein	**Cmr3gr5**	mkCas0067	CAS-III-C					
2760938 -> 2760135	-	AKB36819.1	CP009508	Methanosarcina siciliae C2J	Archaea	Euryarcheota	Methanomicrobia	Methanosarcinales	Methanosarcinaceae	Methanosarcina	CRISPR-associated RAMP Cmr4	**Cmr4gr7**	COG1336	CAS-III-C					
2764092 -> 2761117	-	AKB36820.1	CP009508	Methanosarcina siciliae C2J	Archaea	Euryarcheota	Methanomicrobia	Methanosarcinales	Methanosarcinaceae	Methanosarcina	CRISPR-associated protein Csx11	**Cas10**	cd09701, cd09701	CAS-III-C; CAS-III-C					
2764993 -> 2764073	-	AKB36821.1	CP009508	Methanosarcina siciliae C2J	Archaea	Euryarcheota	Methanomicrobia	Methanosarcinales	Methanosarcinaceae	Methanosarcina	DUF324 domain containing Cmr2-like protein	**Cmr6gr7**	mkCas0142	CAS-III-C					
2765970 -> 2764993	-	AKB36822.1	CP009508	Methanosarcina siciliae C2J	Archaea	Euryarcheota	Methanomicrobia	Methanosarcinales	Methanosarcinaceae	Methanosarcina	CRISPR-associated RAMP Cmr1	Cmr1gr7	cd09657						
2766984 -> 2766241	-	AKB36823.1	CP009508	Methanosarcina siciliae C2J	Archaea	Euryarcheota	Methanomicrobia	Methanosarcinales	Methanosarcinaceae	Methanosarcina	H	**Cas6**	cls000004	CAS-I, CAS-III					
2768339 -> 2767107	-	AKB36824.1	CP009508	Methanosarcina siciliae C2J	Archaea	Euryarcheota	Methanomicrobia	Methanosarcinales	Methanosarcinaceae	Methanosarcina	H	**RT**	pfam00078	CAS-III					
2769988 -> 2768489	-		CP009508	Methanosarcina siciliae C2J	Archaea	Euryarcheota	Methanomicrobia	Methanosarcinales	Methanosarcinaceae	Methanosarcina		CRISPR array			20 (34-38)	21(37)	NA/NA/F	CTAAAACAGCAACAGCCACTAAAACAAGGATTGAAAC	H
2770555 -> 2771604	+	AKB36825.1	CP009508	Methanosarcina siciliae C2J	Archaea	Euryarcheota	Methanomicrobia	Methanosarcinales	Methanosarcinaceae	Methanosarcina	CRISPR-associated protein Cas1	**Cas1**	cd09634	CAS-I, CAS-II, CAS-III, CASV					
2771659 -> 2771964	+	AKB36826.1	CP009508	Methanosarcina siciliae C2J	Archaea	Euryarcheota	Methanomicrobia	Methanosarcinales	Methanosarcinaceae	Methanosarcina	CRISPR-associated protein Cas2	**Cas2**	COG1343	CAS-I, CAS-II, CAS-III					
CAS-III-D																			
2380972 -> 2380115	-	AKB78435.1	CP009516	Methanosarcina Honorobensis HB-1	Archaea	Euryarcheota	Methanomicrobia	Methanosarcinales	Methanosarcinaceae	Methanosarcina	H	csx1	cd09702						
2381286 -> 2380996	-	AKB78436.1	CP009516	Methanosarcina Honorobensis HB-1	Archaea	Euryarcheota	Methanomicrobia	Methanosarcinales	Methanosarcinaceae	Methanosarcina	CRISPR-associated protein, Csx3 family	**csx3**	cd09681	CAS-I, CAS-III					
2382040 -> 2381363	-	AKB78437.1	CP009516	Methanosarcina Honorobensis HB-1	Archaea	Euryarcheota	Methanomicrobia	Methanosarcinales	Methanosarcinaceae	Methanosarcina	H	**Cas6**	cls000004	CAS-I, CAS-III					
2383970 -> 2382105	-	AKB78438.1	CP009516	Methanosarcina Honorobensis HB-1	Archaea	Euryarcheota	Methanomicrobia	Methanosarcinales	Methanosarcinaceae	Methanosarcina	DUF324 domain containing protein	**Csm3gr7**	cls000253	CAS-III-A, CAS-III-D					
2384617 -> 2383976	-	AKB78439.1	CP009516	Methanosarcina Honorobensis HB-1	Archaea	Euryarcheota	Methanomicrobia	Methanosarcinales	Methanosarcinaceae	Methanosarcina	H	**Csx19**	cls001376	CAS-III-D					
2386101 -> 2384614	-	AKB78440.1	CP009516	Methanosarcina Honorobensis HB-1	Archaea	Euryarcheota	Methanomicrobia	Methanosarcinales	Methanosarcinaceae	Methanosarcina	DUF324 domain containing protein	**Csm3gr7**	cd09683, cd09683	CAS-III-A, CAS-III-D; CAS-III-A, CAS-III-D					
2387858 -> 2386101	-	AKB78441.1	CP009516	Methanosarcina Honorobensis HB-1	Archaea	Euryarcheota	Methanomicrobia	Methanosarcinales	Methanosarcinaceae	Methanosarcina	H	**Csx10gr5**	cd09700	CAS-III-D					
2388525 -> 2387860	-	AKB78442.1	CP009516	Methanosarcina Honorobensis HB-1	Archaea	Euryarcheota	Methanomicrobia	Methanosarcinales	Methanosarcinaceae	Methanosarcina	DUF324 domain containing protein	**Csm3gr7**	mkCas0198	CAS-III-A,CAS-III-D					
2390179 -> 2388512	-	AKB78443.1	CP009516	Methanosarcina Honorobensis HB-1	Archaea	Euryarcheota	Methanomicrobia	Methanosarcinales	Methanosarcinaceae	Methanosarcina	H	**Cas10**	mkCas0133	CAS-III					
2391075 -> 2390269	-	AKB78444.1	CP009516	Methanosarcina Honorobensis HB-1	Archaea	Euryarcheota	Methanomicrobia	Methanosarcinales	Methanosarcinaceae	Methanosarcina	H	csx1	cd09702						
2392416 -> 2391283	-	AKB78445.1	CP009516	Methanosarcina Honorobensis HB-1	Archaea	Euryarcheota	Methanomicrobia	Methanosarcinales	Methanosarcinaceae	Methanosarcina	H	**RT**	pfam00078	CAS-III					
2394112-> 2392664	-		CP009516	Methanosarcina Honorobensis HB-1	Archaea	Euryarcheota	Methanomicrobia	Methanosarcinales	Methanosarcinaceae	Methanosarcina		CRISPR array			19(35-46)	20(37)	NA/NA/E	GTCGAGAAGTAGCTTCCACTAAAATAAGGATTGAAAC	H
2394773 -> 2395729	+	AKB78446.1	CP009516	Methanosarcina Honorobensis HB-1	Archaea	Euryarcheota	Methanomicrobia	Methanosarcinales	Methanosarcinaceae	Methanosarcina	CRISPR-associated protein Cas1	**Cas1**	cd09634	CAS-I, CAS-II, CAS-III, CASV					
2395771 -> 2396076	+	AKB78447.1	CP009516	Methanosarcina Honorobensis HB-1	Archaea	Euryarcheota	Methanomicrobia	Methanosarcinales	Methanosarcinaceae	Methanosarcina	CRISPR-associated protein Cas2	**Cas2**	COG1343	CAS-I, CAS-II, CAS-III					
CAS-III-A																			
3708004 -> 3706511	-	AKB26310.1	CP009505	Methanosarcina sp. MTP4	Archaea	Euryarcheota	Methanomicrobia	Methanosarcinales	Methanosarcinaceae	Methanosarcina	H	**Csx1**	mkCas0109	CAS-III					
3711523 -> 3708017	-	AKB26311.1	CP009505	Methanosarcina sp. MTP4	Archaea	Euryarcheota	Methanomicrobia	Methanosarcinales	Methanosarcinaceae	Methanosarcina	H								
3712721 -> 3711525	-	AKB26312.1	CP009505	Methanosarcina sp. MTP4	Archaea	Euryarcheota	Methanomicrobia	Methanosarcinales	Methanosarcinaceae	Methanosarcina	CRISPR-associated protein, Csm5 family	**Csm5gr7**	cd09662	CAS-III-A					
3713725 -> 3712718	-	AKB26313.1	CP009505	Methanosarcina sp. MTP4	Archaea	Euryarcheota	Methanomicrobia	Methanosarcinales	Methanosarcinaceae	Methanosarcina	CRISPR-associated RAMP protein, Csm4 family	**Csm4gr5**	COG1567	CAS-III-A					
3714512 -> 3713718	-	AKB26314.1	CP009505	Methanosarcina sp. MTP4	Archaea	Euryarcheota	Methanomicrobia	Methanosarcinales	Methanosarcinaceae	Methanosarcina	CRISPR-associated RAMP Csm3	**Csm3gr7**	cd09684	CAS-III-A, CASIII-D					
3715031 -> 3714513	-	AKB26315.1	CP009505	Methanosarcina sp. MTP4	Archaea	Euryarcheota	Methanomicrobia	Methanosarcinales	Methanosarcinaceae	Methanosarcina	CRISPR-associated protein, Csm2 family	**Csm2gr11**	cd09647	CAS-III-A					
3717574 -> 3715031	-	AKB26316.1	CP009505	Methanosarcina sp. MTP4	Archaea	Euryarcheota	Methanomicrobia	Methanosarcinales	Methanosarcinaceae	Methanosarcina	CRISPR-associated protein, Csm1 family	**Cas10**	cd09680	CAS-III					
3718685 -> 3717699	-		CP009505	Methanosarcina sp. MTP4	Archaea	Euryarcheota	Methanomicrobia	Methanosarcinales	Methanosarcinaceae	Methanosarcina		CRISPR array			13(34-40)	14(37)	NA/NA/NA	GTTGCAAAACAGCTTCCAGCAAAACAAGGATTGAAAC	H
3719800 -> 3719129	-	AKB26317.1	CP009505	Methanosarcina sp. MTP4	Archaea	Euryarcheota	Methanomicrobia	Methanosarcinales	Methanosarcinaceae	Methanosarcina	H	**Cas6**	cls000004	CAS-I, CAS-III					
3721131 -> 3719863	-	AKB26318.1	CP009505	Methanosarcina sp. MTP4	Archaea	Euryarcheota	Methanomicrobia	Methanosarcinales	Methanosarcinaceae	Methanosarcina	Retron-type RNA-directed DNA polymerase	**RT**	pfam00078	CAS-III					
3722722 -> 3721596	-		CP009505	Methanosarcina sp. MTP4	Archaea	Euryarcheota	Methanomicrobia	Methanosarcinales	Methanosarcinaceae	Methanosarcina		CRISPR array			15(34-38)	16(37)	NA/NA/NA	GTTGCAAAACAGCTTCCAGCAAAACAAGGATTGAAAC	H
3723258 -> 3724265	+	AKB26319.1	CP009505	Methanosarcina sp. MTP4	Archaea	Euryarcheota	Methanomicrobia	Methanosarcinales	Methanosarcinaceae	Methanosarcina	CRISPR-associated protein Cas1	**Cas1**	pfam01867	CAS-I, CAS-II, CAS-III					
3724268 -> 3724570	+	AKB26320.1	CP009505	Methanosarcina sp. MTP4	Archaea	Euryarcheota	Methanomicrobia	Methanosarcinales	Methanosarcinaceae	Methanosarcina	CRISPR-associated protein Cas2	**Cas2**	mkCas0128	CAS-I, CAS-II, CAS-III					
CAS-III-A																			
2063044 -> 2058965	-	AKB21950.1	CP009504	Methanosarcina sp. WH1	Archaea	Euryarcheota	Methanomicrobia	Methanosarcinales	Methanosarcinaceae	Methanosarcina	H	**Csx1**	mkCas0109	CAS-III					
2064242 -> 2063070	-	AKB21951.1	CP009504	Methanosarcina sp. WH1	Archaea	Euryarcheota	Methanomicrobia	Methanosarcinales	Methanosarcinaceae	Methanosarcina	CRISPR-associated protein, Csm5 family	**Csm5gr7**	cd09662	CAS-III-A					
2065252 -> 2064239	-	AKB21952.1	CP009504	Methanosarcina sp. WH1	Archaea	Euryarcheota	Methanomicrobia	Methanosarcinales	Methanosarcinaceae	Methanosarcina	CRISPR-associated RAMP protein, Csm4 family	**Csm4gr5**	cd09663	CAS-III-A					
2066021 -> 2065245	-	AKB21953.1	CP009504	Methanosarcina sp. WH1	Archaea	Euryarcheota	Methanomicrobia	Methanosarcinales	Methanosarcinaceae	Methanosarcina	CRISPR-associated RAMP Csm3	**Csm3gr7**	cd09684	CAS-III-A, CASIII-D					
2066519 -> 2066022	-	AKB21954.1	CP009504	Methanosarcina sp. WH1	Archaea	Euryarcheota	Methanomicrobia	Methanosarcinales	Methanosarcinaceae	Methanosarcina	CRISPR-associated protein, Csm2 family	**Csm2gr11**	COG1421	CAS-III-A					
2069038 -> 2066516	-	AKB21955.1	CP009504	Methanosarcina sp. WH1	Archaea	Euryarcheota	Methanomicrobia	Methanosarcinales	Methanosarcinaceae	Methanosarcina	CRISPR-associated protein, Csm1 family	**Cas10**	cd09680	CAS-III					
2069707 -> 2069160	-		CP009504	Methanosarcina sp. WH1	Archaea	Euryarcheota	Methanomicrobia	Methanosarcinales	Methanosarcinaceae	Methanosarcina		CRISPR array			7 (34-38)	8 (37)	NA/NA/NA	GTTGAAAAACAGCTTCCAGCAAAACAAGGATTGAAAC	H
2070243 -> 2071250	+	AKB21956.1	CP009504	Methanosarcina sp. WH1	Archaea	Euryarcheota	Methanomicrobia	Methanosarcinales	Methanosarcinaceae	Methanosarcina	CRISPR-associated protein Cas1	**Cas1**	pfam01867	CAS-I, CAS-II, CAS-III					
2071253 -> 2071555	+	AKB21957.1	CP009504	Methanosarcina sp. WH1	Archaea	Euryarcheota	Methanomicrobia	Methanosarcinales	Methanosarcinaceae	Methanosarcina	CRISPR-associated protein Cas2	**Cas2**	mkCas0128	CAS-I, CAS-II, CAS-III					
2071591 -> 2072358	+	AKB21958.1	CP009504	Methanosarcina sp. WH1	Archaea	Euryarcheota	Methanomicrobia	Methanosarcinales	Methanosarcinaceae	Methanosarcina	H	Csx1	pfam9002						
2073287 -> 2072616	-	AKB21959.1	CP009504	Methanosarcina sp. WH1	Archaea	Euryarcheota	Methanomicrobia	Methanosarcinales	Methanosarcinaceae	Methanosarcina	H	**Cas6**	cls000004	CAS-I, CAS-III					
2074551 -> 2073346	-	AKB21960.1	CP009504	Methanosarcina sp. WH1	Archaea	Euryarcheota	Methanomicrobia	Methanosarcinales	Methanosarcinaceae	Methanosarcina	Retron-type RNA-directed DNA polymerase	**RT**	pfam00078	CAS-III					
2076588 -> 2075017	-		CP009504	Methanosarcina sp. WH1	Archaea	Euryarcheota	Methanomicrobia	Methanosarcinales	Methanosarcinaceae	Methanosarcina		CRISPR array			21(33-40)	22(37)	NA/NA/NA	GTTGAAAAACAGCTTCCAGCAAAACAAGGATTGAAAC	H
2077124 -> 2077771	+	AKB21962.1	CP009504	Methanosarcina sp. WH1	Archaea	Euryarcheota	Methanomicrobia	Methanosarcinales	Methanosarcinaceae	Methanosarcina	CRISPR-associated protein Cas1	**Cas1**	cd09634	CAS-I, CAS-II, CAS-III, CASV					

RT-CRISPR-2 (CAS-III-A/D, CAS-III-B)
Planctomycetia (Candidatus Brocardiales), Bacteroidetes (Bacteroidales), (Delta, Epsilon) Proteobacteria (Syntrophobacterales, Desulfurellales, Desulfobacterales; Campylobacterales),

[a](Sub) Type/[b]Coordinates	Strand	Protein_id	Genome Partition ID	Strain	Domain	Phylum	Lineage (class)	Order	Family	Genus	[c]Cas gene (annotated)	[d]Cas gene/[e]Array (identified)	[d]Profiles	[d](Sub)Type-specificity	[e]Spacers (length)	[e]Repeats (length)	[f]Str.motif/Seq.family/Superclass	[f]Seq Repeats	[g]Orientation Confidence
CAS-III-A/CAS-III-D																			
3067046 ->3066547	-		NC_015388.1	Desulfobacca acetoxidans DSM 11109	Bacteria	Proteobacteria	Deltaproteobacteria	Syntrophobacterales	Syntrophaceae	Desulfobacca		CRISPR Array			6 (36-44)	7 (37)	NA/F11/E	CTCTGCTTCATTGACCTGATTACGAAGGGATTGCGAC	M
3070207 ->3067565	-	WP_013707702.1	NC_015388.1	Desulfobacca acetoxidans DSM 11109	Bacteria	Proteobacteria	Deltaproteobacteria	Syntrophobacterales	Syntrophaceae	Desulfobacca	CRISPR-associated endonuclease Cas1	**RT-Cas1**	pfam00078, cd09634	CAS-III; CAS-I, CAS-II, CAS-III, CAS-V					
3071408 ->3070281	-	WP_013707703.1	NC_015388.1	Desulfobacca acetoxidans DSM 11109	Bacteria	Proteobacteria	Deltaproteobacteria	Syntrophobacterales	Syntrophaceae	Desulfobacca	H								
3071920 ->3071405	-	WP_013707704.1	NC_015388.1	Desulfobacca acetoxidans DSM 11109	Bacteria	Proteobacteria	Deltaproteobacteria	Syntrophobacterales	Syntrophaceae	Desulfobacca	H								
3073881 ->3072019	-	WP_013707705.1	NC_015388.1	Desulfobacca acetoxidans DSM 11109	Bacteria	Proteobacteria	Deltaproteobacteria	Syntrophobacterales	Syntrophaceae	Desulfobacca	CRISPR-associated RAMP family protein	**Csm3gr7**	cls000253	CAS-III-A, CAS-III-D					
3075111 ->3073900	-	WP_013707706.1	NC_015388.1	Desulfobacca acetoxidans DSM 11109	Bacteria	Proteobacteria	Deltaproteobacteria	Syntrophobacterales	Syntrophaceae	Desulfobacca	H								
3075835 ->3075104	-	WP_013707707.1	NC_015388.1	Desulfobacca acetoxidans DSM 11109	Bacteria	Proteobacteria	Deltaproteobacteria	Syntrophobacterales	Syntrophaceae	Desulfobacca	CRISPR-associated protein	**Csm6**	pfam09651	CAS-III-A					
3077443 ->3075854	-	WP_013707708.1	NC_015388.1	Desulfobacca acetoxidans DSM 11109	Bacteria	Proteobacteria	Deltaproteobacteria	Syntrophobacterales	Syntrophaceae	Desulfobacca	H	**Csm3gr7**	cls000253, cls000253	CAS-III-A, CAS-III-D; CAS-III-A, CAS-III-D					
3078003 ->3077440	-	WP_013707709.1	NC_015388.1	Desulfobacca acetoxidans DSM 11109	Bacteria	Proteobacteria	Deltaproteobacteria	Syntrophobacterales	Syntrophaceae	Desulfobacca	H	Csx19	mkCas0188						
3079049 ->3078000	-	WP_013707710.1	NC_015388.1	Desulfobacca acetoxidans DSM 11109	Bacteria	Proteobacteria	Deltaproteobacteria	Syntrophobacterales	Syntrophaceae	Desulfobacca	H	**Csm3gr7**	cd09683	CAS-III-A, CAS-III-D					
3079888 ->3079064	-	WP_013707711.1	NC_015388.1	Desulfobacca acetoxidans DSM 11109	Bacteria	Proteobacteria	Deltaproteobacteria	Syntrophobacterales	Syntrophaceae	Desulfobacca	CRISPR-associated RAMP protein	**Csm3gr7**	cd09683	CAS-III-A, CAS-III-D					
3080427 ->3079885	-	WP_013707712.1	NC_015388.1	Desulfobacca acetoxidans DSM 11109	Bacteria	Proteobacteria	Deltaproteobacteria	Syntrophobacterales	Syntrophaceae	Desulfobacca	H								
3081596 ->3080424	-	WP_013707713.1	NC_015388.1	Desulfobacca acetoxidans DSM 11109	Bacteria	Proteobacteria	Deltaproteobacteria	Syntrophobacterales	Syntrophaceae	Desulfobacca	CRISPR-associated RAMP Csx10 family protein	**Csx10gr5**	cls000715	CAS-III-D					
3082230 ->3081607	-	WP_013707714.1	NC_015388.1	Desulfobacca acetoxidans DSM 11109	Bacteria	Proteobacteria	Deltaproteobacteria	Syntrophobacterales	Syntrophaceae	Desulfobacca	H	**Csm3gr7**	COG1337	CAS-III-D					
3083741 ->3082233	-	WP_013707715.1	NC_015388.1	Desulfobacca acetoxidans DSM 11109	Bacteria	Proteobacteria	Deltaproteobacteria	Syntrophobacterales	Syntrophaceae	Desulfobacca	H	**Cas10**	mkCas0133	CAS-III					
Unknown																			
582 ->831	ND		LAQJ01000315.1	Candidatus Brocadia fulgida RU1 BROFUL	Bacteria	Planctomycetes	Planctomycetia	Candidatus Brocardiales	Candidatus Brocardiacea	Candidatus Brocadia		CRISPR Array			2(40,93)	3 (39)	M4/NA/F	GGCGTTCCCTGCTCTTTGAAGAGATGAAAGGGATTGAC	NA
1476->3425	+	KKO17867.1	LAQJ01000315.1	Candidatus Brocadia fulgida RU1 BROFUL	Bacteria	Planctomycetes	Planctomycetia	Candidatus Brocardiales	Candidatus Brocardiacea	Candidatus Brocadia	CRISPR-associated protein	**RT-Cas1**	pfam00078, pfam01867	CAS-III; CAS-I, CAS-III					
3447->3791	+	KKO17868.1	LAQJ01000315.1	Candidatus Brocadia fulgida RU1 BROFUL	Bacteria	Planctomycetes	Planctomycetia	Candidatus Brocardiales	Candidatus Brocardiacea	Candidatus Brocadia	CRISPR-associated protein	**Cas1**	cd09634	CAS-I, CAS-II, CAS-III, CAS-V					
3879->4154	+	KKO17869.1	LAQJ01000315.1	Candidatus Brocadia fulgida RU1 BROFUL	Bacteria	Planctomycetes	Planctomycetia	Candidatus Brocardiales	Candidatus Brocardiacea	Candidatus Brocadia	H	**Cas2**	mkCas0081	CAS-I, CAS-II, CAS-III					
Partial CAS-III-A/CAS-III-D																			
11266->16419	+	KHE91659.1	JRYO01000185.1	Candidatus Scalindua brodae RU1 SCABRO	Bacteria	Planctomycetes	Planctomycetia	Candidatus Brocardiales	Candidatus Brocardiacea	Candidatus Scalindua	RAMP superfamily protein	**Csm3gr7**	cls000253, cls000253	CAS-III-A, CAS-III-D					
16358->16567	+	KHE91658.1	JRYO01000185.1	Candidatus Scalindua brodae RU1 SCABRO	Bacteria	Planctomycetes	Planctomycetia	Candidatus Brocardiales	Candidatus Brocardiacea	Candidatus Scalindua	stationary phase/starvation inducible regulatoryprotein CspD								
17098->17938	+		JRYO01000185.1	Candidatus Scalindua brodae RU1 SCABRO	Bacteria	Planctomycetes	Planctomycetia	Candidatus Brocardiales	Candidatus Brocardiacea	Candidatus Scalindua		CRISPR Array			11 (34-41)	12 (36)	M4/NA/F	GTTATGAAACAAGAGAAGGACTTAATGTCACGGTAC	H
18163->20469	+	KHE91657.1	JRYO01000185.1	Candidatus Scalindua brodae RU1 SCABRO	Bacteria	Planctomycetes	Planctomycetia	Candidatus Brocardiales	Candidatus Brocardiacea	Candidatus Scalindua	CRISPR-associated protein	**RT-cas1**	pfam00078, COG1518	CAS-III; CAS-I, CAS-II, CAS-III					
20754->20557	-	KHE91656.1	JRYO01000185.1	Candidatus Scalindua brodae RU1 SCABRO	Bacteria	Planctomycetes	Planctomycetia	Candidatus Brocardiales	Candidatus Brocardiacea	Candidatus Scalindua	H								
20816->21091	+	KHE91655.1	JRYO01000185.1	Candidatus Scalindua brodae RU1 SCABRO	Bacteria	Planctomycetes	Planctomycetia	Candidatus Brocardiales	Candidatus Brocardiacea	Candidatus Scalindua	H	**Cas2**	mkCas0081	CAS-I, CAS-II, CAS-III					
Partial CAS-IIIA/CAS-III-D																			
151396->156834	+	WP_007220849.1	NZ_BAFH01000003.1	Candidatus Jettenia caeni	Bacteria	Planctomycetes	Planctomycetia	Candidatus Brocardiales	Candidatus Brocardiacea	Candidatus Jettenia	CRISPR-associated RAMP family protein	**Csm3gr7**	cls000253, cls000253	CAS-III-A, CAS-III-D					
157759->158964	+		NZ_BAFH01000003.1	Candidatus Jettenia caeni	Bacteria	Planctomycetes	Planctomycetia	Candidatus Brocardiales	Candidatus Brocardiacea	Candidatus Jettenia		CRISPR Array			16 (35-42)	17 (36)	M4/NA/F	CTTGAAGACTAAAGGAAGGAATTAATGTCACGGTAC	H
159307->159549	+	WP_007220851.1	NZ_BAFH01000003.1	Candidatus Jettenia caeni	Bacteria	Planctomycetes	Planctomycetia	Candidatus Brocardiales	Candidatus Brocardiacea	Candidatus Jettenia	H								
160165->162480	+	WP_007220853.1	NZ_BAFH01000003.1	Candidatus Jettenia caeni	Bacteria	Planctomycetes	Planctomycetia	Candidatus Brocardiales	Candidatus Brocardiacea	Candidatus Jettenia	CRISPR-associated endonuclease Cas1	**RT-cas1**	pfam00078, COG1518	CAS-III; CAS-I, CAS-II, CAS-III					
CAS-III-B																			
28466 ->29134	+	WP_005787766.1	NZ_JGDY01000030.1	Bacteroides fragilis S6L3	Bacteria	Bacteroidetes	Bacteroidia	Bacteroidales	Bacteroidaceae	Bacteroides	H	**Cas6**	cls000004	CAS-I, CAS-III					
29152 ->29778	+	WP_005787769.1	NZ_JGDY01000030.1	Bacteroides fragilis S6L3	Bacteria	Bacteroidetes	Bacteroidia	Bacteroidales	Bacteroidaceae	Bacteroides	NYN domain-containing protein								
29754 ->30533	+	WP_005787771.1	NZ_JGDY01000030.1	Bacteroides fragilis S6L3	Bacteria	Bacteroidetes	Bacteroidia	Bacteroidales	Bacteroidaceae	Bacteroides	H								
30530 ->32005	+	WP_005787774.1	NZ_JGDY01000030.1	Bacteroides fragilis S6L3	Bacteria	Bacteroidetes	Bacteroidia	Bacteroidales	Bacteroidaceae	Bacteroides	H								
32002 ->32481	+	WP_005815846.1	NZ_JGDY01000030.1	Bacteroides fragilis S6L3	Bacteria	Bacteroidetes	Bacteroidia	Bacteroidales	Bacteroidaceae	Bacteroides	H								
32495 ->33904	+	WP_005787778.1	NZ_JGDY01000030.1	Bacteroides fragilis S6L3	Bacteria	Bacteroidetes	Bacteroidia	Bacteroidales	Bacteroidaceae	Bacteroides	H	**Cmr1gr7**	mkCas0086	CAS-III-B					
33909 ->35711	+	WP_032529217.1	NZ_JGDY01000030.1	Bacteroides fragilis S6L3	Bacteria	Bacteroidetes	Bacteroidia	Bacteroidales	Bacteroidaceae	Bacteroides	type III-B CRISPR-associated protein Cas10/Cmr2	**Cas10**	pfam12469, cd09679	CAS-III-B; CAS-III					
35704 ->36843	+	WP_005787781.1	NZ_JGDY01000030.1	Bacteroides fragilis S6L3	Bacteria	Bacteroidetes	Bacteroidia	Bacteroidales	Bacteroidaceae	Bacteroides	CRISPR-associated protein Cmr3	**Cmr3gr5**	pfam09700	CAS-III-B					
36863 ->37702	+	WP_005787783.1	NZ_JGDY01000030.1	Bacteroides fragilis S6L3	Bacteria	Bacteroidetes	Bacteroidia	Bacteroidales	Bacteroidaceae	Bacteroides	type III-B CRISPR module RAMP protein Cmr4	**Cmr4gr7**	cd09682	CAS-III-B					
37715 ->38125	+	WP_005787785.1	NZ_JGDY01000030.1	Bacteroides fragilis S6L3	Bacteria	Bacteroidetes	Bacteroidia	Bacteroidales	Bacteroidaceae	Bacteroides	H	**Cmr5gr11**	mkCas0098	CAS-III-B					
38128 ->39072	+	WP_005787787.1	NZ_JGDY01000030.1	Bacteroides fragilis S6L3	Bacteria	Bacteroidetes	Bacteroidia	Bacteroidales	Bacteroidaceae	Bacteroides	type III-B CRISPR module RAMP protein Cmr6	**Cmr6gr7**	GOG1604	CAS-III-B					

39092 ->41362	+	WP_032556864.1	NZ_JGDY01000030.1	Bacteroides fragilis S6L3	Bacteria	Bacteroidetes	Bacteroidia	Bacteroidales	Bacteroidaceae	Bacteroides	CRISPR-associated endonuclease Cas1	RT-Cas1	pfam00078, cd09634	CAS-III; CAS-I, CAS-II, CAS-III, CAS-V					
41356 ->41646	+	WP_005787793.1	NZ_JGDY01000030.1	Bacteroides fragilis S6L3	Bacteria	Bacteroidetes	Bacteroidia	Bacteroidales	Bacteroidaceae	Bacteroides	CRISPR-associated endonuclease Cas2	Cas2	mkCas0081	CAS-I, CAS-II, CAS-III					
42093 ->42267	+		NZ_JGDY01000030.1	Bacteroides fragilis S6L3	Bacteria	Bacteroidetes	Bacteroidia	Bacteroidales	Bacteroidaceae	Bacteroides		CRISPR Array			2 (33,34)	3 (36)	NA/NA/E	TATGTAGATGTATTCCAGTATAATAAGGATTAAGAC	NA
CAS-III-D																			
990922->991590	+	WP_007481054.1	NZ_JH724308.1	Bacteroides salyersiae CL02T12C01	Bacteria	Bacteroidetes	Bacteroidia	Bacteroidales	Bacteroidaceae	Bacteroides	H	Cas6	cls000004	CAS-III					
991596->992213	+	WP_007481056.1	NZ_JH724308.1	Bacteroides salyersiae CL02T12C01	Bacteria	Bacteroidetes	Bacteroidia	Bacteroidales	Bacteroidaceae	Bacteroides	NYN domain-containing protein								
992217->993800	+	WP_007481058.1	NZ_JH724308.1	Bacteroides salyersiae CL02T12C01	Bacteria	Bacteroidetes	Bacteroidia	Bacteroidales	Bacteroidaceae	Bacteroides	H	Cas10	mkCas0133	CAS-III					
993817->994404	+	WP_007481060.1	NZ_JH724308.1	Bacteroides salyersiae CL02T12C01	Bacteria	Bacteroidetes	Bacteroidia	Bacteroidales	Bacteroidaceae	Bacteroides	H	Csm3gr7	mkCas0198	CAS-III-A, CAS-III-D					
994385->995863	+	WP_007481062.1	NZ_JH724308.1	Bacteroides salyersiae CL02T12C01	Bacteria	Bacteroidetes	Bacteroidia	Bacteroidales	Bacteroidaceae	Bacteroides	H	Csx10gr5	cls000748	CAS-III-D					
995860->997263	+	WP_007481064.1	NZ_JH724308.1	Bacteroides salyersiae CL02T12C01	Bacteria	Bacteroidetes	Bacteroidia	Bacteroidales	Bacteroidaceae	Bacteroides	H	Csm3gr7	pfam03787	CAS-III-A, CAS-III-D					
997266->997718	+	WP_007481065.1	NZ_JH724308.1	Bacteroides salyersiae CL02T12C01	Bacteria	Bacteroidetes	Bacteroidia	Bacteroidales	Bacteroidaceae	Bacteroides	type III CRISPR-associated protein	Csx19	mkCas0123	CAS-III-D					
997725->999779	+	WP_007481067.1	NZ_JH724308.1	Bacteroides salyersiae CL02T12C01	Bacteria	Bacteroidetes	Bacteroidia	Bacteroidales	Bacteroidaceae	Bacteroides	CRISPR-associated RAMP family protein	Csm3gr7	cls000253	CAS-III-A, CAS-III-D					
999779->1000147	+	WP_007481069.1	NZ_JH724308.1	Bacteroides salyersiae CL02T12C01	Bacteria	Bacteroidetes	Bacteroidia	Bacteroidales	Bacteroidaceae	Bacteroides	H	Csx10gr5	cls000118	CAS-I, CAS-III					
1000203->1001306	+	WP_044094007.1	NZ_JH724308.1	Bacteroides salyersiae CL02T12C01	Bacteria	Bacteroidetes	Bacteroidia	Bacteroidales	Bacteroidaceae	Bacteroides	H								
1001332->1003605	+	WP_007481073.1	NZ_JH724308.1	Bacteroides salyersiae CL02T12C01	Bacteria	Bacteroidetes	Bacteroidia	Bacteroidales	Bacteroidaceae	Bacteroides	CRISPR-associated endonuclease Cas1	RT-Cas1	pfam00078, pfam01867	CAS-III; CAS-I, CAS-II, CAS-III					
1004123 -> 1005619		Assembly gap	NZ_JH724308.1	Bacteroides salyersiae CL02T12C01	Bacteria	Bacteroidetes	Bacteroidia	Bacteroidales	Bacteroidaceae	Bacteroides	Assembly gap								
1005902->1006192	+	WP_007481075.1	NZ_JH724308.1	Bacteroides salyersiae CL02T12C01	Bacteria	Bacteroidetes	Bacteroidia	Bacteroidales	Bacteroidaceae	Bacteroides	CRISPR-associated endonuclease Cas2	Cas2	pfam09827	CAS-I, CAS-II, CAS-III					
CAS-III-D																			
8229 ->7228	-	WP_021087734.1	NZ_ANNG01000021.1	Campylobacter concisus UNSWCS	Bacteria	Proteobacteria	Epsiloproteobacteria	Campylobacterales	Campylobacteraceae	Campylobacter	WYL domain-containing protein	WYL	COG2378						
8612 ->8229	-	WP_035143591.1	NZ_ANNG01000021.1	Campylobacter concisus UNSWCS	Bacteria	Proteobacteria	Epsiloproteobacteria	Campylobacterales	Campylobacteraceae	Campylobacter	H	Csx20	xls002255	CAS-I, CAS-III					
9907 ->8609	-	WP_021087743.1	NZ_ANNG01000021.1	Campylobacter concisus UNSWCS	Bacteria	Proteobacteria	Epsiloproteobacteria	Campylobacterales	Campylobacteraceae	Campylobacter	H	Csx1	cd09732	CAS-III					
10705 ->9911	-	WP_021087723.1	NZ_ANNG01000021.1	Campylobacter concisus UNSWCS	Bacteria	Proteobacteria	Epsiloproteobacteria	Campylobacterales	Campylobacteraceae	Campylobacter	H								
12446 ->10920	-		NZ_ANNG01000021.1	Campylobacter concisus UNSWCS	Bacteria	Proteobacteria	Epsiloproteobacteria	Campylobacterales	Campylobacteraceae	Campylobacter	H	Csm3gr7	cls000253	CAS-III-A, CAS-III-D					
12865 ->12443	-	WP_021087727.1	NZ_ANNG01000021.1	Campylobacter concisus UNSWCS	Bacteria	Proteobacteria	Epsiloproteobacteria	Campylobacterales	Campylobacteraceae	Campylobacter	type III CRISPR-associated protein	Csx19	mkCas0121	CAS-III-D					
14156 ->12852	-	WP_021087722.1	NZ_ANNG01000021.1	Campylobacter concisus UNSWCS	Bacteria	Proteobacteria	Epsiloproteobacteria	Campylobacterales	Campylobacteraceae	Campylobacter	H	Csm3gr7	cd09726, cd09726	CAS-III-D					
15637 ->14153	-	WP_021087715.1	NZ_ANNG01000021.1	Campylobacter concisus UNSWCS	Bacteria	Proteobacteria	Epsiloproteobacteria	Campylobacterales	Campylobacteraceae	Campylobacter	H	Csx10gr5	cls000748	CAS-III-D					
16188 ->15637	-	WP_021087725.1	NZ_ANNG01000021.1	Campylobacter concisus UNSWCS	Bacteria	Proteobacteria		Campylobacterales	Campylobacteraceae	Campylobacter	H	Csm3gr7	mkCas0198	CAS-III-A, CAS-III-D					
17696 ->16185	-	WP_021087710.1	NZ_ANNG01000021.1	Campylobacter concisus UNSWCS	Bacteria	Proteobacteria	Epsiloproteobacteria	Campylobacterales	Campylobacteraceae	Campylobacter	H	Cas10	mkCas0133	CAS-III					
18509 ->17841	-	WP_021087712.1	NZ_ANNG01000021.1	Campylobacter concisus UNSWCS	Bacteria	Proteobacteria	Epsiloproteobacteria	Campylobacterales	Campylobacteraceae	Campylobacter	H								
19574->18689	-		NZ_ANNG01000021.1	Campylobacter concisus UNSWCS	Bacteria	Proteobacteria	Epsiloproteobacteria	Campylobacterales	Campylobacteraceae	Campylobacter		CRISPR Array			11 (40-49)	12 (33)	NA/NA/E	GATACAAATTTCCCCGATCTAGGGGATTGAAAC	H
20281 ->20009	-	WP_021087730.1	NZ_ANNG01000021.1	Campylobacter concisus UNSWCS	Bacteria	Proteobacteria	Epsiloproteobacteria	Campylobacterales	Campylobacteraceae	Campylobacter	CRISPR-associated endonuclease Cas2	Cas2	cd9725	CAS-I, CAS-II, CAS-III, CAS-V					
22473 ->20278	-	WP_021087740.1	NZ_ANNG01000021.1	Campylobacter concisus UNSWCS	Bacteria	Proteobacteria	Epsiloproteobacteria	Campylobacterales	Campylobacteraceae	Campylobacter	CRISPR-associated endonuclease Cas1	RT-Cas1	pfam00078, cd09634	CAS-III; CAS-I, CAS-II, CAS-III, CAS-V					
23344 ->22532	-	WP_021087741.1	NZ_ANNG01000021.1	Campylobacter concisus UNSWCS	Bacteria	Proteobacteria	Epsiloproteobacteria	Campylobacterales	Campylobacteraceae	Campylobacter	H	Cas6	mkCas0066	CAS-I, CAS-III					
Partial CAS-III																			
67,340 -> 68,152	+	WP_005873071.1	NZ_CP012196	Campylobacter gracilis ATCC 33236	Bacteria	Proteobacteria	Epsiloproteobacteria	Campylobacterales	Campylobacteraceae	Campylobacter	cytosolic protein	Cas6	mkCas0066	CAS-I, CAS-III					
68,285 -> 70,480	+	WP_005873073.1	NZ_CP012196	Campylobacter gracilis ATCC 33236	Bacteria	Proteobacteria	Epsiloproteobacteria	Campylobacterales	Campylobacteraceae	Campylobacter	CRISPR-associated endonuclease Cas1	RT-Cas1	pfam00078, COG1518	CAS-III; CAS-I, CAS-II, CAS-III					
70,477 -> 70,749	+	WP_005873074.1	NZ_CP012196	Campylobacter gracilis ATCC 33236	Bacteria	Proteobacteria	Epsiloproteobacteria	Campylobacterales	Campylobacteraceae	Campylobacter	CRISPR-associated endonuclease Cas2	Cas2	cd9725	CAS-I, CAS-II, CAS-III, CAS-V					
71182->74341	+		NZ_CP012196	Campylobacter gracilis ATCC 33236	Bacteria	Proteobacteria	Epsiloproteobacteria	Campylobacterales	Campylobacteraceae	Campylobacter		CRISPR Array			39 (35-45)	40 (36)	NA/NA/E	TTTGATTAGAATTTCCCCGATCTAGGGGATTGAAAC	H
74,564 -> 75,169	+	WP_005871601.1	NZ_CP012196	Campylobacter gracilis ATCC 33236	Bacteria	Proteobacteria	Epsiloproteobacteria	Campylobacterales	Campylobacteraceae	Campylobacter	H								
75,332 -> 75,985	+	WP_005871600.1	NZ_CP012196	Campylobacter gracilis ATCC 33236	Bacteria	Proteobacteria	Epsiloproteobacteria	Campylobacterales	Campylobacteraceae	Campylobacter	H								
76,710 -> 77,504	+	WP_005871598.1	NZ_CP012196	Campylobacter gracilis ATCC 33236	Bacteria	Proteobacteria	Epsiloproteobacteria	Campylobacterales	Campylobacteraceae	Campylobacter	H								
77,508 -> 78,755	+	WP_005871597.1	NZ_CP012196	Campylobacter gracilis ATCC 33236	Bacteria	Proteobacteria	Epsiloproteobacteria	Campylobacterales	Campylobacteraceae	Campylobacter	CRISPR-associated protein	Csx1	cd09732	CAS-III					
78,752 -> 79,132	+	WP_005871595.1	NZ_CP012196	Campylobacter gracilis ATCC 33236	Bacteria	Proteobacteria	Epsiloproteobacteria	Campylobacterales	Campylobacteraceae	Campylobacter	H	Csx20	xls002255	CAS-I, CAS-III					
79,132 -> 79,854	+	WP_005871594.1	NZ_CP012196	Campylobacter gracilis ATCC 33236	Bacteria	Proteobacteria	Epsiloproteobacteria	Campylobacterales	Campylobacteraceae	Campylobacter	H	Csx1	cd09747						
79,851 -> 80,132	+	WP_040304029.1	NZ_CP012196	Campylobacter gracilis ATCC 33236	Bacteria	Proteobacteria	Epsiloproteobacteria	Campylobacterales	Campylobacteraceae	Campylobacter	WYL domain-containing protein	WYL	COG2378						
CAS-III-D																			
332541 ->331723	-	WP_075539944.1	NZ_FIZP01000001.1	Campylobacter fetus subsp. fetus RC20	Bacteria	Proteobacteria	Epsiloproteobacteria	Campylobacterales	Campylobacteraceae	Campylobacter	H	Cas6	mkCas0066	CAS-I, CAS-III					
333686 ->332538	-	WP_075539945.1	NZ_FIZP01000001.1	Campylobacter fetus subsp. fetus RC20	Bacteria	Proteobacteria	Epsiloproteobacteria	Campylobacterales	Campylobacteraceae	Campylobacter	H	Csx1	cd09732	CAS-III					
334722 ->333769	-	WP_075539946.1	NZ_FIZP01000001.1	Campylobacter fetus subsp. fetus RC20	Bacteria	Proteobacteria	Epsiloproteobacteria	Campylobacterales	Campylobacteraceae	Campylobacter	H	WYL	pfam13280	CAS-I, CAS-III					
335450 ->334731	-	WP_075531261.1	NZ_FIZP01000001.1	Campylobacter fetus subsp. fetus RC20	Bacteria	Proteobacteria	Epsiloproteobacteria	Campylobacterales	Campylobacteraceae	Campylobacter	H								
336065 ->335460	-	WP_075539947.1	NZ_FIZP01000001.1	Campylobacter fetus subsp. fetus RC20	Bacteria	Proteobacteria	Epsiloproteobacteria	Campylobacterales	Campylobacteraceae	Campylobacter	H								
336596 ->336225	-	WP_075494296.1	NZ_FIZP01000001.1	Campylobacter fetus subsp. fetus RC20	Bacteria	Proteobacteria	Epsiloproteobacteria	Campylobacterales	Campylobacteraceae	Campylobacter	H	Csx23	mkCas0160	CAS-III-D					
338192->336694	-		NZ_FIZP01000001.1	Campylobacter fetus subsp. fetus RC20	Bacteria	Proteobacteria	Epsiloproteobacteria	Campylobacterales	Campylobacteraceae	Campylobacter		CRISPR Array			19 (34-45)	20 (37)	NA/NA/E	GTTTAGACACTTTCTCCGATTATTAGGGGATTGAAAC	H
339042 ->338770	-	WP_075539948.1	NZ_FIZP01000001.1	Campylobacter fetus subsp. fetus RC20	Bacteria	Proteobacteria	Epsiloproteobacteria	Campylobacterales	Campylobacteraceae	Campylobacter	H	Cas2	cd09725	CAS-I,CAS-II,CAS-III,CAS-V					
341243 ->339045	-	WP_075539949.1	NZ_FIZP01000001.1	Campylobacter fetus subsp. fetus RC20	Bacteria	Proteobacteria	Epsiloproteobacteria	Campylobacterales	Campylobacteraceae	Campylobacter	H	RT-Cas1	pfam00078, COG1518	CAS-III; CAS-I, CAS-II, CAS-III					
341612 ->341244	-	WP_075539950.1	NZ_FIZP01000001.1	Campylobacter fetus subsp. fetus RC20	Bacteria	Proteobacteria	Epsiloproteobacteria	Campylobacterales	Campylobacteraceae	Campylobacter	H	Csx20	xls002255	CAS-I, CAS-III					
343066 ->341609	-	WP_075539951.1	NZ_FIZP01000001.1	Campylobacter fetus subsp. fetus RC20	Bacteria	Proteobacteria	Epsiloproteobacteria	Campylobacterales	Campylobacteraceae	Campylobacter	H	Csx1	cd09732						
344861 ->343080	-	WP_075539952.1	NZ_FIZP01000001.1	Campylobacter fetus subsp. fetus RC20	Bacteria	Proteobacteria	Epsiloproteobacteria	Campylobacterales	Campylobacteraceae	Campylobacter	H	Csm3gr7	cls000253	CAS-III-A, CAS-III-D					
345369 ->344863	-	WP_075539953.1	NZ_FIZP01000001.1	Campylobacter fetus subsp. fetus RC20	Bacteria	Proteobacteria	Epsiloproteobacteria	Campylobacterales	Campylobacteraceae	Campylobacter	H	Csx19	cls001376	CAS-III-D					
346628 ->345366	-	WP_075539954.1	NZ_FIZP01000001.1	Campylobacter fetus subsp. fetus RC20	Bacteria	Proteobacteria	Epsiloproteobacteria	Campylobacterales	Campylobacteraceae	Campylobacter	H	Csm3gr7	cd09683, cd09683	CAS-III-A, CAS-III-D					
348667 ->346625	-	WP_075539955.1	NZ_FIZP01000001.1	Campylobacter fetus subsp. fetus RC20	Bacteria	Proteobacteria	Epsiloproteobacteria	Campylobacterales	Campylobacteraceae	Campylobacter	H	Csm3gr7	mkCas0198						
350160 ->348652	-	WP_075539956.1	NZ_FIZP01000001.1	Campylobacter fetus subsp. fetus RC20	Bacteria	Proteobacteria	Epsiloproteobacteria	Campylobacterales	Campylobacteraceae	Campylobacter	H	Cas10	mkCas0133	CAS-III					
CAS-III-B																			
1158607->1156993	-		NZ_JAFP01000001.1	Hippea sp. KM1	Bacteria	Proteobacteria	Deltaproteobacteria	Desulfurellales	Desulfurellaceae	Hippea		CRISPR Array			20 (38-46)	21 (37)	M4/NA/NA	GTCGACTGGGGAGTATGTTAAATGGTGATTTCGAGAC	H
1159286->1159014	-	WP_051453566.1	NZ_JAFP01000001.1	Hippea sp. KM1	Bacteria	Proteobacteria	Deltaproteobacteria	Desulfurellales	Desulfurellaceae	Hippea	CRISPR-associated endonuclease Cas2	Cas2	pfam09827	CAS-I, CAS-II, CAS-III					
1161400->1159286	-	WP_025270209.1	NZ_JAFP01000001.1	Hippea sp. KM1	Bacteria	Proteobacteria	Deltaproteobacteria	Desulfurellales	Desulfurellaceae	Hippea	CRISPR-associated endonuclease Cas1	RT-Cas1	pfam00078, COG1518	CAS-III; CAS-I, CAS-II, CAS-III					
1162493->1161315	-	WP_025270210.1	NZ_JAFP01000001.1	Hippea sp. KM1	Bacteria	Proteobacteria	Deltaproteobacteria	Desulfurellales	Desulfurellaceae	Hippea	H	Csx1	pfam09002						
1163598->1162504	-	WP_025270211.1	NZ_JAFP01000001.1	Hippea sp. KM1	Bacteria	Proteobacteria	Deltaproteobacteria	Desulfurellales	Desulfurellaceae	Hippea	type III-B CRISPR module RAMP protein Cmr6	Cmr6gr7	cd09661	CAS-III-B					
1163941->1163579	-	WP_025270212.1	NZ_JAFP01000001.1	Hippea sp. KM1	Bacteria	Proteobacteria	Deltaproteobacteria	Desulfurellales	Desulfurellaceae	Hippea	H	Cmr5gr7	mkCas0098	CAS-III-B					
1164774->1163938	-	WP_025270213.1	NZ_JAFP01000001.1	Hippea sp. KM1	Bacteria	Proteobacteria	Deltaproteobacteria	Desulfurellales	Desulfurellaceae	Hippea	type III-B CRISPR module RAMP protein Cmr4	Cmr4gr7	cd09682	CAS-III-B					
1165789->1164776	-	WP_025270214.1	NZ_JAFP01000001.1	Hippea sp. KM1	Bacteria	Proteobacteria	Deltaproteobacteria	Desulfurellales	Desulfurellaceae	Hippea	H	Cmr3gr5	pfam09700						
1167236->1165782	-	WP_025270215.1	NZ_JAFP01000001.1	Hippea sp. KM1	Bacteria	Proteobacteria	Deltaproteobacteria	Desulfurellales	Desulfurellaceae	Hippea	H	Cas10	pfam12469, cd09679	CAS-III-B; CAS-III					
1168465->1167239	-	WP_025270216.1	NZ_JAFP01000001.1	Hippea sp. KM1	Bacteria	Proteobacteria	Deltaproteobacteria	Desulfurellales	Desulfurellaceae	Hippea	H	Cmr1gr7	mkCas0086	CAS-III-B					
Partial CAS-III-B																			
29947->31080	+	WP_046996089.1	NZ_JAIQ01000034.1	Arcobacter butzleri L348 isolate CHRB125	Bacteria	Proteobacteria	Epsiloproteobacteria	Campylobacterales	Campylobacteraceae	Arcobacter	H	Cmr1gr7	mkCas0086	CAS-III-B					
31077->32438	+	WP_046996090.1	NZ_JAIQ01000034.1	Arcobacter butzleri L348 isolate CHRB125	Bacteria	Proteobacteria	Epsiloproteobacteria	Campylobacterales	Campylobacteraceae	Arcobacter	type III-B CRISPR-associated protein Cas10/Cmr2	Cas10	cd09679, cls000742	CAS-III; CAS-III					
32435->33430	+	WP_046996091.1	NZ_JAIQ01000034.1	Arcobacter butzleri L348 isolate CHRB125	Bacteria	Proteobacteria	Epsiloproteobacteria	Campylobacterales	Campylobacteraceae	Arcobacter	H								
33430->34239	+	WP_046996092.1	NZ_JAIQ01000034.1	Arcobacter butzleri L348 isolate CHRB125	Bacteria	Proteobacteria	Epsiloproteobacteria	Campylobacterales	Campylobacteraceae	Arcobacter	type III-B CRISPR module RAMP protein Cmr4	Cmr4gr7	cd09682	CAS-III-B					
34236->34595	+	WP_046996093.1	NZ_JAIQ01000034.1	Arcobacter butzleri L348 isolate CHRB125	Bacteria	Proteobacteria	Epsiloproteobacteria	Campylobacterales	Campylobacteraceae	Arcobacter	type III-B CRISPR module-associated protein Cmr5	Cmr5gr7	mkCas0098	CAS-III-B					
34597->35424	+	WP_052943009.1	NZ_JAIQ01000034.1	Arcobacter butzleri L348 isolate CHRB125	Bacteria	Proteobacteria	Epsiloproteobacteria	Campylobacterales	Campylobacteraceae	Arcobacter	type III-B CRISPR module RAMP protein Cmr6	Cmr6gr7	cd09661	CAS-III-B					
35428->37659	+	WP_046996094.1	NZ_JAIQ01000034.1	Arcobacter butzleri L348 isolate CHRB125	Bacteria	Proteobacteria	Epsiloproteobacteria	Campylobacterales	Campylobacteraceae	Arcobacter	CRISPR-associated endonuclease Cas1	RT-Cas1	pfam00078, pfam01867	CAS-III; CAS-I, CAS-II, CAS-III					
37659->37934	+	WP_046996095.1	NZ_JAIQ01000034.1	Arcobacter butzleri L348 isolate CHRB125	Bacteria	Proteobacteria	Epsiloproteobacteria	Campylobacterales	Campylobacteraceae	Arcobacter	CRISPR-associated endonuclease Cas2	Cas2	mkCas0081	CAS-I, CAS-II, CAS-III					
Unknown																			
3004->5310	+	KPA10619.1	JPDT01002544.1	Candidatus Magnetomorum sp. HK-1	Bacteria	Proteobacteria	Deltaproteobacteria	Desulfobacterales	Desulfobacteraceae	Candidatus Magnetomorum	cRISPR-associated endonuclease Cas1	RT-Cas1	pfam00078, cd09634	CAS-III; CAS-I, CAS-II, CAS-III, CAS-V					
5316->5597	+	KPA10618.1	JPDT01002544.1	Candidatus Magnetomorum sp. HK-1	Bacteria	Proteobacteria	Deltaproteobacteria	Desulfobacterales	Desulfobacteraceae	Candidatus Magnetomorum	CRISPR-associated protein Cas2	Cas2	pfam09827	CAS-I, CAS-II, CAS-III					
Unknown																			
2694->4343	+	ETR69258.1	ATBP01000692.1	Candidatus Magnetoglobus multicellularis str. Araruam	Bacteria	Proteobacteria	Deltaproteobacteria	Desulfobacterales	Desulfobacteraceae	Candidatus Magnetoglobus	RNA-directed DNA polymerase CDS	RT-Cas1	pfam00078, cd09634	CAS-III; CAS-I, CAS-II, CAS-III, CAS-V					

RT-CRISPR-3 (CAS-III-D, CAS-III-B/C)
Cyanobacteria (Nostocales, Oscillatoriales, Chroococcales, Synechococcales]

[a](Sub) Type/[b]Coordinates	Strand	Protein_id	Genome Partition ID	Strain	Domain	Phylum	Lineage (class)	Order	Family	Genus	[c]Cas gene (annotated)	[d]Cas gene/[e]Array (identified)	[d]Profiles	[d](Sub)Type-specificity	[e]Spacers (length)	[e]Repeats (length)	[f]Str.motif/Seq.family/Superclass	[f]Seq Repeats	[g]Orientation Confidence
CAS-III-D																			
458787->460808	+	KYC43938.1	ANNX02000012.1	Scytonema hofmannii PCC7110	Bacteria	Cyanobacteria		Nostocales	Scytonemataceae	Scytonema	CRISPR-associated protein Cas1	RT-Cas1	pfam00078, cd09721	CAS-III; CAS-I-C					
461358 -> 462329	+		ANNX02000012.1	Scytonema hofmannii PCC7110	Bacteria	Cyanobacteria		Nostocales	Scytonemataceae	Scytonema		CRISPR Array			13 (33-41)	14 (35)	M4/NA/E	GTTTTAATTCTTCTTCCCCTCACGGGGACGGAAAC	L
462501->462968	+	KYC44227.1	ANNX02000012.1	Scytonema hofmannii PCC7110	Bacteria	Cyanobacteria		Nostocales	Scytonemataceae	Scytonema	aldehyde-activating protein								
463258->464448	+	KYC43939.1	ANNX02000012.1	Scytonema hofmannii PCC7110	Bacteria	Cyanobacteria		Nostocales	Scytonemataceae	Scytonema	H								
464515->465093	+	KYC43940.1	ANNX02000012.1	Scytonema hofmannii PCC7110	Bacteria	Cyanobacteria		Nostocales	Scytonemataceae	Scytonema	H								
465708->465406	-	KYC43941.1	ANNX02000012.1	Scytonema hofmannii PCC7110	Bacteria	Cyanobacteria		Nostocales	Scytonemataceae	Scytonema	H								
466405->465827	-		ANNX02000012.1	Scytonema hofmannii PCC7110	Bacteria	Cyanobacteria		Nostocales	Scytonemataceae	Scytonema	H								
466669->467697	+	KYC43942.1	ANNX02000012.1	Scytonema hofmannii PCC7110	Bacteria	Cyanobacteria		Nostocales	Scytonemataceae	Scytonema	CRISPR-associated protein Cas1	Cas1	cd09634	CAS-I,CAS-II,CAS-III,CAS-V					
467700->467978	+	KYC43943.1	ANNX02000012.1	Scytonema hofmannii PCC7110	Bacteria	Cyanobacteria		Nostocales	Scytonemataceae	Scytonema	CRISPR-associated protein Cas2	Cas2	mkCas0081	CAS-I, CAS-II, CAS-III					
468201->468461	+	KYC43944.1	ANNX02000012.1	Scytonema hofmannii PCC7110	Bacteria	Cyanobacteria		Nostocales	Scytonemataceae	Scytonema	H								
468484->468777	+	KYC43945.1	ANNX02000012.1	Scytonema hofmannii PCC7110	Bacteria	Cyanobacteria		Nostocales	Scytonemataceae	Scytonema	H								
469298->468976	-		ANNX02000012.1	Scytonema hofmannii PCC7110	Bacteria	Cyanobacteria		Nostocales	Scytonemataceae	Scytonema	plasmid stabilization system								
469584->469291	-	KYC43946.1	ANNX02000012.1	Scytonema hofmannii PCC7110	Bacteria	Cyanobacteria		Nostocales	Scytonemataceae	Scytonema	prevent-host-death protein								
470025->471308	+	KYC43947.1	ANNX02000012.1	Scytonema hofmannii PCC7110	Bacteria	Cyanobacteria		Nostocales	Scytonemataceae	Scytonema	abortive infection protein								
471301->471909	+	KYC43948.1	ANNX02000012.1	Scytonema hofmannii PCC7110	Bacteria	Cyanobacteria		Nostocales	Scytonemataceae	Scytonema	abortive phage infection protein								
472499->471999	-	KYC43949.1	ANNX02000012.1	Scytonema hofmannii PCC7110	Bacteria	Cyanobacteria		Nostocales	Scytonemataceae	Scytonema	H	Csx1	pfam09670	CAS-III					
472799->472521	-	KYC43950.1	ANNX02000012.1	Scytonema hofmannii PCC7110	Bacteria	Cyanobacteria		Nostocales	Scytonemataceae	Scytonema	H	Csx1	pfam09670	CAS-III					
473432->472800	-	KYC43951.1	ANNX02000012.1	Scytonema hofmannii PCC7110	Bacteria	Cyanobacteria		Nostocales	Scytonemataceae	Scytonema	H	Csx21	mkCas0116	CAS-III-A, CAS-III-D					
474531->473464	-	KYC43952.1	ANNX02000012.1	Scytonema hofmannii PCC7110	Bacteria	Cyanobacteria		Nostocales	Scytonemataceae	Scytonema	CRISPR-associated protein	Csm3gr7	cls000253	CAS-III-A, CAS-III-D					
474979->474491	-	KYC43953.1	ANNX02000012.1	Scytonema hofmannii PCC7110	Bacteria	Cyanobacteria		Nostocales	Scytonemataceae	Scytonema	H	Csx19	cls000059	CAS-III-D					
475953->474976	-	KYC43954.1	ANNX02000012.1	Scytonema hofmannii PCC7110	Bacteria	Cyanobacteria		Nostocales	Scytonemataceae	Scytonema	CRISPR-associated protein	Csm3gr7	cd09683	CAS-III-A, CAS-III-D					
476607->475987	-	KYC43955.1	ANNX02000012.1	Scytonema hofmannii PCC7110	Bacteria	Cyanobacteria		Nostocales	Scytonemataceae	Scytonema	H	PD-DExK	mkCas0061	CAS-I, CAS-III					
477229->476618	-		ANNX02000012.1	Scytonema hofmannii PCC7110	Bacteria	Cyanobacteria		Nostocales	Scytonemataceae	Scytonema	CRISPR-associated protein Csm3	Csm3gr7	cd09683	CAS-III-A, CAS-III-D					
479217->477808	-	KYC43956.1	ANNX02000012.1	Scytonema hofmannii PCC7110	Bacteria	Cyanobacteria		Nostocales	Scytonemataceae	Scytonema	CRISPR-associated protein	Csx1	pfam09670	CAS-III					
479829->479254	-	KYC43957.1	ANNX02000012.1	Scytonema hofmannii PCC7110	Bacteria	Cyanobacteria		Nostocales	Scytonemataceae	Scytonema	H	Csm2gr11	mkCas0063	CAS-III-D					
481233->479833	-	KYC43958.1	ANNX02000012.1	Scytonema hofmannii PCC7110	Bacteria	Cyanobacteria		Nostocales	Scytonemataceae	Scytonema	CRISPR-associated protein Csx10	Csx10gr5	cls000715	CAS-III-D					
481943->481230	-	KYC43959.1	ANNX02000012.1	Scytonema hofmannii PCC7110	Bacteria	Cyanobacteria		Nostocales	Scytonemataceae	Scytonema	CRISPR-associated protein Csm3	Csm3gr7	cd09683	CAS-III-A, CAS-III-D					
484291->481940	-	KYC43960.1	ANNX02000012.1	Scytonema hofmannii PCC7110	Bacteria	Cyanobacteria		Nostocales	Scytonemataceae	Scytonema	type III-B CRISPR-associated protein Cas10/Cmr2	Cas10	cls000742	CAS-III					
485464->484499	-	KYC43961.1	ANNX02000012.1	Scytonema hofmannii PCC7110	Bacteria	Cyanobacteria		Nostocales	Scytonemataceae	Scytonema	CRISPR-associated protein Csx3	Csx3	mkCas0115	CAS-I, CAS-III					
485884->487341	+	KYC43962.1	ANNX02000012.1	Scytonema hofmannii PCC7110	Bacteria	Cyanobacteria		Nostocales	Scytonemataceae	Scytonema	CRISPR-associated protein	WYL	mkCas0110	CAS-I, CAS-III					
CAS-III-D																			
129955 -> 132345	+	WP_038295545.1	NZ_ALWD01000002	[Scytonema hofmanni] UTEX 2349	Bacteria	Cyanobacteria		Nostocales	Tolypothrichaceae	Tolypothrix	type III-B CRISPR-associated protein Cas10_Cmr2	Cas10	cls000742, cls000742	CAS-III; CAS-III					
132354 -> 133172	+	WP_029630482.1	NZ_ALWD01000002	[Scytonema hofmanni] UTEX 2349	Bacteria	Cyanobacteria		Nostocales	Tolypothrichaceae	Tolypothrix	CRISPR-associated protein Csm3	Csm3gr7	cd09683	CAS-III-A, CAS-III-D					
133169 -> 134515	+	WP_029630483.1	NZ_ALWD01000002	[Scytonema hofmanni] UTEX 2349	Bacteria	Cyanobacteria		Nostocales	Tolypothrichaceae	Tolypothrix	CRISPR-associated RAMP protein Csx10	Csx10gr5	cls000715	CAS-III-D					
134512 -> 134952	+	WP_038295547.1	NZ_ALWD01000002	[Scytonema hofmanni] UTEX 2349	Bacteria	Cyanobacteria		Nostocales	Tolypothrichaceae	Tolypothrix	H	Csm2gr11	mkCas0063	CAS-III-D					
134986 -> 135894	+	WP_038295549.1	NZ_ALWD01000002	[Scytonema hofmanni] UTEX 2349	Bacteria	Cyanobacteria		Nostocales	Tolypothrichaceae	Tolypothrix	CRISPR-associated RAMP protein	Csm3gr7	cd09683	CAS-III-A, CAS-III-D					
135904 -> 136914	+	WP_029630484.1	NZ_ALWD01000002	[Scytonema hofmanni] UTEX 2349	Bacteria	Cyanobacteria		Nostocales	Tolypothrichaceae	Tolypothrix	CRISPR-associated protein	Csm3gr7	cd09683	CAS-III-A, CAS-III-D					
136896 -> 137420	+	WP_029630485.1	NZ_ALWD01000002	[Scytonema hofmanni] UTEX 2349	Bacteria	Cyanobacteria		Nostocales	Tolypothrichaceae	Tolypothrix	H	Csx19	cls000059	CAS-III-D					
137413 -> 138456	+	WP_029630487.1	NZ_ALWD01000002	[Scytonema hofmanni] UTEX 2349	Bacteria	Cyanobacteria		Nostocales	Tolypothrichaceae	Tolypothrix	CRISPR-associated protein	Csm3gr7	cls000253	CAS-III-A, CAS-III-D					
138456 -> 139163	+	WP_029630489.1	NZ_ALWD01000002	[Scytonema hofmanni] UTEX 2349	Bacteria	Cyanobacteria		Nostocales	Tolypothrichaceae	Tolypothrix	H	Csx21	mkCas0076	CAS-III-A					
140044 -> 139190	-	WP_029630490.1	NZ_ALWD01000002	[Scytonema hofmanni] UTEX 2349	Bacteria	Cyanobacteria		Nostocales	Tolypothrichaceae	Tolypothrix	H								
140774->140092	-	WP_029630490.1	NZ_ALWD01000002	[Scytonema hofmanni] UTEX 2349	Bacteria	Cyanobacteria		Nostocales	Tolypothrichaceae	Tolypothrix		CRISPR Array			9 (35-43)	10 (35)	M4/NA/E	GTTTTCAACCAATTACCCCTCACGGGGATGGAAAC	L
142242 -> 141127	-	WP_029630492.1	NZ_ALWD01000002	[Scytonema hofmanni] UTEX 2349	Bacteria	Cyanobacteria		Nostocales	Tolypothrichaceae	Tolypothrix	CRISPR-associated endonuclease Cas6	Cas6	pfam10040	CAS-III, CAS-IV					
143311->142696	-	WP_029630490.1	NZ_ALWD01000002	[Scytonema hofmanni] UTEX 2349	Bacteria	Cyanobacteria		Nostocales	Tolypothrichaceae	Tolypothrix		CRISPR Array			8 (34-39)	9 (35)	NA/NA/C	GTTTTAATTCTTCTTCCCCTCACGGGGACGGAAAC	H
143630 -> 143370	-	WP_029630494.1	NZ_ALWD01000002	[Scytonema hofmanni] UTEX 2349	Bacteria	Cyanobacteria		Nostocales	Tolypothrichaceae	Tolypothrix	H								

144073 -> 143759	-	WP_029630496.1	NZ_ALWD01000002	[Scytonema hofmanni] UTEX 2349	Bacteria	Cyanobacteria		Nostocales	Tolypothrichaceae	Tolypothrix	H								
144522 -> 144253	-	WP_029630497.1	NZ_ALWD01000002	[Scytonema hofmanni] UTEX 2349	Bacteria	Cyanobacteria		Nostocales	Tolypothrichaceae	Tolypothrix	H								
144956 -> 144678	-	WP_038295551.1	NZ_ALWD01000002	[Scytonema hofmanni] UTEX 2349	Bacteria	Cyanobacteria		Nostocales	Tolypothrichaceae	Tolypothrix	CRISPR-associated endonuclease Cas2	**Cas2**	mkCas0081	CAS-I, CAS-II, CAS-III					
146002 -> 144956	-	WP_029630501.1	NZ_ALWD01000002	[Scytonema hofmanni] UTEX 2349	Bacteria	Cyanobacteria		Nostocales	Tolypothrichaceae	Tolypothrix	CRISPR-associated endonuclease Cas1	**Cas1**	COG1518	CAS-I, CAS-II, CAS-III					
147037 -> 146216	-	WP_029630502.1	NZ_ALWD01000002	[Scytonema hofmanni] UTEX 2349	Bacteria	Cyanobacteria		Nostocales	Tolypothrichaceae	Tolypothrix	H								
147116->147297	+	WP_029630490.1	NZ_ALWD01000002	[Scytonema hofmanni] UTEX 2349	Bacteria	Cyanobacteria		Nostocales	Tolypothrichaceae	Tolypothrix		CRISPR Array			2 (38,39)	3(35)	NA/NA/NA	GTTTCCATCCCCGTGAGGGGAAGAGGAATTAAAAC	M
150073 -> 147389	-	WP_051502720.1	NZ_ALWD01000002	[Scytonema hofmanni] UTEX 2349	Bacteria	Cyanobacteria		Nostocales	Tolypothrichaceae	Tolypothrix	Transposase								
150073 -> 150495	+	WP_029630504.1	NZ_ALWD01000002	[Scytonema hofmanni] UTEX 2349	Bacteria	Cyanobacteria		Nostocales	Tolypothrichaceae	Tolypothrix	H								
150598->150926	+	WP_029630490.1	NZ_ALWD01000002	[Scytonema hofmanni] UTEX 2349	Bacteria	Cyanobacteria		Nostocales	Tolypothrichaceae	Tolypothrix		CRISPR Array			4(36,44)	5(35)	NA/NA/NA	GTTTCCATCCCCGTGAGGGGAAGAGGAATTAAAAC	M
153377 -> 151368	-	WP_029630506.1	NZ_ALWD01000002	[Scytonema hofmanni] UTEX 2349	Bacteria	Cyanobacteria		Nostocales	Tolypothrichaceae	Tolypothrix	CRISPR-associated endonuclease Cas1	**RT-Cas1**	pfam00078, COG1518	CAS-III; CAS-I, CAS-II, CAS-III					
154104 -> 154856	+	WP_029630507.1	NZ_ALWD01000002	[Scytonema hofmanni] UTEX 2349	Bacteria	Cyanobacteria		Nostocales	Tolypothrichaceae	Tolypothrix	H								
156500->155390	-	WP_029630490.1	NZ_ALWD01000002	[Scytonema hofmanni] UTEX 2349	Bacteria	Cyanobacteria		Nostocales	Tolypothrichaceae	Tolypothrix		CRISPR Array			15(33,40)	16(35)	NA/NA/NA	GTTTCCATCCCCGTGAGGGGAAGAGAAATTGAAAC	H
**Unknown**																			
10446 -> 12452	+	WP_041039832.1	NZ_JXCB01000011	Tolypothrix campylonemoides VB511288	Bacteria	Cyanobacteria		Nostocales	Tolypothrichaceae	Tolypothrix	CRISPR-associated endonuclease Cas1	**RT-Cas1**	pfam00078, COG1518	CAS-III; CAS-I, CAS-II, CAS-III					
12656-13688	+		NZ_JXCB01000011	Tolypothrix campylonemoides VB511288	Bacteria	Cyanobacteria		Nostocales	Tolypothrichaceae	Tolypothrix		CRISPR Array			14(34,39)	15(35)	NA/NA/C	CTTTAAACTTCTTCGGAAGTTGAATTAATGGAAAC	M
**CAS-III-D**																			
1727817 ->1728071	+		NC_003272	Noctoc sp. PCC7120	Bacteria	Cyanobacteria		Nostocales	Nostocaceae	Nostoc		CRISPR Array			3 (37-40)	4 (35)	NA/NA/C	GTTTTCATCCCCGTGAGGGGTAAAGGAATTAAAAC	H
1729151 -> 1728408	-	WP_010995637.1	NC_003272	Noctoc sp. PCC7120	Bacteria	Cyanobacteria		Nostocales	Nostocaceae	Nostoc	H								
1729649 -> 1731655	+	WP_010995638.1	NC_003272	Noctoc sp. PCC7120	Bacteria	Cyanobacteria		Nostocales	Nostocaceae	Nostoc	CRISPR-associated endonuclease Cas1	**RT-Cas1**	pfam00078, COG1518	CAS-III; CAS-I, CAS-II, CAS-III					
1733321 -> 1732269	-		NC_003272	Noctoc sp. PCC7120	Bacteria	Cyanobacteria		Nostocales	Nostocaceae	Nostoc		CRISPR Array			14 (34-40)	15 (35)	NA/NA/C	GTTTCCATCCCCGTGAGGGGTAAAGGAATTAAAAC	H
1733488 -> 1734309	+	WP_010995640.1	NC_003272	Noctoc sp. PCC7120	Bacteria	Cyanobacteria		Nostocales	Nostocaceae	Nostoc	H								
1735078 -> 1734362	-	WP_010995641.1	NC_003272	Noctoc sp. PCC7120	Bacteria	Cyanobacteria		Nostocales	Nostocaceae	Nostoc	H	**Csx21**	mkCas0076	CAS-III-A					
1736143 -> 1735088	-	WP_010995642.1	NC_003272	Noctoc sp. PCC7120	Bacteria	Cyanobacteria		Nostocales	Nostocaceae	Nostoc	CRISPR-associated protein	**Csm3gr7**	cls000253, cls000253	CAS-III-A, CAS-III-D; CAS-III-A, CAS-III-D					
1736672 -> 1736145	-	WP_010995643.1	NC_003272	Noctoc sp. PCC7120	Bacteria	Cyanobacteria		Nostocales	Nostocaceae	Nostoc	H	**Csx19**	cls000059	CAS-III-D					
1736672 -> 1736145	-	WP_010995644.1	NC_003272	Noctoc sp. PCC7120	Bacteria	Cyanobacteria		Nostocales	Nostocaceae	Nostoc	CRISPR-associated protein	**Csm3gr7**	cd09683	CAS-III-A, CAS-III-D					
1738604 -> 1737678	-	WP_010995645.1	NC_003272	Noctoc sp. PCC7120	Bacteria	Cyanobacteria		Nostocales	Nostocaceae	Nostoc	CRISPR-associated RAMP protein	**Csm3gr7**	cd09683	CAS-III-A, CAS-III-D					
1739075 -> 1738620	-	WP_010995646.1	NC_003272	Noctoc sp. PCC7120	Bacteria	Cyanobacteria		Nostocales	Nostocaceae	Nostoc	H	**Csm2gr11**	mkCas0063	CAS-III-D					
1740376 -> 1739072	-	WP_010995647.1	NC_003272	Noctoc sp. PCC7120	Bacteria	Cyanobacteria		Nostocales	Nostocaceae	Nostoc	CRISPR-associated RAMP protein Csx10	**Csx10gr5**	cls000715	CAS-III-D					
1741131 -> 1740373	-	WP_010995648.1	NC_003272	Noctoc sp. PCC7120	Bacteria	Cyanobacteria		Nostocales	Nostocaceae	Nostoc	CRISPR-associated protein Csm3	**Csm3gr7**	COG1337	CAS-III-D					
1743494 -> 1741128	-	WP_010995649.1	NC_003272	Noctoc sp. PCC7120	Bacteria	Cyanobacteria		Nostocales	Nostocaceae	Nostoc	type III-B CRISPR-associated protein Cas10/Cmr2	**Cas10**	cls000742	CAS-III					
1744053 -> 1743700	-	WP_044520963.1	NC_003272	Noctoc sp. PCC7120	Bacteria	Cyanobacteria		Nostocales	Nostocaceae	Nostoc	H								
1744833 -> 1745966	+	WP_010995652.1	NC_003272	Noctoc sp. PCC7120	Bacteria	Cyanobacteria		Nostocales	Nostocaceae	Nostoc	CRISPR-associated endoribonuclease Cas6	**Cas6**	COG5551	CAS-III					
**Partial CAS-III-B/CAS-III-C**																			
314350 -> 316356	+	AFY99362.1	NC_019751	Calothrix PCC6303	Bacteria	Cyanobacteria		Nostocales	Nostocaceae	Calothrix	RNA-directed DNA polymerase	**RT-Cas1**	pfam00078, COG1518	CAS-III; CAS-I, CAS-II, CAS-III					
316560 -> 317384	+		NC_019751	Calothrix PCC6303	Bacteria	Cyanobacteria		Nostocales	Nostocaceae	Calothrix		CRISPR Array			11 (35-40)	12 (35)	NA/NA/C	CTTTAAACTTCTTCGGAAGTTGAATTAATGGAAAC	L
317686 -> 318726	+	AFY99363.1	NC_019751	Calothrix PCC6303	Bacteria	Cyanobacteria		Nostocales	Nostocaceae	Calothrix	CRISPR-associated protein Cas1	**Cas1**	COG1518	CAS-I, CAS-II, CAS-III					
318738 -> 319016	+	AFY99364.1	NC_019751	Calothrix PCC6303	Bacteria	Cyanobacteria		Nostocales	Nostocaceae	Calothrix	CRISPR-associated protein Cas2	**Cas2**	mkCas0081	CAS-I, CAS-II, CAS-III					
319268 -> 319537	+	AFY99365.1	NC_019751	Calothrix PCC6303	Bacteria	Cyanobacteria		Nostocales	Nostocaceae	Calothrix	H								
319634 -> 319933	+	AFY99366.1	NC_019751	Calothrix PCC6303	Bacteria	Cyanobacteria		Nostocales	Nostocaceae	Calothrix	H								
320157 -> 320330	+	AFY99367.1	NC_019751	Calothrix PCC6303	Bacteria	Cyanobacteria		Nostocales	Nostocaceae	Calothrix	H								
320451 -> 320843	+	AFY99368.1	NC_019751	Calothrix PCC6303	Bacteria	Cyanobacteria		Nostocales	Nostocaceae	Calothrix	H								
321420 -> 321713	+	AFY99369.1	NC_019751	Calothrix PCC6303	Bacteria	Cyanobacteria		Nostocales	Nostocaceae	Calothrix	H								
321911 -> 322243	+	AFY99370.1	NC_019751	Calothrix PCC6303	Bacteria	Cyanobacteria		Nostocales	Nostocaceae	Calothrix	H								
324319 -> 325428	+		NC_019751	Calothrix PCC6303	Bacteria	Cyanobacteria		Nostocales	Nostocaceae	Calothrix		CRISPR Array			15 (35-44)	16 (35)	NA/NA/C	CTTTAGACTTCTTCGGAAGTTGAATTAATGGAAAC	M
327913 -> 325820	-	AFY99371.1	NC_019751	Calothrix PCC6303	Bacteria	Cyanobacteria		Nostocales	Nostocaceae	Calothrix	H	WYL	COG2378						
328873-> 328043	-		NC_019751	Calothrix PCC6303	Bacteria	Cyanobacteria		Nostocales	Nostocaceae	Calothrix		CRISPR Array			11 (33-37)	12 (37)	NA/NA/C	GTTTCAAAGCTTCTTTTGAAGCTGAATTAATGGAAAC	M
329578 -> 331143	+	AFY99372.1	NC_019751	Calothrix PCC6303	Bacteria	Cyanobacteria		Nostocales	Nostocaceae	Calothrix	H	**Cas10**	cd09679	CAS-III					
332142 -> 332951	+	AFY99373.1	NC_019751	Calothrix PCC6303	Bacteria	Cyanobacteria		Nostocales	Nostocaceae	Calothrix	CRISPR-associated protein, Cmr4 family	**Cmr4gr7**	COG1336	CAS-III-C					
333003 -> 333431	+	AFY99374.1	NC_019751	Calothrix PCC6303	Bacteria	Cyanobacteria		Nostocales	Nostocaceae	Calothrix	H	**Cmr5gr11**	mkCas0065	CAS-III-B					
333428 -> 335047	+	AFY99375.1	NC_019751	Calothrix PCC6303	Bacteria	Cyanobacteria		Nostocales	Nostocaceae	Calothrix	H								
335225 -> 336832	+	AFY99376.1	NC_019751	Calothrix PCC6303	Bacteria	Cyanobacteria		Nostocales	Nostocaceae	Calothrix	H	**Csx1**	mkCas0108	CAS-III					
**CAS-III-D**																			
3036751 -> 3039057	+	AFY33108.1	NC_019682	Calothrix PCC7507	Bacteria	Cyanobacteria		Nostocales	Nostocaceae	Calothrix	CRISPR-associated protein, Crm2 family	**Cas10**	cls000742	CAS-III					
3039054 -> 3039767	+	AFY33109.1	NC_019682	Calothrix PCC7507	Bacteria	Cyanobacteria		Nostocales	Nostocaceae	Calothrix	protein of unknown function DUF324	**Csm3gr7**	cd09683	CAS-III-A, CAS-III-D					
3039764 -> 3041038	+	AFY33110.1	NC_019682	Calothrix PCC7507	Bacteria	Cyanobacteria		Nostocales	Nostocaceae	Calothrix	CRISPR-associated RAMP protein, Csx10 family	**Csx10gr5**	cls000715	CAS-III-D					
3041035 -> 3041517	+	AFY33111.1	NC_019682	Calothrix PCC7507	Bacteria	Cyanobacteria		Nostocales	Nostocaceae	Calothrix	H	**Csm2gr11**	mkCas0063	CAS-III-D					
3041523 -> 3042383	+	AFY33112.1	NC_019682	Calothrix PCC7507	Bacteria	Cyanobacteria		Nostocales	Nostocaceae	Calothrix	CRISPR-associated protein, Csx7 family	**Csm3gr7**	cd09683	CAS-III-A, CAS-III-D					
3042392 -> 3043369	+	AFY33113.1	NC_019682	Calothrix PCC7507	Bacteria	Cyanobacteria		Nostocales	Nostocaceae	Calothrix	protein of unknown function DUF324	**Csm3gr7**	cd09683	CAS-III-A, CAS-III-D					
3043366 -> 3043803	+	AFY33114.1	NC_019682	Calothrix PCC7507	Bacteria	Cyanobacteria		Nostocales	Nostocaceae	Calothrix	H	**Csx19**	cls000059	CAS-III-D					
3043826 -> 3044902	+	AFY33115.1	NC_019682	Calothrix PCC7507	Bacteria	Cyanobacteria		Nostocales	Nostocaceae	Calothrix	H	**Csm3gr7**	cls000253,cls000253	CAS-III-A, CAS-III-D					
3044970 -> 3045614	+	AFY33116.1	NC_019682	Calothrix PCC7507	Bacteria	Cyanobacteria		Nostocales	Nostocaceae	Calothrix	H	**Csx21**	mkCas0076	CAS-III-A					
3046444 -> 3045626	-	AFY33117.1	NC_019682	Calothrix PCC7507	Bacteria	Cyanobacteria		Nostocales	Nostocaceae	Calothrix	H								
3047665 -> 3046553	-	AFY33118.1	NC_019682	Calothrix PCC7507	Bacteria	Cyanobacteria		Nostocales	Nostocaceae	Calothrix	CRISPR-associated protein, Cas6 family	**Cas6**	pfam10040	CAS-III, CAS-IV					
3048107 -> 3047871	-	AFY33119.1	NC_019682	Calothrix PCC7507	Bacteria	Cyanobacteria		Nostocales	Nostocaceae	Calothrix	H								
3048534 -> 3048232	-	AFY33120.1	NC_019682	Calothrix PCC7507	Bacteria	Cyanobacteria		Nostocales	Nostocaceae	Calothrix	H								
3048886 -> 3048617	-	AFY33121.1	NC_019682	Calothrix PCC7507	Bacteria	Cyanobacteria		Nostocales	Nostocaceae	Calothrix	H								
3049298 -> 3049017	-	AFY33122.1	NC_019682	Calothrix PCC7507	Bacteria	Cyanobacteria		Nostocales	Nostocaceae	Calothrix	CRISPR-associated protein Cas2	**Cas2**	mkCas0081	CAS-I, CAS-II, CAS-III					
3050335 -> 3049295	-	AFY33123.1	NC_019682	Calothrix PCC7507	Bacteria	Cyanobacteria		Nostocales	Nostocaceae	Calothrix	CRISPR-associated protein Cas1	**Cas1**	COG1518	CAS-I, CAS-II, CAS-III					
3050924 -> 3050499	-	AFY33124.1	NC_019682	Calothrix PCC7507	Bacteria	Cyanobacteria		Nostocales	Nostocaceae	Calothrix	HNH endonuclease								
3051325 -> 3050924	-	AFY33125.1	NC_019682	Calothrix PCC7507	Bacteria	Cyanobacteria		Nostocales	Nostocaceae	Calothrix	H								
3053058-> 3051419	-		NC_019682	Calothrix PCC7507	Bacteria	Cyanobacteria		Nostocales	Nostocaceae	Calothrix		CRISPR Array			22 (36-43)	23 (35)	M4/F9/E	GTTTTAATTCCTTTACCCCTCACGGGGATGGAAAC	L
3055466 -> 3053445	-	AFY33126.1	NC_019682	Calothrix PCC7507	Bacteria	Cyanobacteria		Nostocales	Nostocaceae	Calothrix	RNA-directed DNA polymerase	**RT-Cas1**	pfam00078, cd09634	CAS-III; CAS-I, CAS-II, CAS-III, CAS-V					
**Partial CAS-III-B/CAS-III-C**																			
115492 -> 115214	-	WP_044448075.1	NZ_JXIJ01000035	Mastigocladus laminosus UU774	Bacteria	Cyanobacteria		Nostocales	Hapalosiphonaceae	Mastigocladus	CRISPR-associated endonuclease Cas2	**Cas2**	mkCas0081	CAS-I, CAS-II, CAS-III					
116532 -> 115492	-	WP_044448017.1	NZ_JXIJ01000035	Mastigocladus laminosus UU774	Bacteria	Cyanobacteria		Nostocales	Hapalosiphonaceae	Mastigocladus	CRISPR-associated endonuclease Cas1	**Cas1**	COG1518	CAS-I, CAS-II, CAS-III					
119991 -> 117985	-	WP_044448019.1	NZ_JXIJ01000035	Mastigocladus laminosus UU774	Bacteria	Cyanobacteria		Nostocales	Hapalosiphonaceae	Mastigocladus	CRISPR-associated endonuclease Cas1	**RT-Cas1**	pfam00078, COG1518	CAS-III; CAS-I, CAS-II, CAS-III					
120070 -> 120843	+	WP_044448020.1	NZ_JXIJ01000035	Mastigocladus laminosus UU774	Bacteria	Cyanobacteria		Nostocales	Hapalosiphonaceae	Mastigocladus	H								
120886 -> 122489...	+		NZ_JXIJ01000035	Mastigocladus laminosus UU774	Bacteria	Cyanobacteria		Nostocales	Hapalosiphonaceae	Mastigocladus	H								
122669 -> 123788	+		NZ_JXIJ01000035	Mastigocladus laminosus UU774	Bacteria	Cyanobacteria		Nostocales	Hapalosiphonaceae	Mastigocladus	H								
128110 -> 126728	-	WP_044448024.1	NZ_JXIJ01000035	Mastigocladus laminosus UU774	Bacteria	Cyanobacteria		Nostocales	Hapalosiphonaceae	Mastigocladus	H	**Csx1**	mkCas0108	CAS-III					
129849 -> 128107	-	WP_044448026.1	NZ_JXIJ01000035	Mastigocladus laminosus UU774	Bacteria	Cyanobacteria		Nostocales	Hapalosiphonaceae	Mastigocladus	H	Cmr6gr7	COG1604						
130255 -> 129842	-	WP_044448028.1	NZ_JXIJ01000035	Mastigocladus laminosus UU774	Bacteria	Cyanobacteria		Nostocales	Hapalosiphonaceae	Mastigocladus	H	**Cmr5gr11**	mkCas0065	CAS-III-B					
131125 -> 130268	-	WP_044448030.1	NZ_JXIJ01000035	Mastigocladus laminosus UU774	Bacteria	Cyanobacteria		Nostocales	Hapalosiphonaceae	Mastigocladus	CRISPR-associated protein, Cmr4	**Cmr4gr7**	COG1336	CAS-III-C					
132177 -> 131125	-	WP_044448032.1	NZ_JXIJ01000035	Mastigocladus laminosus UU774	Bacteria	Cyanobacteria		Nostocales	Hapalosiphonaceae	Mastigocladus	CRISPR-associated protein, Cmr3								
133742 -> 132162	-	WP_026087287.1	NZ_JXIJ01000035	Mastigocladus laminosus UU774	Bacteria	Cyanobacteria		Nostocales	Hapalosiphonaceae	Mastigocladus	H	**Cas10**	cls000742, cls000742	CAS-III; CAS-III					
**CAS-III-B**																			
149604 ->151610	+	AFZ61061.1	CP003660	Anabaena cylindrica PCC7122	Bacteria	Cyanobacteria		Nostocales	Nostocaceae	Anabaena	RNA-directed DNA polymerase	**RT-Cas1**	pfam00078, COG1518	CAS-III; CAS-I, CAS-II, CAS-III					
151821 ->154033	+		CP003660	Anabaena cylindrica PCC7122	Bacteria	Cyanobacteria		Nostocales	Nostocaceae	Anabaena		CRISPR Array			30 (35-43)	31 (35)	NA/NA/C	CTTTTAACTTCTTAGAAAGTTGAACTAATGGAAAC	NA
154306 ->155358	+	AFZ61062.1	CP003660	Anabaena cylindrica PCC7122	Bacteria	Cyanobacteria		Nostocales	Nostocaceae	Anabaena	CRISPR-associated protein Cas1	**Cas1**	COG1518	CAS-I, CAS-II, CAS-III					
155932 ->155585	-	AFZ61063.1	CP003660	Anabaena cylindrica PCC7122	Bacteria	Cyanobacteria		Nostocales	Nostocaceae	Anabaena	CHP02436-containing protein								
156119 ->156397	+	AFZ61064.1	CP003660	Anabaena cylindrica PCC7122	Bacteria	Cyanobacteria		Nostocales	Nostocaceae	Anabaena	CRISPR-associated protein Cas2	**Cas2**	mkCas0081	CAS-I, CAS-II, CAS-III					
156580 ->156840	+	AFZ61065.1	CP003660	Anabaena cylindrica PCC7122	Bacteria	Cyanobacteria		Nostocales	Nostocaceae	Anabaena	H								
157182 ->157478	+	AFZ61066.1	CP003660	Anabaena cylindrica PCC7122	Bacteria	Cyanobacteria		Nostocales	Nostocaceae	Anabaena	H								
157618 ->157791	+	AFZ61067.1	CP003660	Anabaena cylindrica PCC7122	Bacteria	Cyanobacteria		Nostocales	Nostocaceae	Anabaena	H	Csn2	cls000024						
158033 ->158887	+	AFZ61068.1	CP003660	Anabaena cylindrica PCC7122	Bacteria	Cyanobacteria		Nostocales	Nostocaceae	Anabaena	H								
158960 ->162091	+	AFZ61069.1	CP003660	Anabaena cylindrica PCC7122	Bacteria	Cyanobacteria		Nostocales	Nostocaceae	Anabaena	CRISPR-associated protein, Crm2 family	**Cas10**	cd09679, cd9679	CAS-III					
162095 ->163306	+	AFZ61070.1	CP003660	Anabaena cylindrica PCC7122	Bacteria	Cyanobacteria		Nostocales	Nostocaceae	Anabaena	CRISPR-associated protein, Cmr3	**Cmr3gr5**	cd09748	CAS-III-B					
163299 ->164198	+	AFZ61071.1	CP003660	Anabaena cylindrica PCC7122	Bacteria	Cyanobacteria		Nostocales	Nostocaceae	Anabaena	CRISPR-associated protein, Cmr4 family	**Cmr4gr7**	COG1336	CAS-III-C					
164201 ->164662	+	AFZ61072.1	CP003660	Anabaena cylindrica PCC7122	Bacteria	Cyanobacteria		Nostocales	Nostocaceae	Anabaena	H	**Cmr5gr11**	mkCas0205	CAS-III-B					
164697 ->166421	+	AFZ61073.1	CP003660	Anabaena cylindrica PCC7122	Bacteria	Cyanobacteria		Nostocales	Nostocaceae	Anabaena	H	**Cmr6gr7**	COG1604	CAS-III-B					
**CAS-III-B**																			
835033->832946	-	ADN17986.1	CP_002199	Cyanothece PCC7822	Bacteria	Cyanobacteria	Oscillatoriophycideae	Oscillatoriales	Cyanothecaceae	Cyanothece	protein of unknown function DUF324	**Cmr6gr7**	COG1604,COG1367	CAS-III-B					
835515->835036	-	ADN17987.1	CP_002199	Cyanothece PCC7822	Bacteria	Cyanobacteria	Oscillatoriophycideae	Oscillatoriales	Cyanothecaceae	Cyanothece	H	**Cmr5gr11**	cls000022	CAS-III-B					
836543->835515	-	ADN17988.1	CP_002199	Cyanothece PCC7822	Bacteria	Cyanobacteria	Oscillatoriophycideae	Oscillatoriales	Cyanothecaceae	Cyanothece	CRISPR-associated RAMP protein, Cmr4 family	**Cmr4gr7**	COG1336	CAS-III-C					
837718->836555	-	ADN17989.1	CP_002199	Cyanothece PCC7822	Bacteria	Cyanobacteria	Oscillatoriophycideae	Oscillatoriales	Cyanothecaceae	Cyanothece	CRISPR-associated protein, Crm3	**Cmr3gr5**	cd09748	CAS-III-B					
840611->837711	-	ADN17990.1	CP_002199	Cyanothece PCC7822	Bacteria	Cyanobacteria	Oscillatoriophycideae	Oscillatoriales	Cyanothecaceae	Cyanothece	CRISPR-associated protein, Crm2 family	**Cas10**	cls000742	CAS-III					
841965->841042	-		CP_002199	Cyanothece PCC7822	Bacteria	Cyanobacteria	Oscillatoriophycideae	Oscillatoriales	Cyanothecaceae	Cyanothece		CRISPR Array			12 (35-42)	13 (36)	M15/NA/F	CTTTTCCTCTTAGGAGGAAGCTAATAGAATGGAAAC	L
842242->842775	+	ADN17991.1	CP_002199	Cyanothece PCC7822	Bacteria	Cyanobacteria	Oscillatoriophycideae	Oscillatoriales	Cyanothecaceae	Cyanothece	H								
843786->843250	-	ADN17993.1	CP_002199	Cyanothece PCC7822	Bacteria	Cyanobacteria	Oscillatoriophycideae	Oscillatoriales	Cyanothecaceae	Cyanothece	H	Cas8b5	mkCas0131						
844194->843886	-	ADN17994.1	CP_002199	Cyanothece PCC7822	Bacteria	Cyanobacteria	Oscillatoriophycideae	Oscillatoriales	Cyanothecaceae	Cyanothece	H								
844512->844222	-	ADN17995.1	CP_002199	Cyanothece PCC7822	Bacteria	Cyanobacteria	Oscillatoriophycideae	Oscillatoriales	Cyanothecaceae	Cyanothece	CRISPR-associated protein Cas2	**Cas2**	mkCas0081	CAS-I, CAS-II, CAS-III					
846553->844538	-	ADN17996.1	CP_002199	Cyanothece PCC7822	Bacteria	Cyanobacteria	Oscillatoriophycideae	Oscillatoriales	Cyanothecaceae	Cyanothece	CRISPR-associated protein Cas1	**RT-Cas1**	pfam00078, COG1518	CAS-III; CAS-I, CAS-II, CAS-III					
849039->846786	-		CP_002199	Cyanothece PCC7822	Bacteria	Cyanobacteria	Oscillatoriophycideae	Oscillatoriales	Cyanothecaceae	Cyanothece		CRISPR Array			31 (34-41)	32 (35)	M15/NA/F	CTTTTCTCTTAGGAGAAAACTAACAGAATGGAAAC	NA
**CAS-III-D**																			
8048 -> 8232	+		CP_003633	Microcoleus sp. PCC7113	Bacteria	Cyanobacteria	Oscillatoriophycideae	Oscillatoriales	Microcoleaceae	Microcoleus		CRISPR Array			2 (38,39)	3 (36)	NA/NA/C	GTTTCCGTCCCCGTGAAGGGGAAGTGAATTGAAAC	H
10793-> 8790	-	AFZ22158.1	CP_003633	Microcoleus sp. PCC7113	Bacteria	Cyanobacteria	Oscillatoriophycideae	Oscillatoriales	Microcoleaceae	Microcoleus	CRISPR-associated endonuclease Cas1	**RT-Cas1**	pfam00078, COG1518	CAS-III; CAS-I, CAS-II, CAS-III					
11789-> 11240	-		CP_003633	Microcoleus sp. PCC7113	Bacteria	Cyanobacteria	Oscillatoriophycideae	Oscillatoriales	Microcoleaceae	Microcoleus		CRISPR Array			7 (34-44)	8 (35)	NA/F18/C	GTTTCCGTCCCCGTAAAGGGGAAGAGATTTGAAAC	M
11988-> 12989	+	AFZ22159.1	CP_003633	Microcoleus sp. PCC7113	Bacteria	Cyanobacteria	Oscillatoriophycideae	Oscillatoriales	Microcoleaceae	Microcoleus	CRISPR-associated endonuclease Cas1	**Cas1**	cd09634	CAS-I,CAS-II,CAS-III,CAS-V					
13029-> 13310	+	AFZ22160.1	CP_003633	Microcoleus sp. PCC7113	Bacteria	Cyanobacteria	Oscillatoriophycideae	Oscillatoriales	Microcoleaceae	Microcoleus	CRISPR-associated endonuclease Cas2	**Cas2**	mkCas0081	CAS-I, CAS-II, CAS-III					
13462-> 14001	+	AFZ22161.1	CP_003633	Microcoleus sp. PCC7113	Bacteria	Cyanobacteria	Oscillatoriophycideae	Oscillatoriales	Microcoleaceae	Microcoleus	H								
14091-> 14393	+	AFZ22162.1	CP_003633	Microcoleus sp. PCC7113	Bacteria	Cyanobacteria	Oscillatoriophycideae	Oscillatoriales	Microcoleaceae	Microcoleus	H								
14671-> 15114	+	AFZ22163.1	CP_003633	Microcoleus sp. PCC7113	Bacteria	Cyanobacteria	Oscillatoriophycideae	Oscillatoriales	Microcoleaceae	Microcoleus	H								
16257-> 15495	-		CP_003633	Microcoleus sp. PCC7113	Bacteria	Cyanobacteria	Oscillatoriophycideae	Oscillatoriales	Microcoleaceae	Microcoleus		CRISPR Array			10(35-39)	11 (35)	NA/F18/C	GTTTCCGTCCCCGTAAAGGGGAAGTTAATTGAAAC	H
16431 -> 17270	+	AFZ22164.1	CP_003633	Microcoleus sp. PCC7113	Bacteria	Cyanobacteria	Oscillatoriophycideae	Oscillatoriales	Microcoleaceae	Microcoleus	CRISPR-associated protein	**Csx1**	pfam09670	CAS-III					
18398-> 17271	-	AFZ22165.1	CP_003633	Microcoleus sp. PCC7113	Bacteria	Cyanobacteria	Oscillatoriophycideae	Oscillatoriales	Microcoleaceae	Microcoleus	CRISPR-associated endoribonuclease Cas6	**Cas6**	COG5551	CAS-III					
19199-> 18579	-	AFZ22166.1	CP_003633	Microcoleus sp. PCC7113	Bacteria	Cyanobacteria	Oscillatoriophycideae	Oscillatoriales	Microcoleaceae	Microcoleus	H	**Csx21**	mkCas0116	CAS-III-A, CAS-III-D					
20306 -> 19200	-	AFZ22167.1	CP_003633	Microcoleus sp. PCC7113	Bacteria	Cyanobacteria	Oscillatoriophycideae	Oscillatoriales	Microcoleaceae	Microcoleus	RAMP superfamily protein	**Csm3gr7**	cls000253,cls000253	CAS-III-A, CAS-III-D					
20743 -> 20303	-	AFZ22168.1	CP_003633	Microcoleus sp. PCC7113	Bacteria	Cyanobacteria	Oscillatoriophycideae	Oscillatoriales	Microcoleaceae	Microcoleus	H	**Csx19**	cls000059	CAS-III-D					
21693-> 20740	-	AFZ22169.1	CP_003633	Microcoleus sp. PCC7113	Bacteria	Cyanobacteria	Oscillatoriophycideae	Oscillatoriales	Microcoleaceae	Microcoleus	RAMP superfamily protein	**Csm3gr7**	cd09683	CAS-III-A, CAS-III-D					
22343-> 21723	-	AFZ22170.1	CP_003633	Microcoleus sp. PCC7113	Bacteria	Cyanobacteria	Oscillatoriophycideae	Oscillatoriales	Microcoleaceae	Microcoleus	H	**PD-DExK**	mkCas0061	CAS-I, CAS-III					
22991 -> 22371	-	AFZ22171.1	CP_003633	Microcoleus sp. PCC7113	Bacteria	Cyanobacteria	Oscillatoriophycideae	Oscillatoriales	Microcoleaceae	Microcoleus	H	**PD-DExK**	mkCas0061	CAS-I, CAS-III					
23871-> 22978	-	AFZ22172.1	CP_003633	Microcoleus sp. PCC7113	Bacteria	Cyanobacteria	Oscillatoriophycideae	Oscillatoriales	Microcoleaceae	Microcoleus	CRISPR-associated RAMP protein	**Csm3gr7**	cd09683	CAS-III-A, CAS-III-D					
25030-> 23909	-	AFZ22173.1	CP_003633	Microcoleus sp. PCC7113	Bacteria	Cyanobacteria	Oscillatoriophycideae	Oscillatoriales	Microcoleaceae	Microcoleus	CRISPR-associated protein	**Csm6**	cd09742	CAS-III-A, CAS-III-D					
25489->25076	-	AFZ22174.1	CP_003633	Microcoleus sp. PCC7113	Bacteria	Cyanobacteria	Oscillatoriophycideae	Oscillatoriales	Microcoleaceae	Microcoleus	H	**Csm2gr11**	mkCas0063	CAS-III-D					
26811-> 25486	-	AFZ22175.1	CP_003633	Microcoleus sp. PCC7113	Bacteria	Cyanobacteria	Oscillatoriophycideae	Oscillatoriales	Microcoleaceae	Microcoleus	CRISPR-associated RAMP protein Csx10	**Csx10gr5**	cls000715	CAS-III-D					
27524-> 26808	-	AFZ22176.1	CP_003633	Microcoleus sp. PCC7113	Bacteria	Cyanobacteria	Oscillatoriophycideae	Oscillatoriales	Microcoleaceae	Microcoleus	RAMP superfamily protein	**Csm3gr7**	cd09683	CAS-III-A, CAS-III-D					
29835-> 27529	-	AFZ22177.1	CP_003633	Microcoleus sp. PCC7113	Bacteria	Cyanobacteria	Oscillatoriophycideae	Oscillatoriales	Microcoleaceae	Microcoleus	type III-B CRISPR-associated protein Cas10/Cmr2	**Cas10**	cls000742	CAS-III					
**CAS-III-B**																			
1597 -> 2058	+	WP_007355610.1	NZ_CACA01000266	[Oscillatoria] sp. PCC 6506	Bacteria	Cyanobacteria	Oscillatoriophycideae	Oscillatoriales	Microcoleaceae	Kamptonema	CRISPR-associated endonuclease Cas1	**Cas1**	pfam01867	CAS-I, CAS-II, CAS-III					

2612 -> 3343	+	WP_007355612.1	NZ_CACA01000266	[Oscillatoria] sp. PCC 6506	Bacteria	Cyanobacteria	Oscillatoriophycideae	Oscillatoriales	Microcoleaceae	Kamptonema	H								
3361 -> 4563	+	WP_007355613.1	NZ_CACA01000266	[Oscillatoria] sp. PCC 6506	Bacteria	Cyanobacteria	Oscillatoriophycideae	Oscillatoriales	Microcoleaceae	Kamptonema	H								
4691 -> 6508	+	WP_007355614.1	NZ_CACA01000266	[Oscillatoria] sp. PCC 6506	Bacteria	Cyanobacteria	Oscillatoriophycideae	Oscillatoriales	Microcoleaceae	Kamptonema	H								
6505 -> 7578	+	WP_007355615.1	NZ_CACA01000266	[Oscillatoria] sp. PCC 6506	Bacteria	Cyanobacteria	Oscillatoriophycideae	Oscillatoriales	Microcoleaceae	Kamptonema	H								
7601 -> 8620	+	WP_007355616.1	NZ_CACA01000266	[Oscillatoria] sp. PCC 6506	Bacteria	Cyanobacteria	Oscillatoriophycideae	Oscillatoriales	Microcoleaceae	Kamptonema	CRISPR-associated protein	**Cmr4gr7**	COG1336	CAS-III-C					
8704 -> 9297	+	WP_007355617.1	NZ_CACA01000266	[Oscillatoria] sp. PCC 6506	Bacteria	Cyanobacteria	Oscillatoriophycideae	Oscillatoriales	Microcoleaceae	Kamptonema	H								
11248 -> 9539	-	WP_007355618.1	NZ_CACA01000266	[Oscillatoria] sp. PCC 6506	Bacteria	Cyanobacteria	Oscillatoriophycideae	Oscillatoriales	Microcoleaceae	Kamptonema	H	**Csm3gr7**	pfam03787	CAS-III-A, CAS-III-D					
12113->11429	-		NZ_CACA01000266	[Oscillatoria] sp. PCC 6506	Bacteria	Cyanobacteria	Oscillatoriophycideae	Oscillatoriales	Microcoleaceae	Kamptonema		CRISPR Array			8 (35,39)	9(35)	NA/NA/C	GTTTGACCCTTACTAAAGGGAAAAGGTGCTGAAAC	NA
14368 -> 12365	-	WP_007355619.1	NZ_CACA01000266	[Oscillatoria] sp. PCC 6506	Bacteria	Cyanobacteria	Oscillatoriophycideae	Oscillatoriales	Microcoleaceae	Kamptonema	RTCas1	**RT-Cas1**	pfam00078, pfam01867	RT; CAS-I, CAS-II, CAS-III					
**CAS-III-B**																			
2897927 ->2897528	-		NC_016640	Arthrospira platensis NIES-39	Bacteria	Cyanobacteria	Oscillatoriophycideae	Oscillatoriales	Microcoleaceae	Arthrospira		CRISPR Array			5 (35-41)	6 (35)	NA/NA/C	CTTTAAACTTCTTTGAAAGTTAAACGTATGGAAAC	NA
2899929->2898130	-	WP_014275551.1	NC_016640	Arthrospira platensis NIES-39	Bacteria	Cyanobacteria	Oscillatoriophycideae	Oscillatoriales	Microcoleaceae	Arthrospira	CRISPR-associated endonuclease Cas1	**RT-Cas1**	pfam0078,COG1518	CAS-III; CAS-I, CAS-II, CAS-III					
2900316->2901212	+	WP_006616612.1	NC_016640	Arthrospira platensis NIES-39	Bacteria	Cyanobacteria	Oscillatoriophycideae	Oscillatoriales	Microcoleaceae	Arthrospira	CRISPR-associated endonuclease Cas1	**Cas1**	COG1518	CAS-I, CAS-II, CAS-III					
2901209->2901490	+	WP_006616611.1	NC_016640	Arthrospira platensis NIES-39	Bacteria	Cyanobacteria	Oscillatoriophycideae	Oscillatoriales	Microcoleaceae	Arthrospira	CRISPR-associated endonuclease Cas2	**Cas2**	COG1343	CAS-I, CAS-II, CAS-III					
2901539->2901799	+	WP_006616610.1	NC_016640	Arthrospira platensis NIES-39	Bacteria	Cyanobacteria	Oscillatoriophycideae	Oscillatoriales	Microcoleaceae	Arthrospira	H								
2901900->2902421	+	WP_014275552.1	NC_016640	Arthrospira platensis NIES-39	Bacteria	Cyanobacteria	Oscillatoriophycideae	Oscillatoriales	Microcoleaceae	Arthrospira	H								
2902414->2902713	+	WP_006616608.1	NC_016640	Arthrospira platensis NIES-39	Bacteria	Cyanobacteria	Oscillatoriophycideae	Oscillatoriales	Microcoleaceae	Arthrospira	H								
2902792->2902974	+	WP_006616607.1	NC_016640	Arthrospira platensis NIES-39	Bacteria	Cyanobacteria	Oscillatoriophycideae	Oscillatoriales	Microcoleaceae	Arthrospira	H								
2904079-> 2903090	-		NC_016640	Arthrospira platensis NIES-39	Bacteria	Cyanobacteria	Oscillatoriophycideae	Oscillatoriales	Microcoleaceae	Arthrospira		CRISPR Array			13 (34-43)	14 (35)	NA/NA/C	CTTTAGACTTCTCTGAAAGTTAAACGTATGGAAAC	H
2904626->2904336	-	WP_006617696.1	NC_016640	Arthrospira platensis NIES-39	Bacteria	Cyanobacteria	Oscillatoriophycideae	Oscillatoriales	Microcoleaceae	Arthrospira	H								
2905452->2904733	-	WP_006617695.1	NC_016640	Arthrospira platensis NIES-39	Bacteria	Cyanobacteria	Oscillatoriophycideae	Oscillatoriales	Microcoleaceae	Arthrospira	H								
2906103->2905525	-	WP_006617694.1	NC_016640	Arthrospira platensis NIES-39	Bacteria	Cyanobacteria	Oscillatoriophycideae	Oscillatoriales	Microcoleaceae	Arthrospira	H								
2906998->2906354	-	WP_006617693.1	NC_016640	Arthrospira platensis NIES-39	Bacteria	Cyanobacteria	Oscillatoriophycideae	Oscillatoriales	Microcoleaceae	Arthrospira	H								
2909029->2907060	-		NC_016640	Arthrospira platensis NIES-39	Bacteria	Cyanobacteria	Oscillatoriophycideae	Oscillatoriales	Microcoleaceae	Arthrospira	type III-B CRISPR module RAMP protein Cmr6	pseudo							
2909429->2909034	-	WP_006617692.1	NC_016640	Arthrospira platensis NIES-39	Bacteria	Cyanobacteria	Oscillatoriophycideae	Oscillatoriales	Microcoleaceae	Arthrospira	H	**Cmr5gr11**	cls000022	CAS-III-B					
2910194->2909445	-	WP_006617691.1	NC_016640	Arthrospira platensis NIES-39	Bacteria	Cyanobacteria	Oscillatoriophycideae	Oscillatoriales	Microcoleaceae	Arthrospira	type III-B CRISPR module RAMP protein Cmr4	**Cmr4gr7**	cd09682	CAS-III-B					
2910567->2910343	-	WP_006617690.1	NC_016640	Arthrospira platensis NIES-39	Bacteria	Cyanobacteria	Oscillatoriophycideae	Oscillatoriales	Microcoleaceae	Arthrospira	CRISPR-associated protein Cmr4	**Csm3gr7**	cd09726	CAS-III-D					
2911727->2910564	-	WP_006617689.1	NC_016640	Arthrospira platensis NIES-39	Bacteria	Cyanobacteria	Oscillatoriophycideae	Oscillatoriales	Microcoleaceae	Arthrospira	H	**Cmr3gr5**	cd09748	CAS-III-B					
2915057->2911728	-	WP_008049614.1	NC_016640	Arthrospira platensis NIES-39	Bacteria	Cyanobacteria	Oscillatoriophycideae	Oscillatoriales	Microcoleaceae	Arthrospira	type III-B CRISPR-associated protein Cas10/Cmr2	**Cas10**	cls000742	CAS-III					
**Unknown**																			
1125->233	-		NZ_AQPY01001269	Microcystis aeruginosa PCC 7005	Bacteria	Cyanobacteria	Oscillatoriophycideae	Chroococcales	Microcystaceae	Microcystis		CRISPR Array			12 (34-39)	13 (35)	NA/NA/C	CTTCTGACTTCCTCGGAAGTTGAATTAATGGAAAC	M
3317 -> 1341	-	WP_024971209.1	NZ_AQPY01001269	Microcystis aeruginosa PCC 7005	Bacteria	Cyanobacteria	Oscillatoriophycideae	Chroococcales	Microcystaceae	Microcystis	CRISPR-associated endonuclease Cas1	**RT-Cas1**	pfam00078, COG1518	CAS-III; CAS-I, CAS-II, CAS-III					
**CAS-III-D**																			
3213 -> 5183	+	KPQ33062.1	LJZR01000039	Phormidesmis priestleyi Ana ITZX	Bacteria	Cyanobacteria		Synechococcales	Leptolyngbyaceae	Phormidesmis	cas1-2	**RT-Cas1**	pfam00078, COG1518	CAS-III; CAS-I, CAS-II, CAS-III					
5262 -> 5540	+	KPQ33063.1	LJZR01000039	Phormidesmis priestleyi Ana ITZX	Bacteria	Cyanobacteria		Synechococcales	Leptolyngbyaceae	Phormidesmis	cas2-2	**Cas2**	COG1343	CAS-I, CAS-II, CAS-III					
5612 -> 6028	+	KPQ33064.1	LJZR01000039	Phormidesmis priestleyi Ana ITZX	Bacteria	Cyanobacteria		Synechococcales	Leptolyngbyaceae	Phormidesmis	four helix bundle protein								
6034 -> 6693	+	KPQ33065.1	LJZR01000039	Phormidesmis priestleyi Ana ITZX	Bacteria	Cyanobacteria		Synechococcales	Leptolyngbyaceae	Phormidesmis	H								
7172 -> 7663	+	KPQ33066.1	LJZR01000039	Phormidesmis priestleyi Ana ITZX	Bacteria	Cyanobacteria		Synechococcales	Leptolyngbyaceae	Phormidesmis	H								
7055->9448	+		LJZR01000039	Phormidesmis priestleyi Ana ITZX	Bacteria	Cyanobacteria		Synechococcales	Leptolyngbyaceae	Phormidesmis		CRISPR Array			33 (33-41)	34 (35)	M4/NA/NA	GTTCAAATTCCATTACCCCGCAAGGGGACGGAAAC	M
9674 -> 11950	+	KPQ33067.1	LJZR01000039	Phormidesmis priestleyi Ana ITZX	Bacteria	Cyanobacteria		Synechococcales	Leptolyngbyaceae	Phormidesmis	Tetratricopeptide repeat								
13399 -> 11954	-	KPQ33068.1	LJZR01000039	Phormidesmis priestleyi Ana ITZX	Bacteria	Cyanobacteria		Synechococcales	Leptolyngbyaceae	Phormidesmis	CRISPR-associated protein, TIGR03985 family	**WYL**	mkCas0075	CAS-I, CAS-III					
13448 -> 14467	+	KPQ33069.1	LJZR01000039	Phormidesmis priestleyi Ana ITZX	Bacteria	Cyanobacteria		Synechococcales	Leptolyngbyaceae	Phormidesmis	csx3	**Csx3**	mkCas0115	CAS-I, CAS-III					
14571 -> 17030	+	KPQ33070.1	LJZR01000039	Phormidesmis priestleyi Ana ITZX	Bacteria	Cyanobacteria		Synechococcales	Leptolyngbyaceae	Phormidesmis	cmr2-2	**Cas10**	mkCas0133						
17027 -> 17878	+	KPQ33071.1	LJZR01000039	Phormidesmis priestleyi Ana ITZX	Bacteria	Cyanobacteria		Synechococcales	Leptolyngbyaceae	Phormidesmis	subtype III-B CRISPR-associated RAMP protein	**Csm3gr7**	cd09683	CAS-III-A, CAS-III-D					
17875 -> 19137	+	KPQ33072.1	LJZR01000039	Phormidesmis priestleyi Ana ITZX	Bacteria	Cyanobacteria		Synechococcales	Leptolyngbyaceae	Phormidesmis	csx10	**Csx10gr5**	cls000715	CAS-III-D					
19134 -> 19628	+	KPQ33073.1	LJZR01000039	Phormidesmis priestleyi Ana ITZX	Bacteria	Cyanobacteria		Synechococcales	Leptolyngbyaceae	Phormidesmis	H		mkCas0063						
19632 -> 20528	+	KPQ33074.1	LJZR01000039	Phormidesmis priestleyi Ana ITZX	Bacteria	Cyanobacteria		Synechococcales	Leptolyngbyaceae	Phormidesmis	csm3	**Csm3gr7**	cd09683	CAS-III-A, CAS-III-D					
20543 -> 21517	+	KPQ33075.1	LJZR01000039	Phormidesmis priestleyi Ana ITZX	Bacteria	Cyanobacteria		Synechococcales	Leptolyngbyaceae	Phormidesmis	subtype III-B CRISPR-associated RAMP protein	**Csm3gr7**	cd09683	CAS-III-A, CAS-III-D					
21514 -> 21969	+	KPQ33076.1	LJZR01000039	Phormidesmis priestleyi Ana ITZX	Bacteria	Cyanobacteria		Synechococcales	Leptolyngbyaceae	Phormidesmis	H	**Csm2gr11**	mkCas0063	CAS-III-D					
21959 -> 22996	+	KPQ33077.1	LJZR01000039	Phormidesmis priestleyi Ana ITZX	Bacteria	Cyanobacteria		Synechococcales	Leptolyngbyaceae	Phormidesmis	subtype III-B CRISPR-associated RAMP protein	**Csm3gr7**	cls000253, cls000253	CAS-III-A, CAS-III-D; CAS-III-A, CAS-III-D					
22996 -> 23652	+	KPQ33078.1	LJZR01000039	Phormidesmis priestleyi Ana ITZX	Bacteria	Cyanobacteria		Synechococcales	Leptolyngbyaceae	Phormidesmis	H	**Csx21**	mkCas0116	CAS-III-A, CAS-III-D					
25240 -> 23882	-	KPQ33079.1	LJZR01000039	Phormidesmis priestleyi Ana ITZX	Bacteria	Cyanobacteria		Synechococcales	Leptolyngbyaceae	Phormidesmis	transposase								
27105 -> 26185	-	KPQ33080.1	LJZR01000039	Phormidesmis priestleyi Ana ITZX	Bacteria	Cyanobacteria		Synechococcales	Leptolyngbyaceae	Phormidesmis	H								
27379 -> 28263	+	KPQ33081.1	LJZR01000039	Phormidesmis priestleyi Ana ITZX	Bacteria	Cyanobacteria		Synechococcales	Leptolyngbyaceae	Phormidesmis	transposase								
29624 -> 28284	-	KPQ33082.1	LJZR01000039	Phormidesmis priestleyi Ana ITZX	Bacteria	Cyanobacteria		Synechococcales	Leptolyngbyaceae	Phormidesmis	cas6	**Cas6**	pfam10040	CAS-III, CAS-IV					
30197 ->33024	+		LJZR01000039	Phormidesmis priestleyi Ana ITZX	Bacteria	Cyanobacteria		Synechococcales	Leptolyngbyaceae	Phormidesmis		CRISPR Array			39 (33-42)	40 (35)	M4/NA/NA	GTTCAAATTCCATTACCCCGAAAGGGGACGGAAAC	M
**Partial**																			
32777->31533	-		NZ_KI913950	Leptolyngbya sp. PCC 6406	Bacteria	Cyanobacteria		Synechococcales	Leptolyngbyaceae	Leptolyngbya		CRISPR Array			16 (33-41)	17 (35)	M4/NA/NA	GTTCAAAATCCATTACCCCGCAAGGGGACGGAAAC	M
33758 -> 33027	-	WP_008312846.1	NZ_KI913950	Leptolyngbya sp. PCC 6406	Bacteria	Cyanobacteria		Synechococcales	Leptolyngbyaceae	Leptolyngbya	H								
34149 -> 33763	-	WP_008312849.1	NZ_KI913950	Leptolyngbya sp. PCC 6406	Bacteria	Cyanobacteria		Synechococcales	Leptolyngbyaceae	Leptolyngbya	four helix boundle protein								
34765 -> 34346	-	WP_008312851.1	NZ_KI913950	Leptolyngbya sp. PCC 6406	Bacteria	Cyanobacteria		Synechococcales	Leptolyngbyaceae	Leptolyngbya	four helix boundle protein								
35058 -> 34780	-	WP_008312853.1	NZ_KI913950	Leptolyngbya sp. PCC 6406	Bacteria	Cyanobacteria		Synechococcales	Leptolyngbyaceae	Leptolyngbya	CRISPR-associated endonuclease Cas2	**Cas2**	COG1343	CAS-I, CAS-II, CAS-III					
37037 -> 35058	-	WP_008312855.1	NZ_KI913950	Leptolyngbya sp. PCC 6406	Bacteria	Cyanobacteria		Synechococcales	Leptolyngbyaceae	Leptolyngbya	CRISPR-associated endonuclease Cas1	**RT-Cas1**	pfam00078, cd09634	CAS-III; CAS-I, CAS-II, CAS-III, CAS-V					

RT-CRISPR-4 (CAS-III-A/D)

Planctomycetia (Candidatus Brocardiales); Deltaproteobacteria (Syntrophobacterales); Gammaproteobacteria (Methylococcales); Chlorobia (Chlorobiales); Tectomicrobia

[a](Sub) Type/[b]Coordinates	Strand	Protein_id	Genome Partition ID	Strain	Domain	Phylum	Lineage (class)	Order	Family	Genus	[c]Cas gene (annotated)	[d]Cas gene/[e]Array (identified)	[d]Profiles	[d](Sub)Type-specificity	[e]Spacers (length)	[e]Repeats (length)	[f]Str.motif/Seq.family/Superclass	[f]Seq Repeats	[g]Orientation Confidence
**CAS-III-A**																			
1438988 -> 1439950	+	CAJ74563.1	CT573071.1	Candidatus Kuenenia stuttgartiensis	Bacteria	Planctomycetes	Planctomycetia	Candidatus Brocardiales	Candidatus Brocadiaceae	Candidatus Kuenenia	H	**Cas6**	mkcas0066	CAS-I, CAS-III					
1440384 -> 1440172	-	CAJ74564.1	CT573071.1	Candidatus Kuenenia stuttgartiensis	Bacteria	Planctomycetes	Planctomycetia	Candidatus Brocardiales	Candidatus Brocadiaceae	Candidatus Kuenenia	unknown protein								
1440242 -> 1440580	+		CT573071.1	Candidatus Kuenenia stuttgartiensis	Bacteria	Planctomycetes	Planctomycetia	Candidatus Brocardiales	Candidatus Brocadiaceae	Candidatus Kuenenia		CRISPR Array			4 (38-42)	5 (37)	M16/NA/E	GTGGAAATCATTGACCTGATGTAGAAGGGATTGAGAC	H
1440637 -> 1440819	+	CAJ74565.1	CT573071.1	Candidatus Kuenenia stuttgartiensis	Bacteria	Planctomycetes	Planctomycetia	Candidatus Brocardiales	Candidatus Brocadiaceae	Candidatus Kuenenia	unknown protein								
1440872 -> 1440937	+	CAJ74566.1	CT573071.1	Candidatus Kuenenia stuttgartiensis	Bacteria	Planctomycetes	Planctomycetia	Candidatus Brocardiales	Candidatus Brocadiaceae	Candidatus Kuenenia	unknown protein								
1441110 -> 1440958	-	CAJ74567.1	CT573071.1	Candidatus Kuenenia stuttgartiensis	Bacteria	Planctomycetes	Planctomycetia	Candidatus Brocardiales	Candidatus Brocadiaceae	Candidatus Kuenenia	unknown protein								
1441293 -> 1443803	+	CAJ74568.1	CT573071.1	Candidatus Kuenenia stuttgartiensis	Bacteria	Planctomycetes	Planctomycetia	Candidatus Brocardiales	Candidatus Brocadiaceae	Candidatus Kuenenia	H	**Cas10**	cd09680	CAS-III					
1443825 -> 1444259	+	CAJ74569.1	CT573071.1	Candidatus Kuenenia stuttgartiensis	Bacteria	Planctomycetes	Planctomycetia	Candidatus Brocardiales	Candidatus Brocadiaceae	Candidatus Kuenenia	H	**Csm2gr11**	pfam03750	CAS-III-A					
1444278 -> 1445075	+	CAJ74570.1	CT573071.1	Candidatus Kuenenia stuttgartiensis	Bacteria	Planctomycetes	Planctomycetia	Candidatus Brocardiales	Candidatus Brocadiaceae	Candidatus Kuenenia	H	**Csm3gr7**	cd09684	CAS-III-A, CAS-III-D					
1445085 -> 1446059	+	CAJ74571.1	CT573071.1	Candidatus Kuenenia stuttgartiensis	Bacteria	Planctomycetes	Planctomycetia	Candidatus Brocardiales	Candidatus Brocadiaceae	Candidatus Kuenenia	unknown protein *	**Csm4gr5**	cd09663	CAS-III-A					
1446070 -> 1447239	+	CAJ74572.1	CT573071.1	Candidatus Kuenenia stuttgartiensis	Bacteria	Planctomycetes	Planctomycetia	Candidatus Brocardiales	Candidatus Brocadiaceae	Candidatus Kuenenia	H	**Csm5gr7**	cd09662	CAS-III-A					
1447262 -> 1447657	+	CAJ74573.1	CT573071.1	Candidatus Kuenenia stuttgartiensis	Bacteria	Planctomycetes	Planctomycetia	Candidatus Brocardiales	Candidatus Brocadiaceae	Candidatus Kuenenia	H								
1447665 -> 1449050	+	CAJ74574.1	CT573071.1	Candidatus Kuenenia stuttgartiensis	Bacteria	Planctomycetes	Planctomycetia	Candidatus Brocardiales	Candidatus Brocadiaceae	Candidatus Kuenenia	H	**Csx1**	mkCas0109	CAS-III					
1449052 -> 1449192	+	CAJ74575.1	CT573071.1	Candidatus Kuenenia stuttgartiensis	Bacteria	Planctomycetes	Planctomycetia	Candidatus Brocardiales	Candidatus Brocadiaceae	Candidatus Kuenenia	unknown protein								
1449714 -> 1450619	+	CAJ74576.1	CT573071.1	Candidatus Kuenenia stuttgartiensis	Bacteria	Planctomycetes	Planctomycetia	Candidatus Brocardiales	Candidatus Brocadiaceae	Candidatus Kuenenia	unknown protein*	Csx1	pfam09670						
1450616 -> 1450855	+	CAJ74577.1	CT573071.1	Candidatus Kuenenia stuttgartiensis	Bacteria	Planctomycetes	Planctomycetia	Candidatus Brocardiales	Candidatus Brocadiaceae	Candidatus Kuenenia	unknown protein	Csx3	cd09681						
1450852 -> 1451802	+	CAJ74578.1	CT573071.1	Candidatus Kuenenia stuttgartiensis	Bacteria	Planctomycetes	Planctomycetia	Candidatus Brocardiales	Candidatus Brocadiaceae	Candidatus Kuenenia	H	**RT**	pfam00078	CAS-III					
1451799 -> 1452785	+	CAJ74579.1	CT573071.1	Candidatus Kuenenia stuttgartiensis	Bacteria	Planctomycetes	Planctomycetia	Candidatus Brocardiales	Candidatus Brocadiaceae	Candidatus Kuenenia	H	**Cas1**	cd09634	CAS-I, CAS-II, CAS-III, CAS-V					
1452789 -> 1453064	+	CAJ74580.1	CT573071.1	Candidatus Kuenenia stuttgartiensis	Bacteria	Planctomycetes	Planctomycetia	Candidatus Brocardiales	Candidatus Brocadiaceae	Candidatus Kuenenia	H	**Cas2**	mkCas0081	CAS-I, CAS-II, CAS-III					
1453270 -> 1453903	+		CT573071.1	Candidatus Kuenenia stuttgartiensis	Bacteria	Planctomycetes	Planctomycetia	Candidatus Brocardiales	Candidatus Brocadiaceae	Candidatus Kuenenia		CRISPR Array			8 (34-44)	9 (37)	M16/NA/E	GTGGAAATCATTGACCTGATGTAGAAGGGATTGAGAC	M
**Partial**																			
1481961-> 1482950	+	WP_013706256.1	NC_015388.1	Desulfobacca acetoxidans DSM 11109	Bacteria	Proteobacteria	Deltaproteobacteria	Syntrophobacterales	Syntrophaceae	Desulfobacca	H	**Cas6**	mkCas0066	CAS-I, CAS-III					
1482947-> 1483279	+	WP_013706257.1	NC_015388.1	Desulfobacca acetoxidans DSM 11109	Bacteria	Proteobacteria	Deltaproteobacteria	Syntrophobacterales	Syntrophaceae	Desulfobacca	H								
1483300-> 1483536	+	WP_013706258.1	NC_015388.1	Desulfobacca acetoxidans DSM 11109	Bacteria	Proteobacteria	Deltaproteobacteria	Syntrophobacterales	Syntrophaceae	Desulfobacca	DUF4926 domain-containing protein								
1483793-> 1484182	+	WP_013706260.1	NC_015388.1	Desulfobacca acetoxidans DSM 11109	Bacteria	Proteobacteria	Deltaproteobacteria	Syntrophobacterales	Syntrophaceae	Desulfobacca	H								
1484521-> 1485555	+	WP_013706261.1	NC_015388.1	Desulfobacca acetoxidans DSM 11109	Bacteria	Proteobacteria	Deltaproteobacteria	Syntrophobacterales	Syntrophaceae	Desulfobacca	H								
1485545-> 1486504	+	WP_013706262.1	NC_015388.1	Desulfobacca acetoxidans DSM 11109	Bacteria	Proteobacteria	Deltaproteobacteria	Syntrophobacterales	Syntrophaceae	Desulfobacca	RNA-directed DNA polymerase	**RT**	pfam00078	CAS-III					
1486581-> 1486781	+	WP_013706263.1	NC_015388.1	Desulfobacca acetoxidans DSM 11109	Bacteria	Proteobacteria	Deltaproteobacteria	Syntrophobacterales	Syntrophaceae	Desulfobacca	H								
1486768-> 1487028	+	WP_013706264.1	NC_015388.1	Desulfobacca acetoxidans DSM 11109	Bacteria	Proteobacteria	Deltaproteobacteria	Syntrophobacterales	Syntrophaceae	Desulfobacca	addiction module toxin RelE								
1487143-> 1488144	+	WP_013706265.1	NC_015388.1	Desulfobacca acetoxidans DSM 11109	Bacteria	Proteobacteria	Deltaproteobacteria	Syntrophobacterales	Syntrophaceae	Desulfobacca	CRISPR-associated endonuclease Cas1	**Cas1**	pfam01867	CAS-I, CAS-II, CAS-III					
1488148-> 1488426	+	WP_013706266.1	NC_015388.1	Desulfobacca acetoxidans DSM 11109	Bacteria	Proteobacteria	Deltaproteobacteria	Syntrophobacterales	Syntrophaceae	Desulfobacca	CRISPR-associated endonuclease Cas2	**Cas2**	mkCas0081	CAS-I, CAS-II, CAS-III					
1488840-> 1490146	+		NC_015388.1	Desulfobacca acetoxidans DSM 11109	Bacteria	Proteobacteria	Deltaproteobacteria	Syntrophobacterales	Syntrophaceae	Desulfobacca		CRISPR Array			17 (33-40)	18 (37)	NA/NA/E	CTTACAACGATAAGAAGAGTCTTCGGACGATTGCGAC	H
**CAS-III-D**																			
4307564-> 4307952	+		NZ_JMLA01000001.1	Methylobacter tundripaludum	Bacteria	Proteobacteria	Gammaproteobacteria	Methylococcales	Mthylococcaceae	Methylobacter		CRISPR Array			5 (33,35)	6 (36)	NA/NA/NA	GTTGCACCGCCCGGACTTTGATAACGGGATGAAAAC	H
4308265-> 4309233	+	WP_027150705.1	NZ_JMLA01000001.1	Methylobacter tundripaludum	Bacteria	Proteobacteria	Gammaproteobacteria	Methylococcales	Mthylococcaceae	Methylobacter	H	**Cas6**	mkCas0066	CAS-I, CAS-III					
4309407-> 4311038	+	WP_027150706.1	NZ_JMLA01000001.1	Methylobacter tundripaludum	Bacteria	Proteobacteria	Gammaproteobacteria	Methylococcales	Mthylococcaceae	Methylobacter	H								
4311035-> 4312615	+	WP_027150707.1	NZ_JMLA01000001.1	Methylobacter tundripaludum	Bacteria	Proteobacteria	Gammaproteobacteria	Methylococcales	Mthylococcaceae	Methylobacter	H	**Cas10**	cls000707	CAS-III					
4312608-> 4314476	+	WP_027150708.1	NZ_JMLA01000001.1	Methylobacter tundripaludum	Bacteria	Proteobacteria	Gammaproteobacteria	Methylococcales	Mthylococcaceae	Methylobacter	H	**Csm4gr5**	cls000259	CAS-III-D					
4314481-> 4318146	+	WP_027150709.1	NZ_JMLA01000001.1	Methylobacter tundripaludum	Bacteria	Proteobacteria	Gammaproteobacteria	Methylococcales	Mthylococcaceae	Methylobacter	CRISPR-associated RAMP family protein	**Csm3gr7**	cls000253	CAS-III-A, CAS-III-D					
4318172-> 4318468	+	WP_027150710.1	NZ_JMLA01000001.1	Methylobacter tundripaludum	Bacteria	Proteobacteria	Gammaproteobacteria	Methylococcales	Mthylococcaceae	Methylobacter	H								
4318688-> 4319004	+		NZ_JMLA01000001.1	Methylobacter tundripaludum	Bacteria	Proteobacteria	Gammaproteobacteria	Methylococcales	Mthylococcaceae	Methylobacter		CRISPR Array			4 (33-36)	5 (36)	NA/NA/NA	GTTGCACCGCCCGGACTTTGATAACGGGATGAAAAC	H
4319083-> 4321161	+	WP_027150711.1	NZ_JMLA01000001.1	Methylobacter tundripaludum	Bacteria	Proteobacteria	Gammaproteobacteria	Methylococcales	Mthylococcaceae	Methylobacter	CRISPR-associated endonuclease Cas1	**RT-Cas1**	pfam00078, cd09634	CAS-III; CAS-I, CAS-II, CAS-III, CAS-V					
4321158-> 4321436	+	WP_027150712.1	NZ_JMLA01000001.1	Methylobacter tundripaludum	Bacteria	Proteobacteria	Gammaproteobacteria	Methylococcales	Mthylococcaceae	Methylobacter	CRISPR-associated endonuclease Cas2	**Cas2**	mkCas0081	CAS-I, CAS-II, CAS-III					
4321433-> 4321714	+	WP_027150713.1	NZ_JMLA01000001.1	Methylobacter tundripaludum	Bacteria	Proteobacteria	Gammaproteobacteria	Methylococcales	Mthylococcaceae	Methylobacter	CRISPR-associated endonuclease Cas2	**Cas2**	mkCas0081	CAS-I, CAS-II, CAS-III					
4321952-> 4322201	+		NZ_JMLA01000001.1	Methylobacter tundripaludum	Bacteria	Proteobacteria	Gammaproteobacteria	Methylococcales	Mthylococcaceae	Methylobacter		CRISPR Array			4 (34,36)	5 (36)	NA/NA/NA	GTTGCACCGCCCGGACTTTGATAACGGGATGAAAAC	H
**CAS-III-A/CAS-III-D**																			
2532487->2531693	-	WP_012509107.1	NC_011060.1	Pelodictyon phaeoclathratiforme BU-1	Bacteria	Chlorobi	Chlorobia	Chlorobiales	Chlorobiaceae	Pelodictyon	CRISPR-associated endoribonuclease Cas6	**Cas6**	pfam01881	CAS-I, CAS-III					
2534005->2532500	-	WP_012509108.1	NC_011060.1	Pelodictyon phaeoclathratiforme BU-1	Bacteria	Chlorobi	Chlorobia	Chlorobiales	Chlorobiaceae	Pelodictyon	H	**Csx1**	cd09747	CAS-III					
2535276->2533978	-	WP_012509109.1	NC_011060.1	Pelodictyon phaeoclathratiforme BU-1	Bacteria	Chlorobi	Chlorobia	Chlorobiales	Chlorobiaceae	Pelodictyon	H	Csm5gr7	cd09662						
2536346->2535282	-	WP_012509110.1	NC_011060.1	Pelodictyon phaeoclathratiforme BU-1	Bacteria	Chlorobi	Chlorobia	Chlorobiales	Chlorobiaceae	Pelodictyon	type III-A CRISPR-associated RAMP protein Csm4	**Csm4gr5**	cd09663	CAS-III-A					
2537200->2536343	-	WP_012509111.1	NC_011060.1	Pelodictyon phaeoclathratiforme BU-1	Bacteria	Chlorobi	Chlorobia	Chlorobiales	Chlorobiaceae	Pelodictyon	type III-A CRISPR-associated RAMP protein Csm3	**Csm3gr7**	cd09684	CAS-III-A, CAS-III-D					
2537635->2537216	-	WP_012509112.1	NC_011060.1	Pelodictyon phaeoclathratiforme BU-1	Bacteria	Chlorobi	Chlorobia	Chlorobiales	Chlorobiaceae	Pelodictyon	type III-A CRISPR-associated protein Csm2	**Csm2gr11**	pfam03750	CAS-III-A					
2539934->2537649	-	WP_012509113.1	NC_011060.1	Pelodictyon phaeoclathratiforme BU-1	Bacteria	Chlorobi	Chlorobia	Chlorobiales	Chlorobiaceae	Pelodictyon	type III-A CRISPR-associated protein Cas10/Csm1	**Cas10**	cd09680	CAS-III					
2540770->2540375	-	WP_012509114.1	NC_011060.1	Pelodictyon phaeoclathratiforme BU-1	Bacteria	Chlorobi	Chlorobia	Chlorobiales	Chlorobiaceae	Pelodictyon	H	**Csx23**	mkCas0160	CAS-III-D					
2541350->2540778	-	WP_012509115.1	NC_011060.1	Pelodictyon phaeoclathratiforme BU-1	Bacteria	Chlorobi	Chlorobia	Chlorobiales	Chlorobiaceae	Pelodictyon	H	**Csx15**	cd09766	CAS-I, CAS-III					
2541671->2541387	-	WP_012509116.1	NC_011060.1	Pelodictyon phaeoclathratiforme BU-1	Bacteria	Chlorobi	Chlorobia	Chlorobiales	Chlorobiaceae	Pelodictyon	CRISPR-associated endonuclease Cas2	**Cas2**	mkCas0081	CAS-I, CAS-II, CAS-III					
2541825->2544008	+	WP_012509117.1	NC_011060.1	Pelodictyon phaeoclathratiforme BU-1	Bacteria	Chlorobi	Chlorobia	Chlorobiales	Chlorobiaceae	Pelodictyon	CRISPR-associated endonuclease Cas1	**RT-Cas1**	pfam00078, cd09634	CAS-III; CAS-I, CAS-II, CAS-III, CAS-V					
2544259->2546301	+		NC_011060.1	Pelodictyon phaeoclathratiforme BU-1	Bacteria	Chlorobi	Chlorobia	Chlorobiales	Chlorobiaceae	Pelodictyon		CRISPR Array			28 (35-39)	29 (35)	NA/NA/F	CATCATATCTTATTGCACGCATAGCGGATTGAAAC	H
2546436->2546753	+		NC_011060.1	Pelodictyon phaeoclathratiforme BU-1	Bacteria	Chlorobi	Chlorobia	Chlorobiales	Chlorobiaceae	Pelodictyon		CRISPR Array			12 (21-50)	13 (35)	NA/NA/F	CATCATATCTTATTGCACGCATAGCGGATTGAAAC	H
**CAS-III-A**																			
2365503->2364700	-	WP_011745858.1	NC_008639.1	Chlorobium phaeobacteroides DSM 266	Bacteria	Chlorobi	Chlorobia	Chlorobiales	Chlorobiaceae	Chlorobium	CRISPR-associated endoribonuclease Cas6	**Cas6**	pfam01881	CAS-I, CAS-III					
2367130->2365517	-	WP_011745859.1	NC_008639.1	Chlorobium phaeobacteroides DSM 266	Bacteria	Chlorobi	Chlorobia	Chlorobiales	Chlorobiaceae	Chlorobium	H	**Csx1**	cd09702	CAS-III					
2369109->2367139	-	WP_041467343.1	NC_008639.1	Chlorobium phaeobacteroides DSM 266	Bacteria	Chlorobi	Chlorobia	Chlorobiales	Chlorobiaceae	Chlorobium	type III-A CRISPR-associated RAMP protein Csm5	Csm5gr7	cd09662, cd09662						
2370166->2369102	-	WP_011745861.1	NC_008639.1	Chlorobium phaeobacteroides DSM 266	Bacteria	Chlorobi	Chlorobia	Chlorobiales	Chlorobiaceae	Chlorobium	type III-A CRISPR-associated RAMP protein Csm4	**Csm4gr5**	cd09663	CAS-III-A					
2371034->2370177	-	WP_011745862.1	NC_008639.1	Chlorobium phaeobacteroides DSM 266	Bacteria	Chlorobi	Chlorobia	Chlorobiales	Chlorobiaceae	Chlorobium	type III-A CRISPR-associated RAMP protein Csm3	**Csm3gr7**	cd09684	CAS-III-A, CAS-III-D					
2371523->2371047	-	WP_011745863.1	NC_008639.1	Chlorobium phaeobacteroides DSM 266	Bacteria	Chlorobi	Chlorobia	Chlorobiales	Chlorobiaceae	Chlorobium	type III-A CRISPR-associated protein Csm2	**Csm2gr11**	COG1421	CAS-III-A					
2373811->2371523	-	WP_011745864.1	NC_008639.1	Chlorobium phaeobacteroides DSM 266	Bacteria	Chlorobi	Chlorobia	Chlorobiales	Chlorobiaceae	Chlorobium	type III-A CRISPR-associated protein Cas10/Csm1	**Cas10**	cd09680	CAS-III					
2375253->2374660	-	WP_011745865.1	NC_008639.1	Chlorobium phaeobacteroides DSM 266	Bacteria	Chlorobi	Chlorobia	Chlorobiales	Chlorobiaceae	Chlorobium	H								
2375484->2375269	-	WP_041467344.1	NC_008639.1	Chlorobium phaeobacteroides DSM 266	Bacteria	Chlorobi	Chlorobia	Chlorobiales	Chlorobiaceae	Chlorobium	H								

2376310->2375738	-	WP_011745866.1	NC_008639.1	Chlorobium phaeobacteroides DSM 266	Bacteria	Chlorobi	Chlorobia	Chlorobiales	Chlorobiaceae	Chlorobium	H	**Csx15**	cd09766	CAS-I, CAS-III					
2377212->2376349	-	WP_011745867.1	NC_008639.1	Chlorobium phaeobacteroides DSM 266	Bacteria	Chlorobi	Chlorobia	Chlorobiales	Chlorobiaceae	Chlorobium	H	csx1	cd09702						
2379138->2377889	-		NC_008639.1	Chlorobium phaeobacteroides DSM 266	Bacteria	Chlorobi	Chlorobia	Chlorobiales	Chlorobiaceae	Chlorobium		CRISPR Array			16 (37-46)	17 (35)	NA/NA/F	CTTTATATCTTATTGCACTCATAGAGGATTGAAAC	H
2381598->2379403	-	WP_011745868.1	NC_008639.1	Chlorobium phaeobacteroides DSM 266	Bacteria	Chlorobi	Chlorobia	Chlorobiales	Chlorobiaceae	Chlorobium	CRISPR-associated endonuclease Cas1	**RT-Cas1**	pfam00078, pfam01867	CAS-III; CAS-I, CAS-II, CAS-III					
2381923->2381639	-	WP_011745869.1	NC_008639.1	Chlorobium phaeobacteroides DSM 266	Bacteria	Chlorobi	Chlorobia	Chlorobiales	Chlorobiaceae	Chlorobium	CRISPR-associated endonuclease Cas2	**Cas2**	mkCas0081	CAS-I, CAS-II, CAS-III					
2382802->2382131	-	WP_041467345.1	NC_008639.1	Chlorobium phaeobacteroides DSM 266	Bacteria	Chlorobi	Chlorobia	Chlorobiales	Chlorobiaceae	Chlorobium	H								
2383057->2384197	+		NC_008639.1	Chlorobium phaeobacteroides DSM 266	Bacteria	Chlorobi	Chlorobia	Chlorobiales	Chlorobiaceae	Chlorobium		CRISPR Array			16 (33-45)	17 (35)	NA/NA/F	CTTTATATCTTATTGCACTCATAGAGGATTGAAAC	H
**Partial CAS-III-D**																			
188->1	-	ETX03375.1	AZHX01001441.1	Candidatus Entotheonella sp. TSY2	Bacteria	Nitrospinae	Tectomicrobia				H	**Cas2**	mkCas0081	CAS-I, CAS-II, CAS-III					
3124->194	-	ETX03376.1	AZHX01001441.1	Candidatus Entotheonella sp. TSY2	Bacteria	Nitrospinae	Tectomicrobia				H	**RT-Cas1**	pfam00078, cd09634	CAS-III; CAS-I, CAS-II, CAS-III, CAS-V					
4593->3127	-	ETX03377.1	AZHX01001441.1	Candidatus Entotheonella sp. TSY2	Bacteria	Nitrospinae	Tectomicrobia				H	**Csm3gr7**	cls000253	CAS-III-A, CAS-III-D					
7994->6366	-	ETX03370.1	AZHX01001441.1	Candidatus Entotheonella sp. TSY2	Bacteria	Nitrospinae	Tectomicrobia				H	**Csm3gr7**	pfam03787, cd09683	CAS-III-A, CAS-III-D; CAS-III-A, CAS-III-D					
9142->7966	-	ETX03371.1	AZHX01001441.1	Candidatus Entotheonella sp. TSY2	Bacteria	Nitrospinae	Tectomicrobia				H	**Csm4gr5**	cls000259	CAS-III-D					

RT-CRISPR-5 (CAS-III-A/D, CAS-III-B)
Cyanobacteria (Oscillatoriales, Nostocales, Synechoccales)

[a](Sub) Type/[b]Coordinates	Strand	Protein_id	Genome Partition ID	Strain	Domain	Phylum	Lineage (class)	Order	Family	Genus	[c]Cas gene (annotated)	[d]Cas gene/[e]Array (identified)	[d]Profiles	[d](Sub)Type-specificity	[e]Spacers (length)	[e]Repeats (length)	[f]Str.motif/Seq.family/Superclass	[f]Seq Repeats	[g]Orientation Confidence
**Partial**																			
698003->699046	+	WP_015201683.1	NC_019753.1	Crinalium epipsammum PCC 9333	Bacteria	Cyanobacteria	Oscillatoriophycideae	Oscillatoriales	Gomontiellaceae	Crinalium	group II intron reverse transcriptase/maturase	**RT**	pfam00078	CAS-III					
699099->700103	+	WP_015201684.1	NC_019753.2	Crinalium epipsammum PCC 9334	Bacteria	Cyanobacteria	Oscillatoriophycideae	Oscillatoriales	Gomontiellaceae	Crinalium	CRISPR-associated endonuclease Cas1	**Cas1**	cd09634	CAS-I, CAS-II, CAS-III, CAS-V					
700189->700464	+	WP_041225891.1	NC_019753.3	Crinalium epipsammum PCC 9335	Bacteria	Cyanobacteria	Oscillatoriophycideae	Oscillatoriales	Gomontiellaceae	Crinalium	CRISPR-associated endonuclease Cas2	**Cas2**	mkCas0081	CAS-I, CAS-II, CAS-III					
**Partial CAS-III-A**																			
261377->260007	-	WP_049858587.1	NC_011738.1	Cyanothece sp. PCC 7424	Bacteria	Cyanobacteria	Oscillatoriophycideae	Oscillatoriales	Cyanothecaceae	Cyanothece	WYL domain-containing protein	**WYL**	mkCas0075	CAS-I, CAS-III					
261504->262655	+	WP_012599792.1	NC_011738.1	Cyanothece sp. PCC 7424	Bacteria	Cyanobacteria	Oscillatoriophycideae	Oscillatoriales	Cyanothecaceae	Cyanothece	CRISPR-associated protein, APE2256 family	**Csm6**	pfam09651	CAS-III-A					
262869->263274	+		NC_011738.1	Cyanothece sp. PCC 7424	Bacteria	Cyanobacteria	Oscillatoriophycideae	Oscillatoriales	Cyanothecaceae	Cyanothece		CRISPR Array			4 (35-42)	5 (36)	NA/NA/C	CCCTACCTATTGGGTTAAATAGGAATAGTTGGAAAC	L
263337->263669	+	WP_012599793.1	NC_011738.1	Cyanothece sp. PCC 7424	Bacteria	Cyanobacteria	Oscillatoriophycideae	Oscillatoriales	Cyanothecaceae	Cyanothece	H								
263672->264190	+	WP_012599794.1	NC_011738.1	Cyanothece sp. PCC 7424	Bacteria	Cyanobacteria	Oscillatoriophycideae	Oscillatoriales	Cyanothecaceae	Cyanothece	toxin								
264545->265474	+	WP_012599795.1	NC_011738.1	Cyanothece sp. PCC 7424	Bacteria	Cyanobacteria	Oscillatoriophycideae	Oscillatoriales	Cyanothecaceae	Cyanothece	group II intron reverse transcriptase/maturase	**RT**	pfam00078	CAS-III					
265618->266622	+	WP_012599796.1	NC_011738.1	Cyanothece sp. PCC 7424	Bacteria	Cyanobacteria	Oscillatoriophycideae	Oscillatoriales	Cyanothecaceae	Cyanothece	CRISPR-associated endonuclease Cas1	**Cas1**	COG1518	CAS-I, CAS-II, CAS-III					
266623->266898	+	WP_012599797.1	NC_011738.1	Cyanothece sp. PCC 7424	Bacteria	Cyanobacteria	Oscillatoriophycideae	Oscillatoriales	Cyanothecaceae	Cyanothece	CRISPR-associated endonuclease Cas2	**Cas2**	mkCas0081	CAS-I, CAS-II, CAS-III					
**Partial**																			
6175493->6177022	+	WP_015120900.1	NC_019678.1	Rivularia sp. PCC 7116	Bacteria	Cyanobacteria		Nostocales	Rivulariaceae	Rivularia	CRISPR-associated protein	**WYL**	mkCas0075	CAS-I, CAS-III					
6177292->6177882	+	WP_015120901.1	NC_019678.1	Rivularia sp. PCC 7116	Bacteria	Cyanobacteria		Nostocales	Rivulariaceae	Rivularia	H								
6178420->6179382	+	WP_015120902.1	NC_019678.1	Rivularia sp. PCC 7116	Bacteria	Cyanobacteria		Nostocales	Rivulariaceae	Rivularia	Retron-type reverse transcriptase	**RT**	pfam00078	CAS-III					
6179547->6180551	+	WP_044292273.1	NC_019678.1	Rivularia sp. PCC 7116	Bacteria	Cyanobacteria		Nostocales	Rivulariaceae	Rivularia	CRISPR-associated endonuclease Cas1	**Cas1**	COG1518	CAS-I, CAS-II, CAS-III					
6180650->6180925	+	WP_015120904.1	NC_019678.1	Rivularia sp. PCC 7116	Bacteria	Cyanobacteria		Nostocales	Rivulariaceae	Rivularia	CRISPR-associated endonuclease Cas2	**Cas2**	mkCas0081	CAS-I, CAS-II, CAS-III					
6181145->6188351	+		NC_019678.1	Rivularia sp. PCC 7116	Bacteria	Cyanobacteria		Nostocales	Rivulariaceae	Rivularia		CRISPR Array			100 (31-41)	101 (36)	NA/NA/C	CCCCACCGATTGGGTTAATTCGGATTAGTTGGAAAC	NA
**Partial**																			
1329->48	-	WP_009625637.1	NZ_ALWB01000016.1	Pseudanabaena biceps PCC 7429	Bacteria	Cyanobacteria		Synechococcales	Pseudanabaenaceae	Pseudanabaena		CRISPR Array			18 (32-37)	19 (35)	NA/NA/NA	GTTTCAATTTTTTTACCCCGCAAGGGGACGGAAAC	NA
1898->1668	-	WP_040687146.1	NZ_ALWB01000016.1	Pseudanabaena biceps PCC 7429	Bacteria	Cyanobacteria		Synechococcales	Pseudanabaenaceae	Pseudanabaena	H								
2871->1951	-	WP_009625639.1	NZ_ALWB01000016.1	Pseudanabaena biceps PCC 7429	Bacteria	Cyanobacteria		Synechococcales	Pseudanabaenaceae	Pseudanabaena	H	**Csx3**	mkCas0115	CAS-I, CAS-III					
3417->4874	+	WP_040687145.1	NZ_ALWB01000016.1	Pseudanabaena biceps PCC 7429	Bacteria	Cyanobacteria		Synechococcales	Pseudanabaenaceae	Pseudanabaena	WYL domain-containing protein	**WYL**	mkCas0075	CAS-I, CAS-III					
5168->5995	+	WP_009625641.1	NZ_ALWB01000016.1	Pseudanabaena biceps PCC 7429	Bacteria	Cyanobacteria		Synechococcales	Pseudanabaenaceae	Pseudanabaena	H								
6067->6420	+	WP_009625642.1	NZ_ALWB01000016.1	Pseudanabaena biceps PCC 7429	Bacteria	Cyanobacteria		Synechococcales	Pseudanabaenaceae	Pseudanabaena	H								
6435->8156	+	WP_009625643.1	NZ_ALWB01000016.1	Pseudanabaena biceps PCC 7429	Bacteria	Cyanobacteria		Synechococcales	Pseudanabaenaceae	Pseudanabaena	type II secretion system protein E								
8355->9176	+	WP_009625644.1	NZ_ALWB01000016.1	Pseudanabaena biceps PCC 7429	Bacteria	Cyanobacteria		Synechococcales	Pseudanabaenaceae	Pseudanabaena	H	**Csx1**	pfam09670	CAS-III					
9719->9300	-	WP_009625644.1	NZ_ALWB01000016.1	Pseudanabaena biceps PCC 7429	Bacteria	Cyanobacteria		Synechococcales	Pseudanabaenaceae	Pseudanabaena	PilT protein								
9719->9300	-	WP_009625645.1	NZ_ALWB01000016.1	Pseudanabaena biceps PCC 7429	Bacteria	Cyanobacteria		Synechococcales	Pseudanabaenaceae	Pseudanabaena	PilT protein								
9945->9706	-	WP_009625646.1	NZ_ALWB01000016.1	Pseudanabaena biceps PCC 7429	Bacteria	Cyanobacteria		Synechococcales	Pseudanabaenaceae	Pseudanabaena	H								
10467->10078	-		NZ_ALWB01000016.1	Pseudanabaena biceps PCC 7429	Bacteria	Cyanobacteria		Synechococcales	Pseudanabaenaceae	Pseudanabaena	four helix bundle protein								
10622->11630	+	WP_009625647.1	NZ_ALWB01000016.1	Pseudanabaena biceps PCC 7429	Bacteria	Cyanobacteria		Synechococcales	Pseudanabaenaceae	Pseudanabaena		CRISPR Array			13 (35-42)	14 (35)	NA/NA/NA	GTTTCACACCCATTACCCCGCAAGGGGACGGAAAC	H
12150->11875	-	WP_009625648.1	NZ_ALWB01000016.1	Pseudanabaena biceps PCC 7429	Bacteria	Cyanobacteria		Synechococcales	Pseudanabaenaceae	Pseudanabaena	CRISPR-associated endonuclease Cas2	**Cas2**	mkCas0081	CAS-I, CAS-II, CAS-III					
14244->12151	-	WP_009625650.1	NZ_ALWB01000016.1	Pseudanabaena biceps PCC 7429	Bacteria	Cyanobacteria		Synechococcales	Pseudanabaenaceae	Pseudanabaena	CRISPR-associated endonuclease Cas1	**RT-Cas1**	pfam00078, pfam01867	CAS-III; CAS-I, CAS-II, CAS-III					
14779->14411	-		NZ_ALWB01000016.1	Pseudanabaena biceps PCC 7429	Bacteria	Cyanobacteria		Synechococcales	Pseudanabaenaceae	Pseudanabaena	four helix bundle protein								
17148->14934	-	WP_009625651.1	NZ_ALWB01000016.1	Pseudanabaena biceps PCC 7429	Bacteria	Cyanobacteria		Synechococcales	Pseudanabaenaceae	Pseudanabaena		CRISPR Array			29 (36-44)	30 (35)	NA/NA/NA	GTTTCACGCCTAACACCCCGCAAGGGGACGGAAAC	NA
18046->17420	-	WP_009625652.1	NZ_ALWB01000016.1	Pseudanabaena biceps PCC 7429	Bacteria	Cyanobacteria		Synechococcales	Pseudanabaenaceae	Pseudanabaena	H	**PD-DExK**	mkCas0061	CAS-I, CAS-III					
19327->18077	-		NZ_ALWB01000016.1	Pseudanabaena biceps PCC 7429	Bacteria	Cyanobacteria		Synechococcales	Pseudanabaenaceae	Pseudanabaena	CRISPR-associated protein, Csx2 family	**Csx1**	pfam09455	CAS-III					
**CAS-III-A**																			
2471428->2473290	+	WP_006515493.1	NZ_JH993797.1	Leptolyngbya sp. PCC 7375	Bacteria	Cyanobacteria		Synechococcales	Leptolyngbyaceae	Leptolyngbya	CRISPR-associated endonuclease Cas1	**RT-Cas1**	pfam00078, COG1518	CAS-III; CAS-I, CAS-II, CAS-III					
2473290->2473568	+	WP_006515494.1	NZ_JH993797.1	Leptolyngbya sp. PCC 7375	Bacteria	Cyanobacteria		Synechococcales	Leptolyngbyaceae	Leptolyngbya	CRISPR-associated endonuclease Cas2	**Cas2**	pfam09827	CAS-I, CAS-II, CAS-III					
2473926->2474482	+		NZ_JH993797.1	Leptolyngbya sp. PCC 7375	Bacteria	Cyanobacteria		Synechococcales	Leptolyngbyaceae	Leptolyngbya		CRISPR Array			7 (37-43)	8 (35)	NA/NA/NA	CTTTCATTCCAATTACCCCGCAAGGGGACGGAAAC	NA
2475073->2474687	-	WP_052588393.1	NZ_JH993797.1	Leptolyngbya sp. PCC 7375	Bacteria	Cyanobacteria		Synechococcales	Leptolyngbyaceae	Leptolyngbya	IS110 family transposase								
2476526->2475150	-	WP_006515495.1	NZ_JH993797.1	Leptolyngbya sp. PCC 7375	Bacteria	Cyanobacteria		Synechococcales	Leptolyngbyaceae	Leptolyngbya	CRISPR-associated protein	**WYL**	mkCas0075	CAS-I, CAS-III					
2476728->2477087	+	WP_006515496.1	NZ_JH993797.1	Leptolyngbya sp. PCC 7375	Bacteria	Cyanobacteria		Synechococcales	Leptolyngbyaceae	Leptolyngbya	CRISPR-associated protein, Csx3 family	**Csx3**	cd09740	CAS-I, CAS-III					
2478117->2480423	+	WP_006515498.1	NZ_JH993797.1	Leptolyngbya sp. PCC 7375	Bacteria	Cyanobacteria		Synechococcales	Leptolyngbyaceae	Leptolyngbya	type III-A CRISPR-associated protein Cas10/Csm1	**Cas10**	cd09680	CAS-III					
2480488->2480910	+	WP_052588702.1	NZ_JH993797.1	Leptolyngbya sp. PCC 7375	Bacteria	Cyanobacteria		Synechococcales	Leptolyngbyaceae	Leptolyngbya	type III-A CRISPR-associated protein Csm2	**Csm2gr11**	pfam03750	CAS-III-A					
2480913->2481884	+	WP_006515500.1	NZ_JH993797.1	Leptolyngbya sp. PCC 7375	Bacteria	Cyanobacteria		Synechococcales	Leptolyngbyaceae	Leptolyngbya	type III-A CRISPR-associated RAMP protein Csm3	**Csm3gr7**	cd09684	CAS-III-A, CAS-III-D					
2481887->2482999	+	WP_006515501.1	NZ_JH993797.1	Leptolyngbya sp. PCC 7375	Bacteria	Cyanobacteria		Synechococcales	Leptolyngbyaceae	Leptolyngbya	type III-A CRISPR-associated RAMP protein Csm4	**Csm4gr5**	cd09663	CAS-III-A					
2483058->2484326	+	WP_006515502.1	NZ_JH993797.1	Leptolyngbya sp. PCC 7375	Bacteria	Cyanobacteria		Synechococcales	Leptolyngbyaceae	Leptolyngbya	type III-A CRISPR-associated RAMP protein Csm5	**Csm5gr7**	cd09662	CAS-III-A					
2484329->2485093	+	WP_006515503.1	NZ_JH993797.1	Leptolyngbya sp. PCC 7375	Bacteria	Cyanobacteria		Synechococcales	Leptolyngbyaceae	Leptolyngbya	DUF2276 domain-containing protein	**Cas6**	pfam10040	CAS-III, CAS-IV					
2485398->2486261	+	WP_006515505.1	NZ_JH993797.1	Leptolyngbya sp. PCC 7375	Bacteria	Cyanobacteria		Synechococcales	Leptolyngbyaceae	Leptolyngbya	CRISPR-associated protein, Cas02710 family	**Csx1**	pfam09670	CAS-III					
2486549->2488028	+		NZ_JH993797.1	Leptolyngbya sp. PCC 7375	Bacteria	Cyanobacteria		Synechococcales	Leptolyngbyaceae	Leptolyngbya		CRISPR Array			19 (34-42)	20 (37)	NA/NA/E	GTCACAACCAACCACTTCCCCTCACGGGGATTGAAAC	H
**CAS-III-D**																			
447->181	-		NZ_BAWS01000034.1	Synechococcus sp. NKBG042902	Bacteria	Cyanobacteria		Synechococcales	Synechococcaceae	Synechococcus	repeat region	CRISPR Array			3 (41-43)	4 (35)	NA/NA/E	CTTTCCAACCCAACTCCCCGCAAGGGGACGGAAAC	H
708->983	+	WP_030008159.1	NZ_BAWS01000034.1	Synechococcus sp. NKBG042902	Bacteria	Cyanobacteria		Synechococcales	Synechococcaceae	Synechococcus	CRISPR-associated endonuclease Cas2	**Cas2**	pfam09827	CAS-I, CAS-II, CAS-III					
983->3094	+	WP_030008160.1	NZ_BAWS01000034.1	Synechococcus sp. NKBG042902	Bacteria	Cyanobacteria		Synechococcales	Synechococcaceae	Synechococcus	CRISPR-associated endonuclease Cas1	**RT-Cas1**	pfam00078, pfam01867	CAS-III; CAS-I, CAS-II, CAS-III					
3228->3677	+	WP_030008161.1	NZ_BAWS01000034.1	Synechococcus sp. NKBG042902	Bacteria	Cyanobacteria		Synechococcales	Synechococcaceae	Synechococcus	H								
4100->5830	+	WP_030008162.1	NZ_BAWS01000034.1	Synechococcus sp. NKBG042902	Bacteria	Cyanobacteria		Synechococcales	Synechococcaceae	Synechococcus	H	**Cas10**	mkCas0133	CAS-III					
5875->8258	+		NZ_BAWS01000034.1	Synechococcus sp. NKBG042902	Bacteria	Cyanobacteria		Synechococcales	Synechococcaceae	Synechococcus	H	**Csx10gr5**	cd09700	CAS-III-D					
8261->9868	+	WP_030008163.1	NZ_BAWS01000034.1	Synechococcus sp. NKBG042902	Bacteria	Cyanobacteria		Synechococcales	Synechococcaceae	Synechococcus	H	**Csm3gr7**	cd09726	CAS-III-D					
9865->10458	+	WP_045442558.1	NZ_BAWS01000034.1	Synechococcus sp. NKBG042902	Bacteria	Cyanobacteria		Synechococcales	Synechococcaceae	Synechococcus	CRISPR-associated protein	**Csx19**	cls001376	CAS-III-D					
10579->13042	+		NZ_BAWS01000034.1	Synechococcus sp. NKBG042902	Bacteria	Cyanobacteria		Synechococcales	Synechococcaceae	Synechococcus	CRISPR-associated RAMP family protein	**Csm3gr7**	cls000253	CAS-III-A, CAS-III-D					
13141->14481	+	WP_030008166.1	NZ_BAWS01000034.1	Synechococcus sp. NKBG042902	Bacteria	Cyanobacteria		Synechococcales	Synechococcaceae	Synechococcus	CRISPR-associated protein	**Csx1**	cd09668	CAS-III					
**Partial**																			
250036->251763	+	BAU44853.1	AP017367.1	Leptolyngbya sp. O-77	Bacteria	Cyanobacteria		Synechococcales	Leptolyngbyaceae	Leptolyngbya	cas1 1	**RT-Cas1**	pfam00078, COG1518	CAS-III; CAS-I, CAS-II, CAS-III					
251776->252051	+	BAU44852.1	AP017367.1	Leptolyngbya sp. O-77	Bacteria	Cyanobacteria		Synechococcales	Leptolyngbyaceae	Leptolyngbya	cas2 2	**Cas2**	mkCas0081	CAS-I, CAS-II, CAS-III					
252379->252936	+		AP017367.1	Leptolyngbya sp. O-77	Bacteria	Cyanobacteria		Synechococcales	Leptolyngbyaceae	Leptolyngbya	repeat region	CRISPR Array			7 (37-42)	8 (35)	NA/NA/E	CTTTCACCACCACTTCCCCGCAAGGGGACTGAAAC	NA
253441->253818	+	BAU44851.1	AP017367.1	Leptolyngbya sp. O-77	Bacteria	Cyanobacteria		Synechococcales	Leptolyngbyaceae	Leptolyngbya	H								
254272->253895	-	BAU44850.1	AP017367.1	Leptolyngbya sp. O-77	Bacteria	Cyanobacteria		Synechococcales	Leptolyngbyaceae	Leptolyngbya	H								
254647->255777	+	BAU44849.1	AP017367.1	Leptolyngbya sp. O-77	Bacteria	Cyanobacteria		Synechococcales	Leptolyngbyaceae	Leptolyngbya	H	**Cas6**	pfam10040	CAS-III, CAS-IV					
**CAS-III-B**																			
369323->365777	-		NZ_JH976538.1	Nodosilinea nodulosa PCC 7104	Bacteria	Cyanobacteria		Synechococcales	Leptolyngbyaceae	Nodosilinea	repeat region	CRISPR Array			45 (30-60)	46 (36)	NA/NA/C	CCCTACCGAATGGGTTAATTCGGAATAGTTGGAAAC	H
369820->369545	-	WP_017302243.1	NZ_JH976538.1	Nodosilinea nodulosa PCC 7104	Bacteria	Cyanobacteria		Synechococcales	Leptolyngbyaceae	Nodosilinea	CRISPR-associated endonuclease Cas2	**Cas2**	pfam09827	CAS-I, CAS-II, CAS-III					
371925->369820	-	WP_017302244.1	NZ_JH976538.1	Nodosilinea nodulosa PCC 7104	Bacteria	Cyanobacteria		Synechococcales	Leptolyngbyaceae	Nodosilinea	CRISPR-associated endonuclease Cas1	**RT-Cas1**	pfam00078, COG1518	CAS-III; CAS-I, CAS-II, CAS-III					
372902->372063	-	WP_017302245.1	NZ_JH976538.1	Nodosilinea nodulosa PCC 7104	Bacteria	Cyanobacteria		Synechococcales	Leptolyngbyaceae	Nodosilinea	H	**Csx1**	cd09702	CAS-III					
373225->375042	+	WP_040697961.1	NZ_JH976538.1	Nodosilinea nodulosa PCC 7104	Bacteria	Cyanobacteria		Synechococcales	Leptolyngbyaceae	Nodosilinea	H	**Cas10**	pfam12469, cd09679	CAS-III-B, CAS-III					
375045->376274	+	WP_017302247.1	NZ_JH976538.1	Nodosilinea nodulosa PCC 7104	Bacteria	Cyanobacteria		Synechococcales	Leptolyngbyaceae	Nodosilinea	H	**Cmr3gr5**	pfam09700	CAS-III-B					
376320->377369	+	WP_017302248.1	NZ_JH976538.1	Nodosilinea nodulosa PCC 7104	Bacteria	Cyanobacteria		Synechococcales	Leptolyngbyaceae	Nodosilinea	H	**Cmr4gr7**	COG1336	CAS-III-C					
377388->377825	+	WP_017302249.1	NZ_JH976538.1	Nodosilinea nodulosa PCC 7104	Bacteria	Cyanobacteria		Synechococcales	Leptolyngbyaceae	Nodosilinea	H	**Cmr5gr11**	mkCas0065	CAS-III-B					
378065->379738	-	WP_071527438.1	NZ_JH976538.1	Nodosilinea nodulosa PCC 7104	Bacteria	Cyanobacteria		Synechococcales	Leptolyngbyaceae	Nodosilinea	H	**Cmr6gr7**	COG1604	CAS-III-B					

RT-CRISPR-6 (CAS-III-D)
Gammaproteobacteria (Vibrionales, Alteromonadales)

[a](Sub) Type/[b]Coordinates	Strand	Protein_id	Genome Partition ID	Strain	Domain	Phylum	Lineage (class)	Order	Family	Genus	[c]Cas gene (annotated)	[d]Cas gene/[e]Array (identified)	[d]Profiles	[d](Sub)Type-specificity	[e]Spacers (length)	[e]Repeats (length)	[f]Str.motif/Seq.family/Superclass	[f]Seq Repeats	[g]Orientation Confidence
**CAS-III-D**																			
1681792->1680902	-	WP_011152741.1	NC_005140.1	Vibrio vulnificus YJ016	Bacteria	Proteobacteria	Gammaproteobacteria	Vibrionales	Vibrionaceae	Vibrio	H	**Cas6**	mkCas0066	CAS-I, CAS-III					
1683951->1681789	-	WP_043877603.1	NC_005140.1	Vibrio vulnificus YJ016	Bacteria	Proteobacteria	Gammaproteobacteria	Vibrionales	Vibrionaceae	Vibrio	CRISPR-associated RAMP family protein	**Csm3gr7**	cls000253	CAS-III-A, CAS-III-D					
1684481->1683966	-	WP_011152743.1	NC_005140.1	Vibrio vulnificus YJ016	Bacteria	Proteobacteria	Gammaproteobacteria	Vibrionales	Vibrionaceae	Vibrio	H								
1685905->1684478	-	WP_011152744.1	NC_005140.1	Vibrio vulnificus YJ016	Bacteria	Proteobacteria	Gammaproteobacteria	Vibrionales	Vibrionaceae	Vibrio	H	**Csm3gr7**	cd09683	CAS-III-A, CAS-III-D					
1687479->1685902	-	WP_011152745.1	NC_005140.1	Vibrio vulnificus YJ016	Bacteria	Proteobacteria	Gammaproteobacteria	Vibrionales	Vibrionaceae	Vibrio	H	**Csx10gr5**	cls000748	CAS-III-D					
1688078->1687476	-	WP_011152746.1	NC_005140.1	Vibrio vulnificus YJ016	Bacteria	Proteobacteria	Gammaproteobacteria	Vibrionales	Vibrionaceae	Vibrio	H	**Csm3gr7**	COG1337	CAS-III-D					
1689850->1688075	-	WP_043877604.1	NC_005140.1	Vibrio vulnificus YJ016	Bacteria	Proteobacteria	Gammaproteobacteria	Vibrionales	Vibrionaceae	Vibrio	H	**Cas10**	cls000686	CAS-III					
1690471->1691262	+	WP_011152748.1	NC_005140.1	Vibrio vulnificus YJ016	Bacteria	Proteobacteria	Gammaproteobacteria	Vibrionales	Vibrionaceae	Vibrio	H								
1691478->1691647	+		NC_005140.1	Vibrio vulnificus YJ016	Bacteria	Proteobacteria	Gammaproteobacteria	Vibrionales	Vibrionaceae	Vibrio		CRISPR Array			3 (34,37)	3 (35)	NA/NA/E	GTTTCAGACATGCCCGGTTTAGACGGGATTAAGAC	H
1691704->1693776	+	WP_011152750.1	NC_005140.1	Vibrio vulnificus YJ016	Bacteria	Proteobacteria	Gammaproteobacteria	Vibrionales	Vibrionaceae	Vibrio	CRISPR-associated endonuclease Cas1	**RT-Cas1**	pfam00078, cd09634	CAS-III; CAS-I, CAS-II, CAS-III, CAS-V					
1693776->1694060	+	WP_043877605.1	NC_005140.1	Vibrio vulnificus YJ016	Bacteria	Proteobacteria	Gammaproteobacteria	Vibrionales	Vibrionaceae	Vibrio	CRISPR-associated endonuclease Cas2	**Cas2**	mkCas0081	CAS-I, CAS-II, CAS-III					
1694069->1694347	+	WP_011152752.1	NC_005140.1	Vibrio vulnificus YJ016	Bacteria	Proteobacteria	Gammaproteobacteria	Vibrionales	Vibrionaceae	Vibrio	CRISPR-associated endonuclease Cas2	**Cas2**	cd09725	CAS-I, CAS-II, CAS-III					
1694586->1695317	+		NC_005140.1	Vibrio vulnificus YJ016	Bacteria	Proteobacteria	Gammaproteobacteria	Vibrionales	Vibrionaceae	Vibrio		CRISPR Array			9 (33-37)	10 (35)	NA/NA/E	GTTTCAGACATGCCCGGTTTAGACGGGATTAAGAC	L
1696662->1695361	-	WP_052281496.1	NC_005140.1	Vibrio vulnificus YJ016	Bacteria	Proteobacteria	Gammaproteobacteria	Vibrionales	Vibrionaceae	Vibrio	CRISPR-associated protein	**Csx1**	cd09686	CAS-III					
1696970->1697260	+	WP_043877606.1	NC_005140.1	Vibrio vulnificus YJ016	Bacteria	Proteobacteria	Gammaproteobacteria	Vibrionales	Vibrionaceae	Vibrio	putative CRISPR-associated protein	**Csx16**	pfam09652	CAS-I, CAS-III					
1697291->1698520	+	WP_043877607.1	NC_005140.1	Vibrio vulnificus YJ016	Bacteria	Proteobacteria	Gammaproteobacteria	Vibrionales	Vibrionaceae	Vibrio	putative CRISPR-associated protein	**Csx16**	pfam09652	CAS-I, CAS-III					
1698533->1699708	+	WP_011152756.1	NC_005140.1	Vibrio vulnificus YJ016	Bacteria	Proteobacteria	Gammaproteobacteria	Vibrionales	Vibrionaceae	Vibrio	CRISPR-associated protein	**Csx1**	cd09668	CAS-III					
**CAS-III-D**																			
6571->8313	+	WP_038884968.1	NZ_BAOI01000066.1	Vibrio rotiferianus CAIM 577	Bacteria	Proteobacteria	Gammaproteobacteria	Vibrionales	Vibrionaceae	Vibrio	H	**Cas10**	cls000686	CAS-III					
8304->8921	+	WP_038884968.1	NZ_BAOI01000066.1	Vibrio rotiferianus CAIM 577	Bacteria	Proteobacteria	Gammaproteobacteria	Vibrionales	Vibrionaceae	Vibrio	H	**Csm3gr7**	cd09726	CAS-III-D					
8923->10494	+	WP_038884969.1	NZ_BAOI01000066.1	Vibrio rotiferianus CAIM 577	Bacteria	Proteobacteria	Gammaproteobacteria	Vibrionales	Vibrionaceae	Vibrio	H	**Csx10gr5**	cls000748	CAS-III-D					
10491->11882	+	WP_038884970.1	NZ_BAOI01000066.1	Vibrio rotiferianus CAIM 577	Bacteria	Proteobacteria	Gammaproteobacteria	Vibrionales	Vibrionaceae	Vibrio	H	**Csm3gr7**, Csm4gr5	pfam03787, mkCas0089	CAS-III-A, CAS-III-D					
11879->12355	+	WP_038884971.1	NZ_BAOI01000066.1	Vibrio rotiferianus CAIM 577	Bacteria	Proteobacteria	Gammaproteobacteria	Vibrionales	Vibrionaceae	Vibrio	H								
12358->14499	+	WP_038884972.1	NZ_BAOI01000066.1	Vibrio rotiferianus CAIM 577	Bacteria	Proteobacteria	Gammaproteobacteria	Vibrionales	Vibrionaceae	Vibrio	CRISPR-associated RAMP family protein	**Csm3gr7**	cls000253	CAS-III-A, CAS-III-D					
14496->15362	+	WP_038884973.1	NZ_BAOI01000066.1	Vibrio rotiferianus CAIM 577	Bacteria	Proteobacteria	Gammaproteobacteria	Vibrionales	Vibrionaceae	Vibrio	H	**Cas6**	mkCas0066	CAS-I, CAS-III					
15356->16486	+	WP_038884974.1	NZ_BAOI01000066.1	Vibrio rotiferianus CAIM 577	Bacteria	Proteobacteria	Gammaproteobacteria	Vibrionales	Vibrionaceae	Vibrio	H	**Csx1**	pfam09002	CAS-III					
17477->16530	-	WP_038884977.1	NZ_BAOI01000066.1	Vibrio rotiferianus CAIM 577	Bacteria	Proteobacteria	Gammaproteobacteria	Vibrionales	Vibrionaceae	Vibrio	H								
18756->17449	-	WP_050568962.1	NZ_BAOI01000066.1	Vibrio rotiferianus CAIM 577	Bacteria	Proteobacteria	Gammaproteobacteria	Vibrionales	Vibrionaceae	Vibrio	CRISPR-associated protein	**Csx1**	cd09686	CAS-III					
19006->20253	+	WP_038884980.1	NZ_BAOI01000066.1	Vibrio rotiferianus CAIM 577	Bacteria	Proteobacteria	Gammaproteobacteria	Vibrionales	Vibrionaceae	Vibrio	putative CRISPR-associated protein	**Csx16**	pfam09652	CAS-I, CAS-III					
20371->20637	+	WP_050568963.1	NZ_BAOI01000066.1	Vibrio rotiferianus CAIM 577	Bacteria	Proteobacteria	Gammaproteobacteria	Vibrionales	Vibrionaceae	Vibrio	H								

20728->21531	+	WP_050568964.1	NZ_BAOI01000066.1	Vibrio rotiferianus CAIM 577	Bacteria	Proteobacteria	Gammaproteobacteria	Vibrionales	Vibrionaceae	Vibrio	H								
21581->23653	+	WP_038884984.1	NZ_BAOI01000066.1	Vibrio rotiferianus CAIM 577	Bacteria	Proteobacteria	Gammaproteobacteria	Vibrionales	Vibrionaceae	Vibrio	CRISPR-associated endonuclease Cas1	**RT-Cas1**	pfam00078, pfam01867	CAS-III;CAS-I, CAS-II, CAS-III					
23653->23937	+	WP_038884986.1	NZ_BAOI01000066.1	Vibrio rotiferianus CAIM 577	Bacteria	Proteobacteria	Gammaproteobacteria	Vibrionales	Vibrionaceae	Vibrio	CRISPR-associated endonuclease Cas2	**Cas2**	mkCas0081	CAS-I, CAS-II, CAS-III					
23946->24224	+	WP_038884988.1	NZ_BAOI01000066.1	Vibrio rotiferianus CAIM 577	Bacteria	Proteobacteria	Gammaproteobacteria	Vibrionales	Vibrionaceae	Vibrio	CRISPR-associated endonuclease Cas2	**Cas2**	mkCas0081	CAS-I, CAS-II, CAS-III					
**Unknown**																			
834->2885	+	WP_046007427.1	NZ_JXYA01000100.1	Pseudoalteromonas rubra strain S2471	Bacteria	Proteobacteria	Gammaproteobacteria	Alteromonadales	Pseudoalteromonadace	Pseudoalteromonas	CRISPR-associated endonuclease Cas1	**RT-Cas1**	pfam00078, cd09634	CAS-III; CAS-I, CAS-II, CAS-III, CAS-V					
3165->3437	+	WP_046007428.1	NZ_JXYA01000100.1	Pseudoalteromonas rubra strain S2471	Bacteria	Proteobacteria	Gammaproteobacteria	Alteromonadales	Pseudoalteromonadace	Pseudoalteromonas	CRISPR-associated endonuclease Cas2	**Cas2**	COG1343	CAS-I, CAS-II, CAS-III					
3843->4291	+		NZ_JXYA01000100.1	Pseudoalteromonas rubra strain S2471	Bacteria	Proteobacteria	Gammaproteobacteria	Alteromonadales	Pseudoalteromonadace	Pseudoalteromonas	repeat region	CRISPR Array			6 (32-36)	7 (35)	NA/NA/F	GTACCGCAAGCCCCGACTTTAGAGGGGATTGAGAC	M
4408->4806	+	WP_046007429.1	NZ_JXYA01000100.1	Pseudoalteromonas rubra strain S2471	Bacteria	Proteobacteria	Gammaproteobacteria	Alteromonadales	Pseudoalteromonadace	Pseudoalteromonas	H								
5967->5425	-	WP_046007430.1	NZ_JXYA01000100.1	Pseudoalteromonas rubra strain S2471	Bacteria	Proteobacteria	Gammaproteobacteria	Alteromonadales	Pseudoalteromonadace	Pseudoalteromonas	H								
6195->5971	-	WP_052713340.1	NZ_JXYA01000100.1	Pseudoalteromonas rubra strain S2471	Bacteria	Proteobacteria	Gammaproteobacteria	Alteromonadales	Pseudoalteromonadace	Pseudoalteromonas	transcriptional regulator								
6735->7390	+		NZ_JXYA01000100.1	Pseudoalteromonas rubra strain S2471	Bacteria	Proteobacteria	Gammaproteobacteria	Alteromonadales	Pseudoalteromonadace	Pseudoalteromonas	Repeat region	CRISPR Array			9 (33-35)	10 (35)	NA/NA/F	GTACCGGAAGCCCCGACTTTAGAGGGGATTGAGAC	NA

RT-CRISPR-7 (CAS-III-D, CAS-III-B/C)
Alphaproteobacteria (Rhodospirillales, Rhodobacterales, Caulobacterales); Deltaproteobacteria (Desulfarcucales)

[a](Sub) Type/[b]Coordinates	Strand	Protein_id	Genome Partition ID	Strain	Domain	Phylum	Lineage (class)	Order	Family	Genus	[c]Cas gene (annotated)	[d]Cas gene/[e]Array (identified)	[d]Profiles	[d](Sub)Type-specificity	[e]Spacers (length)	[e]Repeats (length)	[f]Str.motif/Seq.family/Superclass	[f]Seq Repeats	[g]Orientation Confidence
**Unknown**																			
15051 ->13219	-	WP_062763150.1	NZ_LPZR01000092.1	Tistrella mobilis MCCC 1A02139	Bacteria	Proteobacteria	Alphaproteobacteria	Rhodospirillales	Rhodospirillaceae	Tistrella	CRISPR-associated endonuclease Cas1	RT-**Cas1** (RT DV mutated)	pfam00078, cd09634	CAS-I, CAS-II, CAS-III, CAS-V					
**CAS-III-D**																			
403679->405373	+	AFK55767.1	CP003237.2	Tistrella mobilis KA081020-065	Bacteria	Proteobacteria	Alphaproteobacteria	Rhodospirillales	Rhodospirillaceae	Tistrella	H	**Cas10**	cls000707	CAS-III					
405379->407313	+	AFK55768.1	CP003237.2	Tistrella mobilis KA081020-065	Bacteria	Proteobacteria	Alphaproteobacteria	Rhodospirillales	Rhodospirillaceae	Tistrella	H	**Csm4gr5**	cls000259	CAS-III-D					
407310->408947	+	AFK55769.1	CP003237.2	Tistrella mobilis KA081020-065	Bacteria	Proteobacteria	Alphaproteobacteria	Rhodospirillales	Rhodospirillaceae	Tistrella	H	**Csm3gr7**	COG1337, cd09726	CAS-III-D; CAS-III-D					
408944->411208	+	AFK55770.1	CP003237.2	Tistrella mobilis KA081020-065	Bacteria	Proteobacteria	Alphaproteobacteria	Rhodospirillales	Rhodospirillaceae	Tistrella	Cold-shock DNA-binding domain protein	**Csm3gr7**	cls000253	CAS-III-A, CAS-III-D					
411205->412182	+	AFK55771.1	CP003237.2	Tistrella mobilis KA081020-065	Bacteria	Proteobacteria	Alphaproteobacteria	Rhodospirillales	Rhodospirillaceae	Tistrella	H	**Cas6**	cls000976	CAS-III					
412551 ->412244	-		CP003237.2	Tistrella mobilis KA081020-065	Bacteria	Proteobacteria	Alphaproteobacteria	Rhodospirillales	Rhodospirillaceae	Tistrella		CRISPR Array			4 (29-32)	5 (37)	NA/NA/NA	GTTTGAAGCCATGACCAATGGCTCCGGTCATGGAAAC	M
416011->412664	-	AFK55772.1	CP003237.2	Tistrella mobilis KA081020-065	Bacteria	Proteobacteria	Alphaproteobacteria	Rhodospirillales	Rhodospirillaceae	Tistrella	H								
416721-> 416140	-		CP003237.2	Tistrella mobilis KA081020-065	Bacteria	Proteobacteria	Alphaproteobacteria	Rhodospirillales	Rhodospirillaceae	Tistrella		CRISPR Array			8 (29-34)	9 (37)	NA/NA/NA	GTTTGAAGCCATGACCAATCGCTCCGGTCATGGAAAC	M
416875->418866	+	AFK55773.1	CP003237.2	Tistrella mobilis KA081020-065	Bacteria	Proteobacteria	Alphaproteobacteria	Rhodospirillales	Rhodospirillaceae	Tistrella	aI2	**RT-Cas1**	pfam00078, COG1518	CAS-III; CAS-I, CAS-II, CAS-III					
418867->419184	+	AFK55774.1	CP003237.2	Tistrella mobilis KA081020-065	Bacteria	Proteobacteria	Alphaproteobacteria	Rhodospirillales	Rhodospirillaceae	Tistrella	CRISPR-associated protein	**Cas2**	cd09725	CAS-I,CAS-II,CAS-III,CAS-V					
**Partial CAS-III-D**																			
2379086->2379994	+	ADE85033.1	CP001312.1	Rhodobacter capsulatus SB 1003	Bacteria	Proteobacteria	Alphaproteobacteria	Rhodobacterales	Rhodobacteraceae	Rhodobacter	conserved domain protein	**Cas6**	cls000976	CAS-III					
2380311-> 2379988	-		CP001312.1	Rhodobacter capsulatus SB 1003	Bacteria	Proteobacteria	Alphaproteobacteria	Rhodobacterales	Rhodobacteraceae	Rhodobacter		CRISPR Array			4 (34,37)	5 (37)	NA/NA/E	GCCCAGACACCTCTCCCGCCAGTAAGCGGATTGAGAC	M
2380455->2380985	+	ADE85032.1	CP001312.1	Rhodobacter capsulatus SB 1003	Bacteria	Proteobacteria	Alphaproteobacteria	Rhodobacterales	Rhodobacteraceae	Rhodobacter	RNA-directed DNA polymerase								
2380832->2382433	+	ADE85031.1	CP001312.1	Rhodobacter capsulatus SB 1003	Bacteria	Proteobacteria	Alphaproteobacteria	Rhodobacterales	Rhodobacteraceae	Rhodobacter	CRISPR-associated protein, reverse transcriptasefamily/Cas1 family	**RT-Cas1**	pfam00078, cd09634	CAS-III; CAS-I, CAS-II, CAS-III, CAS-V					
2382659 -> 2382484	-		CP001312.1	Rhodobacter capsulatus SB 1003	Bacteria	Proteobacteria	Alphaproteobacteria	Rhodobacterales	Rhodobacteraceae	Rhodobacter		CRISPR Array			2 (32,33)	3 (37)	NA/NA/E	GCCCAGACACCTCTCCCGCCAGTAAGCGGATTGAGAC	NA
2383007->2383297	+	ADE85030.1	CP001312.1	Rhodobacter capsulatus SB 1003	Bacteria	Proteobacteria	Alphaproteobacteria	Rhodobacterales	Rhodobacteraceae	Rhodobacter	CRISPR-associated protein, Cas2 family	**Cas2**	pfam09827	CAS-I, CAS-II, CAS-III					
2385921->2383294	-	ADE85029.1	CP001312.1	Rhodobacter capsulatus SB 1003	Bacteria	Proteobacteria	Alphaproteobacteria	Rhodobacterales	Rhodobacteraceae	Rhodobacter	CRISPR-associated protein, RAMP family	**Csm3gr7**	cd09726, cls000253	CAS-III-D; CAS-III-A, CAS-III-D					
2387564->2385918	-	ADE85028.1	CP001312.1	Rhodobacter capsulatus SB 1003	Bacteria	Proteobacteria	Alphaproteobacteria	Rhodobacterales	Rhodobacteraceae	Rhodobacter	conserved H	**Csm3gr7**	cls000708	CAS-III-D					
2387561-> 2389027	-	ADE85027.1	CP001312.1	Rhodobacter capsulatus SB 1003	Bacteria	Proteobacteria	Alphaproteobacteria	Rhodobacterales	Rhodobacteraceae	Rhodobacter	conserved H	**Cas10**	cls000707	CAS-III					
2389399-> 2389157	-		CP001312.1	Rhodobacter capsulatus SB 1003	Bacteria	Proteobacteria	Alphaproteobacteria	Rhodobacterales	Rhodobacteraceae	Rhodobacter		CRISPR Array			3 (41,43)	4 (29)	M4/NA/E	GCCCAGATACCTCTCCCGCCAGTAAGCGGATTGAGAC	M
**Partial CAS-III-D**																			
3647->5749	+	EJW09347.1	AKZI01000198	Rhodovulum sp. PH10	Bacteria	Proteobacteria	Alphaproteobacteria	Rhodobacterales	Rhodobacteraceae	Rhodovulum	CRISPR-associated protein Cas1	**RT-Cas1**	pfam00078, COG1518	CAS-III; CAS-I, CAS-II, CAS-III					
5923->6210	+	EJW09346.1	AKZI01000198	Rhodovulum sp. PH10	Bacteria	Proteobacteria	Alphaproteobacteria	Rhodobacterales	Rhodobacteraceae	Rhodovulum	H	**Cas2**	mkCas0081	CAS-I, CAS-II, CAS-III					
6668 -> 6287	-		AKZI01000198	Rhodovulum sp. PH10	Bacteria	Proteobacteria	Alphaproteobacteria	Rhodobacterales	Rhodobacteraceae	Rhodovulum		CRISPR Array			5 (31-34)	6 (37)	M4/NA/F	GTTCAGTGATCCCTCCGCCTCGGATGCGGATTGAGAC	M
6985->7926	+	EJW09345.1	AKZI01000198	Rhodovulum sp. PH10	Bacteria	Proteobacteria	Alphaproteobacteria	Rhodobacterales	Rhodobacteraceae	Rhodovulum	H	**Cas6**	cls000976	CAS-III					
8041->9852	+	EJW09344.1	AKZI01000198	Rhodovulum sp. PH10	Bacteria	Proteobacteria	Alphaproteobacteria	Rhodobacterales	Rhodobacteraceae	Rhodovulum	H	**Cas10**	cls000707	CAS-III					
9849->11687	+	EJW09343.1	AKZI01000198	Rhodovulum sp. PH10	Bacteria	Proteobacteria	Alphaproteobacteria	Rhodobacterales	Rhodobacteraceae	Rhodovulum	H	**Csm3gr7**	cls000708	CAS-III-D					
11684->13216	+	EJW09342.1	AKZI01000198	Rhodovulum sp. PH10	Bacteria	Proteobacteria	Alphaproteobacteria	Rhodobacterales	Rhodobacteraceae	Rhodovulum	Uncharacterized protein DUF324	**Csm3gr7**	cd09683, cd09683	CAS-III-A, CAS-III-D; CAS-III-A, CAS-III-D					
13213->15438	+	EJW09341.1	AKZI01000198	Rhodovulum sp. PH10	Bacteria	Proteobacteria	Alphaproteobacteria	Rhodobacterales	Rhodobacteraceae	Rhodovulum	H	**Csm3gr7**	cls000253	CAS-III-A, CAS-III-D					
**Partial CAS-III-B/CAS-III-C**																			
2733->4676	+	WP_019956891.1	NZ_KB908004.1	Loktanella vestfoldensis DSM 16212	Bacteria	Proteobacteria	Alphaproteobacteria	Rhodobacterales	Rhodobacteraceae	Loktanella	CRISPR-associated endonuclease Cas1	**RT-Cas1**	pfam00078, cd09634	CAS-III; CAS-I, CAS-II, CAS-III, CAS-V					
5278->5481	+	WP_019956892.1	NZ_KB908004.1	Loktanella vestfoldensis DSM 16212	Bacteria	Proteobacteria	Alphaproteobacteria	Rhodobacterales	Rhodobacteraceae	Loktanella	H								
6170->5478	-	WP_026352691.1	NZ_KB908004.1	Loktanella vestfoldensis DSM 16212	Bacteria	Proteobacteria	Alphaproteobacteria	Rhodobacterales	Rhodobacteraceae	Loktanella	type III-B CRISPR module RAMP protein Cmr6	**Cmr6gr7**	COG1604	CAS-III-B					
6685->6323	-	WP_019956894.1	NZ_KB908004.1	Loktanella vestfoldensis DSM 16212	Bacteria	Proteobacteria	Alphaproteobacteria	Rhodobacterales	Rhodobacteraceae	Loktanella	H								
7827->6844	-	WP_019956895.1	NZ_KB908004.1	Loktanella vestfoldensis DSM 16212	Bacteria	Proteobacteria	Alphaproteobacteria	Rhodobacterales	Rhodobacteraceae	Loktanella	type III-B CRISPR module RAMP protein Cmr4	**Cmr4gr7**	COG1336	CAS-III-C					
11067->9076	-	WP_019956896.1	NZ_KB908004.1	Loktanella vestfoldensis DSM 16212	Bacteria	Proteobacteria	Alphaproteobacteria	Rhodobacterales	Rhodobacteraceae	Loktanella	H	**Cas10**	cd09679	CAS-III					
12947->11259	-	WP_019956897.1	NZ_KB908004.1	Loktanella vestfoldensis DSM 16212	Bacteria	Proteobacteria	Alphaproteobacteria	Rhodobacterales	Rhodobacteraceae	Loktanella	H								
13545 -> 13180	-		NZ_KB908004.1	Loktanella vestfoldensis DSM 16212	Bacteria	Proteobacteria	Alphaproteobacteria	Rhodobacterales	Rhodobacteraceae	Loktanella		CRISPR Array			5 (44,45)	6 (24)	NA/NA/C	GTCTGCTGTGGTCGACAGAGGCTGCGGCCTATGAAAC	M
13794->14078	+	WP_019956898.1	NZ_KB908004.1	Loktanella vestfoldensis DSM 16212	Bacteria	Proteobacteria	Alphaproteobacteria	Rhodobacterales	Rhodobacteraceae	Loktanella	CRISPR-associated endonuclease Cas2	**Cas2**	cd09725	CAS-I, CAS-II, CAS-III, CAS-V					
14075->15019	+	WP_019956899.1	NZ_KB908004.1	Loktanella vestfoldensis DSM 16212	Bacteria	Proteobacteria	Alphaproteobacteria	Rhodobacterales	Rhodobacteraceae	Loktanella	H	**Cas6**	cls000976	CAS-III					
15071->16864	+	WP_019956900.1	NZ_KB908004.1	Loktanella vestfoldensis DSM 16212	Bacteria	Proteobacteria	Alphaproteobacteria	Rhodobacterales	Rhodobacteraceae	Loktanella	H	**Csx1**	cd09741	CAS-III					
**Partial CAS-III-D**																			
1710->37	-	EJW09479.1	AKZI01000170.1	Rhodovulum sp. PH10	Bacteria	Proteobacteria	Alphaproteobacteria	Rhodobacterales	Rhodobacteraceae	Rhodovulum	H	**Csm3gr7**	cls000670	CAS-III-D					
3394->1778	-	EJW09480.1	AKZI01000170.1	Rhodovulum sp. PH10	Bacteria	Proteobacteria	Alphaproteobacteria	Rhodobacterales	Rhodobacteraceae	Rhodovulum	H	**Cas10**	cls000707	CAS-III					
3780 ->3540	-		AKZI01000170.1	Rhodovulum sp. PH10	Bacteria	Proteobacteria	Alphaproteobacteria	Rhodobacterales	Rhodobacteraceae	Rhodovulum		CRISPR Array			3 (27,31)	4 (38)	M17/NA/E	GTCTGACTGCATGCACCGCGCACCAGCGGAACGTAAA	L
3805->5895	+	EJW09481.1	AKZI01000170.1	Rhodovulum sp. PH10	Bacteria	Proteobacteria	Alphaproteobacteria	Rhodobacterales	Rhodobacteraceae	Rhodovulum	CRISPR-associated protein Cas1	**RT-Cas1**	pfam00078, pfam01867	CAS-III; CAS-I, CAS-II, CAS-III, CAS-V					
5892->6185	+	EJW09482.1	AKZI01000170.1	Rhodovulum sp. PH10	Bacteria	Proteobacteria	Alphaproteobacteria	Rhodobacterales	Rhodobacteraceae	Rhodovulum	H	**Cas2**	cd09725	CAS-I, CAS-II, CAS-III, CAS-V					
6200->7051	+	EJW09483.1	AKZI01000170.1	Rhodovulum sp. PH10	Bacteria	Proteobacteria	Alphaproteobacteria	Rhodobacterales	Rhodobacteraceae	Rhodovulum	H	**Cas6**	cls000976	CAS-III					
**CAS-III-D**																			
2,045,872 -> 2,045,204	-	WP_041787431.1	NC_014664	Rhodomicrobium vannielii ATCC 17100	Bacteria	Proteobacteria	Alphaproteobacteria	Rhizobiales	Hyphomicrobiaceae	Rhodomicrobium	type I-U CRISPR-associated protein Cas5/Cas6	csb2gr5	cls00315						
2,046,846 -> 2,045,953	-	WP_049779315.1	NC_014664	Rhodomicrobium vannielii ATCC 17100	Bacteria	Proteobacteria	Alphaproteobacteria	Rhizobiales	Hyphomicrobiaceae	Rhodomicrobium	H	**RT**	pfam00078	CAS-III					
2,047,142 -> 2,046,849	-	WP_013419503.1	NC_014664	Rhodomicrobium vannielii ATCC 17100	Bacteria	Proteobacteria	Alphaproteobacteria	Rhizobiales	Hyphomicrobiaceae	Rhodomicrobium	CRISPR-associated protein Cas2	**Cas2**	COG1343	CAS-I, CAS-II, CAS-III					
2,048,239 -> 2,047,139	-	WP_013419504.1	NC_014664	Rhodomicrobium vannielii ATCC 17100	Bacteria	Proteobacteria	Alphaproteobacteria	Rhizobiales	Hyphomicrobiaceae	Rhodomicrobium	CRISPR-associated protein Cas1	**Cas1**	COG1518	CAS-I, CAS-II, CAS-III					
2,048,647 -> 2,049,436	+		NC_014664	Rhodomicrobium vannielii ATCC 17100	Bacteria	Proteobacteria	Alphaproteobacteria	Rhizobiales	Hyphomicrobiaceae	Rhodomicrobium	repeat region	CRISPR Array			11(32-34)	12(36)	NA/NA/NA	CTCTCAGGCCGCTTCGGCGGCTTGCCCGCGTGAAAC	M
2,051,802 -> 2,049,502	-	WP_013419505.1	NC_014664	Rhodomicrobium vannielii ATCC 17100	Bacteria	Proteobacteria	Alphaproteobacteria	Rhizobiales	Hyphomicrobiaceae	Rhodomicrobium	CRISPR-associated RAMP family protein	**csm3gr7**	cls000253	CAS-III-A,CAS-III-D					
2,052,440 -> 2,051,799	-	WP_013419506.1	NC_014664	Rhodomicrobium vannielii ATCC 17100	Bacteria	Proteobacteria	Alphaproteobacteria	Rhizobiales	Hyphomicrobiaceae	Rhodomicrobium	CRISPR-associated protein	**csx19**	cls001376	CAS-III-D					
2,054,125 -> 2,052,437	-	WP_013419507.1	NC_014664	Rhodomicrobium vannielii ATCC 17100	Bacteria	Proteobacteria	Alphaproteobacteria	Rhizobiales	Hyphomicrobiaceae	Rhodomicrobium	H	**csm3gr7**	cd09726	CAS-III-D					
2,056,485 -> 2,054,122	-	WP_041787432.1	NC_014664	Rhodomicrobium vannielii ATCC 17100	Bacteria	Proteobacteria	Alphaproteobacteria	Rhizobiales	Hyphomicrobiaceae	Rhodomicrobium	H	**csm3gr7, csx10gr5**	cd09726, cls000748	CAS-III-D; CAS-III-D					
2,058,251 -> 2,056,512	-	WP_013419509.1	NC_014664	Rhodomicrobium vannielii ATCC 17100	Bacteria	Proteobacteria	Alphaproteobacteria	Rhizobiales	Hyphomicrobiaceae	Rhodomicrobium	H	**cas10**	mkCas0133	CAS-III					
2,058,649 -> 2,059,300	+		NC_014664	Rhodomicrobium vannielii ATCC 17100	Bacteria	Proteobacteria	Alphaproteobacteria	Rhizobiales	Hyphomicrobiaceae	Rhodomicrobium	repeat region	CRISPR Array			9 (31-34)	10 (36)	NA/NA/NA	CTTTCAGGCCGCTTCGGCGGCTTGCCCGCGTGAAAC	M
2,059,716 -> 2,059,378	-	WP_049779317.1	NC_014664	Rhodomicrobium vannielii ATCC 17100	Bacteria	Proteobacteria	Alphaproteobacteria	Rhizobiales	Hyphomicrobiaceae	Rhodomicrobium	H								
2,060,842 -> 2,059,760	-	WP_013419511.1	NC_014664	Rhodomicrobium vannielii ATCC 17100	Bacteria	Proteobacteria	Alphaproteobacteria	Rhizobiales	Hyphomicrobiaceae	Rhodomicrobium	CRISPR-associated protein	**csm6**	pfam09651	CAS-III-A					
2,062,020 -> 2,060,854	-	WP_013419512.1	NC_014664	Rhodomicrobium vannielii ATCC 17100	Bacteria	Proteobacteria	Alphaproteobacteria	Rhizobiales	Hyphomicrobiaceae	Rhodomicrobium	CRISPR-associated protein	**csx1**	pfam09623	CAS-III-A					
2,062,728 -> 2,062,432	-	WP_013419514.1	NC_014664	Rhodomicrobium vannielii ATCC 17100	Bacteria	Proteobacteria	Alphaproteobacteria	Rhizobiales	Hyphomicrobiaceae	Rhodomicrobium	H	csx23	mkCas0160						
2,062,728 -> 2,062,432	-	WP_013419514.1	NC_014664	Rhodomicrobium vannielii ATCC 17100	Bacteria	Proteobacteria	Alphaproteobacteria	Rhizobiales	Hyphomicrobiaceae	Rhodomicrobium	CRISPR-associated Csx16 protein	**csx16**	pfam09652	CAS-I, CAS-III					
2,063,020 -> 2,063,286	+	WP_013419515.1	NC_014664	Rhodomicrobium vannielii ATCC 17100	Bacteria	Proteobacteria	Alphaproteobacteria	Rhizobiales	Hyphomicrobiaceae	Rhodomicrobium	H	cas4	pfam01930						
**CAS-III-B**																			
59556->58543	-	WP_014188711.1	NC_016586.1	Azospirillum lipoferum 4B	Bacteria	Proteobacteria	Alphaproteobacteria	Rhodospirillales	Rhodospirillaceae	Azospirillum	H	**Cas6**	cls000976	CAS-III					
59847 -> 60022	+		NC_016586.1	Azospirillum lipoferum 4B	Bacteria	Proteobacteria	Alphaproteobacteria	Rhodospirillales	Rhodospirillaceae	Azospirillum		CRISPR Array			2 (44,45)	3 (29)	M16/NA/E	GTCGCGACGGCATCCGCGTTCGGGAGCGGATTGAAAC	H
60382->60095	-	WP_014188712.1	NC_016586.1	Azospirillum lipoferum 4B	Bacteria	Proteobacteria	Alphaproteobacteria	Rhodospirillales	Rhodospirillaceae	Azospirillum	CRISPR-associated endonuclease Cas2	**Cas2**	cd09725	CAS-I, CAS-II, CAS-III, CAS-V					
62415->60397	-	WP_014188713.1	NC_016586.1	Azospirillum lipoferum 4B	Bacteria	Proteobacteria	Alphaproteobacteria	Rhodospirillales	Rhodospirillaceae	Azospirillum	CRISPR-associated endonuclease Cas1	**RT-Cas1**	pfam00078, COG1518	CAS-III; CAS-I, CAS-II, CAS-III					
62645 -> 63046	+		NC_016586.1	Azospirillum lipoferum 4B	Bacteria	Proteobacteria	Alphaproteobacteria	Rhodospirillales	Rhodospirillaceae	Azospirillum		CRISPR Array			5 (43-50)	6 (29)	M16/NA/E	GTTCCGCCGGTGTCCGCGTTTGGGAGCGGATTGAAAC	H
64479->63358	-	WP_014188715.1	NC_016586.1	Azospirillum lipoferum 4B	Bacteria	Proteobacteria	Alphaproteobacteria	Rhodospirillales	Rhodospirillaceae	Azospirillum	CRISPR-associated RAMP family protein	**Csm3gr7**	cls000253	CAS-IIIA, CAS-IIID					
65502->67100	+	WP_014188716.1	NC_016586.1	Azospirillum lipoferum 4B	Bacteria	Proteobacteria	Alphaproteobacteria	Rhodospirillales	Rhodospirillaceae	Azospirillum	H								
67124->67777	+	WP_014188717.1	NC_016586.1	Azospirillum lipoferum 4B	Bacteria	Proteobacteria	Alphaproteobacteria	Rhodospirillales	Rhodospirillaceae	Azospirillum	H								
67774->69801	+	WP_014188718.1	NC_016586.1	Azospirillum lipoferum 4B	Bacteria	Proteobacteria	Alphaproteobacteria	Rhodospirillales	Rhodospirillaceae	Azospirillum	H								
69826->71010	+	WP_014188719.1	NC_016586.1	Azospirillum lipoferum 4B	Bacteria	Proteobacteria	Alphaproteobacteria	Rhodospirillales	Rhodospirillaceae	Azospirillum	type III-B CRISPR module RAMP protein Cmr1	**Cmr1gr7**	COG1367	CAS-III-B					
71007->74255	+	WP_014188720.1	NC_016586.1	Azospirillum lipoferum 4B	Bacteria	Proteobacteria	Alphaproteobacteria	Rhodospirillales	Rhodospirillaceae	Azospirillum	type III-B CRISPR-associated protein Cas10/Cmr2	**Cas10**	cls000742, cls000742	CAS-III-B; CAS-III-B					
74252->75487	+	WP_014188721.1	NC_016586.1	Azospirillum lipoferum 4B	Bacteria	Proteobacteria	Alphaproteobacteria	Rhodospirillales	Rhodospirillaceae	Azospirillum	H	**Cmr3gr5**	cd09748	CAS-III-B					
75484->76479	+	WP_014188722.1	NC_016586.1	Azospirillum lipoferum 4B	Bacteria	Proteobacteria	Alphaproteobacteria	Rhodospirillales	Rhodospirillaceae	Azospirillum	type III-B CRISPR module RAMP protein Cmr4	**Cmr4gr7**	COG1336	CAS-III-C					
76602->76913	+	WP_044552090.1	NC_016586.1	Azospirillum lipoferum 4B	Bacteria	Proteobacteria	Alphaproteobacteria	Rhodospirillales	Rhodospirillaceae	Azospirillum	H	**Cmr5gr11**	cls001851	CAS-III-B					
76910->77989	+	WP_014188724.1	NC_016586.1	Azospirillum lipoferum 4B	Bacteria	Proteobacteria	Alphaproteobacteria	Rhodospirillales	Rhodospirillaceae	Azospirillum	type III-B CRISPR module RAMP protein Cmr6	**Cmr6gr7**	cd09661	CAS-III-B					
**CAS-III-D**																			
4264032->4264283	-		NZ_AP014800.1	Rhodovulum sulfidophilum DSM 2351	Bacteria	Proteobacteria	Alphaproteobacteria	Rhodobacterales	Rhodobacteraceae	Rhodovulum		CRISPR Array			3 (33-36)	4 (37)	NA/NA/NA	GTTCGAACGCCATGACCGCGAGGACGCGGATTGAGAC	NA
4264505->4266490	+	WP_060836241.1	NZ_AP014800.1	Rhodovulum sulfidophilum DSM 2351	Bacteria	Proteobacteria	Alphaproteobacteria	Rhodobacterales	Rhodobacteraceae	Rhodovulum	CRISPR-associated endonuclease Cas1	**RT-Cas1**	pfam00078, cd09634	CAS-III; CAS-I, CAS-II, CAS-III, CAS-V					
4266750->4266496	-		NZ_AP014800.1	Rhodovulum sulfidophilum DSM 2351	Bacteria	Proteobacteria	Alphaproteobacteria	Rhodobacterales	Rhodobacteraceae	Rhodovulum		CRISPR Array			3 (33,41)	4 (37)	NA/NA/NA	GTCCGTGACCCATGTCCTCGAAGACGAGGATTGAGAC	NA
4266997->4267284	+	WP_060836242.1	NZ_AP014800.1	Rhodovulum sulfidophilum DSM 2351	Bacteria	Proteobacteria	Alphaproteobacteria	Rhodobacterales	Rhodobacteraceae	Rhodovulum	CRISPR-associated endonuclease Cas2	**Cas2**	pfam09827	CAS-I, CAS-II, CAS-III					
4267529->4268302	+	WP_060836243.1	NZ_AP014800.1	Rhodovulum sulfidophilum DSM 2351	Bacteria	Proteobacteria	Alphaproteobacteria	Rhodobacterales	Rhodobacteraceae	Rhodovulum	H	**Cas6**	mkCas0066	CAS-I, CAS-III					
4268304->4270139	+	WP_060836244.1	NZ_AP014800.1	Rhodovulum sulfidophilum DSM 2351	Bacteria	Proteobacteria	Alphaproteobacteria	Rhodobacterales	Rhodobacteraceae	Rhodovulum	H	**Cas10**	cls000707	CAS-III					
4270136->4271938	+	WP_060836245.1	NZ_AP014800.1	Rhodovulum sulfidophilum DSM 2351	Bacteria	Proteobacteria	Alphaproteobacteria	Rhodobacterales	Rhodobacteraceae	Rhodovulum	H	**Csm4gr5**	cls000259	CAS-III-D					
4271935->4273203	+	WP_060836246.1	NZ_AP014800.1	Rhodovulum sulfidophilum DSM 2351	Bacteria	Proteobacteria	Alphaproteobacteria	Rhodobacterales	Rhodobacteraceae	Rhodovulum	H	**Csm3gr7**	cd09683, cd09726	CAS-III-A,CAS-III-D; CAS-III-D					
4273196->4275346	+	WP_060836247.1	NZ_AP014800.1	Rhodovulum sulfidophilum DSM 2351	Bacteria	Proteobacteria	Alphaproteobacteria	Rhodobacterales	Rhodobacteraceae	Rhodovulum	CRISPR-associated RAMP family protein	**Csm3gr7**	cls000253	CAS-III-A, CAS-III-D					
4275534 -> 4275931	+		NZ_AP014800.1	Rhodovulum sulfidophilum DSM 2351	Bacteria	Proteobacteria	Alphaproteobacteria	Rhodobacterales	Rhodobacteraceae	Rhodovulum		CRISPR Array			5 (34-37)	6 (37)	NA/NA/NA	GTGCGCAGCCCATGTCCGCGAAGACGCGGATTGAGAC	H
**Partial CAS-III-D**																			
1167219->1168829	+	WP_042442596.1	NC_013854.1	Azospirillum sp. B510	Bacteria	Proteobacteria	Alphaproteobacteria	Rhodospirillales	Rhodospirillaceae	Azospirillum	H	**Cas10**	cls000707	CAS-III					
1168826->1170592	+	WP_042442599.1	NC_013854.1	Azospirillum sp. B510	Bacteria	Proteobacteria	Alphaproteobacteria	Rhodospirillales	Rhodospirillaceae	Azospirillum	H	**Csm3gr7**	cls000670	CAS-III-D					
1170585->1173296	+	WP_012973660.1	NC_013854.1	Azospirillum sp. B510	Bacteria	Proteobacteria	Alphaproteobacteria	Rhodospirillales	Rhodospirillaceae	Azospirillum	H	**Csm3gr7**	cd09726, cls000253	CAS-III-D; CAS-IIA, CAS-III-D					
1173332->1173970	+	WP_012973661.1	NC_013854.1	Azospirillum sp. B510	Bacteria	Proteobacteria	Alphaproteobacteria	Rhodospirillales	Rhodospirillaceae	Azospirillum	H	**Csm3gr7**	cls000253	CAS-III-A, CAS-III-D					
1175114->1174161	-	WP_012973662.1	NC_013854.1	Azospirillum sp. B510	Bacteria	Proteobacteria	Alphaproteobacteria	Rhodospirillales	Rhodospirillaceae	Azospirillum	H	**Cas6**	cls000976	CAS-III					
1175854->1175567	-	WP_012973663.1	NC_013854.1	Azospirillum sp. B510	Bacteria	Proteobacteria	Alphaproteobacteria	Rhodospirillales	Rhodospirillaceae	Azospirillum	CRISPR-associated endonuclease Cas2	**Cas2**	cd09725	CAS-I, CAS-II, CAS-III, CAS-V					
1177894->1175897	-	WP_012973664.1	NC_013854.1	Azospirillum sp. B510	Bacteria	Proteobacteria	Alphaproteobacteria	Rhodospirillales	Rhodospirillaceae	Azospirillum	CRISPR-associated endonuclease Cas1	**RT-Cas1**	pfam00078, COG1518	CAS-III; CAS-I, CAS-II, CAS-III					
1178058 -> 1178606	+		NC_013854.1	Azospirillum sp. B510	Bacteria	Proteobacteria	Alphaproteobacteria	Rhodospirillales	Rhodospirillaceae	Azospirillum		CRISPR Array			7 (32-44)	8 (35)	M14/NA/E	AGTTCGGAGGCGTTCCGCTATGGGAAGCGGATTGGAA	L
**CAS-III-B/CAS-III-C**																			
1133577->1133257	-	WP_019960641.1	NZ_KB908056.1	Woodsholea maritima DSM 17123	Bacteria	Proteobacteria	Alphaproteobacteria	Caulobacterales	Caulobacteraceae	Woodsholea	putative CRISPR-associated protein	**Csx16**	pfam09652	CAS-I, CAS-III					
1133629->1134942	+	WP_019960642.1	NZ_KB908056.1	Woodsholea maritima DSM 17123	Bacteria	Proteobacteria	Alphaproteobacteria	Caulobacterales	Caulobacteraceae	Woodsholea	H								
1135707->1134955	-	WP_019960643.1	NZ_KB908056.1	Woodsholea maritima DSM 17123	Bacteria	Proteobacteria	Alphaproteobacteria	Caulobacterales	Caulobacteraceae	Woodsholea	type III-B CRISPR module RAMP protein Cmr6	**Cmr6gr7**	cd09661	CAS-III-B					
1136107->1135712	-	WP_019960644.1	NZ_KB908056.1	Woodsholea maritima DSM 17123	Bacteria	Proteobacteria	Alphaproteobacteria	Caulobacterales	Caulobacteraceae	Woodsholea	H								
1136955->1136104	-	WP_019960645.1	NZ_KB908056.1	Woodsholea maritima DSM 17123	Bacteria	Proteobacteria	Alphaproteobacteria	Caulobacterales	Caulobacteraceae	Woodsholea	type III-B CRISPR module RAMP protein Cmr4	**Cmr4gr7**	COG1336	CAS-III-C					
1138145->1136955	-	WP_019960646.1	NZ_KB908056.1	Woodsholea maritima DSM 17123	Bacteria	Proteobacteria	Alphaproteobacteria	Caulobacterales	Caulobacteraceae	Woodsholea	H	**Cmr3gr5**	cd09748	CAS-III-B					
1140030->1138138	-	WP_019960647.1	NZ_KB908056.1	Woodsholea maritima DSM 17123	Bacteria	Proteobacteria	Alphaproteobacteria	Caulobacterales	Caulobacteraceae	Woodsholea	H	**Cas10**	cls000742	CAS-III					
1141928->1140318	-	WP_019960648.1	NZ_KB908056.1	Woodsholea maritima DSM 17123	Bacteria	Proteobacteria	Alphaproteobacteria	Caulobacterales	Caulobacteraceae	Woodsholea	type III-B CRISPR module RAMP protein Cmr1	**Cmr1gr7**	cd09657	CAS-III-C					
1142682->1144643	+	WP_019960649.1	NZ_KB908056.1	Woodsholea maritima DSM 17123	Bacteria	Proteobacteria	Alphaproteobacteria	Caulobacterales	Caulobacteraceae	Woodsholea	CRISPR-associated endonuclease Cas1	**RT-Cas1**	pfam00078, COG1518	CAS-III; CAS-I, CAS-II, CAS-III					
1144646->1144927	+	WP_019960650.1	NZ_KB908056.1	Woodsholea maritima DSM 17123	Bacteria	Proteobacteria	Alphaproteobacteria	Caulobacterales	Caulobacteraceae	Woodsholea	CRISPR-associated endonuclease Cas2	**Cas2**	pfam09827	CAS-I, CAS-II, CAS-III					
1145005->1145832	+	WP_019960651.1	NZ_KB908056.1	Woodsholea maritima DSM 17123	Bacteria	Proteobacteria	Alphaproteobacteria	Caulobacterales	Caulobacteraceae	Woodsholea	H	**Cas6**	cls000976	CAS-III					
1141995 -> 1142515	ND		NZ_KB908056.1	Woodsholea maritima DSM 17123	Bacteria	Proteobacteria	Alphaproteobacteria	Caulobacterales	Caulobacteraceae	Woodsholea		CRISPR Array			7 (28-35)	8 (36)	NA/NA/E	GTTTTGATCAGCTCTGCAGAAAGATTCGAGCAGAGC	NA
**CAS-III-D**																			
32134 ->32730	+	KQB14195.1	LLVV01000015.1	Rhodobacter capsulatus strain A52	Bacteria	Proteobacteria	Alphaproteobacteria	Rhodobacterales	Rhodobacteraceae	Rhodobacter	H	**Cas6**	cls000976	CAS-III					

33657 ->32749	+	KQB14194.1	LLVV01000015.1	Rhodobacter capsulatus strain A52	Bacteria	Proteobacteria	Alphaproteobacteria	Rhodobacterales	Rhodobacteraceae	Rhodobacter	H	Cas10	cls000707	CAS-III					
33901 ->35739	+	KQB14193.1	LLVV01000015.1	Rhodobacter capsulatus strain A52	Bacteria	Proteobacteria	Alphaproteobacteria	Rhodobacterales	Rhodobacteraceae	Rhodobacter	H	Csm4gr5	cls000259	CAS-III-D					
35736 ->37619	+	KQB14192.1	LLVV01000015.1	Rhodobacter capsulatus strain A52	Bacteria	Proteobacteria	Alphaproteobacteria	Rhodobacterales	Rhodobacteraceae	Rhodobacter	H	Csm3gr7	cd09683, cd09683						
37619 ->39193	+	KQB14191.1	LLVV01000015.1	Rhodobacter capsulatus strain A52	Bacteria	Proteobacteria	Alphaproteobacteria	Rhodobacterales	Rhodobacteraceae	Rhodobacter	H	Csm3gr7	cls000253, cls000253	CAS-III-A, CAS-III-D; CAS-III-A, CAS-III-D					
39190 ->41166	+	KQB14190.1	LLVV01000015.1	Rhodobacter capsulatus strain A52	Bacteria	Proteobacteria	Alphaproteobacteria	Rhodobacterales	Rhodobacteraceae	Rhodobacter	H	RT-Cas1	pfam00078, pfam01867	CAS-III; CAS-I, CAS-II, CAS-III					
41408->41240	ND		LLVV01000015.1	Rhodobacter capsulatus strain A52	Bacteria	Proteobacteria	Alphaproteobacteria	Rhodobacterales	Rhodobacteraceae	Rhodobacter		CRISPR Array			2 (28,29)	3 (37)	M17/NA/E	GTCTGGCACCGCGTCCGGCGGCCCTGCCGGTTGGCAC	NA
41568 ->43580	+	KQB14189.1	LLVV01000015.1	Rhodobacter capsulatus strain A52	Bacteria	Proteobacteria	Alphaproteobacteria	Rhodobacterales	Rhodobacteraceae	Rhodobacter	H	Cas2	pfam09827	CAS-I, CAS-II, CAS-III					
44024 -> 44258	ND		LLVV01000015.1	Rhodobacter capsulatus strain A52	Bacteria	Proteobacteria	Alphaproteobacteria	Rhodobacterales	Rhodobacteraceae	Rhodobacter		CRISPR Array			3 (28-30)	4 (37)	M17/NA/E	GTCTGGCACCATGTCCGGCGGCCCTGCCGGTTGGCAC	NA
Partial CAS-III-D																			
1895008 ->1894474	-		NC_014365.1	Desulfarculus baarsii DSM 2075	Bacteria	Proteobacteria	Deltaproteobacteria	Desulfarcucales	Desulfarculaceae	Desulfarculus		CRISPR Array			7 (31-38)	8 (37)	M16/NA/E	CTATTGAGGGATGCCGCTAGAGGAAGCGGATTGAAAC	H
1897155 ->1895233	-	WP_043814083.1	NC_014365.1	Desulfarculus baarsii DSM 2075	Bacteria	Proteobacteria	Deltaproteobacteria	Desulfarcucales	Desulfarculaceae	Desulfarculus	CRISPR-associated RAMP family protein	Csm3gr7	cls000253, cls000253	CAS-III-A, CAS-III-D; CAS-III-A, CAS-III-D					
1898626 ->1897208	-	WP_043814085.1	NC_014365.1	Desulfarculus baarsii DSM 2075	Bacteria	Proteobacteria	Deltaproteobacteria	Desulfarcucales	Desulfarculaceae	Desulfarculus	H	Csm3gr7	cd09726	CAS-III-D					
1900472 ->1898610	-	WP_013258510.1	NC_014365.1	Desulfarculus baarsii DSM 2075	Bacteria	Proteobacteria	Deltaproteobacteria	Desulfarcucales	Desulfarculaceae	Desulfarculus	H	Csm3gr7	cls000670	CAS-III-D					
1902763 -> 1902297	-		NC_014365.1	Desulfarculus baarsii DSM 2075	Bacteria	Proteobacteria	Deltaproteobacteria	Desulfarcucales	Desulfarculaceae	Desulfarculus		CRISPR Array			6 (33-37)	7 (37)	M16/NA/E	CTATTGAGGGATGCCGCTAGAGGAAGCGGATTGAAAC	H
1902120 ->1900465	-	WP_013258511.1	NC_014365.1	Desulfarculus baarsii DSM 2075	Bacteria	Proteobacteria	Deltaproteobacteria	Desulfarcucales	Desulfarculaceae	Desulfarculus	H	Cas10	cls000707	CAS-III					
1902918 ->1904885	+	WP_013258512.1	NC_014365.1	Desulfarculus baarsii DSM 2075	Bacteria	Proteobacteria	Deltaproteobacteria	Desulfarcucales	Desulfarculaceae	Desulfarculus	CRISPR-associated endonuclease Cas1	RT-Cas1	pfam00078, pfam01867	CAS-III; CAS-I, CAS-II, CAS-III					
1904897 ->1905184	+	WP_013258513.1	NC_014365.1	Desulfarculus baarsii DSM 2075	Bacteria	Proteobacteria	Deltaproteobacteria	Desulfarcucales	Desulfarculaceae	Desulfarculus	CRISPR-associated protein Cas2	Cas2	pfam09827	CAS-I, CAS-II, CAS-III					
1905197 ->1906117	+	WP_013258514.1	NC_014365.1	Desulfarculus baarsii DSM 2075	Bacteria	Proteobacteria	Deltaproteobacteria	Desulfarcucales	Desulfarculaceae	Desulfarculus	H	Cas6	cls000976	CAS-III					

RT-CRISPR-8
Nitrospira (Nitrospirales); Plantomycetia (Plantomycetales); Deltaproteobacteria (Desulfovibrionales, Syntrophobacterales); Betaproteobacteria; Gammaproteobacteria (Chromatiales, Cellvibrionales, Thiotrichales, Vibrionales, Oceanospirillales, Pseudomonadales)

RT-CRISPR-8a (CAS-III-A/D, CAS-III-B)
Gammaproteobacteria (Chromatiales, Cellvibrionales, Thiotrichales, Vibrionales, Oceanospirillales)

[a](Sub) Type/[b]Coordinates	Strand	Protein_id	Genome Partition ID	Strain	Domain	Phylum	Lineage (class)	Order	Family	Genus	[c]Cas gene (annotated)	[d]Cas gene/[e]Array (identified)	[d]Profiles	[d](Sub)Type-specificity	[e]Spacers (length)	[e]Repeats (length)	[f]Str.motif/Seq.family/Superclass	[f]Seq Repeats	[g]Orientation Confidence
CAS-III-D																			
62 -> 438	+		AVFR01001361	uncultured Thiohalocapsa sp. PB-PSB1	Bacteria	Proteobacteria	Gammaproteobacteria	Chromatiales	Chromatiaceae	Thiohalocapsa		CRISPR array			5 (33-37)	6 (35)	NA/NA/NA	GCCTCATTTCCTTTAGCCGTTCAGGCTGTTGAAAC	M
867->2069	+	ESQ08047.1	AVFR01001361	uncultured Thiohalocapsa sp. PB-PSB1	Bacteria	Proteobacteria	Gammaproteobacteria	Chromatiales	Chromatiaceae	Thiohalocapsa	H	Cas10	cls000707	CAS-III					
1967->2707	+	ESQ08046.1	AVFR01001361	uncultured Thiohalocapsa sp. PB-PSB1	Bacteria	Proteobacteria	Gammaproteobacteria	Chromatiales	Chromatiaceae	Thiohalocapsa	H	Cas10	cls000707	CAS-III					
2704->4602	+	ESQ08045.1	AVFR01001361	uncultured Thiohalocapsa sp. PB-PSB1	Bacteria	Proteobacteria	Gammaproteobacteria	Chromatiales	Chromatiaceae	Thiohalocapsa	H	Csm4gr5	cls000259	CAS-III-D					
4589->6199	+	ESQ08044.1	AVFR01001361	uncultured Thiohalocapsa sp. PB-PSB1	Bacteria	Proteobacteria	Gammaproteobacteria	Chromatiales	Chromatiaceae	Thiohalocapsa	H	Csm3gr7	COG1337, cd09683	CAS-III-D; CAS-III-A, CAS-III-D					
6192->8864	+	ESQ08043.1	AVFR01001361	uncultured Thiohalocapsa sp. PB-PSB1	Bacteria	Proteobacteria	Gammaproteobacteria	Chromatiales	Chromatiaceae	Thiohalocapsa	H	Csm3gr7	cls000253	CAS-IIIA, CAS-III-D					
8725->11682	+	ESQ08042.1	AVFR01001361	uncultured Thiohalocapsa sp. PB-PSB1	Bacteria	Proteobacteria	Gammaproteobacteria	Chromatiales	Chromatiaceae	Thiohalocapsa	H	Cas6-RT-Cas1	mkCas0066, pfam0078, COG1518	CAS-I, CAS-III; CAS-III; CAS-I, CAS-II, CAS-III					
11682->11963	+	ESQ08041.1	AVFR01001361	uncultured Thiohalocapsa sp. PB-PSB1	Bacteria	Proteobacteria	Gammaproteobacteria	Chromatiales	Chromatiaceae	Thiohalocapsa	H	Cas2	pfam09827	CAS-I,CAS-II,CAS-III					
CAS-III-A/CAS-III-D																			
32743->34494	+	CRI67875.1	CVPF01000361	Thiocapsa sp. KS1	Bacteria	Proteobacteria	Gammaproteobacteria	Chromatiales	Chromatiaceae	Thicocapsa	H	Cas10	cls000707	CAS-III					
34491->36347	+	CRI67874.1	CVPF01000361	Thiocapsa sp. KS1	Bacteria	Proteobacteria	Gammaproteobacteria	Chromatiales	Chromatiaceae	Thicocapsa	H	Csm4gr5	cls000259	CAS-III-D					
36334->37830	+	CRI67873.1	CVPF01000361	Thiocapsa sp. KS1	Bacteria	Proteobacteria	Gammaproteobacteria	Chromatiales	Chromatiaceae	Thicocapsa	H	Csm3gr7	pfam03787, cd09683	CAS-III-A, CAS-III-D; CAS-IIIA, CAS-III-D					
37849->40242	+	CRI67872.1	CVPF01000361	Thiocapsa sp. KS1	Bacteria	Proteobacteria	Gammaproteobacteria	Chromatiales	Chromatiaceae	Thicocapsa	putative Cold-shock DNA-binding protein family	Csm3gr7	cls000253, cls000253	CAS-III-A, CAS-III-D; CAS-III-A, CAS-III-D					
40239->43286	+	CRI67871.1	CVPF01000361	Thiocapsa sp. KS1	Bacteria	Proteobacteria	Gammaproteobacteria	Chromatiales	Chromatiaceae	Thicocapsa	putative RNA-directed DNA polymerase	Cas6-RT-Cas1	mkCas0066, pfam0078, pfam01867	CAS-I, CAS-III; CAS-III; CAS-I, CAS-II, CAS-III					
43271->43564	+	CRI67870.1	CVPF01000361	Thiocapsa sp. KS1	Bacteria	Proteobacteria	Gammaproteobacteria	Chromatiales	Chromatiaceae	Thicocapsa	putative CRISPR-associated protein Cas2	Cas2	cd09638	CAS-I, CAS-II, CAS-III					
43823 -> 44200	+		CVPF01000361	Thiocapsa sp. KS1	Bacteria	Proteobacteria	Gammaproteobacteria	Chromatiales	Chromatiaceae	Thicocapsa		CRISPR array			5 (33-34)	6 (35)	M14/NA/NA	GTCATCCAGCCCATAGCCGTTCAGGCTGTTGAAAC	NA
Unknown																			
1 -> 2435	+	ESQ17084.1	AVFR01000074	uncultured Thiohalocapsa sp. PB-PSB1	Bacteria	Proteobacteria	Gammaproteobacteria	Chromatiales	Chromatiaceae	Thiohalocapsa	H	Cas6-RT-Cas1	mkCas0091, pfam0078, pfam01867	CAS-I, CAS-III; CAS-III; CAS-I, CAS-II, CAS-III					
2551 -> 2829	+	ESQ17085.1	AVFR01000074	uncultured Thiohalocapsa sp. PB-PSB1	Bacteria	Proteobacteria	Gammaproteobacteria	Chromatiales	Chromatiaceae	Thiohalocapsa	H	Cas2	pfam09827	CAS-I,CAS-II,CAS-III					
3078 -> 3811	+		AVFR01000074	uncultured Thiohalocapsa sp. PB-PSB1	Bacteria	Proteobacteria	Gammaproteobacteria	Chromatiales	Chromatiaceae	Thiohalocapsa		CRISPR array			10 (34-38)	11(35)	NA/NA/E	GTTGAATCCATTCAGAGCTGCTAAAGCTGTGCGAC	L
CAS-III-B (it has not a Cas1 recognizable domain*)																			
4826430 -> 4825348	-	WP_015817246.1	NC_012997.1	Teredinibacter turnerae T7901	Bacteria	Proteobacteria	Gammaproteobacteria	Cellvibrionales	C ellvibrionaceae	Teredinibacter	Cmr6	Cmr6gr7	COG1604	CAS-III-B					
4826815 -> 4826414	-	WP_015817724.1	NC_012997.1	Teredinibacter turnerae T7901	Bacteria	Proteobacteria	Gammaproteobacteria	Cellvibrionales	C ellvibrionaceae	Teredinibacter	Cmr5	Cmr5gr11	pfam09701	CAS-III-B					
4827708 -> 4826812	-	WP_015817966.1	NC_012997.1	Teredinibacter turnerae T7901	Bacteria	Proteobacteria	Gammaproteobacteria	Cellvibrionales	C ellvibrionaceae	Teredinibacter	Cmr4	Cmr4gr7	COG1336	CAS-III-C					
4828911 -> 4827724	-	WP_015818380.1	NC_012997.1	Teredinibacter turnerae T7901	Bacteria	Proteobacteria	Gammaproteobacteria	Cellvibrionales	C ellvibrionaceae	Teredinibacter	Cmr3	Cmr3gr5	cd09748	CAS-III-B					
4830721 -> 4828916	-	WP_015819132.1	NC_012997.1	Teredinibacter turnerae T7901	Bacteria	Proteobacteria	Gammaproteobacteria	Cellvibrionales	C ellvibrionaceae	Teredinibacter	Cmr2	Cas10	cls000742	CAS-III					
4832135 -> 4830714	-	WP_015819721.1	NC_012997.1	Teredinibacter turnerae T7901	Bacteria	Proteobacteria	Gammaproteobacteria	Cellvibrionales	C ellvibrionaceae	Teredinibacter	Cmr1	Cmr1gr7	COG1367	CAS-III-B					
4834602 -> 4832581	-	WP_015817555.1	NC_012997.1	Teredinibacter turnerae T7901	Bacteria	Proteobacteria	Gammaproteobacteria	Cellvibrionales	C ellvibrionaceae	Teredinibacter	H	Cas6-RT*	mkCas0091, pfam00078	CAS-I, CAS-III					
4836538 -> 4834731	-		NC_012997.1	Teredinibacter turnerae T7901	Bacteria	Proteobacteria	Gammaproteobacteria	Cellvibrionales	C ellvibrionaceae	Teredinibacter		CRISPR array			25 (33-36)	26 (37)	NA/F17/E	GCGTCGCTGGAGGTCTAGCCGCTAAGGCTGTTGAAAC	L
CAS-III-D																			
47863->49812	+	WP_019606011.1	NZ_KB906812	Teredinibacter turnerae T8412	Bacteria	Proteobacteria	Gammaproteobacteria	Cellvibrionales	C ellvibrionaceae	Teredinibacter	H	Cas10	cls000707	CAS-III					
49809->51824	+	WP_019606012.1	NZ_KB906812	Teredinibacter turnerae T8412	Bacteria	Proteobacteria	Gammaproteobacteria	Cellvibrionales	C ellvibrionaceae	Teredinibacter	H	Csm4gr5	cls000259	CAS-III-D					
51821->53680	+	WP_019606013.1	NZ_KB906812	Teredinibacter turnerae T8412	Bacteria	Proteobacteria	Gammaproteobacteria	Cellvibrionales	C ellvibrionaceae	Teredinibacter	H	Csm3gr7	COG1337, cd09683	CAS-III-D; CAS-III-A, CAS-III-D					
53677->55314	+	WP_019606014.1	NZ_KB906812	Teredinibacter turnerae T8412	Bacteria	Proteobacteria	Gammaproteobacteria	Cellvibrionales	C ellvibrionaceae	Teredinibacter	H	Csm3gr7	cls000253, cls000253	CAS-III-A, CAS-III-D					
55331->55891	+	WP_019606015.1	NZ_KB906812	Teredinibacter turnerae T8412	Bacteria	Proteobacteria	Gammaproteobacteria	Cellvibrionales	C ellvibrionaceae	Teredinibacter	H								
55932->58976	+	WP_019606016.1	NZ_KB906812	Teredinibacter turnerae T8412	Bacteria	Proteobacteria	Gammaproteobacteria	Cellvibrionales	C ellvibrionaceae	Teredinibacter	CRISPR-associated endonuclease Cas1	Cas6-RT-Cas1	mkCas0091, pfam0078, cd09634	RT;CAS-I, CAS-II, CAS-III, CAS-V					
59045->59269	+	WP_037991351.1	NZ_KB906812	Teredinibacter turnerae T8412	Bacteria	Proteobacteria	Gammaproteobacteria	Cellvibrionales	C ellvibrionaceae	Teredinibacter	CRISPR-associated endonuclease Cas2	Cas2	mkCas0081	CAS-I, CAS-II, CAS-III					
59558 -> 59876	+		NZ_KB906812	Teredinibacter turnerae T8412	Bacteria	Proteobacteria	Gammaproteobacteria	Cellvibrionales	C ellvibrionaceae	Teredinibacter		CRISPR array			4 (34-37)	5 (35)	NA/NA/E	GTCGCAATCGACATTAGCCGCTAAGGCTGTGAAAC	H
CAS-III-D																			
1784134 ->1782307	-		NZ_JQMT01000001	Thiomicrospora sp. Milos-T1	Bacteria	Proteobacteria	Gammaproteobacteria	Thiotricalles	Piscirickettsiaceae	Thiomicrospora		CRISPR array			26 (30-36)	27 (35)	M4/NA/E	GTTCTAGAACCCCAAAGCCGAAAGGCTGTTAAGAC	M
1784682->1784392	-	WP_038137808.1	NZ_JQMT01000001	Thiomicrospora sp. Milos-T1	Bacteria	Proteobacteria	Gammaproteobacteria	Thiotricalles	Piscirickettsiaceae	Thiomicrospora	CRISPR-associated endonuclease Cas2	Cas2	pfam09827	CAS-I, CAS-II, CAS-III					
1787661->1784716	-	WP_038137810.1	NZ_JQMT01000001	Thiomicrospora sp. Milos-T1	Bacteria	Proteobacteria	Gammaproteobacteria	Thiotricalles	Piscirickettsiaceae	Thiomicrospora	CRISPR-associated endonuclease Cas1	Cas6-RT-Cas1	mkCas0066, pfam00078, pfam01867	RT; CAS-I, CAS-II, CAS-III					
1787968->1787678	-	WP_038137813.1	NZ_JQMT01000001	Thiomicrospora sp. Milos-T1	Bacteria	Proteobacteria	Gammaproteobacteria	Thiotricalles	Piscirickettsiaceae	Thiomicrospora	H								
1790125->1787999	-	WP_038137815.1	NZ_JQMT01000001	Thiomicrospora sp. Milos-T1	Bacteria	Proteobacteria	Gammaproteobacteria	Thiotricalles	Piscirickettsiaceae	Thiomicrospora	CRISPR-associated RAMP family protein	Csm3gr7	cls000253	CAS-III-A, CAS-III-D					
1790616->1790125	-	WP_038137817.1	NZ_JQMT01000001	Thiomicrospora sp. Milos-T1	Bacteria	Proteobacteria	Gammaproteobacteria	Thiotricalles	Piscirickettsiaceae	Thiomicrospora	H								
1792049->1790613	-	WP_038137818.1	NZ_JQMT01000001	Thiomicrospora sp. Milos-T1	Bacteria	Proteobacteria	Gammaproteobacteria	Thiotricalles	Piscirickettsiaceae	Thiomicrospora	H	Csm3gr7	cd09726, cd09726	CAS-III-D; CAS-III-D					
1793554->1792049	-	WP_038137821.1	NZ_JQMT01000001	Thiomicrospora sp. Milos-T1	Bacteria	Proteobacteria	Gammaproteobacteria	Thiotricalles	Piscirickettsiaceae	Thiomicrospora	H	Csx10gr5	cls000748	CAS-III-D					
1794135->1793551	-	WP_038137823.1	NZ_JQMT01000001	Thiomicrospora sp. Milos-T1	Bacteria	Proteobacteria	Gammaproteobacteria	Thiotricalles	Piscirickettsiaceae	Thiomicrospora	H	Csm3gr7	cd09726	CAS-III-D					
1795805->1794138	-	WP_038137826.1	NZ_JQMT01000001	Thiomicrospora sp. Milos-T1	Bacteria	Proteobacteria	Gammaproteobacteria	Thiotricalles	Piscirickettsiaceae	Thiomicrospora	H	Cas10	cls000686	CAS-III					
1796377->1797501	-	WP_051920144.1	NZ_JQMT01000001	Thiomicrospora sp. Milos-T1	Bacteria	Proteobacteria	Gammaproteobacteria	Thiotricalles	Piscirickettsiaceae	Thiomicrospora	CRISPR-associated protein	Csx1	pfam09623	CAS-III					
CAS-III-D																			
142948 -> 144768	+	KUI97415.1	LQIY01000027	Vibrio sp. MEBiC08052 200.VRK.1_28	Bacteria	Proteobacteria	Gammaproteobacteria	Vibrionales	Vibrionaceae	Vibrio	H	Cas10	mkCas0133	CAS-III					
144761 -> 146848	+	KUI97416.1	LQIY01000027	Vibrio sp. MEBiC08052 200.VRK.1_28	Bacteria	Proteobacteria	Gammaproteobacteria	Vibrionales	Vibrionaceae	Vibrio	H	Csx10gr5	cls000715	CAS-III-D					
146845 -> 148296	+	KUI97417.1	LQIY01000027	Vibrio sp. MEBiC08052 200.VRK.1_28	Bacteria	Proteobacteria	Gammaproteobacteria	Vibrionales	Vibrionaceae	Vibrio	H	Csm3gr7	cd09726, cd09726	CAS-III-D; CAS-III-D					
148293 -> 148892	+	KUI97418.1	LQIY01000027	Vibrio sp. MEBiC08052 200.VRK.1_28	Bacteria	Proteobacteria	Gammaproteobacteria	Vibrionales	Vibrionaceae	Vibrio	H								
148892 -> 151057	+	KUI97419.1	LQIY01000027	Vibrio sp. MEBiC08052 200.VRK.1_28	Bacteria	Proteobacteria	Gammaproteobacteria	Vibrionales	Vibrionaceae	Vibrio	H	Csm3gr7	cls000253, cls000253	CAS-III-A, CAS-III-D; CAS-IIIA, CAS-III-D					
151066 -> 153126	+	KUI97420.1	LQIY01000027	Vibrio sp. MEBiC08052 200.VRK.1_28	Bacteria	Proteobacteria	Gammaproteobacteria	Vibrionales	Vibrionaceae	Vibrio	H								
153179 -> 155965	+	KUI97421.1	LQIY01000027	Vibrio sp. MEBiC08052 200.VRK.1_28	Bacteria	Proteobacteria	Gammaproteobacteria	Vibrionales	Vibrionaceae	Vibrio	H	Cas6-RT-Cas1	mkCas0066, pfam0078, cd09634	CAS-I, CAS-III;CAS-III; CAS-I, CAS-II, CAS-III, CAS-V					
155981 -> 156286	+		LQIY01000027	Vibrio sp. MEBiC08052 200.VRK.1_28	Bacteria	Proteobacteria	Gammaproteobacteria	Vibrionales	Vibrionaceae	Vibrio	CRISPR-associated protein Cas2	Cas2	pfam09827	CAS-I,CAS-II,CAS-III					
												No-Array (end-contig)							
Partial CAS-III-A/CAS- III-D																			
208300->210123	+	WP_028302061.1	NZ_KE383828	Oceanospirillum beijerinckii DSM 7166	Bacteria	Proteobacteria	Gammaproteobacteria	Oceanospirillales	Oceanospirilaceae	Oceanospirillum	H	Cas10	mkCas0133	CAS-III					
210134->212329	+	WP_028302062.1	NZ_KE383828	Oceanospirillum beijerinckii DSM 7166	Bacteria	Proteobacteria	Gammaproteobacteria	Oceanospirillales	Oceanospirilaceae	Oceanospirillum	H	Csx10gr5	cd09700						
212326->213837	+	WP_028302063.1	NZ_KE383828	Oceanospirillum beijerinckii DSM 7166	Bacteria	Proteobacteria	Gammaproteobacteria	Oceanospirillales	Oceanospirilaceae	Oceanospirillum	H	Csm3gr7	cd09683						
213834->214391	+	WP_028302064.1	NZ_KE383828	Oceanospirillum beijerinckii DSM 7166	Bacteria	Proteobacteria	Gammaproteobacteria	Oceanospirillales	Oceanospirilaceae	Oceanospirillum	H								
214394->216628	+	WP_028302065.1	NZ_KE383828	Oceanospirillum beijerinckii DSM 7166	Bacteria	Proteobacteria	Gammaproteobacteria	Oceanospirillales	Oceanospirilaceae	Oceanospirillum	H	Csm3gr7	cls000253	CAS-III-A, CAS-III-D					
216653->218728	+	WP_028302066.1	NZ_KE383828	Oceanospirillum beijerinckii DSM 7166	Bacteria	Proteobacteria	Gammaproteobacteria	Oceanospirillales	Oceanospirilaceae	Oceanospirillum	H								
218747->221563	+	WP_028302067.1	NZ_KE383828	Oceanospirillum beijerinckii DSM 7166	Bacteria	Proteobacteria	Gammaproteobacteria	Oceanospirillales	Oceanospirilaceae	Oceanospirillum	CRISPR-associated endonuclease Cas1	Cas6-RT-Cas1	mkCas0066, pfam0078, cd09634	CAS-I, CAS-III;CAS-III; CAS-I, CAS-II, CAS-III, CAS-V					
221603->221896	+	WP_036566871.1	NZ_KE383828	Oceanospirillum beijerinckii DSM 7166	Bacteria	Proteobacteria	Gammaproteobacteria	Oceanospirillales	Oceanospirilaceae	Oceanospirillum	CRISPR-associated endonuclease Cas2	Cas2	pfam09827	CAS-I, CAS-II, CAS-III					
												No-Array (assembly gap)							
Partial CAS-III-A/CAS- III-D																			
37356 -> 39038	+	WP_036801315.1	NZ_AMZO01000034	Photobacterium marinum AK15	Bacteria	Proteobacteria	Gammaproteobacteria	Vibrionalles	Vibrionaceae	Photobacterium	H	Cas10	mkCas0133	CAS-III					
39035 -> 41227	+	WP_036801317.1	NZ_AMZO01000034	Photobacterium marinum AK15	Bacteria	Proteobacteria	Gammaproteobacteria	Vibrionalles	Vibrionaceae	Photobacterium	H	Csm3gr7	cd09683						
41220 -> 42740	+	WP_007469736.1	NZ_AMZO01000034	Photobacterium marinum AK15	Bacteria	Proteobacteria	Gammaproteobacteria	Vibrionalles	Vibrionaceae	Photobacterium	DUF324 domain-containing protein	Csm3gr7	cd09683						
42740 -> 43348	+	WP_007469738.1	NZ_AMZO01000034	Photobacterium marinum AK15	Bacteria	Proteobacteria	Gammaproteobacteria	Vibrionalles	Vibrionaceae	Photobacterium	H								
43345 -> 45345	+	WP_007469740.1	NZ_AMZO01000034	Photobacterium marinum AK15	Bacteria	Proteobacteria	Gammaproteobacteria	Vibrionalles	Vibrionaceae	Photobacterium	DUF324 domain-containing protein	Csm3gr7	cls000253	CAS-III-A, CAS-III-D					
45348 -> 47393	+	WP_007469741.1	NZ_AMZO01000034	Photobacterium marinum AK15	Bacteria	Proteobacteria	Gammaproteobacteria	Vibrionalles	Vibrionaceae	Photobacterium	H								
47394 -> 50144	+	WP_007469744.1	NZ_AMZO01000034	Photobacterium marinum AK15	Bacteria	Proteobacteria	Gammaproteobacteria	Vibrionalles	Vibrionaceae	Photobacterium	CRISPR-associated endonuclease Cas1	Cas6-RT-Cas1	mkCas0066, pfam0078, cd09634	CAS-I, CAS-III;CAS-III; CAS-I, CAS-II, CAS-III, CAS-V					
50146 -> 50436	+	WP_007469746.1	NZ_AMZO01000034	Photobacterium marinum AK15	Bacteria	Proteobacteria	Gammaproteobacteria	Vibrionalles	Vibrionaceae	Photobacterium	CRISPR-associated endonuclease Cas2	Cas2	pfam09827	CAS-I, CAS-II, CAS-III					
50678 -> 51619	+		NZ_AMZO01000034	Photobacterium marinum AK15	Bacteria	Proteobacteria	Gammaproteobacteria	Vibrionalles	Vibrionaceae	Photobacterium		CRISPR array			13 (33-37)	14 (35)	M14/NA/NA	GTTGCAGCATCATTTGCCGTTAAGGCAGTAGAAAC	M
CAS-III-A/CAS-III-D																			
872437->874104	+	WP_038188746.1	NZ_JRWP01000004	Vibrio sinaloensiss T08	Bacteria	Proteobacteria	Gammaproteobacteria	Vibrionalles	Vibrionaceae	Vibrio	H	Cas10	cls000707	CAS-III					
874107->876089	+	WP_038188749.1	NZ_JRWP01000004	Vibrio sinaloensiss T08	Bacteria	Proteobacteria	Gammaproteobacteria	Vibrionalles	Vibrionaceae	Vibrio	H	Csm4gr5	cls000259	CAS-III-D					
876082->877518	+	WP_038188753.1	NZ_JRWP01000004	Vibrio sinaloensiss T08	Bacteria	Proteobacteria	Gammaproteobacteria	Vibrionalles	Vibrionaceae	Vibrio	H	Csm3gr7	cd09683, cd09683	CAS-III-A, CAS-III-D; CAS-III-A, CAS-III-D					
877518->879848	+	WP_038188757.1	NZ_JRWP01000004	Vibrio sinaloensiss T08	Bacteria	Proteobacteria	Gammaproteobacteria	Vibrionalles	Vibrionaceae	Vibrio	CRISPR-associated RAMP family protein	Csm3gr7	cls000253, cls000253	CAS-III-A, CAS-III-D; CAS-III-A, CAS-III-D					
879859->882669	+	WP_038188758.1	NZ_JRWP01000004	Vibrio sinaloensiss T08	Bacteria	Proteobacteria	Gammaproteobacteria	Vibrionalles	Vibrionaceae	Vibrio	CRISPR-associated endonuclease Cas1	RT-Cas1	pfam00078, pfam01867	CAS-III; CAS-I, CAS-II, CAS-III					
882673->882951	+	WP_038188761.1	NZ_JRWP01000004	Vibrio sinaloensiss T08	Bacteria	Proteobacteria	Gammaproteobacteria	Vibrionalles	Vibrionaceae	Vibrio	CRISPR-associated endonuclease Cas2	Cas2	pfam09827	CAS-I, CAS-II, CAS-III					
883109 -> 885110	+		NZ_JRWP01000004	Vibrio sinaloensiss T08	Bacteria	Proteobacteria	Gammaproteobacteria	Vibrionalles	Vibrionaceae	Vibrio		CRISPR array			28 (33-37)	29 (35)	NA/NA/NA	GCTTCATTAGTACTGGTCGTTCAGACCGTTGAGAC	H
885179->885736	+	WP_038188763.1	NZ_JRWP01000004	Vibrio sinaloensiss T08	Bacteria	Proteobacteria	Gammaproteobacteria	Vibrionalles	Vibrionaceae	Vibrio	H								
885832->886983	+	WP_072016631.1	NZ_JRWP01000004	Vibrio sinaloensiss T08	Bacteria	Proteobacteria	Gammaproteobacteria	Vibrionalles	Vibrionaceae	Vibrio	H	Csx1	pfam09002	CAS-III					
CAS-III-D																			
255395-> 257047	+	WP_055043545.1	NZ_LBGR01000002	Vibrio metoecus YB9D03 YB09_D03	Bacteria	Proteobacteria	Gammaproteobacteria	Vibrionalles	Vibrionaceae	Vibrio	H	Cas10	cls000707	CAS-III					
257044-> 259020	+	WP_055043546.1	NZ_LBGR01000002	Vibrio metoecus YB9D03 YB09_D03	Bacteria	Proteobacteria	Gammaproteobacteria	Vibrionalles	Vibrionaceae	Vibrio	H	Csm4gr5	cls000259	CAS-III-D					
259013-> 260512	+	WP_055043547.1	NZ_LBGR01000002	Vibrio metoecus YB9D03 YB09_D03	Bacteria	Proteobacteria	Gammaproteobacteria	Vibrionalles	Vibrionaceae	Vibrio	H	Csm3gr7	cd09726, cd09683	CAS-III-D; CAS-III-A, CAS-III-D					
260512-> 262905	+	WP_055043548.1	NZ_LBGR01000002	Vibrio metoecus YB9D03 YB09_D03	Bacteria	Proteobacteria	Gammaproteobacteria	Vibrionalles	Vibrionaceae	Vibrio	CRISPR-associated RAMP family protein	Csm3gr7	cls000253, cls000253	CAS-III-A, CAS-III-D; CAS-III-A, CAS-III-D					
262914-> 265730	+	WP_055043549.1	NZ_LBGR01000002	Vibrio metoecus YB9D03 YB09_D03	Bacteria	Proteobacteria	Gammaproteobacteria	Vibrionalles	Vibrionaceae	Vibrio	CRISPR-associated endonuclease Cas1	Cas6-RT-Cas1	mkCas0091, pfam0078, pfam01867	CAS-I, CAS-III; RT; CAS-I, CAS-II, CAS-III					
265734-> 266012	+	WP_055043550.1	NZ_LBGR01000002	Vibrio metoecus YB9D03 YB09_D03	Bacteria	Proteobacteria	Gammaproteobacteria	Vibrionalles	Vibrionaceae	Vibrio	CRISPR-associated protein Cas2	Cas2	mkCas0081	CAS-I, CAS-II, CAS-III					
266170 -> 268208	+		NZ_LBGR01000002	Vibrio metoecus YB9D03 YB09_D03	Bacteria	Proteobacteria	Gammaproteobacteria	Vibrionalles	Vibrionaceae	Vibrio		CRISPR array			28 (33-41)	29 (35)	M14/NA/NA	GCTTCATTAGATAAGGTCGTTCAGACCGTTGAGAC	NA
CAS-III-D																			
37851->37466	-		NZ_LDOV01000030	Photobacterium aphoticum DSM 255995	Bacteria	Proteobacteria	Gammaproteobacteria	Vibrionalles	Vibrionaceae	Photobacterium		CRISPR array			5 (32-37)	6 (35)	M14/NA/NA	CTTTCAGTGCACTTGGCCGTTTAGGCCGTTGAAAC	M
38682->38086	-	WP_047875590.1	NZ_LDOV01000030	Photobacterium aphoticum DSM 255995	Bacteria	Proteobacteria	Gammaproteobacteria	Vibrionalles	Vibrionaceae	Photobacterium	H								
39474->38734	-		NZ_LDOV01000030	Photobacterium aphoticum DSM 255995	Bacteria	Proteobacteria	Gammaproteobacteria	Vibrionalles	Vibrionaceae	Photobacterium		CRISPR array			9 (34-38)	10 (35)	M14/NA/NA	CTTTCAGTGCGCTTGGCCGTTTAGGCCGTTGAAAC	NA
39939->39661	-	WP_047875591.1	NZ_LDOV01000030	Photobacterium aphoticum DSM 255995	Bacteria	Proteobacteria	Gammaproteobacteria	Vibrionalles	Vibrionaceae	Photobacterium	CRISPR-associated protein Cas2	Cas2	mkCas0081	CAS-I, CAS-II, CAS-III					
42836->39975	-	WP_047875592.1	NZ_LDOV01000030	Photobacterium aphoticum DSM 255995	Bacteria	Proteobacteria	Gammaproteobacteria	Vibrionalles	Vibrionaceae	Photobacterium	CRISPR-associated endonuclease Cas1	RT-Cas1	pfam0078, COG1518	CAS-III; CAS-I, CAS-II, CAS-III					
45263->42885	-	WP_047875593.1	NZ_LDOV01000030	Photobacterium aphoticum DSM 255995	Bacteria	Proteobacteria	Gammaproteobacteria	Vibrionalles	Vibrionaceae	Photobacterium	CRISPR-associated RAMP family protein	Csm3gr7	cls000253, cls000253	CAS-III-A, CAS-III-D					
46780->45260	-	WP_047875594.1	NZ_LDOV01000030	Photobacterium aphoticum DSM 255995	Bacteria	Proteobacteria	Gammaproteobacteria	Vibrionalles	Vibrionaceae	Photobacterium	H	Csm3gr7	cd09683, cd09683	CAS-III-A, CAS-III-D; CAS-III-A, CAS-IIID					
48752->46791	-	WP_047875595.1	NZ_LDOV01000030	Photobacterium aphoticum DSM 255995	Bacteria	Proteobacteria	Gammaproteobacteria	Vibrionalles	Vibrionaceae	Photobacterium	H	Csm3gr7	cls000708	CAS-III-D					
50443->48749	-	WP_047875596.1	NZ_LDOV01000030	Photobacterium aphoticum DSM 255995	Bacteria	Proteobacteria	Gammaproteobacteria	Vibrionalles	Vibrionaceae	Photobacterium	H	Cas10	cls000707	CAS-III					
CAS-III-B																			
698099->694195	-		CP002583	Marinomonas mediterranea MMB-1	Bacteria	Proteobacteria	Gammaproteobacteria	Oceanospirillales	Oceanospirilaceae	Marinomonas		CRISPR array			55 (24-42)	56 (35)	NA/NA/NA	GTTTCAGACCCGCTGGCCGCTTAGGCCGTTGAGAC	M

698600 -> 698322	-	ADZ89952.1	CP002583	Marinomonas mediterranea MMB-1	Bacteria	Proteobacteria	Gammaproteobacteria	Oceanospirillales	Oceanospirilaceae	Marinomonas	CRISPR-associated protein Cas2	Cas2	mkCas0081	CAS-I, CAS-II, CAS-III					
701470 -> 698597	-	ADZ89953.1	CP002583	Marinomonas mediterranea MMB-1	Bacteria	Proteobacteria	Gammaproteobacteria	Oceanospirillales	Oceanospirilaceae	Marinomonas	CRISPR-associated protein Cas1	Cas6-RT-Cas1	mkCas0091, pfam0078, pfam01867	CAS-I, CAS-III; RT; CAS-I, CAS-II, CAS-III					
702249 -> 701479	-	ADZ89954.1	CP002583	Marinomonas mediterranea MMB-1	Bacteria	Proteobacteria	Gammaproteobacteria	Oceanospirillales	Oceanospirilaceae	Marinomonas	H	PD-DExK	mkCas0090	CAS-I, CAS-III					
702859 -> 702290	-	ADZ89955.1	CP002583	Marinomonas mediterranea MMB-1	Bacteria	Proteobacteria	Gammaproteobacteria	Oceanospirillales	Oceanospirilaceae	Marinomonas	H								
704075 -> 702960	-	ADZ89956.1	CP002583	Marinomonas mediterranea MMB-1	Bacteria	Proteobacteria	Gammaproteobacteria	Oceanospirillales	Oceanospirilaceae	Marinomonas	CRISPR-associated RAMP protein, Cmr6 family	Cmr6gr7	COG1604	CAS-III-B					
704497 -> 704072	-	ADZ89957.1	CP002583	Marinomonas mediterranea MMB-1	Bacteria	Proteobacteria	Gammaproteobacteria	Oceanospirillales	Oceanospirilaceae	Marinomonas	CRISPR-associated protein, Cmr5 family	Cmr5gr11	cd09749	CAS-III-B					
705396 -> 704494	-	ADZ89958.1	CP002583	Marinomonas mediterranea MMB-1	Bacteria	Proteobacteria	Gammaproteobacteria	Oceanospirillales	Oceanospirilaceae	Marinomonas	CRISPR-associated RAMP protein, Cmr4 family	Cmr4gr7	COG1336	CAS-III-C					
706613 -> 705396	-	ADZ89959.1	CP002583	Marinomonas mediterranea MMB-1	Bacteria	Proteobacteria	Gammaproteobacteria	Oceanospirillales	Oceanospirilaceae	Marinomonas	CRISPR-associated protein, Cmr3 family	Cmr3gr5	cd09748	CAS-III-B					
708520 -> 706613	-	ADZ89960.1	CP002583	Marinomonas mediterranea MMB-1	Bacteria	Proteobacteria	Gammaproteobacteria	Oceanospirillales	Oceanospirilaceae	Marinomonas	CRISPR-associated protein, Cmr2 family	Cas10	pfam12469, cd09679	CAS-IIIB; CAS-III					
709794 -> 708523	-	ADZ89961.1	CP002583	Marinomonas mediterranea MMB-1	Bacteria	Proteobacteria	Gammaproteobacteria	Oceanospirillales	Oceanospirilaceae	Marinomonas	CRISPR-associated protein TM1795 family protein	Cmr1gr7	COG1367	CAS-III-B					
710944->710355	-		CP002583	Marinomonas mediterranea MMB-1	Bacteria	Proteobacteria	Gammaproteobacteria	Oceanospirillales	Oceanospirilaceae	Marinomonas		CRISPR array			8 (26-38)	9 (35)	NA/NA/NA	GTTTCAGACCCGCTGGCCGCTTAGGCCGTTGAGAC	NA
CAS-III-B																			
1580987->1580709	-	WP_028883448.1	NZ_KK211138	Teredinibacter turnerae 1133Y.S.0a.04	Bacteria	Proteobacteria	Gammaproteobacteria	Cellvibrionales	C ellvibrionaceae	Teredinibacter	CRISPR-associated endonuclease Cas2	Cas2	mkCas0081	CAS-I, CAS-II, CAS-III					
1583888->1580991	-	WP_028883449.1	NZ_KK211138	Teredinibacter turnerae 1133Y.S.0a.04	Bacteria	Proteobacteria	Gammaproteobacteria	Cellvibrionales	C ellvibrionaceae	Teredinibacter	CRISPR-associated endonuclease Cas1	RT-Cas1	pfam0078, pfam01867	CAS-III; CAS-I, CAS-II, CAS-III					
1585036->1583888	-	WP_038010205.1	NZ_KK211138	Teredinibacter turnerae 1133Y.S.0a.04	Bacteria	Proteobacteria	Gammaproteobacteria	Cellvibrionales	C ellvibrionaceae	Teredinibacter	type III-B CRISPR module RAMP protein Cmr6	Cmr6gr7	COG1604	CAS-III-B					
1585437->1585033	-	WP_028883450.1	NZ_KK211138	Teredinibacter turnerae 1133Y.S.0a.04	Bacteria	Proteobacteria	Gammaproteobacteria	Cellvibrionales	C ellvibrionaceae	Teredinibacter	type III-B CRISPR module-associated protein Cmr5	Cmr5gr11	cd09654	CAS-III-B					
1586289->1585447	-	WP_028883451.1	NZ_KK211138	Teredinibacter turnerae 1133Y.S.0a.04	Bacteria	Proteobacteria	Gammaproteobacteria	Cellvibrionales	C ellvibrionaceae	Teredinibacter	type III-B CRISPR module RAMP protein Cmr4	Cmr4gr7	COG1336	CAS-III-C					
1587448->1586306	-	WP_028883452.1	NZ_KK211138	Teredinibacter turnerae 1133Y.S.0a.04	Bacteria	Proteobacteria	Gammaproteobacteria	Cellvibrionales	C ellvibrionaceae	Teredinibacter	H	Cmr3gr5	cd09748	CAS-III-B					
1589397->1587451	-	WP_051529229.1	NZ_KK211138	Teredinibacter turnerae 1133Y.S.0a.04	Bacteria	Proteobacteria	Gammaproteobacteria	Cellvibrionales	C ellvibrionaceae	Teredinibacter	type III-B CRISPR-associated protein Cas10/Cmr2	Cas10	cd9679, cd9679	CAS-III; CAS-III					
1590680->1589394	-	WP_028883453.1	NZ_KK211138	Teredinibacter turnerae 1133Y.S.0a.04	Bacteria	Proteobacteria	Gammaproteobacteria	Cellvibrionales	C ellvibrionaceae	Teredinibacter	type III-B CRISPR module RAMP protein Cmr1	Cmr1gr7	COG1367	CAS-III-B					

RT-CRISPR-8b (CAS-III-A/D, CAS-III-B/C)
Plantomycetia; Deltaproteobacteria (Desulfovibrionales, Syntrophobacterales); Betaproteobacteria; Gammaproteobacteria (Pseudomonadales

[a](Sub) Type/[b]Coordinates	Strand	Protein_id	Genome Partition ID	Strain	Domain	Phylum	Lineage (class)	Order	Family	Genus	[c]Cas gene (annotated)	[d]Cas gene/[e]Array (identified)	[d]Profiles	[d](Sub)Type-specificity	[e]Spacers (length)	[e]Repeats (length)	[f]Str.motif/Seq.family/Superclass	[f]Seq Repeats	[g]Orientation Confidence
Unknown																			
2130 -> 2333	+	KKO19836.1	LAQJ01000147.1	Candidatus Brocadia fulgida RU1 BROFUL	Bacteria	Plantomycetes	Plantomycetia	Candidatus Brocadiales	Candidatus Brocadiaceae	Candidatus Brocadia	CRISPR-associated protein	Cas2	mkCas0081	CAS-I, CAS-II, CAS-III					
2814 -> 3380	+	KKO19837.1	LAQJ01000147.1	Candidatus Brocadia fulgida RU1 BROFUL	Bacteria	Plantomycetes	Plantomycetia	Candidatus Brocadiales	Candidatus Brocadiaceae	Candidatus Brocadia	H								
3721 -> 5787	+	KKO19838.1	LAQJ01000147.1	Candidatus Brocadia fulgida RU1 BROFUL	Bacteria	Plantomycetes	Plantomycetia	Candidatus Brocadiales	Candidatus Brocadiaceae	Candidatus Brocadia	H	RT-Cas1	pfam00078, pfam01867	CAS-III; CAS-I, CAS-II, CAS-III					
Partial CAS-III-C																			
3962710 -> 3962153	-	WP_052565449.1	NZ_BAFN01000001	Candidatus Brocadia sinica JPN1	Bacteria	Plantomycetes	Plantomycetia	Candidatus Brocadiales	Candidatus Brocadiaceae	Candidatus Brocadia	CRISPR-associated endonuclease Cas1	Cas1	COG1518	CAS-I, CAS-II, CAS-III					
3963866 -> 3962874	-	WP_052565451.1	NZ_BAFN01000001	Candidatus Brocadia sinica JPN1	Bacteria	Plantomycetes	Plantomycetia	Candidatus Brocadiales	Candidatus Brocadiaceae	Candidatus Brocadia	H	RT-Cas1 (truncated)	pfam00078, pfam01867	CAS-III					
3964912 -> 3963890	-	WP_052565453.1	NZ_BAFN01000001	Candidatus Brocadia sinica JPN1	Bacteria	Plantomycetes	Plantomycetia	Candidatus Brocadiales	Candidatus Brocadiaceae	Candidatus Brocadia	H	Cas6	mKCas0066	CAS-I, CAS-III					
3965437 -> 3965249	-	WP_052565456.1	NZ_BAFN01000001	Candidatus Brocadia sinica JPN1	Bacteria	Plantomycetes	Plantomycetia	Candidatus Brocadiales	Candidatus Brocadiaceae	Candidatus Brocadia	H								
3965607 -> 3965422	-	WP_052565458.1	NZ_BAFN01000001	Candidatus Brocadia sinica JPN1	Bacteria	Plantomycetes	Plantomycetia	Candidatus Brocadiales	Candidatus Brocadiaceae	Candidatus Brocadia	H								
3966828 -> 3966445	-	WP_052565459.1	NZ_BAFN01000001	Candidatus Brocadia sinica JPN1	Bacteria	Plantomycetes	Plantomycetia	Candidatus Brocadiales	Candidatus Brocadiaceae	Candidatus Brocadia	four helix bundle protein								
3967420 -> 3967136	-	WP_052565460.1	NZ_BAFN01000001	Candidatus Brocadia sinica JPN1	Bacteria	Plantomycetes	Plantomycetia	Candidatus Brocadiales	Candidatus Brocadiaceae	Candidatus Brocadia	CRISPR-associated endonuclease Cas2	Cas2	mkCas0081	CAS-I, CAS-II, CAS-III					
3968348 -> 3967680	-	WP_052565461.1	NZ_BAFN01000001	Candidatus Brocadia sinica JPN1	Bacteria	Plantomycetes	Plantomycetia	Candidatus Brocadiales	Candidatus Brocadiaceae	Candidatus Brocadia	type III-B CRISPR module RAMP protein Cmr1	Cmr1gr7	cd09657	CAS-III-C					
Unknown																			
1 -> 1685	+	KFZ44108.1	JQOA01000247	Smithella sp. D17	Bacteria	Proteobacteria	Deltaproteobacteria	Syntrophobacterales	Syntrophacea	Smithella	H	RT(partial)-Cas1	pfam00078, pfam01867	CAS-III; CAS-I, CAS-II, CAS-III					
1673 -> 1966	+	KFZ44109.1	JQOA01000247	Smithella sp. D17	Bacteria	Proteobacteria	Deltaproteobacteria	Syntrophobacterales	Syntrophacea	Smithella	H	Cas2	mkCas0081	CAS-I, CAS-II, CAS-III					
2073 -> 2740	+		JQOA01000247	Smithella sp. D17	Bacteria	Proteobacteria	Deltaproteobacteria	Syntrophobacterales	Syntrophacea	Smithella		CRISPR Array			9 (30-52)	10 (36)	M16/NA/E	GTCTGAATAGACAACCGTAGGACTACGGTCTGAAAC	L
Partial CAS-III-A/CAS-III-D																			
334->2358	+	KFB71599.1	JDVG02000521	Candidatus Accumulibacter sp. BA-91	Bacteria	Proteobacteria	Betaproteobacteria	Unclassified			CRISPR-associated RAMP protein, Csx10 family	Csm4gr5	cls000259						
2355->3719	+	KFB71598.1	JDVG02000521	Candidatus Accumulibacter sp. BA-91	Bacteria	Proteobacteria	Betaproteobacteria	Unclassified			CRISPR-associated RAMP protein, family	Csm3gr7	COG1337, COG1337	CAS-III-D; CAS-III-D					
3716->4201	+	KFB71597.1	JDVG02000521	Candidatus Accumulibacter sp. BA-91	Bacteria	Proteobacteria	Betaproteobacteria	Unclassified			H								
4198->6324	+	KFB71596.1	JDVG02000521	Candidatus Accumulibacter sp. BA-91	Bacteria	Proteobacteria	Betaproteobacteria	Unclassified			CRISPR-associated protein	Csm3gr7	cls000253	CAS-III-A, CAS-III-D					
6349->8013	+	KFB71595.1	JDVG02000521	Candidatus Accumulibacter sp. BA-91	Bacteria	Proteobacteria	Betaproteobacteria	Unclassified			H	Csx19	cls000059						
8026->10668	+	KFB71594.1	JDVG02000521	Candidatus Accumulibacter sp. BA-91	Bacteria	Proteobacteria	Betaproteobacteria	Unclassified			cas1	Cas6-RT-Cas1	mkCas0066, pfam00078, pfam01867	RT; CAS-I, CAS-II, CAS-III					
10661->10942	+	KFB71593.1	JDVG02000521	Candidatus Accumulibacter sp. BA-91	Bacteria	Proteobacteria	Betaproteobacteria	Unclassified			CRISPR-associated endoribonuclease Cas2	Cas2	mkCas0081	CAS-I, CAS-II, CAS-III					
11050 -> 11416	+		JDVG02000521	Candidatus Accumulibacter sp. BA-91	Bacteria	Proteobacteria	Betaproteobacteria	Unclassified				CRISPR Array			5 (29-32)	6 (36)	M3/NA/F	GCTGAATAGGGAATCCTGTTCGAACAGGCTTGAAAC	L
CAS-III-B																			
23185 -> 24384	+	WP_019672857.1	NZ_KB907634.1	Psychrobacter lutiphocae DSM 21542	Bacteria	Proteobacteria	Gammaproteobacteria	Pseudomonadales	Moraxellaceae	Psychrobacter	H	Csx1	pfam09002	CAS-III					
24503 -> 25636	+	WP_019672858.1	NZ_KB907634.1	Psychrobacter lutiphocae DSM 21542	Bacteria	Proteobacteria	Gammaproteobacteria	Pseudomonadales	Moraxellaceae	Psychrobacter	CRISPR-associated protein	Csx1	pfam09623	CAS-III					
26113 -> 27300	+	WP_019672859.1	NZ_KB907634.1	Psychrobacter lutiphocae DSM 21542	Bacteria	Proteobacteria	Gammaproteobacteria	Pseudomonadales	Moraxellaceae	Psychrobacter	type III-B CRISPR module RAMP protein Cmr1	Cmr1gr7	COG1367	CAS-III-B					
27300 -> 29390	+	WP_019672860.1	NZ_KB907634.1	Psychrobacter lutiphocae DSM 21542	Bacteria	Proteobacteria	Gammaproteobacteria	Pseudomonadales	Moraxellaceae	Psychrobacter	type III-B CRISPR.associated protein Cas10_Cmr2	Cas10	pfam12469, cd09679	CAS-III-B; CAS-III					
29390 -> 30670	+	WP_019672861.1	NZ_KB907634.1	Psychrobacter lutiphocae DSM 21542	Bacteria	Proteobacteria	Gammaproteobacteria	Pseudomonadales	Moraxellaceae	Psychrobacter	H	Cmr3gr5	cd09748	CAS-III-B					
30732 -> 31643	+	WP_019672862.1	NZ_KB907634.1	Psychrobacter lutiphocae DSM 21542	Bacteria	Proteobacteria	Gammaproteobacteria	Pseudomonadales	Moraxellaceae	Psychrobacter	type III-B CRISPR module RAMP protein Cmr6	Cmr6gr7	cd09661	CAS-III-B					
31670 -> 32074	-	WP_019672863.1	NZ_KB907634.1	Psychrobacter lutiphocae DSM 21542	Bacteria	Proteobacteria	Gammaproteobacteria	Pseudomonadales	Moraxellaceae	Psychrobacter	type III-B CRISPR module RAMP protein Cmr4	Cmr4gr7	COG1336	CAS-III-C					
32111 -> 33397	+	WP_019672864.1	NZ_KB907634.1	Psychrobacter lutiphocae DSM 21542	Bacteria	Proteobacteria	Gammaproteobacteria	Pseudomonadales	Moraxellaceae	Psychrobacter	type III-B CRISPR module-associated protein Cmr5	Cmr5gr11	cd09749	CAS-III-B					
33720 -> 33941	+	WP_019672866.1	NZ_KB907634.1	Psychrobacter lutiphocae DSM 21542	Bacteria	Proteobacteria	Gammaproteobacteria	Pseudomonadales	Moraxellaceae	Psychrobacter	H								
34281 -> 33994	-	WP_019672867.1	NZ_KB907634.1	Psychrobacter lutiphocae DSM 21542	Bacteria	Proteobacteria	Gammaproteobacteria	Pseudomonadales	Moraxellaceae	Psychrobacter	H								
36157 -> 37338	+	WP_019672868.1	NZ_KB907634.1	Psychrobacter lutiphocae DSM 21542	Bacteria	Proteobacteria	Gammaproteobacteria	Pseudomonadales	Moraxellaceae	Psychrobacter	CRISPR-associated protein	Csx1	cd09668	CAS-III					
37781 -> 38022	+			Psychrobacter lutiphocae DSM 21542	Bacteria	Proteobacteria	Gammaproteobacteria	Pseudomonadales	Moraxellaceae	Psychrobacter		CRISPR Array (assembly gap)			>5 (31-35)	>6 (36)	M15/NA/F	GTTCTGCACTATGCAGAGGTACGTACCGATTAAGAC	NA
38411 -> 38722	+	WP_019672869.1	NZ_KB907634.1	Psychrobacter lutiphocae DSM 21542	Bacteria	Proteobacteria	Gammaproteobacteria	Pseudomonadales	Moraxellaceae	Psychrobacter	putative CRISPR-associated protein	Csx16	cd09743	CAS-I, CAS-III					
38731 -> 41739	+	WP_019672870.1	NZ_KB907634.1	Psychrobacter lutiphocae DSM 21542	Bacteria	Proteobacteria	Gammaproteobacteria	Pseudomonadales	Moraxellaceae	Psychrobacter	CRISPR-associated endonuclease Cas1	RT-Cas1	pfam00078; COG1518	CAS-III; CAS-I, CAS-II, CAS-III					
41783 -> 42076	+	WP_019672871.1	NZ_KB907634.1	Psychrobacter lutiphocae DSM 21542	Bacteria	Proteobacteria	Gammaproteobacteria	Pseudomonadales	Moraxellaceae	Psychrobacter	CRISPR-associated endonuclease Cas2	Cas2	mkCas0081	CAS-I, CAS-II, CAS-III					
42249 -> 42695	+			Psychrobacter lutiphocae DSM 21542	Bacteria	Proteobacteria	Gammaproteobacteria	Pseudomonadales	Moraxellaceae	Psychrobacter		CRISPR Array			6 (31-35)	7 (36)	M15/NA/F	GTTCTGCACTATGCAGAGGTACGTACCGATTAAGAC	M
CAS-III-B																			
1662-> 2894	+	NZ_KE387016.1	WP_027180396.1	Desulfovibrio bastinii DSM 16055	Bacteria	Proteobacteria	Deltaproteobacteria	Desulfovibrionales	Desulfovibrionaceae	Desulfovibrio	type III-B CRISPR module RAMP protein Cmr1	Cmr1gr7	COG1367	CAS-III-B					
2891-> 4675	+	NZ_KE387016.1	WP_027180397.1	Desulfovibrio bastinii DSM 16055	Bacteria	Proteobacteria	Deltaproteobacteria	Desulfovibrionales	Desulfovibrionaceae	Desulfovibrio	type III-B CRISPR-associated protein Cas10_Cmr2	Cas10	cls000742	CAS-III					
4668-> 5888	+	NZ_KE387016.1	WP_027180398.1	Desulfovibrio bastinii DSM 16055	Bacteria	Proteobacteria	Deltaproteobacteria	Desulfovibrionales	Desulfovibrionaceae	Desulfovibrio	H	Cmr3gr5	cd09748	CAS-III-B					
5892-> 6827	+	NZ_KE387016.1	WP_027180399.1	Desulfovibrio bastinii DSM 16055	Bacteria	Proteobacteria	Deltaproteobacteria	Desulfovibrionales	Desulfovibrionaceae	Desulfovibrio	type III-B CRISPR module RAMP protein Cmr4	Cmr4gr7	COG1336	CAS-III-C					
6820-> 7218	+	NZ_KE387016.1	WP_027180400.1	Desulfovibrio bastinii DSM 16055	Bacteria	Proteobacteria	Deltaproteobacteria	Desulfovibrionales	Desulfovibrionaceae	Desulfovibrio	type III-B CRISPR module-associated protein Cmr5	Cmr5gr11	pfam09701	CAS-III-B					
7222-> 8628	+	NZ_KE387016.1	WP_027180401.1	Desulfovibrio bastinii DSM 16055	Bacteria	Proteobacteria	Deltaproteobacteria	Desulfovibrionales	Desulfovibrionaceae	Desulfovibrio	type III-B CRISPR module RAMP protein Cmr6	Cmr6gr7	cd09661	CAS-III-B					
8935 -> 9252	+			Desulfovibrio bastinii DSM 16055	Bacteria	Proteobacteria	Deltaproteobacteria	Desulfovibrionales	Desulfovibrionaceae	Desulfovibrio		CRISPR Array			4 (34-35)	5 (36)	M14/NA/E	CTCAAAAGAGGTTTCCGATCTGGATCGGGTTTTGAC	NA
9377 -> 9655	+	NZ_KE387016.1	WP_034633809.1	Desulfovibrio bastinii DSM 16055	Bacteria	Proteobacteria	Deltaproteobacteria	Desulfovibrionales	Desulfovibrionaceae	Desulfovibrio	CRISPR-associated endonuclease Cas2	Cas2	pfam09827	CAS-I, CAS-II, CAS-III					
9655-> 12279	+	NZ_KE387016.1	WP_027180402.1	Desulfovibrio bastinii DSM 16055	Bacteria	Proteobacteria	Deltaproteobacteria	Desulfovibrionales	Desulfovibrionaceae	Desulfovibrio	CRISPR-associated endonuclease Cas1	Cas6-RT-Cas1	mkCas0091; pfam00078; COG1518	CAS-III; CAS-I, CAS-II, CAS-III					
12519 -> 12835	+			Desulfovibrio bastinii DSM 16055	Bacteria	Proteobacteria	Deltaproteobacteria	Desulfovibrionales	Desulfovibrionaceae	Desulfovibrio		CRISPR Array			4 (33-35)	5 (36)	M14/NA/E	CTCAAAAGAGGTTTCCGATCTGGATCGGGTTTTGAC	NA
13563-> 12892	-	NZ_KE387016.1	WP_027180403.1	Desulfovibrio bastinii DSM 16055	Bacteria	Proteobacteria	Deltaproteobacteria	Desulfovibrionales	Desulfovibrionaceae	Desulfovibrio	H								
13824-> 13576	-	NZ_KE387016.1	WP_027180404.1	Desulfovibrio bastinii DSM 16055	Bacteria	Proteobacteria	Deltaproteobacteria	Desulfovibrionales	Desulfovibrionaceae	Desulfovibrio	H	Csx3	mkCas0115	CAS-I, CAS-III					
CAS-III-B																			
24678->24430	-			Desulfovibrio hydrothermalis AM13	Bacteria	Proteobacteria	Deltaproteobacteria	Desulfovibrionales	Desulfovibrionaceae	Desulfovibrio		CRISPR Array			3 (34-36)	4 (36)	M14/NA/E	CTCAAAAGAGGTATCCATATCGGTATGGGTTTTGAC	M
27546-> 24919	-	NC_020055	WP_015334627.1	Desulfovibrio hydrothermalis AM13	Bacteria	Proteobacteria	Deltaproteobacteria	Desulfovibrionales	Desulfovibrionaceae	Desulfovibrio	CRISPR-associated endonuclease Cas1	Cas6-RT-Cas1	mkCas0091; pfam00078; cd09721	CAS-III, CAS-I-C					
27828-> 27550	-	NC_020055	WP_034624493.1	Desulfovibrio hydrothermalis AM13	Bacteria	Proteobacteria	Deltaproteobacteria	Desulfovibrionales	Desulfovibrionaceae	Desulfovibrio	CRISPR-associated endonuclease Cas2	Cas2	cd09725	CAS-I, CAS-II, CAS-III, CAS-V					
27885 ->28051	+			Desulfovibrio hydrothermalis AM13	Bacteria	Proteobacteria	Deltaproteobacteria	Desulfovibrionales	Desulfovibrionaceae	Desulfovibrio		CRISPR Array			2 (41,45)	3 (28)	M3/NA/NA	CCATACCGATATGGATACCTCTTTTGAG	M
29566-> 28541	-	NC_020055	WP_015334631.1	Desulfovibrio hydrothermalis AM13	Bacteria	Proteobacteria	Deltaproteobacteria	Desulfovibrionales	Desulfovibrionaceae	Desulfovibrio	type III-B CRISPR module RAMP protein Cmr6	Cmr6gr7	cd09661	CAS-III-B					
29981-> 29556	-	NC_020055	WP_015334632.1	Desulfovibrio hydrothermalis AM13	Bacteria	Proteobacteria	Deltaproteobacteria	Desulfovibrionales	Desulfovibrionaceae	Desulfovibrio	type III-B CRISPR module-associated protein Cmr5	Cmr5gr11	pfam09701	CAS-III-B					
30819-> 29974	-	NC_020055	WP_015334633.1	Desulfovibrio hydrothermalis AM13	Bacteria	Proteobacteria	Deltaproteobacteria	Desulfovibrionales	Desulfovibrionaceae	Desulfovibrio	type III-B CRISPR module RAMP protein Cmr4	Cmr4gr7	COG1336	CAS-III-C					
32008-> 30809	-	NC_020055	WP_015334634.1	Desulfovibrio hydrothermalis AM13	Bacteria	Proteobacteria	Deltaproteobacteria	Desulfovibrionales	Desulfovibrionaceae	Desulfovibrio	H	Cmr3gr5	cd09748	CAS-III-B					
34026-> 31993	-	NC_020055	WP_015334635.1	Desulfovibrio hydrothermalis AM13	Bacteria	Proteobacteria	Deltaproteobacteria	Desulfovibrionales	Desulfovibrionaceae	Desulfovibrio	type III-B CRISPR-associated protein Cas10_Cmr2	Cas10	pfam12469, cls000742	CAS-III-B, CAS-III					
35033-> 34026	-	NC_020055	WP_015334636.1	Desulfovibrio hydrothermalis AM13	Bacteria	Proteobacteria	Deltaproteobacteria	Desulfovibrionales	Desulfovibrionaceae	Desulfovibrio	type III-B CRISPR module RAMP protein Cmr1	Cmr1gr7	COG1367	CAS-III-B					

RT-CRISPR-8 OTHERS (CAS-III-A/D)
Nitrospira (Nitrospirales); Plantomycetia (Plantomycetales)

Partial CAS-III-A/CAS-III-D																			
166->1794	+	JZJI01001005.1	KJR40062.1	Candidatus Magnetoovum chiemensis CS-04 SAG2981	Bacteria	Nitrospirae	Nitrospira	Nitrospirales	Nitrospiraceae		H	Cas10	mkCas0133	CAS-III					
1784->4222	+	JZJI01001005.1	KJR40061.1	Candidatus Magnetoovum chiemensis CS-04 SAG2981	Bacteria	Nitrospirae	Nitrospira	Nitrospirales	Nitrospiraceae		protein of unknown function DUF324								
4219->5550	+	JZJI01001005.1	KJR40060.1	Candidatus Magnetoovum chiemensis CS-04 SAG2981	Bacteria	Nitrospirae	Nitrospira	Nitrospirales	Nitrospiraceae		H	Csm3gr7	pfam03787						
5540->6043	+	JZJI01001005.1	KJR40059.1	Candidatus Magnetoovum chiemensis CS-04 SAG2981	Bacteria	Nitrospirae	Nitrospira	Nitrospirales	Nitrospiraceae		H								
6061->8475	+	JZJI01001005.1	KJR40058.1	Candidatus Magnetoovum chiemensis CS-04 SAG2981	Bacteria	Nitrospirae	Nitrospira	Nitrospirales	Nitrospiraceae		H	Csm3gr7	cls000253	CAS-III-A, CAS-III-D					
8484->11276	+	JZJI01001005.1	KJR40057.1	Candidatus Magnetoovum chiemensis CS-04 SAG2981	Bacteria	Nitrospirae	Nitrospira	Nitrospirales	Nitrospiraceae		CRISPR-associated protein Cas1	Cas6-RT-Cas1	mkCas0091; pfam00078; pfam01867	CAS-I, CAS-III; CAS-III; CAS-I, CAS-II, CAS-III					
11279->11542	+	JZJI01001005.1	KJR40056.1	Candidatus Magnetoovum chiemensis CS-04 SAG2981	Bacteria	Nitrospirae	Nitrospira	Nitrospirales	Nitrospiraceae		CRISPR-associated Cas2 family protein	Cas2	pfam09827	CAS-I, CAS-II, CAS-III					
CAS-III-D																			
1403843 -> 1405020	+			Pirellula staleyi DSM 6068	Bacteria	Plantomycetes	Plantomycetia	Plantomycetales	Plantomycetaceae	Pirellula		CRISPR Array			16 (33-40)	17 (35)	NA/NA/F	GTGTGAGAAAGAGGCCCTTCGGGGCCGATTGAGAC	H
1405107->1407935	+	NC_013720.1	WP_012910084.1	Pirellula staleyi DSM 6068	Bacteria	Plantomycetes	Plantomycetia	Plantomycetales	Plantomycetaceae	Pirellula	CRISPR-associated protein Cas1	Cas6-RT-Cas1	mkCas0066; pfam00078; pfam01867	CAS-I, CAS-III; CAS-III; CAS-I, CAS-II, CAS-III					
1407932->1408204	+	NC_013720.1	WP_012910085.1	Pirellula staleyi DSM 6068	Bacteria	Plantomycetes	Plantomycetia	Plantomycetales	Plantomycetaceae	Pirellula	CRISPR-associated endonuclease Cas2	Cas2	pfam09827	CAS-I, CAS-II, CAS-III					
1408802 -> 1409837	+			Pirellula staleyi DSM 6068	Bacteria	Plantomycetes	Plantomycetia	Plantomycetales	Plantomycetaceae	Pirellula		CRISPR Array			14 (34-40)	15 (35)	NA/NA/F	GTGTGAGAAAGAGGCCCTTCGGGGCCGATTGAGAC	H
1410068->1411780	+	NC_013720.1	WP_012910086.1	Pirellula staleyi DSM 6068	Bacteria	Plantomycetes	Plantomycetia	Plantomycetales	Plantomycetaceae	Pirellula	H								
1411810->1413309	+	NC_013720.1	WP_012910087.1	Pirellula staleyi DSM 6068	Bacteria	Plantomycetes	Plantomycetia	Plantomycetales	Plantomycetaceae	Pirellula	H	Cas10	cls000707	CAS-III					
1413306->1415291	+	NC_013720.1	WP_012910088.1	Pirellula staleyi DSM 6068	Bacteria	Plantomycetes	Plantomycetia	Plantomycetales	Plantomycetaceae	Pirellula	H	Csm4gr5	cls000259	CAS-III-D					
1415288->1419106	+	NC_013720.1	WP_012910089.1	Pirellula staleyi DSM 6068	Bacteria	Plantomycetes	Plantomycetia	Plantomycetales	Plantomycetaceae	Pirellula	CRISPR-associated RAMP family protein	Csm3gr7	cd09683, cd09683, cls000253	CAS-III-A, CAS-IIID; CAS-III-A, CAS-III-D; CAS-IIIA, CAS-III-D					

RT-CRISPR-9 (CAS-III)
Chloroflexia (Chloroflexales)

[a](Sub) Type/[b]Coordinates	Strand	Protein_id	Genome Partition ID	Strain	Domain	Phylum	Lineage (class)	Order	Family	Genus	[c]Cas gene (annotated)	[d]Cas gene/[e]Array (identified)	[d]Profiles	[d](Sub)Type-specificity	[e]Spacers (length)	[e]Repeats (length)	[f]Str.motif/Seq.family/Superclass	[f]Seq Repeats	[g]Orientation Confidence
Partial CAS-III																			
3786321->3781897	-		NC_009523.1	Roseiflexus sp. RS-1	Bacteria	Chloroflexi	Chloroflexia	Chloroflexales	Roseiflexineae	Roseiflexaceae	repeat region	CRISPR Array			59 (34-40)	60 (37)	M16/NA/E	GAAGCGACCACTGCCCCGCAATGAAGGGGATTGAAAC	H
3786481 ->3788568	+	WP_011957717.1	NC_009523.1	Roseiflexus sp. RS-1	Bacteria	Chloroflexi	Chloroflexia	Chloroflexales	Roseiflexineae	Roseiflexaceae	WYL domain-containing protein	WYL	pfam13280	CAS-I, CAS-III					
3788758 ->3788537	-	WP_049767605.1	NC_009523.1	Roseiflexus sp. RS-1	Bacteria	Chloroflexi	Chloroflexia	Chloroflexales	Roseiflexineae	Roseiflexaceae	CRISPR-associated protein Cas2	Cas2	mkCas0081	CAS-I, CAS-II, CAS-III					
3791269 ->3788945	-	WP_011957719.1	NC_009523.1	Roseiflexus sp. RS-1	Bacteria	Chloroflexi	Chloroflexia	Chloroflexales	Roseiflexineae	Roseiflexaceae	VWA domain-containing protein								
3792389 ->3791391	-	WP_011957720.1	NC_009523.1	Roseiflexus sp. RS-1	Bacteria	Chloroflexi	Chloroflexia	Chloroflexales	Roseiflexineae	Roseiflexaceae	CRISPR-associated endonuclease Cas1	Cas1	pfam01867	CAS-I, CAS-II, CAS-III					
3794231 ->3792897	-	WP_011957721.1	NC_009523.1	Roseiflexus sp. RS-1	Bacteria	Chloroflexi	Chloroflexia	Chloroflexales	Roseiflexineae	Roseiflexaceae	RNA-directed DNA polymerase	RT	pfam00078	CAS-III					
3794728 ->3794264	-	WP_011957722.1	NC_009523.1	Roseiflexus sp. RS-1	Bacteria	Chloroflexi	Chloroflexia	Chloroflexales	Roseiflexineae	Roseiflexaceae	H								
3796078 ->3795557	-	WP_011957724.1	NC_009523.1	Roseiflexus sp. RS-1	Bacteria	Chloroflexi	Chloroflexia	Chloroflexales	Roseiflexineae	Roseiflexaceae	nucleic acid-binding protein								
3796377 ->3796075	-	WP_011957725.1	NC_009523.1	Roseiflexus sp. RS-1	Bacteria	Chloroflexi	Chloroflexia	Chloroflexales	Roseiflexineae	Roseiflexaceae	H								
3796717 ->3796418	-	WP_041333819.1	NC_009523.1	Roseiflexus sp. RS-1	Bacteria	Chloroflexi	Chloroflexia	Chloroflexales	Roseiflexineae	Roseiflexaceae	H								
3797595 ->3797218	-	WP_011957726.1	NC_009523.1	Roseiflexus sp. RS-1	Bacteria	Chloroflexi	Chloroflexia	Chloroflexales	Roseiflexineae	Roseiflexaceae	H	Csx15	cd09766	CAS-I, CAS-III					
3798341 ->3797592	-	WP_041333821.1	NC_009523.1	Roseiflexus sp. RS-1	Bacteria	Chloroflexi	Chloroflexia	Chloroflexales	Roseiflexineae	Roseiflexaceae	H	Csx1	pfam09670						
3799377 ->3798319	-	WP_011957728.1	NC_009523.1	Roseiflexus sp. RS-1	Bacteria	Chloroflexi	Chloroflexia	Chloroflexales	Roseiflexineae	Roseiflexaceae	WYL domain-containing protein	WYL	COG2378	CAS-I, CAS-III					
3800785 ->3799388	-	WP_011957729.1	NC_009523.1	Roseiflexus sp. RS-1	Bacteria	Chloroflexi	Chloroflexia	Chloroflexales	Roseiflexineae	Roseiflexaceae	CRISPR-associated protein	Csx1	cd09747	CAS-III					
Partial CAS-III																			
3453071 ->3447453	-		NC_009767.1	Roseiflexus castenholzii DSM 13941	Bacteria	Chloroflexi	Chloroflexia	Chloroflexales	Roseiflexineae	Roseiflexaceae	repeat region	CRISPR Array			75 (36-41)	76 (37)	M16/NA/E	AAAAAAACAAAAGCCCCGCCATGAAGGGGATTGAAAC	H
3453182 ->3455278	+	WP_012121168.1	NC_009767.1	Roseiflexus castenholzii DSM 13941	Bacteria	Chloroflexi	Chloroflexia	Chloroflexales	Roseiflexineae	Roseiflexaceae	WYL domain-containing protein	WYL	pfam13280	CAS-I, CAS-III					

3455753 ->3455478	-	WP_012121169.1	NC_009767.1	Roseiflexus castenholzii DSM 13941	Bacteria	Chloroflexi	Chloroflexia	Chloroflexales	Roseiflexineae	Roseiflexaceae	CRISPR-associated endonuclease Cas2	Cas2	mkCas0081	CAS-I, CAS-II, CAS-III					
3458266 ->3455933	-	WP_012121170.1	NC_009767.1	Roseiflexus castenholzii DSM 13941	Bacteria	Chloroflexi	Chloroflexia	Chloroflexales	Roseiflexineae	Roseiflexaceae	VWA domain-containing protein								
3459385 ->3458387	-	WP_012121171.1	NC_009767.1	Roseiflexus castenholzii DSM 13941	Bacteria	Chloroflexi	Chloroflexia	Chloroflexales	Roseiflexineae	Roseiflexaceae	CRISPR-associated endonuclease Cas1	Cas1	pfam01867	CAS-I, CAS-II, CAS-III					
3461067 ->3459724	-	WP_012121172.1	NC_009767.1	Roseiflexus castenholzii DSM 13941	Bacteria	Chloroflexi	Chloroflexia	Chloroflexales	Roseiflexineae	Roseiflexaceae	RNA-dependent DNA polymerase	RT	pfam00078	CAS-III					
3462543 ->3461083	-	WP_012121173.1	NC_009767.1	Roseiflexus castenholzii DSM 13941	Bacteria	Chloroflexi	Chloroflexia	Chloroflexales	Roseiflexineae	Roseiflexaceae	H								
3463007 ->3462558	-	WP_012121174.1	NC_009767.1	Roseiflexus castenholzii DSM 13941	Bacteria	Chloroflexi	Chloroflexia	Chloroflexales	Roseiflexineae	Roseiflexaceae	H								
3463192 ->3463004	-	WP_012121175.1	NC_009767.1	Roseiflexus castenholzii DSM 13941	Bacteria	Chloroflexi	Chloroflexia	Chloroflexales	Roseiflexineae	Roseiflexaceae	H								
3464346 ->3463969	-	WP_012121176.1	NC_009767.1	Roseiflexus castenholzii DSM 13941	Bacteria	Chloroflexi	Chloroflexia	Chloroflexales	Roseiflexineae	Roseiflexaceae	H	Csx15	cd09766	CAS-I, CAS-III					
3465086->3464343	-	WP_012121177.1	NC_009767.1	Roseiflexus castenholzii DSM 13941	Bacteria	Chloroflexi	Chloroflexia	Chloroflexales	Roseiflexineae	Roseiflexaceae	H	Csx1	cd09702						
3466129->3465083	-	WP_012121178.1	NC_009767.1	Roseiflexus castenholzii DSM 13941	Bacteria	Chloroflexi	Chloroflexia	Chloroflexales	Roseiflexineae	Roseiflexaceae	WYL domain-containing protein	WYL	COG2378	CAS-I, CAS-III					
3467688->3466291	-	WP_012121179.1	NC_009767.1	Roseiflexus castenholzii DSM 13941	Bacteria	Chloroflexi	Chloroflexia	Chloroflexales	Roseiflexineae	Roseiflexaceae	CRISPR-associated protein	Csx1	cd09747	CAS-III					
Partial																			
4528939 ->4530243	+	YP_001636958.1	NC_010175.1	Chloroflexus aurantiacus J-10-fl	Bacteria	Chloroflexi	Chloroflexia	Chloroflexales	Chloroflexaceae	Chloroflexus	RNA-directed DNA polymerase	RT	pfam00078	CAS-III					
4530275 ->4531270	+	YP_001636959.1	NC_010175.1	Chloroflexus aurantiacus J-10-fl	Bacteria	Chloroflexi	Chloroflexia	Chloroflexales	Chloroflexaceae	Chloroflexus	CRISPR-associated Cas1 family protein	Cas1	pfam01867	CAS-I, CAS-II, CAS-III					
4531267 ->4531548	+	YP_001636960.1	NC_010175.1	Chloroflexus aurantiacus J-10-fl	Bacteria	Chloroflexi	Chloroflexia	Chloroflexales	Chloroflexaceae	Chloroflexus	CRISPR-associated Cas2 family protein	Cas2	mkCas0081	CAS-I, CAS-II, CAS-III					
4533651 ->4531549	-	YP_001636961.1	NC_010175.1	Chloroflexus aurantiacus J-10-fl	Bacteria	Chloroflexi	Chloroflexia	Chloroflexales	Chloroflexaceae	Chloroflexus	H	WYL	COG2378	CAS-I, CAS-III					
4533814 ->4540010	+		NC_010175.1	Chloroflexus aurantiacus J-10-fl	Bacteria	Chloroflexi	Chloroflexia	Chloroflexales	Chloroflexaceae	Chloroflexus		CRISPR Array			83 (34-40)	84 (37)	M16/NA/E	CAGCAGAGCATTGCCCCGCAATGAAGGGGTTTGAAAC	H

RT-CRISPR-10 (CAS-III-A/D, CAS-III-B/C)

Actinobacteria (Pseudonocardiales, Propionibacteriales, Corynebacteriales, Nakamurellales, Actinomycetales, Frankiales, Micromonosporales, Streptosporangiales, Micrococcales

[a](Sub) Type/[b]Coordinates	Strand	Protein_id	Genome Partition ID	Strain	Domain	Phylum	Lineage (class)	Order	Family	Genus	[c]Cas gene (annotated)	[d]Cas gene/[e]Array (identified)	[d]Profiles	[d](Sub)Type-specificity	[e]Spacers (length)	[e]Repeats (length)	[f]Str.motif/Seq.family/Superclass	[f]Seq Repeats	[g]Orientation Confidence
CAS-III-B																			
11612415 -> 11614145	+	WP_043727711.1	NZ_KK037166	Kutzneria sp. 744	Bacteria	Actinobacteria	Actinobacteria	Pseudonocardiales	Pseudonocardiaceae	Kutzneria	H	Cas10	cls000742	CAS-III					
11614376 -> 11614681	+	WP_043727713.1	NZ_KK037166	Kutzneria sp. 744	Bacteria	Actinobacteria	Actinobacteria	Pseudonocardiales	Pseudonocardiaceae	Kutzneria	H	Cmr3gr5	pfam09700						
11614675 -> 11615856	+	WP_052396491.1	NZ_KK037166	Kutzneria sp. 744	Bacteria	Actinobacteria	Actinobacteria	Pseudonocardiales	Pseudonocardiaceae	Kutzneria	type III-B CRISPR module RAMP protein Cmr4	Cmr4gr7	cd09682	CAS-III-B					
11616615 -> 11617001	+	WP_043727714.1	NZ_KK037166	Kutzneria sp. 744	Bacteria	Actinobacteria	Actinobacteria	Pseudonocardiales	Pseudonocardiaceae	Kutzneria	transcriptional regulator	Csa3	COG0640	CAS-I-A					
11617110 -> 11617766	+	WP_052396492.1	NZ_KK037166	Kutzneria sp. 744	Bacteria	Actinobacteria	Actinobacteria	Pseudonocardiales	Pseudonocardiaceae	Kutzneria	type III-B CRISPR module RAMP protein Cmr6	Cmr6gr7	COG1604	CAS-III-B					
11617766 -> 11618686	+	WP_043727717.1	NZ_KK037166	Kutzneria sp. 744	Bacteria	Actinobacteria	Actinobacteria	Pseudonocardiales	Pseudonocardiaceae	Kutzneria	H								
11618683 -> 11620086	+	WP_043727720.1	NZ_KK037166	Kutzneria sp. 744	Bacteria	Actinobacteria	Actinobacteria	Pseudonocardiales	Pseudonocardiaceae	Kutzneria	H								
11620083 -> 11621303	+	WP_043727722.1	NZ_KK037166	Kutzneria sp. 744	Bacteria	Actinobacteria	Actinobacteria	Pseudonocardiales	Pseudonocardiaceae	Kutzneria	type III-B CRISPR module RAMP protein Cmr1	Cmr1gr7	COG1367	CAS-III-B					
11621307 -> 11622041	+	WP_043727725.1	NZ_KK037166	Kutzneria sp. 744	Bacteria	Actinobacteria	Actinobacteria	Pseudonocardiales	Pseudonocardiaceae	Kutzneria	H	Cas6	pfam1040						
11622034 -> 11623908	+	WP_052396493.1	NZ_KK037166	Kutzneria sp. 744	Bacteria	Actinobacteria	Actinobacteria	Pseudonocardiales	Pseudonocardiaceae	Kutzneria	CRISPR-associated endonuclease Cas1	RT-Cas1	pfam00078, cd09634	CAS-III; CAS-I, CAS-II, CAS-III, CASV					
11623899 -> 11624183	+	WP_043727727.1	NZ_KK037166	Kutzneria sp. 744	Bacteria	Actinobacteria	Actinobacteria	Pseudonocardiales	Pseudonocardiaceae	Kutzneria	CRISPR-associated endonuclease Cas2	Cas2	COG1343	CAS-I, CAS-II, CAS-III					
11624362 -> 11625218	+		NZ_KK037166	Kutzneria sp. 744	Bacteria	Actinobacteria	Actinobacteria	Pseudonocardiales	Pseudonocardiaceae	Kutzneria		CRISPR Array			11(35-41)	12(37)	M17/NA/E	GTTAGGTCAGACCTCACGCCGAAGGGCGTATAGAA	ND
CAS-III-D; CAS-III-D																			
1233492 -> 1235552	+	BAK34144.1	AP012204	Microlunatus phosphovorus NM-1	Bacteria	Actinobacteria	Actinobacteria	Propionibacteriales	Propionibacteriaceae	Microlunatus	H	Csmr3gr7	cls000253	CAS-III-A, CAS-III-D					
1235549 -> 1237012	+	BAK34145.1	AP012204	Microlunatus phosphovorus NM-1	Bacteria	Actinobacteria	Actinobacteria	Propionibacteriales	Propionibacteriaceae	Microlunatus	H	Cas10	cls000742	CAS-III					
1237009 -> 1237560	+	BAK34146.1	AP012204	Microlunatus phosphovorus NM-1	Bacteria	Actinobacteria	Actinobacteria	Propionibacteriales	Propionibacteriaceae	Microlunatus	H	Csm3gr7	cls000184	CAS-III-D					
1237557 -> 1238825	+	BAK34147.1	AP012204	Microlunatus phosphovorus NM-1	Bacteria	Actinobacteria	Actinobacteria	Propionibacteriales	Propionibacteriaceae	Microlunatus	H	Csx10gr5	cls000715	CAS-III-D					
1238822 -> 1240093	+	BAK34148.1	AP012204	Microlunatus phosphovorus NM-1	Bacteria	Actinobacteria	Actinobacteria	Propionibacteriales	Propionibacteriaceae	Microlunatus	H	Csm3gr7	cd09726	CAS-III-D					
1240090 -> 1240527	+	BAK34149.1	AP012204	Microlunatus phosphovorus NM-1	Bacteria	Actinobacteria	Actinobacteria	Propionibacteriales	Propionibacteriaceae	Microlunatus	H	Csx24	mkCas0135	CAS-III-D					
1240524 -> 1241765	+	BAK34148.1	AP012204	Microlunatus phosphovorus NM-1	Bacteria	Actinobacteria	Actinobacteria	Propionibacteriales	Propionibacteriaceae	Microlunatus	H								
1241762 -> 1242127	+	BAK34151.1	AP012204	Microlunatus phosphovorus NM-1	Bacteria	Actinobacteria	Actinobacteria	Propionibacteriales	Propionibacteriaceae	Microlunatus	H								
1242127 -> 1242864	+	BAK34152.1	AP012204	Microlunatus phosphovorus NM-1	Bacteria	Actinobacteria	Actinobacteria	Propionibacteriales	Propionibacteriaceae	Microlunatus	H	Cas6	pfam10040	CAS-III, CAS-IV					
1242875 -> 1244752	+	BAK34153.1	AP012204	Microlunatus phosphovorus NM-1	Bacteria	Actinobacteria	Actinobacteria	Propionibacteriales	Propionibacteriaceae	Microlunatus	Cas1	RT-Cas1	pfam00078, cd09634	CAS-III; CAS-I, CAS-II, CAS-III, CAS-V					
1244743 -> 1245027	+	BAK34154.1	AP012204	Microlunatus phosphovorus NM-1	Bacteria	Actinobacteria	Actinobacteria	Propionibacteriales	Propionibacteriaceae	Microlunatus	Cas2	Cas2	mkCas0081	CAS-I, CAS-II, CAS-III					
1245236 -> 1249826	+		AP012204	Microlunatus phosphovorus NM-1	Bacteria	Actinobacteria	Actinobacteria	Propionibacteriales	Propionibacteriaceae	Microlunatus		CRISPR Array			62 (30-57)	63 (37)	M17/NA/E	GTCAGCGACCGCATCTCGCGAATGAGCGAATAGAA	H
1250253 -> 1252712	+	BAK34155.1	AP012204	Microlunatus phosphovorus NM-1	Bacteria	Actinobacteria	Actinobacteria	Propionibacteriales	Propionibacteriaceae	Microlunatus	H	Csm3gr7	cls000253	CAS-III-A, CAS-III-D					
1252712 -> 1254100	+	BAK34156.1	AP012204	Microlunatus phosphovorus NM-1	Bacteria	Actinobacteria	Actinobacteria	Propionibacteriales	Propionibacteriaceae	Microlunatus	H	Cas10	mkCas0133	CAS-III					
1254097 -> 1254660	+	BAK34157.1	AP012204	Microlunatus phosphovorus NM-1	Bacteria	Actinobacteria	Actinobacteria	Propionibacteriales	Propionibacteriaceae	Microlunatus	H	Csm3gr7	cls000184	CAS-III-D					
1254657 -> 1255679	+	BAK34158.1	AP012204	Microlunatus phosphovorus NM-1	Bacteria	Actinobacteria	Actinobacteria	Propionibacteriales	Propionibacteriaceae	Microlunatus	H	Csx10gr5	cls000715	CAS-III-D					
1255732 -> 1257174	+	BAK34159.1	AP012204	Microlunatus phosphovorus NM-1	Bacteria	Actinobacteria	Actinobacteria	Propionibacteriales	Propionibacteriaceae	Microlunatus	H	Csm3gr7	cd09684						
CAS-III-D																			
17953 -> 17489	-	WP_028709289.1	NZ_KE384022	Propionicicella superfundia DSM 22317	Bacteria	Actinobacteria	Actinobacteria	Propionibacteriales	Nocardioidaceae	Propionicicella	H	Csx24	mkCas0135	CAS-III-D					
18645 -> 17950	-	WP_028709290.1	NZ_KE384022	Propionicicella superfundia DSM 22317	Bacteria	Actinobacteria	Actinobacteria	Propionibacteriales	Nocardioidaceae	Propionicicella	H	Csm3gr7	cd09726	CAS-III-D					
20240 -> 19230	-	WP_028709291.1	NZ_KE384022	Propionicicella superfundia DSM 22317	Bacteria	Actinobacteria	Actinobacteria	Propionibacteriales	Nocardioidaceae	Propionicicella	H	Csx10gr5	cls000715	CAS-III-D					
21163 -> 20591	-	WP_051209227.1	NZ_KE384022	Propionicicella superfundia DSM 22317	Bacteria	Actinobacteria	Actinobacteria	Propionibacteriales	Nocardioidaceae	Propionicicella	H	Csm3gr7	cls000184	CAS-III-D					
22209 -> 21160	-	WP_028709292.1	NZ_KE384022	Propionicicella superfundia DSM 22317	Bacteria	Actinobacteria	Actinobacteria	Propionibacteriales	Nocardioidaceae	Propionicicella	H	Cas10	cls000742	CAS-III					
23471 -> 22629	-	WP_028709293.1	NZ_KE384022	Propionicicella superfundia DSM 22317	Bacteria	Actinobacteria	Actinobacteria	Propionibacteriales	Nocardioidaceae	Propionicicella	H	Csm3gr7	cls000253	CAS-III-A, CAS-III-D					
24658 -> 24969	+	WP_028709294.1	NZ_KE384022	Propionicicella superfundia DSM 22317	Bacteria	Actinobacteria	Actinobacteria	Propionibacteriales	Nocardioidaceae	Propionicicella	H								
26345 -> 25146	-	WP_028709295.1	NZ_KE384022	Propionicicella superfundia DSM 22317	Bacteria	Actinobacteria	Actinobacteria	Propionibacteriales	Nocardioidaceae	Propionicicella	H								
26829 -> 26545	-	WP_028709296.1	NZ_KE384022	Propionicicella superfundia DSM 22317	Bacteria	Actinobacteria	Actinobacteria	Propionibacteriales	Nocardioidaceae	Propionicicella	CRISPR-associated endonuclease Cas2	Cas2	mkCas0081	CAS-I, CAS-II, CAS-III					
28451 -> 26820	-	WP_051209229.1	NZ_KE384022	Propionicicella superfundia DSM 22317	Bacteria	Actinobacteria	Actinobacteria	Propionibacteriales	Nocardioidaceae	Propionicicella	CRISPR-associated endonuclease Cas1	RT-Cas1	pfam00078, cd09634	CAS-III; CAS-I, CAS-II, CAS-III, CASV					
29412 -> 28699	-	WP_028709297.1	NZ_KE384022	Propionicicella superfundia DSM 22317	Bacteria	Actinobacteria	Actinobacteria	Propionibacteriales	Nocardioidaceae	Propionicicella	H								
31116->31852	-		NZ_KE384022	Propionicicella superfundia DSM 22317	Bacteria	Actinobacteria	Actinobacteria	Propionibacteriales	Nocardioidaceae	Propionicicella		CRISPR Array			9(31-42)	10(37)	M17/NA/E	GTCCATAGGGACATCTCGCGAAACAGCGAATTGAA	H
CAS-III-D																			
590580 -> 591341	+	WP_040360112.1	NZ_KB290831	Corynebacterium durum F0235	Bacteria	Actinobacteria	Actinobacteria	Corynebacteriales	Corynebacteriaceae	Corynebacterium	H	Cas6	pfam10040	CAS-III, CAS-IV					
591346 -> 593238	+	WP_006063846.1	NZ_KB290831	Corynebacterium durum F0235	Bacteria	Actinobacteria	Actinobacteria	Corynebacteriales	Corynebacteriaceae	Corynebacterium	CRISPR-associated endonuclease Cas1	RT-Cas1	pfam00078, COG1518	CAS-III; CAS-I, CAS-II, CAS-III					
593229 -> 593513	+	WP_006063847.1	NZ_KB290831	Corynebacterium durum F0235	Bacteria	Actinobacteria	Actinobacteria	Corynebacteriales	Corynebacteriaceae	Corynebacterium	CRISPR-associated protein Cas2	Cas2	mkCas0081	CAS-I, CAS-II, CAS-III					
593707 -> 594841	+		NZ_KB290831	Corynebacterium durum F0235	Bacteria	Actinobacteria	Actinobacteria	Corynebacteriales	Corynebacteriaceae	Corynebacterium		CRISPR Array (assembly gaps)			ND	>8(37)	M17/NA/E	GTTGGAAACCAAGTACCGCGATATAGCGGATGTAA	ND
596382 -> 595090	-	WP_040360115.1	NZ_KB290831	Corynebacterium durum F0235	Bacteria	Actinobacteria	Actinobacteria	Corynebacteriales	Corynebacteriaceae	Corynebacterium	H								
596805 -> 596395	-	WP_006063849.1	NZ_KB290831	Corynebacterium durum F0235	Bacteria	Actinobacteria	Actinobacteria	Corynebacteriales	Corynebacteriaceae	Corynebacterium	H	Csx24	mkCas0135	CAS-III-D					
598193 -> 596820	-	WP_006063850.1	NZ_KB290831	Corynebacterium durum F0235	Bacteria	Actinobacteria	Actinobacteria	Corynebacteriales	Corynebacteriaceae	Corynebacterium	CRISPR-associated RAMP protein	Csm3gr7	cd09684						
599239 -> 598193	-	WP_006063851.1	NZ_KB290831	Corynebacterium durum F0235	Bacteria	Actinobacteria	Actinobacteria	Corynebacteriales	Corynebacteriaceae	Corynebacterium	H	Csx10gr5	cls000715	CAS-III-D					
599807 -> 599232	-	WP_006063852.1	NZ_KB290831	Corynebacterium durum F0235	Bacteria	Actinobacteria	Actinobacteria	Corynebacteriales	Corynebacteriaceae	Corynebacterium	CRISPR-associated RAMP protein	Csm3gr7	cls000184	CAS-III-D					
601285 -> 599804	-	WP_006063853.1	NZ_KB290831	Corynebacterium durum F0235	Bacteria	Actinobacteria	Actinobacteria	Corynebacteriales	Corynebacteriaceae	Corynebacterium	H	Cas10	cls000742	CAS-III					
603210 -> 601291	-	WP_040360119.1	NZ_KB290831	Corynebacterium durum F0235	Bacteria	Actinobacteria	Actinobacteria	Corynebacteriales	Corynebacteriaceae	Corynebacterium	H	Csm3gr7	cls000253	CAS-III-A, CAS-III-D					
CAS-III-D																			
1347640 -> 1349157		ACV77630.1	CP001737	Nakamurella multipartita DSM 44233	Bacteria	Actinobacteria	Actinobacteria	Nakamurellales	Nakamurellaceae	Nakamurella	H	Cas10	mkCas0133	CAS-III					
1349154 -> 1349705	+	ACV77631.1	CP001737	Nakamurella multipartita DSM 44233	Bacteria	Actinobacteria	Actinobacteria	Nakamurellales	Nakamurellaceae	Nakamurella	H	Csm3gr7	cls000184	CAS-III-D					
1349786 -> 1351159	+	ACV77632.1	CP001737	Nakamurella multipartita DSM 44233	Bacteria	Actinobacteria	Actinobacteria	Nakamurellales	Nakamurellaceae	Nakamurella	H	Csx10gr5	cls000715	CAS-III-D					
1351156 -> 1352517	+	ACV77633.1	CP001737	Nakamurella multipartita DSM 44233	Bacteria	Actinobacteria	Actinobacteria	Nakamurellales	Nakamurellaceae	Nakamurella	Protein of unknown function DUF324	Csm3gr7	cd09726	CAS-III-D					
1352514 -> 1352963	+	ACV77634.1	CP001737	Nakamurella multipartita DSM 44233	Bacteria	Actinobacteria	Actinobacteria	Nakamurellales	Nakamurellaceae	Nakamurella	H	Csx24	mkCas0135	CAS-III-D					
1352973 -> 1354085	+	ACV77635.1	CP001737	Nakamurella multipartita DSM 44233	Bacteria	Actinobacteria	Actinobacteria	Nakamurellales	Nakamurellaceae	Nakamurella	H	Cas6	pfam10040						
1354085 -> 1354459	+	ACV77636.1	CP001737	Nakamurella multipartita DSM 44233	Bacteria	Actinobacteria	Actinobacteria	Nakamurellales	Nakamurellaceae	Nakamurella	H								
1354443 -> 1355168	+		CP001737	Nakamurella multipartita DSM 44233	Bacteria	Actinobacteria	Actinobacteria	Nakamurellales	Nakamurellaceae	Nakamurella	H	Cas6	pfam10040	CAS-III, CAS-IV					
1355212 -> 1355586	+	ACV77638.1	CP001737	Nakamurella multipartita DSM 44233	Bacteria	Actinobacteria	Actinobacteria	Nakamurellales	Nakamurellaceae	Nakamurella	reverse transcriptase_maturase family protein	RT(truncated)							
1356877 -> 1355630	-	ACV77639.1	CP001737	Nakamurella multipartita DSM 44233	Bacteria	Actinobacteria	Actinobacteria	Nakamurellales	Nakamurellaceae	Nakamurella	transposase mutator type								
1356930 -> 1357865	+	ACV77640.1	CP001737	Nakamurella multipartita DSM 44233	Bacteria	Actinobacteria	Actinobacteria	Nakamurellales	Nakamurellaceae	Nakamurella	H	RT-Cas1	pfam00078, cd09634	CAS-III					
1358730 -> 1357855	-	ACV77641.1	CP001737	Nakamurella multipartita DSM 44233	Bacteria	Actinobacteria	Actinobacteria	Nakamurellales	Nakamurellaceae	Nakamurella	integrase catalytic region								
1358929 -> 1359933	+	ACV77642.1	CP001737	Nakamurella multipartita DSM 44233	Bacteria	Actinobacteria	Actinobacteria	Nakamurellales	Nakamurellaceae	Nakamurella	integrase family protein								
1359930 -> 1361783	+	ACV77643.1	CP001737	Nakamurella multipartita DSM 44233	Bacteria	Actinobacteria	Actinobacteria	Nakamurellales	Nakamurellaceae	Nakamurella	integrase family protein								
1362091 -> 1361756	-	ACV77644.1	CP001737	Nakamurella multipartita DSM 44233	Bacteria	Actinobacteria	Actinobacteria	Nakamurellales	Nakamurellaceae	Nakamurella	transposase IS3_IS911 family								
1362747 -> 1363034	+	ACV77645.1	CP001737	Nakamurella multipartita DSM 44233	Bacteria	Actinobacteria	Actinobacteria	Nakamurellales	Nakamurellaceae	Nakamurella	transposase IS3_IS911 family								
1363055 -> 1363936	+	ACV77646.1	CP001737	Nakamurella multipartita DSM 44233	Bacteria	Actinobacteria	Actinobacteria	Nakamurellales	Nakamurellaceae	Nakamurella	integrase catalytic region								
1363985 -> 1364143	+	ACV77647.1	CP001737	Nakamurella multipartita DSM 44233	Bacteria	Actinobacteria	Actinobacteria	Nakamurellales	Nakamurellaceae	Nakamurella	H								
1364134 -> 1364418	+	ACV77648.1	CP001737	Nakamurella multipartita DSM 44233	Bacteria	Actinobacteria	Actinobacteria	Nakamurellales	Nakamurellaceae	Nakamurella	CRISPR-associated protein Cas2	Cas2	cd09725	CAS-I, CAS-II, CAS-III, CASV					
1364626 -> 1365761	+		CP001737	Nakamurella multipartita DSM 44233	Bacteria	Actinobacteria	Actinobacteria	Nakamurellales	Nakamurellaceae	Nakamurella		CRISPR Array			15 (31-44)	16 (37)	M17/NA/E	GTCAGAAACCACGTCACGCCGTCAGGCGTATAGAA	M
1365872 -> 1367176	+	ACV77649.1	CP001737	Nakamurella multipartita DSM 44233	Bacteria	Actinobacteria	Actinobacteria	Nakamurellales	Nakamurellaceae	Nakamurella	transposase IS204_IS1001_IS1096_IS1165 family protein								
1367179 -> 1367813	+		CP001737	Nakamurella multipartita DSM 44233	Bacteria	Actinobacteria	Actinobacteria	Nakamurellales	Nakamurellaceae	Nakamurella		CRISPR Array			8 (33-42)	9 (37)	M17/NA/E	GTCAGAAACCACGTCACGCCGTCAGGCGTATAGAA	M
1368280 -> 1370439	+	ACV77650.1	CP001737	Nakamurella multipartita DSM 44233	Bacteria	Actinobacteria	Actinobacteria	Nakamurellales	Nakamurellaceae	Nakamurella	H	Csm3gr7	cls000253, cls000253	CAS-III-A, CAS-III-D; CAS-III-A, CAS-III-D					
CAS-III-D																			
2845722 -> 2847611	+	WP_053587370.1	NZ_CP012590	Actinomyces sp. oral taxon 414 strain F0588	Bacteria	Actinobacteria	Actinobacteria	Actinomycetales	Actinomycetaceae	Actynomyces	H	Cas10	mkCas0133, mkCas0133	CAS-III; CAS-III					
2847671 -> 2849770	+	WP_053587371.1	NZ_CP012590	Actinomyces sp. oral taxon 414 strain F0588	Bacteria	Actinobacteria	Actinobacteria	Actinomycetales	Actinomycetaceae	Actynomyces	H	Csx10gr5	cls000715						
2849767 -> 2851575	+	WP_053587372.1	NZ_CP012590	Actinomyces sp. oral taxon 414 strain F0588	Bacteria	Actinobacteria	Actinobacteria	Actinomycetales	Actinomycetaceae	Actynomyces	H	Csm3gr7	cd09726	CAS-III-D					
2851598 -> 2852092	+	WP_053587373.1	NZ_CP012590	Actinomyces sp. oral taxon 414 strain F0588	Bacteria	Actinobacteria	Actinobacteria	Actinomycetales	Actinomycetaceae	Actynomyces	H								
2852094 -> 2854328	+	WP_053587374.1	NZ_CP012590	Actinomyces sp. oral taxon 414 strain F0588	Bacteria	Actinobacteria	Actinobacteria	Actinomycetales	Actinomycetaceae	Actynomyces	H	Csm3gr7	cls000253, cls000253	CAS-III-A, CAS-III-D; CAS-III-A, CAS-III-D					
2856232 -> 2854574	-	WP_053587375.1	NZ_CP012590	Actinomyces sp. oral taxon 414 strain F0588	Bacteria	Actinobacteria	Actinobacteria	Actinomycetales	Actinomycetaceae	Actynomyces	H								
2858484 -> 2856244	-	WP_053587376.1	NZ_CP012590	Actinomyces sp. oral taxon 414 strain F0588	Bacteria	Actinobacteria	Actinobacteria	Actinomycetales	Actinomycetaceae	Actynomyces	H	Cas3	cd09639						
2858898 -> 2860943	+		NZ_CP012590	Actinomyces sp. oral taxon 414 strain F0588	Bacteria	Actinobacteria	Actinobacteria	Actinomycetales	Actinomycetaceae	Actynomyces	Pseudo	Cas10	mkCas0133, mkCas0133	CAS-III; CAS-III					
2861015 -> 2863144	+	WP_053587377.1	NZ_CP012590	Actinomyces sp. oral taxon 414 strain F0588	Bacteria	Actinobacteria	Actinobacteria	Actinomycetales	Actinomycetaceae	Actynomyces	H	Csx10gr5	cls000715	CAS-III-D					
2863141 -> 2864871	+	WP_053587378.1	NZ_CP012590	Actinomyces sp. oral taxon 414 strain F0588	Bacteria	Actinobacteria	Actinobacteria	Actinomycetales	Actinomycetaceae	Actynomyces	H	Csm3gr7	cd09726						
2864864 -> 2865388	+	WP_053587379.1	NZ_CP012590	Actinomyces sp. oral taxon 414 strain F0588	Bacteria	Actinobacteria	Actinobacteria	Actinomycetales	Actinomycetaceae	Actynomyces	H								
2865390-> 2867627	+	WP_053587380.1	NZ_CP012590	Actinomyces sp. oral taxon 414 strain F0588	Bacteria	Actinobacteria	Actinobacteria	Actinomycetales	Actinomycetaceae	Actynomyces	H	Csm3gr7	cls000253	CAS-III-A, CAS-III-D					
2868159 -> 2869069	+		NZ_CP012590	Actinomyces sp. oral taxon 414 strain F0588	Bacteria	Actinobacteria	Actinobacteria	Actinomycetales	Actinomycetaceae	Actynomyces		CRISPR Array			12 (34-38)	13 (37)	M17/NA/NA	GTCAGGGAGCACCACGCACCTGTAGGTGCATTGAG	M
2870580 -> 2869294	-		NZ_CP012590	Actinomyces sp. oral taxon 414 strain F0588	Bacteria	Actinobacteria	Actinobacteria	Actinomycetales	Actinomycetaceae	Actynomyces	Pseudo-Transposase								
2871180 -> 2873318	+	WP_053587381.1	NZ_CP012590	Actinomyces sp. oral taxon 414 strain F0588	Bacteria	Actinobacteria	Actinobacteria	Actinomycetales	Actinomycetaceae	Actynomyces	CRISPR-associated endonuclease Cas1	RT-Cas1	pfam00078, cd09634	CAS-III; CAS-I, CAS-II, CAS-III, CASV					
2873309 -> 2873596	-	WP_026410503.1	NZ_CP012590	Actinomyces sp. oral taxon 414 strain F0588	Bacteria	Actinobacteria	Actinobacteria	Actinomycetales	Actinomycetaceae	Actynomyces	CRISPR-associated endonuclease Cas2	Cas2	cd09725	CAS-I,CAS-II,CAS-III,CAS-V					
2873932 -> 2873663	+	WP_053587382.1	NZ_CP012590	Actinomyces sp. oral taxon 414 strain F0588	Bacteria	Actinobacteria	Actinobacteria	Actinomycetales	Actinomycetaceae	Actynomyces	prevent-host-death protein								
2876302-> 2874002	-	WP_053587383.1	NZ_CP012590	Actinomyces sp. oral taxon 414 strain F0588	Bacteria	Actinobacteria	Actinobacteria	Actinomycetales	Actinomycetaceae	Actynomyces	H	Csm3gr7	cls000253, cls000253	CAS-III-A, CAS-III-D; CAS-III-A, CAS-III-D					
2877146->2876530	-		NZ_CP012590	Actinomyces sp. oral taxon 414 strain F0588	Bacteria	Actinobacteria	Actinobacteria	Actinomycetales	Actinomycetaceae	Actynomyces		CRISPR Array			8 (34-38)	9 (37)	M17/NA/NA	GTCAGGGAGCACCACGCACCTGTAGGTGCATTGAG	L
CAS-III-A/CAS-III-D																			
56481 -> 57242	+	ENO18587.1	AQHZ01000010	Actinomyces cardiffensis F0333	Bacteria	Actinobacteria	Actinobacteria	Actinomycetales	Actinomycetaceae	Actinomyces	universal stress protein	Cas6	pfam10040						
58914 -> 57262	-	ENO18588.1	AQHZ01000010	Actinomyces cardiffensis F0333	Bacteria	Actinobacteria	Actinobacteria	Actinomycetales	Actinomycetaceae	Actinomyces	H								
59407 -> 58955	-	ENO18589.1	AQHZ01000010	Actinomyces cardiffensis F0333	Bacteria	Actinobacteria	Actinobacteria	Actinomycetales	Actinomycetaceae	Actinomyces	H								
61643 -> 59400	-	ENO18590.1	AQHZ01000010	Actinomyces cardiffensis F0333	Bacteria	Actinobacteria	Actinobacteria	Actinomycetales	Actinomycetaceae	Actinomyces	H								
61804 -> 63456	+	ENO18591.1	AQHZ01000010	Actinomyces cardiffensis F0333	Bacteria	Actinobacteria	Actinobacteria	Actinomycetales	Actinomycetaceae	Actinomyces	H	Cas10	mkCas0133	CAS-III					
63453 -> 65243	+	ENO18592.1	AQHZ01000010	Actinomyces cardiffensis F0333	Bacteria	Actinobacteria	Actinobacteria	Actinomycetales	Actinomycetaceae	Actinomyces	CRISPR-associated RAMP protein	Csm4gr5	cls000259						
65444 -> 66748	+	ENO18593.1	AQHZ01000010	Actinomyces cardiffensis F0333	Bacteria	Actinobacteria	Actinobacteria	Actinomycetales	Actinomycetaceae	Actinomyces	CRISPR-associated RAMP protein	Csm3gr7	cd09683, cd09683	CAS-III-A, CAS-III-D; CAS-III-A, CAS-III-D					
66745 -> 67263	+	ENO18594.1	AQHZ01000010	Actinomyces cardiffensis F0333	Bacteria	Actinobacteria	Actinobacteria	Actinomycetales	Actinomycetaceae	Actinomyces	H								
67265 -> 69409	+	ENO18595.1	AQHZ01000010	Actinomyces cardiffensis F0333	Bacteria	Actinobacteria	Actinobacteria	Actinomycetales	Actinomycetaceae	Actinomyces	protein of hypothetical function DUF324	Csm3gr7	cls000253, cls000253	CAS-III-A, CAS-III-D					
69733 -> 69446	-	HMPREF9004_0645	AQHZ01000010	Actinomyces cardiffensis F0333	Bacteria	Actinobacteria	Actinobacteria	Actinomycetales	Actinomycetaceae	Actinomyces	cas2	Cas2	COG1343	CAS-I, CAS-II, CAS-III					
71643 -> 69724	-	ENO18597.1	AQHZ01000010	Actinomyces cardiffensis F0333	Bacteria	Actinobacteria	Actinobacteria	Actinomycetales	Actinomycetaceae	Actinomyces	H	RT-Cas1	pfam00078, pfam01867	CAS-III; CAS-I,CAS-II,CAS-III					
72032 -> 75854	+		AQHZ01000010	Actinomyces cardiffensis F0333	Bacteria	Actinobacteria	Actinobacteria	Actinomycetales	Actinomycetaceae	Actinomyces		CRISPR Array			52(31-42)	53(37)	M17/NA/NA	GTCAGAAAGCACCTCGCGCCATAAGGTGCATTAAG	M
CAS-III-B/CAS-III-C																			
70512 -> 69256	-	WP_047221154.1	NZ_JWIO01000001	Frankia coriariae strain BMG5.1	Bacteria	Actinobacteria	Actinobacteria	Frankiales	Frankiaceae	Frankia	H	Cmr1gr7	cd09657	CAS-III-C					
72160 -> 70406	-	WP_047221155.1	NZ_JWIO01000001	Frankia coriariae strain BMG5.1	Bacteria	Actinobacteria	Actinobacteria	Frankiales	Frankiaceae	Frankia	H								

73142 -> 72216	-	WP_047221156.1	NZ_JWIO01000001	Frankia coriariae strain BMG5.1	Bacteria	Actinobacteria	Actinobacteria	Frankiales	Frankiaceae	Frankia	H	Csm6	cd09742						
73942 -> 73139	-	WP_047221157.1	NZ_JWIO01000001	Frankia coriariae strain BMG5.1	Bacteria	Actinobacteria	Actinobacteria	Frankiales	Frankiaceae	Frankia	Type III-B CRISPR module RAMP protein Cmr6	**Cmr6gr7**	COG1604	CAS-III-B					
74289 -> 73903	-	WP_047221158.1	NZ_JWIO01000001	Frankia coriariae strain BMG5.1	Bacteria	Actinobacteria	Actinobacteria	Frankiales	Frankiaceae	Frankia	H								
74753 -> 74286	-	WP_047221159.1	NZ_JWIO01000001	Frankia coriariae strain BMG5.1	Bacteria	Actinobacteria	Actinobacteria	Frankiales	Frankiaceae	Frankia	Type III-B CRISPR module-associated protein Cmr5	**Cmr5gr11**	cd09654	CAS-III-B					
75742 -> 74753	-	WP_047221160.1	NZ_JWIO01000001	Frankia coriariae strain BMG5.1	Bacteria	Actinobacteria	Actinobacteria	Frankiales	Frankiaceae	Frankia	Type III-B CRISPR module RAMP protein Cmr4	**Cmr4gr7**	COG1336	CAS-III-C					
77082 -> 75742	-	WP_047221161.1	NZ_JWIO01000001	Frankia coriariae strain BMG5.1	Bacteria	Actinobacteria	Actinobacteria	Frankiales	Frankiaceae	Frankia	H	**Cmr3gr5**	pfam09700, pfam09700	CAS-III-B					
79277 -> 77226	-	WP_047221162.1	NZ_JWIO01000001	Frankia coriariae strain BMG5.1	Bacteria	Actinobacteria	Actinobacteria	Frankiales	Frankiaceae	Frankia	Type III-B CRISPR-associated protein Cas10	**Cas10**	cd09679	CAS-III					
80206 ->79507	-		NZ_JWIO01000001	Frankia coriariae strain BMG5.1	Bacteria	Actinobacteria	Actinobacteria	Frankiales	Frankiaceae	Frankia		CRISPR Array			9(33-39)	10(37)	M16/NA/E	CTCGCTAACCCCAGCCCGATCATGAGGGCATAGAA	M
80537 -> 82408	+	WP_052914180.1	NZ_JWIO01000001	Frankia coriariae strain BMG5.1	Bacteria	Actinobacteria	Actinobacteria	Frankiales	Frankiaceae	Frankia	CRISPR-associated endonuclease Cas1	**RT-Cas1**	pfam00078, COG1518	CAS-III; CAS-I,CAS-II,CAS-III					
82448 -> 82729	+	WP_047221163.1	NZ_JWIO01000001	Frankia coriariae strain BMG5.1	Bacteria	Actinobacteria	Actinobacteria	Frankiales	Frankiaceae	Frankia	CRISPR-associated endonuclease Cas2	**Cas2**	mkCas0081	CAS-I, CAS-II, CAS-III					
83330 -> 83554	+	WP_047221164.1	NZ_JWIO01000001	Frankia coriariae strain BMG5.1	Bacteria	Actinobacteria	Actinobacteria	Frankiales	Frankiaceae	Frankia	H	WYL	COG2378						
83542 -> 83931	+	WP_047221165.1	NZ_JWIO01000001	Frankia coriariae strain BMG5.1	Bacteria	Actinobacteria	Actinobacteria	Frankiales	Frankiaceae	Frankia	H								
84849 -> 84456	-		NZ_JWIO01000001	Frankia coriariae strain BMG5.1	Bacteria	Actinobacteria	Actinobacteria	Frankiales	Frankiaceae	Frankia	chromosome condensation protein CrcB								
85190 -> 84846	-	WP_013872357.1	NZ_JWIO01000001	Frankia coriariae strain BMG5.1	Bacteria	Actinobacteria	Actinobacteria	Frankiales	Frankiaceae	Frankia	H								
85645 -> 85187	-	WP_047221264.1	NZ_JWIO01000001	Frankia coriariae strain BMG5.1	Bacteria	Actinobacteria	Actinobacteria	Frankiales	Frankiaceae	Frankia	chromosome condensation protein CrcB								
86616 -> 87161	+	WP_052914181.1	NZ_JWIO01000001	Frankia coriariae strain BMG5.1	Bacteria	Actinobacteria	Actinobacteria	Frankiales	Frankiaceae	Frankia	H	**Cas6**	pfam10040	CAS-III, CAS-IV					
**CAS-III-B/CAS-III-C**																			
622-> 351	-		LRQV01000138	Micromonospora rosaria strain DSM 803	Bacteria	Actinobacteria	Actinobacteria	Micromonosporales	Micromonosporaceae	Micromonospora		CRISPR Array			3(37-45)	4(37)	M16/NA/E	ATCGCCATCCACAGCCCAACCCAGCGGGCATAGAA	M
1140 -> 847	-	KXK58997.1	LRQV01000138	Micromonospora rosaria strain DSM 803	Bacteria	Actinobacteria	Actinobacteria	Micromonosporales	Micromonosporaceae	Micromonospora	H	**Cas2**	mkCas0081	CAS-I, CAS-II, CAS-III					
2807 -> 1131	-	KXK58998.1	LRQV01000138	Micromonospora rosaria strain DSM 803	Bacteria	Actinobacteria	Actinobacteria	Micromonosporales	Micromonosporaceae	Micromonospora	H	**RT-Cas1**	pfam00078, COG1518	CAS-III; CAS-I,CAS-II,CAS-III					
3667-> 3028	-		LRQV01000138	Micromonospora rosaria strain DSM 803	Bacteria	Actinobacteria	Actinobacteria	Micromonosporales	Micromonosporaceae	Micromonospora		CRISPR Array			8(34-42)	9(37)	M16/NA/E	ATTGCCATCCACAGCCCAACCCAGCGGGCATAGAA	H
4557 -> 3895	-	KXK58999.1	LRQV01000138	Micromonospora rosaria strain DSM 803	Bacteria	Actinobacteria	Actinobacteria	Micromonosporales	Micromonosporaceae	Micromonospora	H	**Cas6**	pfam10040	CAS-III, CAS-IV					
5419 -> 4652	-	KXK59000.1	LRQV01000138	Micromonospora rosaria strain DSM 803	Bacteria	Actinobacteria	Actinobacteria	Micromonosporales	Micromonosporaceae	Micromonospora	H								
5979 -> 5404	-	KXK59001.1	LRQV01000138	Micromonospora rosaria strain DSM 803	Bacteria	Actinobacteria	Actinobacteria	Micromonosporales	Micromonosporaceae	Micromonospora	H	**Cmr1gr7**	cd09657	CAS-III-C					
7411 -> 5984	-	KXK59002.1	LRQV01000138	Micromonospora rosaria strain DSM 803	Bacteria	Actinobacteria	Actinobacteria	Micromonosporales	Micromonosporaceae	Micromonospora	H								
8348 -> 7416	-	KXK59003.1	LRQV01000138	Micromonospora rosaria strain DSM 803	Bacteria	Actinobacteria	Actinobacteria	Micromonosporales	Micromonosporaceae	Micromonospora	H								
9160 -> 8345	-	KXK59004.1	LRQV01000138	Micromonospora rosaria strain DSM 803	Bacteria	Actinobacteria	Actinobacteria	Micromonosporales	Micromonosporaceae	Micromonospora	H	**Cmr6gr7**	cd09661	CAS-III-B					
9620 -> 9153	-	KXK59005.1	LRQV01000138	Micromonospora rosaria strain DSM 803	Bacteria	Actinobacteria	Actinobacteria	Micromonosporales	Micromonosporaceae	Micromonospora	H	Cmr5gr11	cd09654						
10548 -> 9607	-	KXK59006.1	LRQV01000138	Micromonospora rosaria strain DSM 803	Bacteria	Actinobacteria	Actinobacteria	Micromonosporales	Micromonosporaceae	Micromonospora	H	**Cmr4gr7**	COG1336	CAS-III-C					
11618 -> 10548	-	KXK59007.1	LRQV01000138	Micromonospora rosaria strain DSM 803	Bacteria	Actinobacteria	Actinobacteria	Micromonosporales	Micromonosporaceae	Micromonospora	H	**Cmr3gr5**	cd09748	CAS-III-B					
13417 -> 11615	-	KXK59008.1	LRQV01000138	Micromonospora rosaria strain DSM 803	Bacteria	Actinobacteria	Actinobacteria	Micromonosporales	Micromonosporaceae	Micromonospora	H	**Cas10**	cls000742	CAS-III					
**CAS-III-B**																			
107127 -> 107819	+	WP_017559358.1	NZ_ANAS01000036	Nocardiopsis baichengensis YIM 90130	Bacteria	Actinobacteria	Actinobacteria	Streptosporangiales	Nocardiopsaceae	Nocardiopsis	H	**Cas6**	pfam10040	CAS-III, CAS-IV					
107849 -> 109714	+	WP_017559359.1	NZ_ANAS01000036	Nocardiopsis baichengensis YIM 90130	Bacteria	Actinobacteria	Actinobacteria	Streptosporangiales	Nocardiopsaceae	Nocardiopsis	H	**Cas10**	cd09679	CAS-III					
109792 -> 110904	+	WP_017559360.1	NZ_ANAS01000036	Nocardiopsis baichengensis YIM 90130	Bacteria	Actinobacteria	Actinobacteria	Streptosporangiales	Nocardiopsaceae	Nocardiopsis	H	**Cmr3gr5**	cd09748	CAS-III-B					
110901 -> 111866	+	WP_017559361.1	NZ_ANAS01000036	Nocardiopsis baichengensis YIM 90130	Bacteria	Actinobacteria	Actinobacteria	Streptosporangiales	Nocardiopsaceae	Nocardiopsis	Type III-B CRISPR module RAMP protein Cmr4	**Cmr4gr7**	COG1336	CAS-III-C					
111859 -> 112353	+	WP_017559362.1	NZ_ANAS01000036	Nocardiopsis baichengensis YIM 90130	Bacteria	Actinobacteria	Actinobacteria	Streptosporangiales	Nocardiopsaceae	Nocardiopsis	H	Cmr5gr11	mkCas0172						
112350 -> 113393	+	WP_017559363.1	NZ_ANAS01000036	Nocardiopsis baichengensis YIM 90130	Bacteria	Actinobacteria	Actinobacteria	Streptosporangiales	Nocardiopsaceae	Nocardiopsis	Type III-B CRISPR module RAMP protein Cmr6	**Cmr6gr7**	cd09661	CAS-III-B					
113390 -> 114448	+	WP_017559364.1	NZ_ANAS01000036	Nocardiopsis baichengensis YIM 90130	Bacteria	Actinobacteria	Actinobacteria	Streptosporangiales	Nocardiopsaceae	Nocardiopsis	H								
114445 -> 116067	+	WP_017559365.1	NZ_ANAS01000036	Nocardiopsis baichengensis YIM 90130	Bacteria	Actinobacteria	Actinobacteria	Streptosporangiales	Nocardiopsaceae	Nocardiopsis	H								
116089 -> 117309	+	WP_017559366.1	NZ_ANAS01000036	Nocardiopsis baichengensis YIM 90130	Bacteria	Actinobacteria	Actinobacteria	Streptosporangiales	Nocardiopsaceae	Nocardiopsis	Type III-B CRISPR module RAMP protein Cmr1	**Cmr1gr7**	COG1367	CAS-III-B					
117574 -> 117827	+		NZ_ANAS01000036	Nocardiopsis baichengensis YIM 90130	Bacteria	Actinobacteria	Actinobacteria	Streptosporangiales	Nocardiopsaceae	Nocardiopsis		CRISPR Array			3(34-36)	4(37)	NA/NA/E	GGTCCGAAGGACGTCAAAGCCCGAAGGGCATAAGA	M
117921 -> 119747	+	WP_017559367.1	NZ_ANAS01000036	Nocardiopsis baichengensis YIM 90130	Bacteria	Actinobacteria	Actinobacteria	Streptosporangiales	Nocardiopsaceae	Nocardiopsis	CRISPR-associated endonuclease Cas1	**RT-Cas1**	pfam00078, pfam01867	CAS-III; CAS-I,CAS-II,CAS-III					
119755 -> 120057	+	WP_017559368.1	NZ_ANAS01000036	Nocardiopsis baichengensis YIM 90130	Bacteria	Actinobacteria	Actinobacteria	Streptosporangiales	Nocardiopsaceae	Nocardiopsis	CRISPR-associated endonuclease Cas2	**Cas2**	COG1343	CAS-I, CAS-II, CAS-III					
120244 -> 120564	+		NZ_ANAS01000036	Nocardiopsis baichengensis YIM 90130	Bacteria	Actinobacteria	Actinobacteria	Streptosporangiales	Nocardiopsaceae	Nocardiopsis		CRISPR Array			4(33-35)	5(37)	NA/NA/E	GGTCCGCAGGACGTCAAAGCCCGAAGGGCATAAGA	H
**CAS-III-B**																			
4483 -> 3302	-	WP_017593828.1	NZ_ANBB01000038	Nocardiopsis potens DSM 45234	Bacteria	Actinobacteria	Actinobacteria	Streptosporangiales	Nocardiopsaceae	Nocardiopsis	Type III-B CRISPR module RAMP protein Cmr1	**Cmr1gr7**	COG1367	CAS-III-B					
6284 -> 4494	-	WP_016187977.1	NZ_ANBB01000038	Nocardiopsis potens DSM 45234	Bacteria	Actinobacteria	Actinobacteria	Streptosporangiales	Nocardiopsaceae	Nocardiopsis	H								
7258 -> 6281	-	WP_017593830.1	NZ_ANBB01000038	Nocardiopsis potens DSM 45234	Bacteria	Actinobacteria	Actinobacteria	Streptosporangiales	Nocardiopsaceae	Nocardiopsis	H	Csm6	pfam09651						
8397 -> 7255	-	WP_017593831.1	NZ_ANBB01000038	Nocardiopsis potens DSM 45234	Bacteria	Actinobacteria	Actinobacteria	Streptosporangiales	Nocardiopsaceae	Nocardiopsis	Type III-B CRISPR module RAMP protein Cmr6	**Cmr6gr7**	cd09661	CAS-III-B					
8818 -> 8384	-	WP_017593832.1	NZ_ANBB01000038	Nocardiopsis potens DSM 45234	Bacteria	Actinobacteria	Actinobacteria	Streptosporangiales	Nocardiopsaceae	Nocardiopsis	Type III-B CRISPR module-associated protein Cmr5	**Cmr5gr11**	cd09654	CAS-III-B					
9740 -> 8811	-	WP_020380190.1	NZ_ANBB01000038	Nocardiopsis potens DSM 45234	Bacteria	Actinobacteria	Actinobacteria	Streptosporangiales	Nocardiopsaceae	Nocardiopsis	Type III-B CRISPR module RAMP protein Cmr4	**Cmr4gr7**	COG1336	CAS-III-C					
10855 -> 9737	-	WP_017593834.1	NZ_ANBB01000038	Nocardiopsis potens DSM 45234	Bacteria	Actinobacteria	Actinobacteria	Streptosporangiales	Nocardiopsaceae	Nocardiopsis	H	**Cmr3gr5**	cd09748	CAS-III-B					
12747 -> 10852	-	WP_017593835.1	NZ_ANBB01000038	Nocardiopsis potens DSM 45234	Bacteria	Actinobacteria	Actinobacteria	Streptosporangiales	Nocardiopsaceae	Nocardiopsis	Type III-B CRISPR-associated protein Cas10_Cmr2	**Cas10**	cls000742	CAS-III					
13410 -> 12811	-	WP_017593836.1	NZ_ANBB01000038	Nocardiopsis potens DSM 45234	Bacteria	Actinobacteria	Actinobacteria	Streptosporangiales	Nocardiopsaceae	Nocardiopsis	H	**Cas6**	pfam10040	CAS-III, CAS-IV					
14238 -> 14594	+	WP_017593837.1	NZ_ANBB01000038	Nocardiopsis potens DSM 45234	Bacteria	Actinobacteria	Actinobacteria	Streptosporangiales	Nocardiopsaceae	Nocardiopsis	H								
14645 -> 15577	+	WP_020380191.1	NZ_ANBB01000038	Nocardiopsis potens DSM 45234	Bacteria	Actinobacteria	Actinobacteria	Streptosporangiales	Nocardiopsaceae	Nocardiopsis	H	**RT-Cas1**	pfam00078, cd09634	CAS-III; CAS-I, CAS-II, CAS-III, CAS-V					
15927 -> 16109	+	WP_017593839.1	NZ_ANBB01000038	Nocardiopsis potens DSM 45234	Bacteria	Actinobacteria	Actinobacteria	Streptosporangiales	Nocardiopsaceae	Nocardiopsis	H	Cas2	COG1343						
**CAS-III-D**																			
45003 -> 44552	-		NZ_AXCZ01000003	Cellulomonas bogoriensis 69B4	Bacteria	Actinobacteria	Actinobacteria	Micrococcales	Cellulomonadaceae	Cellulomonas		CRISPR Array			6(32-35)	7(36)	M16/NA/E	GTTCCACCCATGGCCCGAAACTGAGGGCATTGAAA	H
45435 -> 45157	-	WP_052104812.1	NZ_AXCZ01000003	Cellulomonas bogoriensis 69B4	Bacteria	Actinobacteria	Actinobacteria	Micrococcales	Cellulomonadaceae	Cellulomonas	CRISPR-associated endonuclease Cas2	Cas2	cd09725						
47295 -> 45439	-	WP_052104813.1	NZ_AXCZ01000003	Cellulomonas bogoriensis 69B4	Bacteria	Actinobacteria	Actinobacteria	Micrococcales	Cellulomonadaceae	Cellulomonas	CRISPR-associated endonuclease Cas1	**RT-Cas1**	pfam00078, COG1518	CAS-III; CAS-I,CAS-II,CAS-III					
48090 -> 47332	-	WP_052104814.1	NZ_AXCZ01000003	Cellulomonas bogoriensis 69B4	Bacteria	Actinobacteria	Actinobacteria	Micrococcales	Cellulomonadaceae	Cellulomonas	H								
50254 -> 48065	-	WP_035056486.1	NZ_AXCZ01000003	Cellulomonas bogoriensis 69B4	Bacteria	Actinobacteria	Actinobacteria	Micrococcales	Cellulomonadaceae	Cellulomonas	CRISPR-associated RAMP family protein	**Csmr3gr7**	cls000253	CAS-III-A, CAS-III-D					
50756 -> 50256	-	WP_035056491.1	NZ_AXCZ01000003	Cellulomonas bogoriensis 69B4	Bacteria	Actinobacteria	Actinobacteria	Micrococcales	Cellulomonadaceae	Cellulomonas	H	**Csx19**	cls001376	CAS-III-D					
52357 -> 50774	-	WP_035056494.1	NZ_AXCZ01000003	Cellulomonas bogoriensis 69B4	Bacteria	Actinobacteria	Actinobacteria	Micrococcales	Cellulomonadaceae	Cellulomonas	H	**Csm3gr7**	cd09684	CAS-III-A, CAS-III-D					
54210 -> 52354	-	WP_035056497.1	NZ_AXCZ01000003	Cellulomonas bogoriensis 69B4	Bacteria	Actinobacteria	Actinobacteria	Micrococcales	Cellulomonadaceae	Cellulomonas	H	**Csx10gr5**	cls000715	CAS-III-D					

RT-CRISPR-11 (CAS-III-B)
Bacteroidetes (Bacteroidales)

[a](Sub) Type/[b]Coordinates	Strand	Protein_id	Genome Partition ID	Strain	Domain	Phylum	Lineage (class)	Order	Family	Genus	[c]Cas gene (annotated)	[d]Cas gene/[e]Array (identified)	[d]Profiles	[d](Sub)Type-specificity	[e]Spacers (length)	[e]Repeats (length)	[f]Str.motif/Seq.family/Superclass	[f]Seq Repeats	[g]Orientation Confidence
**CAS-III-B**																			
170739 ->172049	+	WP_018964670.1	NZ_JQJE01000003.1	Porphyromonas gulae OH3161B	Bacteria	Bacteroidetes	Bacteroidia	Bacteroidales	Porphyromonadaceae	Porphyromonas	H	**Cmr1gr7**	mkCas0086	CAS-III-B					
172039 ->173676	+	WP_026292125.1	NZ_JQJE01000003.1	Porphyromonas gulae OH3161B	Bacteria	Bacteroidetes	Bacteroidia	Bacteroidales	Porphyromonadaceae	Porphyromonas	type III-B CRISPR-associated protein Cas10_Cmr2	**Cas10**	pfam12469, cls000742	CAS-III-B, CAS-III					
173678 ->174913	+	WP_018964672.1	NZ_JQJE01000003.1	Porphyromonas gulae OH3161B	Bacteria	Bacteroidetes	Bacteroidia	Bacteroidales	Porphyromonadaceae	Porphyromonas	type III-B CRISPR module-associated protein Cmr3	**Cmr3gr5**	cd09656	CAS-III-B					
174927 ->175655	+	WP_026292126.1	NZ_JQJE01000003.1	Porphyromonas gulae OH3161B	Bacteria	Bacteroidetes	Bacteroidia	Bacteroidales	Porphyromonadaceae	Porphyromonas	type III-B CRISPR module RAMP protein Cmr4	**Cmr4gr7**	cd09682	CAS-III-B					
175648 ->176091	+	WP_018964674.1	NZ_JQJE01000003.1	Porphyromonas gulae OH3161B	Bacteria	Bacteroidetes	Bacteroidia	Bacteroidales	Porphyromonadaceae	Porphyromonas	type III-B CRISPR module-associated protein Cmr5	**Cmr5gr11**	mkCas0098	CAS-III-B					
176088 ->176852	+	WP_018964675.1	NZ_JQJE01000003.1	Porphyromonas gulae OH3161B	Bacteria	Bacteroidetes	Bacteroidia	Bacteroidales	Porphyromonadaceae	Porphyromonas	type III-B CRISPR module RAMP protein Cmr6	**Cmr6gr7**	cd09661	CAS-III-B					
176908 ->180006	+	WP_039443024.1	NZ_JQJE01000003.1	Porphyromonas gulae OH3161B	Bacteria	Bacteroidetes	Bacteroidia	Bacteroidales	Porphyromonadaceae	Porphyromonas	CRISPR-associated endonuclease Cas1	Cas6-**RT-Cas1**	mkCas0066, pfam00078, pfam01867	CAS-III; CAS-I, CAS-II, CAS-III					
180027 ->180317	+	WP_039443055.1	NZ_JQJE01000003.1	Porphyromonas gulae OH3161B	Bacteria	Bacteroidetes	Bacteroidia	Bacteroidales	Porphyromonadaceae	Porphyromonas	CRISPR-associated endonuclease Cas2	**Cas2**	pfam09827	CAS-I, CAS-II, CAS-III					
180717 ->182000	+		NZ_JQJE01000003.1	Porphyromonas gulae OH3161B	Bacteria	Bacteroidetes	Bacteroidia	Bacteroidales	Porphyromonadaceae	Porphyromonas		CRISPR Array			17 (34-39)	18 (37)	M4/NA/E	CTCACTATACATACACAGTCCGTAAGGCTATTAAGAC	L
**CAS-III-B**																			
2068904->2068360	-		NC_002950.2	Porphyromonas gingivalis W83	Bacteria	Bacteroidetes	Bacteroidia	Bacteroidales	Porphyromonadaceae	Porphyromonas		CRISPR Array			7 (34-38)	8 (37)	M4/NA/E	CTCACTATACATACACAGTCCGTAAGGCTATTAAGAC	L
2069595->2069305	-		NC_002950.2	Porphyromonas gingivalis W83	Bacteria	Bacteroidetes	Bacteroidia	Bacteroidales	Porphyromonadaceae	Porphyromonas		**Cas2**	pfam09827	CAS-I, CAS-II, CAS-III					
2072710->2069615	-	WP_005874916.1	NC_002950.2	Porphyromonas gingivalis W83	Bacteria	Bacteroidetes	Bacteroidia	Bacteroidales	Porphyromonadaceae	Porphyromonas	CRISPR-associated endonuclease Cas1	Cas6-**RT-Cas1**	mkCas0066, pfam00078, pfam01867	CAS-III; CAS-I, CAS-II, CAS-III					
2073532->2072771	-	WP_005874906.1	NC_002950.2	Porphyromonas gingivalis W83	Bacteria	Bacteroidetes	Bacteroidia	Bacteroidales	Porphyromonadaceae	Porphyromonas	type III-B CRISPR module RAMP protein Cmr6	**Cmr6gr7**	cd09661	CAS-III-B					
2073972->2073529	-	WP_005874927.1	NC_002950.2	Porphyromonas gingivalis W83	Bacteria	Bacteroidetes	Bacteroidia	Bacteroidales	Porphyromonadaceae	Porphyromonas	H	**Cmr5gr11**	mkCas0098	CAS-III-B					
2074670->2073969	-	WP_010956463.1	NC_002950.2	Porphyromonas gingivalis W83	Bacteria	Bacteroidetes	Bacteroidia	Bacteroidales	Porphyromonadaceae	Porphyromonas	type III-B CRISPR module RAMP protein Cmr4	**Cmr4gr7**	cd09682	CAS-III-B					
2075874->2074684	-	WP_005874912.1	NC_002950.2	Porphyromonas gingivalis W83	Bacteria	Bacteroidetes	Bacteroidia	Bacteroidales	Porphyromonadaceae	Porphyromonas	type III-B CRISPR module-associated protein Cmr3	**Cmr3gr5**	cd09656	CAS-III-B					
2077522->2075876	-	WP_005874937.1	NC_002950.2	Porphyromonas gingivalis W83	Bacteria	Bacteroidetes	Bacteroidia	Bacteroidales	Porphyromonadaceae	Porphyromonas	type III-B CRISPR-associated protein Cas10/Cmr2	**Cas10**	cd09680, cls000742	CAS-III, CAS-III					
2078966->2077515	-	WP_005874902.1	NC_002950.2	Porphyromonas gingivalis W83	Bacteria	Bacteroidetes	Bacteroidia	Bacteroidales	Porphyromonadaceae	Porphyromonas	H	**Cmr1gr7**	mkCas0086	CAS-III-B					
2079224->2078973	-	WP_005874934.1	NC_002950.2	Porphyromonas gingivalis W83	Bacteria	Bacteroidetes	Bacteroidia	Bacteroidales	Porphyromonadaceae	Porphyromonas	H	**Cmr1gr7**	mkCas0102	CAS-III-B					
**CAS-III-B**																			
259953->259611	-		NC_015571.1	Porphyromonas gingivalis TDC60	Bacteria	Bacteroidetes	Bacteroidia	Bacteroidales	Porphyromonadaceae	Porphyromonas		CRISPR Array			4 (35-43)	5 (37)	M4/NA/E	CTCACTATACATACACAGTCCGTAAGGCTATTAAGAC	NA
260644 -> 260354	-			Porphyromonas gingivalis TDC60	Bacteria	Bacteroidetes	Bacteroidia	Bacteroidales	Porphyromonadaceae	Porphyromonas		**Cas2**	pfam09827	CAS-I, CAS-II, CAS-III					
263759->260664	-	WP_013815267.1	NC_015571.1	Porphyromonas gingivalis TDC60	Bacteria	Bacteroidetes	Bacteroidia	Bacteroidales	Porphyromonadaceae	Porphyromonas	CRISPR-associated endonuclease Cas1	Cas6-**RT-Cas1**	mkCas0066, pfam00078, pfam01867	CAS-III; CAS-I, CAS-II, CAS-III					
264594->263821	-	WP_013815268.1	NC_015571.1	Porphyromonas gingivalis TDC60	Bacteria	Bacteroidetes	Bacteroidia	Bacteroidales	Porphyromonadaceae	Porphyromonas	type III-B CRISPR module RAMP protein Cmr6	**Cmr6gr7**	cd09661	CAS-III-B					
265028->264591	-	WP_013815269.1	NC_015571.1	Porphyromonas gingivalis TDC60	Bacteria	Bacteroidetes	Bacteroidia	Bacteroidales	Porphyromonadaceae	Porphyromonas	H	**Cmr5gr11**	mkCas0098	CAS-III-B					
265726->265025	-	WP_013815270.1	NC_015571.1	Porphyromonas gingivalis TDC60	Bacteria	Bacteroidetes	Bacteroidia	Bacteroidales	Porphyromonadaceae	Porphyromonas	type III-B CRISPR module RAMP protein Cmr4	**Cmr4gr7**	cd09682	CAS-III-B					
266951->265740	-	WP_013815271.1	NC_015571.1	Porphyromonas gingivalis TDC60	Bacteria	Bacteroidetes	Bacteroidia	Bacteroidales	Porphyromonadaceae	Porphyromonas	type III-B CRISPR module-associated protein Cmr3	**Cmr3gr5**	cd09656	CAS-III-B					
268602->266953	-	WP_013815272.1	NC_015571.1	Porphyromonas gingivalis TDC60	Bacteria	Bacteroidetes	Bacteroidia	Bacteroidales	Porphyromonadaceae	Porphyromonas	type III-B CRISPR-associated protein Cas10/Cmr2	**Cas10**	cls000742, cls000742	CAS-III, CAS-III					
270046->268595	-	WP_013815273.1	NC_015571.1	Porphyromonas gingivalis TDC60	Bacteria	Bacteroidetes	Bacteroidia	Bacteroidales	Porphyromonadaceae	Porphyromonas	H	**Cmr1gr7**	mkCas0086	CAS-III-B					
270304->270053	-	WP_005874934.1	NC_015571.1	Porphyromonas gingivalis TDC60	Bacteria	Bacteroidetes	Bacteroidia	Bacteroidales	Porphyromonadaceae	Porphyromonas	H	**Cmr1gr7**	mkCas0102	CAS-III-B					
**Unknown**																			
605->315	-	WP_036885016.1	NZ_JQZW01000017.1	Porphyromonas gingivicanis COT-022	Bacteria	Bacteroidetes	Bacteroidia	Bacteroidales	Porphyromonadaceae	Porphyromonas	CRISPR-associated endonuclease Cas2	**Cas2**	pfam09827	CAS-I, CAS-II, CAS-III					
3702->610	-	WP_036885018.1	NZ_JQZW01000017.1	Porphyromonas gingivicanis COT-022	Bacteria	Bacteroidetes	Bacteroidia	Bacteroidales	Porphyromonadaceae	Porphyromonas	CRISPR-associated endonuclease Cas1	Cas6-**RT-Cas1**	mkCas0091, pfam00078, pfam01867	CAS-III; CAS-I, CAS-II, CAS-III					
4479->3849	-		NZ_JQZW01000017.1	Porphyromonas gingivicanis COT-022	Bacteria	Bacteroidetes	Bacteroidia	Bacteroidales	Porphyromonadaceae	Porphyromonas		CRISPR Array			8 (35-41)	9 (37)	M4/NA/E	GCATCTATACAAAGACAGTCAGTAAGACTATTAAGAC	NA

RT-CRISPR-12 (CAS-IIIA/D, CAS-III-B)
Firmicutes (Clostridiales, Erysipelotrichales)

[a](Sub) Type/[b]Coordinates	Strand	Protein_id	Genome Partition ID	Strain	Domain	Phylum	Lineage (class)	Order	Family	Genus	[c]Cas gene (annotated)	[d]Cas gene/[e]Array (identified)	[d]Profiles	[d](Sub)Type-specificity	[e]Spacers (length)	[e]Repeats (length)	[f]Str.motif/Seq.family/Superclass	[f]Seq Repeats	[g]Orientation Confidence
**CAS-III-D**																			
1671184 -> 1672914	+	WP_008751392.1	NZ_GL622296.1	Lachnoanaerobaculum saburreum DSM 3986	Bacteria	Firmicutes	Clostridia	Clostridiales	Lachnospiraceae	Lachnoanaerobaculum	H	**Cas10**	mkCas0133	CAS-III					
1672862 -> 1675195	+	WP_008751393.1	NZ_GL622296.1	Lachnoanaerobaculum saburreum DSM 3986	Bacteria	Firmicutes	Clostridia	Clostridiales	Lachnospiraceae	Lachnoanaerobaculum	CRISPR-associated protein	**Csm3gr7-Csx10gr5**	mkCas0198, cd09700	CAS-III-A, CAS-III-D; CAS-III-D					
1675209 -> 1676684	+	WP_008751394.1	NZ_GL622296.1	Lachnoanaerobaculum saburreum DSM 3986	Bacteria	Firmicutes	Clostridia	Clostridiales	Lachnospiraceae	Lachnoanaerobaculum	CRISPR-associated protein	**Csm3gr7**	cd09726	CAS-III-D					
1676659->1677195	+	WP_008751394.1	NZ_GL622296.1	Lachnoanaerobaculum saburreum DSM 3986	Bacteria	Firmicutes	Clostridia	Clostridiales	Lachnospiraceae	Lachnoanaerobaculum	H	**Csx19**	cls001376	CAS-III-D					
1677198->1679108	+	WP_008751395.1	NZ_GL622296.1	Lachnoanaerobaculum saburreum DSM 3986	Bacteria	Firmicutes	Clostridia	Clostridiales	Lachnospiraceae	Lachnoanaerobaculum	CRISPR-associated RAMP family protein	**Csm3gr7**	cls000253, cls000253	CAS-III-A, CAS-III-D; CAS-III-A, CAS-III-D					
1679162->1680706	+	WP_040461790.1	NZ_GL622296.1	Lachnoanaerobaculum saburreum DSM 3986	Bacteria	Firmicutes	Clostridia	Clostridiales	Lachnospiraceae	Lachnoanaerobaculum	H								
1680720->1681025	+	WP_008751397.1	NZ_GL622296.1	Lachnoanaerobaculum saburreum DSM 3986	Bacteria	Firmicutes	Clostridia	Clostridiales	Lachnospiraceae	Lachnoanaerobaculum	ketoacid-CoA transferase								
1681997->1681122	-		NZ_GL622296.1	Lachnoanaerobaculum saburreum DSM 3986	Bacteria	Firmicutes	Clostridia	Clostridiales	Lachnospiraceae	Lachnoanaerobaculum		CRISPR Array			12 (35-44)	13 (30)	NA/NA/NA	GTAAGTACCTTACCTATAAGGAATGGAAAC	H
1684368->1682449	-	WP_008751399.1	NZ_GL622296.1	Lachnoanaerobaculum saburreum DSM 3986	Bacteria	Firmicutes	Clostridia	Clostridiales	Lachnospiraceae	Lachnoanaerobaculum	subtype I-B CRISPR-associated endonuclease Cas1	**RT-Cas1**	pfam00078, cd09722	CAS-III, CAS-I-B					
1684637->1684377	-	WP_008751400.1	NZ_GL622296.1	Lachnoanaerobaculum saburreum DSM 3986	Bacteria	Firmicutes	Clostridia	Clostridiales	Lachnospiraceae	Lachnoanaerobaculum	CRISPR-associated endonuclease Cas2	**Cas2**	COG1343	CAS-I, CAS-II, CAS-III					
1685706->1684689	-		NZ_GL622296.1	Lachnoanaerobaculum saburreum DSM 3986	Bacteria	Firmicutes	Clostridia	Clostridiales	Lachnospiraceae	Lachnoanaerobaculum		CRISPR Array			14 (33-48)	15 (30)	NA/NA/NA	GTAAGTACCTTACCTATAAGGAATGGAAAC	H
**CAS-III-D**																			
220701->219899	-		NZ_KQ959848.1	Lachnoanaerobaculum saburreum DNF00896	Bacteria	Firmicutes	Clostridia	Clostridiales	Lachnospiraceae	Lachnoanaerobaculum		CRISPR Array (Assembly Gaps)			>10 (34-47)	>11 (30)	NA/NA/NA	GTAAGTACCTTACCTATAAGGAATGGAAAC	M
221537->221893	+	WP_060932232.1	NZ_KQ959848.1	Lachnoanaerobaculum saburreum DNF00896	Bacteria	Firmicutes	Clostridia	Clostridiales	Lachnospiraceae	Lachnoanaerobaculum	H	**Csx20**	cls000118	CAS-I, CAS-III					
221920->222672	+	WP_060932233.1	NZ_KQ959848.1	Lachnoanaerobaculum saburreum DNF00896	Bacteria	Firmicutes	Clostridia	Clostridiales	Lachnospiraceae	Lachnoanaerobaculum	CRISPR-associated endoribonuclease Cas6	**Cas6**	COG1583	CAS-I, CAS-III, CAS-IV					
222714->224258	+	WP_060932234.1	NZ_KQ959848.1	Lachnoanaerobaculum saburreum DNF00896	Bacteria	Firmicutes	Clostridia	Clostridiales	Lachnospiraceae	Lachnoanaerobaculum	H								
224298->226022	+	WP_060932235.1	NZ_KQ959848.1	Lachnoanaerobaculum saburreum DNF00896	Bacteria	Firmicutes	Clostridia	Clostridiales	Lachnospiraceae	Lachnoanaerobaculum	H	**Cas10**	mkCas0133	CAS-III					
225964->228459	+	WP_060932236.1	NZ_KQ959848.1	Lachnoanaerobaculum saburreum DNF00896	Bacteria	Firmicutes	Clostridia	Clostridiales	Lachnospiraceae	Lachnoanaerobaculum	CRISPR-associated protein	**Csm3gr7-Csx10gr5**	mkCas0198, cd09700	CAS-III-A, CAS-III-D; CAS-III-D					
228473->229990	+	WP_060932237.1	NZ_KQ959848.1	Lachnoanaerobaculum saburreum DNF00896	Bacteria	Firmicutes	Clostridia	Clostridiales	Lachnospiraceae	Lachnoanaerobaculum	CRISPR-associated protein	**Csm3gr7**	cd09726	CAS-III-D					
229962->230495	+	WP_060932238.1	NZ_KQ959848.1	Lachnoanaerobaculum saburreum DNF00896	Bacteria	Firmicutes	Clostridia	Clostridiales	Lachnospiraceae	Lachnoanaerobaculum	H								
230498->232495	+	WP_060932239.1	NZ_KQ959848.1	Lachnoanaerobaculum saburreum DNF00896	Bacteria	Firmicutes	Clostridia	Clostridiales	Lachnospiraceae	Lachnoanaerobaculum	CRISPR-associated RAMP family protein	**Csm3gr7**	cls000253	CAS-III-A, CAS-III-D					
232543->234087	+	WP_060932254.1	NZ_KQ959848.1	Lachnoanaerobaculum saburreum DNF00896	Bacteria	Firmicutes	Clostridia	Clostridiales	Lachnospiraceae	Lachnoanaerobaculum	H								
234101->234406	+	WP_060932240.1	NZ_KQ959848.1	Lachnoanaerobaculum saburreum DNF00896	Bacteria	Firmicutes	Clostridia	Clostridiales	Lachnospiraceae	Lachnoanaerobaculum	ketoacid-CoA transferase								

234998->234502	-		NZ_KQ959848.1	Lachnoanaerobaculum saburreum DNF00896	Bacteria	Firmicutes	Clostridia	Clostridiales	Lachnospiraceae	Lachnoanaerobaculum		CRISPR Array			7 (34-40)	8 (30)	NA/NA/NA	GTAAGTACCTTACCTATAAGGAATGGAAAC	H
237380->235461	-	WP_060932241.1	NZ_KQ959848.1	Lachnoanaerobaculum saburreum DNF00896	Bacteria	Firmicutes	Clostridia	Clostridiales	Lachnospiraceae	Lachnoanaerobaculum	type I-B CRISPR-associated endonuclease Cas1	**RT-Cas1**	pfam00078, cd09722	CAS-III, CAS-I-B					
237649->237389	-	WP_060932242.1	NZ_KQ959848.1	Lachnoanaerobaculum saburreum DNF00896	Bacteria	Firmicutes	Clostridia	Clostridiales	Lachnospiraceae	Lachnoanaerobaculum	CRISPR-associated protein Cas2	**Cas2**	COG1343	CAS-I, CAS-II, CAS-III					
238029->237698	-		NZ_KQ959848.1	Lachnoanaerobaculum saburreum DNF00896	Bacteria	Firmicutes	Clostridia	Clostridiales	Lachnospiraceae	Lachnoanaerobaculum		CRISPR Array			4 (38-56)	5 (30)	NA/NA/NA	GTAAGTACCTTACCTATAAGGAATGGAAAC	NA
**CAS-III-B**																			
1255->524		WP_051592780.1	NZ_JMLH01000027.1	[Clostridium] saccharogumia DSM 17460	Bacteria	Firmicutes	Erysipelotrichia	Erysipelotrichales	Erysipelotrichaceae	Erysipelatoclostridium	CRISPR-associated endoribonuclease Cas6	**Cas6**	COG1583	CAS-I, CAS-III, CAS-IV					
1527->1267		WP_027089678.1	NZ_JMLH01000027.1	[Clostridium] saccharogumia DSM 17460	Bacteria	Firmicutes	Erysipelotrichia	Erysipelotrichales	Erysipelotrichaceae	Erysipelatoclostridium	CRISPR-associated endonuclease Cas2	**Cas2**	COG1343	CAS-I, CAS-II, CAS-III					
3476->1533		WP_051592781.1	NZ_JMLH01000027.1	[Clostridium] saccharogumia DSM 17460	Bacteria	Firmicutes	Erysipelotrichia	Erysipelotrichales	Erysipelotrichaceae	Erysipelatoclostridium	subtype I-B CRISPR-associated endonuclease Cas1	**RT-Cas1**	pfam00078, cd09722	CAS-III; CAS-I-B					
5592->3430		WP_027089680.1	NZ_JMLH01000027.1	[Clostridium] saccharogumia DSM 17460	Bacteria	Firmicutes	Erysipelotrichia	Erysipelotrichales	Erysipelotrichaceae	Erysipelatoclostridium	H								
6392->5601		WP_034864417.1	NZ_JMLH01000027.1	[Clostridium] saccharogumia DSM 17460	Bacteria	Firmicutes	Erysipelotrichia	Erysipelotrichales	Erysipelotrichaceae	Erysipelatoclostridium	type III-B CRISPR module RAMP protein Cmr6	**Cmr6gr7**	cd09661	CAS-III-B					
6651->6394		WP_027089682.1	NZ_JMLH01000027.1	[Clostridium] saccharogumia DSM 17460	Bacteria	Firmicutes	Erysipelotrichia	Erysipelotrichales	Erysipelotrichaceae	Erysipelatoclostridium	H	Cmr5gr11	mkCas0098						
7011->6703		WP_027089683.1	NZ_JMLH01000027.1	[Clostridium] saccharogumia DSM 17460	Bacteria	Firmicutes	Erysipelotrichia	Erysipelotrichales	Erysipelotrichaceae	Erysipelatoclostridium	H								
7819->6992		WP_027089684.1	NZ_JMLH01000027.1	[Clostridium] saccharogumia DSM 17460	Bacteria	Firmicutes	Erysipelotrichia	Erysipelotrichales	Erysipelotrichaceae	Erysipelatoclostridium	type III-B CRISPR module RAMP protein Cmr4	**Cmr4gr7**	COG1336	CAS-III-C					
8919->7819		WP_027089685.1	NZ_JMLH01000027.1	[Clostridium] saccharogumia DSM 17460	Bacteria	Firmicutes	Erysipelotrichia	Erysipelotrichales	Erysipelotrichaceae	Erysipelatoclostridium	H	Cmr3gr5	pfam09700						
10498->8912		WP_027089686.1	NZ_JMLH01000027.1	[Clostridium] saccharogumia DSM 17460	Bacteria	Firmicutes	Erysipelotrichia	Erysipelotrichales	Erysipelotrichaceae	Erysipelatoclostridium	H	**Cas10**	cd09679, cls00742	CAS-III; CAS-III					
11897->10488		WP_027089687.1	NZ_JMLH01000027.1	[Clostridium] saccharogumia DSM 17460	Bacteria	Firmicutes	Erysipelotrichia	Erysipelotrichales	Erysipelotrichaceae	Erysipelatoclostridium	H	**Cmr1gr7**	mkCas0086	CAS-III-B					
**Unknown**																			
52 ->562	+		CVRS01000124.1	Roseburia inulinivorans	Bacteria	Firmicutes	Clostridia	Clostridiales	Lachnospiraceae	Roseburia		CRISPR Array			7 (35-42)	8 (29)	NA/NA/A	GTTTTACCTTACCTATGAGGAATTGACAC	H
590 ->2491	+	CRL43259.1	CVRS01000124.1	Roseburia inulinivorans	Bacteria	Firmicutes	Clostridia	Clostridiales	Lachnospiraceae	Roseburia	H	**RT-Cas1**	pfam00078, cd09722	CAS-III; CAS-I-B					
2500 ->2760	+	CRL43258.1	CVRS01000124.1	Roseburia inulinivorans	Bacteria	Firmicutes	Clostridia	Clostridiales	Lachnospiraceae	Roseburia	H	**Cas2**	cd09725	CAS-I, CAS-II, CAS-III, CAS-V					
**Partial**																			
102556->102791	+		FCNR01000048.1	Eubacteriaceae bacterium CHKCI004	Bacteria	Firmicutes						CRISPR Array			3 (34-43)	4 (30)	NA/NA/NA	GTAGATAGCCCGATATAGAGGGCAATAAAC	NA
102882->103142	+	CVI70778.1	FCNR01000048.1	Eubacteriaceae bacterium CHKCI004	Bacteria	Firmicutes					cas2 2	**Cas2**	cd09725	CAS-I, CAS-II, CAS-III, CAS-V					
103163->105070	+	CVI70780.1	FCNR01000048.1	Eubacteriaceae bacterium CHKCI004	Bacteria	Firmicutes					cas1 2	**RT-Cas1**	pfam00078, cd09722	CAS-III; CAS-I-B					
105345->105569	+		FCNR01000048.1	Eubacteriaceae bacterium CHKCI004	Bacteria	Firmicutes						CRISPR Array			3 (34-40)	4 (29)	NA/NA/NA	GTAGATAGCCCGATATAGAGGGCAATAAA	H

Supplementary Table 2. List of 118 unique RT sequences associated with CRISPR-Cas systems described in this work.

RTCRISPR-Clade	Genome Partition ID	Accesion	Type of RT	Domain	Phylum	Class	*Species*
**1**	CP003362	AGB49377.1	RT	Archaea	Euryarcheota	Methanomicrobia	*Methanomethylovorans hollandica DSM15978*
**1**	CP009516	AKB78445.1	RT	Archaea	Euryarcheota	Methanomicrobia	*Methanosarcina Honorobensis HB-1*
**1**	CP009508	AKB36824.1	RT	Archaea	Euryarcheota	Methanomicrobia	*Methanosarcina siciliae C2J*
**1**	CP009504	AKB21960.1	RT	Archaea	Euryarcheota	Methanomicrobia	*Methanosarcina sp. WH1*
**1**	CP009505	AKB26318.1	RT	Archaea	Euryarcheota	Methanomicrobia	*Methanosarcina sp. MTP4*
**2**	NC_015388.1	WP_013707702.1	RT-Cas1	Bacteria	Proteobacteria	Deltaproteobacteria	*Desulfobacca acetoxidans DSM 11109*
**2**	NZ_JH724308.1	WP_007481073.1	RT-Cas1	Bacteria	Bacteroidetes	Bacteroidia	*Bacteroides salyersiae CL02T12C01*
**2**	NZ_JGDY01000030.1	WP_032556864.1	RT-Cas1	Bacteria	Bacteroidetes	Bacteroidia	*Bacteroides fragilis S6L3*
**2**	LAQJ01000315.1	KKO17867.1	RT-Cas1	Bacteria	Planctomycetes	Planctomycetia	*Candidatus Brocadia fulgida RU1 BROFUL*
**2**	JRYO01000185.1	KHE91657.1	RT-Cas1	Bacteria	Planctomycetes	Planctomycetia	*Candidatus Scalindua brodae RU1 SCABRO*
**2**	NZ_BAFH01000003.1	WP_007220853.1	RT-Cas1	Bacteria	Planctomycetes	Planctomycetia	*Candidatus Jettenia caeni*
**2**	JPDT01002544.1	KPA10619.1	RT-Cas1	Bacteria	Proteobacteria	Deltaproteobacteria	*Candidatus Magnetomorum sp. HK-1*
**2**	ATBP01000692.1	ETR69258.1	RT-Cas1	Bacteria	Proteobacteria	Deltaproteobacteria	*Candidatus Magnetoglobus multicellularis str. Araruama*
**2**	NZ_JAFP01000001.1	WP_025270209.1	RT-Cas1	Bacteria	Proteobacteria	Deltaproteobacteria	*Hippea sp. KM1*
**2**	NZ_JAIQ01000034.1	WP_046996094.1	RT-Cas1	Bacteria	Proteobacteria	Epsiloproteobacteria	*Arcobacter butzleri L348 isolate CHRB125*
**2**	NZ_FIZP01000001.1	WP_075539949.1	RT-Cas1	Bacteria	Proteobacteria	Epsiloproteobacteria	*Campylobacter fetus subsp. fetus RC20*
**2**	NZ_ANNG01000021.1	WP_021087740.1	RT-Cas1	Bacteria	Proteobacteria	Epsiloproteobacteria	*Campylobacter concisus UNSWCS*
**2**	NZ_CP012196	WP_005873073.1	RT-Cas1	Bacteria	Proteobacteria	Epsiloproteobacteria	*Campylobacter gracilis ATCC 33236*
**3**	CP_002199	ADN17996.1	RT-Cas1	Bacteria	Cyanobacteria		*[Scytonema hofmanni] UTEX 2349*
**3**	LJZR01000039	KPQ33062.1	RT-Cas1	Bacteria	Cyanobacteria		*Tolypothrix campylonemoides VB511288*
**3**	NZ_KI913950	WP_008312855.1	RT-Cas1	Bacteria	Cyanobacteria		*Noctoc sp. PCC7120*
**3**	NZ_AQPY01001269	WP_024971209.1	RT-Cas1	Bacteria	Cyanobacteria		*Calothrix PCC6303*
**3**	NC_016640	WP_014275551.1	RT-Cas1	Bacteria	Cyanobacteria		*Calothrix PCC7507*
**3**	NZ_CACA01000266	WP_007355619.1	RT-Cas1	Bacteria	Cyanobacteria		*Mastigocladus laminosus UU774*
**3**	CP_003633	AFZ22158.1	RT-Cas1	Bacteria	Cyanobacteria		*Anabaena cylindrica PCC7122*
**3**	CP003660	AFZ61061.1	RT-Cas1	Bacteria	Cyanobacteria		*Cyanothece PCC7822*
**3**	NZ_JXIJ01000035	WP_044448019.1	RT-Cas1	Bacteria	Cyanobacteria		*Microcoleus sp. PCC7113*
**3**	ANNX02000012.1	KYC43938.1	RT-Cas1	Bacteria	Cyanobacteria		*Scytonema hofmannii PCC7110*
**3**	NZ_JXCB01000011	WP_041039832.1	RT-Cas1	Bacteria	Cyanobacteria		*[Oscillatoria] sp. PCC 6506*
**3**	NZ_ALWD01000002	WP_029630506.1	RT-Cas1	Bacteria	Cyanobacteria		*Arthrospira platensis NIES-39*
**3**	NC_019751	AFY99362.1	RT-Cas1	Bacteria	Cyanobacteria		*Microcystis aeruginosa PCC 7005*
**3**	NC_003272	WP_010995638.1	RT-Cas1	Bacteria	Cyanobacteria		*Phormidesmis priestleyi Ana ITZX*
**3**	NC_019682	AFY33126.1	RT-Cas1	Bacteria	Cyanobacteria		*Leptolyngbya sp. PCC 6406*
**4**	NZ_JMLA01000001.1	WP_027150711.1	RT-Cas1	Bacteria	Proteobacteria	Gammaproteobacteria	*Methylobacter tundripaludum*
**4**	CT573071.1	CAJ74578.1	RT-Cas1	Bacteria	Planctomycetes	Planctomycetia	*Candidatus Kuenenia stuttgartiensis*
**4**	NC_015388.1	WP_013706262.1	RT-Cas1	Bacteria	Proteobacteria	Deltaproteobacteria	*Desulfobacca acetoxidans DSM 11109*
**4**	AZHX01001441.1	ETX03376.1	RT-Cas1	Bacteria	Nitrospinae	Tectomicrobia	*Candidatus Entotheonella sp. TSY2*
**4**	NC_011060.1	WP_012509117.1	RT-Cas1	Bacteria	Chlorobi	Chlorobia	*Pelodictyon phaeoclathratiforme BU-1*
**4**	NC_008639.1	WP_011745868.1	RT-Cas1	Bacteria	Chlorobi	Chlorobia	*Chlorobium phaeobacteroides DSM 266*
**5**	NZ_JH976538.1	WP_017302244.1	RT-Cas1	Bacteria	Cyanobacteria		*Nodosilinea nodulosa PCC 7104*
**5**	NZ_JH993797.1	WP_006515493.1	RT-Cas1	Bacteria	Cyanobacteria		*Leptolyngbya sp. PCC 7375*

**5**	AP017367.1	BAU44853.1	RT-Cas1	Bacteria	Cyanobacteria		*Leptolyngbya sp. O-77*
**5**	NZ_BAWS01000034.1	WP_030008160.1	RT-Cas1	Bacteria	Cyanobacteria		*Synechococcus sp. NKBG042902*
**5**	NZ_ALWB01000016.1	WP_009625650.1	RT-Cas1	Bacteria	Cyanobacteria		*Pseudanabaena biceps PCC 7429*
**5**	NC_019678.1	WP_015120902.1	RT	Bacteria	Cyanobacteria		*Rivularia sp. PCC 7116*
**5**	NC_019753.1	WP_015201683.1	RT	Bacteria	Cyanobacteria	Oscillatoriophycideae	*Crinalium epipsammum PCC 9333*
**5**	NC_011738.1	WP_012599795.1	RT	Bacteria	Cyanobacteria	Oscillatoriophycideae	*Cyanothece sp. PCC 7424*
**6**	NZ_JXYA01000100.1	WP_046007427.1	RT-Cas1	Bacteria	Proteobacteria	Gammaproteobacteria	*Pseudoalteromonas rubra strain S2471*
**6**	NZ_BAOI01000066.1	WP_038884984.1	RT-Cas1	Bacteria	Proteobacteria	Gammaproteobacteria	*Vibrio rotiferianus CAIM 577*
**6**	NC_005140.1	WP_011152750.1	RT-Cas1	Bacteria	Proteobacteria	Gammaproteobacteria	*Vibrio vulnificus YJ016*
**7**	NC_014365.1	WP_013258512.1	RT-Cas1	Bacteria	Proteobacteria	Deltaproteobacteria	*Desulfarculus baarsii DSM 2075*
**7**	NZ_KB908056.1	WP_019960649.1	RT-Cas1	Bacteria	Proteobacteria	Alphaproteobacteria	*Woodsholea maritima DSM 17123*
**7**	CP001312.1	ADE85031.1	RT-Cas1	Bacteria	Proteobacteria	Alphaproteobacteria	*Rhodobacter capsulatus SB 1003*
**7**	AKZI01000198	EJW09347.1	RT-Cas1	Bacteria	Proteobacteria	Alphaproteobacteria	*Rhodovulum sp. PH10*
**7**	NC_013854.1	WP_012973664.1	RT-Cas1	Bacteria	Proteobacteria	Alphaproteobacteria	*Azospirillum sp. B510*
**7**	NZ_AP014800.1	WP_060836241.1	RT-Cas1	Bacteria	Proteobacteria	Alphaproteobacteria	*Rhodovulum sulfidophilum DSM 2351*
**7**	NC_016586.1	WP_014188713.1	RT-Cas1	Bacteria	Proteobacteria	Alphaproteobacteria	*Azospirillum lipoferum 4B*
**7**	LLVV01000015.1	KQB14190.1	RT-Cas1	Bacteria	Proteobacteria	Alphaproteobacteria	*Rhodobacter capsulatus strain A52*
**7**	NZ_KB908004.1	WP_019956891.1	RT-Cas1	Bacteria	Proteobacteria	Alphaproteobacteria	*Loktanella vestfoldensis DSM 16212*
**7**	AKZI01000170.1	EJW09481.1	RT-Cas1	Bacteria	Proteobacteria	Alphaproteobacteria	*Rhodovulum sp. PH10*
**7**	NC_014664	WP_049779315.1	RT	Bacteria	Proteobacteria	Alphaproteobacteria	*Rhodomicrobium vannielii ATCC 17100*
**7**	NZ_LPZR01000092.1	WP_062763150.1	:as1 (RT DV muta	Bacteria	Proteobacteria	Alphaproteobacteria	*Tistrella mobilis MCCC 1A02139*
**7**	CP003237.2	AFK55773.1	RT-Cas1	Bacteria	Proteobacteria	Alphaproteobacteria	*Tistrella mobilis KA081020-065*
**8a**	NZ_JQMT01000001	WP_038137810.1	Cas6-RT-Cas1	Bacteria	Proteobacteria	Gammaproteobacteria	*Thiomicrospora sp. Milos-T1*
**8a**	NC_012997.1	WP_015817555.1	Cas6-RT*	Bacteria	Proteobacteria	Gammaproteobacteria	*Teredinibacter turnerae T7901*
**8a**	NZ_KB906812	WP_019606016.1	Cas6-RT-Cas1	Bacteria	Proteobacteria	Gammaproteobacteria	*Teredinibacter turnerae T8412*
**8a**	AVFR01000074	ESQ17084.1	Cas6-RT-Cas1	Bacteria	Proteobacteria	Gammaproteobacteria	*uncultured Thiohalocapsa sp. PB-PSB1*
**8a**	AVFR01001361	ESQ08042.1	Cas6-RT-Cas1	Bacteria	Proteobacteria	Gammaproteobacteria	*uncultured Thiohalocapsa sp. PB-PSB1*
**8a**	CVPF01000361	CRI67871.1	Cas6-RT-Cas1	Bacteria	Proteobacteria	Gammaproteobacteria	*Thiocapsa sp. KS1*
**8a**	NZ_AMZO01000034	WP_007469744.1	Cas6-RT-Cas1	Bacteria	Proteobacteria	Gammaproteobacteria	*Photobacterium marinum AK15*
**8a**	LQIY01000027	KUI97421.1	Cas6-RT-Cas1	Bacteria	Proteobacteria	Gammaproteobacteria	*Vibrio sp. MEBiC08052 200.VRK.1_28*
**8a**	NZ_KE383828	WP_028302067.1	Cas6-RT-Cas1	Bacteria	Proteobacteria	Gammaproteobacteria	*Oceanospirillum beijerinckii DSM 7166*
**8a**	NZ_KK211138	WP_028883449.1	RT-Cas1	Bacteria	Proteobacteria	Gammaproteobacteria	*Teredinibacter turnerae 1133Y.S.0a.04*
**8a**	CP002583	ADZ89953.1	Cas6-RT-Cas1	Bacteria	Proteobacteria	Gammaproteobacteria	*Marinomonas mediterranea MMB-1*
**8a**	NZ_LDOV01000030	WP_047875592.1	RT-Cas1	Bacteria	Proteobacteria	Gammaproteobacteria	*Photobacterium aphoticum DSM 255995*
**8a**	NZ_JRWP01000004	WP_038188758.1	RT-Cas1	Bacteria	Proteobacteria	Gammaproteobacteria	*Vibrio sinaloensiss T08*
**8a**	NZ_LBGR01000002	WP_055043549.1	Cas6-RT-Cas1	Bacteria	Proteobacteria	Gammaproteobacteria	*Vibrio metoecus YB9D03 YB09_D03*
**8b**	NZ_KB907634.1	WP_019672870.1	RT-Cas1	Bacteria	Proteobacteria	Gammaproteobacteria	*Psychrobacter lutiphocae DSM 21542*
**8b**	WP_015334627.1	NC_020055	Cas6-RT-Cas1	Bacteria	Proteobacteria	Deltaproteobacteria	*Desulfovibrio hydrothermalis AM13*
**8b**	WP_027180402.1	NZ_KE387016.1	Cas6-RT-Cas1	Bacteria	Proteobacteria	Deltaproteobacteria	*Desulfovibrio bastinii DSM 16055*
**8b**	JDVG02000521	KFB71594.1	Cas6-RT-Cas1	Bacteria	Proteobacteria	Betaproteobacteria	*Candidatus Accumulibacter sp. BA-91*
**8b**	JQOA01000247	KFZ44108.1	RT(partial)-Cas1	Bacteria	Proteobacteria	Deltaproteobacteria	*Smithella sp. D17*
**8b**	LAQJ01000147.1	KKO19838.1	RT-Cas1	Bacteria	Plantomycetes	Plantomycetia	*Candidatus Brocadia fulgida RU1 BROFUL*
**8b**	NZ_BAFN01000001	WP_052565451.1	T-Cas1 (truncate	Bacteria	Plantomycetes	Plantomycetia	*Candidatus Brocadia sinica JPN1*
**8**	WP_012910084.1	NC_013720.1	Cas6-RT-Cas1	Bacteria	Plantomycetes	Plantomycetia	*Pirellula staleyi DSM 6068*

**8**	KJR40057.1	JZJI01001005.1	Cas6-RT-Cas1	Bacteria	Nitrospirae	Nitrospira	*Candidatus Magnetoovum chiemensis CS-04 SAG2981*
**9**	NC_010175.1	YP_001636958.1	RT	Bacteria	Chloroflexi	Chloroflexia	*Chloroflexus aurantiacus J-10-fl*
**9**	NC_009523.1	WP_011957721.1	RT	Bacteria	Chloroflexi	Chloroflexia	*Roseiflexus sp. RS-1*
**9**	NC_009767.1	WP_012121172.1	RT	Bacteria	Chloroflexi	Chloroflexia	*Roseiflexus castenholzii DSM 13941*
**10**	NZ_AXCZ01000003	WP_052104813.1	RT-Cas1	Bacteria	Actinobacteria	Actinobacteria	*Cellulomonas bogoriensis 69B4*
**10**	NZ_JWIO01000001	WP_052914180.1	RT-Cas1	Bacteria	Actinobacteria	Actinobacteria	*Frankia coriariae strain BMG5.1*
**10**	LRQV01000138	KXK58998.1	RT-Cas1	Bacteria	Actinobacteria	Actinobacteria	*Micromonospora rosaria strain DSM 803*
**10**	NZ_ANAS01000036	WP_017559367.1	RT-Cas1	Bacteria	Actinobacteria	Actinobacteria	*Nocardiopsis baichengensis YIM 90130*
**10**	NZ_ANBB01000038	WP_020380191.1	RT-Cas1	Bacteria	Actinobacteria	Actinobacteria	*Nocardiopsis potens DSM 45234*
**10**	NZ_CP012590	WP_053587381.1	RT-Cas1	Bacteria	Actinobacteria	Actinobacteria	*Actinomyces sp. oral taxon 414 strain F0588*
**10**	AQHZ01000010	ENO18597.1	RT-Cas1	Bacteria	Actinobacteria	Actinobacteria	*Actinomyces cardiffensis F0333*
**10**	NZ_KK037166	WP_052396493.1	RT-Cas1	Bacteria	Actinobacteria	Actinobacteria	*Kutzneria sp. 744*
**10**	CP001737	ACV77640.1	RT-Cas1	Bacteria	Actinobacteria	Actinobacteria	*Nakamurella multipartita DSM 44233*
**10**	AP012204	BAK34153.1	RT-Cas1	Bacteria	Actinobacteria	Actinobacteria	*Microlunatus phosphovorus NM-1*
**10**	NZ_KE384022	WP_051209229.1	RT-Cas1	Bacteria	Actinobacteria	Actinobacteria	*Propionicicella superfundia DSM 22317*
**10**	NZ_KB290831	WP_006063846.1	RT-Cas1	Bacteria	Actinobacteria	Actinobacteria	*Corynebacterium durum F0235*
**11**	NZ_JQZW01000017.1	WP_036885018.1	Cas6-RT-Cas1	Bacteria	Bacteroidetes	Bacteroidia	*Porphyromonas gingivicanis COT-022*
**11**	NZ_JQJE01000003.1	WP_039443024.1	Cas6-RT-Cas1	Bacteria	Bacteroidetes	Bacteroidia	*Porphyromonas gulae OH3161B*
**11**	NC_002950.2	WP_005874916.1	Cas6-RT-Cas1	Bacteria	Bacteroidetes	Bacteroidia	*Porphyromonas gingivalis W83*
**11**	NC_015571.1	WP_013815267.1	Cas6-RT-Cas1	Bacteria	Bacteroidetes	Bacteroidia	*Porphyromonas gingivalis TDC60*
**12**	CVRS01000124.1	CRL43259.1	RT-Cas1	Bacteria	Firmicutes	Clostridia	*Roseburia inulinivorans*
**12**	FCNR01000048.1	CVI70780.1	RT-Cas1	Bacteria	Firmicutes		*Eubacteriaceae bacterium CHKCI004*
**12**	NZ_JMLH01000027.1	WP_051592781.1	RT-Cas1	Bacteria	Firmicutes	Erysipelotrichia	*[Clostridium] saccharogumia DSM 17460*
**12**	NZ_GL622296.1	WP_008751399.1	RT-Cas1	Bacteria	Firmicutes	Clostridia	*Lachnoanaerobaculum saburreum DSM 3986*
**12**	NZ_KQ959848.1	WP_060932241.1	RT-Cas1	Bacteria	Firmicutes	Clostridia	*Lachnoanaerobaculum saburreum DNF00896*
**GII intron ML**	CP000875.1	YP_001544692	RT	Bacteria	Chloroflexi	Chloroflexia	*Herpetosiphon aurantiacus DSM 785*
**-**	NZ_AATI01000019	ZP_01425057.1	RT	Bacteria	Chloroflexi	Chloroflexia	*Herpetosiphon aurantiacus ATCC 23779*
**-**	FP929056.1	CBL28738	RT	Bacteria	Synergistetes	Synergistia	*Fretibacterium fastidiosum*
**-**	JDST02000051	KFB76584.1	RT-Cas1	Bacteria	Proteobacteria	Betaproteobacteria	*Candidatus Accumulibacter sp. SK-02*
**-**	NZ_AEWG01000021	WP_009855610	RT-Cas1	Bacteria	Proteobacteria	Betaproteobacteria	*Rubrivivax benzoatilyticus JA2*
**-**	CP001312.1	ADE85032.1	RT	Bacteria	Proteobacteria	Alphaproteobacteria	*Rhodobacter capsulatus*
**-**	CP003051.1	AGA89197.1	RT	Bacteria	Proteobacteria	Gammaproteobacteria	*Thioflavicoccus mobilis 8321*
**Retron**	NC_015512.1	WP_013769055.1	RT	Bacteria	Bacteroidetes	Saprospiria	*Haliscomenobacter hydrossis DSM 1100*

Supplementary Dataset 1. MSA of RT sequences used in Figure 1.

>W_[Haliscomenobacter_hydrossis] 332661943
------------------------------YQD-------------------VIKK------QNY--------
HQFSMPK---K-----------------D---G---T-------LRAITAPGNKLKFLQKTLSHAFQ---
LTYLNK-IPDCVHGY---VPGDIHPEVGTRHILSNAQVH---------FG---------------------
AKY---LLNLDIDDFF-PNLDSLRILDAL-------------------RYW-YPEMPQATQELLVGI---C--
----
--CY-------NN------------

-------------------------------QLPMGAPSS-PVLSNMCVH-QMD-------------------
-----LAL-LT--------------------------------------LC---------AQ-----------
--------------------------HQVTYSRYVDDMSF----------SSSSD----
NLLALEPALIEALQ-SFGFTINQAKRMHFG----P--EG---------------------------
PKIVTGLIM-
>DS_fid|22599913|locus|VBIMetSil55537_2322| [Methylocella silvestris BL2]
-IETRRE--------------------VEAFEA---NSQS--NLKRIAD---QLLH------RKF---
IFPAAKGVPIQK-AKG-K---------------R---G---D-------IRPLVVAKVEARIVQRAIHDVLI-
--EVPSIRRYVRTPYSFGGVRKEKDDSVSAVPAAIDAAMAA----I----GD-------------G-------
FSY---YIRSDITAFF-TKIPKSAVAALV--SDAVG------------HQSEFMDLFR--RAIHVEL------
---ENMA--
--R-LARTV-NAFPIYD------------

-----------------------------I-GVAQGNSLS-PLLGNILLY-DFD-------------------
-----QQM---NG----------N-----------
--------------------------PDAVCLRYIDDFII--FA------KTQQL----AE-
NMFQKAIHILA-SHGMSVAKHKTVKGL----V--RD---------------------------KFEFLGIEF-
>DS_gi|82702063|ref|YP_411629.1 [Nitrosospira multiformis ATCC 25196]
PTIRTEI--------------------EQ-FSL---SLEK--NLRRIAD---QLRE------KRY---
VFSQSYGVAVKK-KNN-------------------------P----S-KKRPIVISPIPNRIVQRALLDVVQ-
EIPSVRAKLDSGFNFGG---IAEIG-----VPQAILKAYKT----A----LE---------------------
KPY---FIRTDICAFF-DNIPRSQALEII--TSASK------------DDD-FNTLLT--QATTTELSNLI--
---TLGR--
--D-KELFP-L----EG------------

-----------------------------K-GVAQGSCLS-PVLCNLLLD-DFD-------------------
-----KKM-NA--------------------------------------RG---------IV-----------
------------------------------CIRYIDDFIL--FA------PSESK----AF-
KAFASASAFLE-KLNLSVYDPRHSPDK-AEHGVSNK---------------------------GFEFLGCSV-
>DS_gi|71735515|ref|YP_277063.1 [Pseudomonas syringae pv. phaseolicola 1448A]
SSDEIK---------------------RD-AEEFESRLPD--SLVEIQR---SLSK------QTF---
TFLQQTGVAQKK---P-----------------G---G---K-------
ARPLVLAPIPNRVVQRALLDVLQRRVRFVRKVLDTPTSYGG---IPTKR-----VAMAISDARDA----M---
-RN-------------G-------ARF---HIRSDIPAFF-TKINKDRVQDLL-RSH--I--N---------
CDA-TLKLLD--LAITTDL---ANIDDLRR-Q---
---G---------L-
NEIF--P----IG--
--IEGVAQGSPLS-
PLLANIYLA-DFD------------------------VAMNAD------------------------------
---------G---------IT---CLRYIDDFLL-
-LG------ESLSN----VD-RAFNRALKTLD-KIGLSAYDPRVDKVK----A--SRGSTDK-----------
-----------GFDFLGCNV-
>PF_WP_051592781.1 [[Clostridium] saccharogumia]

GIDGVML--------------------SQ-LKE---YLIL--NWQDIEN---QLIL------GTY---
EPNIVQVYELLS---K-----------------K---G---K-------VREIYKFTIIDNFIQKAVSLILQ-
--DKLDCL-LSDNNYSF---RKGKG-----TIDVIRKGLEL----I----EE-------------G-------
YEY---IVEIDIKKYF-ENIDHVLLSKML--FD--I-ID---------DKV-LISLIM--KYQNCLI---Q--
---K--D--
------------------------------------G------------KIK--R----KN------------
----
-----------------------------K-GLITGSSIS-PVISNLYLM-DLD-------------------
-----RQY---LE----------Y-----------
--------------------------N---YIRYCDNIYI--FI------NNKDD----GL-
TLINDISKCLKDKYKLEINQNKTSITH----Y--L----------------------------SKRMLGYYF-
>PF_WP_008751399.1 [Lachnoanaerobaculum saburreum]
GIDGLYL--------------------SE-LRD---DWNI--NGERYLS---LLRK------GKY---
KPGIVQIYEIVN---Y-----------------T---G---K-------RRSISSFNSIDRLVLRCLATSLE-
--KYYDSI-FSSSSFAF---RPGLG-----VDKAVATFANN----L----NT-------------G-------
LTR---VAIIDIKHYF-DSIPIDRLEMIL--KR--I-ID---------DNV-LLSLFH--NLLYCRI---S--
---E--E--
------------------------------------N------------VIK--T----KS------------
----
-----------------------------K-GILQGSPIS-PFLGNLYLS-LLD-------------------
-----TQL-ES--------------------------------------MH----------V-----------
--------------------------S---FCRYCDDIAM--FF------ASFEE----AK-
ETYTKVYDILKNDLEMDINPQKSGIYE----G--I----------------------------KQNYLGYSF-
>PF_WP_060932241.1 [Lachnoanaerobaculum saburreum]
EIDGLHL--------------------SE-LRD---YWDI--NGERYLS---MLRA------GKY---
KPGIVQIYEIIN---Y-----------------T---G---K-------RRSISSFNSVDRLILRCLATSLE-
--KYYNSI-FSACSFAF---RPGLG-----VDKAVAAFVSN----L----NK-------------G-------
LDK---VVVIDIKHYF-DSIPIDRLEMIL--KR--I-ID---------DKI-LLSLFH--KFLYCRI---S--
---E--E--
------------------------------------N------------IIK--T----KN------------
----
-----------------------------K-GILQGSPIS-PFLGNLYLS-LLD-------------------
-----TQL-ES--------------------------------------MQ----------I-----------
--------------------------Q---FCRYCDDITM--FF------SSFDE----AK-
EAYTKVSNILKNDLEMDIHTQKSGIYE----G--I----------------------------KQNYLGYNF-
>PF_CRL43259.1 [Roseburia inulinivorans]
GLDGVKL--------------------SE-LRA---YWET--NGKKIKE---SIFN------GTY---
KVGAVEQRQIVN--RK---------------------G---K-------KRTISLMNSIDRFIFRALYQKMA-
--SEWEKQ-FSQYSYAY---QNNKG-----VLTAVEQAAKY----M----EE-------------G-------
KDW---SVELDIQNFF-DNINHSIIISKL--KA--G-IE---------DVR-VLDLLI--AYLTCTL---L--
---D--D--
------------------------------------H-------------AFH-Q----ME------------
----
-----------------------------Q-GVLQGGPLS-PLLANVYMN-ELD-------------------
-----HYM-EK--------------------------------------QG---------YS-----------
------------------------------FGRFGDDINI--YC------STYEE----AT-
VAFSDVTARMEKIEQLPLNHGKTGIFK-------GI---------------------------NRKYLGYRF-
>PF_CVI70780.1 [Eubacteriaceae bacterium CHKCI004]
GIDGIFV--------------------KD-FEE---YWIL--NGQKILK---QVMN------GVY---
MVSPVQLREIIM---P-----------------T---G---K-------HRIIAHYTCTDRLITRILAESLQ-
--KEVDDS-LSEYSYAY---RKQRG-----VIKAVEQAAAY----M----QA-------------G-------
KIW---VLELDIENYF-NNINLTLMEEKI--RE--I-IL---------DKN-LFSLME--QYLRCEV---M--
---E--E--
------------------------------------E---------Y-TKTY--I----KD------------

----
-----------------------------K-GLVQGCSLS-PVLSNIYLN-KLD-------------------
-----QQM-EK--------------------------------------EG----------L-----------
--------------------------S---FCRFGDNINI--YF------YNKLE----AA-
EWYAKIKAIIENEFDLHLNIRKSGIYL----G--V----------------------------NRIFLGYSF-
>W_[Teredinibacter_turnerae] 254788194
GSGANAIAAPRTSKLQLPPAPSVPGEPDDYPPELWPRARVQDHLHTLAS---VVLN------QQY---
RVPPLRGWRTWH---A-----------------D---G---R-------ESVAMVPPFWDRVLQQAVKQLLE-
--SLVASL-QVPAHKTVPHQSPQQT-----AKNGIKSADRK--ARQQALQQG---------------------
YRW---AFRATPDDLL-LSANRQRAYDRL--RA--L-CY---------DDP-LLDLIA--DWLAAPV------
---AL-E--
------------------------------------G---------S-------E----RQ-----------
----
-----------------------------RAGLPLGSPLS-PLLADLLLD-DFG-------------------
-----RDM-AA--------------------------------------AG---------FY-----------
------------------------------LLRTSHEVTV--LS------RSQKH----AQ-
HTAALAYDTVQ-------RREPARVQN----I--EH---------------------------RVNFSGYVF-
>PF_WP_036885018.1 [Porphyromonas gingivicanis]
QCDTSLY--------------------QTWLSSIAQDTHSPLKKAELENLLEALHS------LSY---
IPSVAHAIHIPK---S-----------------D---G---S-------YRTLSIPSPIDLYLQRNLINVLY-
--PIIDKT-NSPQSYAY---RKGKG-----ALEAIKQVELL--KRK----LG---------------------
KKYY--VVRCDIDNFF-DSIPIEQLMGMF--QN--I-TR---------DPL-LSRMVR--LWIKSGV---V--
---DN-K--
------------------------------------S---------H---FH--P----HL------------
----
-----------------------------Q-GLPQGSPLS-PLLSNFYLT-DTD-------------------
-----RYI---SN----------N-----------
--------------------------ITEYFIRYADDILL--FI------PEHSD----PL-
SSLQALSNHLKNQKKLSLN--KDFIVT----E--INS--------------------------EFSFLGISF-
>PF_WP_039443024.1 [Porphyromonas gulae]
-VNDALY--------------------RKWLSSLAADRDLPMAEAERQDLLEALRV------CSY---
IPQPYHSVNIPK---G-----------------D---G---S-------YRQLHIPSAVDLHLQRSLAGILY-
--PITESL-SIAQSYAY---RKGKG-----AVAAIRKVQHL--LDS----LD---------------------
ENYT--VVRCDIDNFF-DSIPVPSLLQKV--LR--T-TE---------DPL-LTRMLS--LWMKSGV---V--
---DR-T--
------------------------------------Q---------Q---YT--P----AS------------
----
-----------------------------S-GIPQGSPLA-PLLSNLYLE-DTD-------------------
-----RYI---AG----------H-----------
--------------------------ITTEFIRYADDLLL--FL------PERAD----PL-
KALQDLSEHLKYRKGLKLN--RDFVVS----S--IKS--------------------------SFSFLGITF-
>W_[Porphyromonas_gingivalis] 34541577
----RKW--------------------LSSLAADRDLPMAEAERQDLLE---ALRI------CSY---
IPQPYHSVNIPK--GD---------------------G---S-------YRQLHIPSAVDLHLQRSLAGILY-
--PITESL-SIAQSYAY---RKGKG-----AVAAVRRVQHL----L----DS---------------------
LDENHTVVRCDIDNFF-DSIPVPSLLQKV--QRTTE------------DPF-LTRMLS--LWMKSGV---V--
---DRKQ--
--QYARA----SS------------

-------------------------------GIPQGSPLA-PLLSNLYLE-DTD-------------------
-----RYI---AG----------H-----------
--------------------------ITTEFIRYADDLLL--FL------PEKVD----PL-
NALQDLSEHLKYRKGLKLNRDFVVSSI-------KS---------------------------SFSFLGITF-
>PF_WP_013815267.1 [Porphyromonas gingivalis]

```
DTLYRKW--------------------LSSLAADRDLPMAETERQDLLE---ALRI------CSY---
IPQPYHSVNIPK---G-----------------D---G---S-------YRQLHIPSAVDLHLQRSLAGILY-
--PITESL-SIAQSYAY---RKGKG-----AVAAVRRVQHL--LDS----LD---------------------
ENYT--VVRCDIDNFF-DSIPVPSLLQKV--QR--T-TE---------DPL-LTRMLS--LWMKSGV---V--
---DR-K------------------------------------------------------------------
----------------------------------------------Q---QYA-P----AS------------
-------------------------------------------------------------------------
-----------------------------S-GIPQGSPLA-LLLSNLYLE-DTD-------------------
-----RYI-----------------------------------------AG----------H-----------
--------------------------ITTEFIRYADDLLL--FL------PEKVD----PL-
NALQDLSEHLKYRKGLKLNRD--FVVS----S--IKS--------------------------SFSFLGITF-
>PF_KJR40057.1 [Candidatus Magnetoovum chiemensis]
GPDTITT--------------------DD-LKK-----AGDQFLDKLKN---NIVN------GNY---
KQGKTKQYRIPK---N-----------------D---D---T-------FRYIYVLNTTDRLVHKTIADYIS-
--PIVDNI-ISNSAYAY---RRGLN-----TKGAANALNNA----L----KE-------------G-------
YTS---GIKADISEFF-DSINISALSMMI--DS--L-FP---------FEP-LADFIN--GILENNT---R--
------D------------------------------------------------------------------
------------------------------------G---------I--------------------------
-------------------------------------------------------------------------
-----------------------------K-GILQGSPLS-PLLSNLYLT-RFD-------------------
-----SDM-ES--------------------------------------KG---------FF-----------
--------------------------K---LIRYADDFVL--LL------KTASS----YE-
ETIKHVEDSLS-TLGLKLKPEKTTEIT----Q--GK---------------------------AINFLGYVI-
>PF_WP_019672870.1 [Psychrobacter lutiphocae]
AIEEVLE--------------------RHELEPVVDDKGRIMQVDALTE---LIYTQLST--GVY---
APKPTRAIFVPK---P--------------------KG---G-------KRCIEELEQVDMMVHRLVFNSIA-
--KTIESY-QSPLSLGY---RKGYS-----RQMARDKVQAL----I----DS-------------G-------
FGW---VVEADIESFF-DNVPFERLWQRLATILPQR------------ELQ-TIALIK--KLMQVGY--TV--
---SNAS------------------------------------------------------------------
------------------------------------G---------T-VVKEH-L----RF------------
-------------------------------------------------------------------------
-----------------------------K-GLMQGSPLS-PVLANLYLA-MLD-------------------
-----EQI-NA--------------------------------------EH---------FA-----------
------------------------------FVRYADDVLM--FC------RSEAD----AN-
TTLAWLDQHLS-ELGLNLSLSKTAITA----V--NN---------------------------GFEFLGYRF-
>PF_WP_053587381.1 [Actinomyces sp. oral taxon 414]
GLLQRQT--------------------RL-IAG---DLDR--FLAELSA---SLRG------GTY---
LPAPLLRADIPK-RAP---------------------G---Q-------TRVLHIPTIRDRVVERAVVNAVA-
--HDADRI-MSPCSFAY---RTGIG-----TDDAVHHLATL----R----DD-------------G-------
YRH---VLRTDVEDYF-PNLDVEDALTVL--APVVG------------CPR-TIDLIR--LIARPRR---A--
------R------------------------------------------------------------------
------------------------------------G-------------ERR-T----RS------------
-------------------------------------------------------------------------
-----------------------------R-GIAQGSCLS-PLLANLVLN-DVD-------------------
-----HAL-ND--------------------------------------AG---------YG-----------
------------------------------YARFADDIVV--CA------PARDD----LL-
AARELLGSLVA-AHGLNLNEEKTAMTT----F--DE---------------------------GFCFLGVDF-
>PF_ENO18597.1 [Actinomyces cardiffensis F0333]
GVLQRQS--------------------KR-IIE---NADE--FLNQLSA---LLRN------GTY---
EPEPLNRVDIPK---G-E---------------H---G---K-------TRTLNIPTIHDRIVERAIVDTIA-
--FTADLV-QSSCSFAY---RTGIG-----VDDAVHHVATL----R----EE-------------G-------
YQY---VLRTDIEDFF-PHVNLEHALEAL--PE--S-LQ---------ERD-LLALLRIVALPRRAH------
-------------------------------------------------------------------------
------------------------------------G-------------QRR-A----RS------------
```

-----------------------------R-GVAQGSTLS-PLLANLSLT-RFD-------------------
-----HDI-CD--------------------------------------AG----------Y-----------
--------------------------G---YARFADDIVV--CS------PREQD----IL-
DAIELLSDLAA-AHGLKLNQDKTIMTT----F--DE---------------------------GFCYLGVDF-
>PF_WP_020380191.1 [Nocardiopsis potens]

-------------------------MRADIADCFEQ----I---------------------------PRW--
-PVVTRVKELV-PDAEPCLLIQHL--IA--R------------DAT-GPAARR--VWSGRRR-----------
----
--SR-----------------

--------------------------GLYQGSALS-PALADLYLG-AFG------------------------
KAM-LW--------------------------------------AG---------RQ----------------
-------------------------VLRYADDFAI--PA------GSRTE----AE-SALTTAEDVPA-
EWGPELNGAKSRIVS----F--DE---------------------------GVDFLGRTV-
>PF_WP_017559367.1 [Nocardiopsis baichengensis]
GVKSTAV--------------------QE-FEK---GALR--RLLDISE---QLRE------GTY---
APEPVTAFEVPK--PS---------------------G---E-------ARLLGIGTVGDRVVERAVLAVIE-
--PCIDPV-LLPWSFAY---RKGLG-----VPDAVQALAEA----R----ES-------------G-------
STW---VLRADFADCF-ETIPRWPVITRL--HE--L-VP---------DAE-LCLLVQ--HFIQRKS------
---RG-P--
------------------------------------G---------A-RRLRP-G----SG------------
----
-----------------------------R-GLHQGSALS-PLLSNLYLD-SFD-------------------
-----RAL-LQ--------------------------------------RG---------RQ-----------
------------------------------VLRYGDDFAV--PS------ESRHA----AE-
QALAQATEAAR-EWGLELNAAKSQIVS----F--DE---------------------------GVRFLGRTV-
>PF_WP_052914180.1 [Frankia sp. BMG5.1]
GQPDSEV--------------------DA-FEA---NAAR--NLDELGT---VLAA------GEW---
QASPVRRVDLPK---P-S-------------------G---G-------VRVLGVPRLVDRIVERALLRVLD-
--PVIDPL-LLPWSFAY---RRGLG-----ARDALAALAEA----R----DS-------------G-------
MTW---VARSDIRDCF-PSIPQWEVLRRL--RE--V-VD---------DER-IIHLVG--VLLDRPV---A--
----
------------------------------------G---------G-RTDPK-N----RG------------
----
-----------------------------L-GLHQGSALS-PLLSNLYLN-AFD-------------------
-----RAM-LR--------------------------------------AG---------FR-----------
------------------------------VIRYSDDFAI--PT------TGRVA----AE-
QALVSASTELE-DLRLEINSGKSHVVS----F--DE---------------------------GVRFLGEVT-
>PF_KXK58998.1 [Micromonospora rosaria]

MTRVSIPK---P-----------------D---G---G-------IRSLAIGAIEDRIVERAVLDVLD---
PVVDPT-LSPWSFAY---RRGLG-----VRDAVRALAEA----R----ES-------------G-------
LAF---VVRCDIDDCF-DSIPRWPLLRRL-REL----VS---------DAE-LVALVE--RLVGRPV------
------T--
------------------------------------G-------------ERA-S----GG------------
----
-----------------------------R-GLHQGGSLS-PLLANLYLD-TFD-------------------
-----RAL-MR--------------------------------------HG---------HR-----------
------------------------------VVRYGDDIAI--SV------PDRPT----GL-
RVLDLADAEAE-ALSLRLNTDDRQVIA----F--DE---------------------------GVPFCGQVV-
>PF_WP_052104813.1 [Cellulomonas bogoriensis]

GRVPVSV--------------------RR-FER---GVAA--SLVRLSG---ELSS------GRY---
QPSRVSEVSLRT--GS---------------------G---S-------ERVLRIGAVVDRVVERSLLNALT-
--PVIDPL-LSPFAFGF---RRGLG-----VKDAVAALARA----R----DE-------------G-------
STH---VLRSDIAAAF-DSVPRARAVQAL--SR--L-VP---------DRR-VCDVVA--SLLARLDDYGL--
------E--
------------------------------------G------------------------------------
----
-----------------------------V-GIAQGSAVS-PLLLNLYLL-PFD-------------------
-----EAL-MA--------------------------------------NG---------FT-----------
------------------------------PLRYADDIAV--PA------MSESQ----AQ-
SAAQDVAHQLE-CLGLACSAPKTSIRS----F--DE---------------------------GVHFLGVTL-
>PF_WP_006063846.1 [Corynebacterium durum]
GNLAPSI--------------------LK-FQE---DAEE--KILRLSE---ALLD------GSY---
KPYQFTEVDIET---N---------------------G---K-------ERTLHIPAVQDRIVARAILATTT-
--SRIDPL-LGASAFGY---RPGLG-----VADAVQAVVDA----R----EA-------------G-------
LKW---VLRTDVDDCF-PSLSPDIAFDRF--TQAVH------------DTD-ITDVVE--QLLGRTV---G--
------N--
------------------------------------G-------------KMRGT----TL------------
----
-----------------------------P-GLPLGCPLS-PVLMNLVLV-DLD-------------------
-----DAL-NA--------------------------------------AG---------FT-----------
------------------------------VVRYADDIVV--VG------ESKEE----LE-
DAARFCQRILR-SFNMQLGDDKTDIMT----F--DD---------------------------GFAFLGEDF-
>PF_ACV77640.1 [Nakamurella multipartita DSM 44233]

MAVLVPG---P---------------------------------TR------------------------
PDLPGQGVKVDQWSY---------------TTLVDLTEG--------------------------------
LRW---VCRCDIDNCF-PSIPKDRLRRKL--TA--LFQG---------DPT-LLGILT--RLLARPA------
------G--
------------------------------------G-------------SPA-E----AL------------
----
-----------------------------P-GLPQGSPLS-PLWANLILA-DFD-------------------
-----DAV-AR--------------------------------------TG---------FP-----------
------------------------------LVRYSDDMVI--AA------ADRAE----AW-
EAMRVAHDAAA-GIEMSLGADKSAVMS----F--DE---------------------------GFTFLGEDF-
>PF_WP_051209229.1 [Propionicicella superfundia]
--MP---
--DRIVARAILDTAT---
PFVDPE-LGHCAFAY---RPGLG-----VADAVQAIARQ----R----EE-------------G-------
LGW---VLRTDIDECF-PTLPVDLAHRRL--AA----LV---------DDDDLASVLT--ALSARPY------
----
--R-TATRA-L----RA------------

-----------------------------VTGLPQGCPLS-PVLANLVLV-DVD-------------------
-----RAL-LD--------------------------------------RG---------YA-----------
------------------------------PVRYGDDIAI--PC------ANEDD----AW-
EAARVTSEAAE-RLDMSLGSDKTHAMS----F--TE---------------------------GFVFLGEEF-
>PF_WP_052396493.1 [Kutzneria sp. 744]
DQLSAGV--------------------RT-FGD---EADQ--RLAGLAE---QLAG------GMY---
LPGVLTELVMVT---E-----------------D---G---G-------QRVLRVPAVRDRVVERALLSVLS-
--PRLDPL-LGPASFGF---RPGLG-----VVDAVQALARL--RD------E-------------G-------
FGW---VLRTDLHDCF-PSVDLRRVRRLL-EVL--T-S----------DGD-LLGVLD--LLLARAA------
---RR-P--
------------------------------------G---------E---QTL-R----PA------------

----
-----------------------------H-GLPQGSSLS-PLLANLVLE-DFD-------------------
-----DRM-RH--------------------------------------AG----------F-----------
--------------------------P---LVRYADDIAV--LA------SSERE----AW-
EAARVASAAAK-EIGMTLGADKTEIMS----F--DG---------------------------GFCFLGEDF-
>W_[Microlunatus_phosphovorus] 336116789
GVERFA---------------------ED-PKA---------ELDELGE---QLRT------GTY---
RPRDLTEVVIDD---G---------------------G---G-------SRTLHIPAVRDRVVERSLLNVVT-
--PWVDPV-LGFTSYAY---RPGLG-----VADAVQALVTL----R----SE-------------G-------
LGW---VLRTDVDDCF-PSVPVDHARRLL--GA--L-VP---------DAD-LLAIVD--LLLARAA---V--
---RPGR--
------------------------------------G-------------RGV-M------------------
----
-----------------------------R-GLAQGCALS-PLLTNLVLT-ALD-------------------
-----DAL-LD--------------------------------------EG---------FA-----------
------------------------------VLRYADDICV--AT------ETRDD----AW-
EAARIATAALE-VLGMELGADKTEVMS----F--DE---------------------------GFSFLGEDF-
>PF_WP_013707702.1 [Desulfobacca acetoxidans]
GLDGVSV--------------------QS-FGD---QAAS--HLEDLRQ---ALQA------GNY---
TPEPHQRIKVPK---L-----------------DGS-G---E-------LRPLSLPTIKDKIVQEAVRRIIE-
--PLFEPE-FLPCSYAY---RPGQG-----PRRAIGRAIHY--LEH----DK---------------------
CRW---AVHADFDKFF-DTLDHEVLLRRL--QE--K-IK---------ELP-VLKLVR--MWLRTGS---I--
---GA-K--
------------------------------------G---------T---YD--D----AD------------
----
-----------------------------L-GVGQGGILS-PLLSNIYVH-PLD-------------------
-----VYL-TD--------------------------------------KG---------HR-----------
------------------------------YIRYADNVLI--LA------DSQPR----GT-
EGLNDLIYFSQEMLKLRLNPEAKPLRH----V--AD---------------------------GFTFLGIHF-
>PF_KKO17867.1 [Candidatus Brocadia fulgida]
GLDGVEI--------------------DD-QHT---DADK--MVSALIK---ELRT------GAY---
VPVPYARGAIPKFDEQ---------------------N---Q-------WRKISLPSVRDKVVQQAFVEALG-
--PVFNKT-FLDCSYAY---REGKG-----PVKAIKRVEHI----LHT--HH---------------------
IRW---VTTMDIDNFF-DTMDHDIFIGEF--TK--K-VA---------EPE-ILQLVR--LWLKAGC------
---ISAR--
------------------------------------G-------------DWIEP----YD------------
----
-------------------------------GIAQGAVVS-PLFSNIYLH-PLD-------------------
-----CFA---IG----------------------
--------------------------NNCLYVRYSDNLIV--LS------ETKET----LY-
LWYEQLKSFLEDRLRLRLNEDPYPFKD----K--ER---------------------------GFVFLGIFF-
>PF_KHE91657.1 [Candidatus Scalindua brodae]
GLDNVTV--------------------ES-FGN---RLDQ--HISKLQK---EIME------HRY---
VPKPLKSIHVPK---Y-----------------NKE-N---E-------WRGLALPSVSDKVVQAALLQVVE-
--PLGEKL-FMDSSYAY---RKGKG-----HYKAIRRVEHC--LGN----RK---------------------
KSW---VVHRDIDNFF-DTLNYDRLIDQF--SA--L-VD---------GEPVMTELVA--LWCRTGL---V--
---EA-G--
------------------------------------G---------R---WR--G----VQ------------
----
-----------------------------S-GIRQGNIIS-PLLSNLYLH-PLD-------------------
-----EFA-AR--------------------------------------LR---------ID-----------
------------------------------WVRYCDDYLI--LC------DSRKD----AI-
SADRLIKEYLKEPLCLKLNNSGLSPCH----I--DE---------------------------GFTFLGVSF-
>PF_WP_007220853.1 [Candidatus Jettenia caeni]

GLDGITV--------------------EE-FGH---RLDQ--HITKLQK---DIRE------RRY---
IPQPAAVTYIPK---F-----------------NEE-N---E-------WRELGLPSVADKVVQAAMLEVVE-
--PLAEKM-FLDCSYAY---RPGTG-----HYKAIRRVENS--LNN----RK---------------------
KTW---VVQRDIDNFF-DTVDHNRLMEQF--SA--L-VQ---------GEPTMVELVA--LWCRMGL---V--
---EK-N--
------------------------------------G---------R---WR--N----VQ------------
----
-----------------------------A-GIRQGGVIS-PLLANLYLH-PLD-------------------
-----VFA-TK--------------------------------------LG---------VD-----------
------------------------------WIRYADDYVI--LG------ESQEE----VV-
SSDVQIVEFLKDSLGLMLNRDESSPKH----I--DE---------------------------GFTFLGVRF-
>PF_KPA10619.1 [Candidatus Magnetomorum sp. HK-1]
GVDGVTI--------------------SS-FNA---NLEV--NLSELSN---QILT------NQY---
TPEPLQAAHIPK---P---------------------G---K----S-EKRQLGLPSLKDKIVQSSLASILS-
--DFYEIH-FSNCSYAY---RPGKG-----SVKAIGRVRDF----L----NR-------------K-------
NYW---IASVDIDNFF-DSVDHEICTSIL--KE--Q-IS---------DQS-IIRLIS--LYFSSGM---I--
---KFDQ--
--WQ--D----TE------------

-----------------------------I-GIPQGGAIS-PVISNIYLN-KLD-------------------
-----HFL-HT--------------------------------------LN---------AF-----------
------------------------------FVRYADDIIL--FS------NTQQS----LS-
ETYQKTNEFLNKKLNLKLNALDNPIIN----V--SK---------------------------GFSFLGIYF-
>PF_ETR69258.1 [Candidatus Magnetoglobus multicellularis str. Araruama]
GIDGVSI--------------------SE-FET---ARDK--NLQELSQ---QILY------SQY---
TPEPLQAVQIPK---P---------------------G-KTE-------KRQLGLPSLKDKIVQSSLASLLS-
--DFYDPL-FSNCSYAY---RPQKG-----SVRAIGRVKDF----L----NR-------------K-------
NHW---AAPVDIDNFF-DTVNHETCISIL--QD--K-IS---------DID-IIRLIR--LYFSSGK---I--
---QF-D--
--K---WQ--D----TI------------

-----------------------------I-GIPQGGALS-PVLSNVYLN-ELD-------------------
-----QYL---HA----------------------
--------------------------IQANFVRYADDIIL--FA------NTRQF----LL-
DFYEKTRHFLESKLQLKLNQTSHPVMS----M--EK---------------------------GFAFLGIYF-
>PF_WP_025270209.1 [Hippea sp. KM1]
RVSLKRE--------------------IHLPEK---------EIENLFR---ALQN------STY---
IPEPPQKIELKK---H---------------------D---K-------IRPITIASKKDKIVQALLHEYLT-
--ELFDSS-FSDKSYAY---RPNKG-----PLKAVNRTFDY----I----KR-------------G-------
EKY---VLKTDIKDFF-ETIDHSLLICML--KE--K-IK---------DDS-LIDLIM--MYIKIGTVKNL--
---EYED--
---HN------------

-----------------------------L-GVHQGNIIS-PILSNIYLD-RMD-------------------
-----KFL-ER--------------------------------------HG---------FN-----------
------------------------------FVRFADDFVV--FA------KTHDR----IE-
LIHRNLKRFLK-VYKLGLNEEKTYITT----T--DS---------------------------GFAFLGAYF-
>PF_WP_046996094.1 [Arcobacter butzleri]
GLDNISY--------------------IE-FKQ---NFTS--QIKELIE---TILK------GTY---
SPEPLKKIEIQK---E---------------------D---S----L-EKRPIALSSIKDKLVQRVLYKALN-
--DYFDET-FSNKSYAY---RKDKS-----TLNAINRVGQF----I----QE-------------Q-------
NHF---ILKTDIDNFF-ESINHDKLLTIL--DK--H-IQ---------DKS-IIRLIS--LFLQIGS---F--
---KEFD--
--YFE-H----ED------------

-------------------------------GVHQGDILS-PLLSNIYLD-LMD-------------------
-----KWL-EK--------------------------------------YD---------IF-----------
------------------------------FVRYADDFVV--FS------KKEDE----LK-
TIKENLEKFLE-SLDLKFGIDKTYFTT----I--QK---------------------------GFSFLGVYF-
>PF_CZE46369.1 [Campylobacter fetus subsp. fetus]
GIDNLSE-----------------------LNE---HFIH--KLK---Q---SCLN------QTY---
VPEPVLQKLIPK---------------------SDG-E---N-------YRKLAISSLKDKLIQKVLANELA-
--WYFDKH-FSDKSYAY---RPGKS-----YKNAIFRLRDF--L-R----VK---------------------
PYF---VIKSDIKDCF-ESINHSKLVALL--AK--Y-IK---------DKR-VLNLVE--IWIKNGI---F--
---N--R--
------------------------------------Q---------T---YI--K----HS------------
----
-----------------------------KFGIHQGDVLS-PLLANIYLN-QMD-------------------
-----KFL-ET--------------------------------------NN---------EI-----------
------------------------------FIRYADDFVI--LA------DDEKF----VQ-
AKINSLKTFLST-IDLSLKDTKTAIYS----P--TQ---------------------------SFEFLGVSF-
>PF_WP_021087740.1 [Campylobacter concisus]
GLDELSM--------------------DELCTE--------AFFAELKD---EILN------LSY---
SPQPLKRAFIPK---E---------------------N---K----D-EFRKLAIPSLKDKFTQNILIGELS-
--SYFDKG-FSNRSYAY---RSGKS-----YSNAIFRARDF----C----LT---------------------
HDF---VLKTDIKDFF-ENINHEKLLEIL--RSNIK------------DTR-IIRLIE--LWIKNGI---F--
---EHFD--
--YTS-H----TK------------

-------------------------------GVHQGDVLS-PLLSNIYLD-QMD-------------------
-----KFL-EH--------------------------------------SS---------IE-----------
------------------------------FVRYADDFVL--FF------GSREA----CE-
QALAGLKDFLV-TINLSLNEAKTSLHD----K--DS---------------------------EFTFLGVNF-
>PF_WP_005873073.1 [Campylobacter gracilis]
GFDGLSA--------------------DDICSG--------EFYAELKS---EIFS------LSY---
SPQPLKRAFIPK--EA-----------------KD------E-------LRKLAVPSLKDKFVQNILTRELS-
--GYFDKS-FSNRSYAY---RNGKS-----YANAIYRARDF--FQI---------------------------
FSF---AVKTDIKDFF-ENIDHEKLLEIL--RA--N-IR---------DAR-IIRLIE--LWIKNGI---F--
---E---
--R-FDYRA-H----TK------------

-------------------------------GVHQGDVLS-PLLSNIYLN-QMD-------------------
-----KFL-EN--------------------------------------SG----------V-----------
--------------------------E---FVRYADDFVM--FF------ASYEA----AE-
MRLARLKDFLK-TISLSLNEAKTSIHG----K--DS---------------------------EFVFLGVSF-
>PF_WP_027180402.1 [Desulfovibrio bastinii]
GAAASRL--------------DRDNDFSASLERDYGSIGN--AVFAMRD---HILN------GEF---
QCSPAAPFEINK---P--------------------LG---G-------RRTLGTFSSEDSLAQKLLHRLLS-
--PVLDRM-FEHSSVGF---RKGRS-----REDAKRMIQQA----I----RQ-------------G-------
CRY---VFESDIDSFF-DDIDRSTMLRKLRGVLPQA------------DKM-TFRALE--SCINAGL------
------E--
--NEVDS----TK------------

-------------------------------GLVQGSSLS-PLLSNLYLD-SVD-------------------
-----ERM-DE--------------------------------------HG---------YR-----------
------------------------------FIRYADDFVV--LA------HSEDE----WR-
KACEDMQDSLE-PLGLQLKEGKTHISC----I--DP---------------------------GFKFLGIEL-
>W_[Desulfovibrio_hydrothermalis]_(2) 436839745

GAAASKI--------------NRDSDLSSELERDYGSIEK--AVFEMRD---RILM------GEF---
TCSAAVPFEMHK---P--------------------YG---G-------SRIIGTCPPEDTLTQKLLHQLLS-
--PVMDRM-FEHSSVGF---RKGRS-----REDAKRMIRQA----I----RE-------------G-------
CRY---VFESDIDSFF-DEIDRPTMLRKLQDALPQA------------DHM-TFKALK--SCVNAGL---V--
------D--
--EDRQD----AK------------

-------------------------------GLVQGSSLS-PLLSNLYLD-GVD-------------------
-----ERM-EE--------------------------------------LG---------YR-----------
------------------------------FIRYADDFVV--LA------RSKEE----CR-
KAYEDMRLTLA-PLGLSLKEQKTRISN----I--DP---------------------------GFRFLGIDL-
>PF_KFB71594.1 [Candidatus Accumulibacter sp. BA-91]
DLAVRLS--------------------NTPQAP---DLHE--LAVELAQ---NLRE------GA----
APLPFQAIRVPR--SD---------------------G---R-------LRQFETPAARDLVILNHLTRLLS-
--EPFDRL-FSVHSIGY---RKGHS-----REDAVERVRAA----I----AE-------------G-------
CTH---VLESDISDFF-PSVDLKRLLARLDDVLPRR------------DVR-LRQTLA--AYLGAGW------
---RYGE--
------------------------------------G-------------SVQ-A----RN------------
----
-----------------------------R-GLPLGSPLS-PLLANLYLD-SFD-------------------
-----SQLGAT-------------------------------------VPG---------VR-----------
------------------------------LIRYADDFII--LT------ESEAA----AR-
ALLDTARDAAA-ALGLALNLEKTAIRP----L--SD---------------------------GFDFLGIRF-
>PF_KFZ44108.1 [Smithella sp. D17]
--Y---
TPAPNTAFLIKK--KS---------------------G---V-------DRMVEQIALKDLILQQYLLKTIG-
--NEFERI-FEPESIGF---RKGIS-----RQRAVEMVQAA----L----KA-------------G-------
YQF---IIESDVDDFF-PSVDLKILTGLL--DRYLP-QE---------DHR-IKELLT--KTIHNGY---V--
---L--N--
------------------------------------G-------------QYHER----VR------------
----
-------------------------------GVAQGSPLS-PMLANLYLD-YFD-------------------
-----ETI---KG----------------------
--------------------------WPVRLIRYADDFII--LT------RTKEE----AE-
EYLSRTESCLS-EIGLKIKKEKTGIKH----I--RE---------------------------GFRFLGIKF-
>PF_KKO19838.1 [Candidatus Brocadia fulgida]
ALQALSE--------------------TEKYPF-----DENQYAENLFQ---LIVS------NGY---
LPTPHIAFTIKK--KS---------------------G---V-------DRVVEQLSFRDLIVQQYLLKVIS-
--TVFDRF-FEAESIGF---RKGVS-----RQRSIGMIQSA----I----AE-------------G-------
YQC---VIESDIEDFF-PSVDLDILEHLLDCSIPQN------------DVC-LKNILL--KLIRNGF---I--
---L--N--
------------------------------------G-------------TYYER----RK------------
----
-------------------------------GLAQGGPLS-PILANLYLD-SFD-------------------
-----EQI-KR--------------WGLASHDEDAGTDHAKGGAGNKTAGG--------NAS-----------
--------------------------RGVKLIRYADDFII--LT------RTKEE----AE-
GVLSDTESYLS-TLGLKIKKEKTAIRS----L--RD---------------------------GFHFLGIRF-
>PF_WP_052565451.1 [Candidatus Brocadia sinica]
MVEQIPF--
---RDLIVQQYLLKIIS---
APFDRF-FEAESIGF---RRGVS-----RQRSIEIIQAA----I----AE-------------G-------
YQY---VIESDIEDFF-PSVDLNILAHLLDSYIPQN------------DSC-LKKILL--KFIKNGY---I--
---L--N--
------------------------------------G-------------VYHER----VK------------

----
-------------------------------GLAQGSPLS-PILANLYLD-SFD-------------------
-----EQI-KQ------------------------------WGLLSPDEHGETASDSKDTPS-----------
------------------RAAPAHTPRGVKLVRYADDFII--LT------KTKKD----AE-
DVLSETEAYLS-KLGLKIKKEKTAIRS----M--KD---------------------------GFQFLGIRF-
>W_[Pirellula_staleyi] 283778924
ALDQAAS--------------------KWDLGG--------GEVQSAAR---DLVK------GTY---
QPQPCFRLDIPK--SN---------------------G---D-------RRQLAIPSRLDRVLQRSILDVIA-
--PALELF-FEESSFAY---RRGLG-----RHTAARHLSQA----F----TD-------------G-------
YRW---ALHADFFDFF-DTIDHKLLRRRL--AA--Y-LA---------DPS-LVEVIM--RWVETGA------
----
--PH-------PD------------

-----------------------------H-GIPTGAPLS-PILANLFLD-QFD-------------------
-----EAM-HS--------------------------------------VG---------RR-----------
------------------------------LVRYADDFVV--LF------RDQSE----AQ-
AVISEVRQAAE-SLRLELNRDKTHTLH----L--AT---------------------------SFDFLGLHF-
>PF_WP_038137810.1 [Thiomicrospira sp. Milos-T1]
-IEKKPT--------------------IDKLTE---RTHS--QINSALG---QIIK------QDY---
NAPAMQGFTIPK--KD---------------------G---S-------ERLLAVSPLYDRVLQKAAAIILT-
--PGLDAL-MAQGSYGY---RKGLS-----RQQVRYEIQNA----Y----RQ-------------G-------
YHW---VYESDIEDFF-DAVNRKQLLNRL--QSLFG------------KDP-IWKQLE--DWLGQEI------
---HYQE--
--TIVER----TP------------

-----------------------------HTGLPQGSPLS-PVLANFVLD-DFD-------------------
-----SDM-EL--------------------------------------HG---------FK-----------
------------------------------MIRFADDFII--LC------KSRHE----AE-
LAALGVQHSLK-QVSLDINRDKTHIVE----L--SQ---------------------------GFRFLGYLF-
>W_[Marinomonas_mediterranea] 326793969
DTDEEHH--------------------DA-IDE---------LLTKLYVSRERIFK------REF---
TPSQLHSVEIEK---P--------------------EG---G-------TRLLSVPNWHDRTLQKAVTECLG-
--NTLEHI-WMKHSYGY---RKGHS-----RLQARDQINQY----I----QQ-------------G-------
YEW---VLESDIESFF-DSVNWLNLEQRL--KLLLP------------NEP-LVPLLM--QWVSAAK------
---QTED--
------------------------------------E-------------QTL-A----RH------------
----
-----------------------------N-GLPQGAPIS-PILANLLLD-DLD-------------------
-----QDM-IA--------------------------------------KG---------HQ-----------
------------------------------IVRYADDFVL--LF------KSKAA----AE-
SALDDIITALK-EHHLAINLEKTRIVE----A--SQ---------------------------GFRYLGYLF-
>PF_WP_028883449.1 [Teredinibacter turnerae]
SFNITAT--------------------E--FRT---------TLYQQLA---AIRA------CRY---
HPHPLVPVTIAK--KD---------------------G---T-------DRFLAVPPVGDRALQRVVTAQLS-
--AELDPL-FIQHSFGY---RKGYS-----RQGARDAINQA----I----RA-------------G-------
YGW---ILESDIDSFF-DSVAWSQMATRL--RLFMG------------QDP-LVDLIM--QWLQTPV---Q--
---ETPA--
--ASAPA----PR------------

-----------------------------CAGLPQGAPIS-PLLANLLLD-DFD-------------------
-----QDM-IV--------------------------------------QG---------MK-----------
------------------------------LVRFADDFVL--LF------KHQQQ----AQ-
QALPRVVQSLA-EHGLALKPEKTRIVS----A--QQ---------------------------GFRYLGYLF-
>PF_WP_047875592.1 [Photobacterium aphoticum]

-SETQK---------------------QH-TLT---------QLRQQCA---QLLE------GTF---
TAPTLQQVDIDK---D-D-------------------G---G-------TRTLSIPPWQDRVLQKAVASLLN-
--EAFDPL-WKHQSYGY---RKGRS-----RFNAKDAINDA----I----RQ-------------G-------
YEW---ALESDVDSFF-DSVCWTNLAARL--HL--L-FP---------SDP-LVPVIM--NWVKAPIR-TP--
------D--
------------------------------------G---------D-E-IPR-------T------------
----
-----------------------------Q-GLPQGSPLS-PLLANLILD-DFD-------------------
-----GDM-LA--------------------------------------LD----------Y-----------
--------------------------Q---LVRYADDFVL--LF------TSQQQ----AQ-
QALPHVIASLN-EHGLTLKARKTHIVE----A--KK---------------------------GFRYLGFLF-
>PF_WP_038188758.1 [Vibrio sinaloensis]
SLDSQFS--------------------EQERNQ---------YKNKVIGLSHTILA------GDY---
KAPVLTQVEIDK--SD---------------------G---G-------VRTLSIPPLADRILQKAIARPLA-
--VSLDGL-WKTHSYGY---RKDLS-----RHDAKFAINQA----I----QQ-------------G-------
YEW---VLESDVDSFF-DNVDWRNLQTRL--KLLLP------------NDF-LVDVIM--AWVKAPV------
---KTPS--
------------------------------------G-------------QILER----TQ------------
----
-------------------------------GLPQGSPLS-PLLANLVLD-DFD-------------------
-----ADM-LA--------------------------------------LD---------YK-----------
------------------------------LIRYADDFVL--LF------KKQSE----AQ-
MALDHVIASLN-EHGLNIKAKKTQIVH----A--NK---------------------------GFRYLGFWF-
>PF_WP_055043549.1 [Vibrio metoecus]
SLDNPLS--------------------EQQQRE---------VLQSVMTGSECLIH------QRY---
PVPTLQQVEIEK--EE---------------------G---G-------TRTLSIPPLIDRILQKAVARPLA-
--ASLEGL-WKSHSYGY---RSGLS-----RHDAKLAINQA----I----QN-------------G-------
YEW---ILESDVESFF-DNVDWHNLETRL--TL--L-LP---------NDA-LVDTIM--AWVKAPI---K--
---TV-T--
------------------------------------G---------E---YQ--Q----RQ------------
----
-----------------------------Q-GLPQGSPLS-PLLANLILD-DFD-------------------
-----ADM-LA--------------------------------------LD----------Y-----------
--------------------------Q---LVRYADDFVL--LF------KTEQQ----AQ-
AALHRVIDSLN-EHGLKIKAQKTHIVH----A--KT---------------------------GFRYLGFWF-
>PF_WP_019606016.1 [Teredinibacter turnerae]
ECDLSQY--------------------EC-NEETDAEGDQAELPPTLLKRANALAQ------GRY---
DVPPLRGVIIPK--TD---------------------G---E-------WRALAVAPFFDAVLQRAVAQILA-
--PSLDRV-MDNRSYGY---RRGRS-----RLDAKEQIQLA----Y----RN-------------G-------
ARW---VLEADIEDFF-DSVAFSLVAQRL--RALFH------------QDP-INEAIL--AWLSAPV------
---DY-D--
------------------------------------G-------------LRL-Q----RK------------
----
-----------------------------A-GLPQGSPLS-PVLANLLLD-DFD-------------------
-----SDM-RK--------------------------------------AG---------FN-----------
------------------------------CLRFADDFVV--VC------QSREE----AE-
RAWQRAASSLN-EHGLFLAENKTRVIS----F--ER---------------------------GFRFLGYLF-
>PF_ESQ17084.1 [uncultured Thiohalocapsa sp. PB-PSB1]
GVEWIDA-------------DEQEPDAQDGAEAEADELAA--PIEDLTRAIGHLQE------GKY---
RVPELRGYLLPK--RD---------------------G---G-------LRPLAVPPLRDRVLQRAVQQTLG-
--RGIEPL-FSSGSHGY---RPGHS-----RITAADAIRAA---------WA------------QG-------
YRW---VYESDVRDFF-DSVDLQRLRERL--EAIYG------------DDP-VVAAVL--GWMRAPV------
----
--RFRGERIERRN------------

-------------------------------GLPQGSPLS-PLMANLMLD-DFD-------------------
-----SDM-QA--------------------------------------AG---------FR-----------
------------------------------LIRFADDFIV--LC------KDPEE----AR-
RAGEAARASLA-EQGLALHPDKTRITA----M--ED---------------------------GFRYLGYLF-
>PF_ESQ08042.1 [uncultured Thiohalocapsa sp. PB-PSB1]
PIDPDDP--------------------ES-MPDPEAEEALADRLEAIGE---RLRA------MRY---
QAPALKGVVIRD---P--------------------DG---D-------LRALAIPPFWDRVAQRAVNDCIT-
--PACDLL-MSEASHGY---RRGRS-----RHTASLDINRA---------WQ------------DG-------
YRW---VYEADIEDFF-DSVDWDKLRLRL--EALYR------------DDP-VIDLIL--AWMAAVV------
---DYQG--
--FSVQR----SM------------

-------------------------------GLPQGAPLS-PTMANLMLD-DLD-------------------
-----NDL-EQ--------------------------------------AG---------FR-----------
------------------------------LVRYADDFVV--LC------RDRAQ----AE-
AAGQEVRRSLA-ELGLQLNDAKSRVVS----F--QQ---------------------------GFRFLGFVF-
>PF_CRI67871.1 [Thiocapsa sp. KS1]
--GGELW--------------------DTEYPEAPDPDEEEELADRLERLGKRLLE------GDY---
RPPALRGVVYRD---P--------------------DG---D-------LRGLAIPPFWDRVAQRALVERIA-
--PALEGV-FSAASHGY---RPGLS-----RHTASSAIQRA---------WR------------EG-------
YRW---VYEADIEGFF-DNLDWQRLAERL--RALYR------------DDP-AVDLLL--AWMAAPV------
---DYQG--
--MRIER----SR------------

-------------------------------GLPQGAPLS-PVLANLMLD-DLD-------------------
-----SDL-EH--------------------------------------AG---------FR-----------
------------------------------LVRYADDFVV--LC------KDQER----AR-
AAGEAVRRSLA-ELGLILNESKSRSVS----F--EQ---------------------------GFRYLGFLF-
>W_[Rhodobacter_capsulatus] 294676824
---GQSI--------------------AA-FAA-----KGAAAIARLSG---LLRN------GNY---
APRPLRLHEIPK---P--------------------DG---G-------TRRLAIPAVSDRIVQTAVAAALT-
--PGSSRC-FPPTATAT---APAVR--
WRW---RWT----------

------------------------------------G------------------------------------
----
-------------------------------SRPCGGWAT-P-------------------------------
-----GWS---RP----------------------
--IS------KRPLT----GS-
RMTRCSRRSIP--
>PF_WP_007469744.1 [Photobacterium marinum]
-ESKKLP--------------------KK-LHQ---SLPH-EDFDQLYE---QLHQ------GNY---
QTGLLTPRLLEK---P---------------------G-Q-K-------RRLLLLPPFIDKVAHKCLSRWLA-
--TSLDTL-YSANSYGY---RKGYS-----RLTAKDRISYL----L----SQ-------------G-------
YKW---VVDADIKAFF-ASIDRQQVAARL--QA--L-YG---------DDT-LWPILD--KMLNAAI---
DPNSELP--L---
---E-L----SI---------

--------------------------------S-GLNLGNSLS-PILANLMLD-HFD----------------
--------DVI-RE--------------------------------------HK----------L--------
-----------------------------E---LVRYADDFLI--LC------REQQQ----AN-
HAKDFVEQLLH-SQALSLNPRKTRVTH----V--NK---------------------------GFRFLGYLF-
>PF_KUI97421.1_(2) [Vibrio sp. MEBiC08052]

```
-DEQQAL--------------------EQ-QRS---DLP-----EMLQQ---VLHH-------DY---
WPAPLTPWLMQE---Q---------------------G-R-K-------ERLILLAEFNDKVLHKTISLWLG-
--ASLDQL-YSKTSYGY---RKGYS-----RLSAKDRIINR----I----RA-------------G-------
YVY---AVDADIRDFF-PSVEQSRVLNRL--SA--L-YG---------ADP-LWTLVE--RFLSAPI---R-
RQHLP--A-----------------------------------------------------------------
-------------------------------------G-------------YED-Y----ER-----------
-------------------------------------------------------------------------
------------------------------T-GLDLGNSLS-PVLANLMLD-HLD------------------
------AVM-ES--------------------------------------LG----------Y----------
---------------------------E---LIRYADDFLV--LT------KSRDK----AQ-
QALHMIEEILT-AQGFALNHEKTRIRH----F--SE---------------------------GIHFLGYLF-
>PF_WP_028302067.1 [Oceanospirillum beijerinckii]
TDEHKAW--------------------EQ-SRE---NLP-----DTLYR---CWHL-------DY---
WPELLHPRLLQQ---A---------------------G-K-K-------ERLLLLPPFPDKVIHKTISRWLS-
--DSLDQL-YSKSSYGY---RKGYS-----RLGAKDRIIHL----V----RK-------------G-------
YKY---ALDADITDFF-PSVNTNRILGRL--AA--L-YG---------QDP-LWQLLE--RFLHCQI---D-
RQNLP--A-----------------------------------------------------------------
-------------------------------------G-------------YEH-H----VN-----------
-------------------------------------------------------------------------
------------------------------Q-GLNLGTSLS-PVLANLMLD-HLD------------------
------SVL-NN--------------------------------------MD----------Y----------
---------------------------E---LVRYADDFLV--LC------KHKQQ----AE-
DARVLIEQLLK-QHDLQLNAEKTKVRS----F--AS---------------------------GIYFLGYLF-
>W_[Fretibacterium_fastidiosum] 479198758
---GQTI--------------------DA-FRR---DRDR--NVTRISD---SLKN------GTY---
APSPLRGVKISK---N-G-------------------G---G-------FRRLGVPTVKDRIVFQGANRLLA-
--DVWDPL-FAPLSFAY---RSGRS-----IADAIDAVIER----I----RK-------------G-------
RVW---FVKGDIKGCF-DELSWDVLSACL--HD--W-LP---------DES-LRRLVN--QAIRVPV---V--
---E--G------------------------------------------------------------------
------------------------------------G---------Q---IR--P----RL------------
-------------------------------------------------------------------------
-----------------------------R-GIPQGSPLS-PLLANLYLH-SFD-------------------
-----LQM-LQ--------------------------------------QG----------F-----------
--------------------------P---VIRYADDWLL--LV------GSEPE----AQ-
AALQTAQGILS-VLNIAINEEKSGIGN----L--RC------E--------------------SVAFLGHRI-
>PF_WP_007481073.1 [Bacteroides salyersiae]
GVDGFTV--------------------AH-FEK---KLTD--NLTELHH---ELVT------GTW---
NPEPYLRVEIAK---N-E---------------T-------E-------KRKLGLLCIKDKIVQQAIKTAIE-
--PQMDKT-FLNISYGY---RPGKG-----AERAIRRTIQE--LKK----LK---------------------
NGY---IAKLDIDNYF-DNINQERLFTRL--GN--W-LK---------DDE-TLRLIK--LCVQTGI---V--
------N------------------------------------------------------------------
------------------------------------P---------Q-LKWE--R----TT------------
-------------------------------------------------------------------------
-----------------------------K-GIPQGAILS-PLLANFYLH-PFD-------------------
-----QFA-IS--------------------------------------------------------------
--------------------------KAPMYIRYADDFLI--AS------PSEKQ----TK-
EAVELIKEELADTFYLQLN--KPLVCN----F--HD---------------------------GVEFLGIIV-
>PF_WP_032556864.1 [Bacteroides fragilis]
GIDGFTL--------------------SH-FEK---RLND--NLIELQH---ELIS------QTW---
NPEPYLRIEITK---N-E---------------T-------E-------KRKLGLLCIKDKIVQQAIKTAFE-
--PQLEKT-FLNLSYGY---RPNKG-----PERAIKRVVHD--LKK----LK---------------------
SGY---VAKLDIDNYF-DTINHERLFTRL--AN--W-LK---------DDE-TLRLIR--LCIQTGI---V--
------T------------------------------------------------------------------
------------------------------------P---------Q-LQWQ--E----IN------------
```

```
-------------------------------------------------------------------------
-----------------------------K-GVPQGAILS-PLLANFYLH-PFD-------------------
-----QFA-AN--------------------------------------------------------------
--------------------------KVPMYIRYADDFLI--AT------STEKQ----IK-
EAVELVKEELESQFYLQLN--TPIIHN----F--HD---------------------------GIEFLGITI-
>DS_gi|113939199|ref|ZP_01425057.1 [Herpetosiphon aurantiacus ATCC 23779]
GPDAVTI--------------------LD-FEA---AWVD--HMQQLAM---ELQS------QIY---
RPLPPRRLFLDK---R-D-------------------G---G-------KRSIAILAVRDRIAQRAVLQILE-
--PEIEPT-FLDCSYGF---RPYVG-----VPHALTRIERY----R----QQ-------------G-------
LQW---VAHADISDCF-GTIDHQILLSQL--HQ--R-IS---------DRA-VVELIG--QWLSVGV---M--
---E--
DAATTEASNWWDDGEDLLERLAKHGEDLLWPNQYPQAGPSYAPQMLDFEANRTDSLRKRALQGLASNAALWGI
THSKRVISGLRSLAPLFKQVPGGSLTWGAAGIATLALIPLS-QRLLR-Q----HE------------------
-------------------------------------------------------------------------
-----------------------R-GTLQGGAIS-PMLANIYLD-SFD------------------------
RAM-TE--------------------------------------RG----------H----------------
---------------------I---LVRFADDFVL--LG------AHQAA----VE-QALADATNVLK-
RLRLATKESKTGVQH----F--ND---------------------------GLTFLGHRF-
>DS_fid|22828908|locus|VBIOriTsu129072_1468| [Orientia tsutsugamushi str.
Ikeda]
GIDGITK--------------------ED-YGK---KLKA--NLLSLLT---RIRK------GQY---
QAKPARIVKIPK--ED---------------------G---G-------KRPLVISCFEDKIIESTVSKILN-
--SVFEPI-FLKYSYGF---HPKLN-----AHDALRELNRL----T----YN---------------------
FNKGA-IVEIDITKCF-NTIKHCELMEFL--RK--R-IS---------DKK-FLRLVM--KLIETPI------
---IEND------------------------------------------------------------------
--------------------------------------------------TIVTN----KE------------
-------------------------------------------------------------------------
-------------------------------GCRQGSIVS-PILANVFLHYVID-------------------
-----SWF-AK-----------------------------ISEENLIGQTG----------------------
------------------------------MVRYCDDMVF--VF------ESEAD----AK-
RFYDVLPKRLN-KYGLNINEAKSQMIK----S-------------------------------GRDHAAN---
>DS_fid|35290299|locus|VBILegLon159544_1142| [Legionella longbeachae
NSW150]
GIDGVTK--------------------EV-YGK---KLED--NLQDLLA---RIRR------HAY---
TPQASRLVEIPK---E-----------------D---G---S-------TRPLAISCFEDKIVQMAVTKLLT-
--AIYEPL-FLPCSYGY---REGKN-----GHEALRALMKY--SNE----FR---------------------
KGA---TLEIDLRKYF-NTIPHGKLLEIL--EK--K-IT---------DRR-FLKLIR--KLIRSPV---V--
---A--N------------------------------------------------------------------
------------------------------------G---------K---AE--L----NE------------
-------------------------------------------------------------------------
-----------------------------L-GCPQGSIIS-PILSNIYLHSVVD-------------------
-----SWF-DE---------------------------------ISKS-HL----------I-----------
--------------------------GKTAMVRFADDMVF--LF------QRSED----AE-
KFYKVLPKRLE-KYGLQLHVDKSSLLK----S--GS------KE-AEEAD----TRGE--RLQTYKFLGFTC-
>DS_fid|42464802|locus|VBIXenNem38452_2364| [Xenorhabdus nematophila ATCC
19061]
GIDRMTK--------------------AA-YGE---HLDG--NIHNLIL---RIRR------GTY---
RPKAARITQIPK--ED---------------------G---S-------KRPLAISCTEDKLVQLAVSDILS-
--RIYEPL-FLPCSYGF---RPGLN-----CHAALKALQQQ---------TY---------------------
RNWNGAVVEIDIRKYF-NTIPHIELMSLL--RK--K-IS---------DRR-FLRLIE--VLITAPV---I--
------E------------------------------------------------------------------
------------------------------------G-------------KQV-S----EN------------
-------------------------------------------------------------------------
-----------------------------VRGCPQGSILS-PVLANIYLHQVID-------------------
```

-----EWF-DE-----------------------------------ISRSH---------IH-----------
--------------------------GRAEMVRYADDRVF--TF------EFMSE----AE-
RFYKVLPKRLN-KYGLELHDDKSQRIP-AGHIAALR------------------ASQSGRRLPTFNFLGFTC-
>DS_Ac.ma.I1/CP000840.1/228971..230873/Acaryochloris marina/Bacterial E
GVDGVTK--------------------AE-YQE---NLET--NLQNLHL---KLRQ------MSY---
RPQPVRQVEIPK---E-----------------D---G---S-------MRPLGISCTEDKVVQEMTRRILE-
--AIYEPV-FIDTSYGF---RPKRS-----CHDALRQLNRE--VMR----KP---------------------
VNW---VADIDLAKFF-DTMPHQEILSVL--SI--R-IK---------DGN-LLRLIA--RMLKAGI---Q--
---T--P--
------------------------------------G---------G---VV--Y----DE------------
----
-----------------------------L-GSPQGSIVS-PVIANIFLDYVLD-------------------
-----QWF-TN---------------------------------VVRH-HC----------R-----------
--------------------------GYCAIIRYADDVAA--VF------EHEED----AI-
RFMRVLPRRLE-KYGLRLNTKKTHLLA----F--GK------RN-ARRCF----QTGQ--RPSTFDFLGLTH-
>DS_Chlorobifid|21392973|locus|VBIChlPha122104_2646| [Chlorobium
phaeobacteroides DSM 266]
GTDGKSW--------------------KT-YEA---QLEE--RLPKLHE---EIHT------GSY---
RAQPVKRVYIPK--TD---------------------G---Q-------KRPLGITAIEDKLVQQAVVTVLN-
--QIYETE-FYGFSYGY---RPGRA-----PENALDALATA----ILK--RP---------------------
INW---ILDADLQKFF-DSIPHDKLMALI--SI--R-VG---------DKR-ILRLIG--KWLKTGY---I--
---E--D--
------------------------------------G-------------KRYRQ----TE------------
----
-------------------------------GTPQGSVIS-PLLANIYLHYVVD-------------------
-----EWV-EQ---------------------------------ERRRRNN----------------------
--------------------------GEVIIIRYADDLVL--GF------QYKTE----AE-
RYLEALSERVQ-TYGLKLHPEKTSLKEFGRYA--EE---------------------------RRRKRGEE--
>DS_fid|23677855|locus|VBISorCel80414_10115| [Sorangium cellulosum So
ce56]
----------------------------M-YGE---ELDA--RLLDLQD---RILR------GSY---
HPQPVRRVHIPK---G-----------------S---G-----------TRPLGIPALEDKIVQQAVRRGLE-
--LIYESM-FLGFSYGF---RPRRS-----THDALDALAVA--IGK----RK---------------------
VNW---IVDADIRAFY-DTIAHAWMQRFI--EH--R-IG---------DRR-LVRLLM--KWLHAGV---M--
---E--D--
------------------------------------G---------V---LH--E----VD------------
----
-----------------------------E-GTPQGGIIS-PLMANIYLHYVLD-------------------
-----LWA-HA---------------------------------WRKR-HA----------R-----------
--------------------------GEVYIVRYADDVVM--GF------EDGRD----AR-
SMRAALSKRLA-SFGLELHPDKTRVLF----F--GR------YA-YEKCE----RRGLR-KPATFDFLGFTH-
>DS_Sr.me.I4_(AJ496462.1_) Sinorhizobium meliloti
GVDGMTV--------------------AK-YEE---RLEQ--NLHDLCD---RVHT------GSL---
PAQPVRRVYIPK---A-----------------D---G---G-------KRPLGVPALEDKIVQGAVAEVLS-
--AVYEAD-FCGFSYGF---RPGRN-----PHMALDALHTA--IMS----QR---------------------
VNW---MLDADIRSFF-DSVDHEWLLQMV--AH--R-IA---------DPR-ILQLIK--LWLRAGI---L--
---E--S--
------------------------------------G---------E---TY--E----TD------------
----
-----------------------------R-GTPQGAGIS-PLLANIFLHYILD-------------------
-----LWV-HQ---------------------------------WRRR-HA----------R-----------
--------------------------GRIVIVRYADDFVM--GF------EKKDD----AQ-
EMLLALKERLG-EFGLALHEGKTRLIE----F--GR------FA-ALSRQ----RRGER-KPETFAFLGFIH-

>DS_Gfid|32294365|locus|VBIPseHal105694_0399| [Pseudoalteromonas haloplanktis TAC125]

--K---------------------VNW--
-VLDLDISKFF-DTVEHDWLIKFI--EH--R-IG---------DKR-IIRLIR--QWIKVGT---V-----D-
-S---
------------------------------HG---------H---RQ--Q----ST-----------------
----
------------------------I-GTPQGAVIS-PLLANIYLHYSFD------------------------
LWL-NK----------------------------------QRK-YA----------R----------------
---------------------GNVTIIRYADDAVL--GF------QKHQD----AI-DCQRALTQRLL-
CFGLKVHPNKTKLIR----F--GR------FAPTQYRE----NPSRG-KPGTFDFLGFTH-
>DS_Bfid|45180964|locus|VBIBurRhi170666_0331| [Burkholderia rhizoxinica HKI 454]
GVDGVTW--------------------QS-YEV---GLGS--NLRDLHR---RVHT------GSY---
RALPVLRRYIPK---A-----------------D---A---G-------LRPLGVAALEDKLVQSVMVEVLN-
--AIYEED-FLGFSYGF---RPGRN-----QHDALDALAAA--IQW----RP---------------------
VNW---ILDADIRSFF-DTVNRQWLIRFV--KH--R-VA---------DPR-VIRLIG--KWLDAGV---L--
---D--N--
------------------------------------G---------R---LM--S----VQ------------
----
-----------------------------A-GTPQGSVIC-PLLANIYLHYVFD-------------------
-----LWI-ER---------------------------------WRRQ-RA----------R-----------
--------------------------GTVVVSRYADDTVV--GC------QHEAD----AL-
RLMKELRQRME-EFDLTLHPEKTRVLE----F--GR------YA-AERRR----RKGMG-KPQTFAYLGFTH-
>DS_UB.I1/AY691909/2430..4342/uncultured_bacterium /Bacterial E1
GVDEMTW--------------------RK-YKE---GSPG--RIADLNE---RVHT------GSY---
RAKPVRRSYINK---S-----------------D---G---R-------KRPLGVTALEDKIVQQAVSTILN-
--QIYETD-FMGFSYGF---REKRS-----QHNALDALYIG--ISR----RK---------------------
INY---ILDADISGFF-DKINHDWLLKFL--EH--R-VA---------DRK-ILRLIK--KWLKVGV---I--
---E--D--
------------------------------------G---------K---RT--S----LE------------
----
-----------------------------V-GTPQGSVIS-PVLANVYLHYAQD-------------------
-----LWA-HQ---------------------------------WRKR-HA----------D-----------
--------------------------GDVIIVRYADDSVV--GF------QYRKD----AD-
RFLKDLIERMG-QFGLELHPVKTRLIE----F--GR------FA-VVNRR----KRGER-KPETFDFLGFTH-
>DS_fid|21698146|locus|VBIDesRet71890_0666| [Desulfohalobium retbaense DSM 5692]
GVDGLTC--------------------AE-YED---GLRE--GLKELHA---RVHR------GSY---
RAQPSKRIHIPK---P-----------------D---G---H-------KRPIGIAALEDKIVQHAVGKVLS-
--AIYEED-FLGFSYGF---RPRRG-----AHDALDALNVG--LTH----RK---------------------
VSW---VLDADIQGFF-DTISHEWMIRFL--EH--R-IA---------DPR-ILRLVR--KWLRVGV---S--
---E--D--
------------------------------------G---------V---WS--Q----TS------------
----
-----------------------------M-GTPQGAVIS-PILGNIYLHYVLD-------------------
-----QWV-HH----------------------------------RRR-HA----------R-----------
--------------------------GDIIIVRYADDYVL--GF------QYRHE----AE-
RFLTDLKARLD-RFGLSLHPEKTRLIE----F--GR------FA-TESRR----KRGQG-KPETFDFLGFTH-
>DS_A.v.I5/CP001157/2471407..2473316/Azotobacter vinelandii/Bacterial E1
GVDGMSW--------------------RE-YEE---DLHQ--RVGKLHA---RLHR------GAY---
RATPSRRVYIPK---A-----------------D---G---R-------QRPLGIASLEDKIVQQAVVTVLN-

--AIYEED-FQGFSYGF---RPGRS-----QHDALDALTVA--LKS----QK---------------------
VNW---ILDADITSFF-DEIDHEWMLMFL--GH--R-IA---------DRR-MLGLIC--KWLQAGV---M--
---E--D--
------------------------------------G---------R---RL--A----AT------------
----
-----------------------------K-GTPQGAVIS-PLLANIYLHYVLD-------------------
-----LWA-RQ---------------------------------WRQR-HA----------R-----------
--------------------------GEMIVVRYADDSVV--GF------RTQWQ----AQ-
RFLVQLQERMA-RFGLSLNASKTRLIE----F--GR------FA-VQNRR----RQGLG-KPETFDFLGFTH-
>DS_B.j.I2/BA000040/2069342..2071253/Bradyrhizobium japonicum/Bacterial
E1
GVDGMTW--------------------QD-YEE---DLEP--RLADLHK---RVQR------GTY---
RPQPSRRTYIPK---A-----------------D---G---K-------QRPLAIAALEDKIVQGATVIVLN-
--AIYEGD-FCGFSYGF---RPGRG-----PHDALDALCTA--IET----RQ---------------------
VNW---IIDADIQNFF-GAVSQPWLVRFL--EH--R-IG---------DKR-IIRLIQ--KWLKAGI---L--
---E--D--
------------------------------------G---------V---VT--A----DD------------
----
-----------------------------R-GTGQGPVIS-PLLGNIYLHYALD-------------------
-----LWA-KR---------------------------------WRQR-EV----------S-----------
--------------------------GGMIIVRYADDVVV--GF------EREDD----AR-
RFLDAMRARLE-EFELTLHPAKTRLIE----F--GR------HA-AAQRK----QRGLG-KPETFAFMGFTF-
>DS_Bfid|19071807|locus|VBIBurCen118154_0098| [Burkholderia cenocepacia
J2315]
GVDGVTW--------------------HD-YEQ---DLDR--NLEDLHG---RLRR------QAY---
RALPSRRRYIPK---A-----------------D---G---K-------QRPLGIAALEDKIVQRALVAVLN-
--AVYEMD-FLGFSYGF---RPQRS-----QHDALDALATG--IAR----TS---------------------
VSW---ILDADISRFF-DTVDHDWLIRFV--EH--R-IG---------DQR-VIRLIR--KWLKAGA---M--
---E--D--
------------------------------------G---------V---IE--P----TD------------
----
-----------------------------E-GTPQGSVIS-PLLANIYLHYVFD-------------------
-----LWA-NQ---------------------------------WRKR-HA----------E-----------
--------------------------GNVVIVRYADDVVV--GF------DKPHD----AK-
RFRRAMQQRLE-QFGLSVHPEKTRLIE----F--GR------FA-ARNRA----SRGLG-KPETFNFLGFTH-
>DS_S.ma.I1/BX664015/172056..173964/Serratia marcescens/Bacterial E1
GVDGIRW--------------------MD-YAG---NMKN--NITDLHR---RLHQ------GSY---
RAQPGRRHYIPK---A-----------------D---G---K-------QRPLGIASLEDKIVQYALVKILN-
--AVYEND-FMGFSYGF---RPGRS-----QHDALDALATG--LVR----TN---------------------
VNW---VLDADISQFF-DRVSHEWLIRFT--EH--R-IG---------DRR-VIRLIR--KWLTAGT---S--
---E--E--
------------------------------------G---------Q---WR--A----TE------------
----
-----------------------------E-GTPQGAVIS-PLLANIYLHYVFD-------------------
-----LWA-HQ---------------------------------WRRR-YA----------T-----------
--------------------------GNVVMVRYADDIVI--GF------DKRYD----AR-
RFRIAMQRRLR-EFGLTVHPEKTRLME----F--GR------FA-AENRA----IRGKG-KPETFNFLGFTH-
>DS_Ch.ph.I2/CP000492/3012641..3014550/Chlorobium
phaeobacteroides/Bacterial E1
GVDGITW--------------------KD-YGE---GLEE--NLADLHR---RIHT------GAY---
RAQPSRRKYIPK---A-----------------N---G---Q-------QRPLGIAALEDKIVQRAVVAILT-
--PIYEAE-FLGFSYGF---RPGRS-----QHDALDALAYG--IKV----KK---------------------
IGW---VLDADISRFF-DTISHEWMIRFL--EH--R-IG---------DKR-IVRLII--KWLKAGV---L--
---E--D--

```
------------------------------------S---------V---RI--E----AE------------
-------------------------------------------------------------------------
-----------------------------E-GTPQGAVIS-PLLANIYLHYAYD-------------------
-----LWA-KQ---------------------------------WREK-HC----------K-----------
--------------------------GDMIVVRFADDSVA--GF------QNKED----GE-
RFLADLKERLA-KFALTLHPEKTRLIE----F--GR------YA-AKNRQ----RRGQG-RPETFDFLGFTH-
>DS_fid|42682369|locus|VBIStiAur43712203747_1515| [Stigmatella aurantiaca
DW4/31 (Prj:54333)]
GVDGVTW--------------------EQ-YAG---NLEA--NVRDLHT---RLHR------GAY---
RARPSRRAYIPK---A-----------------D---G---R-------QRPLGIAALEDKLVQRAVVEVLN-
--AVYETD-FLGFSYGF---RPGRS-----QHQALDALSAG--IYL----KK---------------------
VNW---VLDADIRGFF-DAIDHGWMQKFL--EH--R-IE---------DTR-LLRLVQ--KWLAAGV---M--
---E--D------------------------------------------------------------------
------------------------------------G---------K---WT--Q----SK------------
-------------------------------------------------------------------------
-----------------------------E-GTPQGATVS-PLLANLYLHYVFD-------------------
-----LWS-QR---------------------------------WRKR-VA----------R-----------
--------------------------GEVIIVRYADDFVV--GF------QHRSD----AE-
RFWRELRERLR-SFALELHPEKTRLIE----F--GL------YV-AERRR----ERDQG-RPETFNFLGFTH-
>DS_Fr.sp.I4/CP000820/1651830..1653736/Frankia sp./Bacterial E1
GVDGVTW--------------------TD-YGQ---DLEA--NLQDLHV---RVQS------GCY---
RATPSRRAYIPK---A-----------------D---G---R-------LRPLGIASLEDKIVQRAVVEVLG-
--AVYEVD-FRGFSYGF---RPGRG-----PHDALDALAVG--IWR----KR---------------------
VNW---VLDADIRDFF-GQIDHSWLRRFL--EH--R-IA---------DKR-VLRLID--KWLAAGV---V--
---E--D------------------------------------------------------------------
------------------------------------G---------E---WT--A----CE------------
-------------------------------------------------------------------------
-----------------------------E-GSPQGASVS-PLLANVYLHYVLD-------------------
-----LWV-DW---------------------------------WRRR-HA----------R-----------
--------------------------GDVIVVRWADDFIV--GF------EYEED----AR-
RFLDELRERFA-KFGLELHPDKTRLIE----F--GR------YA-ARDRK----RRGLG-KPETFDFLGFTH-
>DS_Ps.tu.I1/AAOH01000003/353461..355380/Pseudoalteromonas
tunicata/Bacterial E2
GIDGITM--------------------PA-YQQ---QLVG--NITRLSD---ALKH------KRF---
RANDIKRVFIPK---A-----------------N---G---K-------QRPLGLPTVDDKLVQQGVSQILQ-
--SIWEAD-FLPNSYGY---RPNKS-----AHQALHSLALN--LQF----KG---------------------
YGY---IVEADIKGFF-NNLDHNWLMKML--KQ--R-ID---------DKA-MLSLIS--QWLKARI---K--
---SP-E------------------------------------------------------------------
------------------------------------G---------V---FE--Y----PK------------
-------------------------------------------------------------------------
-----------------------------S-GTPQGGIIS-PVLANIYLHYALD-------------------
-----LWF-EK---------------------------------KVKP-RM----------R-----------
--------------------------GRAMLIRYADDFVC--AF------QYAND----AE-
RFYEVLPKRLK-KFNLEVAEEKTSLLR----F--SR------FH-PS-------------RKRQFVFLGFAF-
>DS_clostridiafid|115615051|locus|VBIDehSp228777_0955| [Dehalobacter sp.
CF]
GVDKVTA--------------------KE-FAE---ELKQ--NIENLAE---HLEK------KRY---
RAKLLRRVDIPK--GE---------------------G---K-------TRPLGIPAIADKLVQSAAAKILE-
--AIYEQD-FLASSYGY---RPKVS-----AHTAIKDLSKE--LNY----GD---------------------
YSY---IVEADIKGFF-QNIDHAWLIRML--EQ--R-ID---------DKA-FVGLIK--KWLKAGI------
---LKQD------------------------------------------------------------------
------------------------------------G-------------EVEHP----IT------------
-------------------------------------------------------------------------
-------------------------------GSPQGGIIS-PILANTYLHYVLD-------------------
```

-----LWF-EK--------------------------------IVKPNCEG----------------------
---------------------------EAYLCRYCDDFVC--AF------QYKGD----AD-
KFYRSLPKRLE-KFGLELAVDKTQIIQ----F--NR--------------------WLRKQSSSFEYLGFEF-
>DS_fid|115643628|locus|VBIThiNit264030_1141| [Thioalkalivibrio
nitratireducens DSM 14787]
---ME---RLKT------KRY---
RTKLVRRCYIPK--EN---------------------G---Q-------ERALGIPALEDKLVQLACAKLLT-
--AIYEQD-FLPVSYGY---RPGRD-----AKEAVGDLGFN--LQY----GR---------------------
FGH---VVEADIQGFF-DHLDHDWLLRML--AL--R-ID---------DRA-FLHLIR--KWLKAGI------
---LDTD--
------------------------------------G-------------QVLHP----DA------------
----
-------------------------------GTPQGGIVS-PILANVYLHYALD-------------------
-----LWF-ER--------------------------------VVRPRCRG----------------------
---------------------------QALLIRYADDYVC--AF------QYREE----AE-
GFYRVLPKRLA-KFGLAVAPEKTRILR----F--SR--------------------FHPGLPRRFAFLGFEL-
>DS_Co.ca.I1/FP929038.1/3172164..3174036/Coprococcus catus/Bacterial E
GVDEITK--------------------KE-YER---NLEQ--NIDDLVE---RLKR------KSY---
KPQPSIRVYIPK---S-----------------N---G---K-------LRPLGIACYEDKIVQLALKKILE-
--AIYEPR-FLNCMYGF---RPNRG-----CHNAIKELYKR--LNN----TK---------------------
ICY---IVDADIKGFF-DHMKHEWIIKFL--KL--Y-IK---------DPN-IIGLVK--KYLKVGV---M--
---D--N--
------------------------------------G---------E---LM--V----NE------------
----
-----------------------------E-GSAQGNIIS-PILANIYMHNVLT-------------------
-----LWY-KF---------------------------------IITK-EC----------K-----------
--------------------------GDNFLIAYADDFVA--GF------QCKWE----AE-
NYYKLLKERME-KFGLQLEDSKSRLLQ----S--GA------YI-ARAKQ----KSGECIRLQTFDFLGFTF-
>DS_clostridiafid|58517021|locus|VBICloBot180836_2089| [Clostridium
botulinum H04402 065]
GIDRVTK--------------------VE-YGA---NLEE--NISGLVI---RLKN------KSY---
KPLPVLRVFISK---G-----------------N---G---K-------MRPLGIAAYEDKFVQLAIKKILE-
--AIYEPR-FLENMYGF---RPRRG-----CHNAIKAAYDR--IYE----NK---------------------
INY---IVDADIKGFF-DNMSHEWIMKFL--GV--Y-IS---------DPN-FLWLIN--KYLKAGV---M--
---T--D--
------------------------------------G---------T---LI--D----SI------------
----
-----------------------------S-GSAQGSIIS-PVIANVYMHNVLM-------------------
-----LWY-KF---------------------------------IVLN-GI----------K-----------
--------------------------GKSFLVTYADDFIA--GF------QYKWE----AE-
KYYIELKRRMA-KFNLELEDSKSRLLE----F--GR------FA-EGNRK----ARGEG-KPETFDFLGFTF-
>DS_Fa.pr.I2/FP929046.1/829768..831634/Faecalibacterium
prausnitzii/Bacterial E
GIDKVTK--------------------DE-YGK---NLDR--NIKDLVQ---RLKN------KFF---
KPLPSLRVYIPK---A-----------------N---G---K-------KRPLGIASYEDKIVQMAVKKILG-
--AIYEPR-FLNCMYGF---RPNRG-----CHEAIKEVYQR--ISY----GK---------------------
ISY---IVDADIKGFF-DHIDHEWMMKFL--EW--N-IQ---------DKN-LLWLIR--KYLKAGI---M--
---E--Q--
------------------------------------G---------K---FE--P----TE------------
----
-----------------------------E-GSAQGSVMS-PMLANIYMHHVLT-------------------
-----LWF-KL---------------------------------VVKK-EM----------Q-----------
--------------------------GECFLVNFADDFVA--GF------QYKSE----AE-
RYYKELKERME-KFGLELESSKSRLIE----F--GR------FA-EQNRR----ARGEH-KPETFDFLGFTF-

>DS_W.e.I2_(WP_015588904) Wolbachia endosymbiont of Drosophila melanogaster
GIDRVTV--------------------EA-YGE---NLEE--KLKTLVD---SMKR------KQY---
QPLPVKRVYIPK---A-G---------------S---K---E-------KRGLGIPSTEDKLVQVMLKKILE-
--NIYEAN-FMDSSYGF---RPGRN-----CHQAINALDKA--VMH----KP---------------------
INY---IVEVDIKKFF-DNVQHKWLMNCL--RE--R-IA---------DPN-LLWLIK--RFLKAGI---V--
---E--V--
------------------------------------G---------C---YK--A----TD------------
----
-----------------------------Q-GTPQGGIVS-PVLANIYLHYVLD-------------------
-----LWF-EK---------------------------------KFKP-KA----------R-----------
--------------------------GYLQLIRFCDDFVV--GC------EREED----AK-
EFLELLKQRLS-KFGLEIAENKTKIVK----F--GK------KE-WYQAE-----REKR-RTASFNFLGFTH-
>DS_fid|24026234|locus|VBIWolEnd95846_0368| [Wolbachia endosymbiont of Culex quinquefasciatus Pel]
GIDWVTV--------------------EA-YGE---NLKE--RLEGLVD---SMKG------KQY---
QPQPVRRVYIPK---A-G---------------S---K---E-------KRGLGIPSTEDKLVQIMLKKILE-
--NIYEAN-FLDSSYGF---RPGRN-----CHQTVNALDKA--VMY----KP---------------------
INY---IVEVDIKKFY-DNIQHKWLMRCL--RE--R-IT---------DPN-LLWLIK--RFLKAGI---V--
---E--A--
------------------------------------G-------------YYE-A----TK------------
----
-----------------------------Q-GTPQGGIVS-PVLANIYLHYVLD-------------------
-----LWL-EK--------------------------------KFKPRSRG---------YI-----------
--------------------------Q---LIRFCDDFVV--CC------ESKVD----AE-
EFLELLKQRLN-KFGLEVSENKTRVVK----F--GK------R-------------------------EWQQ-
>DS_fid|115349385|locus|VBIThiMob160332_0442| [Thioflavicoccus mobilis 8321]
GIDGISK--------------------EQ-YGA---NLDE--NIKELSS---RLRN------MGY---
RPQPKRRTYIPK---P-G---------------S---V---K-------GRPLAISCFEDKLVELAIKRVLE-
--PIYEVQ-FEDSSYGY---RPGRS-----QHQCLDDLGRT--IQQ----SR---------------------
INT---IVEADIRSFF-NTVDHAWMLKFL--GH--R-IG---------DPR-IIRLIG--CLLKGGI---L--
---E--D--
------------------------------------G---------L---VQ--A----SE------------
----
-----------------------------E-GTPQGSILS-PLLSNIYLHYVLD-------------------
-----LWF-SR---------------------------------RVRP-QC----------R-----------
--------------------------GEAYYFRFADDFVA--GF------QYRQE----AE-
QFQTALGERLG-QFKLRLAEEKTRCLA----F--GR------FA-RSNAQ-----KQGQ-KPGEFTFLGFTH-
>DS_UA.I7/FP565147.1/1619711..1621718/uncultured_archaeon /Bacterial E2
GIDGVTV--------------------GE-YAK---ALDE--NIADLVA---RLKA------KQY---
KPQPVLRVYIPK---P-----------------N---G---E-------KRPLGIPAVEDKIVQMALKKILE-
--AIFEQD-FIDTSYGF---RPNRS-----CHDALTELDRI--IMN----VP---------------------
VNF---VVDMDISKFF-DTVDHKRLMECL--RQ--R-IV---------DPT-LLQLIG--RFLKSGI---M--
---E--E--
------------------------------------G---------K---YS--E----MD------------
----
-----------------------------Q-GTPQGGVLS-PVLANVYLHYVLD-------------------
-----KWF-EN---------------------------------EVLP-QL----------T-----------
--------------------------GFAQLIRYADDFVV--CF------EKETE----AR-
AFGVALRRRMG-KFGLTISEEKSKIIE----F--GR------CT-CTRAK-----RYGR-KCETFDFLGFTH-
>DS_UA.I6/FP565147.1/2174432..2176370/uncultured_archaeon /Bacterial E2
GVDGVTW--------------------RK-YEE---NLDE--NTEDLVT---RLIA------KQY---
RPQPVKRAYIPK---S-----------------N---G---E-------RRPLGIPALEDKIVQLAIKKILE-

--AIFEED-FCDVSYGF---RPNRS-----CHDALDMVDMI--IMT----KP---------------------
VSY---VVDMDIAKFF-DTVDHECLMECL--KQ--R-VV---------DPS-LLRIIA--RCLKSGV---M--
---E--E--
------------------------------------G---------K---YL--E----TD------------
----
-----------------------------K-GTPQGGILS-PILANIYLHYALD-------------------
-----LWF-EK---------------------------------EVKE-QL----------K-----------
--------------------------GFAQLIRYADDFIV--CF------QHDDE----AR-
AFGKTLRERLA-KFGLTISEEKSRIIK----F--GR------YA-CQQAR-----KQSK-KCATFDFLGFTL-
>DS_Cl.be.I3/CP000721/3718265..3720149/Clostridium beijerinckii/Bacterial
E2
GIDDVTK--------------------QE-YSK---ELDN--NIENLIV---KLRN------HSY---
KPQAVKRVYIPK--GD---------------------G---K-------TRPLGIPSYEDKLVQMALNKILQ-
--SIYEAE-FKDFSYGF---RPKRN-----CHSAIKALNKV----I----EN-------------GR------
INY---VVDADIKGFF-NNVNHEWMIKFL--EV--R-IG---------DPN-IISLVK--KFLKAGL---M--
---D--N--
------------------------------------G-------------IIKTT------------------
----
-----------------------------EIGTPQGSIVS-PTLANIYLHYSLD-------------------
-----LWF-EK--------------------------------VIKRNFRG----------------------
---------------------------QSEITRYADDFVC--CF------QYESE----AR-
QFCRLLVSRLN-KFNLEVERTKSKLIL----F--GR------------FAEEIRKSRGFKNAETFDFLGFTH-
>DS_Ta.sp.I2/CP000923.1/1286631..1288551/Thermoanaerobacter sp./Bacterial
E
GVDKVTW--------------------EE-YDV---NVDE--NVETLIA---KMKR------FSY---
RPQPARRVYIPK--AN---------------------G---K-------LRPLGIPCYEDKLVAAVMADILN-
--EVYENI-FLDTSYGF---RPGRS-----CHDAIKELNRI----I----GR-------------------
CKISY---VLEADIKGFF-DNVDQKQLMEFI--AH--D-ID---------DKN-FSRYIV--RFLKSGI---
M-----E--E---
---------------------------------------G-------------KYH-E----SD---------
----
--------------------------------K-GTAQGSPLS-PILANIYLHYTLD----------------
--------VWF------------------------------------AYLKRNG--------KFR--------
-----------------------------GEAYIVRYADDFVM--LF------QYKSD----AD-
KMYEALPKRMA-KFGLELAMDKTKILP----F--GR---------------FAKQNSKDGKTETFDFLGFTF-
>DS_clostridiafid|115615774|locus|VBIDehSp228777_1721| [Dehalobacter sp.
CF]
GIDGETK--------------------AS-YGG---NLEE--NLRNLLE---QLKE------GSY---
RPTPVRRKFIPK---A-G---------------S---N---K-------LRPLGIPVLEDKLVQNALVIILE-
--SIYEQD-FLEDSYGF---RPGRS-----QHDALKDLSRK--IGT----RK---------------------
VGY---IVDADIRGYF-DHVDHEWLLKML--QE--R-IS---------DSK-ILKLIK--RFLKAGV---M--
---E--E--
------------------------------------G---------K---LS--K----TE------------
----
-----------------------------E-GVPQGGSLS-PLLGNIYLHYVLD-------------------
-----LWF-NK---------------------------------IITK-QC----------Q-----------
--------------------------GEAYLTRFADDTVA--CF------QYQKD----AE-
RFYEALKKRLK-KFNLEIAEEKTRIIE----F--GR------YA-QRDVQ----RRGGR-KPETFDFLGITH-
>DS_Cl.be.I2_(YP_001310744.1_) Clostridium beijerinckii NCIMB 8052
GVDKVTK--------------------EE-YET---NLEN--NIDNLLI---RMKT------FKY---
RPQPVRRVYIDK---S-G---------------S---N---K-------KRPLGIPAYEDKVVQLAINKILK-
--SIYEQD-FIDSSFGF---RQNRS-----CHDALKILNVY--LSE----KN---------------------
VNY---VVDADIKGFF-DNVDHKWLMKFL--EH--R-IA---------DKN-LLRYIG--RFLKTGI---M--
---E--N--

```
------------------------------------G---------K---FY--K----VY------------
-------------------------------------------------------------------------
-----------------------------E-GTPQGGIIS-PTLANIYLHYVLD-------------------
-----IWF-NN---------------------------------FIKK-KC----------K-----------
--------------------------GQAYIVRYADDFVC--CF------QYEDE----AK-
AFYEALKNRLD-KFNLQVAEDKTKILY----F--GK------NA-YYDRKFKRAKLESY-KDRTFDFLGFTH-
>DS_Mo.th.I1/CP000232.1/2324936..2328581/Moorella thermoacetica/Bacterial
E
GIDGITK--------------------EQ-YGD---NLEA--NIQSLLE---RLKR------KAY---
RPQPVRRVYIPK---P---------------------G-SDK-------KRPLGIPAYEDKIVQLAASKILN-
--AIYEAE-FLDMSFGF---RPQRG-----CHDALKLLNYL----IVA--RK---------------------
VNY---IVDADIKGFF-DHVNHDWLMKFL--GH--R-IA---------DPN-FLRFIR--RFLKAGI---M--
---E--N------------------------------------------------------------------
------------------------------------G-------------ELRDA----TE------------
-------------------------------------------------------------------------
-------------------------------GTPQGGIVS-PILANIYLHYVLD-------------------
-----LWF-EK--------------------------------AVRKHCRG----------------------
---------------------------EAYMVRYADDFIC--CF------QYKHE----AE-
AFYRALKARLA-KFSLSVAEEKTKIIP----F--GR------------FATQWCKRMGQNKPDTFDFLGFTH-
>DS_clostridiafid|21720033|locus|VBIDesAce42372_1641| [Desulfotomaculum
acetoxidans DSM 771]
-------------------------------------------------------------------------
-------------------------------------------------------------------------
-------------------------------------------------------------------------
-------------------------------------------------------------------------
-------------------------------------------------------------------------
-------------------------------------------------------------------------
-------------------------------------------------------------------------
---------------------------------MS-PILANIYLHYTLD------------------------
LWF-ER---------------------------------VVRK-YC----------R----------------
---------------------REAYIVRYCDDFVC--CF------QYKID----AE-RFYLALIQRLK-
KFNLEIAEERTKIIE----F--GR------FA-CTQCK----KYGKT-KPETFDFLGFTH-
>DS_Sy.wo.I1/NZ_AAJG01000003/20007..22007/Syntrophomonas wolfei/Bacterial
E2
GVDQVTK--------------------QA-YEE---NLEA--NIADLIG---RMKR------QAY---
KPQPVRRVYIPK---E-G---------------S---N---K-------RRPLGIPSYEDKLVQKGLARILN-
--TIYEQD-FLDCSFGF---RPGRG-----CHDALKVLNHI--IER----KK---------------------
VNY---IVDADIRGFF-DHVDHEWMMKFL--EL--R-IA---------DPN-LLRLIK--RFLKAGV---M--
---E--A------------------------------------------------------------------
------------------------------------G---------I---VY--D----TP------------
-------------------------------------------------------------------------
-----------------------------K-GTPQGGIVS-PILANIYLHYVLD-------------------
-----LWF-EK---------------------------------VVKK-RC----------Q-----------
--------------------------GEAYLVRYADDFVC--CF------QNKSD----AE-
WFYANLRERLN-KFNLEVAEEKTRIIA----F--GR------FA-DKESK----KQGRK-KPDTFDFLGFTH-
>DS_Bacteroidetesfid|61290805|locus|VBINiaKor154066_6177| [Niastella
koreensis GR2010]
GVDEETW--------------------ID-YHK---QRET--RIPQLLA---AFKS------GNY---
RAPNIRRVYIPK---D-----------------K---G---K-------LRPLGLPTVEDKVLQTAVTRVLR-
--PVYEDI-FYHSSYGF---RPGKS-----QHQALEELTRQ--VSL----EG---------------------
KRY---IIDADMQNYF-GSINHQCLRDLL--DL--R-IK---------DGV-IRKMID--KWLKAGI---L--
---D--N------------------------------------------------------------------
------------------------------------G---------Q---LV--Y----PT------------
-------------------------------------------------------------------------
```

-----------------------------E-GTPQGGSIS-PLISNVYLHYVLD------------------
-----EWF-YQ---------------------------------QIRP-LL---------K-----------
--------------------------GDSFLIRFADDFLL--GF------TNKED----AL-
RVMHVLPKRLG-KYGLMLHPEKTKLID----L--TT------KK-GGP--------DQ-EKNTFDFLGFCH-
>DS_fid|42685679|locus|VBIStiAur43712203747_3158| [Stigmatella aurantiaca DW4/31 (Prj:54333)]
GIDRQTA-------------------KD-YEA---NLEV--NLKSLLE---RIKS------GRY---
KAPPVRRTYIPK---A----------------D---G---S-------QRPLGIPTFEDKVAQRAIVLLLE-
--PIYEQD-FRPFSFGF---RPGRS-----AHQALRELRSS--ILE----RN--------------------
GRW---VLDVDLRRYF-DTIEHGKLREVL--AR--R-VA--------DGV-VRRMID--KWLKAGV---L--
---E--E---
------------------------------------G---------P---LL--R----LE-----------
----
-----------------------------Q-GTPQGGVIS-PLLANVYLHYVLD------------------
-----EWY-ER---------------------------------EVVP-RM---------K-----------
--------------------------GKCSLIRYADDLVM--VF------EDFLD----CR-
RVLEVLGKRLA-KYGLTLHPGKTRMVD----F--RF------KR-PGGGQ----HPATQ--ATTFDFLGFTH-
>DS_fid|190138491|locus|VBIRhiEtl298076_5694| [Rhizobium etli bv. mimosae str. Mim1]
GIDGRTA-------------------DD-YEK---DLEA--NLESLRI---RMMS------GSY---
RAPPVRRHYIPK--AD--------------------G---S-------RRPLGIPTIEDKVAQRAIVMLLE-
--PIYEED-FLDCSFGF---RPERS-----AHDAIRTLRDG----I----MD-----------TG-------
QRW---VIDADISKYF-DSIDHGHLRSFL--DL--R-IR--------DGV-IRRMID--KWLNAGV---L--
---D--Q---
------------------------------------G------------TSS-R----SV------------
----
-----------------------------A-GTPQGGVIS-PLLANILLLHVLD------------------
-----RWFVEV-----------------------------------VKPR---------LK-----------
--------------------------RRCQMVRYADDFVM--SF------EDHLD----GR-
RMLAVLGKRFE-RYGLRLHPDKTRYVD------------------------------------FRFRRPHG-
>DS_fid|23659127|locus|VBISorCel80414_0791| [Sorangium cellulosum So ce56]
GVDGITK-------------------EQ-YGQ---DLEH--NVRDLHA---RMKS------MRY---
RHQPIRRVHIPK--ER--------------------G---K-------TRPIGISCTEDKIVQAAVREMLE-
--VIYEPV-FRDVSYGF---RPGRS-----AHDALRALNRM----L----LG------------G-------
VEW---ILEADIESFF-DSIDRTKLMEML--QA--R-VA--------DKS-LLRLVG--KCLHVGV---L--
------D---
------------------------------------G------------AEFYA----PE------------
----
-----------------------------D-GTVQGSVLS-PLLGNVYLHHVLD------------------
-----LWI-ER-------------------------------EVQPRLVG--------KAT-----------
------------------------------LIRYADDFII--GF------EREDD----AK-
RVTEVLPRRFE-RYGLKLHPDKTRLLP----FGRPD----------------NGQPGGKGPATFDFLGFTH-
>DS_fid|22095829|locus|VBIHalHal112047_0768| [Halorhodospira halophila SL1]

----------M---

---------------------------------VS-PLLANVFLHEVLD-----------------------
EWFETQ--------------------------------AKPRLRG---------PA----------------

```
---------------------Q---LVRYADDAVL--LF------KLRDD----AE-RVLKVLPRRFE-
KYGLELHPEKTRLIG----F--QR------PPRNVKRPWPKPE--------TFDLLGFTL-
>DS_N.a.I1/AF079317/43084..45661/Novosphingobium aromaticivorans/ML
GVDGQTF--------------------DG-FSP--------DKVRSIIE---RLAN------GTY---
RPQPARRVYIPK--AN---------------------G---Q-------KRPLGVPTTEDKLVQEVVRTILE-
--QIYEPL-FSRHSHGF---RPKRS-----CHTALESIRAI---------WT-------------G-------
VKW---LIDVDVVGFF-DNIDHDVLVSLL--EK--R-IA---------DRR-FVRLIR--GLLKAGY---V--
---E--D------------------------------------------------------------------
------------------------------------W-------------VFH-K----TY------------
-------------------------------------------------------------------------
-----------------------------S-GTPQGGVVS-PMLANIYLH-ELD-------------------
-----MFM-QAKMAGFDKGKQRSPSPDARRIRNRLSYVRRTVDQLRAKGRG---------DD-------
PRVTSFLEEIGRLKAERLAVPASDAFDPNYRRLRYCRYADDFII-GVT------GSKSE----AR-
QIMEEVRTYLSDHLKLAVSAEKSGIHK----A--SD---------------------------GARFLGYEV-
>DS_N.a.I2/AF079317/53812..56360/Novosphingobium aromaticivorans/ML
GIDGKTF--------------------ED-FGP--------DRLAPLIA---SVAT------GAY---
KPKPVRRVFIPK--GK---------------------G---K-------RRPLGIPTRDDRLVQEVARQLLE-
--RIYEPV-FSKASHGF---RPGRS-----CHTALEHVKAV---------WT-------------G-------
VKW---LVDVDVAGFF-ENIDHDILLKLL--RK--R-ID---------DER-FIDLIR--DMLKAGV---M--
------E------------------------------------------------------------------
------------------------------------G-------------RAHTQ----TY------------
-------------------------------------------------------------------------
-----------------------------S-GTPQGGIVS-PILANIYLH-ELD-------------------
-----EFMAGR--------ITAFEKGKTRATNPEYRRLAGRIAKRRERLKR---------
LEASDNADQVTVKAILAEINTLSKQMRSLPSRDAMDAGFRRLRYCRYADDFLI-GVI------GSKDD----
AR-GVFAEVRTFLTEVLALTVSEEKSGIRK----A--SD---------------------------
GTKFLGYEV-
>DS_fid|87114490|locus|VBIEscBla78014_3566| [Shimwellia blattae DSM 4481
= NBRC 105725]
GINNNTM--------------------DEMSVG---------RIINLIQ---LINS------GSY---
KPRPCRRTHIPKDARK-------------------PNG---K-------KRPLGIPTGDDKLIQEVMRMLLE-
--EIYEPV-FSDWNYGF---RPKRS-----CHSALKEIRNS---------WK-------------G-------
TKW---VCDVDIKGYF-DNIDHDLLLKFL--SK--R-IA---------DNK-FLALLK--KFLKAGY---L--
------D------------------------------------------------------------------
------------------------------------N-------------WRYFG----TH------------
-------------------------------------------------------------------------
-----------------------------S-GTPQGGIIS-PILANVFLH-KLD-------------------
-----EFMKNR-----ISEFGKGGRRKPNPIYKRALQNRANRIKWIRQGFG----------
ASGMPADEQKIQKWRHEADELEKKLRTLSSVIMDDSEFKRMRYVRYADDFLI-GVT------GSKNE----
AK-KIMKEVVDFVETELHLEISKEKSGIID----P--KK---------------------------
GFTFLGYEI-
>DS_fid|58846094|locus|VBIPelHal211702_2804| [Pelagibacterium
halotolerans B2]
GVSGNTL--------------------DG-FGE--------ERVAALMH---AIST------GTY---
KPSPVRRTYILKDPKN-------------------PAG---K-------KRPLGIPTGDDKLVQEVVRALLE-
--VIYEPV-FSDRSHGF---RPGRS-----CHTALNQIVRS---------WK-------------G-------
TKW---ICEVDIKGYF-DNIDHETLLGLL--AR--K-ID---------DRA-FLKLIR--EFLVAGY---L--
---E--D------------------------------------------------------------------
--------------------------------------------------WTYNA----TY------------
-------------------------------------------------------------------------
-----------------------------S-GTPQGGVVS-PILANIYLH-ELD-------------------
-----QFMDSRMAAFNRGARRKPNPEYCRLNNLASIRRRKLRVHGDSHSKAARWRQEMQEME-----------
----------AAKALLPSVDMHDEGFKQLHYVRYADDFLI-GIV------GSKEE----AA-
QIMAEVRSFVEGPLKLTISAEKSRMGA----M--SK---------------------------GTVFLGYGV-
```

>DS_L.l.I1/U50902/2854..5345/Lactococcus lactis/ML
GILLDDTA-------------------DG-FSE--------EKIKKIIQ---SLKD------GTY---
YPQPVRRMYIAK---K--------------------NS-K-K-------MRPLGIPTFTDKLIQEAVRIILE-
--SIYEPV-FEDVSHGF---RPQRS-----CHTALKTIKRE----F----GG---------------------
ARW---FVEGDIKGCF-DNIDHVTLIGLI--NL--K-IK---------DMK-MSQLIY--KFLKAGY---L--
---E--N--
------------------------------------W---------Q---YH--K----TY------------
----
-----------------------------S-GTPQGGILS-PLLANIYLH-ELD-------------------
-----KFV-LQLKMKFDRE--SPER-ITPEYRE----LHNEIKRISHR-LK----------KL----
EGEEKAKVLLEYQEKRKRLPTLPCTSQTN---KVLKYVRYADDFII-SVK------GSKED----CQ-
WIKEQLKLFIHNKLKMELSEEKTLITH----S---S------Q--------------------PARFLGYDI-
>DS_Bacillifid|18918679|locus|VBIBacCer120424_5472| [Bacillus cereus Q1]
GTINNTV--------------------DG-FSK--------NRVSKIIN---NIKN------GNY---
KPTPVKRVYIDK---K---------------------G---S----K-KKRPLGIPTFDDKLVQLVIKYILE-
--AIYEPN-FSENSHGF---RKNRG-----CHTALKQIKKS---------GS-------------G-------
TKW---FIEGDIQGFF-DNIDHHILINLL--RK--R-IN---------DET-LIGLIW--KFLRAGY---M--
---E--D--
--WQF-H----KT------------

-----------------------------FSGTPQGGILS-PLLANIYLN-ELD-------------------
-----IYM-EKYAERFGKGQPKDREVDKRYQYLHLKIKRGRKKADLLREQG---------KL-------
NESQELIHQVNEWIKERGQRPYYNPMSDKFKSLKYVRYADDFIV-MLI------GSKDD----AN-
AIKSDIAQFLNEELKLTLSEEKTLITH----S--SK---------------------------KAKFLGYNV-
>W_[Herpetosiphon_aurantiacus_DSM_785] 159898445
GSTDETI--------------------DGMSMA---------KIHRIIA---DLRR------ETY---
RWTPVRRVYIPK--AT---------------------G---K-------TRPLGVPTWSDKLVQEVLRSILD-
--AYYDPQ-MSDHSHGF---RPNRG-----CHTALKAIQRC---------WT-------------G-------
TRW---FIEGDIAQYF-DTINHTTLLTIL--AK--R-IH---------DGR-FLRLIQ--TLLQAGY---L--
---H--D--
--WVYHP----TL------------

-----------------------------S-GTPQGGVIS-PLLANIYLH-EFD-------------------
-----QFVEHT------------------------LIPAYTKGQRRKVNPA--------
YAQMEQRISKLRRQREYASVTPLLKELRTLPSRDVHDPDYRRLRYVRYADDFLL-GFA------GTKVE----
AE-AIKQQINVWLYDHLQLKLSTQKTLITH----A-SSD---------------------------
PAHFLGYDI-
>DS_Bacillifid|190354377|locus|VBIStrAng166616_0608| [Streptococcus anginosus C238]
GVTEETI--------------------DGMSIQ---------KIDMIIE---QLRQ------ETY---
YWRPARREYIPK--KN---------------------G---K-------HRPLGIPVWSDKLLQEVIRMILE-
--AYYEPQ-FSEHSHGF---RPKRG-----CHTALQEIQ-T---------WQ-------------G-------
THW---FIEGDISSYF-DTIDHCVLITML--SK--Q-IQ---------DGR-FIRLIK--NMLEAGY---L--
---D--D--
--WKFRK----TI------------

-----------------------------S-GTPQGGVIS-PLLANIYLH-QFD-------------------
-----KWVGEE------------------------LIPQYTRGKKQKANSA--------
YNRLSRKIKFYQDKGEYKKAHQIIVERRNIPSVDTYDTNYRRLRYVRYADDFIL-GFT------GSKAE----
AK-DIKKQIGDFLNIKLHLELSQEKTLITH----A-TEE---------------------------
SAKFLGYEI-
>DS_clostridiafid|47030643|locus|VBISynGly105927_0075| [Syntrophobotulus glycolicus DSM 8271]

GVDNRTI--------------------DG-FKY--------EMIDTLIE---KLKT------EQY---
YPKPVRRTYIPK--KN---------------------G---K-------TRPLGIPCFEDKLLQEVIRQLLE-
--SIYEPI-FSDNSHGF---RPDRS-----CHTALCQIKNT----M----RG---------------------
ANW---VIEGDITGCF-DNIDHTILLNIL--SQ--K-IE---------DGR-FIELIR--RFLKAGY---L--
------E--
--FKQMH----RS------------

-----------------------------LSGCPQGGIIS-PILSNIYLN-EFD-------------------
-----RYMDEI---------------------------------
INKNTKGKKRRSNPEYQRLRGKRYTAIKKGNLEEIKRLTKEIQSIPSLDPMDSNFTRVKYVRYADDFVI-
EVI------GSKEM----AE-SIKEDVATFLKEKLNLELNQEKTLITN----L-GNE----------------
-----------KANFLGYEF-
>DS_Bacillifid|18911848|locus|VBIBacCer120424_2093| [Bacillus cereus Q1]
GTDKETI--------------------DG-FSM--------DWIENIIS---SLKD------ESY---
KPNPSRRVYIPK---K--------------------DD---K-------QRPLGIPSIKDKIIQEVVKEILV-
--SMYEPI-FSKASHGF---RPNKS-----CHSALNDIKMT----F----GG---------------------
IKW---WIEGDIKGFF-DNIDHHVLIGIL--RK--R-IK---------DEK-FIKLIW--KFLKAGY---M--
---E--D--
------------------------------------W---------K---FN--K----TF------------
----
-----------------------------S-GTPQGGIIS-PVLANIYLH-ELD-------------------
-----AFM-EKQIIKFDEG--KRRR-DNPVYKK----YNTAIWYRKNK-LK----------
EKWNTLNDDERKELQSEISTLEKEREKHSAVDNMDASFKRLKYVRYADDFVV-GVI------GSKED----
SK-RIKEEITEFLHTSLKLELSQEKTLITS----N---K------N--------------------
LIKFLGYEI-
>DS_Bacillifid|54164737|locus|VBIBacThu155232_5952| [Bacillus thuringiensis serovar chinensis CT43]
GVDQRSI--------------------DG-FSM--------KEVEDLIS---VLKS------KSY---
QPYPSRRTYIEK--KN---------------------G---K-------KRPLGIPSFYDKLVQEVIRMILE-
--AIYDSS-FSSSSHGY---RKGKG-----CHSALLEIKRT----F----TG---------------------
SKW---FIEGDIKGFF-DNIEHHTLVTIL--KR--R-IK---------DEA-FIELIW--KFLRAGY---L--
---E--E--
--WKFHN----TY------------

-----------------------------S-GAPQGGIIS-PIISYIYLN-ELDTYMKKYQDRFESGKK----
-----RQI-NKEYSNLQYKVRKIQEKIDTAYLNGEVTRITELKEQQKVLKG--------KLL-----------
--------------QTPYNNPMDENYRRLKYVRYADDFLI-GVI------GSKED----AI-
LIKNEIASFLKEEIKLELSMEKTLITN---AF--KK---------------------------HAKFLGFEI-
>DS_Bacillifid|19729760|locus|VBIStrPyo25933_1754| [Streptococcus pyogenes MGAS10750]
GVDNQTI--------------------SA-MSL--------ERINKIID---SLKD------ESY---
SPTPTKRVYIPK---K--------------------NG---K-------LRPLGIPSIGDKLVQEVCRMLLN-
--SIYDES-FEDTSHGF---RDNRS-----CHTALRQIQNR----F----VR---------------------
CKW---FVEGDIKGFF-DNIDHNIMIDIL--SK--R-ID---------DER-FLRLIR--KFLKSGY---M--
---E--Q--
------------------------------------N---------Q---YH--N----TY------------
----
-----------------------------S-GMPQGSIIS-PILSNIYLD-KFD-------------------
-----KYM-QNYKESFDKG--NKRK-QNKEYKA----LYDRRKRLENK-LS----------KT---
TNKTEIDDIKSEIEEINKRYFNIPCLNPMDENFKRIQYVRYADDFII-GII------GSKAD----AE-
MVKQDIGQFIKSELNLELSDEKTLVTK----S---T------D--------------------RAKFLGFDI-
>DS_clostridiafid|42835086|locus|VBICloCf158569_1256| [Clostridium cf. saccharolyticum K10]

GTDGKTI--------------------DG-MGM--------ARINALIE---KMRN------SSY---
QPNPARRTYIPK--SN---------------------G---K-------MRPLGIPSFDDKLIQEVVRLILE-
--SIYEPT-FSDHSHGF---RMNKS-----CHTALKYVQKY----F----TG---------------------
TKW---FVEGDIKGCF-DNVDHHVLIAIL--RK--R-IA---------DEQ-FIGLLW--KFLKAGY---M--
---E--D--
--WNYHN----TY------------

-----------------------------S-GTPQGSIIS-PILANIYLN-ELD-------------------
-----HFM-AEYAEKFNCGDRRRINPAFKKKLDVCRGKEERLKRNISKMSE--------EEK----------
EGLLAEISELRRSLRSMPYSDQMDEGYKRVFYIRYADDFLI-GVI------GRKAD----AE-
QVKQDVGHFIRENLHLEMSEEKTLITH----G--HD---------------------------FAKFLGYEV-
>DS_Bacteroidetesfid|46993147|locus|VBIOdoSpl147623_0215| [Odoribacter
splanchnicus DSM 220712]
GTDGKTE--------------------DEMSID---------RINKLIE---SIKD------ETY---
SPNPAKRIYIPK--KN---------------------G---K-------MRPLGIPSFEDKLVQEAVRMVLE-
--AIYEGH-FEWTSHGF---RPNRS-----CHTALKSLQNN----F----NG---------------------
AKW---FIEGDIKGFF-DNIDHDVLIEIM--KG--R-IA---------DDR-FLRLIR--KFLNAGY---M--
------E--
------------------------------------E-------------WQFNK----TY------------
----
-----------------------------S-GTPQGGIIS-PVLANIYLD-KFD-------------------
-----KYMNEYANKFNKGTVRSRNKDICKLNSRVHYLKRRINEVEDVNVRTRMVEELHEKQK-----------
-----------RILTMPSGNDMDRNFRRLRYLRYADDFLI-GVI------GTKNE----CE-
TIKADITKFMQEKLRLEMSQEKTLITN----A--QD---------------------------SAKFLGYEI-
>DS_Ba.fr.I1/AY515263/38446..40893/Bacteroides fragilis/ML
GTDGQTI--------------------SGMSIK---------RIQSIID---KLRD------ESY---
QPHPAKRIYIPK--KN---------------------G---K-------QRPLGIPSFEDKLVQKVIQMILE-
--SIYEGS-FEKCSHGF---RPHRN-----CHTAMASIMEG----F----DG---------------------
TRW---FIEGDIKGFF-DNIDHDIMITIL--SE--R-IA---------DER-FLRLIR--KFLNAGY---L--
------E--
------------------------------------K-------------WKFHK----TF------------
----
-----------------------------S-GTPQGGIIS-PILANIYLD-QLD-------------------
-----KYVVEYISQFNRGKMRKRNPEYKRIASRKDKRVKKLKTETDEQKRAALRSEIVELHR-----------
-----------EMQKHPATLDMDEDFRRMRYVRYADDFLI-GII------GSKDD----CV-
NIKADIKRFLCEKLKLELSDEKTLITH----G--HD---------------------------HAKFLGFEV-
>DS_Bacillifid|19673908|locus|VBIStrPne132160_1355| [Streptococcus
pneumoniae ATCC 700669]
GVDELTI--------------------DGMSIA---------RIDQLID---SLKD------ESY---
QPHPSRRTYIPK--KN---------------------G---K-------LRPLGIPSFDDKLLQQVIKMILE-
--AIYEGQ-FEPSSHGF---RPNKS-----CHTALTQIQKT----Y----TG---------------------
TKW---FIEGDIKSFF-DNINHDVMIHIL--RE--R-IT---------DER-FLRLIR--KFLNAGY---V--
---E--D--
--WKFYK----TY------------

-----------------------------S-GTPQGGIIS-PILANIYLD-KFD-------------------
-----KYMTDYVKNFCQGKYRKRTPEYRQNEIALGKARRALECVSTENQRQEVIQRIRQLEK-----------
-----------ERVLIPHSDPMDSSFKRLTYTRYADDFIC-GVI------GSKED----AH-
RIKADIKDYLEAVLKLELSVEKTLITN----A--RD---------------------------KAKFLGYHL-
>DS_Bacteroidetesfid|42711385|locus|VBIAliSha154597_1257| [Alistipes
shahii WAL 8301]

---MVLE---
AIYEGH-FEDTSHGF---RPHRS-----CHTALNAVQKT----F----TG---------------------

KKW---FIEGDIKGFF-DNVNHDILIDIL--KE--R-IS---------DER-FIRLIR--KFLKAGY---L--
------E--
------------------------------------Q-------------WQFHG----TY------------
----
-----------------------------S-GMPQGGIIS-PILANIYLD-KLD-------------------
-----KYMKEYASKFDKGDRGRQQREYEVLTYQKRLVMRELKTATNNVERK--------VLV-----------
---NRLKEIDKTRSAMPCFAPMDGNFKRLKYVRYADDFLI-GII------GSKED----AV-
KIKDDIKRFLADRLALELSDEKTLITH----T--EK---------------------------PAKFLGYEV-
>DS_B.t.I2/AE015928/3241156..3243662/Bacteroides thetaiotaomicron/ML
GADGKTI--------------------DG-MSI--------DRVEQLIG---SLKN------ETY---
QPNPSKRTYIPK---K--------------------NG---K-------KRPLGIPSFDDKLVQEVVRMILE-
--AIYEGS-FEHTSHGF---RPKRS-----CHTALIDIQKT----F----TA---------------------
VKW---FIEGDIKGFF-DNINHDVLINIL--RE--R-IA---------DER-FLRLIR--KFLNAGY---V--
---E--D--
------------------------------------W---------V---FH--R----TY------------
----
-----------------------------S-GTPQGGIIS-PILANIYLD-KFD-------------------
-----KYI-KEYINRFNKG--VTRK-GDARYKL----YEQRRYRLAKK-LK----------NE---
KDVKVRKQMTAEIKRLREERNNYPARNEMDSSIKRLKYVRYADDFLI-GIT------GNLED----CK-
TVKEDIKNYLNEALKLELSDEKTLITN----A---Q------K--------------------PAKFLGYDV-
>DS_Bacteroidetesfid|87116544|locus|VBIAliFin145170_0639| [Alistipes
finegoldii DSM 17242]
GSDGKTI--------------------DGMSLK---------RIENLID---ALKD------ESY---
QPKPARRTYIPK--KN---------------------G---N-------MRPLGIPSIDDKLVQEVLRMLLE-
--AIYEGS-FENTSHGF---RPKRS-----CHTALIQVQKN----F----TA---------------------
AKW---FIEGDIEGFF-DNINHDVLIGIL--KE--R-IA---------DDR-FIRLMW--KFLKAGY---I--
------E--
------------------------------------D-------------WTFHR----TY------------
----
-----------------------------S-GTPQGGIIS-PILANIYLD-KLD-------------------
-----KYMKEYACQFDRGDRRAMNLEYKRYSRKIWWLGTKLKQTKDKDTRKELIDAIKQHQK-----------
-----------NRMHLPSVDEMDEGYRRIKYVRYADDFII-GVI------GSKSD----CE-
AIKEDIKNFLGEKLKLTLSEEKTLITH----G--NR---------------------------KAKFLGYEI-
>DS_Bacteroidetesfid|87116554|locus|VBIAliFin145170_0644| [Alistipes
finegoldii DSM 17242]
GSDGRSI--------------------DEMSLA---------RIETLIA---SLKD------ESY---
QPHPSRRVHIPK--KN---------------------G---K-------TRPLGIPAFEDKLVQEVVRMILE-
--AIYEGH-FETTSHGF---RPKRS-----CHTALLHIQKT----F----SG---------------------
AKW---FIEGDIKGFF-DNIDHDVLVGIL--RE--R-IS---------DDR-FIRLIR--KFLKAGY---V--
------E--
------------------------------------D-------------WTFHN----TY------------
----
-----------------------------S-GMPQGGIVS-PILANIYLD-KLD-------------------
-----KYVKEY--------------IRHFDMGTKRRPGKESNDLANERKRT--------
VRKLKKVKDGTEKAALVARLKAIEQERAAFPSGDEMDGSYRRLKYIRYADDFIL-GVI------GSKED----
AL-RIKEDIKSFLSESLALELSEEKTLITH----T--GK---------------------------
SAKFLGYEI-
>DS_clostridiafid|54666312|locus|VBIDesCar168000_0691| [Desulfotomaculum
carboxydivorans CO1SRB]

MRRQYIPK--KN---------------------G---K-------LRPLGIPNIEDRIVQQAIVNVLS---
PKCEEHIFHKWSCGY---RPNLG-----IKRVMQIILWN----I----ET-------------G-------
YNH---IYDCDIKGFF-DNIPHKKLMKVL--TK--Y-IA---------DGT-VLDMIW--AWLKAGY---M--
---E--E--

------------------------------------G-------------KFH-P----TD------------
----
-----------------------------S-GTPQGGVIS-PLLANLYLN-ELD-------------------
-----WTL-EE--------------------------------------HG---------VR-----------
------------------------------FVRYADDFLL--FA------KSKED----IE-
RAAEVAKTTLD-ELGLEVSIEKTRFVD----F-DKD---------------------------DFNFVGFSF-
>DS_Bacillifid|58992730|locus|VBIStrEqu204605_0781| [Streptococcus equi
subsp. zooepidemicus ATCC 35246]
GTDGKTI--------------------VE-IQK--------LPIEMVIK---TIRNKL----NYY---
QPKNVRRVEIPK---D--------------------NG---K-------TRPLGIPSIWDRLIQQCVLQVLE-
--PICEAK-FHERNNGF---RPYRS-----TQNAIAQCYKM----AQI--QN---------------------
LHF---VVDVDITGFF-DNIDHSKLIRQL-WGL--G-VQ---------DRK-LIMIIK--QMLKADI---L--
---F--K---
------------------------------------D---------I---VI--T----PE------------
----
-----------------------------T-GTPQGGILS-PLLANVVLN-ELD-------------------
-----WWV-ANQWEMFKI---KEGS-TGYEFTK-VDNEGNILTIDRTQ-KW----------N-----------
KLRAK------------------TGLKEMYITRYADDFKI--FC------RDYAT----AV-
KVMKATNLWLAENLHLQTSDEKSGITN----L--RK------N--------------------YTTFLGIKF-
>DS_En.fm.I1/NZ_AAAK03000007/10877..13634/Enterococcus faecium/Bacterial
B
GTDGTII--------------------KD-IGK--------LPAETVVK---KVRYIVAGSPHGY---
RPKPVRRKEIPK---P--------------------NG---K-------TRPLGIPCMWDRLIQQCIKQVLE-
--PICEAK-FSENSYGF---RPNRS-----VENAIKATYNR----LQI--SQ---------------------
LHY---VIEFDIKGFF-DNVNHSKLIKQI-WAM--G-IR---------DKH-LIFILK--RILKAPI---K--
--MT--N--
------------------------------------G---------T---IT--Y----PE------------
----
-----------------------------K-GTPQGGIIS-PLLANIVLN-ELD-------------------
-----HWV-ESQWQENPV---T------KNYVV---HINKSGSPCKSN-AY----------K-----------
EMKK-------------------TKLKEMYMVRYADDFRV--FC------RYKES----AE-
KAKIAITQWIEQRLKLEVSQEKTRIVN----V--RK------R--------------------YSDFLGFKI-
>DS_clostridiafid|42996817|locus|VBIEubSir135646_1742| [Eubacterium
siraeum 70/3]
GTDKLKI--------------------SD-IGK--------LTADEVTA---RVRRIVKGGKNGY---
TPRSVRRKEIPK---P--------------------NG---S-------TRPLGIPCIWDRLVQQCIKQVME-
--PICEAR-FSNNSYGF---RPNRS-----VENAIAAIYRL----MQR--SG---------------------
LYY---VVEFDIKGFF-DNVDHSKLIKQL-WSL--N-IR---------DKE-LLYVIR--RILKAPI---L--
--MP--D--
------------------------------------G---------H---IE--H----PA------------
----
-----------------------------K-GTPQGGIIS-PLLANVVLN-ELD-------------------
-----HWI-ESQWQCNPV---T------ENYSY---RENATGCPIQSH-AY----------R-----------
AMRN-------------------TRLKEMYIVRYADDFRI--LC------RTKEQ----AD-
RTLIAVTHWLKERLRLDVSPEKTRVVD----T--RR------S--------------------YSEFLGFKI-
>DS_B.a.I2/AE011190/30945..33835/Bacillus_extraction anthracis/Bacterial
B
ACDNVNI--------------------KN-IEG----MEQSYFLNEVKR---RFQN--------Y---
QPQKVRRKEISK---P--------------------NG---Q-------TRPLGIPAMWDRIIQQCILQVME-
--PICEAH-FSNRSYGF---RPNRS-----AEHALADASVR----V----NK-------------------
QNLTY---VVDVDIKGFF-DEVNHVKLMRQL-WTLGIR------------DKQ-LLVIIR--KILKAPV----
-----QMPD--
--------------------------------------G-------------TTMFP----TK----------
----

```
---------------------------------GTPQGGILS-PILANVNLN-EFD-----------------
-------WWISRQ------------------------WETFKAKKVKPRCMRG--------IWC---------
--------------NDVVTTQLTKTSKMKPMYIVRYADDFKI--FT------NTRSN----AE-
KIFKATQMWLEERLKLSISAEKSKVTN----L-TKQ---------------------------QSEFLGFTL-
>DS_En.fm.I3/FN424376.1/17411..20180/Enterococcus faecium/Bacterial B
GIDGKTI--------------------KD-IEK----LTTERYLDIVKK---RFKF--------Y---
KPRKVKRTEIPK---P--------------------NG---K-------TRPLGIPSIWDRVAQQCILQVLE-
--PICEAK-FNPHSHGF---RPNRS-----AEHAIADCAKK--MNI----IK---------------------
MGY---CVDIDIQGFF-DEVWHSKLMRQM-WTMGIR------------DKE-LLTIIR--KMLKAPV------
---VLPN------------------------------------------------------------------
------------------------------------G-------------TIQFP----EK------------
-------------------------------------------------------------------------
-------------------------------GTPQGGILS-PLLANINLS-EFD-------------------
-----WWVSEQ------------------------WET-RHMSEIKTQYNANGTEHMGNHHR-----------
------------------KMRSHTKLKEFYIVRYADDFKL--FC------HNRKT----AE-
LLYHASIQWLEQRLHLPVSIEKSKITN----L-RKE---------------------------SSEFLGFNL-
>DS_Bacillifid|202064373|locus|VBICarSp264223_1846| [Carnobacterium sp.
WN1359]
GVDDITI--------------------KD-IEN----LEQTIFVEMVRK---RFSN--------Y---
SPRKVRRVEIPK---P--------------------NG---K-------TRPLGIPSIWDRIAQQCILQVIE-
--PICEAK-FNKHSYGF---RPNRS-----TEHAIADMLFR--INQ----QK---------------------
LHY---VVDVDLQGFF-DEINHKKLMNQV-WTLGIH------------DKQ-LLVIIR--KMLSAPI------
---VLKN------------------------------------------------------------------
------------------------------------G-------------SIMHP----VK------------
-------------------------------------------------------------------------
-------------------------------GTPQGGILS-PLLANISLN-EFD-------------------
-----WWISNQ------------------------WETFETRKKYAAAVMGNGTKNRGLTYR-----------
------------------MLRKNSKLKEIYIVRYADDFKL--IT------SNRRD----AE-
KIFIASQMWLKERLGLPISKEKSKITN----L-RKE---------------------------ESEFLGFTI-
>DS_Bacillifid|18859935|locus|VBIBacCer118379_5432| [Bacillus cereus ATCC
10987]
GIDGVTI--------------------KD-VEK----LSQEDFIKIVQK---RF--------SNY---
TPRKVRRVEIPK---P--------------------NG---K-------TRPLGIPSMWDRIAQQCIKQVLE-
--PICEAK-FNKHSHGF---RPNRS-----PETAMADATLR----V----NR-------------------
SHMQY---VVNVDIQGFF-DEVNHKKLMRQL-WTMGIR------------DKQ-LLVIIR--KMLKAPI----
-----VLPN----------------------------------------------------------------
--------------------------------------G-------------EMQYP----NK----------
-------------------------------------------------------------------------
---------------------------------GTPQGGILS-PLLANINLN-EFD-----------------
-------WWITNQ------------------------WEDRLLKELSLTIKKGGHVDKYPHYSK---------
---------------------MRKTTALKEMYIVRYADDFKI--FT------ATKSN----AQ-
KIFKACEMWLQERLKLPISKEKSKITN----L-RKE---------------------------SSEFLGFEI-
>DS_Bacillifid|190447919|locus|VBIEntSp299569_0686| [Enterococcus sp.
HSIEG1]
GVDGITI--------------------SD-IER----LNENDFVEIIRA---NL--------SNY---
RPGPVRRVYIPK--KN---------------------G---K-------KRPLGIPNLYDRIIQQTIKQVIE-
--PIVEAK-FFKHSYGF---RPLRS-----VEQAMGRMHSV----I----NN-------------------
VQLHY---VVDVDIKGFF-DNVNHNLLRHQI-WNMGIR------------DTK-LIAIIS--KILRAEI---
V-----G--E---------------------------------------------------------------
---------------------------------------G-----------------T----PV---------
-------------------------------------------------------------------------
--------------------------------K-GTPQGGVLS-PLLANIVLN-DLD----------------
--------QWIASQ-------------------WENFPSKHRYSRGKLHRALKG--------TTL--------
```

-----------------------------KEGYLVRYADDFKL--LT------RSYSM----AK-
RWYTAIRGYIEKHLKLEISPEKSGITN----L-RKK---------------------------RTEFLGFEI-
>DS_Bacillifid|87137209|locus|VBIHalHal165146_0228| [Halobacillus halophilus DSM 2266]
GTDGKTI--------------------DD-MKE----LSENDLVNEVRS---KLQN--------Y---
HPKKVRREWIEK--EN---------------------G---K-------WRPLGIPCILDRVIQQCFKQVLE-
--PIVESQ-FFKHSYGF---RPLRS-----AHHAMARIQFL----INH--SQ---------------------
LHY---VVDVDIKSFF-DNVNHRLLKKQLWNIG----IQ---------DRK-VLACIS--KMITSEI------
------D--
------------------------------------G-------------EGV------PD------------
----
-----------------------------K-GSPQGGILS-PLLSNVVLN-DLD-------------------
-----QWVADQ---------------------WEVFPLTKSYSSDDARRRA---------RK-----------
----------------------QTNLKQGYLVRYADDFKI--LC------RDGKT----AQ-
RWYHAVRLYLKERLKLDISPEKSQIVN----L-RKR---------------------------ESEFLGFTI-
>DS_Bacillifid|22412306|locus|VBILysSph89750_0101| Mobile element protein [Lysinibacillus sphaericus C341]
GTDGITI--------------------EQ-YKI--------EDVETFVD---EIRATL----KNY---
KPQTVRRVEIPK---P--------------------NG---K-------TRPLGIPTMRDRLIQQMFKQILE-
--PICEAR-FYNHSYGF---RPNRS-----THHAMGRCQFL----ANI--AL---------------------
NQH---VVDIDIQGFF-DNVSHSKLLKQM-YSI--G-IC---------DKR-VLSVVS--KMLKAPI---K--
---G--I--
------------------------------------G---------I------------PT------------
----
-----------------------------K-GTPQGGILS-PLLSNIVLN-DLD-------------------
-----WWI-SNQWENMKT---K------FNYKE------------RKN-KV----------L-----------
MIKRT------------------TTLKEMYIVRYADDFKI--FT------KSHKN----AI-
KLYHAVKGYLKNHLNLDISNEKSKITN----L--RK------R--------------------ASEFLGFSL-
>DS_Bacillifid|202109853|locus|VBIEntMun281267_2992| [Enterococcus mundtii QU 25]
GTDGITI--------------------DD-YKL--------ANIEIFVS---YIRSVL----SNY---
KPQKVRRVYIPK--SN---------------------G---K-------KRPLGIPTMRDRIIQQMFLQILE-
--PICEAQ-FYNHSYGF---RPNRS-----TKHAMARCKFL----T----RK-------------N-------
FHY---VVDIDIKGFF-DNVNHNKLIKQL--YT--IGIK---------DKR-VLAILA--KMLKATI------
------E--
------------------------------------G-------------EGIPK------------------
----
-----------------------------K-GTPQGGILS-PLLSNVVLN-ELD-------------------
-----WWIANQ-------------------------------WEFLKTKENYHPAARLKSLK-----------
---------------------RKTTLKEMFIVRYADDFKI--FT------KDHQS----AI-
RIYHGVKGYLSNHLSLDISPEKSKITN----LRKRD----------------------------SEFLGFSL-
>DS_Bacillifid|31950635|locus|VBIBacPse80461_3982| [Bacillus pseudofirmus OF4]
GTDGYTI--------------------IH-LAE--------KNKESFIE---EMRLRL----ENY---
KPQRVRRVLIDK---N--------------------YG-T-D-------KRPLGIPTIADRIIQQMFLQVLE-
--PICEAK-FYNHSYGF---RPLRS-----TRHAIARVQTL----INI--NK---------------------
LHY---TVDIDIKGFF-DNVNHNLLIKQL-WNI--G-VK---------DKR-VLAIIS--KMLKAPI---Q--
---K--E--
------------------------------------G---------I------------PK------------
----
-----------------------------K-GVPQGGILS-PLLSNVVLN-DLD-------------------
-----QWV-AGQWECFNT---K------HQYSG------------NDV-KI----------A-----------
NLKRA------------------SNLKEGYIVRYADDFRI--LA------RDHNT----AW-
KWFHAVKGYLKDRLKLEISNEKSRVIN----L--RK------K--------------------SSDFLSYKI-

>DS_Bacillifid|18848241|locus|VBIBacCer122868_5594| [Bacillus cereus
AH820]
GTDDKTI--------------------LD-LAN--------TNQDEFIH---YMRELV----LNY---
KPKSVRRVWIDK---N--------------------YS-K-G-------KRPLGIPCIQDRIVQQMFLNVLE-
--PICEGK-FYNHSYGF---RPTRT-----TRHAVARVQTL----VNI--NK---------------------
YHY---TVDIDIKGFF-DNVNHSILLKQV-WNI--G-IR---------DKR-VIAVIS--KMLKAPI---K--
---G--E--
------------------------------------G---------I------------PT------------
----
-----------------------------K-GVPQGGILS-PLLSNIVLN-DLD-------------------
-----QWV-ADQWECFET---R------YQYSV------------NYS-KY----------V-----------
NLRRN------------------SKLKEGFLVRYADDFRI--MT------NTHDS----AV-
KWFHAVVDFLNKRLKLEISPNKSKIIN----L--RK------K--------------------SSSFLGYKF-
>DS_Bacillifid|47119490|locus|VBIEntFae176554_2204| [Enterococcus
faecalis 62]
GTDSFTI--------------------DN-YKE----MNQAEFIHLILS---QL--------ENY---
KSKSIKRVMIPK---P--------------------NG---E-------KRPLGIPCMIDRIIQQMFKQVLE-
--PICEAK-FYEHSYGF---RPLRS-----AKHALGRIMYL--INI----SK---------------------
MHY---AVDIDIKGFF-DNVNHRLLIKQL-WNIGIC------------DKR-VLAILS--KSLKSPI------
------Q--
------------------------------------G-------------EGISS------------------
----
-----------------------------K-GTIQGGIIS-PLLSNVVLN-DLD-------------------
-----HWVSKQ---------------------WHTFETKYPYTKGYNKFRA---------LR-----------
----------------------DTNLKQGYIVRYADDFKI--MT------NDYPS----AL-
KWFHAVKLYLKDRLKLDISNEKSKIVN----L-RKR---------------------------KSEFLGFTI-
>DS_Bacillifid|101938694|locus|VBIBacThu242010_5758| [Bacillus
thuringiensis MC28]
GIDSFAI--------------------DQ-YKS----MDKAEFLNLVRN---RL--------NQY---
KPKAVKRVFIPK---P--------------------NG---D-------KRPLGIPTMFDRLIQQMIKQILE-
--PICEAK-FYEHSYGF---RPLRG-----ARHAISRVMYL--ISR----NT---------------------
FHY---AVEIDIKGFF-DNVNHTLLLKQL-WNMGIK------------DKR-VLKLIY--LILKAPI------
------K--
------------------------------------G-------------VGIPR------------------
----
-----------------------------K-GTPQGGILS-PLLSNVVLN-DLD-------------------
-----QWIARQ---------------------WHHFQSDYDYTEPGNRSRA---------LK-----------
----------------------RTKLKQGYIVRYADDFKI--MA------KDFRT----AQ-
KWFMATKLYLKERLKLDISPGKSRIIN----L-RKN---------------------------KSEFLGYSL-
>DS_Bacillifid|18820991|locus|VBIBacCer84800_3811| [Bacillus cereus
03BB102]
GTNGHTI--------------------KH-LNK--------IDADKLIR---LTQKRL----ENY---
MPHAVRRLFISK---P--------------------NG---K-------MRPLGIPTIEDRLIQQMFQQVLE-
--PIVEGK-FHPQSYGF---RPKRG-----THDALARCYHM----VNH--SH---------------------
QHF---VVDIDIKGFF-DNVNHKKLMRQL-WTI--G-IR---------DKK-VLSIIK--KMLKAEV---T--
---G--E--
------------------------------------G---------I------------PV------------
----
-----------------------------K-GTPQGGILS-PLLANVVLN-ELD-------------------
-----WWV-SNQWETKPT---R------VPYKL------------KRN-KT----------D-----------
ALKK-------------------TRLKPMYLVRYADDFKI--FT------NSYDN----AR-
KIKIAVEKWLKERLGLEISEEKSKITN----L--RK------N--------------------GTDFLGIRF-
>DS_Bacillifid|202104716|locus|VBIEntMun281267_0501| [Enterococcus
mundtii QU 25]

GVDNKNI--------------------DD-LKS----IPDTDFISIVQT---KLSE--------Y---
KPQPVKRVEIPK---P--------------------NG---K-------TRPLGIPTIWDRIVQQCLLQVLE-
--PIMEAK-FHDKNYGF---RPNRS-----AHHAFAQAVRM--AQV----SK---------------------
LTF---VVDIDIEGFF-DNVNHSKLIKQL-WSLGVR------------DKW-LLGVIR--AMLKAPI------
---IHKD--
------------------------------------G-------------HIEHP----KK------------
----
-------------------------------GTPQGGILS-PLLANVVLN-ELD-------------------
-----WWISSQ-----------------WETHPTRHNYDWYHAEKEYWNKGNKYRALRGTSL-----------
--------------------------KEIYIVRYADDFKI--FC------RKRSD----AD-
KIFLATKLWLKERLKLDISQEKSKVVN----L-KKQ---------------------------KSEFLGFTL-
>DS_clostridiafid|19436501|locus|VBICloCel57783_2839| [Clostridium
cellulolyticum H10]
GTDTLNI--------------------KD-IEK--------LSVEKLVE---MMQRKL----AWY---
QPKPVKRVEIPK---P--------------------NG---K-------TRPLGIPTIVDRLVQQCILQVLE-
--PICEAK-FYERSNGF---RPNRS-----AEHAMAQCYRM----VQK--QN---------------------
LYF---VVDVDIKGFF-DNVNHSKLIRQM-WAM--G-IR---------DKQ-LICIIK--QMLKAPV---V--
--MP--D--
------------------------------------G---------E---TL--Y----PT------------
----
-----------------------------K-GTPQGGILS-PLLANIVLN-ELD-------------------
-----WWI-SSQWEDMLT---H------REYYV---SVNNNGSLNKSG-VF----------R-----------
TLRR-------------------SALKEMYIVRYADDFKI--FC------RKRSD----AN-
KIFVAVKKWLKDRLKLEISEEKSKVVN----L--KK------H--------------------YSEFLGFQF-
>DS_Bacillifid|190355818|locus|VBIStrAng166616_1315| [Streptococcus
anginosus C238]
GVDGRTI--------------------KH-LSR----LNEEEYISLIQK---QF--------HWY---
KPRPVKRVEILK---P--------------------NG---K-------IRPLGIPTIVDRIVQQCILQILE-
--PICEAK-FHDSSYGF---RPNRS-----TEHAIAECARL--MQI----QH---------------------
LHY---VVDIDIQGFF-DNVYHAKLIRQL-WNLGIQ------------DKK-LLCIIK--EMLKADI------
---VMPD--
------------------------------------K-------------EVITP----TK------------
----
-------------------------------GTPQGGILS-PLLSNVVLN-ELD-------------------
-----WWVSSQ------------------------WLTMPTHYPYKQRTNSQGTEIKSHTYR-----------
-------------------ALRTSNLKEIYIVRYADDFKI--FC------RNYYD----AK-
RTYQAVTKWLQDRLKLNVSEEKSKITN----L-KQR---------------------------YSEFLGFKL-
>DS_E.f.I3/AE016830/2249712..2252481/Enterococcus faecalis/Bacterial B
GVDKRTI--------------------AD-LAK----LSEEEYVRLIRK---QF--------SNY---
HPGPVRRVEIPK---P--------------------NG---K-------TRPLGIPTIVDRIVQQCILQVME-
--PICEAK-FSENSNGF---RPNRS-----AETAIAQCMRL--IQV----QH---------------------
LYH---VVDLDIKGFF-DNISHTKLIRQI-WALGIR------------DKK-LLCIIK--EMLKAPV------
---VLPN--
------------------------------------G-------------EKTYP----AR------------
----
-------------------------------GTPQGGILS-PLLANIVLN-ELD-------------------
-----WWIASQ------------------------WEEMPTKTKFKTRSNAQGTEIKSHAYR-----------
-------------------ALRRSRLKEMHAVRYADDFKI--FC------ATHED----AV-
RAYKATELWLKDRLGLEISPDKSKVVN----L-KRQ---------------------------YSDFLGFKL-
>DS_clostridiafid|115616442|locus|VBIDehSp228777_1269| [Dehalobacter sp.
CF]
GTDGLTI--------------------KD-IAG----MTNQEVITMVKR---RLKN--------F---
TPQSVRRVEILK--DN---------------------G---Q-------NRPLGIPTMSDRLIQACIYQILE-
--PICEAR-FHNHSYGF---RPTRR-----TEHALATMHRM--INI----QH---------------------

```
LHF---VVDVDIKGFF-DNVDHGKLLKQMWTMG----IQ---------DKN-LLCIIS--AMLKAEI------
------E------------------------------------------------------------------
------------------------------------G-------------IGI------PN------------
-------------------------------------------------------------------------
-----------------------------K-GVPQGGLCS-PLFSNVVLN-ELD-------------------
-----WWISDQ-----------------WESYETSYPYKRNEGKIRAIRRG--------SKL-----------
--------------------------KECYIIRYCDDFKI--MC------PTRDV----AE-
RMFVAVKLWLKERLNLEISSEKSKITN----L-RKK---------------------------SSEFLGFKI-
>DS_C.d.I1/X98606/13..2658/Clostridium difficile/Bacterial B
GTNKRTI--------------------IDVGEE---------NPYQLVQ---YVQNRF----NNF---
QPHSIRRVEIPK---P--------------------NG---K-------TRPLGIPTIEDRLVQQCIKQILE-
--PILEAK-FHKHSYGF---RPERS-----SHHAIAIFQQW----T----FK-------------G-------
FHY---VVDIDIKGFF-DNVNHGKLVKQLWTMK----IR---------DKT-FISILS--RMLKAEV------
------K------------------------------------------------------------------
------------------------------------G-------------IGKST------------------
-------------------------------------------------------------------------
-----------------------------K-GTPQGGILS-PLLANVVLN-ELD-------------------
-----WWIDSQ---------------------WDGFPTKRKYSSLLSKTQS---------IR-----------
---------------------KYSNLKEIKIVRYADDFKI--MC------KDYHT----AQ-
KIFLATKQWLKVRLDLDISPEKSKVTN----L-RKN---------------------------YSDFLGFKL-
>DS_Bacillifid|67659680|locus|VBIEntFae233823_1913| [Enterococcus faecium
Aus0004]
GTDGMTI--------------------DD-IKQ----LSNAEIVATVRE---SL--------SNY---
RPKSVRRVFIPK---A---------------------G-SDK-------MRPLGIPCIWDRLVQQCILQVLE-
--PICEPK-FHNHSYGF---RANRS-----AHHAVSRVTTL--INL----SK---------------------
YHY---CVDVDIKGFF-DNVNHGKLLKQI-WTLGIR------------DKR-LICIIS--KMLKAEI------
---DG-E------------------------------------------------------------------
------------------------------------G-----------------V----PE------------
-------------------------------------------------------------------------
-----------------------------K-GTPQGGLLS-PLLSLIVLN-ELD-------------------
-----WWVSSQWETFQP---K--NRSKNG-------W-LQYAKKYTKLKSG---------F-----------
------------------------------IVRYADDFKI--MC------STYGE----AQ-
RFYHSTVDFLNKRLKLEISPEKSKVVN----L-KKN---------------------------SSDFLGFKI-
>DS_Bacillifid|18825078|locus|VBIBacCer120511_0128| [Bacillus cereus
AH187]
GVDNLTI--------------------KD-IWH----LNDTKIIHEVRK---RL--------NNY---
QPQAVKRVLIPK---E---------------------G-SDK-------KRPLGIPTIWDRLVQQSILQVLE-
--PICEAK-FHNHSYGF---RPNRS-----THHALSRVVSL--INI----GH---------------------
QHY---CVDIDIKGFF-DNVCHKKLLRQM-WTLGIR------------DKS-LLCVIS--KILKSEI------
---EG-E------------------------------------------------------------------
------------------------------------G-----------------I----PN------------
-------------------------------------------------------------------------
-----------------------------K-GTPQGGIIS-PLLSNIVLN-ELD-------------------
-----WWI-SSQWETYKP---HRISTRHLG-------F-RQYARKYTNLKC----------------------
----------------------------GYVVRYADDFKI--MC------RTYDE----AQ-
RFYHATVDFLKSRLGLEINPKKSKVVN----L-KKN---------------------------SSVFLGFKI-
>DS_Bacillifid|201989473|locus|VBIBacThu93926_0768| [Bacillus
thuringiensis YBT1518]
GVDGLTI--------------------KD-VRQ----LNDFQVINQVRK---RLMN--------Y---
RPSPVRRVYIPKEGSD-------------------------K-------KRPLGIPTIWDRLVQQCILQVLE-
--PICEAK-FHNHNYGF---RPNRS-----THHALSRMVSL--INV----GK---------------------
HHY---CVDIDIKGFF-DNVQHGKLLKQM-WAIGIR------------DKR-LLSIIS--NLLKAEI---I--
-------------------------------------------------------------------------
------------------------------------G-------------EGIPS------------------
```

```
-------------------------------------------------------------------------
-----------------------------K-GTPQGGILS-PLLSNIVLN-ELD-------------------
-----WWISNQ-------------------------WETYKPHRFKDGPNGFTTYARKYTNL-----------
--------------------------KGGYIVRYADDFKI--MC------RTYEE----AQ-
RFYHATVDFLKARLGLEINPEKSKVVH----L-KKN---------------------------SSDFLGFKI-
>DS_Bacillifid|101939315|locus|VBIBacThu242010_6066| [Bacillus
thuringiensis MC28]
GTDGMTI--------------------ND-IKM--------LSTDEVIE---KVKMMF----GWY---
EPQSVRRVFIPK---P--------------------NG---N-------RRPLGIPTIWDRLFQQCVLQILE-
--PICEAK-FHNHSYGF---RPNRS-----THHALARMKSL----VNRKGNG---------------------
FHY---CVDIDIKGFF-DNVHHGKLLKQL-WTIGIR------------DKK-LLSIIS--RLLKAEI------
-------------------------------------------------------------------------
--------------------------------------------------VNEGV----PQ------------
-------------------------------------------------------------------------
-----------------------------K-GTPQGGILS-PLLSNIVLN-ELD-------------------
-----WWVSNQ---------------------WETIKTSHPY----KGNSD--------KYR-----------
-------------------ALKKSKLKECFLIRYADDAKI--LC------RDYVT----AL-
KMFEATKDFLRTRLHLDISLEKSKIIN----L-RKK---------------------------ASHFLGFTV-
>DS_clostridiafid|54454697|locus|VBICloBot178872_0058| [Clostridium
botulinum BKT015925]
GTDGSTI--------------------KD-INN--------IDIDEVIT---KIKTMF----DFY---
TPKSIRRVEIPK---A--------------------NG---K-------TRPLGIPTIWDRLFQQCILQVLE-
--PICEAK-FHKHSYGF---RPNRS-----THHAITRSVYL----INI--TK---------------------
LYH---CVDVDIKGFF-DNVNHGKLLKQL-WALGVK------------DKK-LLKIIS--VMLKAPI------
-------------------------------------------------------------------------
--------------------------------------------------EGIGI----PT------------
-------------------------------------------------------------------------
-----------------------------K-GVPQGGILS-PLLSNIVLN-ELD-------------------
-----WWVSNQ---------------------WETFKTDKDYTKYRTSKTGKIVVDHSIRNK-----------
-------------------MLKKSKLKEIYIVRYADDFKI--FC------RTRSQ----AK-
AIDIAVGDMLKNRLGLECSAEKSKVLN----L-KKS---------------------------YSEFLGFKM-
>DS_B.me.I1/AB022308/3853..6569/Bacillus megaterium/Bacterial B
GDDGLTI--------------------ED-INR--------LSVSEVVS---TIQRMF----EYY---
TPQAVRRVFIPK---A--------------------NG---K-------TRPLGIPTIWDRLFQQCILQVLE-
--PICEAK-FYKHSYGF---RPNRN-----THHAKARFETL----INR--AC---------------------
LYH---CVDVDIKGFF-DNVNHAKLIKQL-WSLGIR------------DKA-LLSIIS--RLLKAEI------
-------------------------------------------------------------------------
--------------------------------------------------IGEGF----PK------------
-------------------------------------------------------------------------
-----------------------------K-GTPQGGILS-PLLSNIVLN-ELD-------------------
-----WWVSNQ---------------------WESFETHKLY----KSNLG--------RYN-----------
-------------------ALKQSNLKHCYIVRYADDFKI--LC------RTRSQ----AI-
KMYYAVNDFLHTRLRLEISEQKSKVVN----L-KKN---------------------------SSEFLGFRS-
>DS_Bacillifid|38137486|locus|VBIBacThu148000_5492| [Bacillus
thuringiensis BMB171]
GSNDTTI--------------------LE-IAE--------QNLTTFVA---KVQKAL----ENY---
NPKPIRRVYIPK---R--------------------NG---D-------KRPLGIPTMEDRIVQQCIKQILE-
--PICEAK-FYNHSYGF---RPNRN-----AKHAIVRAMSL----MNI--SK---------------------
FHY---VVDIDIKGFF-DNVNHGKLLKQI-WSL--G-IR---------DKS-LLSIIS--KILKTEI---E--
---N--V------------------------------------------------------------------
------------------------------------G---------K------------ME------------
-------------------------------------------------------------------------
-----------------------------K-GTPQGGIIS-PLLSNIVLN-ELD-------------------
-----WWI-SSQWETMIT---R------HNYES---IDKRNNTIIRSH-KY----------T-----------
```

ALRRT------------------SNLKEMFLVRYADDFKI--FC------KDFNS----AQ-
KTLIAVKKWLKNRLGLEVNNEKSKVTN----L--RR------N--------------------YTEFLGFKL-
>DS_clostridiafid|19408375|locus|VBICloBot19908_0265| [Clostridium botulinum Ba4 str. 657]
GTNHKTI--------------------NDIAGE---------SEDEIIE---YVRKRL----NKF---
YPHSVKRIYIPK--NN---------------------G---D-------KRPLGIPTIEDRLIQRSILQVLE-
--PICEAK-FHPHSYGF---RPNRS-----TEHAIARAMTL--INM----NK---------------------
LHY---VVDVDIKGFF-DNVNHGKLLKQL-WTLGIK------------DKK-LIKIIS--LMLKAQI---K--
------D--
------------------------------------G-------------SMITN----PV------------
----
-----------------------------K-GTPQGGIIS-PLLANVVLN-ELD-------------------
-----WWISSQ---------------------WETFETKHNYSKLRTFKNGTTTIDKSHKYR-----------
-------------------ALRNGKLKEIYIVRYADDFKV--FC------KNPKD----AE-
KIFIAIKLWLKERLDLETSPEKSKVTN----L-RKH---------------------------PTEFLGFEL-
>DS_clostridiafid|19462591|locus|VBICloKlu111549_0642| [Clostridium kluyveri DSM 555]
GVNTNTI--------------------MD-IGE--------ENPDELAI---YVRERL----INY---
KPQPVRRVEIPK---P--------------------NG---K-------MRPLGIPTIEDRIIQQCIKQVLE-
--PICEAK-FHKDSYGF---RPNRS-----THHAIARTYSL----ANI--NK---------------------
LTY---VVDIDIKGFF-DNVNHSKLLKQM-WTM--G-IQ---------DKN-LLCVIS--KMLKAEI---K--
---G--V--
------------------------------------G---------I------------PN------------
----
-----------------------------K-GTPQGGILS-PLLSNIVLN-ELD-------------------
-----WWI-SNQWQTLKS---K------FPYKR-----------EIF-KY-----------Q-----------
ALKR-------------------SKLKEVYIVRYADDFKL--FC------RSYNN----AK-
KIFKAVTMWLKERLGLEINEEKSSIVN----L--KQ------K--------------------YSEFLGFKF-
>DS_clostridiafid|115343359|locus|VBIHalHal149681_0148| [Halobacteroides halobius DSM 5150]
GTNNKTI--------------------KD-LEE--------KSTEELVE---YVRNRL----EYY---
VPQSVRRVYIPK---P--------------------DG---R-------KRPLGIPTIKDRLIQQCIKQVLE-
--PICEAK-FHNHSYGF---RPNRS-----TKHAIARIMYL----INF--SK---------------------
LHY---TVDIDIKSFF-DNVDHNKLKKQL-WSM--G-IR---------DKK-LISILG--NMLEAKI---E--
---G--E--
------------------------------------G---------V------------PE------------
----
-----------------------------K-GTPQGGIIS-PLLSNIVLN-EMD-------------------
-----WWI-SNQWETFKT---D------YKYNR-----------KGD-KI-----------T-----------
AIKK-------------------TNLKEIYIIRYADDFKI--MC------RDFET----AS-
KIKIATIKWLKERLNLEVSEKKTSITN----L--KK------N--------------------HTEFLGIKL-
>DS_clostridiafid|115343005|locus|VBIHalHal149681_0330| [Halobacteroides halobius DSM 5150]
GTNNKTI--------------------KD-LEE--------LTTQKLVD---YVRNRL----EYY---
IPQSVRRVYIPK---P--------------------DG---R-------KRPLGIPTIEDRLIQQCIKQVLE-
--PICEAK-FHNHSYGF---RPNRS-----TKHAIARTMRL----INQ--SK---------------------
LHY---VVDVDIKGFF-DNVDHAKLKKQM-WSM--G-IK---------DKK-LISIIG--NMLRAEI---E--
---G--E--
------------------------------------G---------I------------PD------------
----
-----------------------------K-GTPQGGIIS-PLLSNIVLN-ELD-------------------
-----WWV-SNQWETFET---D------FKYNQ-----------KSN-KY-----------Q-----------
ALKKR------------------SNLKEVYIVRYADDFKI--MC------RDYEI----AS-
KIKVATIQWLKERLNLDVSKKKTKITN----L--KR------S--------------------YTKFLGIKL-

>DS_clostridiafid|61450525|locus|VBISulAci142080_0388| [Sulfobacillus
acidophilus DSM 10332]
GSDGQVM--------------------SEILQQ---------QYPDIIQ---RVQSAL----HHY---
EPQLLRRVWIPK---P---------------------G---K-----AEKRPLGIPAMIDRIVQEILRSILE-
--PIMEAQ-FFEHSYGF---RPMRD-----AHQALARTTNL----V----HD------------TG-------
YHW---IVEGDIKGCF-DNIPHGKLLKQL-WHMGIR------------DRR-ILMIIK--QMLKAGI------
----
--LHEAP----HV------------

-----------------------------DQGTPQGGILS-PLLANVYLH-KLD-------------------
-----QWV-TR-------------------------EWEAKRTRFPYKKRRIRLEALQERSR-----------
-------------------------LKPAYFVRYADDWIL--IT------DCKAH----AV-
AWKQRIAQYLDQNLSLTLSQDKTKITN----V-RRQ---------------------------SIHFLGFQF-
>DS_G.k.I1/BA000043/1312755..1315536/Geobacillus kaustophilus/Bacterial B
GTDGKTI--------------------SDILTL---------NYDEAIN---FVKRCF----KKY---
TPNPIRRVHIPK---P---------------------G---K----K-EKRPLGILTIADRIIQECVRMVIE-
--PILEAQ-FFQHSYGF---RPYRD-----AKQAIERCVFI----C----NR------------IG-------
YNW---VIEGDIKGFF-DNVNHTILIKQL-WHMGIR------------DRR-MLMIIK--AMLKAGV---I--
----
--KET-K----IN------------

-----------------------------EMGTPQGGIIS-PLLANVYLH-KLD-------------------
-----QWI-TR--------------EWEEKKMRNGTTIRTAKYKSLRDHST---------IT-----------
--------------------------KPEFYVRYADDWVL--FT------NSRGN----AE-
KWKYRIKKYLKENLKLELSDDKTLITN-IKKK-------------------------------PMKFLGFKI-
>DS_Bacillifid|31950695|locus|VBIBacPse80461_4012| [Bacillus pseudofirmus
OF4]
GTDGETI--------------------DDILQD---------GYESVIS---RVRKCF----LAY---
NPKLLRRVHIDK-QVS---------------------K---D-------KRPLGIPAIIDRIIQECIRMIIE-
--PILEAQ-FFSHSYGF---RPYRS-----AEHALSKVTNTAYDTN---------------------------
YCW---VVEGDIKKFF-DNVNHTILIKKLYSMG----IR---------DRR-VLMIIK--AMLQCGV---L--
----
------------------------------------G-------------EAE-Q----TT------------
----
-----------------------------V-GTPQGGIIS-PLLANAYLD-SLD-------------------
-----HWI-TR--------------------EWENKETKHEYSRLDGKYRA---------LK-----------
---------------------NASNLKPAHFVRYADDWVL--IT------NSKAN----AI-
KWKQRIAKHLKEQLKLELSEEKTLITN-IKKK-------------------------------AIKFVGFHF-
>DS_B.c.I5/AE017195/84166..86938/Bacillus cereus/Bacterial B
GVDGKTI--------------------QDYLRL---------SEEKLIE---LIRGRL----TNF---
KAHLIKRVFIPK--AN---------------------G---G-------QRPLGIPTIEDRIIQQMMKQVLE-
--PVLEAQ-FFKYSFGF---RPERT-----TYHALERVKVL----V----HN------------TG-------
YHW---IVEGDIRQFF-DKVNHRILIKKL-WSMGIK------------DRR-ILCLIT--EFLKAGI------
----
--FKNII----RN------------

-----------------------------DNGTPQGGILS-PLLANVYLH-SFD-------------------
-----KWVAKQ---------------------FEEFTTRHEYSKHDHKLRG---------LK-----------
----------------------SSNLKPGYLIRYADDWVL--VT------NNKSH----AY-
RWKTVIKNFLQKELKLELSEEKTRITN----I-RHK---------------------------PIEFLGFKY-
>DS_Bacillifid|202001215|locus|VBIBacThu93926_6557| [Bacillus
thuringiensis YBT1518]
GVDSLTI--------------------ND-ILQ--------ADEEKVIH---LITNTI----RDY---
TPSMVRRVWIPK---A-G------------------KK---E-------LRPLGIPTILDRIIQQCVKQVIE-

--PICEAQ-FFPYSFGF---RPYRD-----GHMAIERVGSL----IHK--TK---------------------
YHW---IVEGDIRKFF-DKVNHNILLKNC-FKI--G-IQ---------DKR-VLMLIK--AMLKAGV---M--
----
------------------------------------H---------E---NT--K----TT------------
----
-----------------------------L-GTPQGGIIS-PILANIYLH-DFD-------------------
-----MWV-YNQWQNKKT---R------KNYAN------------KHS-RT----------T-----------
TLKRT------------------TKLKQGYLIRYADDWVI--VT------NSKTN----AI-
KWKKAVSHYLKDKLKLELSEEKTKITN----V--RK------K--------------------NIEFLGFKL-
>DS_Bacillifid|45223831|locus|VBIGeoSp94955_1285| [Geobacillus sp.
Y412MC52]
GIDQKIV--------------------DDYLLM---------PTEKVFG---MIKAKL----NDY---
KPIPVRRCNKPKGNAK-S---------------SKRKG---NSPNEEGETRPLGISAVTDRIIQEMLRIVLE-
--PIFEAQ-FYPHSYGF---RPYRS-----TEHALAWMLKI----ING--SK---------------------
LYW---VVKGDIESYF-DHINHKKLLNIM-WNMGVR------------DKR-VLCIVK--KMLKAGQ---V--
---I--Q--
------------------------------------G-------------KFY-P----TA------------
----
-----------------------------K-GIPQGGIIS-PLLANVYLN-SFD-------------------
-----WMVGQE----------------YEYHPNNANYREKKNALAALRNKG----------H-----------
--------------------------HPVFYIRYADDWVI--LT------DTKEY----AE-
KIREQCKQYLACELHLTLSDEKTFIAD-IREQ-------------------------------RVKFLGFCI-
>DS_Bacillifid|31950623|locus|VBIBacPse80461_3976| [Bacillus pseudofirmus
OF4]
GIDNKTI--------------------DY-YLH--------LPYEDLVS---QVQTCI----EDY---
NPEPVRRKYIPK---E-N------------------SD---K-------LRPLGIPTMIDRIIQEITRLVIE-
--PIAEAK-FYKFSYGF---RPMRS-----AEHAMAEILEK----ARK--SK---------------------
TYW---VIEGDIKGYF-DNINHNKLITML-WKI--G-IK---------DKR-VLSIIK--KMLKSGI---V--
--EE--D--
------------------------------------G---------E---IY--P----SD------------
----
-----------------------------L-GSPQGGIIS-PLLANIYLN-FFD-------------------
-----WMI-AEEFDQHHY---I------NNYER------------RDK-GL----------R-----------
AIRRD-------------------H-KPVYSIRYADDWVV--LC------SSKKQ----AD-
TLLIKIRKYLKHQLSLELSEEKTKITN----L--VE------E--------------------KASFLGFEF-
>DS_Bacillifid|18919101|locus|VBIBacCer120424_5683| [Bacillus cereus Q1]
GIDKKDV--------------------NYYLQM---------EAKQLIK---LIRQHI----DNY---
KPNPVRREYINK--GN---------------------G---K-------KRPLGIPTMIDRIIQEIARIVLE-
--PIAEAK-FFNHSYGF---RPYRS-----CHYAIGRVLNT----I----SR-------------S-------
KTYI--AIEGDIKSFF-DHINHNKLVEMM-WNMGIK------------DKR-FLIIIK--KMLRAGV---L--
---E--D--
--KVILP----TE------------

-----------------------------I-GTPQGGIIS-PLLANIYLN-NFD-------------------
------WMVAK----------------------EFEEHRARYTVKHAFRSG---LTKVGRRH-----------
--------------------------KKCFLIRYADDWII--LC------EDTVQ----AR-
ILLTKIDKYYKHILKLELSKEKTFITD----L-REK---------------------------PARFLGFDI-
>DS_Bacillifid|96574781|locus|VBIBacCer255427_4629| [Bacillus cereus
FRI35]
GVDGTTI--------------------ND-YLQ--------MDRKQLIN---LIQSQI----DNY---
NPSTVRRTYIPK---G-N------------------TG---K-------LRPLGIPVIVDRIIQEIARMAIE-
--PYCEAK-FYPHSYGF---RPYRS-----SEHAIARIVQN----IN---SK---------------------
AYI---AIEGDIKGYF-DNINHNKLLAIL-WEM--G-IK---------DKQ-FLFLIK--KMLKSKI---L--
---D--N--

------------------------------------G---------N---II--S----SD------------
----
-----------------------------K-GTPQGGIIS-PLLANVYLN-NFD-------------------
-----RMV-SDLWESHSA---V------TTYAA------------TRN-GK----------
TVEEKNYQFLRKKSVAK-------------------H-YKTNLVRYADDWII--LT------ETKEY----
AE-KLLTKLRKYMKHQLSLELSEEKTVITD----S--RE------E--------------------
PLHFLGFRI-
>DS_N.sp.I1/BA000019/6209592..6207287/Nostoc sp./CL2
GVDGKKA-----------------------LEP-----SQRLALYEVLV-------------
KNWKQWKHQPLKRVYIPK--AD---------------------G---T-------
RRGLGIPTISDRAYQCLIKYALE---PAAEAM-FNARSYGF---RPGRS-----CHDVQKLLFSN----
LNGGQAN-------------GL------SKR---ILELDIERCF-DKIDHKFLMQSV--QL------------
---PKA-AKQGIF--WAIKAGV-----------R--------------------------------------
--G--------
-----EFP-S----SE---
---S-GTPQGGVIS-
PLLANIVLH-GLENVGHEL------------------RYK-VR------------------------------
--------SG---------GR-----------------------------------QIDTIKGFRYADDVVF-
-LL------KPEDN----PE-ALRQNIDTFLE-ARGLKVKEAKTKIVH----S--TD----------------
-----------SFDFLGWNF-
>DS_cianobacteriafid|115549836|locus|VBIAnaSp49473_5321| [Anabaena sp. 90]
GVDGKAS--------------------LT-YKE----------RVELDK---LLMEQV----NTW---
THSKLREIPIPK--KD---------------------G---T-------KRILKVPTIKDRAWQCIIKYTIE-
--PAHEAI-FHERSYGF---RPGRS-----THDAQKYLFDN----LRSQSHG-------------K-------
DKI---ILEMDIEKCF-DRISHNHLMSQI--------IA---------PQS-VKLGVW--KCLKAGVNPEF--
----
---PE------------

-----------------------------Q-GTPQGGVCS-PLLANIALH-GIE-------------------
------AI-HK--
------------------------------SVRYADDMVF--IF------KKGDD----QA-
KVFDEITEFLR-IRGLNIKTAKTRFVP----A--TT---------------------------GFNFLGWKF-
>DS_cianobacteriafid|22782216|locus|VBINosSp37423_6520| [Nostoc sp. PCC 7120]
GCGESRT---PRF----
NREVRRI--IPPIDSN--QCLAKYALE--
-PAHEAT-FHEHSYGF---RPGRS-----THDAQSQIANYLASSKGGINKR----------------------
-----ILELDIEKCF-DRINHSTIMSNL--------IA---------PQG-LKQGIF--RALKAGINPEF---
----
--PE-------------

----------------------------Q-GTPQGGVVS-PLLANIALN-GIE--------------------
----DLHQYH-------------------------DCNYKKITPSTPERN-----------------------
-------------------------IKKACVRYADDMVF--FL------RPEDD----AE-EILEKISQFLA-
QRGLKISEKKTKLTA----S--TD---------------------------GFDFLGWNF-
>DS_cianobacteriafid|22782374|locus|VBINosSp37423_6599| [Nostoc sp. PCC 7120]
-MEIGSI--------------------KD-YGK-----------------------------------SPSP-
KRTGLPE--

----------F--

--PE----------------

------------------------Q-GTPQGGVVS-PILANIALN-GIE------------------------
SIHRSK--------------------------------------AK---------GQ----------------
---------------------IIEPSVRYADDMVI--IL------KPKDN----AI-EILERISEFLR-
KRGMQVSQKKTKITA----A--TD---------------------------GFDFLGWHF-
>DS_C.w.I6/NZ_AADV02000041/1584..4153/Crocosphaera watsonii/CL2
GIDGIKS--------------------LN-FKQ---------RFALAER---LLKA------HDW---
KHSKLREIPIPK--KD---------------------G---T-------TRMLKVPTMADRAWQCLVKYALE-
--PAHEAL-FHARSYGF---RPGRS-----THDAQKILFLNLKSDSNGLNKR---------------------
------ILELDIEKCF-DRINHTSIMERV--------IA---------PQT-IKTGIW--RCLKAGVNPEF--
----
---PE------------

-----------------------------Q-GTPQGGVVS-PLLANVALD-GIE-------------------
---------DI--------------------------------------HY----------------------
------------------------------SIRYADDMVV--IL------KPKDD----AD-
KILKDIQEFLA-ARGLKVSEKKTKLVR----A--TE---------------------------GFDFLGWHF-
>DS_cianobacteriafid|115603115|locus|VBIRivSp77222_2588| [Rivularia sp.
PCC 7116]
GIDGKKS--------------------LT-FEE----------RFALEE---LLKA------KSSKW-
KHQKLRAIPIPK---K-D-------------------G-T-T-------TRLLKIPTLADRCWQCLAKYALE-
--PAHEAT-FHKHSYGF---RTGRS-----AHDAQKQVFQN----L----K--------------S--
SSNGINKR---ILELDIEKCF-DRINHSSIISNL--IA---------------PNR-LKLGIF--RCLKVGI-
--N-----P--D---
---FP--------E-------

----------------------------------Q-GTCQGGVVS-PLLANIALN-GIE--------------
----------ELH-KYHTN-----------------------KGRKIKATTPE-KD----------I------
-------------------------------NT-ACVRYADDMVF--FL------RPEDD----EK-
EILDNISQFLA-KRGLKVSEKKTKLTA------------------------------STF---GFDFLGWHF-
>DS_N.sp.I2/BA000020/259212..261419/Nostoc sp./CL2
GIDGVKS--------------------LD-FNG----------RFELEI---TLKQ--SS--GNW---
HHQELREIPIPK---K-D-------------------G---T-------TRMLKIPTIADRCWQCLAKYALE-
--PAHEAT-FHARSYGF---RTGRA-----AHDAQQFLFSN--L---------------------S-
SKAKRISKR---VIELDIEKCF-DRINHSTIMENL--IA---------------PKG-IKLGIY--
RCLKAGI---------NP-E---
--F--P-----
E--
--Q-GTPQGGVVS-PLLANIALN-GIE------
------------------SIH----------------------------RYHKDNQRITNKTPE---------
SD-------------------------------------IRYPSVRYADDMVI--VL------RPQDD----
AN-EILAKIEDFLN-ARGMKVSAKKTKITA----T--TD---------------------------
GFDFLGWHI-
>DS_cianobacteriafid|115514952|locus|VBICalSp227687_3172| [Calothrix sp.
PCC 6303]
GIDGKKS--------------------LT-FRE----------RFELSE---LLKA--SC--NNW---
KHQGLREIPIPK---K-D-------------------G---T-------TRMLKIPTMADRAWQCLAKYALE-
--PAHEAT-FHARSYGF---RSGRS-----AHDAQTVLLTH--L---------------------R-
SNNNGINKR---VIELDIEKCF-DRISHTSIMENL--IA---------------PKG-VKLGIF--
RCLKAGI---------NP-E---
--F--P-----
E--
--Q-GTPQGGVVS-PLLANIALN-GIE------
------------------SIH----------------------------RYHRNGSKITNKTAG---------

KD-------------------------------------ITEPSIRYADDMVI--II------RPQDD----
AQ-KILADIDSFLA-ARGMKVSEKKTKITA----A--TD---------------------------
GFDFLGWHF-
>DS_cianobacteriafid|115337801|locus|VBIAnaCyl106394_6267| [Anabaena cylindrica PCC 7122]
GIDGKTA--------------------LT-FEQ----------RFQLSE---KLRT--EA--NNW---
KHQGLREIPIPK---K-D-------------------G---K-------TRILKVPTIADRAYQCLVKYALE-
--PAHEAT-FHARSYGF---RTGRS-----AQDAQKYLYTN--L---------------------N-
SSVNGIEKR---VIELDIEKCF-DRINHTAIMDRL--IA---------------PYS-IRLGIF--
RCLKAGV---------NP-E---
--F--P-----
E--
--Q-GTPQGGVVS-PLLANIALN-GIE------
------------------SIH----------------------------RYHIQGLRITNKTKG---------
YK-------------------------------------IVEPSVRYADDMII--IL------RPEDD----
AK-EILDKISRFLA-ERGMKVSEKKTKLTA----T--TD---------------------------
GFDFLGWHF-
>DS_cianobacteriafid|115430450|locus|VBICriEpi239080_1694| [Crinalium epipsammum PCC 9333]
GIDGKAS--------------------LN-HEE----------RFALSE---ELRT--RS--SKW---
KHQKLREIPIPK---K-D-------------------G---T-------TRLLKVPTIGDRAWQCLVKLALE-
--PAHEAT-FHAKSYGF---RTGRA-----AHDAQKYLFDH--L---------------------R-
STSHGIEKR---VIELDIEKCF-DRIAHKSIMERL--IA---------------PSG-IKLGIY--
RCLKAGV---------NP-E---
--F--P-----
E--
--Q-GTPQGGVVS-PLLANIALN-GIE------
------------------DIH----------------------------Q-----------------------
---SVRYADDMVF--IL------KPKDD----
AV-AILEQISQFLA-ERGMKISEKKTKLTA----T--TD---------------------------
GFDFLGWHF-
>DS_cianobacteriafid|115683516|locus|VBIOscNig7962_8018| [Oscillatoria nigroviridis PCC 7112]
GIDGRAS--------------------LT-FEE----------RLALSE---ELRAKS----NNW---
KHQKLRSIPIPK--KD---------------------G---S-------TRLLKIPTIADRAWQCLAKYALE-
--PAHEAT-FHARSYGF---RTGRS-----AHDAQKFLFLNLSSKAHGISKR---------------------
------VIELDIEKCF-DRISHTSIMERL--------IA---------PKG-IKTGIF--RCLKSGVNPGF--
----
---PE------------

-----------------------------Q-GTPQGGVVS-PLLANIALN-GIE-------------------
-----EIH---RS----------------------
-------------------------------VRYADDMVI--IL------KPKDD----AK-
AILDKVSEFLA-ARGMKVSEKKTKLTA----T--TD---------------------------GFDFLGWHF-
>DS_Gfid|115641574|locus|VBIThiNit264030_3543| [Thioalkalivibrio nitratireducens DSM 14787]

-------------------------MQRCFLSLAKR---------SS---------------------AEW--
-ILEGDIRACF-DAFDHDWLIEHT--PT---------------DQG----RLR--AWLKSGF---M-------
-E---
-------------------------------Q-------------RRIFP----TE-----------------
----
------------------------R-GTAQGGIIS-PTVANMVLD-GLE------------------------

GRIRAR-----------------------------------FKRRG---------------------------
----------------------KVNLIRFADDFVI--TG------ESRAI----LENDVTPLVTEFLH-
ERGLVLAPEKTRIVH----I--DD---------------------------GFDFLGFRF-
>DS_clostridiafid|161805880|locus|VBICloPas18034_1667| [Clostridium
pasteurianum BC1]
GVDKELW--------------------ST-TAS---------KMQAVL----SLTD------KNY---
KAKPLRRVYIEK---K---------------------G---K----K-AKRPLGIPCMYDRAMQALYALALD-
--PVSEVT-ADTKSFGF---RKNRC-----CQDACEYIFTA----L----SR------------------
ENCAKW---ILEGDIKACF-DYISHEWLIENI--PM---------------DKS----VLK--QFLKAGF---
V-----FENE---
---LF--P----TD---------

--------------------------------D-GTPQGGVIS-PILANMALD-GMQ----------------
--------KALSDR-------------------------FHTNKLGRVDNRFQI----------A--------
-----------------------------NKVYLVRYADDFIV--TA------ATKEI----AE-
EAKELIREFLQ-TRGLELSEEKTKITH----I--ND---------------------------GFDMLGWTF-
>DS_Bacillifid|19653441|locus|VBIStrEqu35012_1915| [Streptococcus equi
subsp. zooepidemicus]
GIDGELW--------------------TT-PAQ---------KMEALL----SLTD------KGY---
KASPLRRVYIDK---K---------------------G---K----K-KKRPLGIPTMYDRAMQALYALALE-
--PIAETT-ADTKSFGF---RKGRS-----CQDACEYIFTA----L----SR------------------
KASPQW---ILKGDIKGCF-DNISHDWLLENI--PM---------------DKS----ILK--QFLKAGF---
V-----FKGE---
---LF--P----TE---------

--------------------------------D-GTPQGGIIS-SILANMALD-GLQ----------------
--------QVLSDR-------------------------FHTNRLGRIDFRFKN----------S--------
-----------------------------HKVNLVRYADDFIV--TA------ATQEI----AL-
EAKELIREFLI-GRGLELSEEKTLVTH----I--ND---------------------------GFDLLGWNF-
>DS_Ms.b.I1/NZ_AAAR02000002/377828..379992/Methanosarcina barkeri/CL1
GIDGEKW--------------------LS-SAS---------KMKAVL----SLTG------KRY---
KAKPLKRVFINK---P---------------------G---K----TK-KRPLGIPTMYDRAIQSLYSLALE-
--PVAEIK-SDLRSFGF---RKHRS-----TKDACQQIFLC----L----SK------------------
KTSAQW---ILEGDIRGCF-DNINHQWLLTNI--PI---------------DKA----ILT--QFLKAGF---
I-----YKRH---
---LN-------PT---------

--------------------------------KAGTPQGGIIS-PILANMTLD-GIE----------------
--------KMLLVK---------------------------------YPKKGKN-------------------
----------------------------SKKVNFIRYADDFIV--TA------NSKET----AG-
EIKDEVVAFLK-ERGLELSDDKTFITN----I--NE---------------------------GFDFLGWNF-
>DS_clostridiafid|161806116|locus|VBICloPas18034_1785| [Clostridium
pasteurianum BC1]
GVDKKLW--------------------ST-SAS---------KIKAVL----TLTD------KQY---
RTKPLKRVYIKK-KGK---------------------N---K-------KRPLGIPTMYDRAMQTLYALALE-
--PVAEVT-GDHISFDF---RKGRS-----AKDACEQTFCV----L----SR------------------
KCSPTW---ILEGDIKGCF-DNINHDWLQKNI--PM---------------DKR----IMK--QFLKSGF---
I-----Y--E---
---------------------------------------G-------------NLF-P----TD---------
----
--------------------------------T-GSPQGGAIS-SLYANMTLD-GLE----------------
--------KLIQDK-------------------------YHRNSKGKIENHYRA----------K--------
-----------------------------TKVNMVRYADDFII--TA------NTKEI----AE-
ELKDIVSKFLK-NRGLNLSQEKTTITH----I--DY---------------------------GFDFLGWTF-

>DS_fid|189809342|locus|VBIAltMac287461_0611| [Alteromonas macleodii str.
'Ionian Sea U4']
GIDGETW--------------------QS-ATK---------KWRAIS----SLKR------SGY---
KASPLRRVFIPK--SN---------------------G---Q-------RRPLGIPTMLDRGMQALYLLAVE-
--PEVETN-SDGNSFGF---RKQRS-----CADAIEQCFKV----L----CR---------------------
KGAGECVLDADIKGCF-DNISHEWMLKHL--SI---------------DKP-ILS-----QWLNAGF---M--
---E--S--
------------------------------------G-------------KVY-P----TL------------
----
-----------------------------A-GTPQGGIIS-PTLMNMVLN-QLQ-------------------
-----GTI-EE--------------------------ASGVKRGKHREIRS---------NV-----------
--------------------------KRVSVIRYADDFVV--TA------HSQAF----
LVDTILPCINEFMS-QRGLALSPEKTHVRH----I--SE---------------------------
GFDFLGQNL-
>DS_fid|35297935|locus|VBIXenBov95754_1334| [Xenorhabdus bovienii SS2004]

--MIDRTQQALHLLALD---
PISETI-ADPNSYGF---RPNRS-----TADAIAQCFK-----CLCQKRS---------------------
ARW---VLEEDLKACF-DKIGYQWLIENI--QI---------------DKR----MLK--QWLGSDF---I--
---D--K--
------------------------------------G-------------LFYRT----AE------------
----
-------------------------------GTPQGGIIS-PTLMLLTLA-GLE-------------------
-----KRVKEV--------------------------------------AR--------KTD-----------
--------------------------DRINSIEYADNFVM--TG------ASEDV----
LLNEVKPQLIDFLR-ERGLTLSEEKTHITH----I--ND---------------------------
GFDFLGFNL-
>DS_fid|54183625|locus|VBISheBal163160_2541| [Shewanella baltica OS117]
GIDGIIW-----------------------------NSDA--RCMTAVN---QLSR------KGY---
HAKPLRRIYIPK--KN---------------------G---K-------LRPLGIPCMIDRAQQALHLLALE-
--PISETV-ADLNSYGF---RPNRS-----AADAIAQCFK-----CLCMKRS---------------------
SQW---VLEGDIKACF-DKIGHQWLIDNI--QL---------------DKR----MLK--QWLGCGY---V--
---D--K--
------------------------------------G-------------LFY-K----TA------------
----
-----------------------------E-GTPQGGIIP-PTLMLLTLA-GLE-------------------
-----QLV-KS----------------------------------IACKTG----------------------
--------------------------NSVNFIGYADDFII--TG------SSKEV----
LVNEIKPQLIGFLQ-ERGLTLSDDKTHITH----I--DD---------------------------
GFDFLGFNI-
>DS_fid|58933841|locus|VBISheBal147952_0958| [Shewanella baltica OS678]
GIDGIIW-----------------------------NTDA--RRMKAVN---QLSR------KAY---
IAKPLKRIYIPK---K-N-------------------G---K-------LRPLGIPCMIDRAQQALHLLALE-
--PVSETL-ADPNSYGF---RPNRS-----TADAVDQCFKC--LAQ----KK-------------S-------
AQW---VLEGDIKACF-DKIGHQWLLDNI--TV---------------DKR----MLE--QWLKSGF---M--
---D--K--
------------------------------------G-------------LFY-R----TD------------
----
-----------------------------E-GTPQGGVIS-PSLMLMTLA-GLE-------------------
-----QHI-KS----------------------------------TALKKG----------------------
--------------------------TRANFIGYADDFVV--TC------ASKEV----
LENDIKPLITDFLA-ERGLTLSEEKTHITH----I--ND---------------------------
GFDFLGFNH-
>DS_Sh.sp.I1/CP000446/2526748..2528903/Shewanella sp./CL1

```
GIDGVIW-----------------------------NTDA--RRIAAVK---QLKR------KAY---
QAKPLKRIYIPK---K-N-------------------G---K-------LRPLGIPCMIDRAQQALHLLALE-
--PISETV-ADPNSYGF---RPHRS-----TADAIAQCFLC--LSQ----RY-------------S-------
SEW---VLEGDIKACF-DKIGHQWLIDNI--AL---------------DKK----MLR--QWLECGF---M--
---D--K------------------------------------------------------------------
------------------------------------G-------------LFY-R----TD------------
-------------------------------------------------------------------------
-----------------------------E-GTPQGGIIS-PTLMLLTLS-GLE-------------------
-----QLL-KA----------------------------------TARRKG----------------------
--------------------------CNVNFIGYADDFVV--TG------SSKEV----
LVNEIKPLIARFLA-ERGLTLSEEKTHVTH----I--ND---------------------------
GFDFLGFNL-
>DS_Bacteroidetesfid|115626437|locus|VBIFibAes90597_0767| [Fibrella
aestuarina]
GVDGQLW-----------------------TNP--------PRKRQAID---ELRS------RGY---
RPQPLKRIYIPK--RN---------------------G---K-------QRPLSIPTMKDRAMQALHLMALQ-
--PVSETT-ADPCSFGF---RPARQ-----VADAVERCFGL----L----SR------------------
QDSPQW---VLEADIEACF-DRIDHDWLLQHI--PM---------------EKT----ILG--QWLKAGY---
I-----EKGN---------------------------------------------------------------
-----------------------------------------------------WW--P----TT---------
-------------------------------------------------------------------------
--------------------------------E-GTPQGGIIS-PVLANMALD-GLA----------------
--------KELAAH-----------------------------FAKSYKRPDRG---------FN--------
-----------------------------PKVRLVRYADDFII--TG------ISRQQ----
LEEQVKPVVCNFLS-KRGLRLSESKTRQTA----I--TE---------------------------
GFDFLGFTF-
>DS_fid|24029979|locus|VBIWolEnd21207_0693| [Wolbachia endosymbiont of
Drosophila melanogaster]
GVDRQIW--------------------ST-CNT----------KFQGIK---LLKQ------RGY---
KPSPLKRIYISK---S-N-------------------G---K-------RRPLGIPTIKDRAMQALYLFALE-
--PIAETI-SDRHSYGF---RPKRS-----CADATVACHLL----L----AS-------------R-----
NQLQW---ILKGDIKWCF-DNINHEWLMKHI--------------------PM-EKKILH--SWLKAGF---
L-----E--S---------------------------------------------------------------
---------------------------------------K---------T---LY--S----TT---------
-------------------------------------------------------------------------
--------------------------------A-GTPQGSIIS-PILANLALN-GLE----------------
--------KSL-ESQFG-----------------------KLGSKRRSKIR-SG----------V--------
-----------------------------N---VIRYADDFII--SG------ITREV----
LENEVKPLVSSFLQ-ERGLILSEEKTKITS------------------------------ITT---
GFDFLGCNV-
>DS_fid|184905921|locus|VBILegPne304526_2043| [Legionella pneumophila
str. 121004]
GIDGVVW--------------------TT-SEE---------KCEAVRN----LKA------RGY---
KATPLRRIYIPK--KN---------------------G---K-------ERPLSIPTLKDRAMQALYLLALE-
--PVGETT-ADLNSYGF---RPKRS-----THDAIYQCYAT----L----AR------------------
KNCAQW---ILEGDIKACF-DEIDHGWLKSNI--------II---------DQR-VLT-----QWLQAGY---
M-----EKNQ---------------------------------------------------------------
-----------------------------------------------------LF--E----TA---------
-------------------------------------------------------------------------
--------------------------------R-GTPQGGPAS-PLLANMVLD-GLE----------------
--------REI-HS-----------------------------------GCGQG-------------------
-----------------------------NKINYIRFADDFIV--TA------NSPDI----
LKEKVMPIISNFLA-QRGLSLSQEKTKIVH----I--EE---------------------------
GFDFLGFNV-
```

>DS_fid|54574176|locus|VBIGlaSp182133_0425| [Glaciecola sp. 4H37+YE5]
GIDGIVW--------------------IN-TKQ---------KWEAAK----ALSC------RNY---
RSQPLRRVYIPK--KN---------------------G---K-------KRPLGIPTMFDRAMQALFLLAYE-
--PVAEVT-ADHHSYGF---RPKRS-----AADAIEKCFNV----L----AQ------------------
KTSAQW---ILEGDIKGCF-DNISHTWLHQHL--KL---------------EQK----VLN--QWLKAGF---
M-----D--K---
---------------------------------------G-------------RLFPT----TA---------
----
----------------------------------GTPQGGIIS-PCLSNGALD-GME----------------
--------AML-KS-----------------------------------ITKPI-------------------
-----------------------------QKVHLIRYADDFVI--TA------NSKEL----
LENTIKPAEMAFLF-ERGLTLSKEKTLITS----I--TK---------------------------
GFDFLGFNV-
>DS_Bacillifid|42436540|locus|VBIStrPne98725_0947| [Streptococcus pneumoniae AP200]
GVDGELW-----------------------LTP--------QAKYKAIE---KLNL------RGY---
KPKPLKRVYIPK--KN---------------------G---K-------KRPLSIPTMTDRAMQTLYKFALE-
--PIAETT-ADPNSYGF---RAKRC-----TQDAIEQCFTS----L----NK------------------
KKSAKW---VLEGDIKGCF-DNISHEWILNNI--PM---------------NKK----LLK--LWLECGY---
I-----EKQK---
---LF-------PT---------

--------------------------------ETGSPQGSPIS-PIISNMVLD-GLE----------------
--------KAIKEK------------------------------YHRRTVNKKT---------YF--------
-----------------------------PKVNFARYADDFIV--TG------ESAEL----
LENGVKPIIVKFLA-ERGLELSEEKTLITH----I--ND---------------------------
GFDFLGVNI-
>DS_Bacillifid|54795537|locus|VBIStrPas183593_1131| [Streptococcus pasteurianus ATCC 43144]
GVDKELW-----------------------LTP--------NAKYQAIK---KLKV------RGY---
CPKPLRRIYIPK--KN---------------------G---K-------KRPLSIPTMTDRAMQTLFKFALE-
--PIAETT-ADPNSYGF---RPKRS-----TQDAIEQCFLA----L----SK------------------
QKSAKW---VLEGDIKGCF-DNISHEWIMKNI--PM---------------NKT----ILG--KWLKSGY---
I-----ENQK---
---LF-------PT---------

--------------------------------ELGSPQGSPIS-PIISNMVLD-GLE----------------
--------RKLSAT--------------------------------FRKKKVNG-------KVYT--------
-----------------------------PKINFVRYADDFIV--TG------VSKEL----
LENEVKPVIIEFLK-ERGLELSEEKTLITH----I--TD---------------------------
GFDFLGINI-
>DS_fid|48578553|locus|VBIEscCol159162_5238| [Escherichia coli UMNK88]
GVDGEIW--------------------QH-PES---------KWSAIT----RLKR------SGY---
HPLPLRRIYIPK--AN---------------------G---K-------FRALGIPTMLDRAMQALYLMALE-
--PLSEIT-ADHHSYGF---RPMRS-----TADAIEQVFNA----C----GK------------------
KASAEW---ILEGDIRGCF-DNLSHEWLVSHI--PM---------------DRM----VLR--NWLKSGY---
C--------E---
---------------------------------------G-------------MSFYP----TK---------
----
--------------------------------G-GTPQGGIIS-PTLMNMALD-GLQ----------------
--------SLL-ER-------------------------------RFPSTTVQG---------RK--------
-----------------------------AKIHLVRYADDFVI------------------------------
-----TGATAELLRNDVMP----I-------------------------------VIDFLGDAA-

```
>DS_fid|45180960|locus|VBIBurRhi170666_0329| [Burkholderia rhizoxinica
HKI 454]
GVDGRIW--------------------ATPMSK-----------LKAAQ---SLTH------RGY---
QALPLRRVYIPK--SN---------------------G---K-------ERALGIPTMRDRAMQALWLTALL-
--PIAETT-ADPNSYGF---RPKRS-----TADAVEQCFKA----L----AK------------------
RNSAQW---VLEGDIRGCF-DNFSHDWLLANI--PM-------------------NKAVLR--KWLQAGF---
V-----D--K---------------------------------------------------------------
---------------------------------------G-------------VLF-P----TD---------
-------------------------------------------------------------------------
--------------------------------A-GTPQGAIAS-PVLANMALD-GLE----------------
--------EAV-RS--------------------------VLGPSKTARQPAKA----------H--------
---------------------------------VVRYADDFIA--TG------ASREL----
LEKQVKPAIEAFLS-ARGLQLASEKTLVTH----I--AR---------------------------
GFDLLGQNV-
>DS_fid|19961745|locus|VBIPseStu31643_0668| [Pseudomonas stutzeri A1501]
GVDGKIW--------------------ST-------PVAKSTGAQALQH-------------RGY---
RPQPLRRIYIPK--SN---------------------G---K-------KRPLGIPTMRDRAMQALWKLALE-
--PVAETR-ADPNSYGF---RPQRS-----TADAIAHCFNA----L----AK------------------
RGSAHW---VLEADIRGCF-DNISHDWLLTNV--PM---------------DKV----VLR--KWLRAGY---
V-----D--Q---------------------------------------------------------------
---------------------------------------G-------------ALF-A----TE---------
-------------------------------------------------------------------------
--------------------------------A-GTPQGGIIS-PVLANWTLD-GLE----------------
--------DVVHAS--------------------------------VASTARKR---------KP--------
-----------------------------FKIHVVRYADDFII--TG------ATKAV----
LQHQVRPAIEAFLK-ERGLELSDEKTQITH----I--SQ---------------------------
GFDFLGQNV-
>DS_P.p.I2/Y18999/752..2957/Pseudomonas putida/CL1
GVDGKIW--------------------AT-------PAAKSSGMESMRH-------------RSY---
RALPLRRIYIPK--SN---------------------G---Q-------KRPLGIPRMLCRSMQALWKLALE-
--PVSESL-ADPNSYGF---RPNRS-----TADAIEYCFIT----L----AK------------------
RTSPVW---VLEGDIRGCF-DNFNHEWMLKNI--PM---------------DKT----ILR--RWLQAGF---
I-----D--E---------------------------------------------------------------
---------------------------------------G-------------TLF-A----TQ---------
-------------------------------------------------------------------------
--------------------------------A-GTPQGGIIS-PVIANMALD-GLE----------------
--------AAVHAS-------------------------------VGPTKRARE----------R--------
-----------------------------SKINVVRYADDFVV--TG------ISKEI----
LEHSVLPAVRQFMA-IRGLELSEEKTKITH----I--AE---------------------------
GFDFLGQNV-
>DS_fid|21165521|locus|VBIBorPet31633_2025| [Bordetella petrii DSM 12804]
GVDRVLW--------------------DS-PES---------KWEAIG----RLRQ------PGY---
RPLPLRRVYIPK--SN---------------------G---K-------ERPLGIPTMRDRAMQALYLLALE-
--PVSEST-SDPNSYGF---RKGRS-----TADAMAQIFVT----L----SG------------------
RASAQW---ILEADIKGCF-DWINHEWLLANV--PM---------------DRR----VLR--KWLKAGV---
I-----HKGQ---------------------------------------------------------------
-----------------------------------------------------LQ-------PT---------
-------------------------------------------------------------------------
--------------------------------TAGTPQGGIIS-PTLANVTLN-KLE----------------
--------TDLAEY------------------------------LGTKLGWTKA---------KR--------
-----------------------------LKVHVVRYADDFIV--TG------ASKDV----
LDTEVRPWIERFLA-VRGLQLSTEKTRIIH----I--DE---------------------------
GFDFLGWNF-
```

>DS_fid|19847262|locus|VBIPseAer79785_0614| [Pseudomonas aeruginosa
UCBPPPA14]

---MQALYLLALS---
PIAETT-GDPNSYGF---RIERS-----TADAMSQLFVC----L----SG------------------
KASAQW---ILEADIQGCF-DHINHDWLLNHV--PT---------------DKV----ILR--KWLKAGV---
I-----H--K---
---------------------------------------G-------------QLQ-A----TD---------
----
--------------------------------A-GTPQGGIIS-PTLANMVLD-GLE----------------
--------SQL-KR---------------------------------HLGVTRA---------KK--------
-----------------------------LKLNVVRYADDFVI--TG------VSPEV----
LEKEVKPWVEQFLA-VRGLQLSLEKTRIAH----I--DQ---------------------------
GFDFLGWNF-
>DS_fid|42537310|locus|VBIRalSol167236_2271| [Ralstonia solanacearum
PSI07]

--MLDRAMQALYLLALE---
PVSEGT-SDPNSYGF---RINRS-----TADAMSQLFVS----L----SQ------------------
KASAQW---VLEADIKGCF-DHISHDWLECNV--HM---------------DKA----ILR--KWLKAGV---
V-----F--Q---
---------------------------------------G-------------QFQ-A----TE---------
----
--------------------------------A-GTPQGGIIS-PTLANVALN-GLE----------------
--------NQLFAH------------------------------LRAKLGAVKT---------KK--------
-----------------------------LKVNVVRYADDFVI--TG------STPEL----
LEDEIKPWVERFLA-VRGLSLSTEKTRIVN----I--TE---------------------------
GFDFLGWNF-
>DS_fid|61234146|locus|VBILegPne178567_1092| [Legionella pneumophila
subsp. pneumophila ATCC 43290]
GVDGKIW--------------------ST-PEA----------KSKAIT---QLKR------RGY---
KPYPLKRVYIPK---S-N-------------------N---T-------KRPLGIPVMRDRAMQALYLLALE-
--PVSETT-ADWNSYGF---RPRRS-----THDAISHLFVM----L----AR-------------K-----
GAAQW---VLEGDIKGCF-DTISHEWILNNV--------------------ML-DKRMLQ--HWLKAGY---
I-----D--K---
---------------------------------------G---------H---LF--P----TQ---------
----
--------------------------------E-GTPQGGIIS-PTLANLVLD-GLE----------------
--------TLL-ATKFG-------------------SLKHDGHASRTSKYQ-------------V--------
-----------------------------H---FVRYADDFVI--TG------KSKTL----
LEDEVKPLIKDFLA-QRGLKLSEQKTKVTH------------------------------ITH---
GFDFLGQNI-
>DS_fid|115293380|locus|VBIRhiTro150571_4588| [Rhizobium tropici CIAT
899]
GVDRIIW--------------------DT-PDK---------CVRGLL----SLKR------RGY---
HPLPLRRVYIPK-ANS---------------------K---K-------LRPLGIPTMKDRAMQALHLLALL-
--PVAETT-ADPNSYGF---RPYRA-----TRDAARQCFIA----L----RG------------------
RGTAEW---VLDADIAGCF-DEISKDWLIANI--PM---------------DKV-VLR-----KWLDSGY---
I-----K--D---
---------------------------------------G---------D---WH-------AT---------
----
--------------------------------KAGTPQGGIIS-PTLANMALD-GME----------------
--------KML-RD--------------------------------FYGPRRRN---------SL--------

-----------------------------TKVHLIRYADDFVV--TG------ASKEV----LE-
EAKSMVEEFLS-ERGLSLSEEKTRIVR----V--EE---------------------------GFDFLGWNV-
>DS_Th.e.I1/BA000039/27344..30566/Thermosynechococcus elongatus/CL1
GVDGITW--------------------ST--QE---------QKTQAIK---SLRR------RGY---
KPQPLRRVYIPK--AN---------------------G---K-------QRPLGIPTMKDRAMQALYALALE-
--PVAETT-ADRNSYGF---RRGRC-----TADAAGQCFLA----L----AR---------------------
AKSAEHVLDADISGCF-DNISHEWLLANT--PL---------------DKG----ILR--KWLKSGF---V--
----
------------------------------------W---------K-QQLF--P----TH------------
----
-----------------------------A-GTPQGGVIS-PVLANITLD-GME-------------------
-----ELL-AK------------------------------------HLRG----------------------
--------------------------QKVNLIRYADDFVV--TG------KDEET----LE-
KARNLIQEFLK-ERGLTLSPEKTKIVH----I--EE---------------------------GFDFLGWNI-
>DS_Bfid|42338528|locus|VBIGalCap53152_0971| [Gallionella
capsiferriformans ES2]
GVDKVVW--------------------DT-PEL---------KAEAVM----SLKR------KGY---
QPQPLRRVFIPK--AN---------------------G---K-------MRPLGIPTMKDRAMQALYLQALE-
--PVSETK-ADPNSYGF---RPMRA-----SRDAAAQCFNS----L----AQ------------------
KYAAKW---VLDADISGCF-DNINHDWLLNNI--PM---------------DKV----TLQ--KWLKSGF---
-------KWN---
---------------------------------------G-------------QLFNT----EA---------
----
----------------------------------GTPQGGIIS-PTLANMTLD-GMA----------------
--------EMLQKR--------------------------------------FGATGSREAAKYK--------
-------------------------------VNLIRYADDLVI--TG------TTKEV----LE-
EVRELMAEFLK-VRGLTLSEEKTKIVH----I--EE---------------------------GFDFLGWNV-
>DS_Bacteroidetesfid|21069184|locus|VBIBacVul85104_2201| [Bacteroides
vulgatus ATCC 8482]
GVDKITW--------------------SS-------PLAKAKAIFTLKR-------------HGY---
KPQPLKRVNIKK--KN---------------------G---K-------LRPLGIPTMKDRAMQALYLMALD-
--PIAETT-GDSHSYGF---RRHRC-----THDAIEQCYIV----L----SR------------------
SVAPEW---ILEGDIKGCF-DHISHAWLINNI--PM---------------DKE----ILR--KWLECGY---
V-----F--N---
---------------------------------------G---------E---LF--P----TE---------
----
--------------------------------E-GTPQGGIIS-PTLANMALD-GLQ----------------
--------DLL-EK-----------------------------SVKKYQVNYKK---------IV--------
-----------------------------PKIHLVRYADDFIV--TA------KDKET----
IEQVILPLVRKFLA-ERGLTLSEEKTKITH----I--NE---------------------------
GFDFLGFNI-
>DS_B.t.I1/AE015928/2871095..2873499/Bacteroides thetaiotaomicron/CL1
GVDKVKW--------------------ST-PNA----------RFKAIG---ELKR------RGY---
KPQPLKRVNIKK--SN---------------------G---K-------LRPLGIPTMKDRAMQALYLLALE-
--PVSETT-ADSNSYGF---RKERS-----TGDAREQCFCV----L----AK------------------
KASPEW---IMEGDIQGCF-DHISHEWLLNNI--PM---------------DKV----MLR--KWLKCGF---
V-----FNKE---
---LF--P----TE---------

--------------------------------E-GTPQGGIIS-PTLANMTLD-GLQ----------------
--------TMLAEK----------------------------YHKKFVTRKTTT---------YY--------
-----------------------------PKVHLVRYADDFII--TG------RNKEA----LE-
EIKPLVVDFLK-ERGLTLSEEKTKITH----I--DD---------------------------GFDFLGYNI-

```
>DS_Bacteroidetesfid|87116550|locus|VBIAliFin145170_0642| [Alistipes
finegoldii DSM 17242]
GVDMVTW--------------------KT-PDA---------KVCAITE----LKR------RGY---
TPQPLRRVHIRK--SN---------------------G---K-------LRPLGIPTMKDRAMQALYLMALA-
--PVAETT-ADANSYGF---RKERS-----TADAVQQCFND----L----AR------------------
TTSPQW---ILEGDIKGCF-DHISHEWLLDNI--PM---------------DKV----LLR--KWLKSGF---
I-----FNKQ---------------------------------------------------------------
-----------------------------------------------------LF-------PT---------
-------------------------------------------------------------------------
--------------------------------EEGTPQGGIIS-PTLANMTLD-GLE----------------
--------KLLADS-------------------------------FPINRSKKN--------YYT--------
-----------------------------PMINLVRYADDFII--TG------ESKEL----
LENHVKPLVIEFLQ-ARGLTLSEEKTKITH----I--EE---------------------------
GFDFLGFNI-
>DS_fid|22088426|locus|VBIHahChe29232_3586| [Hahella chejuensis KCTC
2396]
GVDGVLW--------------------ST-PEA---------KWAAIG----QLKR------RGY---
RALPLRRVRIPK--AN---------------------G---K-------ERLLGIPTMQDRAMQALYLLALQ-
--PVSETR-ADRDSYGF---RPDRS-----TADAIMQCYML----L----RK------------------
KGSAQW---VLEADIKGCF-DHIDHQWLIDNV--PM---------------DKL----MLR--KWLKAGV---
V--------D---------------------------------------------------------------
--------------------------------------MG-------------RVWKT----EE---------
-------------------------------------------------------------------------
----------------------------------GTPQGGIIS-PTLANMALD-GIE----------------
--------ALLAQH-------------------------FGAKGSKKLRQYKVG-------------------
---------------------------------LVRYADDFVI--TG------SSKEL----
LENEVKPLIEKFLA-VRGLKLSVEKTQVTH----I--NH---------------------------
GFDFLGWTV-
>DS_Chlorobifid|21384938|locus|VBIChlPha121022_1466| [Chlorobium
phaeobacteroides BS1]
GVDNQIW-----------------------ITP--------KAKTNAVA---SLKR------RGY---
KPLPLRRINIPK--KN---------------------G---K-------TRPLGIPTMKDRAMQALYLLALE-
--PVAETT-ADDNSYGF---RPWRS-----TADASARCFTC----L----AQ------------------
RNSAQW---VLEADIASCF-DAISHEWLIDNI--PV---------------DTA----ILR--LWLKAGF---
V-----LKNE---------------------------------------------------------------
-----------------------------------------------------LF-------PT---------
-------------------------------------------------------------------------
--------------------------------EAGTPQGGIIS-PVLANMCLD-GLE----------------
--------KALAKA--------------------------------FPQAKKRG-------------------
-----------------------------LKMHMVRYADDFVI--TG------NSKEL----
LENEVLPVVVEFLA-ERGLFLSPEKTKITH----I--TE---------------------------
GFDFLGWNV-
>DS_fid|48579126|locus|VBIEscCol159162_5518| [Escherichia coli UMNK88]
GVDGKTW--------------------SK-PGS---------KMKAIY----TLKR------RGY---
KPLPLRRIYIPK--SN---------------------G---K-------KRPLGIPTMKDRAMQALYLMALE-
--PVAETT-ADPNSFGF---RPCRS-----TADAIEQCFTT----L----HR------------------
ADRAQW---ILEADIRSCF-DEISHEWLIANI--PT---------------DTA----ILK--RWLKAGY---
I-----DLGK---------------------------------------------------------------
-----------------------------------------------------LY-------PT---------
-------------------------------------------------------------------------
--------------------------------SAGTPQGGIIS-PTLANMVLD-GLQ----------------
--------PLL-KK-----------------------------------TFYRG--------GLN--------
----------------------------PEKINIIRYADDFVI--TG------ISHDT----
```

```
LSEKVLPLLENFLA-ERGLTLSPEKTRITH----I--SD---------------------------
GFDFLGMNI-
>DS_fid|42346603|locus|VBIGamPro61291_1949| [gamma proteobacterium HdN1]
GVDKVVW--------------------DT-PEK---------KLCAMGD----LKR------RGY---
RPKPLKRVHIPK--AN---------------------G---K-------LRPLGIPTMKDRAMQALYLLGLL-
--PVSETT-ADGCSYGF---RPERS-----VADAIERCFNA----L----GR------------------
RDAAAW---VLEADIKGCF-DHISHDWLLGNV--PM---------------DKR-VLA-----TWLKCGF---
M--------E---------------------------------------------------------------
-------------------------------------------------K-AVWF--A----TE---------
-------------------------------------------------------------------------
--------------------------------A-GTPQGGIIS-PTLANFALD-GLE----------------
--------QLL-SK-----------------------------TFYRTMRHGKM---------VH--------
-----------------------------PKVHLIRYADDFVI--TG------SSEEL----
LVNEVKPLVERFLA-ERGLMLSAEKTKVTH----I--DE---------------------------
GFDFLGQNV-
>DS_fid|21163595|locus|VBIBorPet31633_1067| [Bordetella petrii DSM 12804]
GVDRVTW--------------------ST-PET---------KSEAVL----SLRR------HGY---
RPRPLRRIYIPK--AN---------------------G---K-------KRPLGIPTMRDRAMQALYLLALE-
--PIAETT-GDKDSYGF---RPGRS-----VADAIRQCHTV----LA---WK-------------------
RSAEW---VLEADIEGCF-DNISHDWLAENI--PM---------------DKA----ILK--SWLKAGY---
V-----ESGS---------------------------------------------------------------
-----------------------------------------------------LF-------PT---------
-------------------------------------------------------------------------
--------------------------------EAGTPQGGIIS-PVLANMALD-GLQ----------------
--------EVLGKS------------------------------FFRTRRQNKH---------YD--------
-----------------------------PKVNFVRYADDFIV--TG------YSREL----
LEIEVLPLVEKFLA-ARGLNISKAKTRVTH----I--SE---------------------------
GFDFLGKNI-
>DS_A.v.I1/AY057439/1648..4444/Azotobacter vinelandii/CL1
GVDGETW--------------------ST-PES---------KWKAIF----RLQR------TGY---
RPRPLRRVYIPK--AN---------------------G---Q-------RRPLGIPTMLDRAMQALYLLALE-
--PVSETT-ADRNSYGF---RPHRS-----TADAIEQLFVN----L----GR------------------
KHSAQW---VMEGDIKGCF-DNISHDWLIANV--PL---------------DKA----VLR--KWLKAGY---
L--------E---------------------------------------------------------------
---------------------------------------S-------------GQLNP----TG---------
-------------------------------------------------------------------------
--------------------------------A-GTPQGGIIS-PVLANLALD-GLE----------------
--------KALESR--------------------------------FGQRNTKA--------SYK--------
-----------------------------TKVNYVRYADDFVI--TG------ISKEL----
LVNEVKPVVAAFMA-ERGLSLAAEKSLFTH----V--SE---------------------------
GFDFLGQNV-
>DS_fid|20838927|locus|VBIAlcBor124741_0664| [Alcanivorax borkumensis
SK2]
GVDGECW--------------------DN-PAS---------KWEAIH----RLKR------HGY---
KPRPLRRVWIPK--AN---------------------G---K-------RRPLGIPTMHDRAMQALYLLALE-
--PVSETT-ADRNSYGF---RPMRA-----TADAIEQCFVV----L----GR------------------
KSSAQW---VLEADIQGCF-DNISHDWLLSHV--PM---------------DKA----VLG--KWLKAGF---
M-----E--S---------------------------------------------------------------
---------------------------------------G-------------RTH-P----TH---------
-------------------------------------------------------------------------
--------------------------------A-GTPQGGIIS-PVLANMALD-GLE----------------
--------EVL-EA------------------------------AFGQRNTKAS---------YR--------
-----------------------------TKVNYVRYADDFVV--SG------ISREL----
```

```
LEREVRPIVEAFMA-ERGLALSAEKTVVTH----V--EE---------------------------
GFDFLGQNI-
>DS_fid|32030132|locus|VBICitRod33214_3055| [Citrobacter rodentium
ICC168]
GVDGKLW--------------------ST-PKT---------KWEAIFD----MKR------TGY---
HPKPLRRVYIPK--SN---------------------G---K-------LRPLGIPTMRDRAMQALYLLALE-
--PVSETT-ADRNSYGF---RPMRS-----TADAIEQCFVA----L----SR-------------GNS-----
AQW---VLEGDIKGCF-DNISHDWLLAHI--PM---------------DKQ----VLG--KWLKAGY---M--
---K--S------------------------------------------------------------------
------------------------------------G-------------HYH-A----TG------------
-------------------------------------------------------------------------
-----------------------------A-GTPQGGIIS-PVLANMALD-GLE-------------------
-----AVLESR-------------------------------FGVKNTKAS---------YK-----------
--------------------------TKVNYVRYADDFII--TG------ISQEL----
LENEVKPLVEAFMA-ERGLQLSPEKTVITH----I--EQ---------------------------
GFDFLGQNV-
>DS_E.c.I5/AF074613/58241..60646/Escherichia coli/CL1
GVDGQTW--------------------SS-PEV---------KFLAIN----LLKR------RGY---
KPQPLKRVYIPK--SN---------------------G---K-------SRPLGIPTMKDRAMQALYLLALE-
--PVAEVT-ADQRSFGF---RTGRS-----TADAIAQCFCV----L----AQ------------------
KTSAEW---VLEGDIRGCF-DNISHQWLIDNT--ST---------------DRQ----ILT--KWLKAGY---
R-----E--K---------------------------------------------------------------
---------------------------------------G-------------QLF-P----VN---------
-------------------------------------------------------------------------
--------------------------------S-GTPQGGIIS-PVLANIALD-GLE----------------
--------ALLASE-------------------------------F-KKRTVKG---------RL--------
---------------------------VNPKVNYVRYADDFII--TG------ESKEL----
LESQVLPVVRRFMA-ERGLMLSPEKTKITH----I--EE---------------------------
GFDFLGQNI-
>DS_fid|86738873|locus|VBIPseAer240047_2455| [Pseudomonas aeruginosa DK2]
GVDGITW--------------------ST-PEA---------KSQAML----SIKR------RGY---
RPQPLKRVYIPK--TN---------------------G---K-------MRPLGIPTMKDRAMQALYLLALE-
--PVAETT-ADGRSFGF---RPERS-----TADAIEQCFTT----L----SK------------------
KVAPQW---ILEGDIKGCF-DNISHDWLMGHV--PT---------------DRE----ILR--KWLKAGY---
M-----E--D---------------------------------------------------------------
---------------------------------------R-------------QLF-P----TE---------
-------------------------------------------------------------------------
--------------------------------A-GTPQGGIIS-PTLANLVLD-GLE----------------
--------AKLDAA-------------------------------FGRKRYANG---------VQ--------
---------------------------TRLMVNYVRYADDFIV--TG------RSKEL----
LEQEVMPIIKDFMQ-ERGLTLSPEKTKITH----I--DD---------------------------
GFDFLGQNV-
>DS_cianobacteriafid|42721577|locus|VBIArtPla153080_2008| [Arthrospira
platensis NIES39]
GVDGVKR-----------------------LRN--------QEKLDLAN---CLKL------GR----
KTQGLRRVSIPE---P---------------------G---R-----DEKRAVGILMMMEKAKQGLVKLALE-
--PEWEAR-FDRNSYGF---RPRRS-----AQDAIAAIFNG----M----KE-------------D-------
HKY---VLDAHIEKCF-EGIYHQKLLAKL--NT---------------YPT-LRREIK--AWLKSGV---M--
------D------------------------------------------------------------------
------------------------------------G-------------KELFP----TE------------
-------------------------------------------------------------------------
-----------------------------T-DTPQGGLM--PLLANIALD-GLE-------------------
-----SLLEDK-----------------------------FQGGVANCGNG----------------------
```

---------------------------KATVVRYADDLVV--LD------GELEV----IL-
AAKETIEAWLM-EMGLKLKDGNTRISHTFIEHEGNI---------------------------GWDFLGYNI-
>DS_cianobacteriafid|115404203|locus|VBIOscAcu116170_2852| [Oscillatoria acuminata PCC 6304]
GVDGIKN-----------------------LDP--------SERIKLAE---TLRL------DG----
KATPLLRVEIPN---P---------------------G---K----K-ETRPLGIPTIEDRAKQALAKLALE-
--PEWEAK-FEPNSYGF---RPGRS-----CHDAIIAIELQ----V----RR-------------Q-------
SKY---ILDADLKGCF-DNIDHEALIGKL--NT---------------FPI-MENQVR--AWLKSGI---M--
------K--
------------------------------------G---------D---VFY-K----TE------------
----
-----------------------------S-GTPTGGVIS-PLLANIALH-GLE-------------------
-----THIADK---------------------------FPTFRTRKGQEKG---------KM-----------
-----------------------KEWSEARTIRYADDFVI--LH------EKLEV----IQ-
EAKSETEKWLA-TIGLKLNENKTRIAH-TIKEVEGQKP-------------------------GFDFLGFNI-
>DS_cianobacteriafid|115405098|locus|VBIOscAcu116170_7132| [Oscillatoria acuminata PCC 6304]
GIDGVKS-----------------------LKP--------KQRLELAK---DLGK------HS----
KAKALRRVWIPK---P---------------------G---R----D-EKRPLGIPIIRDRAEQALVKQALE-
--PEWEAR-FEGTSYGF---RPGRS-----AHDAIGRIYAS----I----NQ-------------G-------
SYY---VLDADITKCF-DKINHEYLLSKL--DC---------------CLQ-HRRQIK--QWLKAGV---V--
---D--N--
------------------------------------G-------------IFE-D----TE------------
----
-----------------------------S-GTPQGGVIS-PLLANIALD-GMA-------------------
-----RLI-EE---------------------------------LYPKRKG---------QK-----------
--------------------------VKATLIRYADDFVV--IS------PEIEI----IN-
QCKIALENWLK-FVGLELKPEKTKICH----TLREI------------------EVNGEKVTPGFDFLGFTI-
>DS_cianobacteriafid|42716490|locus|VBIArtPla153080_5826| [Arthrospira platensis NIES39]

------------------------------------M---------------------------------PL-
----
----------Y------------------------------------VLTILS--------------------
----

-------------------------------CAIS-PILANIALD-GMV------------------------
RLI-ETMYPK----------------------------------KA---------NR----------------
---------------------VQASLVRYADDFVV--IS------PSLDI----IE-PCKNAIYEWLK-
PVGLQIKPEKTRVCH-TLNP---------------------IQYEGRTEEAGFDFLGFNI-
>DS_cianobacteriafid|42717648|locus|VBIArtPla153080_6402| [Arthrospira platensis NIES39]

----------M----NHDYLLSKI------H------------CPSRLKRDVK--QWLKAGV---L-----D-
-N---
-------------------------------G-------------VFE-D----TE-----------------
----
------------------------R-GTPQGGVIS-PLLANIALD-GMA------------------------
RLI-EN---------------------------------CIQKKKG----------R----------------
---------------------KQATLIRYADDFVV--IS------PSIEI----IE-QCKTAISEWLR-
NVGLEIKPEKTRVCH-TLNP---------------------LQHGEQTEEPGFDFLGFNI-

>DS_cianobacteriafid|115401811|locus|VBIOscAcu116170_3674| [Oscillatoria acuminata PCC 6304]
GIDGVVI-----------------------ISP--------NQRLGLAE---EIK-------GRL---
KAKPLRRVWIPK---P---------------------G---R-----DEKRPIGIPTIRDRARQALVKAALE-
--PEWESK-FEGTSYGF---RPGRS-----AHDAIVRIYAA----I----KM-------------N-------
SYY---VLDADIAKCF-DLINPEHLLSKI------H------------CPSRLKRDLK--QWLKAGV---L--
---D--N--
------------------------------------G-------------VFEET----YA------------
----
-------------------------------GTAQGGVIS-PLLANIALD-GMA-------------------
-----RLI-ET-----------------------------------KFPKK--------NSV-----------
--------------------------VQATLIRYADDFVV--IS------PRLEV----IE-
QCQTAISEWLK-PIGLEIKPFKTRVCHTLKPIQYDG----------------------KTEEPGFNFLGFNI-
>DS_cianobacteriafid|22636780|locus|VBIMicAer59304_5685| [Microcystis aeruginosa NIES843]
GVDGMIA-----------------------ISP--------EQRLNLTE---EIK-------GTL---
KAKPLRRVWIPK---P---------------------G---R----D-EKRPLGIPTIKDRARQALIKSALE-
--PEWESK-MEGTSYGF---RPGRS-----DHDAISRIYIT----I----NQ-------------S-------
SYF---VLDADIAKCF-DRINHDFLLSKI------H------------CPSSLKRDIK--QWLKAGV---L--
---D--N--
------------------------------------G-------------VFE-E----TE------------
----
-----------------------------T-GTPQGGVIS-PLLANIALD-GMA-------------------
-----RLI-ET--------------------------------LFPKKGNG----------K-----------
--------------------------NQAVLIRYADDFVV--IS------PSLEI----IE-
QCKTAISEWLK-PIGLELKPEKTRVCH-TLKP---------------------IEYNGKMEEPGFDFLGFNI-
>DS_cianobacteriafid|42725237|locus|VBIArtPla153080_3831| [Arthrospira platensis NIES39]

--------------MNHDYLLSKI------H------------CPSRLKRDLK--QWLKAGV---L-----D-
-N---
-------------------------------G-------------VFEDT----ET-----------------
----
--------------------------GTPQGGVIS-PILANIALL-GMG------------------------
RLIGKM---------------------------------YPQNTNN----------K----------------
---------------------PFATVIRYADDFVV--IS------KDLGI----IE-QCKTAISEWLK-
PVGLEIKPEKTRICHTLKSIEYNG----------------------KAEEPGFDFLGFNI-
>DS_cianobacteriafid|42723055|locus|VBIArtPla153080_2743| [Arthrospira platensis NIES39]

--------------MNHDYLLSKI------R------------CPSSLKKDLK--QWLKAGV---L-----D-
-N---
-------------------------------G-------------VFE-D----TE-----------------
----
------------------------A-GTPQGGVIS-PILANIALD-GMA------------------------
RLV-EI---------------------------------MYPQGTN---------NK----------------
---------------------PFARLVIYADNFVV--IS------KDLRI----IE-QCKTAISEWLK-
PVGLEIKPEKTRVCH-TLKP---------------------IQYEGKTEEQGFDFLGFNI-
>DS_cianobacteriafid|42723187|locus|VBIArtPla153080_2809| [Arthrospira platensis NIES39]

GVDGMVV-----------------------ISP--------KQRLEMAE---KIK-------GNL---
KTKPLRRVWIPK---P---------------------G---R----D-EKRPLGIPTIQDRARQALVKSALE-
--PEWEGR-FEGTSYGF---RPGRS-----AHDAIGRIYTA----I----NQ-------------G-------
QYY---VLDADIAKCF-DRINHDYLLSKI------H------------CPSVIKRDLK--QWLKAGV---L--
---D--N--
------------------------------------G-------------VFE-D----TE------------
----
-----------------------------A-GTPQGGVIS-PILANIALD-GMA-------------------
-----RLI-ET---------------------------------MYPKASG---------GK-----------
--------------------------VKATLIRYADDFVV--IS------PSLDI----IE-
QCKTAISRWLK-PIGLEIKPEKTRVCH-TLNP---------------------IQYEGKTEEPGFDFLGFNI-
>DS_cianobacteriafid|42724297|locus|VBIArtPla153080_3363| [Arthrospira platensis NIES39]

--------------------------------G--------------IGIPTIQDRARQALVKSALE---
PEWESR-FEGTSYGF---RPGRS-----AQDAISRIYLS----I----NK-------------G-------
EYY---VLDADIAKCF-DRINHDYLLSKI------H------------CPSNLKRDLK--QWLKAGV---L--
---D--N--
------------------------------------G-------------IFE-D----TE------------
----
-----------------------------A-GTPQGGVIS-PLLANIALD-GME-------------------
-----RLV---KG---------MY-----------
--------------------PNKRTATQVNLIRYADDFVV--IS------KDLGI----IE-
QCKTAISEWLK-PVGLEIKPEKTRICH-TLNP---------------------IEYNGKIEEPGFDFLGFNI-
>DS_cianobacteriafid|21547658|locus|VBICyaSp130209_4633| [Cyanothece sp. ATCC 51142]
GVDGKKI-----------------------IHP--------QQRYQLAL---SLNL------KGY---
KSKPLRRVWISK---P---------------------G---K----D-EKRPLGIPTITDRAMQCLIKLCME-
--PYWEAK-FEGNSYGF---RPGRS-----THDAIEAIFNH----I----RY-------------K-------
TKY---VLDADISKCF-DKINHKYLLDKT-------------------DCPYFKSIIK--QWLKAGV---M--
---D--K--
------------------------------------G-------------IFE-S----TD------------
----
-----------------------------S-GTPQGGVIS-PLLANIALD-GMI-------------------
-----RDI-QK-------------------------SFPNSITREGKRIRG----------------------
--------------------------YQPKIIRYADDFVI--LH------HELEI----IN-
HTQKLVNKWLE-KVGLELKPSKTRICH----TLNDI------------------MIDGKMEKAGFDFLGFTI-
>DS_C.w.I1/NZ_AADV01000039/6112..8597/Crocosphaera watsonii/CL2
GVDGKKS-----------------------LRP--------NQRLKLVN---ELRL------KGY---
KAKALRRVWIPK---P---------------------G---R-----DEKRGLGIPTMKDRAMQALVKSALE-
--PYWEAQ-FEGTSYGF---RPGRS-----AQDAISRIFLA----I----KT-------------N-------
AKY---VLDADIAKCF-DKINHDYLLSKV------D------------CPHNIKRIIK--QWLECGV---M--
---D--K--
------------------------------------G-------------IFE-E----TD------------
----
-----------------------------S-GTPQGGVIS-PLLANIALH-GMI-------------------
-----IDI-EN-----------------------------HFPRTKRREDG---------SL-----------
-----------------------KQGYKPKIIRYADDFVI--LH------TDYDV----IL-
QCKNLVAQWLE-KVGLELKPEKTSIRH-TLKSIVHN---------------------GKTIEPGFDFLGFNI-
>DS_cianobacteriafid|42716762|locus|VBIArtPla153080_5960| [Arthrospira platensis NIES39]
GVDGVKS--------------------LS-WKG----------EINLVG---DLKL------GS----
KAKPTRRVDLTE---T---------------------V---G----D-APSRNGDQTIFDRAGQGLVRLALE-
--PEWEAK-FESNCYGF---RPGRS-----CHDAIEAISNH----L----ES-------------E-------

```
PKW---VLDAQITKCLGDRINSDRLLDKL--GT---------------FPR-LRRQIR--AWLKSGV---M--
------D------------------------------------------------------------------
------------------------------------G---------K-QLFP-------TK------------
-------------------------------------------------------------------------
-----------------------------E-GTSQGGIIQ-PLLANIALH-GME-------------------
-----EDL-LK-----------------------------MADQLSGRKPV---------NR-----------
--------------------------NELSVIRYADKLVI--LH------EDGAV----IH-
HCQQVIREWLK-PWGLELKPEPTRIAH----TLDGEDA-------------------------GFDFLGFHI-
>DS_cianobacteriafid|23984237|locus|VBITriEry99848_0561| [Trichodesmium
erythraeum IMS101]
GIDGKKS--------------------LT-LKE----------RVNLAN---NLVM-------SH---
KAKSTCRIWIPK---P---------------------G---K-----TEKRPLGILTISERAKQGLVKMAIE-
--PEWEAK-FEPNIYGF---RPGRS-----CMDAVEGIKAA----I----KQ-------------K-------
SKY---VLNADIAKCF-DNIDHEKLLDKI--GT---------------FPK-VRRQIK--AWLKSGV---Y--
---DNES------------------------------------------------------------------
--------------------------------------------------WFLMD------------------
-------------------------------------------------------------------------
-----------------------------E-GISQGEVIS-GLWANIALH-GME-------------------
-----KIV-KE---------------------------------FAYSLSG---------KK-----------
----------------------VKNEKEITLVRYADHFVI--LH------PNLNV----VT-
KAKALVEEFLR-GMGLELKPEKTRLTHTLIQVGEEE--------------------------PGFNFLGFNI-
>DS_cianobacteriafid|21544066|locus|VBICyaSp130209_2854| [Cyanothece sp.
ATCC 51142]
GIDGIKS-----------------------LRP--------SARWKLIQ---ELKF------TG----
KSKPVRRVWIPK---P---------------------G---K----S-EKRPLGIPTIKDRALQTLAKMALE-
--PEWEAK-FEPHSYGF---RPGRS-----VHDAVEAIFTG----I----SK-------------K-------
KKF---ILETDIEKCF-DKINHSELIKKL--NT---------------YPK-LRRQIK--AWLKAGI---I--
---DENQ------------------------------------------------------------------
--------------------------------------------------LF--P----TE------------
-------------------------------------------------------------------------
-----------------------------E-GTPQGGTLS-PLLANIALH-GME-------------------
-----NLL-KK---------------------AFPRLGVGNRQTWFHSKGQ---------EF-----------
--------------------------YSPILIRYADDLVV--IH------EDQKV----IE-
VCKELINEWLR-TIGLRLKDSKTKIVH----TYDEH----------------------KRNKPGFDFLGFNF-
>DS_C.sp.I1/X71404/446..2898/Calothrix sp./CL2
GIDGVKS-----------------------LKP--------SARLTLVM---NMKL-------NH---
KVKATRRVWIPK---P---------------------G---N----V-EKRPLGIPTMQDRATQSLVKLALE-
--PEWEAK-FEPNSYGF---RPGRN-----AHDAREAIFNS----I----RY-------------S-------
NKW---VLDADISKCF-DKINHEKLLTKI--NT---------------FPT-MRRQIK--AWLKAGV---L--
---D--N------------------------------------------------------------------
------------------------------------G-------------HFS-E----TT------------
-------------------------------------------------------------------------
-----------------------------E-GTPQGGVIS-PLLANIALH-GLE-------------------
-----KLV-KE---------------------------------FAASQRG---------GK-----------
----------------------VKNQNSISLIRYADDFVI--LA------PNKTQ----II-
VLKEIVKTWLA-EMGLELNPNKTRIVS----TFKSSEIFASQEV-------------------GFNFLGFNV-
>DS_cianobacteriafid|115439943|locus|VBIStaCya5387_0048| [Stanieria
cyanosphaera PCC 7437]
GVDGIKS-----------------------LTP--------AKRLEMVN---HLNI------EG----
KSKPTRGVWIPKRTSDLRSPYGKRAFEVSSNDVSSANG---E-------KRPLGIPCMKNRVIQCLVKLALE-
--PEWEAK-FEPNSYGF---RPGRS-----CHDAIGAIFNV----I----RY-------------E-------
PKY---VLDAAIAKCF-DKIDHQKLLNKL--ET---------------YPG-IKKQIK--AWLKSGV---I--
---D--D------------------------------------------------------------------
------------------------------------N-------------WF--P----TD------------
```

-----------------------------E-STPQGGVIS-PLLANIALH-GME-------------------
-----MAI-KN---------------------LARNLDLTTSTGQVITDRR--------VKE-----------
--------------------------KKLHLIRYADDFVI--LH------NDINV----IY-
KCKETIESFLL-EMGLELKPSKTKISHTLNKYQGNI---------------------------GFDFLGFNV-
>DS_cianobacteriafid|115388851|locus|VBIDacSal132842_2432|
[Dactylococcopsis salina PCC 8305]
GIDGIKS-----------------------LSP--------KQRLNLAE---NLTL------TG----
KGKSLRRVWIPK---P---------------------G---R-----KEKRGLGIPVMEDRARQALLKLALE-
--PEWEAK-FEPNSYGF---RPGRS-----CHDAGQAIYVA----I----NQ-------------Q-------
SKW---VLDADISKCF-DRIDHNVLLRKL--NT---------------TST-IARQIR--AWLKSGV---L--
---DRGD--
--WF--P----TN------------

-----------------------------E-GTPQGGVIS-PLLANIALH-GLE-------------------
-----KYI-KQ-----------------------WAETWKGYKDMNGKSRG--------KKQ-----------
------------------------KRHSISVIRYADDFVV--LH------KDKSI----IQ-
EAKMLIEQWLH-GLGLELSESKTRTCH----TLHDT----------------------NETRAGFDFLGWNV-
>DS_cianobacteriafid|115392614|locus|VBIDacSal132842_1186|
[Dactylococcopsis salina PCC 8305]
GIDGVKS-----------------------LSP--------KQRLSLAE---SLTL------TG----
KGESLRRVWIPK---P---------------------G---R-----KEKRGLGIPVMEDRARQALLKLALE-
--PEWEAK-FEPNSYGF---RPGRS-----CHDAEQAIFNA----I----RY-------------K-------
PKW---VLDADISKCF-DRINHDVLLQKL--NT---------------IPT-IARQIR--AWLKSGV---L--
---DRGD--
--WM--P----TN------------

-----------------------------E-GTPQGGVIS-PLLANIALH-GLE-------------------
-----DYI-KQ---------------------------------WAETWKG--------GKQ-----------
-----------------------ANRISISLIRYADDFVV--LH------KDKSI----IQ-
QAKTLIEQWLH-GSGLEISESKTRICH----TLYDS----------------------EEKKAGFDFLGWNI-
>DS_cianobacteriafid|115388988|locus|VBIDacSal132842_1986|
[Dactylococcopsis salina PCC 8305]
GVDGVKS-----------------------LNP--------KQRLNLAE---NLTL------TG----
KGKSLRRVYIPK---P---------------------G---K-----AEKRSLGIPVMEDRARQALLKLAME-
--PEWEAK-FEPNSYGF---RPGRS-----CHDAEGAIYVS----I----NQ-------------K-------
PKW---VLDADISKCF-DRINHDVLLRKL--NT---------------TPT-IARQIR--AWLKSGV---L--
---DRGD--
--WM--P----TE------------

-----------------------------E-GTPQGGVIS-PLLANIALH-GLE-------------------
-----EYI-KQ---------------------------------WAETWKG---------YK-----------
-------------NENGRQMSKINRRQSITLIRYADDFVV--LH------RDKSI----VQ-
QAKTLIEHWLH-GLGLELSESKTRICH----TLYDS----------------------EEEEAGFDFLGWNV-
>DS_Tr.e.I2/CP000393/5587083..5589603/Trichodesmium erythraeum/CL2
GIDGIKN-----------------------LPS--------MQRFNLVD---LLKR------HRF---
KASPTRRVWIPK---P---------------------G---K-----DEKRPLGISTMYDRALQALVKLGRS-
--PEWEAH-FEPNSYGL---RPGRS-----THDAIAAIYVS----I----NK-------------K-------
PKY---VLDADISKCF-DRINHDALLRKI-------------------GRTPYRRLIK--QWLKSGV---F--
------D--
------------------------------------N-------------KQFSD----TL------------
----
-----------------------------E-GTPQGGVIS-TLLVNIALH-GME-------------------
-----KCL-EK---------------------------------YAETLPG---------KK-----------

----------------------RDNKQALSLIRYADDFVI--LH------EDIKV----VM-
QAKTVIQEWLN-QVGLELKPEKTKIAHTLEEYEGNK--------------------------PEFDFLGFNI-
>DS_cianobacteriafid|115428732|locus|VBICriEpi239080_3135| [Crinalium epipsammum PCC 9333]
GVDGVKS-----------------------LSP--------EARLKLVR---ELKL------TG----
KSKPTRRVWIPK---P---------------------G-TDE-------KRPLGIPTMYDRALQAVVKATLE-
--PEWEAF-FEPNSYGF---RPGRS-----CHDAVNQVKKA----IM---QK---------------------
AKY---VLDADIAKCF-DRINHEKLLQKL--NT---------------KGK-VRQQIK--AWLKSGV---V--
---D--Q--
------------------------------------G-------------SFTAT----SE------------
----
-------------------------------GTPQGGVIS-PLLANIALH-GME-------------------
-----ERIKQE-----------------------FPRMSHSGRETWYHKKG--------EEF-----------
--------------------------PTPDVIRYADDFVI--FH------QNKTV----VQ-
RCRDIISNWLS-DIGLQLKPEKTRLSHSLNPELSDD------------------------GIAGFDFLGHHI-
>DS_cianobacteriafid|115547536|locus|VBIAnaSp49473_3044| [Anabaena sp. 90]
GVEVVKS-----------------------LGY--------AQRLELAN---SLGT------TR----
KVKPTRRIWIPK---P---------------------G---T--D-E--KRPLGIPTMSDRANQAFAKLALE-
--SEWEAK-FEPNSYGF---RPGRA-----VHDAIEAIYLA----I----HC-------------K-------
AKY---VLDADISKCF-DNIDHQKLLSKL--NT---------------YPS-MKRLIR--SWLKAGF---M--
------D--
------------------------------------R---------R-D--LF-P----TK------------
----
-----------------------------M-GTPQGGVIS-PLLANIALH-GME-------------------
-----EVI-KE---------------YANTLPTRRN-----YGRKQNR-NA----------L-----------
--------------------------S---LIRYADDFVI--IH------EDINV----VL-
GAKAVIEGFLK-DIGLELKPSKTRICH----TFEEY----------------------EGEKPGFDFLGFNI-
>DS_N.sp.I4/AP003604/45422..47908/Nostoc sp./CL2
GVDGRKN-----------------------LSP--------KARLILVQ---SMKL------GD----
KASPTRRVWIPK---P---------------------G---S--SGE--KRPLSIPTLYDRALQSLVKLALE-
--PEWEAR-FEPNSFGF---RPGRN-----AHDAMKAIFNT----I----KF-------------K-------
PKY---VLDADIAKCF-DKIDHNVLLSKL--NT---------------FPT-ISRQIR--AWLKAGV---I--
------D--
------------------------------------F---------S-EYALH-T----TS------------
----
-----------------------------M-GVPQGGTIS-PLLANIALH-GME-------------------
-----NRI-KQ---------------VALTLPGCKSE---------NR-QA----------I-----------
--------------------------S---LIRFADDFVI--LH------KDLAV----IQ-
RCQQIISEWLS-ELGLELKPSKTRISH----TLNMY----------------------EG-KVGFDFLGFTV-
>DS_G.v.I1/BA000045/168850..171364/Gloeobacter violaceus/CL2
GVDGVKS-----------------------LTP--------KARLALTK---NLRI------SE----
KAKPMRRVWIAK---P---------------------G---T---QE--KRPLGIPTMTDRARQALLTLALE-
--PEWEAR-FEPNSYGF---RPGRS-----CHDALQAIYNA----I----RQ-------------Q-------
SKF---VLDADIAKCF-DRIDQQALLKKM--NT---------------SSA-IRRQIR--AWLKAGV---M--
------E--
------------------------------------G---------S-E--LF-P----TP------------
----
-----------------------------T-GTPQGGVIS-PLLANIALH-GME-------------------
-----ERV-KQ---------------VS---------------------KM----------A-----------
--------------------------Q---LIRYADDFVC--IH------TDQQI----VQ-
SCQTVLEEWLA-GMGLELKPSKTRIAH----TLLLE----------------------EG-QPGFDFLGFTV-
>DS_cianobacteriafid|21588186|locus|VBICyaSp112625_3397| [Cyanothece sp. PCC 8802]

GVDGVKS-----------------------LTQ--------KQRMELVE---NLTL------KG----
KAKPTRRVWIPK---P--------------------NG---E-------KRPLGIPTITDRAKQYLVKLALE-
--PQWEAK-FEHNSYGF---RPGRS-----CHDAIEAIYIA----IS---RK---------------------
AKF---VLDADIAKCF-DKINHEKLLTKL--ET---------------YPE-IRKSIK--GWLKSGF---R--
---DDKE--
--WF--P----TD------------

-----------------------------E-GTPQGGVIS-PLLANIALH-GME-------------------
-----TII-KD---------------------------------FARTWKG--------EKA-----------
-----------------------KNEQSISVIRYADDFVI--LH------ENLDI----IQ-
KCKSIIENWLS-EIGLELKPSKTRISHTLQEVEGKI---------------------------GFNFLGFHI-
>DS_cianobacteriafid|21557688|locus|VBICyaSp136448_4440| [Cyanothece sp. PCC 7424]
GVDGVKS-----------------------LTP--------KQRMNLVG---QLKL-------TC---
KTKPTRRVWIPK---P---------------------G---K-----DEKRPFLIPCMSDRALQALVKIALE-
--PEWEAK-FEPNSYGF---RPGRG-----CHDAIGAIFNQ----LG---AK---------------------
AKY---VLDADISKCF-DKINHEKLLQKL--NT---------------FPT-LRRQIR--AWLKAGV---M--
------D--
------------------------------------G-------------NKLFP----TE------------
----
-----------------------------E-GTPQGGVVS-PLLANIALH-GME-------------------
-----EII-KS------------------------FAQNPGELRQEFSNRG--------KGR-----------
-------------------------EQSISLIRYADDFVL--IH------ESLAV----VE-
KGKEIIETWLR-ELGLTLKPEKTQITHTLDKHQGKV---------------------------GFNFLGFNI-
>DS_cianobacteriafid|115683636|locus|VBIOscNig7962_7871| [Oscillatoria nigroviridis PCC 7112]
GIDGVKS-----------------------LSP--------IQRVKLVK---RLRV------TG----
KSKPTRRVMIPK---P---------------------G-SDE-------KRPLGIPTIEDRALQALVKSALE-
--PEWEAQ-FEPNSYGF---RAGRS-----CHDAIEAIFNS----I----RL-------------K-------
AKY---VLDADIAKCF-DRIDHKALLAKV--NT---------------YPT-LRHQLK--VWLKAGY---C--
---AEGS--
--LF--P----TD------------

-----------------------------E-GTPQGGVIS-PLLANIALH-GME-------------------
-----NRV-KQ---------------------------------YAETLKG--------KKR-----------
-----------------------DNRQALSLIRYADDFVI--MH------EDLSV----VK-
KCQEIIAEWLR-DMGLELKASKTKLTHTLIKIDGNV---------------------------GFEFLGFHV-
>DS_cianobacteriafid|115580159|locus|VBIMicSp236384_0858| [Microcoleus sp. PCC 7113]
GVDGKKS-----------------------LTP--------KQRLTLVK---NLRL------TG----
KSKPTRRIWIPK---P---------------------G---K-----DEKRPLGIPTIHDRALQALVKLALE-
--PEWESK-FEPNSYGF---RPGRS-----CHDAVGQIYLS----I----NK-------------Q-------
PKY---VLDADISQCF-DKINHNALLEKL--NT---------------FPT-LRRQVR--SWLKAGA---I--
------D--
------------------------------------E-------------AQLIP----TS------------
----
-----------------------------E-GTPQGGVIS-PLLANIALH-GME-------------------
-----QLT-KA--------------------------------------VS----------------------
--------------------------KTACLVRYADDFVI--LD------KDITV----VQ-
RCKKAIEEFLK-GMGLELKPSKTRISHTLHKYEGNV---------------------------GFDFLGFNI-
>DS_cianobacteriafid|115660493|locus|VBIChaMin231992_2004| [Chamaesiphon minutus PCC 6605]
GVDGVKS-----------------------LTP--------KQRLILVD---KIKL------GT----
KAKPTRRVWIPK---P---------------------G---T-----SEERPLGIPTMEDRALQAVVKMVLE-

--PEWESK-FEPNSYGF---RPGRS-----CHDAIEAIFSS----I----SK------------K-------
SKY---VLDADISKCF-DRINHNKLLSKL--NT---------------FPT-LRKQIR--AWLKAGV---M--
------D--
------------------------------------G---------K---KLF-P----TN------------
----
-----------------------------E-GTPQGGVLS-PLLANIALH-GLE-------------------
-----ELI-MG-------------------LAPKFDMKRPNGNQ-LPVRD---------KL------------
--------------------------KSICCVRYADDFVI--LH------EDLKV----IN-
QCKKEVEEWLS-DIGLELKPSKTRIAH----CLSDL---------------------DGEKAGFNFLGFNI-
>DS_cianobacteriafid|115390726|locus|VBIDacSal132842_0370|
[Dactylococcopsis salina PCC 8305]
GVDGVKS----------------------LSP--------VARMKLVN---NLKL------GS----
KVKPTRRVKIPK---P-------------------NG---E-------ERPLGIPTMYDRALQALVKLALE-
--PEWEAV-FEPNSYGF---RAGRS-----AHDAVTAIFDA----I----RY------------K-------
PKY---VLDADLAKCF-DRINHERLLNKI--KT---------------FPT-FRKQIR--AWLKAGV---M--
------E--
------------------------------------G-------------KEFSP----TS------------
----
-----------------------------E-GTPQGGVIS-PLLANIALH-GME-------------------
-----NEI-KA--------------------------IAHTFDMKTNNG---------YQ-----------
-------------------VSASNKRRSVCVIRYADDFVI--LH------ESLAV----VQ-
RCKEVVSNWLA-DMGLELKPSKTRIAHTLENYENEK-------------------------AGFDFLGFNI-
>DS_cianobacteriafid|21539290|locus|VBICyaSp130209_0491| [Cyanothece sp.
ATCC 51142]
GVDGVKS----------------------LTP--------KQRLTLVN---QLKL------SP----
KVKPTRRVWIPK---S--------------------G---T-----DEERPLGIPTMYDRALQGLVKMALE-
--PEWEAR-FEPNSYGF---RIGRS-----CHDAINAIFKA----I----KC------------K-------
SKF---VLDADISKCF-DRINHKKLLEKL--NT---------------YPT-LRKQIR--AWLKAGV---M--
------D--
------------------------------------G---------K---ELF-P----TL------------
----
-----------------------------E-GTPQGGVLS-PLLANIALH-GME-------------------
-----ECI-KE-------------------LTESHSMKRENGKYEKPLKH---------KR-----------
--------------------------QSVSLIRYADDFVI--LH------EDITF----IL-
KCKDRIAKWLN-GMGLELKPSKTRLTH----TLNDY---------------------EGEKAGFDFLGFHI-
>DS_cianobacteriafid|115579300|locus|VBIMicSp236384_3645| [Microcoleus
sp. PCC 7113]
GVDGQRS----------------------LTP--------KQRQNLIG---QLKL------GT----
KVSPTRRVWIPK---P--------------------G---K-----EEKRPLGIPTMKDRALQALVKLALE-
--PEWEAQ-FEPNSYGF---RPGRS-----CQDAISAIQTV----I----KQ------------K-------
AKY---VLDADIAQCF-DRIDHEALLNKL--NT---------------SPT-IRRQIR--AWLKAGV---M--
------D--
------------------------------------N---------M---QYF-D----TS------------
----
-----------------------------E-GTPQGGVIS-PLLANIALH-GME-------------------
-----WRI-KE-------------------YVETCDLKRSDGKYQLPKRD---------KR-----------
--------------------------DSVSIIRYADDFVI--LH------NDITV----VQ-
GCREVISEWLK-GMGLELKPSKTRIAH----TLNEH---------------------GQEKPGFNFLGYYV-
>DS_cianobacteriafid|115428478|locus|VBICriEpi239080_4618| [Crinalium
epipsammum PCC 9333]
GVDGVKS----------------------LTP--------VQRLALVR---KLAL------KG----
KSKPTRRVWIDK---P--------------------G---T-----TEKRPLGIPTMYDRALQALVKLALE-
--PEWEAR-FEPNSYGF---RPGRS-----CHDAIGAIFVT----I----NQ------------K-------
AKY---VLDADIAKCF-DRINHRELLKKL--NT---------------FPT-LKRQIG--AWLKSGV---M--

------D--
------------------------------------G---------K---QMF-P----TS------------
----
-----------------------------E-GTPQGGVIS-PLLANIALH-GME-------------------
-----ERI-KQ--------------------FAETLPSRSG-----FGKRD---------KR-----------
--------------------------KSLSLIRYADDFVI--LH------EDITV----VK-
RCKEIISEWLM-GMGLELKPSKTRLAH----TLIEY----------------------EGQDAGFNFLGFNI-
>DS_cianobacteriafid|21539742|locus|VBICyaSp130209_0717| [Cyanothece sp. ATCC 51142]
GVDGVKS-----------------------LTP--------KQRLLLVN---KLKL------GT----
KVKPTRRVWIPK---P---------------------G---R-----DEKRPLGIPTMKDRALQGLVKMALE-
--PEWEAK-FEPNSYGF---RPGRS-----CHDAIGAIFSA----I----RL------------K--------
PKY---VLDADIAKCF-DKIDHERLLEKI--NT---------------YPT-LRKQIR--AWLKADV---M--
------D--
------------------------------------G---------K---KLF-P----TS------------
----
-----------------------------E-GTPQGGVIS-PLLANIALH-GME-------------------
-----SRI-KE--------------------MAKDIDWRNEKGHL-ISISA---------RR-----------
--------------------------KSISLIRYADDFVI--IH------ENLTI----VQ-
RCREIISEWLI-GMGLEIKPSKTRLIH----TLQEY----------------------EGEKPGFNFLGFNI-
>DS_cianobacteriafid|21575043|locus|VBICyaSp125535_1555| [Cyanothece sp. PCC 8801]
GVDGVKS-----------------------LTP--------KQRLNLID---KLKL------GT----
KVKPTRRVWIPK---P---------------------G---T-----EEKRPLGIPTMYDRALQGLVKLALE-
--PEWEAK-FEPNSYGF---RPGRS-----CQDAIGAIFLA----I----NK------------K--------
AKY---VLDADIAKCF-DRIDHEQLLNKL--NT---------------YPT-LRKQIR--AWLKAGV---M--
------D--
------------------------------------G---------K---ELF-P----TS------------
----
-----------------------------E-GTPQGGVIS-PLLANIALH-GME-------------------
-----NEI-NK--------------------LAETFDMRGPDGKL-LGKRD---------KR-----------
--------------------------KSVSLIRYADDFVI--LH------EDITI----VQ-
RCKEFISEWLK-DMGLELKPSKTRLAH----TLEEY----------------------NKEKPGFDFLGFNV-
>DS_Actinobacteriafid|23713660|locus|VBIStrAve112782_0248| [Streptomyces avermitilis MA4680]
GIDGQKA--------------------LS-PEK---------RGKTARQ---ILAD------PMS---
HPQPVRRVYIPK--AN---------------------G---K-------RRPLGIPVIRDRVDQARFKNALE-
--PEWEAR-FEARSYGF---RPGRG-----AWDAIEMIFNVAGRRT----AK---------------------
RLW---VLDADLSAAF-DHISHQHLMDSV--GL---------------FPG--RRQIQ--QWLRAGV---M--
---E--D--
------------------------------------G-------------RFV-S----TP------------
----
-----------------------------E-GTPQGGVIS-PLLMNIALH-GMG-------------------
-----EVIGAN-----------------------------------RPWNA---------KT-----------
--------------------------TSPTLVRYADDFVV--FC------TTENE----AI-
KAKQDLAAWLE-PRGLSFNEEKTRVVH----L--SS---------------------------GVDFLGFNV-
>DS_clostridiafid|161807916|locus|VBICloPas18034_2678| [Clostridium pasteurianum BC1]
GVDGEIA--------------------LT-NTE---RLKLFYDLSELHI-------------EKH---
NPKPSRRTYIKK--KN---------------------G---K-------LRPLSIPTIRDRIYQNIIKGTLE-
--PQWEAR-FEPISYGF---RPKRG-----CHDAIARIFRS----CHSGSRK---------------------
-RW---IFEGDFKGCF-DNLKHDYIMEQI-------------------KEFPYDNLVD--KWLKAGY---V--
---D--N--
------------------------------------G-------------VFNKT------------------

----
-----------------------------QFGSGQGNIVS-PLLANIALK-GME-------------------
-----DTLGIE--------------------------------YKPVKNNG----------------------
------------------KIVSYTNVGKYTLVFYADDFVI--MC------NTQKD----AE-
DVYELLKPYLG-KRGLELSKEKTRIVT----I--DE---------------------------GFNFLGFNI-
>DS_Bacillifid|18918903|locus|VBIBacCer120424_5584| [Bacillus cereus Q1]
GIDGYIS-----------------------------NTPQ--ERVELFN---KLSR------
YSVRNIKVKPARRTYIPK--KN---------------------G---K-------
LRPLGIPVIVDRVYQNAFKNALE---PQWEAK-FEMTSYGF---RPKRS-----THDAMSDLFTK----L---
-SK-------------GSA-----KGW---IFEGDFEGCF-DNLNHDYIMGCI-------------------
NNFPNKSIIR--DWLESGY---------VDND---

--VFNET----TK--
--GTPQGGIIS-
PLLANVALH-GME------------------------KEIGVR------------------------------
--YIHTTRQG------DTLYS-------------------------------------NSVGVVRYADDFVI-
-VC------PTEEE----AY-GMYDKLEPYLN-KRGLNLAKDKTRVVH----I--SK----------------
-----------GFDFLGFNF-
>DS_clostridiafid|19384870|locus|VBICloBot822_0094| [Clostridium
botulinum A3 str. Loch Maree]
GIDGFKV-----------------------ITE--------WDRIKLFN---SLKD------
YSIKNIKSQPAKRTYIPK--KN---------------------G---K-------
LRPLGIPIIKDRIYQNIVKNALE---PQWESK-FESIAYGF---RPKRS-----THDAIEQLYLK----L---
-RK-------------GSK-----RQW---IFEGDFKGCF-DNLNHEYIMECI-------------------
NDFPAKEAVY--RWLKAGY---I-----D--N---
---N-----------
--VFR-N----TN--
--E-GTPQGGIIS-
PLLANIALH-GME------------------------EELGVK------------------------------
--YQFTKRQG---------YC----------------------------------LRDNSIGIVKYADDFVI-
-LC------KTKEE----AE-TMYERLSPYLK-KRGLELAEDKTGITH----I--SK----------------
-----------GFDFLGFNI-
>DS_B.a.I1/AE011190/6579..9109/Bacillus anthracis/CL1
GIDGITT--------------------NTPEDR--------VKLFHLLK---GYSV------RNI---
KAFPVKRAYIPK--KN---------------------G---K-------KRPLGIPVIKDRIFQNMVKNALE-
--PQWECR-FESMSYGF---RPKRS-----AHDAMANLFLK----L----SR-------------GTN-----
RAW---IFEGDFQGCF-DNLNHEHILSCI-------------------EGFPYSNAIN--QWLNAGC---I--
------D--
------------------------------------N-------------KTFYK----TE------------
----
-----------------------------T-GTPQGGIIS-PLLANIALH-GME-------------------
-----KELGVR--------------------------------YHFPKRDG----------------------
---------------------AMLYPDSIGIVRYADDFVI--VC------NSKEE----AE-
SMYAKLQPYLD-KRGLKLAEEKTRVVH----I--TD---------------------------GFDFLGFNF-
>DS_Bacillifid|18824950|locus|VBIBacCer120511_0064| [Bacillus cereus
AH187]
GVDEHTA-----------------------LSR-----RERNLLYEQLK---KLNT------LQH---
RPKPAKRIYIVK--KN---------------------G---K-------LRPLGIPTIKDRVYQNIVRNALE-
--PQWEAR-FEAISYGF---RPKRS-----THDAIRSIFNR----INGGTKK---------------------
-KW---IFEGDFQGCF-DHLNHEWILKQT-------------------SYFPGRKLLK--RWLKMGY---M--
------E--
------------------------------------Q-------------SFFAE----TQ------------
----
-----------------------------E-GTPQGGIIS-PLLANIALH-GME-------------------

-----ETLGIT---------------------------------YKKNYKANDSYIMNPACK-----------
----------------------------FTLIRYADDFVV--LT------ETKEQ----AL-
SVYMRLRPYLK-DRGLELSPEKTKVTH----I--EE--------------------------GFEFLGFLI-
>DS_B.me.I2/AF142677/34045..36400/Bacillus megaterium/CL1
GVDGYTA--------------------SK-PNE----------RIKLYQ---QLVK------
CNVFRHRPKPAKRTFIPK--KN--------------------G---K-------
LRPLGIPTMRDRVYQNVVKNALE---PQWEVK-FEPTSYGF---RPKRS-----THDAISNLFNK----
LNTNSKK----------------------KW---VFEGDFLGCF-DHLNHNWIMEQT--SM-----------
---FPG--NTLIK--RWLNMGY---------IEQD-------------------------------------
----
-----MLH-T----TT--
---E-GTPQGGIVS-
PLLANIALC-GME------------------------EEI------------------------------
GIVYKKTYKSNG---------GY--------------------------------
KIDPKKIGRVLYADDFVI--VT------ETKEQ----AE-SMYQNLTPYLR-KRGITLSKEKTRVTH----I-
-ED--------------------------GFDFLGFSL-
>DS_cianobacteriafid|115391721|locus|VBIDacSal132842_4122|
[Dactylococcopsis salina PCC 8305]
GVDKEVL-----------------------------------NTPDERV---KLVN-------
SWEMPKANPTRRVYLPK---P-------------------NG---K-------
KRPLGIPTVRDRVAQAIIKNILE---PEWEAV-FEPNSYGF---RCGRS-----CHDAIEQCFIK----
FRAGNKG-------------G-------HLW---VLDADIKGFF-DNIAHESILTAI----------------
---ESIPRGDLIE--GWLKAGY---L--------D------------------------------------
----
-----KGVLN----PT--
---VMGTPQGGVIS-
PLLANIGLH-GLE-----------------------DFI-KS----------------------------
--------VN--PKLGVIRYADDFVV-
-TS------KDKES----LE-HILDQIKQWML-ERGLEISAEKTRIVS----M--EE----------------
-----------GFDFLGFNL-
>DS_cianobacteriafid|115690797|locus|VBICyaApo239906_2949|
[Cyanobacterium aponinum PCC 10605]
GIDKEVI--------------------NT-PAQ-------------------RVKLV-----
NEWKMPKAVPTKRVYIPK---P-N-------------------G---K-------
KRPLGIPTVRDRVAQAIVKNSLE---PEWEAA-FEPNSYGF---RCGRS-----CHDAIGQCYLR----L---
-RG-------------DSEKGGTHDKW---VLDADIKGFF-DNIAHESILNMI-------------------
DSHPKKELIK--GWLKAGF---I-----D--S--
---G-----------
--VHN-L----TE---
---T-GTPQGGVIS-
PLLANIGLH-GLE-----------------------KHI-KQ----------------------------
--------CN--PKLGIIRYADDFVV-
-TA------KDKES----LE-EVLIQIKQWLS-ERGLEISAEKTRIVH----I--DN----------------
-----------GFNFLGFNL-
>DS_cianobacteriafid|23995189|locus|VBITriEry99848_5996| [Trichodesmium
erythraeum IMS101]
GRDAQTA--------------------KTSVEK----------VKLVKE---MLTY------RLW---
QAKPAKRVYIPKANRQ--------------------------------QGPLGIPTVKNRVAQAVVKNGLE-
--PIWDAE-FETNSYGF---HPGRS-----CHDPLEQFWIR----L----QK-------------GK------
DTW---ILDVDIKQDF-DNITHEYILKAI-------------------GEIPGRELIK--QWLKAGY---L--
------E--
--AEVFH----KT------------

-----------------------------EGGTSSRGIIS-PLLANIAFD-GME-------------------

-----RLL-AR-------------------YKTVKTYQCTRPTTDEEYTKK--------KKL-----------
--------------------------DKYGFIRYADDFII--TA------RSEED----IK-
AIIPTIEKWLS-ERGLELNKDKTNLVH----I--EQ----------------------------GFNFLGFNV-
>DS_cianobacteriafid|115675501|locus|VBIOscNig7962_2874| [Oscillatoria nigroviridis PCC 7112]
GIDGQTA--------------------TT-PSE----------RVKLVK---EMKD--------
YTLWKAQPARRVYIPK--AN---------------------G---K-------
QRPLGIPTVKNRIAQAVIKNALE---PSWEAR-MEGSSYGF---RPGRS-----CHDAIEHSWIR----
LNKQGND----------------------RW---VLDADIKGAF-DNISHNFILKTI--GE----IP------
-------GRELIK--QWLKAGY---------VESE--------------------------------------
----
-----IFH-E----TK---
---S-GTPQGGIIS-
PLLANIALD-GIE------------------------QFL-SQ------------------------------
----FKKRQG---------KN--------------------------------KSPRAPKYGFVRYADDFII-
-TA------ETKED----IE-EIIPSVKELLK-TRGLELNEDKTNIVH----I--EQ----------------
-----------GFNFLGFNV-
>DS_cianobacteriafid|115390656|locus|VBIDacSal132842_3607| [Dactylococcopsis salina PCC 8305]
GVDGRIA--------------------LT-PEE---------RWELVCE---LQVL------NDP---
IASPTRRIQIPK--SN---------------------G---K-------KRPLGIPIVTDRIRQAVVKEALE-
--PHWEAM-FEPSSYGF---RPGRS-----PHDAIARVQAL----T----KQ-------------
SPQGKPPKKQW---VVDADIKGCF-DNIDHQHLLGVI-------------------GNFPARKLIK--
TWLKAGY---I-----E--K---
---G-------------NFN-P----
TE---
---G-GTPQGGVIS-PLLANISLH-GLE-----
-------------------NALGVK-----------------------------WKVRKGRTKSG--------
-IY--------------------------------ATLTQSKRAVIRFADDFII--LC------ESEED----
AK-LAKEEANAFIN-ERGLHLSEEKTSICH----L--ND---------------------------
GFKYLGFRI-
>DS_fid|31973883|locus|VBICanHam112931_1217| [Candidatus Hamiltonella defensa 5AT (Acyrthosiphon pisum)]
GVDNQVI--------------------ND-HKG----------REHLYK---LLSQT-----TSE---
KVYPVKRVYIAK--KN---------------------G---K-------KRPLGIPTILDRCRQAIVKSALE-
--PYWEAK-FEPVSYGF---RPGRS-----AHDAIQKIFCI----ARARGTR---------------------
-HW---VLDADIKGAF-DNIDHNFLIKKI-------------------GGFPERNMIK--QWLQAGV---L--
---E--H--
------------------------------------G-------------NYI-P----NV------------
----
-----------------------------A-GTPQGGIIS-PLLANIALH-GME-------------------
-----TLLGIQ-----------------------------------YWKNG---------TP-----------
-----------------------KQGQPYAVVRYADDFVV--FG------KSREE----CE-
TAKIKLQIWLA-QRGLALSEEKTSIKH----L--KE---------------------------GFDFLGFNI-
>DS_cianobacteriafid|115419880|locus|VBICylSta108647_6126| [Cylindrospermum stagnale PCC 7417]
GIDKVVV--------------------KT-TAA----------RGQLVN---KLTDY-----SPW---
KSSPARRIYIPK--AN---------------------G---K-------KRPLGIPVIQDRAIQAMVKNALE-
--PEWEAT-FERSSYGF---RPGRS-----PHDAIESIYNL----ARPNKRK---------------------
-KW---VVDADIQGCF-DNISHNFLLELL-------------------TGFPARELIK--QWLLAGY---M--
---E--A--
------------------------------------G-------------SWH-P----TD------------
----
-----------------------------A-GTPQGSVVS-PLLANIALH-GME-------------------

-----SALGVK-----------------------------------YNKDG---------EL-----------
-----------------------RAAR--ALVRYADDFVV--FC------ETQED----TK-
NVIQILNYWMQ-VRGLTLSLEKTKISH----L--TE---------------------------GFDFLGFNI-
>DS_cianobacteriafid|115422190|locus|VBICylSta108647_6985|
[Cylindrospermum stagnale PCC 7417]
GVDKLLV-----------------------KTP---EARG--FLVDSLR---KFIP--------W---
KPLPAKRVYIPK--SN---------------------G---K-------KRPLGIQTIIDRCLQAIVKNALE-
--PFWEFH-FELSSYGF---RPGRS-----THDAISKIYMI----VRPNKKK---------------------
-KW---VLDADIKGCF-DNISRNFLMKTI-------------------GNFPARKLID--QWLKAGY---M--
---E--E--
------------------------------------G-------------KFS-E----TL------------
----
-----------------------------T-GIPQGAIIS-PLLANIALH-GME-------------------
-----DALGVK-----------------------------------YNRRG---------EI-----------
-------------------------VSRRAVVRYADDFAI--FC------ETKED----AE-
QAQIDISEWLK-SRGLELSKEKTRIVH----L--NE---------------------------GFCFLGFNI-
>DS_cianobacteriafid|115603860|locus|VBIRivSp77222_4388| [Rivularia sp.
PCC 7116]
GVDKLLV-----------------------LTP---GARG--TLVDILT-----RC------PPW---
KPLPVKRVYIRK--SN---------------------G---K-------QRPLGIPCVIDRCLQAIVKNALE-
--PYWEAQ-FERTSYGF---RPGRG-----VHDAIERIHSM---------SK-----------------
ANSTKSW---VVDADIEGCF-DNIAHSPLLKTI-------------------GNFPAKKLIQ--QWLKAGY--
-V-----D--K--
--G-------------VFN-D----TE--------

---------------------------------T-GVPQGGIIS-PLLANIALH-GME---------------
---------SALGIR-----------------------------------YDKHG---------HT-------
-----------------------------IGNRGIVRYADDLVV--FC------KTQED----AA-
CVVETLSHWMK-SKGLALSKAKTNIVH----L--SE---------------------------GFNFLSFNI-
>DS_clostridiafid|61451056|locus|VBISulAci142080_0653| [Sulfobacillus
acidophilus DSM 10332]
GADGMTK--------------------RR-WEA-----QQAEELERLRT---ELLT------DTY---
RPHPARRIYIPK---P--------------------NG---K-------QRPLGIPCLRDRVVQRAMLMAMD-
--PIWESD-FRWMSYGF---RPGRS-----VHHAVRSVKLA----LTDTVQG-------------------
TAGRW---VIEGDLASYF-DTVHHRLLMKAV--KR--R-IA---------DRR-FLRVLW--RMLKAGL---
I-----D--H---
---------------------------------------G-------------LFRST----HE---------
----
----------------------------------GVPQGGVLS-----------KVD----------------
--------PIV--

---KTDF------
>DS_fid|61475875|locus|VBIVibSp220376_1845| [Vibrio sp. EJY3]
----MTK--------------------HH-LQG---KLGD--YLRKLKL---ELQS------GNY---
QPMPARRIYIPK---A-N-------------------G---K-------QCPLGIPTLRDRIVQRAILMAME-
--PIWEND-FHSLSYGF---RPERS-----VHHAIHTVRLQ----L----AD--------STD-TR-------
GRW---VIEGDLSSYF-DTVHHRLLIKCV--RK--R-IS---------CNG-FLDLLW--RFIKSGH---V--
---E--R--
------------------------------------N---------L---FC--A----TQ------------
----
-----------------------------Q-GVPQGGVIS-PFLSNIMLN-EFDQYLHQRHLSKKARKD----
-----RWY-LNNSI-------------------KIGRRSAIENNWQWQ-PA----------V-----------
--------------------------A---YCRYADDFIL--IV-----QGTKQD----AE-
NIRNESRQFLEGKLKLTLNMEKTHITH----V--ND---------------------------GFVFLGHRI-

>DS_E.c.I4/AB024946/48555..50824/Escherichia coli/Bacterial A
GVDGVNK--------------------TM-LQA---RLAV--ELQILRD---ELLS------GHY---
QPLPARRVYIPK--SN---------------------G---K-------LRPLGIPALRDRIVQRAMLMAME-
--PIWESD-FHTLSYGF---RPERS-----VHHAIRTVKLQ----LTDCGET-------------R-------
GRW---VIEGDLSSYF-DTVHHRLLMKAV--RR--R-IS---------DAR-FMTLLW--KTIKAGH---I--
------D--
-----------------------------------VG-------------LFRAA----SE------------
----
-------------------------------GVPQGGVIS-PLLSNIMLN-EFD-------------------
-----QYLHER--------------------YLSGKARKDRWYWNNSIQRG--------RST-----------
-----------------AVRENWQWKPAVAYCRYADDFVL--IV-----KGTKAQ----AE-
AIREECRGVLEGSLKLRLNMDKTKITH----V--ND---------------------------GFIFLGHRI-
>DS_fid|20797198|locus|VBIAgrRad129173_0726| [Agrobacterium radiobacter
K84]
GIDGMDK--------------------QR-LQV---KLDQ--HLDDLRT---SLLE------ESY---
RPQPVKRIYIPK--SN---------------------G---K-------LRPLGIPTLTDRIVQRAMLMAME-
--PIWESD-FHRLSYGF---RPERS-----VHHAVRTVRIQ----L----QD-------------GADTTR--
GRW---IIEGDLASYF-DTVHHRLLLKCV--RR--R-VQ---------DGR-FVDLLW--RFLKAGH---I--
------D--
-----------------------------------RG-------------LFTAS----SE------------
----
-------------------------------GVPQGGVLS-PLLSNIMLH-EFD-------------------
-----AWLEAK----------YLSDKARKDRWAWNFGIKQGRPITVRESRQ---------WK-----------
--------------------------PAVAYCRYADDFVV--IV-----KGTKAQ----AE-
EIREECRAFLEGELKLTLNMEKTHVTH----V--ND---------------------------GFVFLGHRI-
>DS_fid|18678173|locus|VBIShiBoy33460_0060| [Shigella boydii Sb227]
------------------------------LCQ----------VFGVHR-------------SSY--------
-RYWKNR---P---------------------------------EKPDGRRAV-------LRSQVLE------
----LHGISHGS---AGARSI----ATMATRRGYQM--------------------------------GRW--
--LAGRLMKEL-GLVSCQQPTHRYKRGGHEHVAM---------PKW-VILYCR--RWMEAPM---Q-----
SCEN---
---------------------------------G-------------ELITR----TR---------------
----
----------------------------GTPQGGVIS-PLLANLFLHYAFD----------------------
--LWM-ER------------------------------------EYRG-------------------------
-------------------------VPFERYADDIVV--HC------SRMSD----AT-RLKNRLSERFS-
EVGLVLNAGKTNIAY----I--DT-------------------FKRRNVATSFTFLGYDF-
>DS_fid|47225904|locus|VBIMarMed159599_0649| [Marinomonas mediterranea
MMB1]
GCDGQTM--------------------KQ-FDN---NRDR--NLYKIWN---RLCS------GSY---
LPPPVREKRIPK---A------------------D--G---S-------DRILGIPTVSDRIAQGAVKIYLE-
--TRLDKL-FHNSSFGY---RPNRS-----AHMALTQCERN----C----RF---------------------
NSW---VLEVDIKAFF-DHVDHDLVVKAL--EH--H-DM---------PRW-VVLYCR--RWMQAPM---S--
-DSS--K--
------------------------------------T---------D---ILT-Q----RT------------
----
-----------------------------R-GTPQGGVIS-PILANLFLHYAFD-------------------
-----RWM-AK-----------------------------------QR-RY----------V-----------
--------------------------P---FERYADDIVC--HC------SRMSE----AV-
KLKEAIQRRME-EVGLSINEAKSNVVY----I--DT------F-PR--HN----VKK------VFTFLGYDF-
>DS_Sh_dy_I1/CP000035/29397__31222/Shigella Shigella
dysenteriae/Bacterial D
----------------------------M-FDQ---QRDG--NLYKIWN---RLCS------GTW---
FPPPVLEKRIPK---S------------------N--G---K-------ERILGIPTVSDRIAQGAIKLFME-

```
--EKLDPI-FHADSYGY---RPGKS-----AHDALKQCAIR----C----WR---------------------
YSW---ILEVDISAFF-DHVRHDLVLKAL--EH--H-GM---------PKW-VILYCR--RWMEAPM---Q--
-SCE--N------------------------------------------------------------------
------------------------------------G---------E---VIT------RT------------
-------------------------------------------------------------------------
-----------------------------R-GTPQGGVIS-PLLANLFLHYAFD-------------------
-----LWM-ER-----------------------------------EY-RG----------V-----------
--------------------------P---FERYADDIVV--HC------SRMSD----AT-
RLKNRLSERFS-EVGLVLNAGKTNTAY----I--DT------F-KR--RN----VAT------SFTFLGYDF-
>DS_UMB_I1/AY075117/120__2136/uncultured_marine_bacterium
GVDHVSM--------------------EA-IAS---NPRK--YLYPLWN---RLSS------GSY---
FPPPVKLVPIPK---G-----------------D--G---K-------ERMLGIPTIIDRVAQEVIKAELE-
--VIVEPR-FHPSSFGY---RPHKS-----AHEALEQCAKN----S----WE---------------------
RWY---VVDLDIKGFF-DNIDHEKMMGIL--RK--H-TN---------KKH-ILLYCD--RWLKTPM---Q--
-D-R--V------------------------------------------------------------------
------------------------------------G---------G---VQ--A----RM------------
-------------------------------------------------------------------------
-----------------------------K-GTPQGGVIS-PLLANLYLHEAFD-------------------
-----QWI-ST-----------------------------------TQ-PR----------I-----------
--------------------------V---FERYADDIVI--HT------RSMEQ----SH-
FILDKLKARLK-SYSLELHPDKTKIVY----C--YR------T-ARFHKE----GKEIPV---SFDFLGFTF-
>DS_Bacteroidetesfid|46905862|locus|VBICelAlg158510_0236| [Cellulophaga
algicola DSM 14237]
GIDHQTL--------------------SE-FDS---VRSK--ELYKVWN---RLAS------GSY---
FAPAVKRVNIPK---A-----------------G--G---K-------TRPLGIPTVSDRIAQQVVKQYLE-
--PRLESI-FSENSYGY---RPNRS-----AHSAIEVVRRN----V----LR---------------------
YSW---VIDLDIQEFF-ENVDHGLLLKAL--ER--H-VS---------EKW-VLLYIK--RWLEAPV---I--
-L-E--D------------------------------------------------------------------
------------------------------------G---------T---VKI-S----TG------------
-------------------------------------------------------------------------
-----------------------------K-GTPQGGVIS-PLLSNLYMHYCVD-------------------
-----KWL-EQ-----------------------------------YH-PQ----------V-----------
--------------------------K---MVRYADDLIV--HC------RSYEA----AV-
HTLEVLKERLT-ECGLTAHPEKTKIVY----C--KK------D-GR--DL----KGY-PV---QFDFLGFSF-
>DS_Zu_pr_I2/CP001650_1/3589332__3591217/Zunongwangia
GIDTVSI--------------------EQ-FDE---SLSK--NLYKLWN---RMAS------GSY---
FPPAVKEVEIPK--KD---------------------G---K-------VRKLGIPTISDRIGQMVVKMYLE-
--PRLENV-FNPNSYGY---RPNKS-----AHQALEQVRKN----C----WK---------------------
MDW---VIDLDIKGFF-DNIDHHKMMLAI--EK--H-VP---------ERW-VRLYIA--RWLASPV------
---MTKS------------------------------------------------------------------
------------------------------------G-------------NLVSN----QG------------
-------------------------------------------------------------------------
-----------------------------R-GTPQGGVIS-PLLANLFLHYGLD-------------------
-----KWL-EQ--------------------------------------ND----------------------
--------------------------NTVKFTRYADDVIV--NC------KSQKH----AE-
QTLEAIKSRMH-QIGLELHPEKTKIVY----C--RD-----------------YRRQEKYSNVKFDFLGYSY-
>DS_Ce_ja_I1/CP000934_1/3788874__3790736/Cellvibrio
GADNVCI--------------------DM-FEH---NLEN--ELYKLWN---RMSS------GSY---
MAPPVKRVEMAK---A-----------------D--G---K-------LRPLGIPTVADRVAQMVVKMTLE-
--PEWDSK-FHASSFGY---RPRRS-----AHHAVQAAKIN----C----WK---------------------
YSW---VIDLDIKGFF-DNLNHDQLQKFV--AQ--A-TD---------DPW-CKLYIK--RWITAGV---Q--
-M-P--G------------------------------------------------------------------
------------------------------------G---------E---LH--K----TA------------
-------------------------------------------------------------------------
```

-----------------------------K-GTPQGGVIS-PLLANLYLHKVFD-------------------
-----SWM-QK-----------------------------------YF-PQ----------N-----------
--------------------------P---FERYADDIVC--HC------RTEHE----AE-
QLLSAISRRMQ-RFDLTLHPEKTKIVY----C--GR------R-KI--ER----TKA-Q----SFDFLGFTF-
>DS_D149_(ZP_06641622.1_) Serratia odorifera DSM 4582 416 bp
GVDGMTI--------------------EA-FEH---NLAR--NLYKIWN---RLSS------GCY---
MPPPVKRVEIPK---S------------------D--G---K-------TRPLGIPTVSDRVAQMAVKMILE-
--PQWDPL-FSDSSFGY---RPGKS-----AHDAVAQAKAN----C----WK---------------------
YEW---VIDLDIRGFF-DNLDHALLLKAV--DH--L-HP---------APW-VRLCIV--RWLKAEI---I--
-F-P--D--
------------------------------------G---------H---RH--S----PE------------
----
-----------------------------K-GTPQGGVIS-PLLANLFLHYTQD-------------------
-----KWL-EK-----------------------------------HY-PN----------N-----------
--------------------------S---WERYADDSII--HC------RSRRE----AG-
LLLSQLRERMK-ACGLELHPEKTRIVN----C--HP------L-TR--RK----NDG-HY---SFDFLGFTF-
>DS_Fr_sp_I5/CP000820_1/4042207__4044207/Frankia
GPDGVTV--------------------EQ-FEA---NVKD--RLYVLWN---RMSS------GSY---
FPGPVGAVEIPK---K-G---------------VK--G---G-------ARTLGIPNVVDRVAQTVLKLALE-
--PKVEPV-FHRDSYGY---RPGRS-----QRQALEVCRKR----C----WS---------------------
HDW---VVDLDVRKFF-DTVPWEKLLKAV--AY--H-TD---------QKW-VLMYVE--RCLKAPT---K--
-H-A--D--
------------------------------------G---------T---LQ--E----RT------------
----
-----------------------------M-GTVQGGPFS-PLAANIYLHWGLD-------------------
-----AWM-AR-----------------------------------EF-PT----------V-----------
--------------------------P---FERWADDVVF--HC------VSLEQ----AR-
EVRDAVVARLV-EVGLEAHPDKTRIVY----C--KD------S-NR--GG----DYE-NT---SFTFLSYTF-
>DS_D218_(ZP_06415879.1) Frankia sp. EUN1f   417 bp
GVDGVGL--------------------AG-FES---DLKG--NLYRIWN---RMSS------GSY---
FPPPVKAVEISK---E-H----------------G--A---G-------TRMLGVPTIGDRIAQTVVAARLE-
--GVVEPK-FHPDSYGY---RPRKG-----SLDAVRKCRER----C----WK---------------------
YDW---VIDLDVRKFF-DTVPWDRIIAAV--EA--N-TA---------LPW-VLLYVK--RWLAAPV---R--
-M-P--D--
------------------------------------G---------T---LA--E----RD------------
----
-----------------------------R-GTPQGSAVS-PVLANLFMHYAFD-------------------
-----LWM-VR-----------------------------------EF-PA----------C-----------
--------------------------P---FERYADDAVV--HC------KSLAQ----AR-
FVLDRLRKRME-QVGVSLHPEKTRIVY----C--KD------G-KR--RG----SHE-HT---EFTFLGFTF-
>DS_D154_(ZP_06477373.1_) Frankia (symbiont of Datiscaglomerata) 421 bp
GVDGQSI--------------------DA-FEK---DLKN--NLYRIWN---RMSS------GSY---
FPPPVRAVEIPK---A-H----------------G--G---G-------VRVLGVPTVADRVAQTVVAMTLE-
--PRMEQV-FHDGSYGY---RVGRS-----ALDAVGACRQR----C----WQ---------------------
RDW---VVDLDIQDFF-GSCPHDLIVRAV--EV--N-TD---------QPW-VVLYVR--RWLTAPV---C--
-Y-P--D--
------------------------------------G---------S---LV--T----PD------------
----
-----------------------------R-GTPQGSAVS-PVLANVFLHYALD-------------------
-----LWL-AR-----------------------------------EF-PG----------L-----------
--------------------------P---FERYVDDAVV--HC------ATRRQ----AE-
QVRTAIGRRLE-EVGLRCHPAKTKVVY----C--KD------S-GR--RG----SHE-HT---SFTFLGYTF-
>DS_Actinobacteriafid|162142109|locus|VBIStrFul287543_7023| [Streptomyces
fulvissimus DSM 40593]

--MVAARLE---
RNVEPV-FHSDSFGY---RPGRS-----ALDAVEKCRER----T----WK---------------------
RDW---VVDLDIQKFF-DSVPWSLIVKAV--EA--H-AD---------AVW-VKLYVE--RWLRAPL---Q--
-L-P--D--
------------------------------------G---------T---LQ--R----RD------------
----
-----------------------------R-GTPQGSAVS-PVLANLFLHYAFD-------------------
-----MWI-AR-----------------------------------EF-PD----------I-----------
--------------------------P---FERYVDDAVV--HC------VSERQ----AR-
RLVEAIGNRME-EVGLRLHPAKTRIVY----C--KD------A-NR--RG----AYA-QT---SFTFLGFTF-
>DS_Actinobacteriafid|58753801|locus|VBIMycCan203588_3899| [Mycobacterium
canettii CIPT 140010059]
GVDGVSI--------------------EA-FEA---DLGN--NLYKVWN---RMSS------GSY---
FPPPVRAVEIPK---P-H----------------G--G---G-------TRMLGIPTIADRVAQTVVAEELT-
--SRVEVI-FHDDSHGY---RPGRS-----ALDAVKACRQR----C----WK---------------------
TDW---VIDLDIQKFF-DDVSWDLMLKAV--AA--N-TD---------LPW-VMLYVR--RWLQAPV---A--
-L-P--D--
------------------------------------G---------T---LQ--R----RD------------
----
-----------------------------R-GTPQGSPVS-PVLANLFLHYAFD-------------------
-----TWM-AR-----------------------------------EF-PS----------V-----------
--------------------------R---FERYVDDAVV--HC------VTERQ----AR-
QVLAALQGRMV-EVGLRLHPDKTRIVY----C--KD------G-KR--RG----GYE-HT---SFTFLGFTF-
>DS_B_t_I4/AE015928/3254752 /Bacteroides thetaiotaomicron/Bacterial D
GIDKVTL--------------------ED-YEK---NLRG--NLYKLWN---RMSS------GSY---
FPPSVKLVEIPK---S------------------T--G---G-------KRPLGIPTVSDRVAQMAVVMLIT-
--PSIEPC-FHEDSYAY---RPHRS-----AHDAVGKARER----C----WK---------------------
YAW---VLDMDISKFF-DTIDHELLLKAL--KR--H-TQ---------EKW-VLMYIE--RWLKVPY---E--
-K-S--D--
------------------------------------G---------S---QV--D----RA------------
----
-----------------------------L-GVPQGSVIG-PVLANLFLHYTFD-------------------
-----KWM-EK-----------------------------------NF-PR----------V-----------
--------------------------P---FERYADDTIC--HC------HSLKQ----AE-
YMQAMIQQRFE-CCRLRLNEEKTKIVY----C--KS------S-RQ--KE----CYP-NV---TFDFLGFTF-
>DS_D182_(YP_003997451.1_) Leadbetterella byssophila DSM 17132 410 bp
GIDTQSL--------------------EQ-FEE---RLAD--NLYKIWN---RMTS------GSY---
HPKAVREVQIPK---K------------------S--G---G-------YRGLGIPTVSDRVAQQVVKSYLE-
--PKVEPS-FHQDSYGY---RPNKS-----AHDALAKTVRN----C----GY---------------------
YSW---VVDLDIRGFF-DNIDHELLMKAV--RV--Y-TD---------EKW-IIMYIE--RWLEVGV---V--
-R----E--
------------------------------------G---------K---VH--K----RE------------
----
-----------------------------K-GTPQGGVIS-PLLANIFLHFVFD-------------------
-----KWM-EK-----------------------------------HH-GN----------M-----------
--------------------------P---FERYCDDAII--HC------TTWNQ----AV-
FIKNAVTKRMK-ECKLELNSEKTKIVY----C--KN------S-IH--RE----SNPVPV---SFTFLGHTF-
>DS_Zu_pr_I1/CP001650_1/4279634__4281497/Zunongwangia /Zunongwangia
profunda/Bacterial D
GVDGQSL--------------------QN-FRE---NLSG--NLYKIWN---RMTS------GSY---
FPPVVKEVRITK---K------------------T--G---G-------FRSLGIPTVSDRIAQQVIKSYLE-
--PKVESS-FHQNSYGY---RPRKS-----AHQALEKTVSR----C----GY---------------------
YSW---VVDLDIRGFF-DNIDHTLLMKAV--ER--Y-TK---------EKW-VLMYIG--RWLKTGV---S--

-R----E--
------------------------------------G---------E---IT--D----RI------------
----
-----------------------------K-GTPQGGVIS-PLLANIFLHFAFD-------------------
-----KWM-QI-----------------------------------HH-SN----------M-----------
--------------------------P---FERYCDDAII--HC------TSEKQ----AY-
FIREAVSKRMK-ACKLELNSEKTHIVY----C--KN------H-VH--SE----SHK-NT---SFDFLGYTF-
>DS_W_e_I4/AM999887_1/177114__178961/Wolbachia Wolbachia
endosymbiont/Bacterial D/
GVDEVSI--------------------TK-FEE---NLKD--NLYKLWN---RMSS------GSY---
FPEPVKAVAIPK---D------------------T--G---G-------QRILCVPSVFDRIAQTAATMYLE-
--PLVEPK-FHEDSYGY---RPNKS-----ALDAVYTARKR----C----WK--------------------
NDW---TVDLDISGFF-DNLDHDLALQAI--KK--H-TD---------CKW-VILYVE--RWMKAPI---Q--
-Q-A--D--
------------------------------------G---------S---RV--T----RD------------
----
-----------------------------K-GVPQGGSIS-PIISSIFMHHAFD-------------------
-----MWM-KQ-----------------------------------NY-PT----------V-----------
--------------------------P---FERYVDDAIV--HC------RTKRQ----AG-
FMKVMIEERLA-KCKLKLHPEKTQIVY----S--KD------D-DR--KE----QFP-KQ---SFDFLGYTF-
>DS_D143_(ADO77309.1_) Halanaerobium praevalens DSM 2228 415 bp
GIDKISI--------------------EK-YEK---NLKN--NLYKLWN---RMAS------GTY---
FPKAVKAVEIPK---K------------------N--G---G-------IRVLGVPTVEDRIAQMIVKLSME-
--KIIDPI-FLNDSYGY---RPNRS-----AHDAIKVTRSR----C----WK--------------------
YDW---VLEFDIKGLF-DNINHKLLLKAV--YK--Y-AK---------YKW-EILYIK--RWLANPV---S--
-N-N--N--
------------------------------------K---------I---TK--N----TE------------
----
-----------------------------N-GTPQGGVIS-PLLANLFLHFAFD-------------------
-----KWM-EK-----------------------------------RF-PN----------N-----------
--------------------------K---WCRYADDGII--HC------NSRAE----AI-
FILNCLKERMK-ECKLEIHPGKTKIIY----C--KD------S-NR--KE----NNK-LH---EFTFLGYSF-
>DS_Ma_sp_I3/CP000471/785727 /Magnetococcus sp./Bacterial D
GLDGLTM--------------------EA-FEE---DLKN--QLYRLWN---RMSS------GSY---
FPPPVMRVEIPK---S------------------D--G---G-------VRGLGIPTIGDRIAQAVVKRYLE-
--PLVEPK-FHEDSYGY---RPNRS-----ALDAVRQARQR----C----WR--------------------
DDW---VLDLDISKFF-DKLDHALVMRAV--KR--F-TD---------CKW-VLLYIE--RWLKADV---Q--
-L-Q--D--
------------------------------------E---------T---IL--H----RE------------
----
-----------------------------M-GTPQGGVIS-PLLANIFLHLGFD-------------------
-----QWM-KE-----------------------------------NY-PH----------I-----------
--------------------------H---FERYADDIVV--HC------RSLKQ----LQ-
WIKKRIEQRLK-LCKLSLNDKKTRVVY----C--KD------S-RR--SG----EWT-CQ---SFDFLGYTF-
>DS_D155_(ZP_04698293.1_ _) Rickettsia (endosymbiont of Ixodes
scapularis) 420 bp
GVDEESI--------------------ED-FAL---NLKD--NLYKLWN---RMSS------GTY---
FPPPVKAVEIAK---S------------------D--G---S-------KRLLGIPTVADRIAQAVVKDQLE-
--QLVEPK-FHEDSYGY---RPKKS-----ALDAVGVARQR----C----WQ--------------------
QDW---CIDLDIKNFF-DSLDHQLMMKAI--RF--H-SE---------EKW-IHLYVE--RWLKAPL---Q--
-L-E--S--
------------------------------------G---------E---LI--E----RQ------------
----
-----------------------------S-GTPQGGVAS-PLLANIFMHHAFD-------------------

-----NWM-RR-----------------------------------HY-PE----------V-----------
--------------------------R---FERFADDILA--HC------SSQKQ----AK-
KVLEEIKIRLK-ECGLELHPEKTKIVY----C--KD------D-DR--GG----SYE-YE---SFDFLGYTF-
>DS_Al_me_I4/CP000724/658338 /Alkaliphilus metalliredigens/Bacterial D
GIDEVTL--------------------QE-YEN---NLED--NLYKLWN---SMSS------GSY---
FPQAVRGVEIPK---K------------------N--G---G-------VRVLGVPSIDDRIAQNVMVSELN-
--PKVEPI-FYEDSYGY---RENKS-----AIDAIEVTRKR----C----WE---------------------
YDW---LIEFDIVGLF-DNINHDLLMKAV--KQ--H-TN---------EKW-VILYIE--RTLKVPM---V--
-M-S--D--
------------------------------------G---------I---HV--E----RT------------
----
-----------------------------K-GTPQGGVIS-AVLANLFMHYAFD-------------------
-----HWM-TR-----------------------------------KH-SN----------N-----------
--------------------------P---WVRYADDGLI--HS------HSLKE----AE-
VLLLKLGERFK-DCHLEIHPNKTKIIY----C--KD------D-NR--KQ----NHI-HT---NFDFLGYTF-
>DS_clostridiafid|61052576|locus|VBICloCla155345_0943| [Clostridium
clariflavum DSM 19732]
GVDGVNF--------------------EE-FEK---DLKN--NLYKLWN---RMSS------GSY---
FPKAVRGVEIPK---K------------------N--G---K-------KRLLGIPTIEDRVAQMTVRMSFE-
--QLVEPI-FSSNSYGY---RPNRS-----AIEAVAVTRER----C----WK---------------------
TPW---VLEFDIKGLF-DNIDHELLNRAV--RK--H-TD---------SKW-IILYIE--RFLKAAI---K--
-M-P--D--
------------------------------------G---------T---IQ--Q----RK------------
----
-----------------------------C-GTPQGGVIS-PVLANLFMHYAFD-------------------
-----MWM-KR-----------------------------------EF-PG----------N-----------
--------------------------Q---WVRYADDGII--HC------KTKEE----AE-
YILGKLKERML-KCKLEIHPEKTRIVY----C--RS------D-KN--TE----RHE-HE---SFDFLGYTF-
>DS_clostridiafid|161812148|locus|VBICloPas18034_4761| [Clostridium
pasteurianum BC1]
GTDGVNF--------------------TK-FEE---NLKN--NLYKIWN---RMSS------GCY---
FPASVRGVEITK---K------------------D--G---K-------TRLLGIPTISDRVAQMVVRMNFE-
--PQVEPI-FCDDSYGY---RPNRS-----ALDAVGTARER----C----WE---------------------
MPW---VIDFDIKGLF-DNIDHELMMKAV--CK--H-TD---------NKW-VIMYIE--RFLKAPI---A--
-M-P--D--
------------------------------------G---------T---VQ--E----RN------------
----
-----------------------------A-GTPQGGVIS-PVLADLFMHYAFD-------------------
-----WWM-KQ-----------------------------------KH-PQ----------N-----------
--------------------------P---WERYADDAVI--HC------RTKEE----AK-
VLLVQLKERMT-ECKLEVHPNKTKIVY----C--RS------D-VY--PE----HHE-HE---SFDFLGYTF-
>DS_clostridiafid|42837854|locus|VBICloCf158569_3569| [Clostridium cf.
saccharolyticum K10]

---MVARAYVE---
RAVEPM-FCEDSYGY---RPHKS-----ALDAVEKTRKR----C----WK---------------------
YDY---VIELDVKGLF-DNIDHELLMRVV--RR--H-VK---------EPW-ICLYIE--RWLKSPF---V--
-L-P--D--
------------------------------------G---------S---RI--E----RE------------
----
-----------------------------S-GTPQGGVIS-PVLANMFLHYVFD-------------------
-----MWM-KR-----------------------------------NF-PQ----------A-----------
--------------------------P---FERYADDGVV--HC------RTKEE----AL-
YIKKKLVKRFE-ECKLELHPVKTRIVY----C--KD------K-DR--TK----EEE-LA---EFDFLGYTF-

>DS_D131_(ZP_00738538.1_ _) Bacillus thurigiensis serovar israelensis
ATCC 35646 420 bp
GMDEQSI--------------------EM-YEM---DLKN--NLYKLWN---RMSS------GSY---
FPKPVKAVAIPK---K------------------N--G---G-------TRTLGIPTVEDRVAQMVAKLYFE-
--PNVERL-FYEDSYGY---RPNKS-----AIQAIEATRKR----C----WR---------------------
KDW---VLEFDIKGLF-DNIRHDYLIEMV--KR--H-TN---------QEW-VTLYVQ--RWLITPF---Q--
-M-E--D--
------------------------------------G---------T---LI--E----RT------------
----
-----------------------------A-GTPQGGVIS-PVLANLFLHYTFD-------------------
-----DFM-VK-----------------------------------EF-SS----------I-----------
--------------------------P---WARYADDGIA--HC------TSLKQ----AK-
YLQRRLEERFK-LFGLELNLEKTKIAY----C--KD------D-DR--QL----SYP-NT---SFDFLGYTF-
>DS_D153_(ZP_04856034.1_) Ruminococcus sp. 5_1_39B_FAA 418 bp
GADEQTI--------------------KE-FEE---HLNN--NLYKLWN---RMAS------GSY---
FPKPVRAVAIPK---K------------------N--G---G-------IRILGIPTVEDRIAQMVAKMYFE-
--PLVEPM-FYNDSYGY---RPNKS-----AIQAVGQARER----C----FK---------------------
RDW---ALELDIKGLF-DNIKHGYLMYMV--EK--H-TQ---------IKW-LILYIK--RWLTVPF---I--
-M-S--D--
------------------------------------G---------S---VA--E----RR------------
----
-----------------------------S-GTPQGGVIS-PVLANLFLHYVFD-------------------
-----DFM-TK-----------------------------------AY-PN----------I-----------
--------------------------W---WERYADDGVL--HC------QSYKQ----AA-
FIKQKLEERFQ-QFGLELNKEKTRIVY----C--KD------N-RR--PQ----NYS-CT---QFTFLGYTF-
>DS_fid|186786189|locus|VBIPseSyr242867_5567| [Pseudomonas syringae pv.
actinidiae ICMP 18801]
GIDEQSI--------------------AQ-FEQ---KLQR--NLYKVWN---RMSS------GSY---
FPPPVRQVEIPK---Q------------------S--G---C-------KRKLGIPTVADRVAQTAIKLLIE-
--PSLDCL-FHPDSYGY---RPGKS-----AKQAVEITRRR----C----WN---------------------
INW---VVEFDIKGAF-DHIDHELLLKAV--KH--H-IK---------DEW-ILLYIE--RWLKAPF---E--
-T-A--D--
------------------------------------G---------V---QV--P----RE------------
----
-----------------------------S-GTPQGGVIS-PLLMNLFMHYAFD-------------------
-----AWM-QR-----------------------------------TF-PG----------C-----------
--------------------------P---FARYADDAVV--HC------RSEKQ----AC-
EVMAAIKARLE-VCLLTMHPEKSKIVY----C--KD------S-NR--KA----AYP-TT---QFTFLGFTF-
>DS_R_e_I1/AF261712/2356 R.e.I1/AF261712/2356 4192/Ralstonia
eutropha/Bacterial D
GIDDEAI--------------------AE-FEQ---NLSK--NLYKLWN---RMPS------GSY---
LPPPVKQVEIPK---A------------------S--G---G-------TRKLGVPTVADRVAQTVVKLVIE-
--PGLDAI-FHPDSYGY---RPGRS-----AKQAVAITRER----C----WR---------------------
YDW---VVEFDIKAAF-DQIDHGLLMKAV--RT--H-IR---------EDW-ILLCIE--RWRVAPF---E--
-T-A--D--
------------------------------------G---------V---RV--P----RT------------
----
-----------------------------R-GTPQGGVSS-PILMNLFTHYTFD-------------------
-----RWM-QR-----------------------------------TS-PN----------C-----------
--------------------------P---FARYADDAVV--HC------NSRRQ----AE-
YVMRSIAARLA-ACGLTMHPEKSKIVY----C--RD------SRNR--SE----RHL-HA---SFTFLGFTF-
>DS_fid|86705594|locus|VBIPsePut3905_0289| [Pseudomonas putida ND6]
GIDDETI--------------------AD-FER---NLPK--NLYKLWN---RMSS------GSY---
FPPPVKAVEIPK---A------------------S--G---G-------IRRLGVPTVSDRIAQTVVKLLIE-

```
--PKLDAL-FHPDSYGY---RPGRS-----AKQAIAITRER----C----WR---------------------
YDW---VVEFDIKAAF-DHIDHELLMKAV--RT--H-IK---------EDW-ILLYIE--RWLVAPF---E--
-A-A--D------------------------------------------------------------------
------------------------------------G---------V---RI--Q----RE------------
-------------------------------------------------------------------------
-----------------------------R-GTPQGGVIS-PMLMNLFMHYAFD-------------------
-----AWM-QR-----------------------------------NS-PN----------C-----------
--------------------------P---FARYADDAVV--HC------RSQRQ----AE-
HVMRSIASRLA-VCGLTMHPEKSKIVY----C--KD------S-NR--RA----GYP-HV---SFTFLGFTF-
>DS_Pr_ae_I3/CP001108/2285675__2287507/Prosthecochloris Prosthecochloris
aestuarii/Bacterial D
GVDHETI--------------------EQ-FDR---HLKD--NLYKIWN---RMSS------GSY---
FPPPVKSVPIPK---K------------------S--G---G-------ERVLGIPTVSDRIAQTVVKLMLE-
--PILDPL-FHKNSYGY---RPGRS-----ALDAVAMVRRR----C----WE---------------------
YDW---VVEFDIKGLF-DNIDHDLLMRAL--RK--H-CE---------TPW-ILLYVK--RWLKAPM---Q--
-T-A--T------------------------------------------------------------------
------------------------------------G---------A---IV--E----RS------------
-------------------------------------------------------------------------
-----------------------------S-GTPQGGVVS-PLLANLFLHYAFD-------------------
-----MWV-TQ-----------------------------------NL-RS----------V-----------
--------------------------R---FCRYADDGVI--HC------KSREQ----AE-
LVLHKIRKRFE-QCKLELHPDKTRIAY----C--QD------V-NR--QE----AYP-NV---QFTFLGYTF-
>DS_D115_(YP_911931.1_) Chlorobium phaeobacteroides DSM 266 418 bp
GVDQESI--------------------EA-FEK---NLKG--NLYKLWN---RLSS------GSY---
FPPPVKGVGIPK---K------------------T--G---G-------IRMLGVPTVADRVAQTVGKETLE-
--PLLEPI-FHQDSYGY---RPGRS-----ALDAVGVVRER----C----WK---------------------
YDW---VVEFDISKFF-DTMNHELLMRAV--RK--H-CQ---------IEW-VLLYVE--RWLKAPM---M--
-S-P--E------------------------------------------------------------------
------------------------------------G---------D---LV--E----RT------------
-------------------------------------------------------------------------
-----------------------------K-GTPQGGVIS-PLLANLFLHYAFD-------------------
-----RWV-SE-----------------------------------NL-PG----------V-----------
--------------------------P---FCRYADDGVL--HC------KSKEQ----AV-
LVMKKITKRFE-ACGLRVNPDKTRIVY----C--KD------D-KR--KE----DHP-VT---SFTFLGYTF-
>DS_Pe_ph_I1/CP001110/398581 Pelodictyon phaeoclathratiforme/Bacterial D
GVDRESL--------------------QA-FET---KLKD--NLYKVWN---RLSS------GSY---
FPPPVRGVGIPK---K------------------S--G---G-------VRMLGVPTVADRVAQSVVKMVLE-
--PILEPV-FHEDSYGY---RPGRS-----AHDAIAVVRKR----N----WE---------------------
YDW---VVEFDIKGLF-DNIDHELLMRAL--RK--H-CQ---------TPW-VFLYVE--RWLKAPM---E--
-T-P--E------------------------------------------------------------------
------------------------------------G---------E---LI--E----RT------------
-------------------------------------------------------------------------
-----------------------------K-GTPQGGVVS-PLLANLFLHYAFD-------------------
-----RWV-SE-----------------------------------NL-PG----------V-----------
--------------------------P---FCRYSDDGVL--HC------KSKIQ----AE-
LVKRKIGERFR-ECGLELHPDKTQIVY----C--RD------S-NR--KD----EHP-VN---QFTFLGFTF-
>DS_B_j_I1/BA000040/2212569 Bradyrhizobium japonicum/Bacterial D
GVDGQSL--------------------ED-FAG---DLEN--HRYRLWN---RLVS------GSY---
FPPPVRRVEIPK---A------------------G--G---G-------IRPLGIPTVADRIAQMVVKRCLE-
--PVLDGE-FDPDSYGY---RPGKS-----AHQAIEQARKR----C----WQ---------------------
HDW---VVDLDNKSFF-DTIDHELLMRAV--YR--H-TK---------ADW-IRLYIE--RWLKAPV---E--
-M-P--D------------------------------------------------------------------
------------------------------------G---------S---VR--A----RT------------
-------------------------------------------------------------------------
```

-----------------------------T-GRSQGGVVS-PILANLFLHYVFD-------------------
-----VWM-KG-----------------------------------SY-PH----------I-----------
--------------------------P---FERYADDIIC--HC------RTRQE----AE-
ELKSALERRFA-DCHLLLHPEKTKVVY----C--AD------S-NR--RR----SYP-QI---HFDFLGFSF-
>DS_Pa_de_I1/CP000491/19065 Paracoccus denitrificans/Bacterial D
GVDGQTL--------------------ES-FGE---RLGP--NLYKLWN---RMSS------GSY---
MPSSVRRVMIPK---A------------------D--G---G-------QRPLGIPTVTDRIAQEVVRLYLE-
--PLVEPV-FHRDSYGY---RPERS-----AIDAIRKARQR----C----WR---------------------
YDW---VLDMDIKGFF-DTIDHELLLKAV--RH--H-TD---------CRW-VLLYIE--RWLKAPV---R--
-M-E--D--
------------------------------------G---------S---LV--P----QE------------
----
-----------------------------R-GTPQGGVIS-PLLANLFLHYAFD-------------------
-----RWL-DR-----------------------------------EN-PQ----------V-----------
--------------------------P---FERYADDIIC--HC------RTEDE----AR-
RLWQQVENRLA-GCGLTLHPQKTKIVY----C--KD------T-NR--KG----SFP-TV---AFDFLGYRF-
>DS_Ag_ra_I1/CP000629_1/1749091__1750949/Agrobacterium Agrobacterium radiobacter/Bacterial D
GIDGQTI--------------------AD-FEA---DLRN--NLYKLWN---RLAS------GSY---
FPPPVRRVDIPK---S------------------D--G---K-------TRPLGIPTVADRVAQMVVKRHLE-
--PVVEPE-FHPDSYGY---RPGKS-----ALDAISVARQR----C----WR---------------------
YNW---VLDLDIKAFF-DSIEPDLLMRAV--RK--H-TD---------CPW-VLLYIE--RWLKAPV---Q--
-M-P--D--
------------------------------------G---------N---LV--A----RE------------
----
-----------------------------R-GTPQGGVIS-PLLASLFLHYAFD-------------------
-----MWM-CR-----------------------------------NF-PD----------I-----------
--------------------------P---FERYADDAIC--HC------RSEDQ----AM-
ALQNALDARFT-DCGLTLHPDKTKIVY----C--RD------E-SR--RG----THP-VY---KFDFLGYTF-
>DS_cianobacteriafid|115665938|locus|VBIChaMin231992_1582| [Chamaesiphon minutus PCC 6605]
GVDGQTI--------------------EK-FEE---NLSD--NLYKLWN---RMTS------GSY---
FPSPVLRVEIPK---G------------------D--G---R-------MRPLGIPTVSDRVAQMVAKDLLE-
--PELEKH-FHPDSYGY---RPGKS-----ALDAVGMARKR----C----WK---------------------
SNW---VLELDIKGFF-DNIDHELMMRAV--RV--H-TE---------EKW-VILYIE--RWLKSPI---Q--
-M-P--D--
------------------------------------G---------T---KQ--L----PD------------
----
-----------------------------K-GLPQGGVAS-PLLANLFLHYAFD-------------------
-----KWM-ER-----------------------------------KN-PD----------I-----------
--------------------------Q---FERYADDAVC--HC------KSEAQ----AQ-
KLKQDLNERMK-EVGLELHPEKTNIVY----C--KD------D-DR--RE----EYP-LT---SFDFLGYTF-
>DS_fid|190234795|locus|VBISalEnt166060_0668| [Salmonella enterica subsp. enterica serovar Newport str. USMARCS3124.1]
GVDGQTI--------------------ET-FEG---NLSG--NLYKLWN---RMAS------GSY---
MPPPVRRVEIPK---A------------------T--G---G-------TRPLGIPTVADRIAQMVVKDVLE-
--PILEPH-FHNDSYGY---RPHKS-----AHDALRAARHR----C----WR---------------------
SNW---VLDVDIKGFF-DNIDHDLLMKAV--CK--H-TR---------CKW-TELYIR--RWLTAPV---Q--
-M-P--D--
------------------------------------G---------T---LH--A----RD------------
----
-----------------------------R-GTPQGGVIS-PLLANLFLHYAFD-------------------
-----LWM-TR-----------------------------------QY-PD----------R-----------

--------------------------P---FERYADDIVI--HC------NSQAQ----AI-
VLRNKLEHRLA-ECKLELSQSKTKIVY----C--KD------G-KR--RE----NYP-DI---SFDFLGYTF-
>DS_D172_(YP_003750926.1_) Ralstonia solanacearum 412 bp
GVDGQSI--------------------EE-FEQ---DLSG--NLYKLWN---RLAS------GSY---
MPPAVRCVEIPK---A------------------T--G---G-------TRPLGIPTVADRIGQMVVKDALE-
--PILEPC-FHHDSYGY---RPNKS-----AHDALAVARQR----C----WR---------------------
AAW---VLDVDIKGFF-DNIDHALLMKAV--RK--H-ID---------CRW-ITLYIE--RWLTAPV---Q--
-L-P--D--
------------------------------------G---------T---SQ--A----RN------------
----
-----------------------------K-GTPQGGVIS-PLLANLFLHYVFD-------------------
-----MWM-VR-----------------------------------NF-PA----------N-----------
--------------------------G---FERYADDVVI--HS------TSLKQ----VT-
MLRAQLTERLA-DCKLEMSPGKTKIVY----C--KD------K-RR--KG----GYP-EI---SFDFLGYTF-
>DS_fid|186035780|locus|VBIPhoTem255998_1848| [Photorhabdus temperata subsp. temperata M1021]
GIDGQSI--------------------EE-FEK---NLAG--NLYKLWN---RMAS------GSY---
MPPAVRRVEIPK---A------------------T--G---G-------TRPLGIPTVADRIAQMVVKDMLE-
--PILEPQ-FHVDSYGY---RPHKS-----AHDALRVARQR----C----WR---------------------
TDW---VLDVDIKGFF-DNIDHELLMRAV--RR--H-TD---------CRW-VLLYIE--RWLTASV---H--
-M-P--D--
------------------------------------G---------T---VQ--I----RD------------
----
-----------------------------K-GTPQGGVIS-PLLANLFLHYAFD-------------------
-----MWM-KR-----------------------------------EF-PI----------V-----------
--------------------------R---FERYADDIVI--HC------KSHAQ----AM-
MLRGKLRKRLA-ECKLEMSPGKTKVVY----C--KD------R-ER--TE----AYP-EI---SFDFLGYTF-
>DS_Chlorobifid|23040926|locus|VBIProAes37017_0657| [Prosthecochloris aestuarii DSM 271]
GVDGQSI--------------------AE-FDE---AMEN--NLYKLWN---RLAS------GSY---
MPPPVKRVEIPK---A------------------D--G---G-------LRPLGVPTVADRIAQTVVKQVLE-
--PEMERH-FHPDSYGY---RPGKS-----AHQAVGEARKR----C----WR---------------------
NDW---VVDLDIRGFF-DAIDHELLMRAL--HS--H-TQ---------ERW-VLLYIE--RWLKAPV---Q--
-L-P--D--
------------------------------------G---------T---LQ--K----RG------------
----
-----------------------------A-GTPQGGVIS-PLLANLMLHYTFD-------------------
-----AWM-QR-----------------------------------MF-PH----------V-----------
--------------------------S---FERYADDGVC--HC------RTREQ----AE-
ELMAALKQRFV-DCKLELHPEKTRIIY----C--KD------D-DR--CG----NYP-VT---SFDFLGFTF-
>DS_D177_(NP_928428.1_) Photorhabdus luminescens subsp. laumondii TTO1 469 bp
GIDGQTL--------------------AG-FDE---NVAD--NLYKLWN---RLAS------GSY---
MPQAVRRVEIPK---A------------------D--G---G-------VRPLGIPAVSDRIAQMVVKQVLE-
--PVLEPL-FHADSYGY---RPGKS-----AHQAIAQARTR----C----WQ---------------------
FDW---VVEIDIKGFF-DNIDHALLLKAV--RH--H-TQ---------ERW-LVMYIE--RWLKAPV---Q--
-M-P--D--
------------------------------------G---------R---VQ--V----RN------------
----
-----------------------------R-GTPQGGVIS-PLISNLFLHYAFD-------------------
-----MWM-KR-----------------------------------QF-PG----------V-----------
--------------------------P---FERYADDVVC--HC------HSQSQ----AE-
ALISKAGQRFA-QCGLELHPQKTRMVY----C--KD------A-DR--RG----NYA-ET---RFDFLGYTF-

>DS_UA_I10/FP565147_1/504827__502990/uncultured_archaeon
uncultured_archaeon /Bacterial D
GVDKKSI--------------------AD-FEK---DLNN--NLYKIRN---RMSS------GSY---
FPPPVRTVGIPK---K------------------S--G---G-------ERLLGIPTVADRVAQTVAKMYLE-
--PLVEPY-FHKDSYGY---RPGKS-----AIQAVGVTRKR----C----WR---------------------
YDW---MLEFDIKGLF-DNINHNLLIRAV--RK--H-TN---------CKW-MLLYID--RWLKAPF---Q--
-R-Q--D--
------------------------------------G---------T---LV--Q----RE------------
----
-----------------------------K-GTPQGGVIS-PLLANLVLHYVFD-------------------
-----KWM-ER-----------------------------------NY-PQ----------V-----------
--------------------------P---FCRYADDGVV--HC------RSEAE----AL-
KLRKTLGARFG-KYNLELHPEKTKIVY----C--KD------D-DR--RD----DYP-NT---SFDFLGFTF-
>DS_D152_(YP_003428936.1_) Bacillus pseudofirmus OF4 414 bp
GIDEQSI--------------------DE-FER---NLKD--NLYKVWN---RMSS------GSY---
IPPAVKAVEIPK---K------------------A--G---G-------IRTLGIPTVADRIAQMTVKLYFE-
--PLVEPF-FHEDSYGY---RPKKS-----AIQAIETTRKR----C----WK---------------------
YNW---VLEFDIKGLF-DNIDHELLMRAV--DK--H-TD---------IEW-VKLYIK--RWLTAPF---Q--
-T-K--E--
------------------------------------G-------------IK--E----RT------------
----
-----------------------------S-GTPQGGVIS-PVLANLFLHYAFD-------------------
-----KWM-AI-----------------------------------NH-PR----------N-----------
--------------------------P---FARYADDAVI--HC------KTEEE----AK-
RVLESLNQRMN-ECKLELHPSKTKIVY----C--KD------A-DR--RE----DHK-NI---TFDFLGYTF-
>DS_clostridiafid|161812455|locus|VBICloPas18034_4908| [Clostridium
pasteurianum BC1]
GVDKESI--------------------ED-FEK---NLKN--NLYKIWN---RMSS------GTY---
FPPPVRAVEIPK---K------------------N--G---G-------IRILGVPTVSDRIAQMVVKIHFE-
--PKVEPI-FHPDSYGY---RPMKS-----AIDAIAIVRKR----C----WR---------------------
YNW---VLEFDIKGLF-DNINHELLMKAV--RK--H-TN---------CTW-ILLYIE--RWLTAPL---Q--
-D-K--D--
------------------------------------G---------S---II--T----RT------------
----
-----------------------------S-GTPQGSVIS-PVLANLFLHYTFD-------------------
-----KWM-EI-----------------------------------NF-PS----------N-----------
--------------------------P---WARYADDAVA--HC------KTKYE----AD-
NLLIKLNQRFK-QCALELHPEKTQIVY----C--KD------D-DR--RG----NYP-IT---KFDFLGYTF-
>DS_WP_014731166 Mesotoga prima 411 bp
GIDEVSL--------------------EE-FEA---DLDN--NLYKIWN---RMTS------GSY---
FPPPVKAIEIEK---K------------------S--G---G-------KRVLGIPTVGDRVAQMVAKIYLN-
--PLVDPY-FHKDSYGY---REGKS-----AIDALEVTRQR----C----WR---------------------
YDW---VLEFDIKGLF-DNIDHELLMRAV--KK--H-VK---------IPW-LILYIE--RWLKAPF---I--
-Q-A--N--
------------------------------------G---------R---VE--E----RS------------
----
-----------------------------K-GTPQGGVIS-PVLANLFMHYAFD-------------------
-----KWM-ER-----------------------------------TH-PD----------K-----------
--------------------------P---FARYADDGVI--HC------RTLEE----AR-
LLLESLKERME-ECKLKLHPEKTRIVY----C--KD------D-KR--KG----EYP-NT---SFDFLGYTF-
>DS_M_a_I53/AE011185/2451 Methanosarcina acetivorans/Bacterial D
GVDDENI--------------------AA-FES---DLTN--NLYKIWN---RMSS------GCY---
FPPSVKAIEIPK---K------------------S--G---G-------TRILGIPTVLDRVAQMVTKIYLE-
--PQLEPL-FHPDSYGY---RPGKS-----AADALAATRKR----C----WR---------------------

YNW---LLEFDIKGLF-DNINHDLLMKQV--SM--H-TD---------KPW-IILYIQ--RWLKAPF---Q--
-M-A--D---
------------------------------------G---------T---VN--E----RT-----------
----
-----------------------------K-GTPQGGVVS-PLLANLFLHYAFD------------------
-----QWM-DS----------------------------------HH-RY----------N-----------
--------------------------P---FERYADDSVI--HC------RSREE----AE-
RLWIELDKRLS-EFGLELHPSKTRIVY----C--KD------D-DR--QG----DYP-ET---KFDFLGYTF-
>DS_clostridiafid|61457293|locus|VBISulAci142080_3722| [Sulfobacillus acidophilus DSM 10332]
GVDGESL--------------------RR-FEE---DLKN--NLYKIWN---RMSS------GSY---
FPPPVKAVEIPK---K------------------S--G---G-------VRILGVPTVADRIAQMVVKLTFE-
--PLVEPI-FHPDSYGY---RPGRS-----AHDALAQTRQR----C----WR--------------------
YDW---VLEFDIKGLF-DNIPHDLLMKAV--RQ--H-TD---------NPW-GLLYIE--RWLVAPL---Q--
-R-A--D---
------------------------------------G---------S---QE--P----RT-----------
----
-----------------------------C-GTPQGSVIS-PVLANLFLHYAFD------------------
-----VWM-SR----------------------------------RH-AD----------K-----------
--------------------------P---FERYADDAVV--HC------RSYAL----AA-
ALKEDLARRLA-GCGLEWHPTKTRIVY----C--QD------D-DR--RD----TYP-ET---SFDFLGYTF-
>DS_clostridiafid|21725011|locus|VBIDesAce42372_4086| [Desulfotomaculum acetoxidans DSM 771]
GIDDESL--------------------EA-FEA---NLKN--NLYKIWN---RMSS------GSY---
FPPPVKAVEIPK---K------------------T--G---G-------KRILGVPTVADRVAQMVAKIYFE-
--PLVEPH-FHPDSYGY---RPGKS-----AVDALAVTRQR----C----WK--------------------
YDW---VLEFDIKGLF-DNINHDLLMKAV--RK--H-TD---------NPW-VILYIQ--RWLKAPF---Q--
-M-P--D---
------------------------------------G---------M---LK--E----RT-----------
----
-----------------------------K-GTPQGGVIS-PVLANLFLHYAYD------------------
-----VWM-AR----------------------------------NH-PD----------K-----------
--------------------------P---FARYADDSVA--HC------RSKKD----AE-
KLHDSLKERFA-ECELELHPDKTRIVY----C--KD------D-DR--RG----EHQ-ET---KFDFLGYTF-
>DS_E_c_I9/CP000946_1/2411599__2413485/Escherichia Escherichia coli/Bacterial D
GVDNQTL--------------------KD-FER---DLKG--NLYKIWN---RLSS------GSW---
MPPPVRAVEIPK---K------------------D--G---S-------KRLLGIPTVSDRIAQMTVLVTFE-
--PLVERY-FLNDSYGY---RHGKS-----ALDAIAVTRKR----C----WQ--------------------
YDW---YLEFDIKGLF-DNIPHDLLLRAV--DK--H-CA---------DKW-VRLSIR--RWLTAPV---Q--
-M-P--D---
------------------------------------G---------T---LK--E----RN-----------
----
-----------------------------K-GTPQGGVIS-PVLANLFLHYVFD------------------
-----KWL-SL----------------------------------LY-PE----------I-----------
--------------------------P---WCRYADDGLI--HC------GSKQQ----AE-
ELLNKLAKPFQ-ECGLELHPEKTKIVY----C--KD------S-ER--QA----NHE-TV---QFNFLGYTF-
>DS_Le_pn_I3/CP000675_2/2801059__2799175/Legionella Legionella pneumophila/Bacterial D
GIDNQSI--------------------DE-FSQ---DLKG--NLYKLWN---RMSS------GSY---
FPPAVKEVAIPK---K------------------Q--G---G-------VRKLGIPTVADRIAQMTVKLMME-
--PLLEPH-FLDDSYGY---RPNKS-----ALDAVGVTRKR----C----WE--------------------
YDW---VVEFDIKGLF-DNLSHELLMKAV--KH--H-IS---------DRW-ILLYVE--RWLTAPI---Q--
-D-Q--H---

```
------------------------------------G---------G---CL--P----RT------------
-------------------------------------------------------------------------
-----------------------------A-GTPQGGVIS-PLLSNLFLHYAFD-------------------
-----HWM-TK-----------------------------------HH-PD----------N-----------
--------------------------P---WCRYADDGLA--HC------RTEKE----AE-
QMLKEIDKRFK-SLGLEIHPDKTKIVY----C--KD------G-AR--KG----KYK-NK---SFDFLGYTF-
>DS_fid|108024353|locus|VBILegPne122099_3574| [Legionella pneumophila
subsp. pneumophila]
GVDEESL--------------------ED-FAK---DLKN--NLYKLWN---RMSS------GSY---
FPPAVKAVPIPK---K------------------S--G---G-------ERMLGIPTVADRIAQMVVKLVFE-
--PIVEPH-FHPDSYGY---RPNKS-----ALDAVGITRQR----C----WQ---------------------
YDW---VLEYDIRGLF-DNIDHQLLMKAV--RK--H-TD---------SKW-VLLYIE--RWLVTPM---Q--
-L-P--D------------------------------------------------------------------
------------------------------------G---------T---LQ--E----KV------------
-------------------------------------------------------------------------
-----------------------------K-GVMQGGVIS-PVLSNLFLHYVFD-------------------
-----SWM-VR-----------------------------------NA-TK----------M-----------
--------------------------S---WCRYADDGLV--HC------KTKFE----AQ-
QIRRRLEARFI-ECGLEMHPDKTKIVY----C--KD------S-NR--RL----NYQ-NT---SFDFLGFTF-
>DS_Gfid|23549090|locus|VBIShePut135485_0278| [Shewanella putrefaciens
CN32]
-------------------------------------------------------------------------
-----------------------------------------------------------MVVKLSFE---
PLVEPH-FLNDSYGY---RPNRS-----AIDAVGVTRKR----C----WY---------------------
QDW---VLEFDIKGLF-DNISHELLMKAV--RK--H-TD---------CKW-LLLYIE--RWLKAPM---V--
-K-D--N------------------------------------------------------------------
------------------------------------N---------E---VI--E----RN------------
-------------------------------------------------------------------------
-----------------------------M-GTPQGGVIS-PVLANLFLHYVFD-------------------
-----KWM-HK-----------------------------------NH-PG----------V-----------
--------------------------K---WCRYADDGLV--HC------NSEEQ----AQ-
KMRAELEKRFK-DCGLEMHPTKTKIVY----C--KD------G-TR--KG----QYE-NT---AFDFLGYTF-
>DS_E_c_I2/X77508/518 Escherichia coli/Bacterial D
GIDKQSL--------------------AD-FDK---RLVD--NLYKIWN---RLSS------GSY---
FPPAVKAVAIPK---K------------------L--G---G-------ERILGIPTVSDRIAQTVVKLAFE-
--PQVEPH-FLADSYGY---RPNKS-----ALDAIGVTRKR----C----WY---------------------
YDW---VLEFDIKGLF-DNIPHELIMKAV--DK--H-NP---------ARW-VKLYIQ--RWLTAPM---V--
-M-S--D------------------------------------------------------------------
------------------------------------G---------E---VR--A----RT------------
-------------------------------------------------------------------------
-----------------------------M-GTPQGGVIS-PLLANLFMHYVFD-------------------
-----KWL-AK-----------------------------------YY-PK----------V-----------
--------------------------P---WYRYADDGIL--HC------HSEAE----AT-
EMREVLRKRFS-ECGLEMHPEKTRVIY----C--KD------G-SR--KG----DYE-HT---MFDFLGYTF-
>DS_Dh_re_I1/CP001734_1/751437__753288/Desulfohalobium Desulfohalobium
retbaense/Bacterial D
GVDRQSL--------------------ED-FEK---DLKN--NLYKLWN---RMSS------GSY---
MPPLVKGVEIPK---K------------------S--G---G-------TRLLGVPAVSDRIAQMAARLEFE-
--AQVEPH-FLPDSYGY---RPKKS-----ARQAIDVTRKR----C----WD---------------------
QDW---VLEFDIKGLF-DNIDHDLLMKAV--EK--H-TD---------NPW-VRLYIR--RWLKAPM---Q--
-L-E--S------------------------------------------------------------------
------------------------------------G---------E---LV--D----RD------------
-------------------------------------------------------------------------
-----------------------------K-GTPQGGVIS-PVLANLFLHYVFD-------------------
```

-----AWL-TK-----------------------------------HY-PR----------V-----------
--------------------------K---WCRYADDGLV--HC------ESEAQ----AR-
FLLEALRQRFK-ECGLELHPEKTKIVY----C--KD------G-RR--TG----DYP-QT---SFDFLGYTF-
>DS_Gfid|87202399|locus|VBIMetAlc68050_2225| [Methylomicrobium
alcaliphilum]
GVDRQSL--------------------AD-FER---NLKD--NLYKLWN---RLSS------GSY---
FPPPVKAVAIPK---K------------------A--G---G-------ERILGIPTVSDRIAQMVVKLEFE-
--PQVEPH-FLPDSYGY---RPNKS-----ALDAVGVTRER----C----WR---------------------
YDW---VLEFDIKGLF-DNIPHDLLLKAV--YK--H-TD---------TAW-VRLYIE--RWLTVPM---Q--
-M-P--N--
------------------------------------G---------E---LS--S----RG------------
----
-----------------------------K-GTPQGGVVS-PVLSNLFLHYVFD-------------------
-----KWL-QK-----------------------------------HY-SD----------T-----------
--------------------------P---WCRYADDGLV--HC------RSEAE----AK-
HMLEALKQRFQ-SCGLELHPVKTKIVY----C--KD------G-SR--KG----RYK-HT---SFDFLGYTF-
>DS_Vi_vu_I1/GQ292873_1/3620__5549/Vibrio Vibrio vulnificus/Bacterial D
GVDGVTI--------------------ED-FEK---DLKN--NLYKIWN---RMSS------GSY---
FPTPVAAVSIPK---K------------------S--G---G-------ERVLGIPTVSDRVAQTVVRDKLE-
--IMLEHH-FLDDSYGY---RVGKS-----AHDAIEVTRRR----C----WQ---------------------
YDW---VLEFDIKGLF-DNIRHDLLMKAV--KK--H-VQLAEESQSRDYQW-ITLYIE--RWLVAPL---Q--
-K-A--D--
------------------------------------G---------T---QT--E----RE------------
----
-----------------------------L-GTPQGGVVS-PVLANLFLHYVFD-------------------
-----KWL-EK-----------------------------------NY-PD----------N-----------
--------------------------P---WCRYADDGLV--HA------RTKPK----AE-
KLRDELAKRFK-ECGLEMHPIKTKIVY----C--KD------D-IR--RG----SGK-HIEHKQFDFLGYTF-
>DS_D216_(ZP_08074908__) Methylocystis sp. ATCC 49242 417 bp
GVDGVTI--------------------EQ-FEK---DLKG--NLYKIWN---RMSS------GAY---
FPPPVRAVSIPK---K------------------S--G---G-------QRILGVPTVADRVAQTVVKEIIE-
--PALDAI-FLADSYGY---RPDKS-----ALDAVGVTRER----C----WK---------------------
FDW---VLEFDIKGLF-DNIDHTLLMRAV--RK--H-VA---------CPW-ALLYIE--RWLTAPM---M--
-Q-E--D--
------------------------------------G---------T---LI--E----RT------------
----
-----------------------------R-GTPQGGVVS-PVLANLFMHYTFD-------------------
-----LWM-AR-----------------------------------TF-PH----------L-----------
--------------------------R---WCRYADDGLV--HC------RSERE----AR-
IVWEALASRMA-ECRLELHPTKTKIVY----C--KD------D-RR--KA----NFE-NV---AFDFLGYCF-
>DS_RmInt1_(Y11597.2_) Sinorhizobium meliloti 419 bp
GVDGQTL--------------------EI-FEK---DLAA--NLYKIWN---RMSS------GTY---
FPPPVRAVSIPK---K------------------A--G---G-------ERVLGVPTVSDRIAQMVVKQMIE-
--PDLDSL-FLPDSYGY---RPGKS-----ALDAVGVTRQR----C----WK---------------------
YDW---VLEFDIKGLF-DNLPHDLLLKAV--RK--D-VK---------CNW-ALLYIE--RWLTAP----M--
-E-K--N--
------------------------------------G---------E---VI--E----RS------------
----
-----------------------------R-GTPQGGVVS-PILANLFLHYAFD-------------------
-----LWM-TR-----------------------------------TH-PD----------L-----------
--------------------------P---WCRYADDGLV--HC------QSEQQ----AE-
ALRVELSSRLA-ACGLQMHPTKTKIVY----C--KD------Q-RR--RE----AYP-NV---TFDFLGYQF-
>DS_Afid|115293251|locus|VBIRhiTro150571_4429| [Rhizobium tropici CIAT
899]

GVDGQTI--------------------EQ-FEA---DLKG--NLYKIWN---GMSS------GSY---
FPPPVRAVPIPK---K------------------T--G---G-------QRILGVPTVSDRIAQMVVKRLIE-
--PELDQI-FRPDSYGY---RPGKS-----ALDAVGITRQR----C----WK---------------------
YDW---VLEFDIKGLN-DNLAHDLLLKAV--HK--H-VK---------GQG-ALLYIE--RWLTAP----L--
-E-Q--D--
------------------------------------G---------Q---RI--G----R-------------
----
-----------------------------I-AVPRKGVWS-VRFFQI-CSCTTH-------------------
-----LIS-NR-----------------------------------TY-PD----------L-----------
--------------------------P---WCRYADDGLV--HC------RTEQE----AE-
AVKAALQARLA-ECQLEMHPTKTKIGY----C--KD------P-KR--RG----TYP-NV---SFDFLGYCF-
>DS_RmInt2_(YP_007194308) Sinorhizobium meliloti 419 bp
GVDGQTI--------------------EQ-FEA---DLKG--NLYKIWN---RMSS------GSY---
FPPPVRAVPIPK---K------------------T--G---G-------QRILGVPTVSDRIAQMVVKQLIE-
--PELDQI-FLKDSYGY---RPNKS-----ALDAVGITRQR----C----WK---------------------
YDW---VLEFDIKGLF-DNISHELLLKAV--RK--H-VK---------CKW-ALLYIE--RWLTAP----M--
-E-Q--D--
------------------------------------E---------Q---RI--E----RD------------
----
-----------------------------C-GTPQGGVIS-PILSNLFLHYAFD-------------------
-----LWM-DR-----------------------------------TH-PD----------L-----------
--------------------------P---WCRYADDGLV--HC------RSEQE----AE-
AVKAALQARLA-ECQLEMHPTKTKIVY----C--RD------S-KR--RG----QHP-NV---TFDFLGYCF-
>DS_fid|20872676|locus|VBIAltMac49397_0697| [Alteromonas macleodii str. 'Deep ecotype']
--MRVYY-------------SLY--------
--------------------------------G-------------------------------------
HLLNKA------------R---------LFKDLKRYSKR---------KR-----------------------
---------------GRIDGQSLSAFA--SA--

---TE--------------

---------------------------T-GSPQGGVIS-PLIANIYLD-AFD---------------------
---EEM-KQ--------------------------------------RG---------HR-------------
----------------------------IVRYADDILI--LC------CSRTA----AE-
NAKAQATHILEGKLKLSVNTEKTHITH----S--DD---------------------------GVKFLGVEI-
>DS_Al.or.I2/CP000853/2108190..2110275/Alkaliphilus oremlandii/Bacterial C
GIDGETV-----------------------FNF---HLNLELNIEFLHD---KLKT------NGY---
EPSPVRRVEIQK---P--------------------DG---G-------VRLLGIPTVKDRVVQQAIVNIIE-
--PIFDKT-FHPSSYGY---RPNHS-----QHGAVAKAERF----M----NK------------YG-------
LEH---VVDMDLSKCF-DTLDHEIMMKAV--SE--R-IS---------DGR-VLKLIE--KFLKAGV---M--
---HSDN--
--FS-------RT------------

-----------------------------EVGSPQGGVIS-PLLSNIYLN-QFD-------------------
-----QRM-MS--------------------------------------KG---------IR-----------
------------------------------IVRFADDILI--FA------KDKKT----AG-
NYKAYATQVLENELKLKVNNEKTKLTN----V--NE---------------------------GVEFLGFVI-
>DS_clostridiafid|22670018|locus|VBINatThe92436_0719| [Natranaerobius thermophilus JW/NMWNLF]
GIDQVTV--------------------EA-YGS---NLEE--NLETLHH---DLKI------GAY---
KPQPVRRVKIPK---P--------------------DG---S-------TRPLGIPTVKDRVVQQATLNILQ-
--PIFDPD-FHPSSYGY---RPNRS-----CHKAIAKSEQF----I----NK---------------------

YNLRH-VVDMDLSKCF-DKLNHELIIEEV--AK--K-VS---------DGS-VLKLIK--KFLKSGV---M--
---E--D--
------------------------------------G-------------AIEDT------------------
----
-----------------------------EIGSPQGGVIS-PLLTNIYLD-RFD-------------------
-----KEM-KS--------------------------------------RN---------IR-----------
------------------------------IVRYADDILI--FA------YTPRQ----AK-
RYKDIATEILEDELKLTVNKEKTHITN----D--RK---------------------------GVPYLGVII-
>DS_clostridiafid|38271879|locus|VBITheSp141296_0259| [Thermincola potens JR]
----MTV--------------------EA-FGQ---NLQE--ELRQLHH---ELKT------GIY---
EPQPVLRVEIPK--VD---------------------G---S-------KRPLGIPTVRDRVVQQALLNILQ-
--PIFEPD-FHPSSYGY---RPGRS-----CHQAVAKAEMF----I----NK---------------------
YGLSH-VVDMDLSKCF-DRLNHDLILEGV--NR--K-VS---------DGS-VLKLIK--KFLTAGV---M--
---K--D--
------------------------------------G-------------AWEET------------------
----
-----------------------------DLGSPQGGVIS-PLLTNIYLD-SFD-------------------
-----QEM-KE--------------------------------------RG---------IR-----------
------------------------------MVRYADDILL--FA------ATYQD----AK-
KYQRIATDFLEQELKLTVNREKTHLTD----N--RK---------------------------GVAYLGFVI-
>DS_Pe.th.I1/AP009389/2583061..2585155/Pelotomaculum thermopropionicum/Bacterial C
GIDGETV--------------------EA-FGQ---NLGQ--RLIQLHH---ELKT------GTY---
EPQPVKRVEIPK---P--------------------DG---S-------TRPLGIPTVRDRVVQQALLNILQ-
--PIFEPG-FHPSSYGY---RPGRS-----CHQAVAKAERF----M----NK------------YG-------
LEY---VVDMDLSKCF-DRLDHELILEEV--NR--K-IS---------DGS-VLKLIK--KFLTAGV---M--
---K--D--
------------------------------------G-------------QWD-E----ID------------
----
-----------------------------T-GSPQGGVIS-PLLANIYLD-RFD-------------------
-----QAM-KS--------------------------------------RG---------IR-----------
------------------------------IVRYADDILV--FA------RTRKE----AG-
NYRQVATQILEGELKLEVNKEKTHLTS----V--HE---------------------------GVAYLGFII-
>DS_fid|20854494|locus|VBIAliSal95923_2257| [Aliivibrio salmonicida LFI1238]
GIDGQRI--------------------DD-FTQ---NLEV--ELRKLLL---ELQE------KRY---
QARPVKRVEIAK-DDG---------------------G-----------IRLLGIPTVRDRIVQQCLTNIMT-
--PIFDPN-FHPSSYGY---RVGRS-----CHQAISKATLF----I----RK---------------------
YNK-QHVVDMDLSKCF-DMLDHDLIIKFV--RK--R-IV---------DGS-ILGLIR--QFLKSGV---M--
----
---IG------------

-------------------------------ENWQNSVIGSPLLANIYLD-EFD-------------------
-----QEM-MR--------------------------------------RK---------HR-----------
------------------------------IVRYADDILI--FC------TSKKG----AE-
NALKVASHILEVTLKLKVNERKTHIAH----S--DT---------------------------GIKFLGVEI-
>DS_D.p.I1/CR522871/6124..8213/Desulfotalea psychrophila/Bacterial C
GIDGQSV--------------------KD-FAE---SLDV--NLDRLLT---ELRE------KSY---
QPQPVRRVEIPK--EN---------------------G---G-------IRLLGIPAVRDRVVQQALLDILQ-
--PIFDPD-FHPSSYGY---RPGRS-----CHQAITKATMF----I----RK-------------------
YDRKW---VVDMDLSKCF-DTLDHDLILSSL--SR--R-IK---------DGS-ILGLLK--KILKSGV----
-----MTDE--
--------------------------------------G-------------WQ-------AS----------

----
-------------------------------EVGSPQGGVIS-PLIANIYLD-QFD-----------------
-------QFM-KK--------------------------------------RG---------HR---------
--------------------------------IVRYADDILI--LC------SSKSA----AK-
NALLQASCFLEKGLLLTVNREKTHICH----S--WS---------------------------GVAFLGVSI-
>DS_Bacteroidetesfid|54567245|locus|VBIKroSp190710_0128| [Krokinobacter sp. 4H375]
GVDNVYI--------------------KE-LKS---ILQI--YGKQYVS---HIER------KRY---
QVSPILGVEIPK---S-----------------N---G---K-------KRLLGIPTVVDRVFQQALHQVLQ-
--PLFEPD-FQKHSYGF---RPQRN-----AHQATAESLLN----I----NA-------------G-------
SQD---IVDIDLKSFF-DEVSHCILLELI--YK--K-VQ---------CKA-TMRLLR--SFLRAPI---L--
---I--N--
------------------------------------G---------R---LQ--K----RR------------
----
-----------------------------K-GVPQGSPLS-PLLSNILLN-ELD-------------------
-----KEL-EK--------------------------------------RG----------H-----------
--------------------------R---YVRYADDFSI--YV------KSKVA----AK-
RVGNSIYKYLRDHLQLPINRVKSGVRR----P----------L--------------------DFQVLGFGF-
>DS_Bacteroidetesfid|46881331|locus|VBIBacSal140776_2677| [Bacteroides salanitronis DSM 18170]
GVDGVSI--------------------RE-LRK---VFSE--KKLQLIE---AIKQ------GNY---
QVQPILGIEIPK---G-----------------N---G---K-------TRLLGVPTTTERVLQQALAQTIA-
--PLFEPE-FSNYSYGF---RPHKN-----ARQAVGQSRDY----I----HS-------------G-------
LNH---IVDIDLKNFF-DEVDHCLLLNLI--YQ--K-VK---------CKA-TMQLIR--KWLRAPI---K--
---I--N--
------------------------------------G---------K---LR--K----RR------------
----
-----------------------------K-GVPQGSPLS-PLLSNILLH-QLD-------------------
-----KEM-TR--------------------------------------RG----------H-----------
--------------------------K---FVRYADDFSI--YC------KSHNQ----AK-
ATRVVIEKFLKNKLKLTINEEKSGIRK----P----------I--------------------HFTILGFGF-
>DS_Bacteroidetesfid|87132848|locus|VBIBelBal168934_2118| [Belliella baltica DSM 15883]
--MIY--------
QRVKCPT--

---TLRLIR--KWLRAPI--QI-------
-N---
-------------------------------G-------------KLHRR----RK-----------------
----
--------------------------GVPQGSPLS-PLLSNILLD-LLD------------------------
KEL-ER--------------------------------------RN---------LK----------------
-------------------------YVRYADDFSI--YT------KSKKE----AR-
KVGNEIYLFLKEKLRLPINREKSGIRR-------PS---------------------------NFEMLGHAF-
>DS_Bacteroidetesfid|46912205|locus|VBICelAlg158510_3341| [Cellulophaga algicola DSM 14237]
GIDGMTV--------------------QE-LKA---FIDA--HRSKVVH---QLIS------KSY---
RSQAIKGVAIPK---A-----------------N---G---K-------TRLLGVPTVVDRWLQQAVSQQLV-
--IHFELD-FESESYGF---RPRKN-----LQQAVLKSQEY----I----ND-------------G-------
YQD---LVDIDLKSFF-DEVQHYKLLQLI--YN--K-VK---------CPT-TLWLIR--KWLRAPI---L--
---K--N--
------------------------------------G---------Q---LC--K----RR------------
----
-----------------------------K-GLPQGSPLS-PLLSNIMLD-QLD-------------------

-----KHL-KV-------------------------------------RE----------F-----------
-------------------------R---FIRYADDFSI--YT------KSKAA----AR-
AIGNEVYLFLKEKLDLPVNRAKSGIRR----P---------S--------------------TFKVLGYRF-
>DS_Gfid|186998609|locus|VBIPseSyr250795_4170| [Pseudomonas syringae pv. actinidiae ICMP 19102]
-----------------------------MSQ---------QLTKLWD------------------------
-------------------------------G---G------------------------------------
----FSDHSYGF---RPGRS-----NLDAIRAAKAF----V----VS------------G-------KNW--
-VVDVDIEAFF-DEVSHDRLMTRI--TRDIH-----------DKR-LNKYLG--SNVRADM---I-----L-
-N--
------------------------------G-------------QRQKR----SA-----------------
----
-------------------------GVPQGGPLS-PLLANLYLD-PLD------------------------
KEL-EA-------------------------------------RG---------LS----------------
------------------------FCRYADDLMI--FV------ESERS----AE-
RVLESIVSWIEKHLKLKVNASKSGTGR---PW------------------------------ERAFLGYLI-
>DS_Bacillifid|86663631|locus|VBIStaAur229418_1173| [Staphylococcus aureus subsp. aureus HO 5096 0412]
GIDGMKV-------------------SE-IQG---HFAQ--YFPKIKQ---KLLE------GTY---
KPQAVKKVEIPK---A----------------N---G---K-------KRVLGIPVVRDRVIQQAIKQVIE-
--PSIDRT-FSKHSHGF---RPNRS-----TGTALKECASY----Y----EA------------G-------
YTI---AVDCDLKQCF-DNINHDKLMYLF--ER--H-IK--------DKA-VSTFIR--RRLQVGA---I--
--DL--S---
-----------------------------------G---------E---VA--E----RK------------
----
----------------------------I-GAPQGGVIS-PLLCNIYLH-ELD-------------------
-----KEL-EK-------------------------------------RN----------H-----------
-------------------------R---FVRYADDFVI--FV------KTKRA----GE-
RVMDSIKTFIHKTLKLEVNNDKSKVGS----P---------T--------------------RLKFLS-CL-
>DS_Bacillifid|45364599|locus|VBIStaPse177932_0703| [Staphylococcus pseudintermedius HKU1003]
GIDGMKV-------------------SE-LHA---HFEQ--YFSQITK---KLLD------GSY---
QPQAVRKVQIPK---P----------------N---G---K-------MRVLGIPVARDRVIQQAIRQVIE-
--PGIDRT-FSNHSHGF---RPNRS-----TGTALKQCATY----Y----EE------------G-------
YKI---AVDCDLKQCF-DMLNHDKLMYLF--ER--H-VQ--------DKS-ISTFIR--RSLQVAA---I--
--DL--S---
-----------------------------------G---------E---VA--E----RK------------
----
----------------------------I-GAPQGGVIS-PLLCNIYLH-ELD-------------------
-----KEL-EK-------------------------------------CG----------H-----------
-------------------------R---FVRYADDFVI--FV------RTKRA----GE-
RVMTSVTKFIEKQLKLVVNEEKSRVGA----V---------T--------------------RLKFLN-CL-
>DS_Afid|54139251|locus|VBISinMel152503_4967| [Sinorhizobium meliloti SM11]
GRDGQTV-------------------DM-AEA---KATS--IIGRLRR---ELLN------GKY---
RPGDVRRVWLPK--AG--------------------G---G-------RRGLGIPNIVDRVVQQAVLQVLE-
--PIFEPV-FHDSSHGF---RPKRG-----AHTAIAEASKY----L----KE------------G-------
YQT---IVDLDLASFF-DRVHHQRLLARI--AQ--R-VK--------DQR-IITLIN--LMLKAAV------
---VMPD---
-----------------------------------G-------------TRVAP----QE------------
----
------------------------------GTPQGGPLS-PLLSNIVLD-ELD-------------------
-----REL-AR-------------------------------------RR---------LR-----------

------------------------------FVRYADDSNI--FV------RSERA----GQ-
RVMSSIRDFLERRMRLQVNEEKSGMRT-------PN---------------------------EVHFLGFRF-
>DS_Bacillifid|54204419|locus|VBILacJoh171132_1733| [Lactobacillus johnsonii DPC 6026]
GVDKRTI--------------------YE-IDD---YFKK--HQVEIKQ---SIRA------MKY---
KPQAVRRVYIPK--AN---------------------G---K-------KRPLGIPTVVDRVIQQAISQVLM-
--KIYDPE-FSAYSYGF---RPKRS-----SHDAMEQVLEY----L----DE-------------G-------
YQW---VIDLDIEKYF-DTVNHDKLISTL--RE--Q-IN---------DKT-TLHLIR--SFLKAGI---M--
---E--D--
------------------------------------G-------------LV--K----PN------------
----
-----------------------------KLGVPQGGPLS-PILSNIYLD-KFD-------------------
-----KEL-EE--------------------------------------RG---------LH-----------
------------------------------FVRYADDCNI--FV------KSKMS----AD-
RVMKSATSWLERKLFLKVNATKTKVVR-------PT---------------------------KSNFLGFT--
>DS_M.sp.I1/AF339846/29388..31287/Microscilla sp./Bacterial C
GVDGMQV--------------------KE-LRY---WFSN--NHQKLIE---QLKE------GNY---
RPMTIKGQEIPK---P--------------------GG---G-------VRQLGIPTVQDRLVQQAIAQQLS-
--KRYDPT-FSQYSYGF---RKGRN-----AHQALRQAGAY----V----KE-------------G-------
FNY---VVDLDLEKFF-DKVNHDRLMWLL--GR--R-IS---------DKR-VLKLIG--KFLRSGI---L--
------I--
------------------------------------G-------------GLENQ----RI------------
----
-----------------------------S-GTPQGSPLS-PLLSNIVLD-ELD-------------------
-----KEL-ER--------------------------------------RG---------HR-----------
------------------------------FVRYADDMIL--LV------RSQEA----AE-
RAYSSITSFIENRLLLKVNKDKSRICR-------PY---------------------------QLNFLGHSI-
>DS_Bacteroidetesfid|115236219|locus|VBIEchVie187570_1054| [Echinicola vietnamensis DSM 17526]
GIDGMSV--------------------EE-LRQ---WFSS--HYHEFQS---QIIT------GKY---
RVESVREVQIPK---P--------------------NG---G-------VRILGIPTAKDRLVQQAISQVLS-
--LHYDPT-FSDRSYGF---RPDRG-----AHDALRQAGQE----V----SE-------------G-------
RDW---IVDIDLEKFF-DTVNHDRLMWLL--GT--R-IG---------DKT-LLKLIG--KFLRAGM---L--
---K--D--
------------------------------------G-------------LVSQG----VK------------
----
-------------------------------GMPQGSPLS-PLLSNIILD-ELD-------------------
-----KEL-EV--------------------------------------RG---------HR-----------
------------------------------FVRYADDLIV--MV------KSEPS----AK-
RVLSSLTAFIEQRMLLKVNKSKSKISR-------PY---------------------------ELNFLGHSI-
>DS_E.c.I7/AY785243/414..2383/Escherichia coli/Bacterial C
GIDNMSI--------------------EE-FND---FAKL--HWLGIKQ---QLLN------GSY---
QPLPVKRVMIPK---P-D-----------------G-G-----------ERMLGIPAVIDRVIQQAIAQVIS-
--PYFEPQ-FSPHSYGY---RPHKR-----ASQAVNHVQSC----V----KQ-------------G-------
YKT---AVDIDLSKFF-DEVDHDMLMNRV--SR--K-IK---------DKA-LMRLLG--KYLRAGI---A--
-ERE--T--
------------------------------------G-------------LWF-E----ST------------
----
-----------------------------K-GVPQGGPLS-PLLSNILLD-ELD-------------------
-----KKL-TY--------------------------------------KH---------LK-----------
------------------------------FARYADDIII--LV------KTKSE----GL-
IIQREITAFITKRLKLKVNESKSRVGP-V--S-------------------------------GSKFLGFTF-
>DS_Bacteroidetesfid|42649895|locus|VBILeaBys116579_0020| [Leadbetterella byssophila DSM 17132]

```
GVDGMQI--------------------DN-LRD---YLNT--HWQSLRS---DILS------GTY---
RPQAVRKVEIPK--AS---------------------G---G-------KRMLGIPTVIDRVIQQSISQWLG-
--LKYEGD-FHDNSYGF---RPNRN-----AHQAVSKAQEY----L----NL-------------G-------
YTW---VVELDLEQFF-DQVNHDILMHLL--SK--K-IT---------DHR-VLALIG--KYLRCGI---M--
---D--H------------------------------------------------------------------
------------------------------------G-------------LEQKR----TK------------
-------------------------------------------------------------------------
-------------------------------GTPQGSPLS-PLLSNIILN-ELD-------------------
-----REL-SS--------------------------------------RG---------HR-----------
------------------------------FVRYADDCSI--YT------RSNKS----AT-
RIMGNITSYIESTLKLKVNREKSKVSR-------PS---------------------------QSSLLGFSF-
>DS_Gfid|42345729|locus|VBIGamPro61291_1517| [gamma proteobacterium HdN1]
GIDGMPV--------------------ED-LES---HLRH--HWPTLRQ---SLLD------GTY---
QPKPVKRVEIPK--GD---------------------G---T-------KRALGIPTVIDRFVQQIIAQALS-
--ALWEPH-FHPSSFGF---RPARS-----AQQAVKYVQTL---QR----EK---------------------
YEW---VVDLDLKSFF-DEVNHDRLIARL--KT--R-VE---------DKV-LLRLIN--KFLHAGI------
----NAN------------------------------------------------------------------
------------------------------------G-------------ILLRS----EK------------
-------------------------------------------------------------------------
-------------------------------GVPQGGPLS-PILANIVLD-ELD-------------------
-----WEL-EH--------------------------------------RG---------HK-----------
------------------------------FARYADDCNI--MV------KSKAA----GE-
RVMKSIRRFLETTLRLRVNDQKSAVDR-------PT---------------------------KRNFLGFTF-
>DS_Sh.ba.I2/CP000563/2137684..2139633/Shewanella baltica/Bacterial C
GIDGMTI--------------------EA-FPL---WMQQ-GGWQRCKS---LLER------GEY---
NPSAVRRVEIDK---P--------------------DG---G-------KRKLGIPNVIDRVIQQAIAQILT-
--PLFDPF-FSANSFGF---RPNRN-----AKQAVLQVRDI----I----KQ-------------K-------
RKF---AVDVDLSKFF-DRVNHDLLMTQL--RI--K-VQ---------DKR-LLALIG--KYLRAGV------
---TVND------------------------------------------------------------------
--------------------------------------------------QFEAS----FE------------
-------------------------------------------------------------------------
-------------------------------GVPQGGPLS-PLLSNIMLD-SLD-------------------
-----KEL-ES--------------------------------------RG---------HK-----------
------------------------------FARYADDFII--LV------KSIRA----GE-
RVLKSITRYLATKLKLVVNEQKSQVVE-------VG---------------------------QSKFLGFTF-
>DS_fid|22934106|locus|VBIPhoPro109272_1767| [Photobacterium profundum
SS9]
-------------------------------------------------------------------------
---------------------------------------------MFMRIPCVIDRVIQQAIAQVLT---
PIFDPD-FSNNSYGF---RPGRN-----GQQAVRPVQST----I----KQ-------------R-------
RHY---AVDVDLSKFF-DRVNHDLLMTHL--GY--K-VK---------DKR-LLKLIS--RYLRAGV---
ICQSKGD--N---------------------------------------------------------------
---------------------------------------P-------------LYM-K----SR---------
-------------------------------------------------------------------------
--------------------------------E-GVPQGGPLS-PLLANIMLD-LLD----------------
--------KEL-EK--------------------------------------RG---------HK--------
---------------------------------FARYADDFTI--LV------KSQRA----GQ-
RVLLSISRYLQNRLKLTVNTTKSHVVR-T--T-------------------------------ESKFLGFTF-
>DS_P.ae.I1/AY029772/3515..5441/Pseudomonas aeruginosa/Bacterial C
GIDGMNI--------------------DE-FPA---WVRS-GNWKALKQ---QLVT------GCY---
QPSPVRRVEIAK---P--------------------DG---G-------TRQLGIPTVTDRVIQQAITQVLT-
--PIFDPE-FSEHSFGF---RPGRN-----GQQAVKQVQSI----I----KE-------------G-------
RRF---AVDVDLSKFF-DRVNHDLLMTRL--GDKVK------------DKR-LLRLIK--RYLRAGF---I--
------D------------------------------------------------------------------
```

```
--------------------------------------------------NQFKG----ES------------
-------------------------------------------------------------------------
-----------------------------RVGVPQGGPLS-PLLANIMLD-SLD-------------------
-----KEL-EK--------------------------------------RG--------HK------------
------------------------------FARYADDFTI--LV------KSQRA----GE-
RVLRSISQYLQSRLKLVVNTDKSRVVK-------TN---------------------------ESQFLGFTF-
>DS_N.e.I1/AL954747/2285095..2287101/Nitrosomonas europaea/Bacterial C
GIDGVTT--------------------AE-WPE---HARA--HWPATRE---QIEA------GRY---
RPQPVRRVDIPK---P--------------------DG---G-------QRQLGIPTVTDRVIQQAIAQVLI-
--PIFDPG-FSASSFGF---RPGRN-----AHQAIRQVQAH----V----KA-------------G-------
YRW---AVDLDLARFF-DNVNHDLLMSLL--SR--S-IA---------DKR-LLALIG--RYLRAGV---L--
------V------------------------------------------------------------------
------------------------------------G-------------EHPQP----SE------------
-------------------------------------------------------------------------
-----------------------------V-GTPQGGPLS-PLLANVLLH-QFD-------------------
-----LEL-ER--------------------------------------RG--------HR------------
------------------------------FARYADDVII--LV------KSRRA----AE-
RVMQSLTYFLQSTLKLTVNLAKSQVAP-------MS---------------------------ECSFLGFTL-
>DS_DEfid|124817422|locus|VBIDesSul232581_2428| [Desulfocapsa sulfexigens
DSM 10523]
GIDEITV--------------------GD-FPF---TFRE--CWPEIRS---TILE------GNY---
TPSPVQRVEIPK---P--------------------DG---S-------TRPLGIPTVLDRVIQQAIAQVMS-
--PIFEPH-FSESSCGF---RPGRS-----AHDGVKQIKQY----I----RQ-------------G-------
YKV---AVDMDLSKFF-DTVNHDVLMNRV--SR--R-IE---------DKR-VLKLIG--KYLRAGV---M--
---V--N------------------------------------------------------------------
------------------------------------G-------------RRLAT----PL------------
-------------------------------------------------------------------------
-------------------------------GVPQGGPLS-PLLANILLD-DLD-------------------
-----KEL-EK--------------------------------------RG--------HH------------
------------------------------FVRYADDFII--LV------KSLSA----AE-
RVMASVSRFLKRELRLIVNEKKSSFGK-------VE---------------------------ECSFLGFVF-
>DS_Bfid|21259697|locus|VBICanAcc132554_2105| [Candidatus Accumulibacter
phosphatis clade IIA str. UW1]
GIDGLRI--------------------ED-FPA---YACE--HWPAIRQ---TLSE------GRY---
QPQAVRRVIIPK---P--------------------NG---G-------ERALGIPTVVDRVVQQAIAQIMT-
--PIFDPE-FSESSYGF---RPRRS-----AHGALKQVRAD----L----KA-------------G-------
YRI---AVDLDLAKFF-DNVDHDILMARV--AR--K-VS---------DKR-LLALIG--RYLRAGV---M--
------I------------------------------------------------------------------
------------------------------------G-------------STLQP----SE------------
-------------------------------------------------------------------------
-----------------------------L-GTPQGGPLS-PLLANILLD-DLD-------------------
-----RTL-EG--------------------------------------RG--------HR------------
------------------------------FARYADDLMV--LV------KSERA----GQ-
RVKASLTAYLGRQLKLPVNEKKSQVAK-------IE---------------------------QCVFLGFTF-
>DS_fid|115641687|locus|VBIThiNit264030_2345| [Thioalkalivibrio
nitratireducens DSM 14787]
GMDGMPI--------------------ED-FPT---FARR--HWPQIRR---QLAD------GVY---
QPQPVRRVAIPK---P--------------------KG---G-------ERLLGIPTVMDRVIQQAIAQVLT-
--PIFDPD-FSDSSFGF---RPGRS-----AHGALRQVQGH----I----QA-------------G-------
YRI---AVDLDLAKFF-DNVQHDVLMARV--ARKVR------------DKR-LLALIG--RYLRAGV---L--
------V------------------------------------------------------------------
------------------------------------G-------------KSVQA----TG------------
-------------------------------------------------------------------------
-----------------------------I-GTPQGGPLS-PLLANILLD-DLD-------------------
```

```
-----REL-ER--------------------------------------RG---------HR-----------
------------------------------FTRYADDLVI--LV------KTLRA----GD-
RVKASVTRFLARKLALLVNEQKTRVVK-------TN---------------------------DCQFLGFTF-
>DS_fid|31921407|locus|VBIAllVin64954_0826| [Allochromatium vinosum DSM
180]
GIDGMCI--------------------ED-FPE---FARS--SLPAIRQ---ALRE------GTY---
RPQPVRRVTIPK---P--------------------NG---G-------ERLLGIPTVMDRVIQQAIAQVLG-
--PIFDPG-FSDASFGF---RPGRS-----AHGALRRVQTY----I----GE-------------G-------
YRI---AVDLDLAKFF-DTVQHDVLMARV--GRKVR------------DKR-LLALIG--DYLRAGV---L--
------V------------------------------------------------------------------
------------------------------------G-------------GTLEA----TE------------
-------------------------------------------------------------------------
-----------------------------I-GTPQGGPLS-PLLANILLD-DLD-------------------
-----KEL-ER--------------------------------------RG---------HR-----------
------------------------------FVRYADDLLI--LV------RSHRA----GE-
RVMASVSRYLTGTLKLVVNEQKSRVVK-------TD---------------------------ACKFLGFTF-
>DS_G.s.I1/AE017180/1028657..1030564/Geobacter sulfurreducens/Bacterial C
GVDGVTI--------------------DA-FPE---RFRP--LWGDIRA---SLAT------GTY---
QPQPVLRVEIPK---P-T-----------------G-G-----------TRPLGIPTVLDRLIQQATAQVLT-
--PIFDPE-FSASSFGF---RPGRS-----AHNAVRQLREY----L----RQ-------------G-------
YRI---AVDIDLAKFF-DTVNHDLLMTMV--GR--R-VR---------DKR-VLTLIG--RYLRAGV---E--
---V--D------------------------------------------------------------------
------------------------------------G-------------RLE-K----TR------------
-------------------------------------------------------------------------
-----------------------------M-GVPQGGPLS-PLLANILLD-HLD-------------------
-----KEL-ES--------------------------------------RG---------HK-----------
------------------------------FVRYADDFVI--LV------KSERA----GE-
RVMGSVRKYLTNKLKLTVNEDKSKVAR-S--G-------------------------------DLSFLGFVF-
>DS_DEfid|38166048|locus|VBIDesAlk70802_2461| [Desulfurivibrio
alkaliphilus AHT2]
-MDNMPI--------------------AD-FMA---FARE--HWEEIRA---SLLA------GTY---
QPLPVKRVEIPK---P--------------------TG---G-------TRPLGIPTVLDRLIQQAMAQVLL-
--PIFDPD-FSEASYGF---RPGRS-----AHDAIHRVRDY----I----RQ-------------G-------
YRV---AVDADLSKFF-DTVDHDLLMNRV--GRKVR------------DQR-VLRLVG--KYLRAGV---M--
---I--D------------------------------------------------------------------
------------------------------------G-------------RRRET----RK------------
-------------------------------------------------------------------------
-------------------------------GVPQGGPLS-PLLSNILLD-DLD-------------------
-----KEL-ER--------------------------------------RG---------HR-----------
------------------------------FARYADDFII--LV------KSRRA----GE-
RVMTGITRFLESKLKLVVNQEKSKVAP-------TN---------------------------ESGFLGFIF-
>DS_Actinobacteriafid|42167991|locus|VBIArtAri166201_0751| [Arthrobacter
arilaitensis Re117]
GVDGLEA--------------------HE-LRD---WCRE--HWIETRK---SLDA------GTY---
APLPVRQVMIPK---P-D-----------------G-G-----------ERMLGVPSVLDRLIQQALAQVLS-
--PIFDEG-FAPMSYGF---RPGKS-----AHDAASMARKV----I----EQ-------------G-------
YRW---VVEVDLDAFF-DRVNHDVLMSRV--AR--K-VK---------DKR-VLKLVR--KYLTAGI---M--
---A--Q------------------------------------------------------------------
------------------------------------G-------------VRR-E----TV------------
-------------------------------------------------------------------------
-----------------------------E-GTPQGSPLS-PLLSNIILD-DFD-------------------
-----QEF-WS--------------------------------------RD---------HR-----------
------------------------------FVRYADDIRI--FV------KSKRA----AE-
RVLGQATKVLEQRLKLKVNRQKSVINP-A--S-------------------------------VATLLGFGF-
```

>DS_Bacteroidetesfid|61290801|locus|VBINiaKor154066_6175| [Niastella koreensis GR2010]
GIDGVEA--------------------KD-FKL---QLDG--AWVQVKS---QLEN------GSY---
QPQAVKRVTIPK---P--------------------NG---G-------ERHLGIPTYMDRLIQQAISQVLV-
--KQYEPT-FSENSYGF---RSEKN-----AHQAALKAKEY----I----NA-------------G-------
YSH---VVDLDLSQFF-DRVNHDYLMNEL--SR--R-IT---------DKR-VLKLIH--KILRSEI---A--
------E--
------------------------------------G-------------ANRIP----CK------------
----
-----------------------------Q-GVPQGGPLS-PLLSNIILD-KLD-------------------
-----KEL-EK--------------------------------------RG---------LR-----------
------------------------------YVRYADDCSI--YV------KSKRA----GD-
RVMESITRYIEKELKLVVNAVKSSVTR---PW-------------------------------LMKLLGFTF-
>DS_Gfid|20127583|locus|VBIVibHar24526_0381| [Vibrio harveyi ATCC BAA1116]
GVDKLDI--------------------DATIFK----LRQASNGQALRQ---SLLD------GSY---
RPQPVLGVGIPK---P--------------------SG---G-------VRQLGIPTVIDRIVQQAITSVLS-
--DIYEAK-FSNSSYGF---RPNRS-----AHHALAAASRY----I----RE-------------G-------
RGY---VVDIDLAKYF-DTVNHDRLMHRL--SE--D-IA---------DKR-VLKLIR--SYLQAGI---M--
---R--N--
------------------------------------G-------------LVE-Q----RQ------------
----
-----------------------------R-GTPQGGPLS-PLLSNIVLD-ELD-------------------
-----KEL-ER--------------------------------------RG---------HK-----------
------------------------------FCRYADDCQI--YV------GSEEA----AY-
RVKESITEYLEQKLKLTVNREKSAATR-------VT---------------------------ERTYLSHRF-
>DS_clostridiafid|61457273|locus|VBISulAci142080_3712| [Sulfobacillus acidophilus DSM 10332]
GVDGVTT--------------------DE-FVD---YLWE--HWPTIQG---QLRA------GTY---
HPQPIRGVEIPK---P--------------------TG---G-------VRMLGIPTAIDRFIQQAVLQVLT-
--PIFDPQ-FSDHSYGF---RPGRS-----AHQAVRQVRRQ----A----EA-------------G-------
AEW---VIDLDLEKFF-DRINHDILMARV--AR--R-VQ---------DPQ-VLRLIR--RYLQAGL---M--
---L--H--
------------------------------------G-------------VSTPR----TQ------------
----
-------------------------------GAAQGGPLS-PLLANILLD-DLD-------------------
-----KEL-AR--------------------------------------RG---------LA-----------
------------------------------YVRYADDAMI--LV------HSRRA----GE-
RVLASVSRYLDRTLHLPVNLTKSAVDR-------LV---------------------------RRTYLGFKF-
>DS_clostridiafid|61455293|locus|VBISulAci142080_2739| [Sulfobacillus acidophilus DSM 10332]
GVDGVST--------------------KA-LVD---YLGA--HWPMIRP---QLRD------GTY---
RPHAIRGVEIPK---P--------------------TG---G-------VRTLGIPTVVDRFIQQAVLQVLT-
--PIFDPH-FADFSFGF---RPGRS-----AHQAVRHVRRL----A----ED-------------G-------
AEW---GVDLDLEQFF-DRINHDIRMARV--AR--R-VQ---------DLQ-VLRLIR--RYLQAGI--RV--
------D--
------------------------------------G-------------VSAPR----TA------------
----
-------------------------------GAAQGSPLS-PLLANIVLD-DFD-------------------
-----KEL-ER--------------------------------------RG---------VA-----------
------------------------------FVRYADDAMI--FV------HSQRA----GE-
RVLTSVTRYLEHRLHLPVNTAKSAVDR-------LT---------------------------RRPYLGFKF-
>DS_Bfid|190413778|locus|VBIVarPar264937_3261| [Variovorax paradoxus B4]

GVDGRTV--------------------QQ-TGE---DLKT--QWPDIRR---GLLD------GTY---
RPSPVRRVGIPK--LG---------------------G---G-------TRELGIPTVVDRLIQQALLQVLQ-
--PLIDPT-FSEHSYGF---RPGRS-----AHQAVQAARQY----V----EQ-------------G-------
RRV---VVDVDLGKFF-DRVNHDILMDRL--GK--R-IA---------DKA-VLRLIR--HYLNAGI---M--
---A--H--
------------------------------------G-------------VMQMR----VE------------
----
-------------------------------GTPQGGPLSPPLLANVLLD-EVD-------------------
-----RAL-ER--------------------------------------RG---------RK-----------
------------------------------FVRYADDCNV--YV------KSERA----GQ-
RVLDGVRACYA-KLRLKVNETKTAVAT---AW-------------------------------GRKFLGYCL-
>DS_P.s.I1/AE016853/2381076..2382906/Pseudomonas syringae/Bacterial C
GVDGLGI--------------------VE-TAE---HLKT--AWPGIRA---QLLA------GTY---
RPDPVRRVLIPK---P-G-----------------G-G-----------ERKLGIPTVTDRLIQQALLQVLQ-
--PLLDPD-FSNHSYGF---RPERS-----AHQAVLAAQQY----I----HS-------------G-------
RQI---VVDVDLEQFF-DCVEHDVLIARL--GR--K-VK---------DRD-VLRLIR--AYLNSGA---L--
---I--E--
------------------------------------G-------------MVM-T----ST------------
----
-----------------------------R-GTPQGGPLS-PLLANVVLD-EVD-------------------
-----KEL-ER--------------------------------------RG---------HC-----------
------------------------------FVRYADDANV--YV------RSPKA----GQ-
RVMALLRRLYG-RLGLRVNESKSAVAS-A--F-------------------------------GRKFLGFSF-
>DS_fid|23651005|locus|VBISodGlo61428_2803| [Sodalis glossinidius str.
'morsitans']
GVDGLSI--------------------AQ-TGQ---HLKY--AWPTIRQ---QVMI------GTY---
RPQPVRRVGIPK---P--------------------DG---S-------ERELGIPTVIDRLIQQALLQVLQ-
--PLIDPT-FSEYRYGF---RPGRR-----GHDAVLASHQY----V----QD-------------G-------
YRV---VVDVDLSKFF-DRINHDILIDRL--RK--H-VN---------DAG-VIRLVR--AYLNAGI---M--
------K--
------------------------------------G-------------GVVVE----HA------------
----
-----------------------------E-GTPQGCPLS-LLLTNVLLD-EVD-------------------
-----REL-EL--------------------------------------RG---------HR-----------
------------------------------FARYADDCNV--YV------RSEKT----GE------------
----
>DS_Gfid|161737930|locus|VBIAzoVin292307_2623| [Azotobacter vinelandii
CA]
GVDGLDI--------------------DQ-TES---HLRQ--VWPSIRQ---QLLM------GTY---
QPLSVRRVCIPK---P--------------------DG---S-------ERELGIPSVTDRLIQQALLQVLQ-
--PLIDPS-FSEHSHGF---RPGRR-----AWDAVLSAQRY----A----QE-------------G-------
YCI---VVDVDLSRFF-DRVNHDILIDRL--RR--Q-VN---------DTG-VIRLVR--AYLNAGI---M--
------D--
------------------------------------G-------------GVVVE----RL------------
----
-----------------------------E-GTPQGGPLS-PLLANVLLD-AVD-------------------
-----KEL-ER--------------------------------------RG---------HR-----------
------------------------------FARYADDCNV--YV------RSQKA----GE-
RVMALLKRCYD-KLRLKINESKSAVAG---VF-------------------------------GRSFLGYCL-
>DS_Afid|54141352|locus|VBISinMel152503_5994| [Sinorhizobium meliloti
SM11]
GADGLSI--------------------EA-TAA---HLRT--AWPGIRE---RVLA------GTY---
RPMPVRRVTIPK---P-------------------D-G---G-------ERELGIPTVTDRLIQQALLQVLQ-
--PLLDPT-FSEHSHGF---RPGRS-----AHDAVLEAQSY----V----QS-------------G-------

RRI---VVDVDLEKFF-DRVNHDILIDRL--SK--R-IS---------DKR-VIRLIR--AYLNSGI---M--
---D--H--
------------------------------------G-------------VVQ-E----RV------------
----
-----------------------------M-GTPQGGPLS-PLLANVLLD-EVD-------------------
-----KEL-ER--------------------------------------RG---------HC-----------
------------------------------FVRYADDCNV--YV------GSRKA----GE-
RVMALLRRLYG-RLHLTINEGKSAVTS-------VF---------------------------GRKFLGFSF-
>DS_fid|54129720|locus|VBISinMel152503_0312| [Sinorhizobium meliloti SM11]
GADGLSI--------------------EA-TAA---HLRT--SWPGIRE---RVLA------RTY---
RPMPVRRVTIPK---P--------------------DG---G-------ERELGIPTVTDRLIQQALLQVLQ-
--PLLDPA-FSEHSHGF---RPGRS-----AHGAVLAAQSL----V----QS-------------G-------
RRI---VVDVDLEKFF-DRVNHDILIDRL--SK--R-IS---------DKR-VIRLIR--AYLNSGI---M--
---D--H--
------------------------------------G-------------VVQER------------------
----
-----------------------------VMGTPQGGPLS-PLLANVLLD-EVD-------------------
-----KEL-ER--------------------------------------RG---------HC-----------
------------------------------FVRYADDCNV--YV------GSRKA----AN------------
--------------------G-------------------------------SWRFCGGFT-
>DS_Gfid|31925696|locus|VBIAllVin64954_2919| [Allochromatium vinosum DSM 180]
GVDGLDI--------------------EQ-TAR---LLVT--EWPKIRD---QLLR------GKY---
RPSPVRRVTIPK---P--------------------DG---G-------ERELGIPTVTDRLIQQALLQVLQ-
--PRLEPT-FSEHSYGF---RPGRR-----AHDAILAAQGF----I----QS-------------G-------
RKI---VVDVDLEKFF-DRVNHDILIDRL--QK--R-ID---------DAG-IIQLIR--AYLNSGI---M--
---N--D--
------------------------------------G-------------VVLER----YQ------------
----
-------------------------------GTPQGGPLS-PLLANVLLD-EVD-------------------
-----KVL-EK--------------------------------------HG---------HC-----------
------------------------------FARYADDCNV--YV------RSRKA----GE-
RVMALLRKCYG-TLRLKVNEAKSAVAS-------VT---------------------------GRTFLGYSF-
>DS_B.f.I1/NZ_AAAC01000271/24723..26575/Burkholderia fungorum/Bacterial C
GVDGLDI--------------------GQ-TAR---HLVT--AWPVIRE---QLLK------GTY---
RPDPVRRVTIPK---P--------------------DG---G-------ERELGIPTVTDRLIQQALLQVLQ-
--PILDPT-FSEHSYGF---RPGRR-----AHDAVLAAQSY----V----QS-------------G-------
RRI---VVDVDLEKFF-DRVNHDILIDRL--KR--R-ID---------DAG-VIRLVR--TYLNSGI---M--
---D--D--
------------------------------------G-------------VVQQR----DQ------------
----
-------------------------------GTPQGGPLS-PLLANVLLD-EVD-------------------
-----KEL-ER--------------------------------------RG---------HC-----------
------------------------------FARYADDANV--YV------RSRRA----GE-
RVMALLRRLYG-RLRLKVNETKSAVAS---VF-------------------------------GRKFLGYSL-
>DS_Bu.cp.I1/NZ_AAEH01000016/115992..117833/Burkholderia cepacia/Bacterial C
GVDGKSI--------------------AE-TAE---HLKT--HWPGIRE---ALLD------GSY---
RPWPVRRVQIPK---P--------------------DG---G-------MRELGIPTVADRLIQQALLQVPQ-
--PIIDPT-FSEHSYGF---RPGRR-----ARDAVLMAQRH----V----QD-------------G-------
YRM---VVDVDLEKFF-DRVNHDILMERL--SR--R-ID---------DKA-VLRLIR--LYLVAGI---M--
------D--
------------------------------------G-------------GVVSE----RY------------

```
-------------------------------------------------------------------------
-----------------------------E-GTPQGGPLS-PLLANVLLD-EVD-------------------
-----REL-ER--------------------------------------RG---------HK-----------
------------------------------FVRYADDCNV--YV------RSGRS----GE-
RVLEGLCKLYD-RLHLKVNEAKTAVAP-------AT---------------------------GRKFLGYRL-
>DS_Bfid|22964000|locus|VBIPolSp102244_5444| [Polaromonas sp. JS666]
GVDGLTI--------------------EE-TPE---YLKT--HWSRIRL---ELLN------GTY---
RPQAVRRVEIPK---P--------------------TG---G-------MRELGIPTVLDRLIQQALLQVLQ-
--PMIDLT-FSEFSYGF---RPGRS-----AHDAVLQAQRY----V----QE-------------G-------
FQV---VVDVDLEKFF-DRVNHDILMDRL--AK--R-IA---------DKA-VLRLIR--QYLQAGI---M--
------A------------------------------------------------------------------
------------------------------------G-------------GVVMD----RS------------
-------------------------------------------------------------------------
-----------------------------E-GTPQGGPLS-PLLANVLLD-EVD-------------------
-----LDL-QR--------------------------------------RG---------HR-----------
------------------------------FARYADDCNV--YV------RSQKA----GE-
RVLLSLRKLYE-KLHLKVNEKKTEVGP---VF-------------------------------GRKFLGYCL-
>DS_B.sp.I1/NZ_AAOX01000004/96386..98244/Bacillus sp./Bacterial C
GVDEKDI--------------------EA-TRL---YLRE--NGQEIIQ---LIRE------GKY---
KPQPVRRVEIPK--AN---------------------G---G-------KRQLGIPTVTDRVIQQAVVQRLT-
--PIFERQ-FSHFSYGF---RPNKS-----AHQAIEQARQY----I----EE-------------G-------
YNF---VVDMDLEKFF-DRVQHDKLMSLI--AK--T-IS---------DKP-TLKLIR--RFLQAGV---M--
---V--N------------------------------------------------------------------
------------------------------------G-------------VVITN----RE------------
-------------------------------------------------------------------------
-------------------------------GTPQGGPLS-PLLSNIILN-ELD-------------------
-----KEL-EK--------------------------------------RG---------HK-----------
------------------------------FVRYADDCNI--YV------KSIKA----GE-
RVKQGVTEFLERKLKLKVNEEKSAVGK-------PS---------------------------ARTFLGVSF-
>DS_P.a.I1/U77945/1..1919/Pseudomonas alcaligenes/Bacterial C
GADGMTV--------------------AD-LAG---YVKQ--YWPTLKA---RLLA------GEY---
HPQAVRAVEIPK---P--------------------QG---G-------TRQLGIPSVVDRLIQQALQQQLT-
--PIFDPL-FSDYSYGF---RPGRS-----THQAIEMARAH----V----TA-------------G-------
HRW---CVELDLEKFF-DRVNHDILMACI--ER--R-IK---------DKC-VLRLIR--RYLEAGI---M--
------S------------------------------------------------------------------
------------------------------------G-------------GVVSP----RQ------------
-------------------------------------------------------------------------
-----------------------------E-GTPQGGPLS-PLLSNILLD-ELD-------------------
-----REL-ER--------------------------------------RG---------HR-----------
------------------------------FVRYADDANI--YV------RSPRA----GE-
RVLVSVERFLRERLKLTVNRKKSQVAR---AW-------------------------------KCDYLGYGM-
>DS_Ha.ch.I2/NC_007645/98723..100647/Hahella chejuensis/Bacterial C
GVDQMPV--------------------AA-LKG---HLQQ--HWPTLRE---RLLA------GDY---
HPQPVRRVSIPK---P--------------------QG---G-------ERILGIPTVQDRLIQQALHQVLS-
--PMLEPI-FSDHSYGF---RPGRS-----AHQAVRAMQRH----I----ND-------------G-------
HRW---VVDLDLEQFF-DRVNHDVLMGLL--AR--R-IA---------DRR-MLTLIR--RYLQAGM---L--
------D------------------------------------------------------------------
------------------------------------G-------------GLVSP----RR------------
-------------------------------------------------------------------------
-----------------------------E-GAPQGGPLS-PLLSNVLLT-ELD-------------------
-----REL-ER--------------------------------------RG---------HR-----------
------------------------------FCRYADDCNI--YV------RSERA----GH-
RVMTSITHYLKMHLRLKVNAEKSVVDR---PW-------------------------------RRSYLGYSV-
```

>DS_Bfid|21262583|locus|VBICanAcc132554_3525| [Candidatus Accumulibacter phosphatis clade IIA str. UW1]
GVDGLTV--------------------FE-LKA---WLQQ--HWPSVKA---ALLA------GDY---
LPAAIRKVEMPK---P--------------------NG---G-------VRILGIPTVLDRLIQQALLQVLQ-
--PEFEPE-FSEHSYGF---RPGRN-----AWQAVQRAQGY----I----RE-------------E-------
RRW---VVDLDLEKFF-DRVNHDILMSRV--AR--R-VK---------DER-VLKLIR--RYLEAGM---M--
---S--E--
------------------------------------G-------------MVSAR----TE------------
----
-------------------------------GTPQGGPLS-PLLSNILLT-DLD-------------------
-----REL-ER--------------------------------------RG---------HR-----------
------------------------------FCRYADDCNI--YV------KSKMA----GQ-
HAMDAITDYLEQKLKLRVNRDKSAVAR---PW-------------------------------QRKFLGYSV-
>DS_Bfid|21533277|locus|VBICupTai42494_3259| [Cupriavidus taiwanensis]
GVDALEV--------------------TA-LRD---WLKV--SWPSVRA---ALLG------GQY---
IPQSVRAVDIPK---P--------------------SG---G-------VRTLGIPTVVDRLIQQALLQVLQ-
--PLYEPG-FSESSYGF---RPRRS-----AQQAVLQAQRY----V----QE-------------G-------
RRW---VVDIDLEKFF-DRVNHDILMSRV--ARQVK------------DVR-VLKLIR--RYLEAGL---M--
------R--
------------------------------------G-------------GVVEA----RR------------
----
-----------------------------Q-GTPQGGPLS-PLLSNILLT-DWD-------------------
-----REL-EK--------------------------------------RG---------LA-----------
------------------------------FCRYADDCNI--YV------RSQAA----GQ-
RLLAGMMTFLAERLNLQVNEAKSACAR---PW-------------------------------ARKFLGYSL-
>DS_Bfid|45186915|locus|VBIBurRhi170666_3219| [Burkholderia rhizoxinica HKI 454]
GVDGLPV--------------------EQ-FKD---WLKM--HWPSVKA---ALLD------ARY---
MPAAVRAVDIPK--SA---------------------G---G-------VRTLGIPTVLDRLIQQALHQVLQ-
--PIFEPG-FCESSYGF---RPRRS-----AQQAVLAAQRY----V----QE-------------G-------
RRW---VVDIDLAKFF-DRVNHDILMARV--ARQVK------------DAR-VLKLIR--RYLEAGL---M--
-----RE--
------------------------------------G-------------VAPAR----RE------------
----
-------------------------------GAPQGGPLS-PLLSNILLT-DWD-------------------
-----REL-ER--------------------------------------RG---------HA-----------
------------------------------FCRYADDCNI--YV------RSKAA----GE-
RLLTQMTTFLAKRLKLHINEAKSACAR---PW-------------------------------ERKFLGYSL-
>DS_fid|190304141|locus|VBISerSp8482_4636| [Serratia sp. ATCC 39006]
GVDKLTV--------------------QE-LKP---WLKQ--HWLSVKG---TLIA------GSY---
LPRAIRKVDIPK---P--------------------NG---D-------VRTLGIPTVVDRLIQQAIAQTLS-
--PYVEPS-FSNSSYGF---RPNRN-----AWQAVRQAQQY----I----QS-------------G-------
KRW---VVDMDLEKFF-DRVDHDILMSRL--ARTIK------------DKR-LLKLIR--RYLEADM---V--
------E--
------------------------------------G-------------KEVIK----RD------------
----
-----------------------------K-GMPQGGPLS-PLLSNILLD-ELD-------------------
-----KEL-ER--------------------------------------RG---------HS-----------
------------------------------FCRYADDCNI--YV------SSQKA----GK-
HAQKDISEFLMNTLKLQVNVRKSAVAR---PW-------------------------------ERKFLGYSF-
>DS_fid|190303244|locus|VBISerSp8482_4195| [Serratia sp. ATCC 39006]
GVDNLSV--------------------GE-LKG---WLKQ--HWASVRE---ALLQ------GNY---
VPQAIRQVEIPK---P--------------------DG---G-------VRILGIPTVVDRLIQQAIQQHLT-
--PDYEPE-FSDSSYGF---RPGRN-----AGQAVQQAQSY----M----QS-------------G-------

RRW---VVDLDLEKFF-DRVNHDILMARLSWKI--K------------DTR-LLKLIR--RYLEADR---V--
------A--
------------------------------------G-------------SEITR----RR------------
----
-----------------------------E-GMPQGSPLS-PLLSNILLT-DLD-------------------
-----REL-ER--------------------------------------RG---------HK-----------
------------------------------FCRYADDGNI--YV------CSRQA----GE-
HAMKEISHYLENKLRLKVNAHKSAVDR---PW-------------------------------KRKFLGYSV-
>DS_fid|180719125|locus|VBIEscCol277189_4107| [Escherichia coli HVH 121
(46877826)]
--RRTS------AGH--------
-----PD-------------------------G---G---------GSLHPAGDDAGIAG----------
ALWDSS-FSDNSYGF---RPGRS-----AHQAVIQAREH----I----GA-------------G-------
YHW---VVDLDLEKFF-DRVNHDVLMSRI--EK--R-VS---------DKR-VLSLIR--RFLNAGV---M--
---D--A--
------------------------------------G-------------LVRPV----TE------------
----
-------------------------------GTPQGGPLS-PLLSNLLLD-DLD-------------------
-----KEL-EK--------------------------------------RG---------LK-----------
------------------------------FVRYADDCNV--YV------KSERA----DN-
RIMAGLTHWLSHKLKLKVNAKKSAVAR-------PE---------------------------TRKFPGYSF-
>DS_Gfid|21803083|locus|VBIDicZea111179_3566| [Dickeya zeae Ech1591]
GVEGMSV--------------------SE-LPD---YLKH--HWPELKA---QLLS------GSY---
CPSPVRRVTIPK---P--------------------GG---G-------ERLLGIPTVVDRFVQQATMQVLQ-
--RQWDAS-FSDSSYGF---RPGRS-----AHQAVKQAQGY----I----GS-------------G-------
HHW---VVDLDLEKFF-DRVNHDVLMSRV--AK--R-VS---------DKR-VLSLIR--GFLNAGV---M--
---E--A--
------------------------------------G-------------LVSPV----TE------------
----
-------------------------------GMPQGGPLS-PLLSNLLLD-DFD-------------------
-----KEL-EK--------------------------------------RG---------LK-----------
------------------------------FARYADDCNI--YV------KSERA----GN-
RVMEGLTHWLSRKLKLKVNAKKSAVAH-------PA---------------------------MRKFLGYSF-
>DS_Bu.vi.I2/CP000617/381828..383697/Burkholderia vietnamiensis/Bacterial
C
GVDGMTV--------------------QA-LPA---FLRE--QWPSIRA---TLLN------GTY---
KPQPVRRVEIPK---P-------------------DGG---G-------VRKLGIPCALDRFVQQAVLQVLQ-
--RQWDPT-FSEASYGF---RPGRS-----AHQAVAKAQSY----I----QS-------------G-------
YRW---VVDLDLEKFF-DRVNHDILMSRV--AR--R-VS---------DRR-VLKLIR--SFLTAGV---M--
---E--H--
------------------------------------G-------------LVGAT----DE------------
----
-------------------------------GTPQGGPLS-PLLSNLMLD-DLD-------------------
-----REL-GR--------------------------------------RG---------LR-----------
------------------------------FVRYADDCNV--YV------RSERA----GQ-
RVMVGLKAFLTGKLKLKVNEAKSAVAR-------PH---------------------------TRKFLGFSF-
>DS_So.us.I3/CP000473/9594438..9596378/Solibacter usitatus/Bacterial C
GVDGMTV--------------------IG-IKD---YLKQ--HWPAIRG---QLLS------GTY---
EPKPVRRVEIAK---P--------------------DG---G-------VRKLGIPTVLDRFIQQAVMQVLQ-
--RRWDRT-FSDYSYGF---RPGRS-----AQQAVAQAQQY----I----AE-------------G-------
HGW---CVDLDLEKFF-DRVNHDKLMGQI--AK--R-IA---------DKR-LLKLIR--AFLNAGV---M--
---E--N--
------------------------------------G-------------LVSPS----VE------------
----

```
-------------------------------GTPQGGPLS-PLLSNLVLD-EFD-------------------
-----REL-ER--------------------------------------RG---------HR-----------
------------------------------FVRYADDCNI--YV------RSERA----GQ-
RVMESITQFITQKLKLKVNETKSAVAR-------PQ---------------------------ERKFLGFSF-
>DS_Br.sp.I1/CP000494/6816299..6818172/Bradyrhizobium sp./Bacterial C
GVDGMTV--------------------DD-LPT---YLKA--NWLTIRA---QLLD------GTY---
KPQAVRRVEIPK--AS---------------------G---G-------VRLLGIPTVVDRFIQQAVLQVLQ-
--GEWDRT-FSDASYGF---RPGRS-----AHQAVTKAQAY----IA---SR---------------------
HRI---VVDIDLEKFF-DRVNHDILMGLV--AK--R-VA---------DKR-LLKLIR--GFLTAGV---L--
------E------------------------------------------------------------------
------------------------------------G-------------GLVSP----TE------------
-------------------------------------------------------------------------
-----------------------------E-GAPQGGPLS-PLLSNLMLD-VLD-------------------
-----KEL-ER--------------------------------------RG---------HR-----------
------------------------------FVRYADDCNI--YV------RSRKA----GE-
RVMASIETFLERCLKLKVNRAKSAVAR-------PN---------------------------HRKFLGFSF-
>DS_clostridiafid|42639654|locus|VBIHalSp157090_0576| [Halanaerobium
hydrogeniformans]
GIDGMSV--------------------DE-LLP---LLKR--NGSQLLK---DILE------GNY---
KPQAVRRVEIPK---P--------------------GG---G-------VRLLGIPTVIDRMIQQAITQQLT-
--PIFDPG-FSEYSYGF---RPGRN-----AHQAVNKAKEY----I----ND-------------G-------
YTW---VVDIDLEKYF-DTVQHDKLMSLV--AR--K-VQ---------DKR-VLKLIR--AYLNAGV---M--
---I--D------------------------------------------------------------------
------------------------------------G-------------LIK-K----TD------------
-------------------------------------------------------------------------
-----------------------------E-GCPQGGPLS-PLLSNIMLD-ELD-------------------
-----KEL-EK--------------------------------------RN---------HK-----------
------------------------------FCRYADDSQI--YV------KSRKA----AK-
RVMKSLTVFIEKKLKLKVNATKSAVGR---PW-------------------------------RRKFLGFSF-
>DS_C.a.I1/AE001437/3710916..3712835/Clostridium acetobutylicum/Bacterial
C
GVDGMKV--------------------DE-LLQ---YLKQ--NGKTLIA---SIFN------GKY---
CPKAVRRVEIPK---P--------------------DG---G-------IRLLGIPTVVDRTIQQAISQVLT-
--PIFEKT-FSENSYGF---RPKRS-----AKQAIKKAKEY----M----EE-------------G-------
YKW---VVDIDLAKYF-DTVNHDKLMALV--AR--K-IK---------DKR-VLKLIR--LYLQSGV---M--
---I--N------------------------------------------------------------------
------------------------------------G-------------VVS-E----TE------------
-------------------------------------------------------------------------
-----------------------------R-GCPQGGPLS-PLLSNIMLT-ELD-------------------
-----REL-EK--------------------------------------RG---------HK-----------
------------------------------FCRYADDNNV--YV------RSKKA----GD-
RVMRSITRFIENKLKLKVNKEKSAVDR---PW-------------------------------RRKFLGFTF-
>DS_clostridiafid|124770602|locus|VBICloSac181665_0553| [Clostridium
saccharoperbutylacetonicum N14(HMT)]
GVDGMKT--------------------DE-LRE---HIKK--HWETIKV---KLLE------SKY---
NPSPVRRKEISK---P--------------------DG---G-------VRLLGIPTVQDRLIQQAIAQVLS-
--KIYEPL-FSENSFGF---RPHRG-----AKDAITKSKQY----I----TQ-------------G-------
NRW---VIDMDLEKFF-DKVNHDILMNKL--EK--K-IQ---------DKR-LLSLIR--KYLKSGI---L--
---I--N------------------------------------------------------------------
------------------------------------G-------------VSVAS----EE------------
-------------------------------------------------------------------------
-------------------------------GTPQGGPLS-PLLANIMLD-ELD-------------------
-----KEL-ER--------------------------------------RG---------YK-----------
```

```
------------------------------FCRYADDNNI--YV------KSKRA----GF-
RVMKSITNIIENNLKLKVNKDKSAVDF-------VS---------------------------KRKFLGFSF-
>DS_Bacillifid|54384448|locus|VBIPaeMuc192881_5730| [Paenibacillus
mucilaginosus KNP414]
GVDGVTV--------------------AH-LQV---YLKT--HWEAVRA---ALLT------GTY---
RPSPVKRVEIPK---P--------------------GG---G-------VRLLGIPTVMDRFLQQALLQVMN-
--PIFDAH-FSWHSYGF---RPRKR-----AHDAVKQAQRY----I----QD-------------G-------
LRW---VVDMDLEKFF-DRVNHDMLMARV--AR--K-VT---------DKR-VLKLIR--AYLNAGV---M--
---A--D------------------------------------------------------------------
--------------------------------------------------RALER----TD------------
-------------------------------------------------------------------------
-----------------------------E-GTPQGGPLS-PLLANILLD-DLD-------------------
-----KEL-TK--------------------------------------RG---------LR-----------
------------------------------FVRYADDCNI--FV------ASKRA----GE-
RVMKSMTDFVEGKLKLKVNRDKSAVDR---PW-------------------------------NRKLLGFS--
>DS_Afid|20468517|locus|VBIOchAnt73124_1762| [Ochrobactrum anthropi ATCC
49188]
GIDKMSV--------------------GD-LKQ---HLVS--HWPRIRE---DLLA------GRY---
EPAPVRGVEIPK---P---------------------G-G-G-------KRLLGIPTVLDRLIQQAIHQVLM-
--PIFDPG-FSNSSFGF---RPERS-----AHDAILAARSY----V----AD-------------G-------
YRV---VVDLDLEKFF-DRVNHDVLMARV--AR--K-VY---------DKR-VLRLIR--RYLQAGL---M--
------M------------------------------------------------------------------
------------------------------------G-------------GMTTM----RS------------
-------------------------------------------------------------------------
-----------------------------E-GTPQGGPLS-PLLSNVLLD-DLD-------------------
-----KEL-EQ--------------------------------------RG---------HR-----------
------------------------------FCRYADDCNI--YV------RSIRA----GQ-
RVMASLTAFLGRRLKLKVNATKSAVDH---PW-------------------------------NRVFLGYTM-
>DS_Ge.ur.I2/CP000698/242469..244398/Geobacter uraniireducens/Bacterial C
GVDNMPV--------------------TA-LKG---YLQE--EWPRIRE---ELLT------GTY---
HPQPVRKVEIPK---P--------------------GG---G-------TRMLGIPTVLDRLIQQAVHQVLS-
--PLFDPG-FSISSHGF---RPGRS-----AHQAIKAARKY----V----ES-------------G-------
LRW---VVDIDLEKFF-DRVHHDTLMSLV--KR--K-VG---------DRL-VLSLID--SYLKAGI---L--
------E------------------------------------------------------------------
------------------------------------G-------------GVTSP----RL------------
-------------------------------------------------------------------------
-----------------------------E-GTPQGGPLS-PLLSNILLD-ELD-------------------
-----KKL-ER--------------------------------------RG---------HK-----------
------------------------------FCRYADDANI--YV------ATRRS----GE-
RVMASITGYLSERLKLTVNQGKSAVDR---PW-------------------------------KRSFLSYSM-
>DS_Pe.ca.I2/CP000142/1559685..1561618/Pelobacter carbinolicus/Bacterial
C
GIDGMPV--------------------GD-LKT---YLQE--QWPRIKE---ELLT------GTY---
QPQPVRKVEIPK---P---------------------G-G-G-------MRMLGIPTVLDRLIQQALHQELM-
--RLFEPE-FSEHSYGF---RPGRS-----AHQAVQSARRH----V----AS-------------G-------
RRW---AVDIDLEKFF-DRVGHDILMSRV--AR--K-VK---------DRR-VLGLIR--RYLTVGV---L--
------E------------------------------------------------------------------
------------------------------------G-------------GIISP----RV------------
-------------------------------------------------------------------------
-----------------------------Q-GTPQGGPLS-PLLSNILLD-EFD-------------------
-----KEL-ER--------------------------------------RG---------HA-----------
------------------------------FCRYADDCNI--YV------HSRRA----AE-
RVMTSLTRFLEQQLKLKVNRVKSAVGR---PW-------------------------------ERTFLGYSM-
```

>DS_DEfid|21697130|locus|VBIDesRet71890_0165| [Desulfohalobium retbaense DSM 5692]
GVDGMSV--------------------ND-VWG---YCTL--NWARIKE---ELLD------GRY---
EPQPVLGVEIPK---P---------------------G-G-G-------VRQLGIPTALDRLIQQALHQVLS-
--PIFNPH-FSESSYGF---RPGRS-----AHQAVLKAREH----A----AA-------------G-------
KRW---VVDMDLEKFF-DRVNHDVLMARV--AR--K-VK---------DKR-VLVLIR--RYLQAGL---M--
------Q--
------------------------------------G-------------GIASK----RK------------
----
-----------------------------E-GTPQGGPLS-PLLSNILLD-DLD-------------------
-----KEL-ER--------------------------------------RG---------HA-----------
------------------------------FCRYADDCNI--YV------QTKRS----GE-
RAMASITRFLTERLKLRVNADKSAVDR---PW-------------------------------KRKFLGYSM-
>DS_Sy.fu.I1/CP000478/3922427..3924309/Syntrophobacter fumaroxidans/Bacterial C
GVDGVSV--------------------DA-LRA---CLRE--HWPRIKE---ELLE------GRY---
QPQPVRKVEIPK---P---------------------G-GKG-------MRQLGIPTVMDRLIQQALNQVMQ-
--PIFDPD-FSESSYGF---RPGRS-----AHQAVLRAREY----A----AT-------------D-------
RRW---VVDMDLEKFF-DRVNHDILMARL--AR--K-IA---------DRR-VLQLIR--RYLQAGS---M--
------V--
------------------------------------G-------------GVVSP----RT------------
----
-----------------------------E-GTPQGGPLS-PLLSNILLD-DLD-------------------
-----KEL-EQ--------------------------------------RG---------HA-----------
------------------------------FCRYADDCNI--YV------KSRRA----GQ-
RVLESLTRFLANRLKLKVNVDKSAVAR---PW-------------------------------VRKFLGYSM-
>DS_B.h.I1/AP001507/130149..132031/Bacillus halodurans/Bacterial C
GVDEMDV--------------------KS-LRL---HLHE--NWTSIRN---EIIE------GSY---
FPKPVRRVEIPK---P--------------------NG---G-------VRKLGIPTVMDRFLQQAIAQILT-
--QLYDPT-FSERSFGF---RPHRR-----GHNAVRQAKQW----M----KE-------------G-------
YRW---VVDIDLEKFF-DKVNHDRLMRKL--SS--R-IQ---------DPR-VLQLIR--RYLQTGV---M--
---E--R--
------------------------------------G-------------LVSPN----TE------------
----
-------------------------------GTPQGGPLS-PLLSNIVLD-ELD-------------------
-----NEL-EK--------------------------------------RG---------LK-----------
------------------------------FVRYADDCNI--YV------RSKRA----GL-
RIMESVTSFIENRLKLKVNREKSAVDR---PW-------------------------------NRKFLGFSF-
>DS_Bacillifid|45221880|locus|VBIGeoSp94955_0342| [Geobacillus sp. Y412MC52]
GIDGVST--------------------DQ-LRD---YIRA--HWSTIRA---QLLA------GTY---
RPAPVRRVEIPK---P-G-------------------G---G-------TRQLGIPTVVDRLIQQAILQELT-
--PIFDPD-FSPSSFGF---RPGRN-----AHDAVRQAQGY----I----QE-------------G-------
YRY---VVDMDLEKFF-DRVNHDILMSRV--AR--K-VK---------DKR-VLKLIR--AYLQAGV---M--
---I--E--
------------------------------------G-------------VKV-Q----TE------------
----
-----------------------------E-GTPQGGPLS-PLLANILLD-DLD-------------------
-----KEL-EK--------------------------------------RG---------LK-----------
------------------------------FCRYADDCNI--YV------KSLRA----GQ-
RVKQSIQRFLEKTLKLKVNEEKSAVDR--P-W-------------------------------KRAFLGFS--
>DS_S.th.I1/AP006840/1010793..1012672/Symbiobacterium thermophilum/Bacterial C

GVDGVPT--------------------ER-LRD---QIRV--EWSRIRE---ELLQ------GTY---
RPQPVRRVEIPK---P--------------------GG---G-------KRMLGIPTVMDRLIQQALLQVLT-
--PIFDPT-FSESSYGF---RPGRR-----GHDAVRKARQY----V----EE-------------G-------
YDW---VVDMDLEKFF-DRVNHDVLMARV--AR--R-VT---------DKR-VLRLIR--RYLQAGV---M--
---L--N--
------------------------------------G-------------VVVAT----EE------------
----
-------------------------------GTPQGGPLS-PLLANILLD-DLD-------------------
-----KEL-ER--------------------------------------RG---------HH-----------
------------------------------FVRYADDCNI--YV------RSKRA----GE-
RVYRSVRHFLQERLRLKVNEEKSAVDR---PW-------------------------------KRQFLGFSF-
>DS_clostridiafid|22104403|locus|VBIHelMod36755_0020| [Heliobacterium
modesticaldum Ice1]
GIDGMGL--------------------ES-LRP---YLKE--EWSRIKQ---ELLE------GTY---
RPQPVRRVEIPK---P-Q-------------------G---G-------TRKLGIPTVVDRLIQQALNQILM-
--PIFDPD-FSTNSYGF---RPGKS-----AHQAVKKAKEY----I----AD-------------G-------
YRW---VVDMDLAQFF-DRVNHDILMARV--AR--K-VK---------DKR-ILKLIR--EYLKAGV---M--
---L--N--
------------------------------------G-------------IRV-K----SE------------
----
-----------------------------E-GTPQGGPLS-PLLANIILD-DLD-------------------
-----KAL-ES--------------------------------------RG---------HR-----------
------------------------------FCRYADDCNV--YV------RSRRA----GQ-
RVMEGMAKFLEGRLKLQVNWEKSAVDR--P-W-------------------------------NRKFLGFSF-
>DS_clostridiafid|20880100|locus|VBIAmmDeg104956_0293| [Ammonifex
degensii KC4]
GVDGMEP--------------------EA-LRS---YLKE--HWPRIKE---ELLA------GTY---
RPMPVRRVEIPK---PG--------------------G---G-------VRLLGIPTVLDRLIQQALLQVLT-
--PIFDPG-FSPHSYSF---RPGRS-----AHQAVEQARRY----V----AQ-------------G-------
YRH---VVDLDLAQFF-DRVNHDLLMARV--AR--K-VK---------DKR-VLKLIR--AYLQAGV---M--
---I--N--
------------------------------------G--------------CCVR----TE------------
----
-----------------------------E-GTPQGGPLS-PLLANIILD-DLD-------------------
-----KEL-ER--------------------------------------RG---------HR-----------
------------------------------FVRYADDSNV--YV------KSCRA----GQ-
RVFESLKRFLEQRLKLRINEEKSAVDY---AW-------------------------------RRGILGFSF-
>DS_fid|102053344|locus|VBIDesTol47847_3092| [Desulfobacula toluolica
Tol2]

----MDLSKFF-DRVNHDRLMSRL--AT--R-IK---------DKR-VLKLIR--KYLTAGT---M-----I-
-N---
-------------------------------G-------------LVVFS----TE-----------------
----
--------------------------GTPQGGPLS-PLLSNIVLD-ELD------------------------
KEL-ER--------------------------------------RG---------LR----------------
-------------------------FVRYADDFVI--YL------KSKKA----AQ-
RVMESIKRFITLKLKLKVNEEKSSVGN---AW-------------------------------RSKFLGFSF-
>DS_fid|102051933|locus|VBIDesTol47847_2395| [Desulfobacula toluolica
Tol2]
GIDNMTV--------------------DQ-LPG---YLRR--HWPKVKG---KLLQ------GNY---
KPLPVKRKEIPK---P--------------------DG---G-------VRLLGIPTVLDRLIQQAVSEILQ-

```
--QIWDPH-FSESSHGF---RPGRS-----QHDAILQGKVY----L----LS-------------G-------
YTH---SVNMDLSKFF-DRVNHDRLMSRL--AE--R-IK---------DKR-VLKLIR--SYLTAGV---M--
---I--D------------------------------------------------------------------
------------------------------------G-------------VVVSA----AE------------
-------------------------------------------------------------------------
-------------------------------GTPQGGPLS-PVISNIVLD-ELD-------------------
-----KEL-EK--------------------------------------RG---------HK-----------
------------------------------FVRYADDFVI--YL------KSKKA----AE-
RVMKSVTRFITVKLRLKVNEEKSKVSR----PWLDK------------------------------FLGYTF-
>DS_clostridiafid|32495095|locus|VBITheIta22270_0082| [Thermoanaerobacter
italicus Ab9]
GVDGMGV--------------------DE-LLP---YLKE--NWATIKQ---QLLE------GKY---
KPQPVRRVEIPK---P--------------------DG---G-------KRLLGIPTVLDRLIQQAIAQILN-
--KVYNHT-FSDSSYGF---RPGRS-----AKDAIKAAEAY----I----NE-------------G-------
YTW---VVDMDLEKFF-DRVNHDIIMSKL--EK--R-IG---------DKR-VLKLIR--RYLESGV---M--
---I--N------------------------------------------------------------------
------------------------------------G-------------IKVST----EE------------
-------------------------------------------------------------------------
-------------------------------GTPQGGPLS-PLLANIMLD-ELD-------------------
-----KEL-EK--------------------------------------RG---------HK-----------
------------------------------FCRYADDCNI--YV------RSRSA----GN-
RVMKSIKKFIESKLKLKVNEAKSAVDR---PW-------------------------------RRKFLGFSF-
>DS_Bacillifid|102073184|locus|VBIAmpXyl149409_0044| [Amphibacillus
xylanus NBRC 15112]
GIDQMPT--------------------TD-LRV---YVME--NWHTMRE---QLLS------GTY---
QPQPVRRVEIPK---P--------------------NG---G-------VRKLGIPTVTDRLIQQAIAQQLT-
--LVFDPT-FSEFSYGF---RPNRR-----AHTAVKQARAY----I----EE-------------G-------
YRW---VVDMDLEKFF-DKVHHDRLMARL--AT--R-IK---------DKV-LLHLIR--QFLQAGV---M--
---E--N------------------------------------------------------------------
------------------------------------G-------------LVSPM----TE------------
-------------------------------------------------------------------------
-------------------------------GTPQGGPLS-PLLSNIVLD-ELD-------------------
-----KEL-EK--------------------------------------RG---------HK-----------
------------------------------FVRYADDFHI--YV------KSSRA----GE-
RVMESITTFIEKKLRLKVNREKSAVDR---PW-------------------------------KRKLLGFS--
>DS_Bacillifid|20956876|locus|VBIAnoFla45531_2524| [Anoxybacillus
flavithermus WK1]
GVDMMPV--------------------QN-LRT---HIVE--NWQSIKE---AIIK------GTY---
EPMPVRRVEIPK---P--------------------DG---G-------VRLLGIPTVTDRLIQQAIAQVLS-
--KIYDPM-FSEHSYGF---RPNRS-----AHDAVRKAQGY----I----KE-------------G-------
YRW---VVDIDLEKFF-DQVNHDRLMSTL--AK--R-IH---------DKP-LLKLIR--KYLQSGV---M--
---I--H------------------------------------------------------------------
------------------------------------G-------------VVSST----EK------------
-------------------------------------------------------------------------
-------------------------------GTPQGGPLS-PLLSNIVLD-ELD-------------------
-----KEL-EK--------------------------------------RG---------HQ-----------
------------------------------FVRYADDCNI--YV------KSKRA----GE-
RTMASVQRFIERKLRLKVNEKKSAVDR---PW-------------------------------KRKFLGFS--
>DS_O.i.I1/BA000028/2785523..2787411/Oceanobacillus iheyensis/Bacterial C
GIDEMSV--------------------KF-LRR---HLYD--NWDSLRE---NLRK------GTY---
TPSPVRRVEIPK---P--------------------SG---G-------VRMLGIPTVTDRFIQQAIAQVLH-
--TIFDPS-FSEHSYGF---RPNRR-----GHDAVRKARGF----I----KE-------------G-------
YRW---VIDMDLEKFF-DKVNHDKLMGVL--AK--R-IK---------DKE-LLRLIR--KYLQSGV---M--
---I--N------------------------------------------------------------------
```

------------------------------------G-------------IVVSS----EE------------
----
-------------------------------GTPQGGPLS-PLLSNIILD-DLD-------------------
-----KEL-EE--------------------------------------RG--------LR------------
------------------------------FVRYADDCNI--YV------RTKKA----GN-
RVMNSITTFIEEKLRLKVNKEKSAVDR---PW-------------------------------KRKFLGFSF-
>DS_Bacillifid|21969758|locus|VBIGeoSp101709_0522| [Geobacillus sp.
WCH70]
GTDGMSV--------------------KD-LRR---HLVE--HWDVIRR---ALEE------GTY---
EPCPVRRVEIPK---P--------------------NG---G-------VRLLGIPTVTDRFIQQAIAQVLT-
--PIFDPS-FSEHSYGF---RPGRR-----GHDAVKKAKQY----I----QE-------------G-------
YTW---VVDIDLEKFF-DRVNHDKLMGIL--AK--R-IP---------DKI-LLKLIR--KYLQAGV---M--
---I--N--
------------------------------------G-------------VVMET----QE------------
----
-------------------------------GTPQGGPLS-PLLSNILLD-ELD-------------------
-----KEL-EK--------------------------------------RG--------HK------------
------------------------------FVRYADDCNI--YV------RTKKA----GE-
RVMKSITAFIEKKLRLKVNETKSAVDR---PW-------------------------------RRKFLGFS--
>DS_Bacteroidetesfid|54587919|locus|VBIPorAsa172508_0403| [Porphyromonas
asaccharolytica DSM 20707]
GIDGMKV--------------------EE-LRD---YMNA--NWTSIKQ---SILE------RRY---
KPAPVRRVEIPK---P-----------------N---G---G-------VRKLGIPTVVDRTLQQSIVQVLT-
--PIFEAE-FQENSYGF---RPGRS-----CEQAVQKLLEY----L----NE-------------G-------
AEW---IVDIDLEKFF-DNVPQDKLMSYV--GR--V-IH---------DPD-TESLIR--KYLKSGV---M--
---E--N--
------------------------------------G---------L---YE--A----TE------------
----
-----------------------------L-GTPQGGNLS-PLLSNVMLN-ELD-------------------
-----KEM-VR--------------------------------------RG----------L-----------
--------------------------R---YVRYADDCVI--AV------RSEAS----AK-
RVMHSVTQWIERVLGLKVNATKTHVCR----P----------S--------------------KLKYLGFGF-
>DS_S.ag.I1/AJ292930/182..2038/Streptococcus agalactiae/Bacterial C
GIDGVTI--------------------EQ-MDD---YLHQ--NWRETKK---LIKE------RSY---
KPQPVLRVEIPK---P-----------------N---G---G-------VRNLGIPTAMDRMIQQAIVQVLS-
--PLCEKH-FSEYSYGF---RPNRS-----CETAIVQLLEY----L----ND-------------G-------
YEW---IVDIDLEKFF-DTVPQDRLMSLV--HN--I-IQ---------DGD-TESLIR--KYFHSGV---V--
---I--N--
------------------------------------G---------Q---RH--K----TL------------
----
-----------------------------V-GTPQGGNLS-PLLSNIMLN-ELD-------------------
-----KGL-EK--------------------------------------RG----------L-----------
--------------------------R---FVRYADDCVI--TV------GSEAA----AK-
RVMHSVSSYIEKRLGLKVNMTKTKIVR----P----------N--------------------KLKYLGFGF-
>DS_clostridiafid|42987381|locus|VBIEubRec155814_2884| [Eubacterium
rectale M104/1]
GVDGMKY--------------------TE-LKE---HLAK--NGETIKG---QLRT------RKY---
KPQPARRVEIPK---P-----------------D---G---G-------VRNLGVPTVTDRFIQQAIAQVLT-
--PIYEEQ-FHDHSYGF---RPNRC-----AQQAILTALNI----M----ND-------------G-------
NDW---IVDIDLEKFF-DTVNHDKLMTLI--GR--T-IK---------DGD-VISIVR--KYLVSGI---M--
---I--D--
------------------------------------D---------E---YE--D----SI------------
----
-----------------------------V-GTPQGGNLS-PLLANIMLN-ELD-------------------

-----KEM-EK--------------------------------------RG----------L-----------
--------------------------N---FVRYADDCII--MV------GSEMS----AN-
RVMRNISRFIEEKLGLKVNMTKSKVDR----P----------S--------------------GLKYLGFGF-
>DS_Bacillifid|201988153|locus|VBIBacThu93926_0157| [Bacillus
thuringiensis YBT1518]
GVDGVTV--------------------DE-LKQ---YLKE--NKDELRQ---RIRT------RKY---
QPQAALRVEIPK---E-----------------N---G---K-------MRKLGIPTVVDRVVQQAIHQILS-
--PIFEKQ-FSEFSYGF---RPKRS-----CEMAIVKSLEF----L----NA-------------G-------
YEW---IVDIDLERFF-DTVHHDKLMRII--SN--T-IS---------DGD-VISLIR--KYLVSGV---M--
---V--N--
------------------------------------G---------K---YE--E----TS------------
----
-----------------------------V-GTPQGGNLS-PLLSNIMLN-ELD-------------------
-----KEL-ES--------------------------------------RE----------L-----------
--------------------------Q---FVRYADDALI--FV------KSEKA----AS-
RVMKSIVRFIEKNLGLIVNTEKSKISR----P----------E--------------------DLKFLGFG--
>DS_Bacillifid|102075684|locus|VBIAmpXyl149409_1227| [Amphibacillus
xylanus NBRC 15112]
GVDGMSV--------------------EQ-IQG---YLAL--NKDDLLK---SIRN------RTY---
KPEPVLRVEIPK---P-----------------N---G---G-------VRLLGIPTVKDRVIQQAIAQILT-
--PLFDRQ-FSDYSYGF---RPRRY-----AEMAIIKGLEY----M----ND-------------G-------
YEW---IVDIDLERFF-DTVNHDRLMNLV--AR--T-VE---------DGD-VISLIR--KFLVSGV---Q--
---I--D--
------------------------------------E---------E---YK--E----TI------------
----
-----------------------------I-GTPQGGNLS-PLLSNIMLH-ELD-------------------
-----MEL-EN--------------------------------------RG----------L-----------
--------------------------R---FVRYADDCII--FA------KSQMA----AN-
RIMRSITRFIEEKLGLIVNADKSKVTN----P----------N-NT-----------------DFKFLGFG--
>DS_Bacteroidetesfid|47240589|locus|VBISphSp165585_3447|
[Sphingobacterium sp. 21]
-------------------------------QD---YLVE--NKDELIT---AILR------GRY---
RPNPVRRVAIPK---D-----------------N---G---Q-------QRQLGIPTVVDRFIQQAIAQVLL-
--PLYEPQ-FSEHSYGF---RPRRN-----AHHALKQCRDY----I----TA-------------G-------
YSY---AVDLDIEKFF-DQVNHSKLIEVL--SG--T-IK---------DGR-VLSLIH--KYLNAGV---Q--
---V--G--
------------------------------------G---------S---YE--R----SE------------
----
-----------------------------M-GVPQGGPLS-PLLSNIML------------------------
----
-------------------------------VRYADDLLL--MC------KSKRS----GQ-
RVMGSLISFIENKLHLKVNRDKSQTAP----V----------S--------------------RVKFLGYSF-
>DS_Fl.jo.I1/CP000685/4416242..4418139/Flavobacterium
johnsoniae/Bacterial C
GIDKLST--------------------EH-LQE---WLLK--HKESLIE---SLEK------GKY---
KPQAVRRVEIPK---E-----------------G---G---K-------TRSLGIPTVVDRLVQQSIIQILT-
--PIYEQE-FHTSSHGF---RPKRG-----CHTALKEVESH----L----ND-------------E-------
YCY---VVDLDLEKFF-DTVNHSRLIELL--SK--K-VK---------DPR-VISLIH--KYLRAGV---V--
---V--V--
------------------------------------N---------K---FE--E----SV------------
----
-----------------------------L-GVPQGGPLS-PLLSNIMLH-ELD-------------------
-----KEL-AR--------------------------------------RG----------H-----------

--------------------------R---FVRYADDCLI--FC------KSKRA----CL-
RVKESITAFIESVLYLRVNKEKTTVGY----I----------R--------------------GKKFLGYSF-
>DS_B.t.I3/AE015928/3254698..3258524/Bacteroides
thetaiotaomicron/Bacterial C
GIDKMSC--------------------EQ-LLS---WLKA--NKDELII---SLQS------GTY---
RPNPVRRVEIPK---D-----------------N---G---K-------KRLLGIPTVVDRLVQQAINQVLT-
--LIYERQ-FSKTSYGF---RPQRG-----CHDALRKAQKI----V----SE-------------G-------
YIY---VVDLDLERFF-DTVSHSKLIEIL--SR--T-IK---------DGR-VISLIH--KYLRSGV---I--
---N--R--
------------------------------------G---------M---FE--M----ST------------
----
-----------------------------E-GTPQGGPLS-PLLSNIMLN-ELD-------------------
-----KEL-ER--------------------------------------RG----------H-----------
--------------------------P---FVRYADDAMI--FC------KSKRA----AK-
RVRESITLFIEGKLFLKVNHEKTVVSY----V----------K--------------------GVKFLGYSF-
>DS_D.h.I4/AP008230.1/5193183..5195085/Desulfitobacterium
hafniense/Bacterial C
GIDGMQV--------------------DE-LLP---FLEN--HKDELVK---SLWD------GKY---
RPKPVRRVEILK---E-----------------N---G---K-------MRKLGIPTVVDRLIQQAITQVLS-
--PIFEEQ-FSDSSFGF---RPKRS-----AHDALRRCQSH----I----NG-------------G-------
YRY---VVDMDLEKYF-DTVNQSKLIQIL--SE--T-IK---------DGR-VISLIH--KFLQSGV---M--
---V--D--
------------------------------------G---------L---FE--E----SP------------
----
-----------------------------E-GVPQGGPLS-PLLGNIMLN-ECD-------------------
-----HEL-ER--------------------------------------RG----------H-----------
--------------------------R---FVRYADDMMI--FC------KSKKA----AK-
RTLDHILPYIEGKLFLKVNREKTKVAH----V----------N--------------------YVKFLGYSF-
>DS_clostridiafid|22668882|locus|VBINatThe92436_0159| [Natranaerobius
thermophilus JW/NMWNLF]
GIDGMGV--------------------DE-LLQ---YLKE--NGDHLRQ---RVLD------GKY---
RPNPVRRVEIPK---E-----------------D---G---K-------KRKLGIPTVVDRVIQQAIAQVLS-
--PIYEEQ-FSDNSYGF---RPGRS-----THDAIKKSQQN----I----NE-------------G-------
YKY---VVDMDLEKYF-DTVNQSKLIEVL--SK--T-IK---------DGR-VISLIN--KYLRAGV---M--
---I--K--
------------------------------------H---------T---YK--D----TE------------
----
-----------------------------V-GVPQGGPLS-PILSNIMLH-ELD-------------------
-----KEL-EK--------------------------------------RG----------H-----------
--------------------------E---FVRYADDLLI--FC------KSRRS----AG-
RTLKNILPFIENKLFLKVNKDKTVVAY----V----------G--------------------KVRFLGFGF-
>DS_clostridiafid|43183350|locus|VBIRumTor148568_2143| [Ruminococcus
torques L214]
GNDGMQV--------------------DE-LLP---FLRE--NQDTLIR---KIRE------GKY---
KPNPVRRVEIPK---E-T---------------E---G---E-------FRKLGVPTVVDRVIQQAIAQELS-
--PVYEKQ-FSENSFGF---RPKRG-----AHDALRQCQKN----V----ND-------------G-------
YVY---VVDMDLEKFF-DTVCQSKLIEVL--SR--T-IK---------DGR-VISLIH--KYLNAGV---I--
---A--K--
------------------------------------G---------M---FE--R----TE------------
----
-----------------------------V-GMPQGGPLS-PLLSNVMLN-ELD-------------------
-----KEL-ES--------------------------------------RG----------H-----------
--------------------------R---FVRYADDCMI--FC------KSRKS----AE-
RTLKNIIPFIEGKLFLKVNRKKTEVSH----I----------S--------------------KVKYLGYSF-

>DS_clostridiafid|42783608|locus|VBIButBac39087_0859| [butyrateproducing bacterium SM4/1]
----MQV--------------------DE-LLP---YLIN--HRDELVR---QLRE------GKY---
KPNPVRRVEIPK---E-E---------------K---G---K-------FRKLRIPTVVDRMIQQAIAQELT-
--PIYEEQ-FSDNSYGF---RPGRS-----AHDALAKCRKY----V----DE-------------G-------
HVY---AISMDLEAYF-DTVNHSKLIEVL--SR--T-MK---------DGR-VISLIH--RYLNAGV---M--
---E--D--
------------------------------------G---------G---FH--A----TP------------
----
-----------------------------E-GVPQGGPLS-PLCGNVMLN-ELD-------------------
-----KEL-ER--------------------------------------RG----------H-----------
--------------------------K---FVRYADDCII--LC------KSRKS----AE-
RTLKHIIPFITEKLYLKINLEKTTVSH----I----------S--------------------KVKYLGYGF-
>DS_Bacillifid|38119357|locus|VBIBacSel78655_0161| [Bacillus selenitireducens MLS10]
GVDGMTV--------------------DQ-LEA---HVRQ--YAKPLIA---KIQK------GTY---
QPLPVKRVEIPK---E-----------------N---G---K-------KRKLGIPAVRDRMVQQAIFQVIE-
--PIIDPH-FSPNSYGF---RPGKN-----AKQAIKQAAKY----Y----DE-------------G-------
FKM---VVDIDLKSYF-DTIPHQKLMNYL--EQ--Y-IQ---------DPI-ILKLIW--KFLKSGI---M--
---I--G--
------------------------------------D---------N---WE--S----SR------------
----
-----------------------------N-GAPQGGNLS-PILSNVYLH-ELD-------------------
-----KEL-ER--------------------------------------RG----------H-----------
--------------------------R---FVRYADDFCI--YV------KSRRA----AE-
RVLLNTTTFLEGTLKLSVNQEKSAIGS----P----------T--------------------KRKFLGFC--
>DS_Bacillifid|45159896|locus|VBIBacCel7049_2030| [Bacillus cellulosilyticus DSM 2522]
GIDGMSV--------------------DE-LLP---YLAL--EDRNLIL---SIKD------GSY---
RPQPVKRVEIKK---P-----------------D---G---G-------KRKLGIPTVKDRLVQQMILQVIE-
--KKIDPQ-FSDNSYGF---RPNRS-----AHDAMRKAKQY----Y----EE-------------G-------
FRY---VVDIDMKQYF-DTVNQDKLMHHV--EQ--F-ID---------DPT-VLILIR--KFLRSGI---S--
---I--D--
------------------------------------E---------E---IE--P----SE------------
----
-----------------------------V-GTPQGGNLS-PILGNIYLH-QLD-------------------
-----LEL-ER--------------------------------------RG----------H-----------
--------------------------K---FIRYADDCNI--YV------KSRKA----GD-
RVLKSITKFLEEELKLTVNKDKSEVGR----P----------T--------------------KRKFLGFC--
>DS_clostridiafid|115615696|locus|VBIDehSp228777_0406| [Dehalobacter sp. CF]
GVDGMTV--------------------DE-MLP---WLRK--HREELLQ---SLGN------GMY---
RPQPVRRVEIPK---P-----------------D---G---G-------VRKLGVPTVIDRMVQQALVQILQ-
--PIFEPL-FSEASYGY---RPGRS-----AQQAMKEAKEY----Y----EQ-------------G-------
YTR---AADIDLSKYF-DTMNHELLMNII--RK--E-VK---------DKR-IIDLIK--KFLKSGV---M--
---E--N--
------------------------------------G---------V---KS--K----TE------------
----
-----------------------------E-GSPQGGPLS-PLLSNIYLN-EFD-------------------
-----KEM-ER--------------------------------------RG----------H-----------
--------------------------K---HLRYADDIAV--YT------KSRRA----AE-
RVLESCKQYLEKKLKLKVNSEKSKAGS----P----------L--------------------KLKFLGFAL-
>DS_Bacillifid|87094488|locus|VBILacPen232155_1085| [Lactobacillus pentosus KCA1]

GVDGMTI--------------------DQ-LPE---YTRK--HRKELLE---SLRN------GTY---
RPQPVRRVEIPK---P--------------------DG---S-------TRKLGVPTVIDRMIQQAVVQVLS-
--PIYEQV-FSDNSYGF---RPGRS-----AHDAIKSVTSL----Y----NQ-------------G-------
YHY---VVDLDLKAYF-DTVNHDLLMNFI--QQ--Q-VT---------DPW-LLHLIR--RFLTSGV---M--
------N--
------------------------------------G-------------KLFQD----TT------------
----
-----------------------------E-GTPQGGNLS-PLLANIYLN-ELD-------------------
-----TLL-AQ--------------------------------------RG---------HQ-----------
------------------------------FVRYADDCNI--YV------KSKRA----GE-
RVLRNVTAFLENRLKLTINRHKTTVGS-------PL---------------------------RLKFLGFT--
>DS_Bacillifid|43088904|locus|VBILacSal150030_1121| [Lactobacillus
salivarius CECT 5713]
GIDEMTV--------------------DE-LFQ---YLRE--NKEELTT---SLRE------GSY---
KPLPVKRVEIPK--LN---------------------G---G-------TRKLGIPTVIDRMVQQAVAQVLT-
--PIFEEI-FSENSFGF---RPNRG-----AQDAIDKVISY----Y----NQ-------------G-------
YKR---VVDLDLKSYF-DNVNHDLMIKYL--QQ--Y-ID---------DEW-TLKLIR--KFLTSGI---L--
---D--N--
------------------------------------G-------------LFVKS----EK------------
----
-------------------------------GTPQGGPLS-PLLANIYLN-ELD-------------------
-----KEL-TK--------------------------------------RG---------HR-----------
------------------------------FVRYADDCNI--YV------KSQRA----GE-
RVMRSITKFLEKQLKVKVNTDKTRVGS-------PI---------------------------KLKFLGFS--
>DS_La.re.I1/AY911856/603..2512/Lactobacillus reuteri/Bacterial C
GIDDMTV--------------------ND-LLP---YLRE--NKTELIA---SLRE------GKY---
KPAPVKRVEIPK---P--------------------NG---G-------VRKLGIPTVVDRMVQQAVAQILT-
--PIFERV-FSDNSFGF---RPHRG-----AHDAIAKVVDL----Y----NQ-------------G-------
YRR---VVDLDLKAYF-DNVNHDLMIKYL--QQ--Y-ID---------DPW-TLRLIR--KFLTSGV---L--
---D--H--
------------------------------------G-------------LFAKS----EK------------
----
-------------------------------GTPQGGPLS-PILANIYLN-ELD-------------------
-----KEL-TR--------------------------------------RG---------HH-----------
------------------------------FVRYADDCNI--YV------KSQRA----GE-
RVMRSITQFLEKRLKVKVNPDKTKVGS-------PL---------------------------RLKFLGFSL-
>DS_cianobacteriafid|115659900|locus|VBIChaMin231992_1871| [Chamaesiphon
minutus PCC 6605]
GIDGITK--------------------KT-LAA---ESAKTEFVNALRT---ELQT------KQF---
RPMPVRRVYIPK--SN---------------------G---K-------QRPLGIPTLKDRTVQMLLKMVLE-
--PIYESD-FLNCSNGF---RPQRR-----TQDCIARLDSY----I----NR------------------
RNKYYW---VIEGDIKAAF-DSIHHQILLKIM--AK--R-IA---------DNR-LLKLVE--SFLKAGI---
M--------E---
---------------------------------------G---------H---LFK-H----TD---------
----
--------------------------------I-GTPQGGICS-PLLANIYLH-QLD----------------
--------LYWWQK-------------------------YGNLDRKEKERRRTR---------HQ--------
-----------------------------GNCALIRYADDWLL--LT-----NGSKAE----AM-
RLKEEFSIFLKEELQLELSLEKTHITH----V--ND---------------------------GIDFLGFHI-
>DS_clostridiafid|61456050|locus|VBISulAci142080_3112| [Sulfobacillus
acidophilus DSM 10332]
GVDHQSC--------------------DQ-FAE---HLGE--ELDRLGQ---AMRE------HRY---
QPLPVRRIWIPK---P---------------------G-TRK-------QRPLGVPAIRDRVAEEAMRRVRE-
--PIGEPT-FSPDSYGF---RPGRS-----AHDAVHRIFDH----L----AH-------------G-------

YHW---VVDADIQDYF-GSIDQQLLIDKV--AE--R-IS---------DGT-VLGWIR--DMLRAGV---M--
---DAGQ--
--WHATP------------------

-----------------------------R-GTRQGSVIS-PLLANIYLD-ALD-------------------
-----QAMAKL--------------------------------------PG---------VQ-----------
------------------------------FIRYADDWCA--LA------RTKEE----AE-
AALTTAQTVLD-ELRLTLHPEKTRIVD----V--RE--------------------------TAFDFLGFTF-
>DS_clostridiafid|43053635|locus|VBIHalPra106773_0208| [Halanaerobium praevalens DSM 2228]
GVDRIDT--------------------VE-FKE---NYAV--HMRELYR---EFLE------DRY---
QPKPALRVFIPK--SD---------------------G---R-------QRPLGIPTVKDRIAQAAVRGILE-
--PIYEKE-FCDCSLGF---RKGKS-----QIDAINKIEEY----K----EQ-------------G-------
YKW---VLDADIKGFF-DNINHELLIEFI--RQ--K-VT---------DGW-VIEIIK--SWLTMGV---M--
---K--D--
------------------------------------G-------------EYI-P----KE------------
----
-----------------------------K-GTPQGGVIS-PLLANIFLH-EFD-------------------
-----KIM-VE--------------------------------------RG---------YK-----------
------------------------------LVRFADDFVV--MT------KSKRK----AK-
RAYEVVKEIITEKLKLELHPEKTVITN----F--GE---------------------------GFVFLGFEF-
>DS_clostridiafid|115343859|locus|VBIHalHal149681_1092| [Halobacteroides halobius DSM 5150]
GIDGVEV--------------------EE-FRE---NYTK--NMSALYR---QLTE------DRY---
EPQPVLRTYISK--GN---------------------G---E-------QRPLGIPVIKDRIAQQAVKQILE-
--IHFEEI-FCDCSYGF---RPNRS-----TEDAIKKVEEY----K----EQ-------------G-------
YNW---VLDADVKSYF-DTIDHEILMELI--AE--E-VS---------DGW-ILDIIR--SWLTIGV---M--
---TE-Q--
------------------------------------G-------------RE--E----TT------------
----
-----------------------------E-GTPQGGVIS-PLLANIYLH-HFD-------------------
-----KKM-TR--------------------------------------RG----------Y-----------
--------------------------K---IVRFADDFII--MA------KSKAK----AE-
RALEVTRQIIENELNLRLHPRKTVITN----F--DD---------------------------GFKFLEFRF-
>DS_Mx.xa.I1/CP000113/2433780..2435766/Myxococcus xanthus/Bacterial F
GQDGITF--------------------EH-IEE--RGRAG--FLGAVAE---ELRT------GTY---
RPRPYRRREIPK---E-G-------------------G---K-------VRVISIPSIRDRVVQGALRLVLE-
--PIFEAD-FSGSSFGA---RPGRS-----AHEAIDTVRQG----L----RR-------------R-------
RHR---VVDVDLKAYF-DSIRHAPLLERV--AR--R-VQ---------DGE-VLALVK--QFLRSTG------
----
--D------------

-----------------------------R-GIPQGSPLS-PLLANIALN-DLD-------------------
-----HVL-DR-------------------------GRGFL-T------------------------------
------------------------------YARYLDDMVV--LA------PDSEKGRRWAA-
RALERIRQEAE-ALGVSLNKEKTRTVT----M--TD------R-NA-----------------SFAFLGFDF-
>DS_Sg.ce.I1/AM746676.1/9205316..9207294/Sorangium cellulosum/Unclassified
GVDGVTF--------------------EQ-IET--AGRGE--FLAGLAA---ELRG------RTY---
RPAPLRRREIPK---E-G-------------------G---K-------ARVISIPTIRDRVVQAALRMILE-
--PIFEAD-FSDSSYGA---RPGRS-----AHQALKEVREG----L----RR-------------R-------
QHR---VVDVDLSRYF-DTIRHDRLLAKV--AR--R-VC---------DDE-VLALIK--QFLVRTG------
----
--E------------

-----------------------------R-GVPQGSPLS-PLLANVALN-ELD-------------------
-----QAL-NR-------------------------GKALL-T------------------------------
------------------------------YVRYLDDMVV--LA------PDSLRGRAWAD-
RALERIREEAE-AIGVSLNTDKTRVVT----L--TD------R-DA-----------------VFTFLGFDF-
>DS_Bfid|58553039|locus|VBICupNec201015_1883| [Cupriavidus necator N1]
GIDDLSF--------------------ED-IEA----SGRIVFLAEIQA---DLKT------GRY---
EPKPNRRVEIPK--SN---------------------G---K-------VRVLQVPCIRDRVVQGALKLILE-
--AVFEAD-FCPNSYGF---RPKRS-----PHRALAEVRRS----V---------------------------
LRRMSTVVDVDLSRYF-DTIQHSTLLGKI--AK--R-IQ---------DPQ-VMHLVK--QVIKAA-------
----
------------------------------------G------------------------------------
----
-----------------------------KVGVPQGGPFS-PLAANIYLT-EID-------------------
------WMLDE--------------------------------IRRKTAQG---------PY-----------
--------------------------EAVNYHRFADDIVI--TV------SGHHTKRGWAE-
RALLRLREQLV-PLGVELNTEKTTVVD----TLHGE---------------------------AFGFLGFDL-
>DS_B.th.I3/DQ363750/30070..32039/Bacillus thuringiensis/Unclassified
GIDGKSF--------------------ADIELE---GVIP--FLTGIQE---ELQA------GIY---
QPQANRKVEIPK--TN---------------------G---K-------MRTLQIPCIRDRVVQGALKLILE-
--AIFEAD-FCPNSYGF---RPKRS-----PHQALAEVRRS----I---------------------------
LRRMTIIIDVDLSRYF-DTIRHNILLEKI--AK--R-VQ---------DPQ-VMHLVK--QVIKAT-------
----
------------------------------------G------------------------------------
----
-----------------------------KIGVPQGGPFS-PLAANIYLN-EVD-------------------
-----WTF-DT--------------------------------IRRKTADG---------NY-----------
--------------------------EAVNYHRFADDIVI--AV------SGHSSKSGWAE-
LALRRLWEQLK-PLGVELNLEKTQMVN----VLKGE---------------------------SFGFLGFDL-
>DS_W.e.I5/AM999887.1/284826..286812/Wolbachia endosymbiont/Unclassified
GIDGVTF--------------------ESIETE---GSRK--YLQRIRH---ELIT------KTY---
SPNRNRRKEIPK---S---------------------G--EK-------FRTLNIPCIRDRIVQTALKLILE-
--PIFESD-FQKGSYGY---RPKRN-----AHEAVQKVTEA----AIKGNTK---------------------
------VIDVDLKSYF-DSVRHHILMEKI--AK--R-IN---------DKE-IMRMIK--LILKIG-------
----
------------------------------------G----------------------KR------------
----
-------------------------------GMAQGSPLS-PLLSNIYLN-EVD-------------------
-----KML-EK--------------------------------AKEVTKEG---------KY-----------
--------------------------QRMEYARWADDLVI--LI------REYPKREWLER-
AVYRRLEEELA-KLEVRVNEEKTKVIN----LKKGE---------------------------TFSFLGFDF-
>DS_gi|302392022|ref|YP_003827842.1| Acetohalobium arabaticum DSM 5501
GVDGITF--------------------ED-IEG----IGVLKYLKKIRE---ELVN------ETY---
KPQENRKQEIPK--GN---------------------G---K-------VRVLGIPTIKDRIVQGALKLILE-
--PIFEAD-FQESSYGY---RPKRT-----AHQAVKKIEKA----IVSGKRK---------------------
------VIDLDLSSYF-DTVKHHILLAKI--AK--R-VI---------DKE-VMHLIK--LMLKAS-------
----
------------------------------------G----------------------KE------------
----
-------------------------------GVPQGGVIS-PLFANLYLN-EVD-------------------
-----RML-ER-------------------------------AKEVTKSKG---------KY-----------
--------------------------TELEYARFADDIVI--AV------SSHPSMNWLLS-
KVIQRLKEELD-KIKVKVNKEKTKVVN----LEKGE---------------------------RISFLGFTL-
>DS_Ma.sp.I2/CP000471/2464047..2465973/Magnetococcus sp./Unclassified

GIDGVTF--------------------EA-IEE--SGVEQ--FLGEVRK---ELVS------GSY---
RPLKNRRKAIPK--GD---------------------G---K-------ERVLGIPSIRDRVVQGALKLILE-
--PIFEAD-FQSGSYGY---RPKRM-----AHQAVNRVAIA----I----AQ-------------G-------
KTQ---VIDADLKSYF-DTVQHDLALRKV--SE--R-VD---------DDQ-VMHLLK--LIFKTS-------
----
------------------------------------G----------------------KR------------
----
-------------------------------GVPQGGVIS-PLISNLYLN-EVD-------------------
-----KML-ER---------------------------------AKEVTRK--------GKY-----------
--------------------------THIEYARFADDLVI--LV------DGHHRWNGLAR-
KVYQRLGEELA-KLKVQLNLEKTRVVD----LTRGE---------------------------DFTFLGFNI-
>DS_gi|258515071|ref|YP_003191293.1| Desulfotomaculum acetoxidans DSM 771
GIDGITF--------------------DN-IEA--SGIEI--FLQQIQK---ELIS------GTY---
WPTQNRRKEIPK--GD---------------------G---K-------YRILGIPTIRDRVVQGALKLILE-
--PIFEAD-FQEGSYGY---RPKRN-----PHQAIDRVAKA----VVENKTR---------------------
------VIDLDLRSYF-DTVRHDLLLKKV--AK--R-VN---------DEN-VMRLLK--LILKAS-------
----
------------------------------------G----------------------KR------------
----
-------------------------------GVPQGGVIS-PLLANLYLN-EVD-------------------
-----KML-EK---------------------------------AKEVTRH--------EQY-----------
--------------------------THIEYARFADDIVI--LI------DAYPKWNWLEK-
AVYQRLLEELT-KLDVQLNEEKTRIVN----LANGE---------------------------SFGFLGFDF-
>DS_Actinobacteriafid|115168014|locus|VBIMycSme175119_3779|
[Mycobacterium smegmatis JS623]
GIDKRTA--------------------RG-IEASADGVAG--FLEQLRE---ALRS------GTF---
RPVPVRRVEIPK--AS---------------------G---K-------VRKLGIPTVADRVVQASLKLVLE-
--PVFETD-FSDSSYGF---RPRRR-----AQDAIEDIRMF----A----HR-------------G-------
YEW---VFEADIAACF-DEIDHSALLQQV--RG--R-IG---------DKR-ILGLVK--AFLKAGV------
---LDTD--
------------------------------------G-------------DTY-D----TF------------
----
-----------------------------T-GTPQGGILS-PLLANIALS-VIDDHFDT--------------
-----QWAAHR---------------------------NGSARRSHRQHGG----------------------
--------------------------ATYRLVRYADDFVV--LV-----YGEREH----AE-
QLWEHMSDLLA-PMGLRLAPDKTQVVH----I--DE---------------------------GFDFLGFRL-
>DS_Rh.js.I1/CP000433/185762 187934/Rhodococcus
jostii/Unclassified/Unclassified
GVDGMTV--------------------AD-VEA---SVGMSGFLDDLRT---QLKD------GSF---
RPLPVRERKIPK---P---------------------GGSGK-------VRKLGIPTVADRVVQAALKLVLE-
--PIFEAD-FLPVSYGF---RPKRR-----AHDAVAEIQYF----G----TK-------------G-------
YRW---VLDADIEACF-DSIEHTALMGRV--RE--R-VK---------DKR-VLLLVK--SFLKAGV------
---MSET--
------------------------------------G-------------HHEDN----PT------------
----
-------------------------------GTPQGGILS-PLLANIALS-VLD-------------------
-----EHVHGP--------------------WQPGGAMSTPTGRALRRRRG----------L-----------
--------------------------PNWRIVRYADDFVV--LV-----FGSCDD----VN-
DLREEIADVIA-PLGLRFSESKTRVVH----M--GE---------------------------GFDFLGFRL-
>DS_gi|317124020|ref|YP_004098132.1| Intrasporangium calvum DSM 43043
GVDGVTV--------------------RQ-IRQ---RGEVGVFLAGIAA---SLRD------GTY---
RPAPVRRVLIPK---P---------------------G---G------KSRPLGIPTVTDRVVQQSLRMVLE-
--PIFEAD-FLPVSYGF---RPKRR-----AHDAVAEIHFY----A----GR-------------G-------
YRW---VLDADIEGCF-DHIDHTALLGLV--RE--R-IK---------DKK-TVALVR--AFLKAGVLSDL--

```
-------------------------------------------------------------------------
------------------------------------G-------------LEA-A----AG------------
-------------------------------------------------------------------------
-----------------------------E-GTPQGGIIS-PLLANIALS-VLD-------------------
-----EAIMAP--------------------WAQGGDQSTQTGRAKRRYHG----------L-----------
--------------------------GNWRIVRYADDFVI--MT------NGSRD----
DVLALKEQAAEVLA-RVGLRLSESKTRVTH----L--SE---------------------------
GIDFLGFHI-
>DS_Ca.ac.I1/CP001700/4538431 Catenulispora acidiphila/Unclassified
GIDGRTV--------------------SR-IEG--QGVEE--FLAGLRE---SLKS------GEF---
WPVPVKERMIPK--AN---------------------G---K-------LRRLGIPTVADRVVQAALKLVLE-
--PIFEVD-FEPCSYGF---RPNRR-----AHDAIAEIHHY----A----SR-------------G-------
YEW---VLEGDIEACF-DNIDHTALMGRV--RE--R-VG---------DKR-VLRLIK--AFLKSGI---F--
---S--E------------------------------------------------------------------
------------------------------------G-------------RAV-R----DT------------
-------------------------------------------------------------------------
-----------------------------RTGTPQGGILS-PLLANVALA-VLD-------------------
-----EHF-AQ--------------------VWQETGRTWAARDWHRRRGG----------------------
--------------------------ATFKLVRYADDFVI--LA-----YGSRQH----VE-
DLTADVAQVLS-TVGLRLSPTKTAVAH----I--DE---------------------------GFDFLGFRI-
>DS_gi|336177663|ref|YP_004583038.1| Frankia symbiont of Datisca
glomerata
GVDGRTA--------------------AS-IVA---RIGIPEYLDGLRS---ALKD------RSF---
RPLPVRERMIPK---A---------------------G---G----K--LRRLGIATITDRVVQASLKLALE-
--PIFEAD-FLPCSYGF---RPMRR-----AHDAVAEIRYL----T----SK------------------
PRCYEW---IVEGDIKACF-DEISHTSLTGRV--RA--R-IG---------DRR-VLALVK--AFLKSGI---
L-----VEDR---------------------------------------------------------------
-----------------------------------------------------LVR-P----TT---------
-------------------------------------------------------------------------
--------------------------------A-GTPQGSILS-PLLSNVALS-VLD----------------
--------EHV-AR---------------------SPGGPGTGKTEKAKRLRHG----------L--------
-----------------------------PNFKLVRYADDWCL--VI-----KGTKAD----AE-
ALREEIAGVLS-TMGLRLSREKTLITH----I--DD---------------------------GLDFLGWRI-
>DS_gi|296169325|ref|ZP_06850953.1| Mycobacterium parascrofulaceum ATCC
BAA614
GADGTAP--------------------RS-VGA----AEAVGLLQRLRE---ELKE------RIF---
RPDPVREVMIPK--AN---------------------G---K-------LRRLGIATVADRVVQASLKLVLE-
--PIFEAD-FHPCAYGF---RPGRR-----AQDAIAEIHHL---------AS-------------GSRA----
YHW---VFEGDITACF-DEISHSALMGRV--RR--R-VG---------DKR-VLALVK--SFLKAGI---L--
---SKDL------------------------------------------------------------------
------------------------------------G-------------YRD-T----IT------------
-------------------------------------------------------------------------
-------------------------------GTPQGGILS-PLLSNVALS-VLD-------------------
-----EHFAAK------------------------WKALGPEWTRAKHRRA---------GV-----------
--------------------------PTMKIVRYADDFCV--MV-----HGTRAD----AE-
ALWDEIAAVLA-PMGLRLSVEKSRICH----V--DE---------------------------GFEFLGFRI-
>DS_gi|260905481|ref|ZP_05913803.1| Brevibacterium linens BL2
GVDGVAP--------------------RS-LLHG--QAVE--VLTMIRR---QVKT------GEF---
RPLPVRERRIPK--SN---------------------G---K-------TRSLGIPTLADRVVQASLKLVLE-
--PIFEAD-FYPSSYGF---RPRRR-----AQDAIAEIHKFTSRPL-------------------N-------
YEW---VFEADITACF-DEIDHTGLIQRL--RG--R-IT---------DKR-VLALVR--RFLKAGI---L--
---SE-D------------------------------------------------------------------
----------------------------------------------G---VNR-N----TH------------
-------------------------------------------------------------------------
```

```
-----------------------------T-GTPQGGILS-PLLANIALS-GLD-------------------
----------------------DHF-QKKWE-----SLGPSWTRAKLRRRG----------I-----------
--------------------------PVMKLIRYADDFVVLVH-------GSVEH----VE-
ALWHEVAEVLA-PMGLRLSVEKTKVTH----I--DE---------------------------GFDFLGWRI-
>DS_fid|42684501|locus|VBIStiAur43712203747_2581| [Stigmatella aurantiaca
DW4/31 (Prj:54333)]
GVDGLTA--------------------RK-VVA-K-GVDT--FIDEVRK---ELRS------GAY---
RPCPVRRVLIPK---P-G---------------QP--W---K-------FRPLGIPTVRDRVVQAAVKNILE-
--PIFEAD-FFPSSYGF---RPGRS-----AHAALEELRKL----L----LPQHANTEAGTEIRLP-------
YQW---AIEGDIKGCF-DNIDHHGLMERV--RR--R-VG---------DTK-VNRLIV--AFLKAGV---M--
---A--E------------------------------------------------------------------
-------------------------------------------------------------------------
------------------------------------EQ--------F---LR-------SS------------
-------------------------------------------------------------------------
-----------------------------T-GTPQGGILS-PLLANIALA-
VIDERYERHVWPRRTPTLLHDTRMVQLRAA-QN-------------------------RNNDRRSRRDGR-
LV----------L-------------------------------------V---PIRYADDFII--
LVGAKPGPGSHERARTAAL-AEKAALAALLKETLNLELSEAKTAITP----V------------TS-------
----------PMRFLGHHV-
>DS_Bacteroidetesfid|32443874|locus|VBISpiLin97822_6935| [Spirosoma
linguale DSM 74]
GIDGMTV--------------------GS-IRQ---RIGEAPFLATLQQ---QLRT------GSY---
KPSPCRRKLIPKAGKP---------------------G---K-------FRPLGIPTIADRVVQSAIKQVLE-
--PILEAR-FWPVSYGF---RPGRG-----CHGALEHIRMS----M----RPRKVNKQDNKRHEMP-------
YQW---VIEGDIQSCF-DHIDHHQLMDRI--RQHSA------------DRR-VNQLLV--QFLKAGI------
---LSEE------------------------------------------------------------------
--------------------------------------------------QFLRT----DA------------
-------------------------------------------------------------------------
-------------------------------GTPQGGIVS-PLLANVALG-LIEERYE---------------
-----RWVNHQ--------------TKRRQSRQCDGIKAAMWSRSVDRQAG----------R-----------
--------------------------AVYFPFRYADDFVI--LV-----SGTQEN----AQ-
AERKVLQTLLQEKMGLTLSPEKTKITP----L--TE---------------------------GFQFLGHRV-
>DS_Sr.me.I2/AE006469/1065613..1067822/Sinorhizobium
meliloti/Unclassified
GVDGMTV--------------------GR-IRN----RSEHRFLVDLQA---DLRS------GAY---
RPSPARRKLIPK-AGK--------------------PG---Q-------FRPLGIPTIRDRVVQGAAKILLE-
--PIFEAQ-FWHVSYGF---RPGRN-----THGALEYIRRA---ALPQKRDE-------------
DTRRNRLPYPW---VIEGDIKGCF-DNINHHHLLERM--RK--R-IG---------DRR-VVRLVG--
LFLKAGV---L--------------------------------------------------------------
-----------------------------------------------------------T-EDQFL-R----
TD-----------------------------------------------------------------------
-------------------------------------------A-GTPQGGIIS-PLLANIALS-AIEERYE-
-------------------RWTYHR--------------KKTQARRKSNGVAAAASARDSDRIAG--------
--R-------------------------------------CVYLPVRYADDFVV--LV-----SGSLEE----
AM-AEKSALADYLIKTTGLTLLPEKTKVTA----M--TE---------------------------
GFEFLGFRF-
>DS_gi|354961371|dbj|BAL14050.1| Bradyrhizobium japonicum USDA
GADGITF--------------------AQIETE---GRER--WLENVRQ---ELTA------GDY---
RPQPLLRVWIPK--SN---------------------G---G-------RRPLSIPTVKDRTVMTAAMLVIG-
--AIFEAD-LLENQYGF---RPKVD-----AKMAVRRVFWH----I----RD---------------------
HRRSE-IVDADLRDYF-TSIPHAPLMKCL--TR--R-IA---------DGR-LLSMIK--GWLTVAV------
---IEKD------------------------------------------------------------------
------------------------------------G-------------RRITRTAEART-------------
-------------------------------------------------------------------------
----------------------------KKRGTPQGSPLS-PLLANLYFRRFLL-------------------
```

```
-----AWR-----------------------------------------HG---------HQ-----------
------------------------DQLDAHIVNYADDFVI--CC------RPGS-----SE-
TAMARMQTLMN-RLGLEVNDTKTRLAR----V--PE---------------------------SVTFLGYTI-
>DS_My.va.I1/CP000511/2360134..2362120/Mycobacterium
vanbaalenii/Unclassified
GVDRVTL--------------------VA-VEE--YGVDR--MLRELRH---DLRE------GVY---
CPAPARRVEIPK---P-R-------------------G---G-------TRPLGIPTVRDRVAQAAAKIVLE-
--PIFEAD-FMSCSYGF---RPKRS-----ATQAMERLRVG----F----IE-------------G-------
SQF---VVEFDIANFF-GEIDHDRLLAEV--SR--R-VS---------DRR-VLKLLR--LWLQAGV---M--
---V--D------------------------------------------------------------------
------------------------------------G---------V---VS--R----TV------------
-------------------------------------------------------------------------
-----------------------------A-GTPQGGVIS-PLLANIYLH-VLD-------------------
-----TEL-AR------------------------------------R-NV----------G-----------
--------------------------E---LVRYADDGVV--LC------RSAAQ----AE-
HALAAVGEILA-SLGLRLHPDKTKVVD----L--RE------G-GE-----------------GLDFLGCHF-
>DS_Rh.sp.I1/CP000432/23005..25058/Rhodococcus sp./Unclassified
GIDRITL--------------------EE-VEE--YGVAR--LLDELAV---ELKE------GSY---
RPLPARRVFIPK---P-G-------------------T-V-E-------QRPLSIPSVRDRIVQAAWKLVAE-
--PVFEAD-FLPCSFGF---RPRRG-----AHDALQVLIDE----S----WR-------------G-------
CRW---VVETDIANCF-EAIPIEKLMQAV--EE--R-VC---------DQP-FLKLLR--VMLRAGV---M--
---E--E------------------------------------------------------------------
------------------------------------G---------Q---VR--R----PV------------
-------------------------------------------------------------------------
-----------------------------T-GTPQGGVAS-ALLCNVYLH-RLD-------------------
-----RAW-DV------------------------------------D-EH----------G-----------
--------------------------V---LVRYADDALV--MC------RSRRQ----AE-
AALTRLRELLA-DLGLEPKEAKTRIVH----L--RV------G-GE-----------------GVDFLGFHH-
>DS_Actinobacteriafid|115244729|locus|VBIMycCan270121_2577|
[Mycobacterium canettii CIPT 140070010]
-------------------------------------------------------------------------
-------------------------------------------------------------------------
-------------------------------MLIDE----S----WQ-------------G-------KRW--
-VVETGIANCF-SGIPQEKLMQAI--EE--R-VS---------DQG-VLRLLR--AMLRAGV---M-----Q-
-D-----------------------------------------------------------------------
-------------------------------G---------S---VR--R----EA-----------------
-------------------------------------------------------------------------
------------------------S-GTPQGGPLS-PLLYNVYLH-RMD------------------------
RVW-DT------------------------------------E-EH----------G----------------
---------------------V---LVRYCDDLVV--MC------RSREQ----AE-AALQRLTVLLG-
DLGLAPKASKTRIVH----L--VE------G-GQ-----------------GVDFLGFHN-
>AC_fig|867904.9.peg.1159|Metho_1143| [Methanomethylovorans hollandica
DSM 15978 | 867904.9]
GVDSISV--------------------ADIKKT---GVDE--FLQSLKE---DIKE------NRY---
EPDRILKHEILK---P--------------------NG---K-------VRKLGILTVKDRVVQYNMKLVLE-
--PIFEAD-FDSSSFGF---RANRS-----AQLASLEVYKW----L----EA-------------G-------
NHY---VFKSDISQCF-DNIPHELLMKRI--KT--R-IK---------DRQ-VRRIIR--AWLNVNS---
ACLLNEEATY---------------------------------------------------------------
---------------------------------------G----------------------KD---------
-------------------------------------------------------------------------
--------------------------------K-GILQGGIIS-PLLLNIYLD-QFD----------------
--------DER-EN----------------------------IGLKSIRSDGQG----------H--------
---------------------------------LVRYADDFVI--LS------KQPID----TK-----
PVEGSLK-KIGLELNPEKTYQTH----I--ED---------------------------GFEFLGFYF-
```

>AC_fig|1434110.4.peg.2496|MSHOH_1962| [Methanosarcina horonobensis HB-1 = JCM 15518 | 1434110.4]
GVDSLTI--------------------QQ-VEA--SGIEN--FIRAVKK---ELEI------NRY---
AADEVRRVEIPK--RN---------------------G---E-------TRQLGILTLKDRLVQGAVKLVLE-
--PIFEAD-FENCSYGY---RAYRS-----AKLASLEVYKW----L----EA-------------G-------
STH---YLKGDIEDCF-DSIPHDKLMKIL--KT--R-IE---------DKL-ILSLVQ--DWLKKGS---Q--
---AGSS--
------------------------------------G---------K-ASGK--A----SG------------
----
-----------------------------K-GLLQGGIIS-PLLVNFYLD-QFD-------------------
-----NHWTEI---------------------------------GLKNVEG----------E-----------
--------------------------SIEHLVRFSDDFVV--LS------KEWID----PE-----
RIKAFMG-DLGLELNKEKTYVGT----A--AN---------------------------GFEFVGFYF-
>AC_fig|1434118.4.peg.2864|MSSAC_2234| [Methanosarcina siciliae C2J | 1434118.4]
GFDSLTV--------------------QQ-VEA--SGVEN--FIRSVKK---ELEK------GRY---
TADAVKRVEIPK--RN---------------------G---E-------TRQLGILTLKDRLVQGAVKLVLE-
--PIFEAD-FENCSYGY---RAYRS-----AKLASLEVYKW----L----EA-------------G-------
NTH---YLKGDIEGCF-DNIPHDKLMKIL--KT--R-IG---------DEL-ILSLVE--SWLKKGS---F--
---ESSS--
------------------------------------G-------------KN-------SN------------
----
-----------------------------K-GLLQGGIIS-PLLVNFYLD-QFD-------------------
-----NHWAEI---------------------------------GLKNVEG----------E-----------
--------------------------SIEHLVRFADDFVV--LS------KEWIE----PE-RAETIMA----
-ELGLELNREKTYVGA----A--AN---------------------------GFEFVGFYF-
>AC_fig|1434102.4.peg.2173|MSWH1_1689| [Methanosarcina sp. WH1 | 1434102.4]
GVDSLTI--------------------RQ-VEE--SGVEE--FLQTVRE---ELKT------KQY---
RADNIRRVDIPK---E-D-------------------G---S-------QRHLGIMTVKDRLVQGAMKLVLE-
--PIFEAD-FENCSFGF---RPSKS-----TKLASLEVYKW----L----ET-------------G-------
LNQ---VVKGDIKNCF-DNIPHDKLMDCL--KV--R-IE---------DKY-VLSIIE--SWLKTGV---V--
---E--A--
------------------------------------D---------S---VF--Y----PK------------
----
-----------------------------K-GVPQGGIIS-PLLVNIFLD-QFD-------------------
-----KNW-KR-------------------------DVFETHA---GE-PG----------E-----------
--------------------------R---LVRYADDFVV--LG------KNWVD----FA-H----
IRAVLA-DLGLEVNREKTTVNN----I------------KK-----------------GFEFLGYSF-
>AC_fig|1434100.4.peg.3809|MSMTP_2849| [Methanosarcina sp. MTP4 | 1434100.4]
GVDFLSI--------------------RQVEEA---GVEE--FLRTVRE---ELKT------ERY---
RAENVRRVDIPK--DS---------------------G---G-------KRHLGIMTVKDRLVQGAVKLVIE-
--PIFEAD-FEDSSFGF---RPCRS-----TRLASLEVYRW----L----ET-------------G-------
MNR---VVKGDVKDCF-DNIPHEKLMDCL--KT--R-IG---------DKY-VLSLIE--SWLKVGV---V--
------K--
------------------------------------G-------------GSVSY----PE------------
----
-----------------------------K-GVLQGGVIS-PLLVNIYLD-QFD-------------------
-----KCW-KR-----------------------------EVFAGDAGEPG----------ER----------
------------------------------LVRYADDFVV--LG------KNWVE---------
FARIRKVLA-DLDLEVNKEKTLISN----L--EK---------------------------GFEFLGYYF-
>DS_So.us.I2/CP000473/3231872..3233814/Solibacter usitatus/Bacterial F

```
GVDGVTI--------------------EEIMKT---DQGVAGFLEGIEN---SLRR------KTY---
RPEAVQRVYIEK--EN---------------------G---K-------LRPLGIPTVRDRVVQMATLLILE-
--PIFEAD-FLDCSYGF---RPGRS-----AHQALEEIRGH----V----EA-------------G-------
YQA---VYDADLKGYF-DSIPHTQLLACV--RM--R-VV---------DRS-VLKLIR--MWLEAPVVERE--
---EGGG------------------------------------------------------------------
------------------------------------G-------------SKWSR----PE------------
-------------------------------------------------------------------------
-----------------------------K-GTPQGGVAS-PLLANLYLH-WFD-------------------
-----ALFYGP------------------------------------EGPG---------GK-----------
--------------------------ADAKLVRYADDFVV--MA------KQMGT-------
ETIEFIESRLEGKFQLEINREKTRVVD----L--RE------------------------EGASLDFLSHTF-
>DS_Ge.ur.I1/CP000698/1525569..1527641/Geobacter uraniireducens/Bacterial
F
GVDGVSI--------------------ES-IEVRADGISG--YLDEIQE---SLRT------KNY---
KPSPVRRVYITK---P--------------------NG---K-------LRPLGIPCVRDRIVQAAVLLILE-
--PIFEVD-FLDCSHGF---RPKRR-----PHGALDQVGNN----L----QL-------------G-------
RQE---VYDADLSSYF-DSIPHEHLIVEL--ER--R-IA---------DRS-VLKLIR--QWLHSPV--RE--
---E--D------------------------------------------------------------------
------------------------------------G---------S---ISR-P----KQ------------
-------------------------------------------------------------------------
-------------------------------GTPQGGVIS-PLLANIYLH-RLD-------------------
-----RAFHEE-----------------------------------ADSPY---------HF-----------
--------------------------ARARMVRFADDFVV--MA------RHMGN-------
RITGWLEEKLETDLGLSINRDKTGIVR-MNKK---E---------------------------SLNFLGFTL-
>AC_fig|115547.10.peg.1390| [uncultured archaeon | 115547.10]
GVDNQTL--------------------DDIREE---GIEQ--LLEQIQH---ELKT------GTY---
RASCVRRVFIPK--SS---------------------G---K-------LRPLGIPTVKDRIVQQAVKLIIE-
--PIFEAD-FLEFSYGY---RPNRS-----AKDASLEIYKW----L----NY-------------G-------
LTN---IVDVDIEGFF-DHIDHELLLKFV--KE--R-VT---------DGY-ILSLIK--QWLKAGI---V--
------Y------------------------------------------------------------------
------------------------------------G-------------KSVTN----PT------------
-------------------------------------------------------------------------
-----------------------------E-GTPQG--VS---------------------------------
-------------------------------------------------------------------------
------------------------------FLR----------------------------------------
--------------------------------------------------------------
>AC_fig|115547.10.peg.767| [uncultured archaeon | 115547.10]
GVDGETI--------------------EDIENR---GVDQ--FLTEIQQ---QLRM------KTY---
RIPKVKRVFIPK--GD---------------------G---K-------LRPLGIPTIRDRVVQQAVKSIIE-
--PIFEAD-FKDCSFGY---RPGRS-----AMQASEKIRHL----L----NL-------------G-------
YTN---IVDMDIKGFF-DHIDHEKMVFSV--MK--R-IT---------DPY-VIKLIR--EWLRAGI------
-------------------------------------------------------------------------
--------------------------------------------------VFQGNTSYPEQ------------
-------------------------------------------------------------------------
-------------------------------GTPQGGVIS-PLLANIYLN-ELD-------------------
-----SLW-TR--------------------------------------RG-----MESPLK-----------
--------------------------HSAHLVRYADDLLA--LT------NKDPQ-------
AVAETLERIIS-LLGLEPNREKSSVIT----A--ED---------------------------GFDFLGFHFI
>DS_gi|344200432|ref|YP_004784758.1| Acidithiobacillus ferrivorans
GVDGERF--------------------ED-VEA--YGVER--WIGELAE---TLRK------KMY---
QPQAVKRVYIPK---P--------------------GG---K-------MRPLGIPTLRDRVVQTATMMVIE-
--PIFEAD-LQPEQYAY---RAGRN-----ALTAVREVHSL----L----KT-------------G-------
HKQ---VVDADLSSYF-DTIPHAELMKSV--AR--R-IV---------DRH-LLHLIK--MWLDAPV------
---EGGD------------------------------------------------------------------
```

------------------------------------G----------RGNMQRTTVNRDQG------------
----
-----------------------------R-GTPQGAPIS-PLLSSLYMRRFIL-------------------
-----GWK-QR---------------------------------GYEERFG---------SR-----------
------------------------------IVCYADDLVI--CC------RWQAE-------
QAMAAMQDMMG-RLKLTVNAEKTRICR----V--PE--------------------------AYFDFLGYTF-
>DS_D.a.I1/CP000089/759875..761862/Dechloromonas aromatica/Bacterial F
GVDRQDF--------------------ED-VEA--YGVRR--WLEELAL---ALKE------ESY---
RPDPIRRVFIPK---A-N-------------------G---K-------LRPLGISTLHDRVCMTAAMLVLE-
--PIFEAD-LPDEQYAY---RPGRN-----AQQAAEEVKNR----L----YL-------------G-------
QTD---VVDADLSDYF-GSIPHSELMKSL--AR--R-IV---------DRR-VLHLIK--MWLECAV---E--
---E--T--
------------------------------------DQRGRKKRTTE---AK--D----QG------------
----
-----------------------------R-GIPQGSPIS-PLLSNLYMR-RFV-------------------
-----LAW-KK-------------------------LG-LE-R----S-LG----------S-----------
--------------------------R---IVTYADDLVI--LC------K-CGK----AE-
EALQWMRTIMG-KLKLTVNEEKTRICQ----V----------P-AG-----------------TFDFLGYSF-
>DS_gi|296163794|ref|ZP_06846488.1| Burkholderia sp.
GVDRQDF--------------------AE-VEA--YGVQK--WLGELAL---ALRL------ETY---
RPDSIRRVFIPK--AN---------------------G---K-------LRPLGISTLRDRVCMTAAMLVLE-
--PIFEAD-LPPEQYAY---RPGRN-----AQQAVIEVEER----L----HR-------------G-------
QTD---VVDADLADYF-GSIPHAEMMLSL--AR--R-IV---------DRR-VLHLIK--MWLECPV------
---EETD--
----------------------------------DRG-------------RQKRTTEARDS------------
----
-----------------------------RRGIPQGSPIS-PLLANVYMR-RFV-------------------
-----LAW-KK--------------------------------LGLQRSLG---------SR-----------
------------------------------IVTYADDLVI--LC------KKGK-----AE-
EALLNLRQIMG-KLKLTVNEEKTRICK----VPEGE----------------------------FDFLGFTF-
>DS_Pe.th.I2/AP009389/2519125..2521096/Pelotomaculum
thermopropionicum/Bacterial F
GADGQSF--------------------KD-IEE---KVGVERFLKEIAE---ELRN------GTY---
RPMPVRRVYILK---P--------------------DG---S-------QRPLGIPTIKDRIAQMACLTVIQ-
--PIFEAD-FLDCSYGF---RPKRN-----AHQAIGAITEN----I----KQ-------------G-------
FTA---VYDADLTKCF-DSIQHRLIMDSL--AE--R-IT---------DGK-VLRLIK--GWLEAPI------
---VEPG--
------------------------------------G-------------PKQGR----KN------------
----
-----------------------------YQGTPQGGVIS-PLLANIVLN-RLD-------------------
-----RLW-HR--------------------------------------PG------GPRER-----------
--------------------------YNARLVRYADDFVV--LA------RFIGE-------
PIKNELESIIT-SMGLNLNEKKTRILD----LNKGD---------------------------ILNFLGYSI-
>DS_Afid|115293161|locus|VBIRhiTro150571_4165| [Rhizobium tropici CIAT
899]
GVDGVTF--------------------TQ-IEA--SGVDA--WLAGLRE---ELVS------KTY---
RPDPVRRVMIPK---P---------------------G-G-G-------ERALGIPSIRCRVIQTAAKLVLE-
--PIFEAD-FEDGAYGY---RPRRS-----AVDAVKETHRL----M----CR-------------G-------
YTD---VVDADLSKYF-DTIPHSDLLKSV--AR--R-IV---------DRS-VLRLIR--LWLRAPV------
---EERD--
------------------------------------G---------D-GKRRM-TGGKSST------------
----
-----------------------------H-GTPQGGVVS-PLLSVIYMNRFLK-------------------
-----HWRLSG--------------------------------------LG---------EE-----------

--------------------------FRAHVISYADDFVI--LS------RDHAA-------
EALAWTRTVMT-KLGLSLNEAKTSVKD-ARRE-------------------------------HFDFLGYSL-
>DS_gi|354960451|dbj|BAL13130.1| Bradyrhizobium japonicum USDA
GVDGITF--------------------EQ-IDA--SGLEA--WLAGLRD---ELVT------KTY---
RPDPVRRVMIPK---P--------------------GG---G-------ERPLGIPTIRDRVVQAAAKIVLE-
--PIFEAD-FEDGAYGY---RPRRN-----AVDAVKEVHRL----M----CR-------------G-------
YTD---VVDADLSKYF-DTIPHSDLLKSV--AR--R-IV---------DRN-VLRLIK--LWLRVPV------
---EERD--
----------------------------------SNG-------------KRRMSGGKSNK------------
----
-----------------------------C-GTPQGGVIS-PLLSVIYMNRFLK-------------------
-----HWRLSG---------------------------------RCEAFHG----------Q-----------
------------------------------IISYADDFVI--LS------RGHAE-------
DALTWTKAVMT-KLGLTLNETKTSVKN-------AR-------------------------LESFDFLGYTL-
>DS_Ni.ha.I1/CP000320/75444..77354/Nitrobacter hamburgensis/Bacterial F
GVDGMTF--------------------GQ-IEG--AGVDA--WLAGLRE---DLVS------KTY---
QPDPVRRVMIPK---P--------------------GG---G-------ERPLGIPTIRDRVVQAAAKIVLE-
--PIFEAG-FEDSAYGY---RPRRS-----AIDAVKETHRL----L----CR-------------G-------
YTD---VVDADLSKYF-DTIPHADLLRSV--AR--R-VL---------DRN-VLRLIK--LWLQVPV------
---EERD--
------------------------------------G---------DGKRHMS-GGKSSTR------------
----
-------------------------------GTPQGGVAS-PLLSVIYMNRFLK-------------------
-----HWRLTG--------------------------------------RG---------EV-----------
--------------------------FHAHVISYADDFVI--LS------RGHAE-------
EALTWTRAVMT-KLGLTLNEAKTSVKN-ARRE-------------------------------GFDFLGYTL-
>DS_cKu.st.I1/CT573074.1/62738..64755/Candidatus Kuenenia
stuttgartiensis/Unclassified
GADGITF--------------------ED-VES--YGVEK--FLGEIIE---ELEN------KTY---
EPQPVLRVYIPK--TN---------------------G---K-------TRPLGIPVIKDRVVQMSVKLVIE-
--PIFEAD-FEDSSYGF---RPGRS-----AGDAVRKIKEK----L----RE-------------G-------
KTE---VFDADLSSYF-DTIPHKELLLLI--GM--R-IS---------DKN-VLHLIK--MWLKAPV---I--
---E--E--
------------------------------------G---------K-------PGGGRKN------------
----
-----------------------------KIGTPQGSVIS-PLLANIYLH-MLD-------------------
-----KAV-NR-------------------------------ENGVFYKYG---------IT-----------
------------------------------IIRYADDWVL--MA------KRIPR-------
EALDYLNRLLK-KLKLSLNEDKSKIVK-AEEE-------------------------------SFDFLGHTI-
>DS_Pe.ca.I3/CP000142.2/2649551..2651540/Pelobacter
carbinolicus/Bacterial F/ORF Sequence
GIDGVTF--------------------AA-IEE---REGVSALIAELEE---ALRS------KTY---
KPDPVKRVMIPK--AD---------------------G---S-------QRPLGIPTIRDRVAQMAVKLVVE-
--PIFEAD-FCDTSYGF---RPKKS-----AHDAVDDVAYA----M----NI-------------G-------
YTE---VIDADISKYF-DTIPHTNLMAVV--AE--R-IC---------DGA-ILHLIQ--MWLKSSV---M--
--EVGKD--
------------------------------------G-------------KKRNVGGGKGN------------
----
-----------------------------RRGTPQGGVIS-PLLANLYLH-ILD-------------------
-----RIW-ER--------------------------------------RN-------LQQR-----------
--------------------------LNARIVRYADDTVL--LC-----RRNKSD-------
EAMAVLRQILE-RLGLTLNEAKTKVVN---GY--KG---------------------------GFDFLGFSI-
>DS_gi|322417944|ref|YP_004197167.1| Geobacter sp.

GIDGVTF--------------------EA-VEE---KEGVSAFIAELED---ALRN------KTY---
QPDPVKRVMIPK--SD---------------------G---S-------QRPLGIPTIRDRVAQMAVKLVIE-
--PIFEAD-FCESSYGF---RPKRS-----AHDAVDDVAYS----M----NT-------------G-------
YTE---VIDADLSKYF-DTIPHANLMAVI--AE--R-IC---------DGA-ILHLIQ--MWLKAPI---M--
--EVDKD--
------------------------------------G-------------TKRNIGGGKGN------------
----
-----------------------------RKGTPQGGVIS-PLLANLYLH-ILD-------------------
-----RIW-ER--------------------------------GNLQQRLG---------AR-----------
------------------------------IVRYADDIVI--LC------RRAK-----AD-
KAMATLRYVLE-RLGLSLNEAKTTTVN---AY--KD---------------------------KFDFLGFTI-
>DS_gi|350554847|ref|ZP_08923874.1| Thiocystis violascens DSM
GSDGVSF--------------------EA-IEQ---GEGVEGFLKGLAE---ELRE------KRY---
RAQPVRRAMIPK--GD---------------------G---R-------ERPLGIPTIRDRVVQMAVKLVIE-
--PIFEAD-FTPHSYGF---RPQRS-----AHDAIDDIANA----L----WA-------------G-------
HTH---VIDADLSSYF-DTIPHANLMTVV--AE--R-MT---------DGA-ILALLK--QWLKAPI---I--
---GVDD--
-----------------------------------QG-------------KRRTVGGGKAN------------
----
-----------------------------RVGTPQGGVIS-PLLSNLYLH-LLD-------------------
-----RIW-DR-----------------------------------HRLKD----------K-----------
--------------------------LGAHIVRYADDFVV--LC------KQGVE-------
EPLKVVRHVTD-RLGLTLNETKTHVVD----A--KE--------------------------TGFHFLGFTL-
>DS_gi|345870111|ref|ZP_08822065.1| Thiorhodococcus drewsii
GIDGVTF--------------------TA-IEA---GIGKDAYVAALRE---ELEQ------KTY---
RADGVRRVWIPK---P--------------------DG---S-------ERPLGIPTIRDRIVQMAFKLVVE-
--PIFEAD-FCEHSYGF---RPQRS-----AHDAIDAIAEA----L----LR-------------G-------
HTQ---VIDADLSKYF-DTIPHAKLMGVI--AE--R-LV---------DGP-VLGLIR--QWLKAPV---I--
---EEDE--
-----------------------------------RG---------Q---HRP-T-GGKGN------------
----
-----------------------------RRGTPQGGVAS-PLLANLYLH-LLD-------------------
-----RIW-VR--------------------------------HDLERRLG---------AR-----------
------------------------------LVRYADDAVI--LC------RHSTE-------
KPMAVFTAVLE-KLDLTLNVQKTHVVD-ARAD-------------------------------GFEFLGFRI-
>DS_clostridiafid|61054282|locus|VBICloCla155345_1776| [Clostridium clariflavum DSM 19732]
GIDKVSI--------------------DD-VKA----YGEEKLLDEIAE---DLRA------EKY---
RCKPVRRTYIPK--QD---------------------G---R-------KRALGIPTIKDRIVQMAAKIVIE-
--PVFEAD-FQPCSYGF---RPKRN-----AKQAMDRIYEM----A----DK-------------GG------
ALW---VIDADIRDYF-GSINHDKLLLLV--KQ--R-IT---------DRR-VLKLIK--GWLKAGV---L--
---E--D--
------------------------------------G-------------QYSES----TV------------
----
-------------------------------GAPQGGVIS-PLLSNIYLN-YFD-------------------
-----VCWSKR--------------------------------------FG----------------------
--------------------------HLGELVRYADDFVI--LC------KKLSQ----AE-
EALRAVKWIMK-KLELTLHSEKTRLVD---MYFGKD---------------------------SFDFLGFNN-
>DS_S.ag.I2/AE014217/10188..12210/Streptococcus agalactiae/Unclassified
GIDDFTI--------------------EE-IEA--YGVQK--FLDEIED---QLRN------KKY---
QPKAVKRVYIPK--AN---------------------G---K-------KRPLGIPTVRDRVVQTAVKIVIE-
--PIFEAD-FQEFSYGF---RPKRS-----ANQAIREIYKY----L----NY-------------G-------
CEW---VIDADLKGYF-DTIPHDKLLLLV--KE--R-VT---------DKS-IIKLLS--LWLEAGI---M--
---EDNQ--

--VR-------SN------------

-----------------------------ILGTPQGGVIS-PLLANIYLN-ALD-------------------
-----RYW-KN-----------------------------------NRLEG---------RG-----------
--------------------------HDAHLIRYADDFVI--LC------SNNPK-------
KYYQYAKQRID-KLGLTLNEEKTRIVH----A--TE---------------------------GFDFLGYTL-
>DS_clostridiafid|115613588|locus|VBIDehSp228777_0963| [Dehalobacter sp. CF]
GIDKQTL--------------------SD-IEE--MGVEK--FLLTCQR---SLKE------NNY---
RPMPVRRQYIPK--KD---------------------G---K-------MRPLGIPVIRDRVIQMAVKLVIE-
--PIFEAD-FHESSYGF---RPKRS-----AKQALDRVRKA----C----NR-------------K-------
GNW---VCDVDIQSYF-DNINQEKLMKLV--EM--R-IS---------DKK-VLKLIR--KWFKAGV---M--
---E--E--
------------------------------------G-------------VIT------RT------------
----
-----------------------------DIGTPQGGVIS-PLLSNIYLN-VLD-------------------
-----LLW-EK--------------------------------------HG----------K-----------
--------------------------ESGELTRYADDFVI--IC------KTKKD----AD-
KAMVIVQAIMK-RLDLTLHPTKTRLVG---MWTGEE---------------------------GFDFLGMHH-
>DS_UA.I4/AY714820/20258..22206/uncultured_archaeon /Unclassified
GIDDVTI--------------------DE-FER---NLEQ--NLNEIQR---LLRQ------DRY---
VPKPVKRVYIPK---P--------------------DG---K-------QRPLGIPTIRDRVVQQALKNVIE-
--PIFEAE-FLDSSFGY---RPGKS-----AKQAIEQIETV----R----DE-------------G-------
HEW---VVDADIKAFF-DTVNHEKLIDAV--AE--R-IS---------DGR-VLGLIR--AFLEADI---M--
---EQGQ--
------------------------------------G-------------RA-------KN------------
----
-----------------------------VVGTPQGGVIS-PLLANIYLH-YFD-------------------
-----ERM---AL--------GFE-----------
------------------------------VVRYADDVLV--LC------GSEEE----AE-
EAISHVKEILE-ELELTLHPQKTKIKN----F--SE---------------------------GVDFLGFTV-
>DS_clostridiafid|42840809|locus|VBICloCf158569_1553| [Clostridium cf. saccharolyticum K10]
GMDGITF--------------------EM-IEE--YGVEE--YLLDIQE---DLQN------KQY---
RPKPVKRVYIPK---P--------------------DG---K-------QRPLGIPTIRDRIVQQACKIVIE-
--PIFEAN-FLDSSYGF---RPKRD-----AKQATEKVKKE---------LY---------------------
KNWY--AVDADIQGYF-DNINHEILLGLL--KR--R-IS---------DRR-VIKLCR--QWLQAGV---I--
---E--N--
------------------------------------G-------------KYYPT----EK------------
----
-------------------------------GSPQGGVIS-PLLANIYLH-VLD-------------------
-----SYW---KN---------HK-----------
--------------------------ELGVIVRYADDAVI--VC------RTRKD----AE-
LAFEHLKRMMT-KLKLTLNPQKTKIVD----M-NKE---------------------------SFDFLGFRY-
>DS_fid|115349142|locus|VBIThiMob160332_0325| [Thioflavicoccus mobilis 8321]
WSPTVSR--------------------DD-LQH---HLMR--HLLACRE---EVLD------GAY---
RPLPLRQFPVRK---P-D-------------------G---R-------QRVLTAQFLRDKLVQRALLTVLE-
--PRAEAL-FHDDSFAY---RPERN-----VAKALAKVRER----V----RI-------------G-------
LDW---LVDADIEKFF-DSIPHRPLLRVL-DGF-------VA------DAK-AMKLIE--RWLGQGA---H--
---VR--
--S---LL--A----TP------------

-----------------------------R-GIAQGAILS-PLFCNLYLH-GFD-------------------

-----RSL-DS--------------------------------------AH----------I-----------
--------------------------P---FVRFADDFLL--FA------PTRSD----AG-
RAMEHAARRLE-RLDLRLHPDKTRVVR----S--GR---------------------------EVIFLGETL-
>PF_WP_046007427.1 [Pseudoalteromonas rubra]
GVDCQSI--------------------AS-FES---ELQL--GLNSILY---DLRQ------QHY---
TPAALKRSQLKL---P---------------------G---K------KPRWLAFPTVRDRIVHTAIAILLQ-
--PYFEEE-FEHNSYGY---RPGRS-----YIMAVDKVIEH----R----NQ-------------R-------
RRH---VFDADIQGYF-DHIPQDKLLTKL--QATAI------------DPT-LIELIF--TLLFSFQ------
---QSND--
------------------------------------G---------L---VFG-K----AL------------
----
-----------------------------GQGIPQGSAIC-PLLANFYLD-ELD-------------------
-----EHL-NA--------------------------------------LG---------YH-----------
------------------------------MVRYADDFVV--CC------DSAKA----AQ-
HAQYHTEQVLT-HLALTLNLNKTQLTT----F--AD---------------------------GFKFLGHYF-
>PF_WP_038884984.1 [Vibrio rotiferianus]
GADGISI--------------------KE-FAS---DLDT--QLRQLHY---DWKN------NRY---
KPYRYRNITIEK---A---------------------N---K------KPRELAVPTVRDRILHSALAQKLL-
--NIFEAE-FEHISYGY---RPNRS-----YTHAIRHIEQL----R----DQ-------------G-------
YST---VIDADIQGYF-DNICHIKLTELL--NR--H-LP---------SDW-VSAITD--TLLSQQQ---A--
------D--
------------------------------------G---------H-LYFGA-E----IG------------
----
-----------------------------V-GIPQGSPLS-PLLANLYLD-GFD-------------------
-----EAL-LD--------------------------------------RG---------EQ-----------
------------------------------IIRYADDFVI--LL------PNEDR----AQ-
SCLAFVTDYLN-QLKLTLNCEKTKVVS----F--QD---------------------------GFTFLGVTF-
>W__[Vibrio_vulnificus] 37677204
GVTIQTF--------------------AIHLDT---------NLNTLLS---AWNH------GNY---
APSPYRPLTIQP--NE---------------------K---K-------TRQLAIPTVADRIIHTAIAQKLV-
--AKFEPE-FEHISYGY---RPNRS-----YTHAIRHIEQL----R----NQ-------------G-------
YLY---VLDADIKGYF-DHICHKRLKQIL--QK--Y-LE---------DNW-VESIMT--LLLSQQM------
---P--A--
--QTLLFGV-E----LG------------

-----------------------------R-GIPQGSPLS-PLLANLYLD-GFD-------------------
-----EAL-LD--------------------------------------RG---------EQ-----------
------------------------------IVRYADDFVV--LV------THEQQ----AQ-
HCLAFVTQYLA-SLKLQLNTEKTRVVS----F--QD---------------------------GFTFLGVSF-
>PF_KPQ33062.1 [Phormidesmis priestleyi Ana]
GVDGITT--------------------DL-FVG---VANE--QLAQMHR---QLRR------EVY---
EASPAKGFYVPK---K-N-------------------G---G-------QRLIALSTVRDRILQRYLLQSIY-
--PRLEKA-FTDSTFAY---RPGLS-----IYGAVDRVMAI----Y----AP-------------Q-------
PTW---VIKADIQQFF-DNLSWGVLLSQL-ERL-------KV------APA-QVRLIE--QQLKAGL---I--
---LQ--
------------------------------------G---------Q---FY--R----PN------------
----
-----------------------------K-GVLQGGILS-GALANLYLS-EFD-------------------
-----RLC-QE--------------------------------------AE----------I-----------
--------------------------P---LVRYGDDCVA--VC------HSYLQ----AN-
RFLAMMQGWLE-DIYLTLNPDKTRIVG----P--DE---------------------------GFVFLGHMF-
>PF_WP_008312855.1 [Leptolyngbya sp. PCC 6406]
GVDGITV--------------------DL-FKG---IAQE--QIRLLHQ---QMRQ------ERY---
VASPAKGFYLPK---K-T-------------------G---G-------DRLIGIPTVKDRIVQRYLLQGIY-

```
--PHLENT-FSEATFAY---RPGLS-----IYTAVAQVMTR----Y----RH-------------Q-------
PAW---VIKADIQQFF-DRLSWPLLLHQL-DQL-------PL------PPV-WMRWIE--QQLKAGI---V--
---IR--------------------------------------------------------------------
------------------------------------G---------H---FQ--R----PN------------
-------------------------------------------------------------------------
-----------------------------Q-GVLQGSILS-GALANLYLN-DFD-------------------
-----RRC-LA--------------------------------------AD----------I-----------
--------------------------D---LVRYGDDCVA--VC------QSYLE----AT-
RSLALMQDWIE-DLYLSLHPEKTQIIP----P--GE---------------------------AFVFLGHRF-
>PF_WP_024971209.1 [Microcystis aeruginosa]
GIDGIPT--------------------DL-FAG---VVDE--ELSLLQR---QLQQ------EYY---
QADPAKGFYRQK--KS---------------------G---G-------NRLIGIPTVRDRIVQRLLLHSIY-
--PALEDV-FSDRSYAY---RPGLG-----VQSAIAHLSEV----YA---GQ---------------------
TVW---TIKADVSRFF-DSLNWALLLTRL--ERLSL------------EPV-IVRMIE--QQIKSGI---V--
---I--D------------------------------------------------------------------
------------------------------------G-------------QKLRQ----TK------------
-------------------------------------------------------------------------
-------------------------------GVLQGGILS-GALANLYLS-DFD-------------------
-----ARC-----------------------------------------VG----------------------
--------------------------LNLDLVRYGDDFVI--VT------SGLLE----AT-
RVLDSLHHWLA-DIYLALQPEKTRIIA----P--DG---------------------------EFTFLGYQF-
>W__Arthrospira_platensis 479129286
--------------------------------------------------------------MGF--------
--YRVKK---S---------------------G---G-------HRLIGIPTVRDRIVQRLLLRSLY---
PILEET-FQDCSFAY---RPGVG-----VKHAIERVAEV----Y----SS---------------------
QTW---TIKADISQFF-DSLCRTLLLSQL--EELSV------------DQT-VVRYIK--GQLEAGI---V--
---V--G------------------------------------------------------------------
------------------------------------G-------------MPI-L----SG------------
-------------------------------------------------------------------------
-----------------------------R-GVLQGGILS-GALANLYLS-EFD-------------------
-----RRC-LD--------------------------------------AG---------AY-----------
------------------------------LTRYGDDFVI--VA------RSLLE----AT-
RFLNLIEDWLS-DIYLTLQPEKTHIFA----P--GE---------------------------EFVFLGYGF-
>W_[Cyanothece_sp._PCC_7822]_1 307592471
---GITT--------------------DL-FAG---VKKD--ELIRLQQ---ELIE------EIY---
QPYPARGFYLPK--NN---------------------G---D-------KRLLGIPAVRDRVVQRWLLEDLY-
--LPLEEV-FTDCSYAY---RPGRG-----IQMAVKHLYYY----Y----QI-------------Q-------
PKW---IIKSDIRSFF-DSLNWSILLSIL-EHL--K-L----------DPI-IQQLVE--QQLKSGI---V--
---LK--------------------------------------------------------------------
------------------------------------G-------------RYF-P----RN------------
-------------------------------------------------------------------------
-----------------------------Q-GVLQGAVLS-GALANLYLS-EFD-------------------
-----RKC-LE--------------------------------------KG---------IN-----------
------------------------------LVRYGDDFVA--AC------QSLGE----AE-
RTLNLITQWLE-RIYLQLHPKKTEIYA----P--DQ---------------------------EFTFLGYLF-
>PF_WP_007355619.1 [Kamptonema]
GIDNITV--------------------DL-FAG---VARY--QLQVLLW---QLQQ------ENY---
FPRPAKGFYLRK--AS---------------------G---G-------KRLIGIPTVRDRIVQRFLLDELY-
--WPLEDV-FLDCSYAY---RPGRG-----IQMAVKHLYSY----Y----QF-------------G-------
QAW---VIKADIEKFF-DNLCWPLLLTDL-EKL--Q-F----------EPT-LRQLIE--QHLASGI---V--
---VK--------------------------------------------------------------------
------------------------------------G-------------QHF-H----PN------------
-------------------------------------------------------------------------
-----------------------------Q-GVLQGGILS-GALANLYLN-EFD-------------------
```

```
-----RLC-LS--------------------------------------HG---------FN-----------
------------------------------LVRFGDDFAV--AC------ADSIQ----AN-
RCLEQINSWLG-SFYLKLQPEKTRIFA----P--DE---------------------------EFTFLGYLF-
>W_[Microcoleus_sp._PCC_7113] 428314604
---GITT--------------------DL-FAG---VAKE--QLYSLQR---QLQQ------EHY---
AAHPALGFYLRK--TR---------------------G---G-------KRLIGIPVVLDRIVQRLLLEELY-
--LPLEDT-FLDCSYAY---RPGRG-----IQMAVQHLESY----Y----QF-------------Q-------
PTW---VIKADIAQFF-DNLCHALLFTHL-EQL--Q-L----------EPI-VLQLIE--QQLKAGI---V--
---IK--------------------------------------------------------------------
------------------------------------G-------------QRL-F----PQ------------
-------------------------------------------------------------------------
-----------------------------K-GVLQGAVLS-GALANLYLT-EFD-------------------
-----RQC-LS--------------------------------------HG---------LN-----------
------------------------------LVRYGDDFVV--VA------PDWIQ----AN-
RALEQITTGLA-QLYLTLQPEKTKIFA----P--DE---------------------------EFTFLGYQF-
>W_Calothrix_parietina 428297029
---GISI--------------------GF-FES---MATE--QLRNLVS---QLQY------GTY---
TASPAKGFYVPK--KN---------------------G---G-------KRLIGIPTVRDRIIQRLLLDELY-
--FPLEDT-FVDCSYAY---RPGRN-----IQQAVQHLYRY----Y----QY-------------Q-------
PKW---IIKADIVEFF-DNICLALLLNAL-EKL--R-L----------EPN-ILQLIE--QQIKSGI---I--
---IN--------------------------------------------------------------------
------------------------------------G-------------QYQ-N----AG------------
-------------------------------------------------------------------------
-----------------------------K-GLLQGGTLS-GALANLYLT-DFD-------------------
-----QKC-LN--------------------------------------QG---------IN-----------
------------------------------LVRYGDDFVI--AC------SNFAE----AN-
RVLDKITGWLG-GVYLTLKAEKTEIFS----P--DD---------------------------EFTFLGYRF-
>W__[Nostoc_sp._PCC_7120] 17228961
---GISV--------------------DL-FES---MATE--QLQNIAY---QLKE------ETY---
TANPAKGFYIPK--KN---------------------G---T-------KRLIGIHTVRDRIIQRLLLDELY-
--FPLEDT-FLDCSYAY---RPGHS-----IQQAVQHLYGY----Y----QY-------------Q-------
PKW---IIKADVADFF-DNLSWALLLTYL-EEL--S-L----------EPS-LLQLLE--QQLKSGI---I--
---IA--------------------------------------------------------------------
------------------------------------G-------------QYR-N----FG------------
-------------------------------------------------------------------------
-----------------------------K-GVLQGGILS-GALANLYLT-SFD-------------------
-----RKC-LS--------------------------------------QG---------IN-----------
------------------------------LVRYGDDFVI--AC------NSWLE----AN-
RILDKITGWLG-EVYLTLQPEKTQIFT----P--ND---------------------------EFTFLGYRF-
>W_Calothrix_sp 427717966
---GISV--------------------EL-FES---MATE--QLQNIAN---QLYD------ETY---
TASPAKGFYIPK--KN---------------------G---S-------KRLIGIPTVRDRIIQRLLLDELY-
--FPLEDT-FLDCSYAY---RPGHN-----IHQAVQHLYGY----Y----QY-------------Q-------
PKW---IIKTDIADFF-DNLSWALLLTAL-DEL--S-L----------EPI-VLCLLE--QQLHSGI---I--
---IA--------------------------------------------------------------------
------------------------------------G-------------QYR-N----FG------------
-------------------------------------------------------------------------
-----------------------------K-GVLQGGILS-GALANLYLT-NFD-------------------
-----RKC-LS--------------------------------------QS---------IN-----------
------------------------------LVRYGDDFVI--AC------NSWQE----AN-
RILDKITTWLG-EVYLTLQPEKTQIFT----P--NE---------------------------EFTFLGYRF-
>PF_WP_029630506.1 [[Scytonema hofmanni] UTEX B 1581]
GVDGISL--------------------DL-FES---VAAE--QLRNIEY---QLHH------ETY---
TASPAKGFYVPK--KN---------------------G---D-------KRLIGIPTVRDRIVQRLLLEELY-
```

--FPLEDT-FLDCSYAY---RPGRN-----IQQAVQHLYSY----Y----QL-------------Q-------
PKW---VIKADIAEFF-DNLCWALLLTAL--EDLQL------------ESI-VLQLLE--GQLKSGI---V--
---I--A--
------------------------------------G-------------KPV-Y----PG------------
----
-----------------------------K-GVLQGGVLS-GALANLYLT-NFD-------------------
-----RKC-LS--------------------------------------HG---------IN-----------
------------------------------LVRYGDDFAI--AC------TSFHE----AN-
RILDKITTWLG-ELYLQLQPEKTQIYA----P--DD---------------------------EFIFLGYRF-
>PF_WP_033334699.1 [Scytonema hofmannii]
GVDGIDV--------------------DL-FAS---AVND--QLRILLR---QLQQ------ESY---
CASPAKGFYLAK--SS---------------------G---G-------KRLVGIPTVRDRIVQRLLLEELY-
--FPLEDT-FLDCSYAY---RPGRN-----IQQAVQHLYSY----Y----HL-------------R-------
PKW---IIKADIAEFF-DSLSWALLLTAL-EKL--P-L----------EPI-VVQLLE--GQLRSGI---V--
---IN--
------------------------------------G-------------KPI-Y----PG------------
----
-----------------------------K-GVLQGGVLS-GALANLYLN-EFD-------------------
-----KKC-LH--------------------------------------QG---------IN-----------
------------------------------LVRYGDDFAI--AC------SNWRE----AT-
RTLDKVAAWLG-ELYLNLQPEKTQIFA----P--DD---------------------------EFTFLGYRF-
>PF_WP_041039832.1 [Tolypothrix campylonemoides]
GVDGMTV--------------------DL-FAA---GVNE--QLRILLR---QLQQ------ESY---
RASPAKGFFVAK--KS---------------------G---G-------KRLIGIPTVRDRIVQRLLLEELY-
--FPLEDT-FLDCSYAY---RPGRN-----IQQAVQHLYSY----Y----QY-------------Q-------
PKW---IIKADIAEFF-DNLCWALLFTAL--EDLQL------------EPI-LLQLLE--QQLKSGI---V--
---I--A--
------------------------------------G-------------KPI-Y----PG------------
----
-----------------------------K-GVLQGGVLS-GALANLYLT-SFE-------------------
-----RKC-LS--------------------------------------YG---------IN-----------
------------------------------LVRYGDDFAI--AC------SSWLE----AN-
RILDKITTWLG-ELYLNLQPEKTQIFA----P--DD---------------------------EFTFLGYRF-
>W_Anabaenacylindric 440685177
---GISV--------------------DL-FAA---SVDE--QLTILLR---QLQQ------ESY---
HPSPAKGFYLTK--KT---------------------G---G-------KRLVGIPTVQDRIVQRLLLEELY-
--FPLEET-FVDCSYAY---RPGRN-----IQQAVQQLFSY----Y----QY-------------H-------
PTW---IIKADIAQFF-DNLCWALLLTNL-EAL--Q-L----------ESR-ILQLLE--QQLKAGI---I--
---IA--
------------------------------------G-------------KHI-N----FG------------
----
-----------------------------K-GVLQGGIIS-GALANLYLT-IFD-------------------
-----RKC-LS--------------------------------------NG---------IN-----------
------------------------------LVRYGDDFAV--AC------SSWKE----AN-
RILDKIIAWLG-ELYLTLQPEKTQIFA----P--NE---------------------------ELKFLGYRF-
>PF_WP_044448019.1 [Mastigocladus laminosus]
GVDGITV--------------------DL-FAA---SADQ--QLRIILR---QLQQ------KSY---
RASPAKGFYLTK--KS---------------------G---G-------KRLIGISTVRDRIVQRLLLEELY-
--LPLEDT-FVDCSYAY---RPGCN-----IQQAVQRLFSY----Y----QY-------------H-------
PTW---IIKADIAQFF-DNLSWALLFTGL--ETLHL------------EAI-VLELLE--QQIKSGI---V--
---L--G--
------------------------------------G-------------KYI-N----FG------------
----
-----------------------------K-GVLQGGILS-GALANLYLT-AFD-------------------

-----RKC-LS--------------------------------------HG---------IN-----------
------------------------------LVRYGDDFAV--AC------SSWTE----AN-
RILDKITTWLG-GLYLTLQPEKTQVFA----P--HE---------------------------EFTFLGYRF-
>PF_KFB76584.1 [Candidatus Accumulibacter sp. SK-02]
GADEQTL--------------------AE-FAA---DAEA--QLGLLAL---QLTQ------GSY---
RPAPARLIPVAK---P--------------------GG---G-------VRELLLPAVRDRIVQSALARYLA-
--DLLEPD-FGEASHAY---RPGHS-----VATALHRLQAL----R----DG-------------G-------
LVF---VAVCDIHHFF-DSVDHRRLFSLL--DDLPL------------ERR-LREQMK--TCVRIEV------
---ADVQ--
------------------------------------G-------------QGAWS----LA------------
----
-----------------------------R-GLAQGSPLS-PVLANLFLM-AFD-------------------
-----AAC-AR--------------------------------------AG---------LA-----------
------------------------------LVRYADDCVL--AC------ASETE----AQ-
SALAFAADALE-NIGLALNTRKSRLAS----F--AE---------------------------GFEFLGAFC-
>DS_gi|76258629|ref|ZP_00766283.1
GIDQITL--------------------HD-FAA---DWPN--QMVRLAE---ELRD------GSY---
RPLPPRRVAIAK--AS---------------------G---G-------ERAIAILTIRDRIAQRAVQQVLT-
--PLFEPL-FLDCSYGS---RLAVG-----VPEAIERVVRY----T----EQ-------------G-------
LIW---VIDGDIRAYF-DSIDHGILLGLL--RQ--R-ID---------EPA-ILHLIA--QWLAVGS---
VHTETPDETL---------------------
PDSPLVALLRRSGELIHEALNAPSDPLPTAYDYPDLSRPASPHSGIPTGLFAALSLAQPAFEIARQLTPLLKR
IGAQRLAVGGALAVGTVLLSELVHRAQASHDRR--

---GTLQGGPLS-PLLANIYLH-PFD------------------------LAM-TA-----------------
---------------------HG---------AR---------------------------------------
--MVRFVDDFVV--MC------PDRTT----AE-HTLVLVERQLA-TLRLTLNPQKTRIVA----Y--AG---
------------------------GIEFLGQAL-
>DS_gi|148657122|ref|YP_001277327.1
GLDAVTL--------------------RD-FEV---DWTR--QMAQLAD---ELQQ------GTY---
RPLPAKRVAIPK---A-S-------------------G---G-------ERAIAILAVRDRVAQRAVQQVLD-
--PLFDPC-FLDCSYGC---RPYVG-----VPDAIARVQRY----A----DQ-------------G-------
LGW---VVDADIATCF-DSLDQRVLLSLV-RQR-------ID------ELP-VLKLIA--QWLEAGV---L--
---QG-E--
------------------------------------A---------A---LP--G----
DTPPTPLQRGEAAVRRALSWGAERLHPPPPVGPYAAAMWETPGGSIGEDGWAPRQPGLESHLWTAVMLARPVI
DGARQALPYLQRIGGRRLAVAGAVAVGALALSEAAARLRHASRR-GVPQGGALS-PLLANIYLH-PFD-----
-------------------VAM-MG--------------------------------------QG--------
--L-------------------------------------R---LVRFMDDFVV--MC------ATQEE----
AE-CALQFAQRQLH-ILRLTLNAEKTHITA----Y--AD---------------------------
GIEFLGAAL-
>DS_gi|118065097|ref|ZP_01533407.1
GPDAVTL--------------------RD-FEA---DWTR--QMAQLAD---ELQQ------GTY---
RPLPAKRIAIPK---A-S-------------------G---G-------ERAIAILSVRDRVAQRAVQQVLD-
--PLFDPC-FLDCSYGC---RPHVG-----VPEAVARVQRY----A----DQ-------------G-------
LGW---VVDADIAGYF-DAIDQRVLLGLV--RQ--R-ID---------ELP-VLKLIA--QWLEAGM---L--
---PG-
DAALPDEAPATPLQHGEAVLRQVMSWGAERLPPPPTGPYAAAAWEMPGGSVDDGWTVRRSGLESHLWTAMMLA
RPAIDGARRALPYLQRIGARRLAVVGAVAVGALALSEAVAR---MHT-A----QS------------------
----
-----------------------R-GTPQGGALS-PLLANIYLH-PFD------------------------
VAM-TS--------------------------------------QG----------F----------------
---------------------R---LARFVDDFVI--MC------ATQDE----AE-RALNFAQQQLR-
VLRLELNAEKTRIAS----Y--AN---------------------------GIEFLGASL-

>DS_fid|42670984|locus|VBIRhoVan113057_1971| [Rhodomicrobium vannielii ATCC 17100]
GGDGVTI--------------------EI-FAQ---NAEV--ELEKLRA---ETLA------GIY---
RPRKVRHAIVPK---P--------------------KG---G-------ERKLTIPSVVDRILQTATMLSLG-
--QTVDHH-FSSASWAY---REGRG-----VDDALADLRRL----R----NS-------------G-------
LFW---TFDADIMQYF-DRILHKRLIDDL--FI--W-VD---------DLR-IVRLIQ--LWLRSFS---Y--
----
--WG---------------------

-----------------------------R-GIAQGAPIS-PLLANLFLH-PMD-------------------
-----RLL-EL--------------------------------------EG---------LA-----------
------------------------------SVRYADDFVV--LC------RSKAL----AQ-
KAQLIVASHLA-ARGLKLNMSKTRILA----P--SE---------------------------AFIFLGQTV-
>PF_WP_009855610.1 [Rubrivivax benzoatilyticus]
GCDGEEV--------------------EQ-FAQ---GLLG--RLHTLQA---EVAD------GRY---
VARPLRVVALPK---P--------------------SG---G-------QRLLAIPGVRDRVLQAAMAHALG-
--RRIEPT-LDEASHAY---RPGRS-----VLGALAALLAL----R----DQ-------------G-------
RST---VLKADVASFF-DRIHQPTLLAQL--RRFSA------------DPG-LLALVG--QVLAAVL---D--
---D--D--
------------------------------------G-------------ERR-L----MT------------
----
-----------------------------R-GVPQGSPLS-PLLANLYLH-PFD-------------------
-----VGM-RA--------------------------------------QG---------FQ-----------
------------------------------LIRYADDLVL--AC------LDADE----AA-
RAQDAAARALR-ELHLELNPAKTRIAS----F--VS---------------------------GFDFLGVRF-
>PF_WP_062763150.1 [Tistrella mobilis]
GGDGEGV--------------------AT-FQA---GLDL--RLARLAA---DLLG------GTY---RPGP-
---WLIA-------------------------G---G----------AVVAPVADRVVMTAVATGLP--
DPSSDGD----------------------PAAVMARLAAL----G----QQ-------------G-------
AVH---LLDGTITHVT-DLVPHDLLCERL--AALGG------------DAR-LVDLFG--MWLAVA-------
---DPED--
------------------------------------G------------------------------------
----
-----------------------------L-GIPPGLPVS-GLLARLHLG-AVA-------------------
-----ARI-AA--------------------------------------AG---------VH-----------
------------------------------LVPAAGEILV--LA------TGAAA----AE-
DARGRMLALLA-DHGLYVDVDLPRMIR----L--DQ---------------------------AGPRLGRIM-
>DS_gi|91201518|emb|CAJ74578.1 [Candidatus Kuenenia stuttgartiensis]
GADGVTI--------------------ER-YEG---NLDL--NLRIMRK---ELTE------QTY---
FPLPLLRILVDK--GN---------------------G---E-------ARALCIPSVRDRIVQAAVLQLIE-
--PVLEKE-FEECSFAY---RKGRS-----VKQAVYKVREY----Y----EQ-------------G-------
YQW---VVDADIDAFF-DSVDYSLLLLKF--KCYIH------------DPC-IQNLVG--LWLKGEV---W--
------D--
------------------------------------G-------------KTVTT----LK------------
----
-----------------------------K-GIPQGSPIS-PILANLYLD-EFD-------------------
-----EEL-TR--------------------------------------NG---------YK-----------
------------------------------LVRFSDDFII--LC------KNSGM----AK-
ESLKLTKKILE-KLLLELDEEQ--VIN----F--DQ---------------------------GFKFLGVIF-
>DS_DEfid|54439799|locus|VBIDesAce170587_1406| [Desulfobacca acetoxidans DSM 11109]
GVDGVSL--------------------GG-FKE---DLAV--NLAILGE---ELRS------GEY---
APLPLLRFLVAK--RD---------------------G---S-------PRPLSVPTVRDRVAQAAVLNSIE-
--PIFEAQ-FEEVSFAY---RKGRS-----VRQAAYRIKEL----R----DQ-------------G-------

YRF---VVDADLDAFF-DNINHELLLAKV-ANI----IT---------DPD-ILRLIG--LWVQAEV---Y--
------D--
------------------------------------G---------E-KIYM-------ME------------
----
-----------------------------K-GIPQGAVIS-PVLANLFLD-ELD-------------------
-----EGL-IR--------------------------------------KG---------YA-----------
------------------------------LVRYADDFVI--LA------RTRPE----AE-
AAMAFTEEILE-KMNLALDMEDTEITD----F--KR---------------------------GFTYLGLIF-
>DS_cianobacteriafid|21560796|locus|VBICyaSp136448_5986| [Cyanothece sp. PCC 7424]
GIDGETI--------------------EH-FAL---NLDF--NLTFLLN---SVTN------SNY---
IPQPLKQVLIPK---S-Q-------------------E---K-------WRELRIPTVRDRIVQQALLNVLY-
--PVMEER-FSDASFAY---RPNRS-----YLDAVKRAAYW----R----DL-------------G-------
YQW---VLDADIVEYF-DNISHSLLLKEV-RKT-------VD------NSG-ILCLIK--AWISAGV---S--
---TD--
------------------------------------K---------G---II--F----PE------------
----
-----------------------------K-GVPQGAVIS-PMLANIYLD-EFD-------------------
-----HRI-TQ--------------------------------------SD----------L-----------
--------------------------K---LVRYADDFLV--LS------DTEDG----IM-
RAYSQVVQLLH-FWGLKLHEEKTQITH----F--KK---------------------------GFQFLGHGF-
>DS_cianobacteriafid|115432920|locus|VBICriEpi239080_0668| [Crinalium epipsammum PCC 9333]
GVDEETT--------------------DD-FNH---NLNS--NLSQLRD---AVAN------STY---
QPLPFKQVFIPK--QK---------------------G---S-------WRELKIPTVRDRIVQQALLNVLA-
--PIMENK-FSPASFAY---RPHMS-----YINAVEQVAHW----R----DL-------------G-------
YHW---VMDADVSKYF-DSIDHQRLLIVV--RK--Y-LD---------NPG-ILCLIK--AWISAGV------
---LTKE--
------------------------------------G-------------IVRND------------------
----
-----------------------------K-GIPQGAVIS-PMLANIYLD-EFD-------------------
-----KII---SA----------------------
--------------------------SDLKLVRYADDFLV--LA------TTQER----IV-
KAYSEVEQILN-SFKLTLHPEKTQITN----F--ER---------------------------GFRFLGHGF-
>DS_cianobacteriafid|115605079|locus|VBIRivSp77222_5259| [Rivularia sp. PCC 7116]
GVDGETI--------------------SS-FAS-----NQTVNVYQLMN---SVAD------GSY---
QPFPCKQVIIPK--RN---------------------G---S-------QRELKIPTIRDRIVQQALLNVIS-
--PLMEEK-FSPVSFAY---RPNLS-----YINAVEKIADW----R----DM-------------G-------
YVW---VLDADIVKFF-DNIDHHRLLQQV--RL--H-ID---------HPG-ILCLIK--AWISVGV------
---ETRE--
------------------------------------G-------------LIL-P----QK------------
----
-------------------------------GIPQGAVIS-PILANIYLH-EFD-------------------
-----EII-SA--------------------------------------SD---------LE-----------
------------------------------IVRYADDFLV--LS------TSQER----IA-
IAKSQVIDLLD-SLGLEINTDKTQITS----F--ER---------------------------GFRFLGHGF-
>PF_WP_009625648.1 [Pseudanabaena biceps]
GVDRESV--------------------VH-FAK---NSEA--YLSQLRR---SLAS------GYY---
HPMPLRQLFIPK--KA---------------------G---G-------WRELGVPTVRDRIVQHALLNILH-
--PLLEPQ-FEACSFAY---RPGRS-----HLSAVRQIAQW----R----DR-------------G-------
YEW---VLDADVVRYF-ENILWQRLLDEV--AE--R-LA---------APE-VLSLIS--AWLSVGV------
---LSKE--
------------------------------------G-------------LMF------PQ------------

----
-----------------------------K-GISQGSAIS-PILANVYLD-DFD-------------------
-----EIV-TA--------------------------------------TG---------LK-----------
------------------------------LVRYADDFVV--MS------RSQKR----IV-
EAKDEVADLMN-GIGLQLHPDKTRIVD----F--DR---------------------------GFRFLGHAF-
>PF_WP_006515493.1 [Leptolyngbya sp. PCC 7375]

---MRVPTVRDRIVQQALLNVLH---
PVLEPQ-FEPVSFAY---RPGRS-----HKLAVEKVSAW----H----RR-------------G-------
YDW---LLDGDIVSYF-DQVEHSRLLSEV--DE--R----LGASD--FETL-ALRLIE--QWNTVGT---L--
---TS-A--
------------------------------------G-------------LV--L----PE------------
----
-----------------------------R-GIPQGSVVS-PILANVYLD-DFD-------------------
-----EAL-QA--------------------------------------SR---------FK-----------
------------------------------LVRFADDFVV--MG------RSQRQ----AE-
QAQAKVAELLT-TMGLQLHPDKTQITN----F--DR---------------------------GFRFLGHAF-
>PF_WP_017302244.1 [Nodosilinea nodulosa]
GVDGETI-----------------------YAF---GLHKSRNLTRLLQ---QVAT------STY---
RPLPLRQFFIPK--KS---------------------G---G-------WRELGVPTVRDRIVQQALLQVLH-
--PVFEVE-FEPQSYAY---RPGRS-----HRMAVERVAHW----R----SR-------------G-------
YDW---VLDADIVKYF-DTLQHPRLLAEV--KE--R-LN---------QPW-VLALLQ--GWITAGT------
---LTRE--
------------------------------------G-------------ILL------PT------------
----
-----------------------------C-GVPQGSPIS-PLLANVYLD-DFD-------------------
-----ELL-TQ--------------------------------------AG---------HK-----------
------------------------------LVRYADDFVV--LA------RTQQR----LV-
EAQTYVAQLLE-GMGLSLHPNKTQITT----F--DR---------------------------GFRFLGHAF-
>PF_WP_045442561.1 [Synechococcus sp. NKBG042902]

---MRIPAVADRIVQQALLNVLY---
PILEPE-FEVCSFAY---RPGRS-----HRMAVDQIHAF----S----RR-------------G-------
YRW---VMEADIFDYF-DHIGHRRLLAEV--AE--R----LPGQDPSFCDL-VLQLVQ--QWIAVGV---V--
---TQ-S--
------------------------------------G-------------LI--L----PQ------------
----
-----------------------------A-GIPQGAVIA-PILANVYLD-DFD-------------------
-----EAL-LR--------------------------------------TP---------LK-----------
------------------------------LVRYADDFVI--LG------QRERQ----VQ-
KILPEVAQQMA-EIGLQLNMSKTRITN----F--QK---------------------------GFKFLGHIF-
>PF_BAU44853.1 [Leptolyngbya sp. O-77]

------------MAIGH---------------------------------LVE--QWIGSGV---S-----
TA-S---
---------------------------------G-------------LI--L----PN---------------
----
--------------------------K-GVPQGAVIS-PILANVYFD-DFD----------------------
--EAI-EA--------------------------------------AG---------LK--------------
---------------------------LVRYADDFVI--LA------KSKAR----IE-RAYNLVASLLH-
AMGLELHPDKTRVTT----F--NE---------------------------GFRFLGHTF-
>W__[Rhodobacter_capsulatus] 294676823

AVDRISA--
--LRRMG------------------------
--YTW--
-VVEADIEKAF-DRIPHDPVLEAL--DTALDPAP---------GTRALIDLVG--LWLAHGS-----------
----
-------------------------------G-------------QLG-T----PG-----------------
----
------------------------R-GLAQGSPLS-PLLSNLFFD-GLD------------------------
DRF-DS---------------------------------------G--------AAR----------------
-------------------------IVRFADDFVI--LA------RSEAG----AE-EARALAEEFVA-
GHGLRMVSRETRVVG----F--DR---------------------------GFQFLGQLF-
>PF_WP_019960649.1 [Woodsholea maritima]
GGDGQTL--------------------AQ-FQR---TVLL--HLHRLGD---DVRA------GLY---
MPGPHRVVSIPK-RAG---------------------G-----------WRSLSIPCVRDRVLQTAVAQRLQ-
--PILEPE-FEPESYGY---RPGRS-----VAQAIARVATL----R----RQ-------------G-------
FRW---TVDADIERFF-DCVPHGPLLERL--RPFLG------------DPG-LVGLVE--MWL-AGA------
----
------------------------------------G-----------------P----HG------------
----
-----------------------------R-GLPQGSPIS-PLLANLYLD-DVD-------------------
-----EGL-KS--------------------------------------TH---------TR-----------
------------------------------LVRFADDFVI--LT------RNEDE----AL-
QALERARGLLD-KLGLSLNLEKTRIVP----F--EG---------------------------GLDFLGRKF-
>PF_WP_019956891.1 [Loktanella vestfoldensis]
GGDGVTI--------------------DA-FDA---IAEP--RLQALHA---ALAS------GGY---
WPAPARVIEAKK---P--------------------SG---G-------TRTLRIPAIVDRVVQTAAALVLT-
--PILDRE-FEDASFGY---RPGRS-----VGQAVARVAYL----R----NA-------------G-------
YVW---TVDGDIRAFF-DEVPHAPLLDRV--DRVLG-----------CAR-TADLVE--RWLQVYC------
------D--
------------------------------------G-----------------G------------------
----
-----------------------------R-GLPQGMPLS-PVLSNLYLD-SID-------------------
-----EKI-EK--------------------------------------GG---------VR-----------
------------------------------LVRFADDFLL--LC------RSEAV----AE-
GALARMTGILR-EAGLKIHPEKTAIRR----F--ED---------------------------ATRFLGHMF-
>W_[Tistrella_mobilis] 389875622
---GESL--------------------DA-FHI---GVEP--RLARLAA---DVRG------GTY---
RPGPYRLLDVPK--DD---------------------G---G-------TRRLAIPCVADRVLMTSAALVMG-
--PMLDAT-FEPSSHGY---RPGRG-----VRTAIARVESL----R----DQ-------------G-------
FHW---VLDADITRFF-DRVPHDRLLDRL--QQATG-----------DAR-LVDLVG--LWLDGYD---R--
---EGEA--
------------------------------------G-----------------R----GD------------
----
-----------------------------GLGLPQGSPVS-PLLANLYLD-TVD-------------------
-----ERI-AA--------------------------------------AG---------LH-----------
------------------------------LVRFADDFVI--LA------ADEAA----AE-
GARAHVAALLA-DHGLHLHPDKTRVVS----F--DQ---------------------------GFAFLGKLF-
>W__[Desulfarculus_baarsii] 302343124
---RQTL--------------------DD-FAE---SLER--NLEGLHA---ALRS------ASY---
RPGPIRNVSIPK--RD---------------------G---S-------PRRLSIPSVADRVVQTALCQGLT-
--PILEPE-MEDASFAY---RPGRS-----VQMAVERVGRY----F----RQ-------------G-------
YHW---VVDGDIDDYF-DSIPHHGLMAVL--RR--Y-VD---------DQD-VLGLIA--QWLAHAH---A--
------G--
------------------------------------G------------------------------------

----
-----------------------------V-GVSQGSPLS-PLLANIYLD-DMD-------------------
-----ERI-GR--------------------------------------TG---------AR-----------
------------------------------LVRFADDFLL--LC------KSEER----AR-
ESLAAMSALLA-EYGLGLNPDKTRIVN----F--EQ---------------------------GFEFLGRLF-
>PF_KQB14189.1 [Rhodobacter capsulatus]
GGDGETI--------------------AH-FAR---QAEF--RLARLAH---ELQA------DLY---
RPGPLRQISVPKRKGE---------------------G-----------MRVLSIPCVVDRIAQRATAAVLS-
--AALEPQ-FSDASFGY---RPGRS-----VAQAVARVDAL----R----RQ-------------G-------
FTW---VVDADIKAFF-DSVPHAPLAARL--HAAGI------------EPQ-LIELID--LWLDSFS---A--
------E--
------------------------------------G------------------------------------
----
-----------------------------V-GLAQGSPIS-PVLANLHLD-ALD-------------------
-----DSF-GP--------------------------------------RG--------SVR-----------
------------------------------IVRFADDFVL--LT------RCRPG----AE-
AALAKARDQLA-EAGLRLNLAKTRIVP----Y--DQ---------------------------ALRFLGHLF-
>PF_EJW09481.1_(2) [Rhodovulum sp. PH10]
GGDGMTV--------------------AR-FAL---VAES--MIQRLAG---ALRS------GQY---
RPGPARRAFIPK--KD---------------------G---G-------LRPLDIPCVHDRVVQGAATLVLD-
--PVLDKA-FADSSFAY---RRGRS-----VAQAVARIGSL----R----RQ-------------G-------
FTH---VVDGDIRAYF-ERIPHDRLITKL--EQ--H-VD---------DQA-MVDLIW--LWLETYS---L--
----
---TG------------

-----------------------------R-GVPQGAPIS-PLLANLYLD-AVD-------------------
-----DRI-ER--------------------------------------AG---------VR-----------
------------------------------LVRFADDFVL--LA------KTPAS----AE-
KALVEMTRLLA-EEGLEIHPEKTRLVS----F--EE---------------------------GFRFLGHVF-
>PF_EJW09347.1_(2) [Rhodovulum sp. PH10]
GGDGVPL--------------------AR-FLV---NAPA--RIARLSA---GLRD------GSY---
APGPLRRVDIPK--KS---------------------G---G-------TRPLAIPCVVDRIAQTAVMQALA-
--PRLDEE-FAESSFGY---RLGRG-----VRDAVKRVAAL----R----GK-------------G-------
HVY---VVDADIAKFF-ESVPHDKLLERL-AQS----MT---------DGP-LMRLIG--LWIEHGG------
---AR--
------------------------------------G------------------------------------
----
-----------------------------R-GLPQGSPLS-PLLANLYLD-RLD-------------------
-----DAF-AK--------------------------------------RG---------AH-----------
------------------------------IVRFADDFVI--LA------ESRHG----AE-
GALVRAEKLLA-EHGLSLNREKTRVTS----F--DQ---------------------------GFRFLGHLF-
>PF_WP_060836241.1 [Rhodovulum sulfidophilum]
GGDGVTI--------------------DR-FAR---RAPQ--RLTALSG---ALLD------GRY---
RPGDLRRIDLKK-RDG---------------------G-----------TRPLAIPSVIDRVAQTAAALVLT-
--PILDPL-FDEASFGY---RPGRS-----VAMAVRRIDML----R----RR-------------G-------
FCH---VVEADIVRCF-ERIPHEPVLSSLAKTLAGR-VG---------ADR-LVDLVA--LWLEHAA---M--
----
--FLE-T----PG------------

-----------------------------L-GLAQGSPLS-PLLSNLYLD-RLD-------------------
-----DAL-DR--------------------------------------RD---------VA-----------
------------------------------VVRFADDFVL--LC------RSREA----AA-
KALNRAENLLE-AHGLELHGDGTRIVD----F--DR---------------------------GFEFLGHLF-
>W__Azospirillum_lipoferum_1 374998939

---GLTV-------------------GR-FAE---AAPS--RLLALHR---TLRM------GDY---
RPGPLRRLSIPK--PD--------------------G---A-------LRPLAIPPVTDRVAQTAAALVLT-
--PLLDGE-FEDASFGY---RPGRS-----VPQAVARVARW----R----DQ------------G-------
YDW---VVDADIERYF-ERVPHDRLLIRL-ERS----IG--------AGP-LTELIA--VWLESGA------
---EN---
-----------------------------------G------------------------------------
----
----------------------------V-GLPQGSPLS-PLLSNLYLD-DLD-------------------
-----EAL-DG-------------------------------------RG---------LR-----------
-----------------------------LVRFADDFVL--LC------RSRER----AE-
RALDHAAAVLE-EHGLRLNRDKTRIVP----F--DQ--------------------------GFRFLGHLF-
>W_Azospirillum_lipoferum_2 288957883
---GMTV-------------------EE-FSI---DLPT--RLVRLQL---ALAQ------GTY---
RPGRLRRVDVAK-EDG--------------------G-----------TRPLAIPPVVDRVAQTAVAQVLT-
--PLLDPR-MHDGSFAY---RPGRS-----VAMAVARVAEH----R----RQ------------G-------
FGW---VVDGDIERYF-ERVPHERMMACL--AR----VI--------DEPPLLDLIE--LWLESFS------
----
-----------------------------------A----------------------MG------------
----
----------------------------L-GLPQGAPLS-PLLANLYLD-DID-------------------
-----DRI-AA-------------------------------------RG---------VR-----------
-----------------------------LVRFADDFLL--MC------RGEAA----AE-
DARDRMAALLA-EHGLRLHPDKTRIVP----F--EQ--------------------------GFRFLGHLF-
>DS_gi|68548733|ref|ZP_00588202.1 [Pelodictyon phaeoclathratiforme BU-1]
GYDKQSI-------------------TD-YSW---RIEE--HLADLGR---QLLT------NTY---
EPQPLLKLVMLK---P-------------------TG---K-------LRTLLIPTVMERVAQTAAAIVLT-
--PLVESE-LGANTFAY---RPGLS-----RMTAAREIERL----R----NL------------G-------
YNW---VVDADISSFF-DTVDHPLLFQRF--RE--L-CD--------DEE-LLTLIA--RWLTAEI---V--
---DGQN---
---PKV-K----NT------------

----------------------------I-GLPQGCPIS-PMLANLYLD-KFD-------------------
-----ERM-EQ-------------------------------------EG---------FK-----------
-----------------------------LVRFADDFLI--LC------KSKPK----AE-
AALQLSESALA-ELKLQLNNEKTRITT----F--AE--------------------------GFKYLGYLF-
>DS_gi|119357846|ref|YP_912490.1 [Chlorobium phaeobacteroides DSM 266]
GWDNTSI-------------------QD-YSL---RLEE--NLKSLSH---ALLT------GTY---
RQSPLLKLVMLK---P-------------------DG---K-------ERVLLIPGVIDRVAQTAASIVLS-
--PIIEAE-LGNCTFAY---RPGIS-----REGAAREIDRL----H----RE------------G-------
YQW---VLDADIRNFF-DNVRHDLLFQRL--VELVD-----------DKE-MISLLH--RWLTAEI---V--
------D---
-----------------------------------G-------------LNPRT----RN------------
----
----------------------------TMGLPQGCPIS-PALANLYLD-RFD-------------------
-----ETM-EQ-------------------------------------QG---------FK-----------
-----------------------------LVRFADDYLV--LC------KTRPK----AE-
AALKLSESALA-ELKLELHSDKTRITT----F--AE--------------------------GFKYLGYLF-
>PF_WP_027150711.1 [Methylobacter tundripaludum]
GVDYQTL-------------------AA-FAD---RLHK--NLETLRD---EVNY------ETY---
QPQPLLRIELEK---P-------------------GG---G-------TRPLSIPTVRDRILQTAVTRVIE-
--PLFEAE-FEDCSFAY---RKGRS-----VDQALDRIQLL----Q----RQ------------G-------
YHW---VVDADIQCFF-DSIDHTLLMTMV--GK--L-VT--------DVG-LLRLIE--QWLCATV---V--
---DG-D---
---RRF-V----LS------------

```
-------------------------------------------------------------------------
-----------------------------K-GVAQGSPIG-PLLSNLYLH-HLD-------------------
-----EAL-LD--------------------------------------NN---------LC-----------
------------------------------LIRFADDFLI--LC------KSQDH----AE-
QALELTDSLLG-ELRLTLNTRKTQIVH----F--NQ---------------------------GFRFLGVQF-
>PF_ETX03376.1 [Candidatus Entotheonella sp. TSY2]
GIDGETI--------------------QE-FSA---DLEQ--QLLNLQR---EVRE------QRY---
HPKALLRIYVEK--DD---------------------G---S-------PRPLSIPAVRDRVLQTAAALVLT-
--PILEPA-FEDVSYAY---RPGRS-----VNQAVQHIMAL----R----EK-------------G-------
YQW---VVDADIRRYF-DEIPHGKLVACL--RE--H-VA---------DAG-VLALVQ--QWLTMEV---V--
-------------------------------------------------------------------------
------------------------------------G-------------NHERF----RL------------
-------------------------------------------------------------------------
-----------------------------QKGVPQGSPLS-PLLANLYLD-RFD-------------------
-----ETL-TD--------------------------------------RG---------YK-----------
------------------------------LIRFADDFVI--LC------KSRPK----AE-
AALELTEAVLD-DLQLALHPGKTRITH----F--DH---------------------------GFRYLGVQF-
```

Supplementary Data 1. Alignment of 537 sequences used to infer the phylogenetic tree shown in Fig. 1. The annotations DS, PF and W were used for the initial identification of sequences: our published data set7 (DS), RT-Cas1 sequences obtained from NCBI (PF), and RTs associated with Cas1 within CRISPR-Cas modules described by Makarowa et al.21 (W). The specific accession identification indicated by “fid” corresponds to sequences from the PATRIC database.

Supplementary Dataset 2. MSA of Cas1 sequences used in Figure 3.

>PF_WP_024721321.1 subtype I-B CRISPR-associated endonuclease Cas1 [Clostridiales bacterium VE202-01]
YHIINDG-ILTKKDYSILFE-----------------------------------NENK----------
KIEIPAEVTEHINIYSQ-TIFSSNFFKTLNQHNIRVSLFD-QYDHYIGSFIPINY--
HKSASILIKQVENYINKAKRLEIAKKIIDSGTTNIISNLKYYNK--HNDDI-------QLDKIIDDLKICK--
--QEMK---AAKTLEILLL----QEARMRELYYSSYNIIL--SNSEFKF------TKRTRRPP-
KDALNAMISFGNTIIYQKIANEIYKTKLDIRISYLHSAMRR-
YENLNLDISEIIKPILVDKVIFSLINKRIIDAKVHFEKR-------NNG-----------
VFLNKEGKYLFIDQLNKKLS------STITINNKKVS-YQEILQQEVLKLLRYFKYDE--EYEPFKYR
>PF_WP_051592781.1 subtype I-B CRISPR-associated endonuclease Cas1 [[Clostridium] saccharogumia]
YHIINDG-ILTKKDYSILFE-----------------------------------NENK----------
KIEIPAEVTEHINIYSQ-TIFSSNFFKTLNQHNIRVSLFD-QYDHYIGSFMPINY--
HKSASILIKQVENYINKAKRLEIAKKIIDSGTTNIISNLKYYNK--HNDDI-------QLDKIIDDLKICK--
--QEMK---AAKTLEILLL----QEARMRELYYSSYNIIL--SNPEFKF------TKRTRRPP-
KDALNAMISFGNTIIYQKIANEIYKTKLDIRISYLHSAMRR-
YENLNLDISEIIKPILVDKVIFSLINKRIIDAKVHFEKR-------NNG-----------
VFLNKEGKYLFIDQLNKKLS------STITINNKKVS-YQEILQQEVLKLLRYFKYDE--EYEPFKYR
>PF_CDC93231.1 cRISPR-associated endonuclease Cas1 2 [Firmicutes bacterium CAG:227]
FHILSDG-IINRQDYSLLFE-----------------------------------NEQK----------
RHYIPVEVVDQINIYGN-VTLANNILETFNNKGIQTSFYD-KYGYLIGTFIPEKN--
KKNAKIILEQSKNYMDTAVRIDMARKMEIAGLHNMRANLRYYEK--RHKGR--------FTEVIESIGEFA--
--KQMK---SITDINAMML----IEAKARQKYYSVFDQIL--ENEEFKF------VQRTKRPP-
KNEINACLSFGNTLLYNQFSSLIWKKGLDIRFGIVHATNRR-
TRSLNLDFADIFKPIIIDRVIFTMINKKMLTVSMDF------EKTSMGG-----------
VYLSKEGKKVFLRMYEEKLN------SKIIVKGERMT-YRRLLENEVQSYKNYLLNGE--KYNPYKY-
>PF_CRL43259.1 hypothetical protein RIL183_34091 [Roseburia inulinivorans]
YHLINEGILTKQDFNILFES-----------------------------------ENGK-----------
KYIPVETTDSLYIYSN-VIMSGNFFDFMNQVGLNVSFIN-KYGEKIGSFVPNNS--
RRNIKTELKQLRMYDSEKERLDMARRLEIASVSNIRANLRYYQR--RKNAT-------ELEAAVKDMTDII--
--TKLN---EARDINHMMM----LEAQARQKYYGCFNSIL--EGKQFYF------DKRTRRPP-
QDPLNAMISFGNTLLYQRIANEINRTSLDIRIGIVHAAGNR-
PESLNLDLADLFKPILVDRTIFTLVNRKMINVN-DF------VEVENNG-----------
IYLNNRAKKIFISEYENKLY------QKVTFDGAERT-YDYLIKNEIQKLKKYIENGE--KYKPYKYV
>PF_CVI70780.1 CRISPR-associated endonuclease Cas1 [Eubacteriaceae bacterium CHKCI004]
YHIINDG-ILNKKDYTLLFE------------------------------------NEK---------
EKKYLPVETVDKLNIYSN-VIFDTGFFEMVSRYNIDVSIFD-KYGKHCGTFCGSKH--
ARTSDMVIKQVSLYNNDAKRLSVAKSMLIAAAHNMRANVRYYVK--KEKLK---------KENVDKLSAFI--
--KKLN---DASSISNLMM----IEAQCRQSYYTYMGKII--GGGPFHF------AQRTKRPP-
RDAVNAMISFGNVFLYEKIATEIYKTSLDIKVGFLHSTNRR-
KATLNLDIAEIFKPVIVDRVIFTVIHKRILDVS-RHFE----SQE-NNG-----------
VYLNREGKRPFINELMRKMY------TKITVDRKLMT-YEALIRNEIWKIYRMIERGE--SYKPYKYT
>PF_ETO97675.1 CRISPR-associated endonuclease Cas1 [Lachnoanaerobaculum sp. MSX33]
YHIINNGILNRKDFTLLFEN-----------------------------------DHGK-----------
KYIPVEATESLNVYSS-IIFSSDFFKYISSKKICVNIFD-KYGELTGTFALPES--
LHGGLTILKQAAIYLYNDKRKIIAKKIEIASLHNMRSNLKYYER--HHSNK-------NLKNGISSFSEYI--
--TAMN---EVTNVTTLLT----IEARASQLYYSLFHEII--CDPAFEF------TKRTRRPP-
KAPLNALISFENVFLYNGVATEIQKTSLDIRIGFVHATNRR-

SQSLNLDIADLFKPLIVDRAIFTIINRHMIHASEHF------EKTEDGG-----------
IYLNKEGKQIFINELENKVY------QKQTEENQPRT-YDTRIREEIHKIFRFVCYDE--KYKPFKYN
>PF_WP_008751399.1 subtype I-B CRISPR-associated endonuclease Cas1
[Lachnoanaerobaculum saburreum]
YHIINNGILNRKDFTLLFEN----------------------------------DRGK----------
KYLPVEATESLNVYTS-VIFSSDFFKYVGNKKICVNIFD-KYGELAGTFSPPES--
LHGGLTMLKQAAIYLDDEKRKLIARKLEIASLHNMRSNLKYYER--HHSSE-------NLKDGITSFSEYI--
--TAMN---EATNIVMLLT----IEARARQLYYSLFHEII--CDPAFEF------TKRTRRPP-
KDPLNALISFGNVFLYNRIATEIQKTSLDIRIGFVHATNRR-
NQSLNLDIADLFKPLIVDRAIFTIINRHMIHASEHF------EKTEDGG-----------
IYLNKEGKQIFINELENKVY------QKQTEENKPRT-YDTRIREEIHKIFRLVCYDE--KYKPFKYN
>PF_WP_009447486.1 subtype I-B CRISPR-associated endonuclease Cas1
[Lachnospiraceae bacterium oral taxon 082]
YHIINNGILNRKDFTLLFEN----------------------------------DRGK----------
KYLPVEATESLNVYSS-VIFSSDFFKYISSRKICVNIFD-KYGELAGIFSPPES--
LHGGLTMLKQAAIYLDDEKRKLIAKKFEIASLHNMRSNLKYYER--HHSNE-------NLKNGISSFSEYI--
--TALN---EATTVTTLLT----IEARARQLYYSLFHEII--CDPAFEF------IKRTRRPP-
KDPLNALISFGNVFLYNRVATEIQKTSLDIRIGFVHATNRR-
NQSLNLDIADLFKPLIVDRAIFTIINRHMIHTSEHF------EKTEDGG-----------
IYLNKEGKQIFINELENKVY------QKQTEENQPRT-YDTRIREEIHKIFRFVCYDE--KYKPFKYN
>PF_WP_060932241.1 type I-B CRISPR-associated endonuclease Cas1
[Lachnoanaerobaculum saburreum]
YHIINNGILNRKDFTLLFEN----------------------------------DRGK----------
KYLPVDATESLNVYSS-VIFSSDFFKYISSKKICVNIFD-KYGELAGIFAPPES--
LNGGLTMLKQAAIYLDDEKRKLIAKKFEIASLHNMRSNLKYYER--HHSSE-------NLKNGIYSFSEYI--
--TALN---EATNVTTLLT----IEARARQLYYSLFHEII--CDPAFEF------TKRTRRPP-
KDPLNALISFGNVFLYNRVATEIQKTSLDIRIGFVHATNRR-
NQSLNLDIADLFKPLIVDRAIFTIINRHMIHTSEHF------EKTEDGG-----------
IYLNKEGKQIFINELENKVY------QKQTEENQPRT-YDTRIREEIHKIFRFVCYDE--KYKPFKYN
>W_Arthrospiraplatensis_(479129287) A new protein sequence entered
manually
IYLTEPQSYLEADLRDFAIV----------------------------------YNDR---------
IQSRHSIDTTQQIIAFEG-CTLGRSAIAAIYRYRIRLSFIG-DNGHITAQITP-----
QETPFYTVLQQERNSDRNFRIAFIHSLLPGRWYNATKVLQSLGY----------------SGIGGCLEQQL--
--EVLP---GLNSWLALQT----WQVKVFQRYQKALRSVL--------V------SVKDFEPT--------
LELAYALLSGELYVLLRSSGCCAEVGTLHLHCQN-HLPLPCDLMEPFR-PVVEAWVFKKAP--------
KFSD--------RGG-------------LSQSYRATFIQNWESLMA----TQIFHAYAGQVS--
LRSALAWQVEEYVRAITETL--EYRPFLLK
>W_CRISPR-associated_protein_Cas1_[Pirellula_staleyi] 283778924
LIAGPGIEEILVHGDVLQFK----------------------------------HQDQ--------
RGIESIPLHTLREVVVLGA-VSLSHRVLSAIQQNAISLLLLD-ESANRTAYIDCQNAEPDSAGIE--
AQVDLIRNPESSLAIARQLISAKLHNYATLADAYPP--KSHSG----------NAHRSLMRLA----KDAQ--
-QASSLPELLG----IEGSGAALWYGEIGMRL---SPGFHW------ERRVAPNA-
HDPVNILLNLAQTVLYRMTQHAIAQAKLVDTLGFLHQPRAG-HAALASDMQEPFR-HLMDRAVLETVRR--
IRPE-EF------EPD-ERG---------PFPVRMKPRALRESLAWIHRILA----LPCNGAGQSSPTA-
YRLQIFKQVRSFRRFLMDPGG-RFEPFRHP
>PF_WP_027180402.1 CRISPR-associated endonuclease Cas1 [Desulfovibrio
bastinii]
VYVCNHFASLGVDVDCLAIR----------------------------------KSGS---------
VIARVPFERTNEIVVYGN-SAVSTKLIQKCSYEKIPITFCT-PSGHYTNTLYPESKNFHVISGQ-
HLARHNSMVQVERELICKRLISAKINNYIKWLSSREL---------------HEDVISNLEYYL----KRVS-
--QATAKEHLFG----IEGNAAKVCFAALNDLI--LDDSFRS------ENRIKLSK-
PDRLNTLLDCCYSLLFNRINSMLRVRGMNPYLGILHSDENN-YESLVADLQEPFRAR-

MDRFAVRLLNKNIITEK-DF-E----NVTPIKF------------KLSGRSMGKVLEHFEREMC--------
IRIATESGT-FAQLVDAQVQNLRNWVYERD--DLKFFRSG
>W_CRISPR_associated_endonuclease_Cas1_[Desulfovibrio_hydrothermalis]_(2)
436839745
VYVCNHFASLGVDIDCLAVK----------------------------------KNGT---------
AIARIPFARTNEIVVYGN-SSVSTKLIQKCSYEKIPVTFCT-PSGYYINTLYPDSRQYHITSGK-
HMARYESMSDTNKAKAARKLVSAKINNYRQWLSSRGA--------------ACDISDKLKSIE----KSVQ-
--DASGNSTLMG----IEGNAARLCFSVFNSFI--MDSTMRS------HVRLPRSK-
PDQLNALLDFCYTLLFNRINSLLRVRGLNPYLGILHSSKNN-YESLVADLQEPFRAR-
MDRFALRLLNKNIITAK-DF------EPVTAKK-----------FKLNGRSMGKILESFEKEMC--------
IRIATEAGT-FAQLLDAQVQNLRNWVMDRDELKFFRSGER
>PF_WP_015334627.1 CRISPR-associated endonuclease Cas1 [Desulfovibrio
hydrothermalis]
VYVCNHFASLGVDIDCLAVK----------------------------------KNGT---------
AIARIPFARTNEIVVYGN-SSVSTKLIQKCSYEKIPVTFCT-PSGYYINTLYPDSRQYHITSGK-
HMARYESMSDTNKAKAARKLVSAKINNYRQWLSSRGA--------------ACDISDKLKSIE----KSVQ-
--DASGNSTLMG----IEGNAARLCFSVFNSFI--MDSTMRS------HVRLPRSK-
PDQLNALLDFCYTLLFNRINSLLRVRGLNPYLGILHSSKNN-YESLVADLQEPFRAR-
MDRFALRLLNKNIITAK-DF------EPVTAKK-----------FKLNGRSMGKILESFEKEMC--------
IRIATEAGT-FAQLLDAQVQNLRNWVMDRD--ELKFFRSG
>PF_WP_019672870.1 CRISPR-associated endonuclease Cas1 [Psychrobacter
lutiphocae]
IMITGSRKYLGINGGALEVR------------------QKQARKQPGRSLSTSPLSNQ---------
LVQVIPLRRISQLVVMGN-HSLSSPLLTACAKHHISVHLVN-QWGFQVGTFKPSHAEYYAVSAE-
QYQRHQQLKPSERMAIAADLVLAKINNYQTWIINSYR------------KGDAQVNKQLDQIA----NQAS-
--SATTIEALMG----YEGQAAKICFQRLQSVM-IGDQQAAF-----SSKRRSRGG-
PDRLNSMLNFGYYWLFTRISGLLHSHGLNPYLSFLHEPEQN-YETLVYDIMELFRVQ-
VDKTVLRLINRKQIQAD-SF------HLDAKKG-----------WKLNNDALHLLSNQLQSTFA----
SKINQTF-------LEDIILIQVRTLLHWATQQQ--SLVWFYWY
>PF_KFB71594.1 CRISPR-associated endonuclease Cas1 [Candidatus
Accumulibacter sp. BA-91]
LYITEPYAFVGSNHGTIEVH----------------------------------AGSK---------
SLGSFPLARTAGVVTLVP-CTLSSALIARLADQCIPLAIAG-TQGRQIATVAGDTARRFATAAT-
QANRHASLGEAGRCRAAGAFATAKLANYIALIRQRGP------------AGTAALVARLENGI----AAIA-
--SATDIDAIRG----VEGDCARECFPFIAGWI--NSPDFPW------
QGRRRHGEFPDRLNSLLNFGYHLLFTRINALLRVSGLNPYLGFLHAANGR-YEALACDVQEAFRPH-
IDRLVVRLLNLKVIEAA-DFEE----S---EEG-----------WWLIRPARTRFLQQFAREIE--------
RRPMRRRYS-LGEAIEGQVRALHAWLIEDR--ELVLYRWS
>PF_KIE18281.1_(2) hypothetical protein DS62_12155, partial [Smithella
sp. SC_K08D17]
LYITEPYIFLSLNADAIDIC----------------------------------RSKK---------
IIESIPLRRISEIMVMEK-SVVSTALLKKCTDAGIPVTLTL-NSGYYVTTIKPDSKQYFDTAYV-
HARAYYALTETENLCMAKEFAAGKLQNYAAFFKQRYA------------KDQNLFIQQIERTA----ANIH-
--AASDINQVRG----YEGSMAKKIYEQYNSII--ENPAFHI------KKRDRKTP--
DPINALLNFGYYLLFTRVNSTVRAVGLNPYLGFLHSPEDN-YESFVCDVEELFRSR-
IDRFIISLINLKAITEA-DF-T-----PM-ENQ-----------CRISRDAIRKFISQLEKEME--------
RKNTRNTLS-LKESIYVQVLVLKKYFTGNG--NLSFYRWI
>PF_WP_052565451.1 hypothetical protein [Candidatus Brocadia sinica]
LYIVESYLFLSLNGDAIDIC----------------------------------KNRK---------
IVETIPLRRISEIMVMEK-TVLSTALLRKCTEGNIPLTIAL-GTGYYITTVKPDSK-----------------
----
----------------------KIL---

-
>PF_KKO19838.1 hypothetical protein BROFUL_01440 [Candidatus Brocadia fulgida]
LYIVEPYLFLSLNGDAVDLC-----------------------------------KNRK---------
VVETIPLRRISEIMVMEK-TVFSTALLRKCTEGNIPLTIAL-GSGYYITTVKPDSKKYYDVSYE-
HGRKYYSLSETELLCIAKEFAAGKLQNYLALFQQRFR-------------KEQYRFIGELEGVI----CQMH-
--QAGDVAQVRG----LEGAAAKKIYQRLNNFI--DDDAFAL------QKRDRRSP--
DRMNSLLNLGYYLLFSRINATVRAVGLNPYLGFLHSPQDN-YESLVCDIEELFRAR-
IDRFIIRLINLKVVGRE-DFVE-----T--ERG------------LHLEKDAVRKFIDQFESEME--------
KKGAQGTLS-MKEDLYVQTIVLKKWALENG--SLSFYRWK
>PF_WP_007481073.1 CRISPR-associated endonuclease Cas1 [Bacteroides salyersiae]
LVVSTPGSYIGASYKGVTVK-----------------------------------LQGK----------
IINKPSPTLRHITVIGKGVSFSSNAMMFCMNHKIPIDFFD-SKGKQYATVLNPVF--LDETLW---
SKQVSLPLERKVKLASQIIVGKLKNQLNLIKYYHK--YHKDILGGELLEKYIEAVLKLEQQI-EKAKSYPIT-
DGKYASGLMG----IESQGAIAYWAYIRVLI--ADDGINF------ISREHQGA-
TDLMNSLLNYGYAILYARVWKNILAAKLNPSIGILHAHQDG-
KPTLVFDIVELFRSQVVDRIVISMIQKKISLKM-------------HDG-------------
LLNESTKRTLIRHILERLN------RYEKFRGEEIT-FSQIILKQSQDIAHYIAGENN-EFKPYVAK
>PF_WP_044299432.1 CRISPR-associated endonuclease Cas1 [Bacteroides fragilis]
LVISIPGSYIGATYKGITVK-----------------------------------LQGK----------
IINKPSPALKHITVVGRGISLSSNAITYCMNHKIPIDFFD-SRGKQYGTVLNPVF--LDVTLW-
NKQVALPLEQ--KIKLATQIIIGKLKNQLNLIKYYHK--YHKDILGGKLSEKYVEVVLKIDKLI-
EKAKNYSQR-NEKYTAELMA----IESQAAIAYWSYIRVLT--ADDGIDF------IRREHQGA-
TDLLNSLLNYGYAILYARVWKNILAAKLNPSIGVLHAKQDG-
KPTLVFDVVELFRAQMVDRVVVSLIQKKVSLKM-------------HDG-------------
LLNESSKRALIRYILERLN------RYEKYRGEEIT-FSQIILRQAQEIALFISGDNL-IFKPYVAK
>PF_WP_008660287.1 MULTISPECIES: CRISPR-associated endonuclease Cas1 [Bacteroides]
LVISIPGSYIGATYKGITVK-----------------------------------LQGK----------
IINKPSPALKHITVVGKGISLSSNAITYCMNHKIPIDFFD-GRGKQYSTVLNPVF--LDGTLW-
NKQVELPLEQ--KIKLATQIIIGKLKNQLNLIKYYHK--YHKDILGGKLSEKYVEVVLKIDKLI-
EKAKNYSQR-NEKYTAELMA----IESQAAIAYWSYIRVLT--ADDGIDF------IRREHQGA-
TDLLNSLLNYGYAILYARVWKNILAAKLNPSIGVLHAKQDG-
KPTLVFDVVELFRAQMVDRVVISLIQKKVSLKM-------------HDG-------------
LLNESSKRVLIRYILERLN------RYEKYRGEEIT-FSQIILRQAQEIALFISGDNL-IFKPYVAK
>PF_WP_032562078.1 CRISPR-associated endonuclease Cas1 [Bacteroides fragilis]
LVISIPGSYIGATYKGITVK-----------------------------------LQGK----------
IINKPSPALKHITVVGKGISLSSNAITYCMNHKIPIDFFD-GRGKQYGTVLNPVF--LDVTLW-
NKQVELPLEQ--KIKLATQIIIGKLKNQLNLIKYYHK--YHKDILGGKLSEKYVEVVLKIDKLI-
EKAKNYSQR-NEKYTAELMA----IESQAAIAYWSYIRVLT--ADDGIDF------IRREHQGA-
TDLLNSLLNYGYAILYARVWKNILAAKLNPSIGVLHAKQDG-
KPTLVFDVVELFRAQMVDRVVISLIQKKVSLKM-------------HDG-------------
LLNESSKRVLIRYILERLN------RYEKYRGEEIT-FSQIILRQAQEIALFISGDNL-IFKPYVAK
>PF_EXY33263.1 CRISPR-associated endonuclease Cas1, partial [Bacteroides fragilis str. 3397 T10]
LVISIPGSYIGATYKGITVK-----------------------------------LQGK----------
IINKPSPALKHITVVGKGISLSSNAITYCMNHKIPIDFFD-GRGKQYGTVLNPVF--LDGTLW-
NKQVELPLEQ--KIKLATQIIIGKLKNQLNLIKYYHK--YHKDILGGKLSEKYVEVVLKIDKLI-
EKAKNYSQR-NEKYTAELMA----IESQAAIAYWSYIRVLT--ADDGIDF------IRREHQGA-
TDLLNSLLNYGYAILYARVWKNILAAKLNPSIGVLHAKQDG-

KPTLVFDVVELFRAQMVDRVVISLIQKKVSLKM------------HDG------------
LLNESSKRVLIRYILERLN------RYEKYRGEEIT-FSQIILRQAQEIALFISGDNL-IF------
>PF_WP_032563007.1 CRISPR-associated endonuclease Cas1 [Bacteroides fragilis]
LVISIPGSYIGATYKGITVK----------------------------------LQGK----------
IINKPSPALKHITVVGKGISLSSNAITYCMNHKIPIDFFD-GRGKQYGTVLNPVF--LDGTLW-
NKQVELPLEQ--KIKLATQIIIGKLKNQLNLIKYYHK--YHKDILGGKLSEKYVEVVLKIDKLI-
EKAKNYSQR-NEKYTAELMA----IESQAAIAYWSYIRVLT--ADDGIDF------IRREHQGA-
TDLLNSLLNYGYAILYARVWKNILAGKLNPSIGVLHAKQDG-
KPTLVFDVVELFRAQMVDRVVISLIQKKVSLKM------------HDG------------
LLNESSKRVLIRYILERLN------RYEKYRGEEIT-FSQIILRQAQEIALFISGDNL-IFKPYVAK
>PF_WP_032585076.1 CRISPR-associated protein Cas1, partial [Bacteroides fragilis]
LVISIPGSYIGATYKGITVK----------------------------------LQGK----------
IINKPSPALKHITVVGKGISLSSNAITYCMNHKIPIDFFD-GRGKQYGTVLNPVF--LDGTLW----------
-------------------------NK--

>PF_WP_036885018.1 CRISPR-associated endonuclease Cas1 [Porphyromonas gingivicanis]
FVVSEAGAFIGLNRNYLVVK----------------------------------KFGN---------
TICKHPAHQVEQISIIAPGVSFSSHVTNYCHKKNIRIIYYK-ATGEAYASVGSMNS--ILPSHM---
KAQMSLPENRIREFSSALVHNKIKNQTKLLKYYQK--YFRKE--EELCQYLQQSIEQLNAI-----EKLSI--
SGNTVEQFRQNAMLIEARGAKIYWNAFALLI--QRTGHIF------DGRVQQGA-
SDIVNQMLNYGYAILQSYVMRTIDLWQLNPNIGILHSTQDN-
KPALCFDLMEQYRSFVVDRSILALLSKKEEVRQ----E--------KSG------------
LLDIQTRTRIISKIKERWC-----SPEYFRTGQKTL--SEIMTLQTKNVRDFCLKKEN-KIKFYTPK
>W_CRISPR-associated_protein_Cas1_[Porphyromonas_gingivalis]_(2) 188995791
LVVTEPGAFIGISRNHVLVR----------------------------------KYGK---------
TICKQPAAQIEQISIISDGVSLSSNVTKYCRKKNIRVIF--------------------------------
----

-
>PF_WP_039421078.1_(2) CRISPR-associated endonuclease Cas1 [Porphyromonas gulae]
LVVTEPGAFIGISRNHVLVR----------------------------------KYGK---------
TICKRPAARIEQISIISDGVSLSSNVTKYCRKKNIRVIFYN-ATGQAYASLDGMNT--ILPSVM---
EAQMRLSEEKKQEFILALIRNKVRNQGKLLRYYHK--YYRHD--KELKEPLSNAIAELKQL-----EDIPIA-
EGSSLADFRQHAMLHEARCAQVYWRAFALLV--HRSGHEF------EGREHKGA-
EGLVNQMLNYGYAILRSYVMKTIDLWQLNPNIGILHSTQDN-
RPALCFDLMEQYRAFVVDRSILALLAKGEDVGQ----D--------NKG------------
LLDMSTRSRIITKINERWF-----ATEYYRSGEKLF--SDIMKLQTKDVRAFCCGKVK-RIKFYTPK
>PF_WP_039438358.1_(2) CRISPR-associated endonuclease Cas1 [Porphyromonas gulae]
LVVTEPGAFIGISRNHVLVR----------------------------------KYGK---------
TICKRPAARIEQISIISDGVSLSSNVTKYCRKKNIRVIFYN-ATGQAYASLDGMNT--ILPSVM---
EAQMRLSEEKKQEFILALIRNKVRNQGKLLRYYHK--YYRHD-K-ELKEPLSNAIAELKQL-----
EDIPIAAEGSSLADFRQHAMLHEARCAQVYWRAFALLV--HRSGHEF------EGREHKGA-
EGLVNQMLNYGYAILRSYVMKTIDLWQLNPNIGILHSTQDN-

RPALCFDLMEQYRAFVVDRSILALLAKGEDVGQ----D--------NKG-------------
LLDMSTRSRIITKINERWF-----ATEYYRSGEKLF--SDIMKLQTKDVRAFCCGKVK-RIKFYTPK
>PF_WP_039428138.1 CRISPR-associated endonuclease Cas1 [Porphyromonas sp. COT-052 OH4946]
LVVTESGAFIGISRNHVLVR-----------------------------------KYGK---------
TICKQPAARIEQISIISDGVSLSSNVTKYCRKKNIRVIFYN-ATGQAYASLDGMNT--ILPSVM---
EAQMRLSEEKKQEFILALIRNKVRNQGKLLRYYHK--YYRHD--KELKEPLSNAIAELKQL-----
EDIPIAAEGSSLADFRQHAMLHEARCAQVYWRAFALLV--HRSGHEF------EGREHKGA-
EGLVNQMLNYGYAILRSYVMKTIDLWQLNPNIGILHSTQDN-
RPALCFDLMEQYRAFVVDRSILALLAKGEDVGQ----D--------NKG-------------
LLDMSTRSRIITKINERWF-----ATEYYRSGEKLF--SDIMKLQTKDVRAFCCGKVK-RIKFYTPK
>PF_WP_039424514.1 CRISPR-associated endonuclease Cas1 [Porphyromonas gulae]
LVVTEPGAFIGISRNHVLVR-----------------------------------KYGK---------
TICKQPAARIEQISIISDGVSLSSNVTKYCRKKNIRVIFYN-ATGQAYASLDGMNT--ILPSVM---
EAQMRLSEEKKQEFILALIRNKVRNQGKLLRYYHK--YYRHD--KELKEPLSNAIAELKQL-----
EDIPIAAEGSSLADFRQHAMLHEARCAQVYWRAFALLV--HRSGHEF------EGREHKGA-
EGLVNQMLNYGYAILQSYVMKTIDLWQLNPNIGILHSTQDN-
RPALCFDLMEQYRAFVVDRSILALLAKGEDVGQ----D--------NKG-------------
LLDMSTRSRIITKINERWF-----ATEYYRSGEKLF--SDIMKLQTKDVRAFCCGKVK-RIKFYTPK
>PF_WP_046200570.1_(4) CRISPR-associated endonuclease Cas1 [Porphyromonas gulae]
LVVTEPGAFIGISRNHVLVR-----------------------------------KYGK---------
TICKQPAARIEQISIISDGVSLSSNVTKYCRKKNIRVIFYN-ATGQAYASLDGMNT--ILPSVM---
EAQMRLSEEKKQEFILALIRNKVRNQGKLLRYYHK--YYRHDK--ELKEPLSNAIAELKQL-----
EDIPIAAEGSSLADFRQHAMLHEARCAQVYWRAFALLV--HRSGHEF------EGREHKGA-
EGLVNQMLNYGYAILRSYVMKTIDLWQLNPNIGILHSTQDN-
RPALCFDLMEQYRAFVVDRSILALLAKGEDVGQ----D--------NKG-------------
LLDMSTRSRIITKINERWF-----ATEYYRSGEKLF--SDIMKLQTKDVRAFCCGKVK-RIKFYTPK
>PF_WP_039434532.1 CRISPR-associated endonuclease Cas1 [Porphyromonas gulae]
LVVTEPGAFIGISRNHVLVR-----------------------------------KYGK---------
TICKRPAARIEQISIISDGVSLSSNVTKYCRKKNIRVIFYN-ATGQAYASLDGMNT--ILPSVM---
EAQMRLSEEKKQEFILALIRNKVRNQGKLLRYYHK--YYRHD--KELKEPLSNAIAELKQL-----
EDIPIAAEGSSLADFRQHAMLHEARCAQVYWRAFALLV--HRSGHEF------EGREHKGA-
EGLVNQMLNYGYAILRSYVMKTIDLWQLNPNIGILHSTQDN-
RPALCFDLMEQYRAFVVDRSILALLAKGEDVGQ----N--------HKG-------------
LLDMPTRSRIISKINERWF-----ATEYYRSGEKLF--SDIMKLQTKDVRAFCCGKVK-RIKFYTPK
>PF_WP_039440907.1 CRISPR-associated endonuclease Cas1 [Porphyromonas gulae]
LVVTEPGAFIGISRNHVLVR-----------------------------------KYGK---------
TICKRPAARIEQISIISDGVSLSSNVTKYCRKKNIRVIFYN-ATGQAYASLDGMNT--ILPSVM---
EAQMRLSEEKKQEFILALIRNKVRNQGKLLRYYHK--YYRHDK--ELKEPLSNAIAELKQL-----
EDIPIAAEGSSLADFRQHAMLHEARCAQVYWRAFALLV--HRSGHEF------EGREHKGA-
EGLVNQMLNYGYAILRSYVMKTIDLWQLNPNIGILHSTQDN-
RPALCFDLMEQYRAFVVDRSILALLAKGEDVGQ----D--------NKG-------------
LLDMPTRSRIISKINERWF-----ATEYYRSGEKLF--SDIMKLQTKDVRAFCCGKVK-RIKFYTPK
>PF_KKO17867.1 CRISPR-associated protein [Candidatus Brocadia fulgida]
LIISSPGVFLGKTGERIVLR-----------------------------------EKRK---------
NIAEYLFSRIKNITVNTAGVSLSSDIIFRCSRSKIPITFYT-LRGMPYATLQSPLHSMGSVSVL----
QIRTYETEKSLVFIKKVLTGKAKNQINLMKFYLR---SRKKTQPEFTRITTENILGMKNLL----
KQMLQLEHGEVFSVVRDRIFSFEGRISALYWECMRLLV---APELGF------EKRNRFQA-
PDLVNNMLNYGYGILYQRV---

>PF_KPA10619.1 cRISPR-associated endonuclease Cas1 [Candidatus Magnetomorum sp. HK-1]
LIITKFGHPIGYTKHKFTVR-----------------------------------HKGQ---------
IVASIPKNRLKRIAIKSTGVSLSSNLIYQCCTRNIAIEFFS-NNGETYAMIYTPQRSISQSSEV----
QLKARNDKKGSKLAYYFIKGKAKNQINMIKYFNKYLKKTNPENSKIIEHNIQQMDKLYESF-QLPENMD---
RKDVRDRLMG----TEGIISQHYWEAVKLII---PVSVGF------ERRVTRGA-
KDLFNSCLNYGYGILYNRVQKALADAGAALHISFLHEPNNK-
KPTLVYDLIEEFRQFVIDRTIVVLFNRNEPLST-D-----------KKA-------------
LLTMKSRQLIALNVQERLS----SYTTWRGRRWKC---EDIIYHQARLIMHYLNDEK--KYRPFLGR
>PF_CZE46369.1_(3) CRISPR-associated protein Cas1 [Campylobacter fetus subsp. fetus]
LHISTPHCFLGVSNVNFVIR-----------------------------------YKGK---------
VILKVRIDQIHQIIIACD-ISLSTNAIKAATKRNISIDFLG-FNNQIYASLFSHTS--TITPAY-
KAQIDFLNSPN-SLNLAKEFIKAKATNQINYLKYLDK--HYK---------ILASNIDKMHKNL----
KKALI--SATTTSELMG----YEGAISSLYFDAIASTL----EDKEF-------KRVGKGA-
TDLVNSLLNYGYAILYSTVQSALIKAGLYLNISYFHVSA---
KFSLSFDFIEEFRVVAVDRVVFTLLHQKTKLSL-------------KDG-------------
LLDVPTKKKLTQKVALALL------STHKYKNEELN-LEQIIQAQAYLLRKQIFNEA--KYKGFLVR
>PF_WP_005873073.1 CRISPR-associated endonuclease Cas1 [Campylobacter gracilis]
IHITTPFYFLALSQGKLVLK-----------------------------------SKGA---------
IKHKFPINQISQIIINAQ-ISLSSAVIKECAKKKISINFIDEKTNLSYATLISANS--AIPKTA--
ASQISLLTTKKSLRIAQQFIIGKLKNQINYLKYLGK--YHK---------NLGAEIKAMQEILK---LRVP--
-GAASVSELMG----FEGSAANSYWQAIAKAV---DYEFGF------SARVTQGA-
TDIVNSALNYGYAILYSKILKSIAAAGLSPHVSYLHALDEQ-
KPTLAFDLIEEFRAFIVDRAVISMVNKNEPFEI-------------KDG-------------
LLSVATRQNIAKNVNEKLF------ACTQYRGEQLK-AQDIIDRQAYALKRAVTQNE--KYKPFIGR
>PF_WP_021087740.1 CRISPR-associated endonuclease Cas1 [Campylobacter concisus]
IHITTPFYFLALSQGKFVLK-----------------------------------DKGT---------
IKHKFPIAQITQIIINAQ-ISLSSAVIKECAKRKISINFIDEKTNLSYATLFTANS--AISKT--
AASQITLLKTKKSMRIAQQFIIGKLKNQINYLKYLDK--YHK---------SLSSHISSMQEILT---SHVP-
--NAQSVSELLG----FEGSSANAYWQAIAKAI---DYKFSF------TARVTQGA-
TDIVNSALNYGYAILYSKILKSIAAVGLSPHVSYLHALDEQ-
KPTLAFDLIEEFRAFIVDRAIISMVNKNEPFEI-------------KDG-------------
LLSAKTRQNIAKNVNEKLF------AYTQYRGEQLK-AQDIIDKQAYALKRAVTQNE--KYKPFIGR
>PF_WP_046996094.1 CRISPR-associated endonuclease Cas1 [Arcobacter butzleri]
LHINSFGLTLGISKNKFVVK-----------------------------------EYGK---------
VKQTYPFDKISRIILEGKGISISTDIIKKAVENNITIDFLN-KDAISYASLITYK---ASTTQK-
IQKQSMVLNTTLHLYLAKSFIKGKAKNQINYLKYLNK--YHK---------ILDSNIKKMELTY----KNIQ-
--KASTTNEVMG----YEGSISVMYWDSIKLIL-----EVPF------EARVTFGA-
KDIVNSSLNYAYAILYGKVQHSLVYAGLSLNISFLHALDEQ-
KPTLVYDMIEEFRTFVVDRTIFSMLNKNEPIKL-D-----------KNG-------------
LLNTKSKQLISKNIKEKLG----SYTMWKKESVKVE---NIIQTQCYKLSKAIDEQSE-TYKPFIGK
>PF_WP_025270209.1 CRISPR-associated endonuclease Cas1 [Hippea sp. KM1]
IHVNRFGVMLGVSRNKLVLK-----------------------------------KNRR---------
VVKSFPIKKVERIIFEGRGFLLSSDVIYRCAKEKIFLDFID-KKHFPYASISYYKA--STHQNI-
HKQAIILGTP-KQLEIAKSFVLGKLKNQKNYLKYLNK-----YH------DILDIEINGLSQSI--KKAE---
--KARDINSLMG----IEGGGSNLYWKAISKVI-----KRDF-------SRITKDA-
KDEINSALNYGYAILYGKIRQALIYAGLSLHISYLHTLDST-

KPTLVFDLIEEFRTYIVDRTIFSMINKNEKIEV-DL----------EG-------------
KLTLESKKNISKNIYERLA----SYTIYRKEQRKLE---NVIFMQALRLKDAILKDK--KYRPFIGR
>W_CRISPR_associated_endonuclease_Cas1_[Desulfobacca_acetoxidans]_(2)
328954440
VAVTSPGTFVGKQGGRLFIR-----------------------------------RERK---------
QVYSIPLIKVRGLNVHSSGVTLSSDLIKALSDKKLAIHFCT-PGGEPYALLHAPIS--PQATLS--
RLQLTASQSRTGLELAKEFVDGKIRNQLNLLKYYLR--SRQAETEDPYTSLYPEKSSILQRCQ----
QELGKLQLDEDYDTGRKRLFALEGQAGAAYWAMIKTLL---APEIDY------PGRERRGA-
VDLVNSLLNYGYGMLYPRVWRALLLAGLNPHISFLHSFQEQ-
KPTLAFDLIEEFRSQVVDRAILSMLTRGEKLTQ----I--------KSG------------
ALLTQETRRKVITQVVERLG------SLHPYRGQKVS-LEEIIRRQARLLADHLEGKK--RYRSFRGR
>PF_KHE91657.1 CRISPR-associated protein [Candidatus Scalindua brodae]
LIVTTPGHFVGKRGERIVVR-----------------------------------YKQK---------
IISDLPVIQLKGLTLSGRGTSISGDVIALCMKRGINIHFVDDNIGKITAIVSPPGGSSGEVSLL----
QITERDKESGLELAKMFILGKVKNQFALLKYYFK---YPLNQKNGYGKTFTENRHYLSDLI----
DKIKKTTGIYDPDAIRQRLMGLEGAFGATYWAVIKHLF---RNEVTF------SGRDRFGA-
NDVVNSALNYGYGILYGECLSAVIRAGLNPMAGFLHSYQSG-
KPTLTYDLIEEFRSFVVDRGIFAMFNRGERLEN----G--------EDG------------
LLTLDARKKIIKSVIGRLS------SEVWFKGRRVT-LQEVIREQAVNIKKHLTGKV--KYRPFLGR
>PF_WP_007220853.1 CRISPR-associated endonuclease Cas1 [Candidatus
Jettenia caeni]
LIVTTPGHFVGKRGERIVVM-----------------------------------SKQR---------
IVSELPVVRLSGLTLSGRGVSISGDVVELCMKKDVYIHFVD-NLGKIIAVVGPPGGSSGEVSLL----
QITERDKEKGLTLAKMFILGKVKNQFALLKYYFK---YPLNRENGFGKIFVERRQFLSDGI----
EKIKNATVLSDPETFRQQMMGWEGAFGAAYWEIVGHLF---RNGVQF------SGRVRHGA-
TDLVNSALNYGYGILYGDCLNAVIRTGLNPMAGFLHSYQTG-
KPTLIYDLIEEFRPFAVDRGIFTMLNRGERLEQ----G--------DDG------------
MLATETRKKISKSVISRLS------NEVWFHGRRLT-LREVIQEQAYNIKRHLSDKA--QYHPFLGR
>AC_M._Hollandica_DSM15978_(AGB49373.1)
LVINSYGSFLKKNHNCFLVR------------------------------------NED---------
KVFEVSADKVESILIATS-ATITTDAVKFAVENNIDIVFLD-HSGDPYGRIWHTKL--GSTTLI-
RRRQLEVAEKKKGFRMVKKWTEVKLNNQITFLKDLKK--NRPEQ-----KAELEEFIANIEKLQ----
AQLLE--LDGTLEEKRGSVMGVEGMASRHYFDALGYIM---PEKWKF------NGRSRNPA-
VDAFNCMLNYGYGILYSLVEKACIISGLDPYVGFLHTDNYN-KKSLVFDIIEMYRIH-
ADRTVVNLFSTKQVHDE-FFDS--I-----PNG-----------MSLNSNGKAVLIGAFNKALD------
QSVEYDGRNIK-IRDIIQHDCHKIANGLIK------------
>AC_NF_Methanosarcina_WH1 WP_052723155
VVIDEYGTYVHKKRNRFVTL-----------------------------------NNKD-------
KAIKWEFSADKVSQILIYRG-AAITADAIDLAVEKGIDIIYLD-RFGKPFARTYSCKF--ENSAAI-
HRCQVRAYDSEKGVQLMRGMIEAKVRNQSFLLKSLAK--NREDR-------ELEEVAEHIFSLC----AGLP-
--EGGDIDMSRNRLFGIEGEASREYFGALGTVL--PEKAYSG-------KRTKQPP-
EDLFNALLSYGYGILYTEVEKACVLAGLDPYMGFMHTDRPG-
KPSMVLDLMEEFRQPVVDRAAISLVSKKVVKPE-ELKA----V---ENG-----------
FYLDRSGRHKMVEAMSSRLS----KIITYR--NYRHS-FSTLVLRQAREVVKFICGQEE-AYSPFLYG
>AC_NF_Mehtanosarcina_MTP4 WP_052718431
VVIDEYGTYVHKKRNRFVTM-----------------------------------NNKD-------
KAVKWEFSADKVTQILVYRG-AAITADAIDLAVKKGIDIIYLD-KFGKPFARTYSCKF--ENSAAV-
HRCQVRAYDSEKGARLMRGMIEAKIRNQSYLLKSLAR--NREDR-------ELEETAEHILSLCAGLPEGGD-
--IDMSRNRLFG----IEGEASREYFGALGTVL--PEKAYSG-------KRTKQPP-
EDLFNALLSYGYGILYTEVEKACVLAGLDPYMGFLHTDRPG-
KPSMVLDLMEEFRQPVVDRAAISLVSKKAVKPD-ELKA----V---ENG-----------
FYLDRSGRHKMVEAMSSRLS----KIITYR--KDRHS-FSSLVLRQAREVVKFICGEEE-AYSPFVYG
>AC_NF_honorobensis AKB78446

------------------M----------------------------------DKTE-------
KLLKKGFSADKVSQILIYTG-AAVTADAIELAVKKGIDIVYLD-RMGKPFARTYSCGQ--ENSATV-
QRYQARAYDDGKGIFLMSSMIGAKIRNQAYFLKSLAK--NRDNERLAGQAERIMSQAEKIKKRA----
EEWGY--IEEARDSLFG----IEGEASRIYFQALSNVL--PEAVYS-------GTRTKRPP-
GDLFNAMLSYGYGILYTEVEKACILSGLNPYMGFLHTDLPG-
KPSLVLDLIEEFRQPVVDRSVLSLISKNLVTKN-DL------KPV-EGG------------
FYLNRAGRHKTLEAVSERLA------KVINYRDFRHS-FSSLVLLQARAVSKFLTGERN-GYAPFVYW
>AC_NF_Siciliae WP_082093010
LIVDEYGTYIHKKSNRFIFV----------------------------------DKTE-------
KLLKKGFSADKVSQILIYKG-AAVTADAIELAVKKGIDIVYFD-RLGKPFARTYSCEQ--ENSATV-
QRYQARAYDDGKGIFLMGSMIGAKIRNQGYLLKSLAK--KRNNERLVGQAERIMSLSEKIKERA----
EEWGY--IEEARESLFG----IEGEASRIYFQALSNIL--PEAVYS-------GTRTKRPP-
GDLFNAMLGYGYGILYTEVEKACVLSGLNPYMGFLHTDLPG-
KPSLVLDMIEEFRQPVVDRAVISLISKKLVTEK-DL------KPV-EGG------------
FYLNRAGRRKTLEAVSERLA------KTVNYRGFRHS-FSILILKQARAVAKFLTGESS-GYAPFVYW
>PF_WP_038137810.1 CRISPR-associated endonuclease Cas1 [Thiomicrospira sp. Milos-T1]
LVIAGDAQILTTDNHNLIVK----------------------------------KEDK---------
FTHKISWEQIHAITLIGL-HTMTLPAQHQALKNKIPVHLAD-RSGQYLGALTSFQPAQNSYKNW-
FIQLQMSDTHHFTLDIAKQIVYARIQNQKQTLFKRQA--HRK---------ELHTTFAKLKKLQ----HKVQ-
--RAEKLTTLNG----LEGAAAREYFACFNLFL---PKWAHF------EKRTRRPP-
KDPFNVLLSLGYTILYSHVDAILQSAGFMTWKGVYHQQSAA-HAALASDIMESYR-
HIIERFAIYVINHGQIKSE-DFRY----EKD-NLGVSI---------IRLSAEARRRYVSGLINRFQ------
----GFSKELT-LHQHLYSQAQTLKQAMLTQKTELFQPWKDT
>PF_WP_007469744.1 CRISPR-associated endonuclease Cas1 [Photobacterium marinum]
ICITGEPAYINCQGKRLHIQ----------------------------------QHNQ---------
VQAKLPFAHIHTLILFGN-HHITTPALKRLMKTGISVHFAN-QFGKHTGVAAALN---LSPELH-
LIQAAKLTNTENRLHFAKKLVYAKIENQIQVLNQRLQ------------------PTEELKQTQ----HSLK-
--NATTLKQCLG----YEGCASKAYYQALAHCL---PEGFLF------NGRIKRNP-
PDPVNSMLSYGYSMLYSHIDSLLRAAGLYPTLGGYHQGRGQ-HAALASDLMEPFR-
FIVERTMLTALNRHQLKPD-HFMT--------KEGQ-----------CRLTDAARKTWSKLLLDGLQ-----
RPNYHEGGKSYS-VLDAANQQNSHFIRWLRSEST-TFEPWSLK
>PF_KUI97421.1_(2) hypothetical protein VRK_36100 [Vibrio sp. MEBiC08052]
LCVVGEHCVLHLAKHRLVVN----------------------------------REDD---------
VVADLPISQLDTVVLFGH-HQITTQALTAAMGQGIHIYFCS-HSGKYEGVASLHA---GNPELY-
LQQVVYFKNQTTQLRFVKSLVTAKLRSQKEVLRRRDL------------------SFDPIDKLL----QQLT-
--HAKAIDECLG----IEGYAAKQYWGQLKLNL---GEEWHF------THRQKRRA-
QDPVNSMLSYGYSLLYSHVDSLIRTVGLSPANGGYHQARGT-HSALASDLMEPFR-
YIVDRSVFTLINTGQIKPA-DFYE--------KGGV-----------CWLDKAARKKYTGYLMDQLQ----
KPQFTDPDGEKRS-VLDQIVQQNHNLLQAIIGEAD-EFYPWQPR
>PF_WP_028302067.1 CRISPR-associated endonuclease Cas1 [Oceanospirillum beijerinckii]
LCLTGDNAFITLDQHRWVIE----------------------------------KKDE---------
VVADIPIKHLSTVVLFGK-HQITTQALTQAMEQGVSIHFAS-GFGRYQGCASQIE---HNPGFH-
LQQAAHFSQPENQLDFARALVAARIRSQKEVLRNRKV------------------NEPGLDQAL----EKLK-
--RITTLDQCLG----TEGNATKLYWEAFRSQI---PDDWEF------THREKRHA-
KDPINSMLSFGYSLLYSHTDSLLRSKGLFPTLGGYHQSRGT-HSALASDMMEPFR-
YLVERAVLSTIKTGQIKRN-QFFI--------NKGQ-----------CWLDKNARQFWCRYLVDQLQ----
KPQFTDPEGKKRS-ALDQIQQQNHNLKQWINGDIE-RFAPWQPR
>PF_WP_019606016.1 CRISPR-associated endonuclease Cas1 [Teredinibacter turnerae]
LCVSGAPALISTDHGRVLVQ----------------------------------RDDE---------
TVMSVPWQGLRSVLLLGR-HHLTHPAMIAALSQGVAIHFAS-RGGQYQGLLDGNQPR-LGPRLW-

LLQEERVADAAACVAVARELTNARIRHQREVLRQRAL----------------SGWHTLGDSL----SQVD-
--ACTDLSALQG----IEGAAARTYFAALAQAV---HPQWGF------HGRNRRPP-
RAPFNALLSLGFTLLYAHTETLLIIDGLNPRAGFYHKPHGS-HSTLASDMMEPFR-
HLVERCALSFLSSGKVKPA-DFSV--------KDNGA----------CELSNAARRLYLERLSERFE-----
TSMKGRDGSEGK-LIQLLRWQNRSLIELIRAKG--PFTAWLQR
>PF_ESQ17084.1 hypothetical protein N838_24440, partial [uncultured Thiohalocapsa sp. PB-PSB1]
LCVTGDPCVIVSRDKHLRVL----------------------------------RDDR---------
ALYHLPWSSLATVLLFGN-HQITTPAMQQALRRDIPIHLAT-GMGSYRGVLWSGSPREPGQNLW-
LRQAALFADPEQCLIAAIAIVSARLRHMKETLRLRNA----------------PGEDAIDAAL----RAAR-
--EAGAIESLRG----IEGSATRTYYLGIGASL---PAAFAF------ERRSRRPP-
RDPFNALLSLGYTLLYGYSEAMVRTVGLLPWRGFYHQGRGR-HAALASDLMEPFR-
HIVERSALTLVKRGQVRPE-DFTQ--------SPAGA----------CFMTSSARRTYLALLIDRFK-----
SQSRPLATRSRP---RC------------------------
>PF_CRI67871.1 putative RNA-directed DNA polymerase [Thiocapsa sp. KS1]
LLLTGAPCLIATKQGRLVAT----------------------------------RADQ---------
VVIESPWRQLQAVVCFGL-HHVTTPALRSAFSHHVPVHFAS-SGGAYQGSAWSGQAGSEGAGIW-
LDQQACFSHPEWAMAAARQIIMSRIRHMRELLRQRAP------------SGFQNTRDALQRAV----AEAE-
--RASDPSVLNG----IEGAATRAYFQALATSV---PAVYEF------DGRNRRPP-
RDPFNALLSFGYTLLYAHVDTLIRVAGLYPWIGFYHQPHGR-HAALASDLMEPFR-
HVVERAALRAVTRQGIKPE-EF------YLDPIKG-----------CRLSPTAMRRYLAMIWERLE----
APAESLGDAESRP-ILQQIHRQNRRLVEAVRGGT--PFSAWVSR
>PF_ESQ08042.1 hypothetical protein N838_03350 [uncultured Thiohalocapsa sp. PB-PSB1]
LMVTGASALVSSSRGRVVVT----------------------------------RDDQ---------
VIAEAPWRSLQAVLLLGP-HHITTPALRHAMAHDVPVHLAS-RGGHYQGLACNAQPGREGAALW-
LTQRQRCDDAIWSLAAAIAVVMARIRHMREVLRQRQP------------AGFVRERDALQRAL----NEAG-
--RADDRSMLNG----IEGQATRTYFAALAKLV---PDAYAF------QGRNRRPP-
RDPFNALLSLGYSLLHAQHITLLRVAGLYPWIGFYHQRHGR-HAALASDLMEPFR-
HIVERAALAAVGRGGIPPE-QF------HADPQRG-----------CRLAPPALKRYLADLWLRLE----
RPVGRIGEQDSYP-VLEQMQRQNRRLIDALRTGS--DFDAWVSR
>PF_WP_028885416.1 CRISPR-associated endonuclease Cas1 [Teredinibacter turnerae]
IILLGEPALISTHQGQVHIE----------------------------------QHSQ----------
TTHYPWNSLDSLIAIGA-HQFTTPALKTAMRQQIPVHFAN-HYGEYEGCCTGAAPQ-GGPGLW-
LLQHNSCNNAEQALAVSRELVCARIAQQHRLIQRRQS--------------TAPELTALKRLQ----KKAE-
--NATTLDRLRG----FEGTAARQLWAYFQRQL---SPEWGF------TGRKRRPP-
PDTIDALLSLGYTWLYNITDTLIRAVGLDSYRGYYHQPQGA-HAALASDLMEPFR-
HLVEGAVLTLVNRGQCRPDDGHHD--------GHG-----------LRLSSASRKQLITQLTETLL----
RPLSATAVSPANR--LEAMRIQVRAIEKACRYGE--PFRAWQE-
>PF_WP_018013804.1 CRISPR-associated endonuclease Cas1 [Teredinibacter turnerae]
LILLGEPALISTHQGQVHIE----------------------------------QHSQ----------
TTHYPWNSLDSLIAIGA-HQFTTPALKAAMHHQVPVHFAN-HYGEYEGCCAGAAPA-GGPGLW-
LLQHNSCNNTEQALAVSRELICARIAQQHRLIQRRQS--------------TAPELTALKRLQ----KKAE-
--NAVTLDRLRG----FEGAAARQLWAYFQRQL---SPEWGF------AGRKRRPP-
PDTINALLSLGYTWLYNITDTLVRSVGLDSYRGYYHQPQGA-HAALASDLMEPFR-
HLVEGAVLTLVNRGQCRPD----------DGHHDGDG----------LRLSSASRKQLITQLTETLL----
QPLSATAISPANR--LEAMRIQVRAIENACRYGD--PFRAWQE-
>PF_WP_045826479.1 CRISPR-associated endonuclease Cas1 [Teredinibacter sp. 991H.S.0a.06]
LILLGEPALISTHQGQVHIE----------------------------------QHSQ----------
TTHYPWNSLDSLIAIGA-HQFTTPALKAAMHHQVPVHFAN-HYGEYEGCCAGAAPA-GGPGLW-
LLQHNSCNNTEQALAVSRELICARIAQQHRLIQRRQS--------------TAPELTALKRLQ----KKAE-

--NAATLDRLRG----FEGAAARQLWAYFQRQL---APEWGF------AGRKRRPP-
PDTINALLSLGYTWLYNITDTLVRSVGLDSYRGYYHQPQGA-HAALASDLMEPFR-
HLVEGAVLTLVNRGQCRPD----------DGHHDGEG----------LRLSSASRKQLITQLTETLL----
RPLSATAVSPANR--LEAMRIQVRAIENACRYGE--PFRAWQE-
>PF_WP_028883449.1 CRISPR-associated endonuclease Cas1 [Teredinibacter turnerae]
LILLGEPALISTHQGQVHIE-----------------------------------QHSQ----------
TTHYPWNSLDSLIAIGA-HQFTTPALKAAMHHQVPVHFAN-HYGEYEGCCAGAAPA-GGPGLW-
LLQHNSCNNTEQALAVSRELICARIAQQHRLIQRRQS---------------TAPELTALKRLQ----KKAE-
--NAATLDRLRG----FEGAAARQLWAYFQRQL---SPEWGF------AGRKRRPP-
PDTINALLSLGYTWLYNITDTLVRSVGLDSYRGYYHQPQGA-HAALASDLMEPFR-
HLVEGAVLTLVNRGQCRPD---------DGH-HDGDG----------LRLSSASRKQLITQLTETLL----
RPLSATAVSPANR--LEAMRIQVRAIENACRYGD--PFRAWQE-
>W_CRISPR-associated_endonuclease_Cas1_[Marinomonas_mediterranea]
326793969
LIIAGDIAMLSSEKQRLIVE-----------------------------------QYDE----------
LHTYPWATLSSVLLVGP-HHITTPALKSAMFHNVPVHFAS-QYGRYQGVSAGAAPSVFGADFW-
LLQAQYLQQETNALNISQVLIQARIEGIRAVISRREK---------------DAPELNKIQRLD----EKRL-
--RAETLDQLRG----YEGQASKQLWAFFQRIL---EEDWGF------TGRNRRPP-
KDPINALLSLGYTYLYSLVDSVNRTVGLYPWQGALHQRHGY-HHTLASDLMEPWR-
YLVEHVVLTLINRHQIHKD-DFVIK-------ENG------------CEMSSGARKTLLKELLVQLT------
-KVPKGGNSLL-----TEMSNQSYRLALSCKMQQRFIAWSPK
>PF_WP_047875592.1 CRISPR-associated endonuclease Cas1 [Photobacterium aphoticum]
LIITGSLAFLTCHNNKLKVT------------------------------------QED---------
KDRYFSWQAIESVLLLGP-HHITTPALRMAMQQGIPVHFAS-RYGQYQGVACANQPS-HGYPLW-
LLQTVQFQQPDTALKYAKAVITARVTGQIALITRRDP--HWHGK-------------EALKRIK----QKIQ-
--RVEDSATLLG----HEGEAAKLLWQFFRESL---PEEWDF------NGRNKRPP-
TDPVNAMLSLGYTYLYHLCDTLIQMVGLCPWAGFYHQNHGA-HKTLASDLMEPIR-
VIVERVVLTMVHKRQLKPE-DFAL----T---DEG------------CEMSAAARKTFLTALLTALT------
--YQRSKKETR-LIDDILQQTRDVKMALKLDT--VFTPWTPQ
>PF_WP_038188758.1 CRISPR-associated endonuclease Cas1 [Vibrio sinaloensis]
LVIAGEVAMLTCENKRLKIT-----------------------------------QQDE----------
SRYYAWEELETILILGP-HNISTPCIRQAMHHQVAIHFAS-RYGQYQGVACSNMPS-QGHQLW-
QLQIAYLQKVDVALTWSIELVCAKIDGHIHLIRNRER---------------QSPLLEKLRTIK----RKAR-
--RSDDLQVLLG----IEGEAAKLQWEFFKQHL---DCEWQF------SGRNRRPP-
KDPINAMLSLGYTYLYHLTDSLIQSNGLCPWAGFYHQPHGA-HRTLASDLMEPLR-
VVVDRTVLALVAKRQIKPD-DFLIL-------EDG------------CEMSREARKVLLTQLLADLT------
-----TKRKKS--DRVIDQMITQVKEVKIATKL-ALNPNFWR
>PF_WP_055051199.1 CRISPR-associated endonuclease Cas1 [Vibrio metoecus]
LVVAGEVALLSSEEGRLKIT------------------------------------QEE---------
QAQFYSWDAIEAIFILGP-HNITTPCLRQAMLKQVPIHFAS-NYGQYQGVACANTPT-QGHHNW-
QVQMAHMQQIEVALHWSIELVVAKIDGHIHLIRNRDP---------------QSPLIEKLRHIK----HKAR-
--RSRELSTLLG----SEGEAAKLLWQFFAQQL---EDQWQF------TGRNRRPP-
RDPVNALLSLGYTYLYHLTDALIQANGLCPWVGFYHQPHGA-HRTLASDLMEPFR-
VVVERTALTLINKRQLKPE-DFVT----Q---TNG------------CEISREARKRFLTQLLADLT------
------GKRPK-QDRIIDQFIAQIREVKIALKL-GSEPKFWR
>PF_WP_055043549.1 CRISPR-associated endonuclease Cas1 [Vibrio metoecus]
LVVAGEVALLSSEEGRLKIT------------------------------------QEE---------
QAQFYSWEAIEAIFILGP-HNITTPCLRQAMLKQVPIHFAS-NYGQYQGVACANTPT-QGHHNW-
QVQIAHMQQIEVALHWSIELVVAKIDGHIHLIRNRDP---------------QSPLIEKLRHIK----HKAR-
--RSRELSTLLG----FEGEAAKLLWQFFAQQL---EEQWQF------TGRNRRPP-
RDPVNALLSLGYTYLYHLTDALIQANGLCPWVGFYHQPHGA-HRTLASDLMEPFR-

VVVERTALTLINKRQLKPE-DFVTQ-------TNG------------CEISREARKRFLTQLLADLT------
------GKRPK-QDRIIDQFIAQIREVKIALKL-GSTPKFWR
>DS_NF_Rhodomicrobium_vanielli WP_013419504
VHVLSGAAVVRVNNSTLLVE-----------------------------------RPGE---------
PVFERPIELVSTLHIHGW-ARVTGACIGRLTAQGATVVWRG-LHGYPVALAQPMHG--AGLDIR--
RAQYFEAAGERGLAIARALISAKIQNMRGLVRRRAN-------------IEGRDCLTALAALA----KKA---
-KHASRESLLG----IEGSATAFYFSAWPHMFAARAGDVEF------EVRSRRPP-
QNAVNATLSYAYAVLSAECVCALAAVGLDPRLGVFHQPRSG-
RASLALDLMEPFRPLIADQAVLTGFNTGQIRTGDAAEA--------DDG------------
WRLGETGKRTVIDLMEKRLT----TAISVSGTEQQVS-YREAIGRQARGIATALQTGA--GFEALERP
>DS_NF_Thioflavococcus_mobilis WP_015279346
LVVDRRDSVLRHDSGVLCLE-----------------------------------REGG---------
ETRRAPINQLELVVVYGN-PLAETAVWRKLAAAGVPTVLLP-VRGRDGAAVLGNGLA-TQLPLR-
RLQHRRAAEPARALELARWILAQKLASYDLPLQPLRR--RHGAD---------EAACAGLLRQRDRTLAALA-
--TARSRDELMG----LEGQLAHAWFALLAKRL---APAWGF------TGRNRRPP-
QDPINALFSLGYTLLGAEVHQGVVAAGLDPSLGFLHQPVPG-REAMVLDLTEPFR-
SAVDHFVLDWIAVDGPRQA-DYYY--------READG----------
CRLSKTARPGFFQAWASYRHHWPYAVRLDPSADWPASTLREQIRGQIERLRSAMKTPEAPS-------
>PF_WP_019956891.1 CRISPR-associated endonuclease Cas1 [Loktanella vestfoldensis]
VYVHEPGRVVTARGNAFVVL---------------------------------DAVGGD---------
EVALVHPGWADGIEIGPA-AAIDDDALRLALAMRLPVVFVA-GDGSLAGTLDPAPA--DRADLH-
LAQARLVLDPAARTDMVRRLVRGRMVNQRQTLKRLNA--KRDIM-------AVDDAVKDMRGLI----ARLP-
--VQGDVDALRG----MEGRGAAVYWTALQALL---DPVLGF------KGRERRPP-
PDPYNVAVSYMSTRLAGDMQALILRQGLHPGFACLHASQDG-
RPNLALDLMEEFRAPLVEGAAVTLFRQGRLKAG-MFV---------QEGDGR---------
LKASSVAIKAMITGYESRLR----TSLDNPADGKKTN-WRGLMEAQVIAYRRHCHGEG--IFECYNRE
>PF_KQB14189.1 hypothetical protein AP073_15680 [Rhodobacter capsulatus]
LYLFEPGYRLVAEREGFTVI-----------------------------------GHGR---------
ARLRLPATMISRIDLGAE-VEAEDAALRLAAAHGIPVALLN-EAGLPQSVLMPTLS--GDAALH-
MAQARLALDPVRAADLASLLVAGRIRNAQALLKRLNR--RRADA-------EVDQACERLKSQW----RKLE-
--VPRPLADVRA----IEAEAARSYWPALSACL---EHGFAL------PSRRDPGR-
ATAVNAVLDYTAALLTRDMRAAVLRARLHPGFGVLHVASDG-
HDACVYDLIEPFRAPLAEALTVYLLNNRILKAE-DFHL---EPPKSARAHG----------
VRLLPAAGRKLVQGYEAWVA----RAIIDPVAGHRTT-WRSLIGAEARRFARMAQDGT--PWTPYRMK
>PF_WP_019960649.1 CRISPR-associated endonuclease Cas1 [Woodsholea maritima]
LYLLEKGHVLGTHNSSFTVR----------------------------------NAQGD---------
PVSTLPTTRVDRIEVGSQ-ARIADEAIRLALDEDVEMRWIN-GRGQTEGYLSRPER--GHGALH-
LAQLRLYDNAEARLAAARILVEGRLRNQRALLRRLNR--RRKRP-------FIAERAKQIGGVL----KYLP-
--EAQTIEALMG----REGQAGALYWPALGACL---EHGWTF------TQRVRRPP-
PDPVNLVLSYLASLLCRDIASLAARQGLHVGIGALHAVQDEPRDTLAFDLAEEFRAPLVEGLCIWMLNTRTLG
HQ-MFHTR-------EDGQ-----------VYAHGEGIKTILRSWENWLD----RPVRSPRSGQDVL-
WRGLIEEQILCWRDHVSGKS--VYQPYRME
>PF_EJW09481.1_(2) CRISPR-associated protein Cas1 [Rhodovulum sp. PH10]
VYVIEPGRRLEAKGARLRLV-----------------------------------DDIG---------
RIVDLPPGQIDRIELGPG-TDATLEALELAAANAIEIDRVN-GHGEVTGRYEDVGP--ERARRH-
LAQARTVLDPARRLALARTIVDARLRNQRALLRRLNR--TRKDA-------EIAEACVVLNRTI----RSLD-
--TAADIDAAMG----YEGAGAAAFWPAFGRCF---PEPFRF------GRRSRRPA-
PDAANLAVNVVCNMLARDVRAVVIRAGLHPGFGVLHETADG-
IDAAVHDLMEELRAPVAEACVAASFNRKALTAD-SFVL--------DVG------------
TRLTRSGWAAVIRAYESWVA----QPVRSPRSRRDVL-WRALMLEQAYAYAEACEQGT--PYRPYEMD
>W_CRISPR_associated_endonuclease_Cas1_[Desulfarculus_baarsii] 302343124

MYLSRKGCRLDVRGRAFVVR-----------------------------------SGP----
EPDAPELMVVLPSQLDRVELWPG-CDISQKAQRFALECRTPVAYVD-GWGRTLGVLEPMVA--DKAALH-
LAQAAVALDETKRLALARLICAGRVRGQRALLMRLNR--RRKNS-------DIESNLAAFKQLP----RRIA-
--TATTISELLG----LEGEAAKRYWASLALLL---DKSWGF------SSRQRRPP-
RDGVNMVISYVASMLYRDLRCLAARHGLHPGFASLHGSLDG-
KPGCISDLVEEFRAPLCEGLAVYLANNHILKKE-MFYK--------TDKWP----------
CHVTPEGRETIIRAYEAWLD----RPVKSPRSGEKVK-WRGLLEEQVLAYRDHVMGRS--VYAPYDMK
>PF_WP_060836241.1 CRISPR-associated endonuclease Cas1 [Rhodovulum sulfidophilum]
LYLVTPGRRLGLRNLSFTVS---------------------------------GEEGR---------
ELLGIAPGRIDRIEIGAR-ADFAPEALRQALATGIDVAFLD-GRGQTEGWLSAGPP--ERADLH-
LAQARLVLDPVASAALARTIVEARLRNQRTKLRVLNR--VSKDP-------EVAAACTALGRVI----SKLP-
--GADTPAALRG----HEGSGAALYWPALGRLC--AAAPQPF--------RRTRPA-
TDPLNAAINWLTAMLERDIRAAIAHAGLHPGFGVLHVPADR-
SAACVWDLMEGYRAALTEGLAVTLFNQNRLRTG-MMTS----F---PGG------------
IRLGRDAIGALISGYEAAVG----RLVTSPYSGRRRT-WRWIMREEAQALARHMRDPEGMPFRPFLQT
>PF_WP_023924108.1 CRISPR-associated endonuclease Cas1 [Rhodobacter capsulatus]
LYLVEPERRLSLRAESFAVL-----------------------------------DGGG--------
SLLLSLPPARVNRIELGPG-AVVDDPVWRHALATDTTLAFLD-GHGETKGYLAPPQP--AEAELQ-
LAQAALVLHPERRLALARVLVGARLRNQRARLSVLNR--SAKAT-------EVDRAIAALGRAI----
RGLAA--PMPDLDSLRG----HEGRAARIYWPALARLC--TAHKGPF-------LRE-RPA-
KTPLNAAINYLTALLARDIRTALIGAGLHPGFGVLHQAQDR-
GDAAVYDLMEVFRATLSEGLAVSLFNRRRLREE-MFE---------PQGDS----------
LRILTPGRRALITGYEEALE----RVIRSSQTGQRHA-LRRIMLEEARALARHCRSPETVPYLPQMQD
>W_CRISPR-associated_endonuclease_Cas1,_partial_[Rhodobacter_capsulatus] 294676823
LYLVEPERRLSLRAESFAVL-----------------------------------DGG-------
GSLLLSLPPARVNRIELGPG-AVVDDPVWRHALATDTTLAFLD-GHGKTQGYLAPPQP--AEAQLQ-
LAQAALVLHPERRLALARVLVGARLRNQRARLAVLNR--SAKAT-------EVDRAIAALGRAI----
RGLAA--PMPDLDTLRG----HEGRAARIYWPALARLC--TAHKGPF--------LCERPA-
KTPLNAAINYLTALLARDIRAALIGAGLHPGFGVLHQAQDR-
GDAAVYDLMEVFRATLSEGLAVSLFNRRRLREE-MF-E--------PQGDS----------
LRLLTPGRRALITGYEEALE----RVIRSSQTGQRHA-LRRIMLEEARALARHCRSPETVPYLPQLQD
>W_CRISPR-associated_protein_[Tistrella_mobilis] 389875622
LYVVEPGRRLSVRNQAFTVE--------------------------------EIETGR---------
ELIALPAARVGRIELGHR-VAADARSLRHAAAARIPVALLD-GLGGVEAWLEPDGG--ARGQRH-
LAQARHILNPDSRLTLARLLVDARLQTTQALLKRLNR--RKRLA-------TVDAAADRLKRIR----RKLP-
--LAADIPQLMG----HEGEAAAIYWPALGALI---DHGWVF------RRRLRRNA-
ADPVNLILDWLASLLAREIKALATRHALDPGLGVLHEVRDG-
HDALVSDLIEIFRAPLAEACTVYLFNNRILGPD-DIIR---
YPPPPDAPDQAGVPGQPDGGWRVLPEAGRRVIRTWEGWLD----RPVTGP-DGRRRA-
WRGLIDTQLAAWCRHIEDDA--PFVPYHLD
>PF_WP_062763150.1 CRISPR-associated endonuclease Cas1 [Tistrella mobilis]
LYLLTPGHRLGIDDRAFTVE--------------------------------AAETGE---------
RLDSRPAAAVSRIELAPG-VQAAAATLRAALARDIPVALLD-GMGGVQAWLAPPPM--HQAPRH-
LAQARHLLDPARRHALARLVVDARLQNMQALLKRLDR--RKRLV-------SVEGAAERLKRIR----RKLR-
--VADTVPDLLA----VADEAALVYWPALGQLI--GHG-WTL------RRRLRRGA-
TDPVNLVLDWLTALLAREIRTAATRHGLHPGFGACHEVATA-
EDGLVADLVEVFAAPLVETCTVYLFNNRILQPD-DI-IR---RPL-
PRIAATDVGRGQDGGWRILPQPGRRVLETWEAQLD----RVATGPD-GEKAS-
WRALIDGRLASWAAHVEETG--VFRPCLID
>PF_EJW09347.1_(2) CRISPR-associated protein Cas1 [Rhodovulum sp. PH10]

LHVVSADRRLSLRNRAFAVE-----------------------------------
AGSGGPGAPPVWREILAIPHAAVDRIDLGPQ-AEVDPDALRHALATETLIAFVD-GHGETAGWLGPALS--
PRAGRH-LAQTRHAGDPALRLALAKRFVEGRVRNQRALLRRLNR--DRHDP-------TVLKALADINALL--
--RRLP---QADTLAALMG----HEGRATALYWPALSRLF--AHG-FRLGPAGEPPRRVREIV-
RDPVNILLNVAAALLARDVAVAAVRAGLHPGFGLLHATDDH-
RDACVFDLMEEFRAPLAESPVVQAINGRAIGDA-AF-E----P---R---P----DGG---
VRLKSEGFAAMVRAYERAVA----REVKSLRDGRRRT-WRGIMLDQALGLAAHVEGRE--TYAPYVID
>W_CRISPR_associated_endonuclease_Cas_Azospirillum_lipoferum_1 288957883
LYVTRRGRRLTRRNQSFAVT-----------------------------------EDGA---------
ELLAIPPARLDRIELGPG-CEADDDVLRHALAHGVDVALVD-GWGDSVGTLERPAG--PRAGLH-
LAQARLAIDPAARLDLARRIVDGRLRNQRALLRRLNR--KRADA-------DVTDAALALNRLI----RKLP-
--VAEDVPALMG----LEGAAAAAYWPALGRCL---EQGWRL------ERRRRRPP-
PDPVNLVISYLSAVLHRDLAAFVVRHGLHPGFGALHSARDG-
AQACVSDLIEEFRAPLVEGLAVYLFNNRILSRA-DFLTTDPPDGDAPDGQPGAAV------
CRIQSAGRDRLIRGYEGWLD----RAVKGP-SGKRML-WRRVMQAQVVAYARHALGEA--PYRPFILD
>W_CRISPR_associated_endonuclease_Cas_Azospirillum_lipoferum_2 374998939
LHVQTPRRRLALRNEAFTVV-----------------------------------ERDD----
LGGERELIAIHHHHLDRIDLGPE-ADADAEALRWALATDTELAFVD-GHGATHGRLTRPEA--RRAALH-
LDQARHALDEGLRLDLARRIVDGRLRNQRALLRRLNR--SRKLG-------VVEDAVLAINALL----RRLD-
--TAADVAALLG----FEGQGAARYWPALAAQI--EGE-WEF------EGRRRRPP-
PDPVNAVLSYLSGLLERDVAALIARHGLHPGFGVLHSPQDR-
HDAGIYDLMEEFRAPLMEGLAVTLFNRRTLRPD-HF-S----R---RET-G----
EGEIKGCRIDPDAVGAIIRAYEQWVR----RPVASPRDGKRTT-WRGLIGFQAQALAAHVQGRE--
PYRAYVMD
>PF_KJR40057.1 CRISPR-associated protein Cas1 [Candidatus Magnetoovum chiemensis]
VYLTSICTAAYSNGPSLIIK------------------------------------TDD---------
DNEDITWNSISRIVIIGR-SPFSGGVVYRALREDIPITFID-IMGRPTGHFHPENK--EYPELL-
ALQKQYAANENNCLTFAKEIVSAKIHNCDVLLRRNSI------------------KSDELESIL----YKIN-
--DASNLDVLRG----LEGSAAKIYFNELSNLV----LPFEF------KRRVYHPP-
DGPVNVMLSLGYTLLYNRIAVALKDKGFEPKQGFFHKPRGS-HAALASDLVEELR-
HIAERITLAVIHNKEIIKS-NF------RVFEKKGIEI---------ARLNGDGFRKFIRRFETTMA------
TKTSYSAHKAITYNAYIDEMADNLKRSLMLNI--NYKALRIH
>PF_WP_046007427.1 CRISPR-associated endonuclease Cas1 [Pseudoalteromonas rubra]
LYLTQVGGVVGIKGNRAYLN-----------------------------------LRGE---------
ELKTWPLNVIDAIIVLGR-IQLTTDFMGYCTEHAIVVILAS-STGRYRGELSTYPG--FNAVLY--
QQIVRCGNKAFERALCEQILQAKFHNCLQVIKQQLR--RRSLPESHKA--QLDHVITKLTKAA----NRLT--
-QTKTREHFFL----LEAKIAKHYFSAIALCV--NDT-WQF------NGRNRLPP-
KDPINALLSLGYNLLFNNTMCLVRKHGLHPDFGLLHCG-DN-
QPSLVLDLMEPFRAVIVDASVLKLINKQQITPA-DF-D--------FTGGQ----------
CLLKPETLKRFITVIEEKFC----ATFQSQRSHQVMD-YRKAMHDQVQALKCHFLSQA--PFSAFKVN
>PF_WP_038884984.1 CRISPR-associated endonuclease Cas1 [Vibrio rotiferianus]
VYIHKQGAAIHKRGNRLNVF-----------------------------------SQGK---------
PIQSIPANSIDMLLCFGA-VHLTTSVQRFCLENDICVVWLT-QSGQYLGCLSAPFQ--GDPMLT-
QAQVISRTTQETRLSLVRFFLRSKINNCLGVMRRHTH--KRQRD---------KPTEKRVVQLR----DQLE-
--HQKSVASMRG----TEGAAAREFFQWYRKQF---DNAWGF------TNRNRQPP-
KDPINAMLSYGYTLLFHNVRALVEARGLLPHLGYLHGSQPK-
QPALVLDIMEGYRPWVVDELVLQLANKQLITPD-QFEVS-------SKG------------
AWLNKEGRRVFIQSLEQRLL----KQYQHPTLKVKAD-IRRLIDLQVLELRQQLLSGQW-TLTQQRIR
>DS_vulnificus_RT-Cas1_(2) WP_011152750
VYIHKQGAAVHKRGNRINVF-----------------------------------FDGK---------
PLNSIPANTIDMMLCFGA-VHLTQAVQRFCLENDISVVWLS-QSGQYLGCMSAPFQ--GDPLLT-

AAQVNSRADPDVRNHLAKFFIRSKINNALGVMRRQGK--KQQRN---------KEQEKRIVHLR----EQLD-
--AHQTLASIRG----TEGAAAREYFSWFRQQF---SADWGF------NSRNRQPP-
KDPINAMLSYGYTLLFHNIRALVEARGLLPHLGYLHGSQPR-
QPSLVLDLMEGYRPWLVDELVLQLAGKKLITPE-QFTTH-------KNG-----------
VLLNREGKRLFIQSLEQRFL----SQHQHPTLKIKAD-TRRLIDIQVLELRHQLLSGQW-SLTQQRIR
>W_CRISPR-associated_endonuclease_Cas1_[Vibrio_vulnificus] 37677204
VYIHKQGAAVHKRGNRINVF-----------------------------------FDGK---------
PLNSIPANTIDMMLCFGA-VHLTQAVQRFCLENDISVVWLS-QSGQYLGCMSAPFQ--GDPLLT-
AAQVNSRADPDVRNHLAKFFIRSKINNALGVMRRQGK--KQQRN---------KEQEKRIVHLR----EQLD-
--AHQTLASIRG----TEGAAAREYFSWFRQQF---SADWGF------NSRNRQPP-
KDPINAMLSYGYTLLFHNIRALVEARGLLPHLGYLHGSQPR-
QPSLVLDLMEGYRPWLVDELVLQLAGKKLITPE-QFTTH-------KNG-----------
VLLNREGKRLFIQSLEQRFL----SQHQHPTLKIKAD-TRRLIDIQVLELRHQLLSG---QWSLTQQR
>DS_NF_H._aurantiacus_ABX05026.1
LYLNEQGTRLGKKDERLIIL-----------------------------------RGQE---------
LINDIPVIKVDRVIVMGQGVQVSHAAIVFLAQRGIPLIFTT-QSGGSQKAMVSAGL--
GNNAALRLAQCRIVDNPHLAVPLVQAIVVGKVANQIQLLERYGS----------DWGGMGLRAKQTMQHVI--
--QQTQ---HMPDIEQLRG----LEGAGAAAYWGTWSAVF---KTAWGF------AGRAYRPT-
PDPLNALLSFGYTLLLNDLMTAVQALSFDPYLGVFHTVQFG-
RPSLALDLEEEFRPCIVDRMVLDVLDAGLLQMS-NFSRT-------EKG-----------
FLLNDRARKSFIQAYEQRMQ----TPIRYQGTGNNEP-MRRVLLLQTQHLARVLQGEEP-RYQPYVWR
>DS_NF_Cloroflexus_aurantiacus_jf10/400 WP_012259223
LYIQEQGTTVRKRDNQIIII-----------------------------------RDGS---------
ILQEIPLNKVDQIVLMGRGVQISTALLVELLLRGIPVMITNQHGSRHYATLSAGPS--RFGPLR-
LRQSQLIIDPVWSLELVRGIVAAKLAAQRLILSGTGW-------------PSAAGAIAQIDTAL----QRLP-
--AATTLDIARG----FEGAAAAAYFGAWRNVF---QQGWGF------QGRAFHPP-
PDPINALLSFGYTLLLHDVLSAVQFTGLDPYLGVFHAVETG-
RPSLALDVMEEFRPLVVDRLVLSLITTGTITRS-QFVR---TQSEQPNA-----------
IYLTPDARKLVVSRYEALMK-----APFRLSDGTQTA-MRRVILLQTQMVARMIRGEQA-AYEGVIR-
>DS_NF_Roseiflexus_castenholzii WP_012121171
LYIQEQGVMVRKRDNQVLVT-----------------------------------RDGQ---------
TLHDVPLAKIDQVVLMGRGVQISTALLIDLLERGIPVTLTNQHGSRHYATLTAGPS--RFGDLR-
TGQMQYVNTPSRALELARAIVIVKLTNQRRLLATTGW-------------PAAASAMQQIDAAL----TAAS-
--QAQNVDILRG----HEGAAAAAYFGAWRASL---PPAWGF------GGRAFYPP-
PDPINAMLSFGYTLALHDVITAVQITGLDPYLGTFHVIETG-
RPSLALDLLEEFRPVIVDRMVLDIVRTNAIGRE-RF-H----RPQ-ERPEA----------
VYLDAEGRAFLVQRYETLLQ----TKVRLPG-GEQTP-MRRVILLQAQAIARVLRGEQE-RYTGFSLN
>DS_NF_Roxiflexus_sp1 WP_011957720
LYIQEQGVMVRKRDNQVLIT-----------------------------------KDGQ---------
TLSEVPLAKIDQVVLMGRGVQLSTALLIDLLERGIPVTFTNQHGSRHYATLTAGPS--RFGDLR-
IRQMQFVGAPDRALRLAKDIVSAKLTNQRRLLAATGW-------------PAAATAIAQIDAAL----TAAA-
--NAPHVDMLRG----HEGAAAAAYFGAWRASL---PPVWGF------GGRAFYPP-
PDPINAMLSFGYTLALHDVITAVQITGLDTYLGVFHVIEPG-
RPSLALDLLEEFRPLIVDRLVIDLVRTNAIGRE-HF-H----HPQ-ERPDA----------
VYLDDVGRTLLVQRYESMLQ----TKVRLPG-GEQTP-LRRVILLQAQAIARIVRGEQE-QYTGFSLN
>W_NF_F._fastidiosum CBL28737
IYLTQDNGVLGRNGEGFSWR-----------------------------------RSRK--------
EPPEKIPNTNLGDVVVVGN-GSITTPALHLLMDRNIPVHFVS-SGGRYKGSLTSGMG--HGYELR-
RAQYDSALSEDRSLDFARSFVVGKVLNQRQTLTRFMY--RRPGG-----GEVFEDAARELAILA----REAS-
--GSSCLEALRG----TEGNAARIYFSVLGNAL---IAPWRF------DGRTRRPP-
RDPVNALLSFAYTLLLGRVTTAVVTVGLDPCVGWLHPAYRG-
RPSASLDLMEEFRSAVVDRLVISILNQGFLSEN-HFSA--------GEGGG----------
VRMAQSAREKLMRLFKERLR----TEVVNSSNNHSSS-YENHIFAQAQSLARSIRDGD--SYLPFVLK

>PF_WP_009855610.1 CRISPR-associated endonuclease Cas1 [Rubrivivax benzoatilyticus]
VYVGEPGSVLRLDGERLLVS-----------------------------------RGRE---------
LLASVPLGQVDQLAVSAN-VLVSSALLRHCAHRRINVHLSDPGGGEAVASLDRGGW--PEMALL-
DAQRRACGSPAAGLPIARAILEGKLHNAKAVLRRFGR--REAPE----VRAVVDEAVAAADHAI----ARLA-
--LCANAAALRG----HEGTAARAHFQALAALL---PPALGF------GGRRRRPP-
PDPVNVLLSFGYSVLHANMASLLRLAGLNAHLGVLHAAAPG-
TLALASDLIEEFRAPVVDAVVLTVLREGQIGIG-DFDF----DDASETP------------
CRLRREGRQRFVRALECKLE----SRFVHPRLKAPTD-MRRAMQQQVRELVLVMQGRVP-RYLPLKFR
>DS_NF_f__Desulfobacca_acetoxidans WP_013706265
LYLSEQGACLQKTGERLVVA-----------------------------------KEGE---------
TLLDLPVGKVEAVLIFGN-VQFTTQAAHLLLQQGVEMALFT-RRGRLVGQLTSPFT--KNVTLR-
QAQYDRAADPEFALDLAKIIVGAKLTNSRGLLQEFAR--NHPES-------GLKGEIERLTELI----LQIG-
--GSPNLAALLG----LEGAAAHTYFQGLARMV--RHG-FGF------SGRQHHPA-
PDPVNALLSLGYTLVYNEISSLLDGMGFDPYMGFYHQPRYG-
HATLASDLLEEFRALLVDRLTLSLINNRVFGEQ-DF-F----RHE-PSG-G----------
MYLGDEPRKRYFQEYERYLT----NVHHCPENGGETD-FRRLFRRQAERLMRAVLLKG--TYQPYTFF
>DS_NF_f_kurnenia_stturgatiensis CAJ74579
--MTEQNSILRKSGDRLIIE-----------------------------------KDDK---------
VLLEVQCHKIDAVLIFGN-VQFTTQAVHELFEHGIEMAILT-RTGKLIGQITSPYT--KNITLR-
VQQFKQYWNDDFRLAFAKVIVCGKIQNCIQLVRSFSY--NHPRN-------SFDVEMDDLSLRL----NEVE-
--SAANISQLFG----IEGNAARVYFTSFGKMI--LSA-FAF------PGRKKYPS-
TDPVNALLSLNYTMIFNEISSLLDGLGFDPYLGYYHGIDYG-
RSSLASDLMEEFRAPIADRITLNLINNRIFCEE-DF-Y----ANP-STG-G----------
VYLKREPLKRYFVEYETMIN----REFLHPQQKENTT-FRKCFRIQAENLASHIQNNI--PYTSFVME
>DS_Pelodiction_RT_cas1_(3) WP_012509117
LYLQEQGSILRKDGERFSVE-----------------------------------KEGK---------
QLNDIIVRRVEQILVLGN-ITLTTPAMQYCMKSNIPITFVS-QHGSYFGRLEATTA--DNSALE-
RFQYLRSLDEPFALGIASAIVEAKIRNSRTMIQKRKA---MAWESNGELKEKFDASLLLMTSLA----EHTK-
--SCDNMEALRG----IEGKAAALYFELFGLLF---KKELPF--YTSAFRRVRRPP-
TDPVNSLLSFGYTLLHNNIFSLVRMKGMNPYLGFLHAEDKG-NPALINDLVEEFR-
TIIDSMTLYTLNKGVLRNK-DFYY----RKD-KAG------------CFLTDEARKKFLELFEQRMW----
AESLDPQSGKSLN-IRRHIESQVVKISEVLAGTRA-VYEPWRSE
>DS_chlorobium_phaeobacteroides_RT-Cas1_(3) WP_011745868
LYLQEQGSLMRKDGERFSIE-----------------------------------KDGS---------
VINEVIVRRIEQVVIFGN-VALTTPVMQYCLQNEIPVTFLS-QHGKYFGRLEATTA--DNAEMQ-
RFHFLRSIDEPFALETARSIVAAKISNSKTMIRRRKT---VVQDRDSTLQNKMAYNLDIMADLA----LKAE-
--ASTDIDALRG----IEGKASALYFECYGMLF---SKNLPF--HTRSFLRVRRPP-
TDPVNSLLSFGYTMLHTNIFSMVQASGLNPYIGFLHAERKG-NPALVNDLVEEFR-
TIVDSLVLYTLNRGLLQEK-DFYY----RKD-EPG------------CFLSNDARKRFLNIFETRMW----
QESRDGCTGKTLN-FRRHIEKQVRIMREVIAGTRT-QYDPYKLP
>W_CRISPR_associated_endonuclease_Cas1_[Chlorobium_limicola]_(2) 189346458
LYLQEQGSLMRKDGERFSIE-----------------------------------KDGA---------
VINEVIVRRIEQVVVFGN-IALTTPVMQYCMQNEIPVTFLS-QHGKYFGRLESTMA--DNAEMQ-
RYHFLRSIDEPFALETARSIVSAKIGNSRTMIRRRKS---VMQDCDGTLQSKMTCNLDIMADLL----LKAE-
--TSTDIDVLRG----LEGKASALYFECYGMFF---SKNLPF--HTASFLRVRRPP-
TDPVNSLLSFGYSLLHTNVFSMVQMSGLNPYIGFLHAERKG-NPALVNDLVEEFR-
TVIDSLVLYTINRGLLHEN-DFYY----RKD-QPG------------CFLSNDARKRFLQIFETRMW----
QESRDGYTGKTLN-FRRHIEKQVRIMRDVISGTRT-QYDPYKLV
>PF_KFB76584.1 CRISPR-associated endonuclease Cas1 [Candidatus Accumulibacter sp. SK-02]
LYLLENGAVLNKEGERFIVA-----------------------------------RHGE---------
VLLQVPMMRIDQIMVFGN-VQITTPALHECLERGIPVMLLS-GRGRFFGVIDPLDA--RSVPLQ-

RAQFALESDEPARLALARPLIAGKILNCRTFLGRLAR--ARQTN--------MDAPLAALKSAA----QAAG-
--QAADLEILRG----IEGAAARTYFAAWQTVL--PAK-WQF------TGRNRQPP-
TDPVNALLSYGYTIVFYNVLALVRARGLNTHVGVLHDVRPG-
HPALASDLMEEFRAPVVDAVVMHLVFDGKLQPG-DF-S----WPE-TPGQP----------
CLMADGSRKHFIHLLEQKLN----TTVSH--AGQRLD-YRRWMDMQVLQYAAALRTPGL-PYVPFAIR
>PF_WP_027150711.1 [Methylobacter tundripaludum]
LYLLQHGQVLGKESERLIVR-----------------------------------QEQK---------
TVREIPAIKVDQIIVFGN-AQITTQAMQFCLQERIPIYLLS-GQGHYYGVIDSFNT--EPVLLQ-
REQFLRANDNAFCLKLAAAMVHGKIANSRLMLQRQAR--RHDSA-------ALHTAADALKNTL----AHLG-
--GASSLDELRG----FEGSAANAYFQALSATV---DDSWGF------SRRVRQPP-
TDGINAMLSYGYTLLFYNIYSLLRSRGLNPQVGFLHALRQG-
HPALASDMMEEFRSIVVDAVVWKLAVNRQLTPD-DFDY----PKTAGEG------------
CFLKPHARQTFIKALEEKFN----SAIAHPVTGTALD-YRRCMEYQVQHLSQVIRGIDA-DYQAMVLR
>PF_WP_052166286.1 [Methylobacter tundripaludum]
LYLLQHGQVLGKESERLIVR-----------------------------------QEQK---------
TVREIPAIKVDQIMVFGN-AQITTQAMQFCLQERIPIYLLS-GQGHYYGVIDSFNT--EPVLLQ-
REQFLRANDDAFCLKLAAAMVHGKIANSRLMLQRQAR--RHDSA-------ALHTAADALKNTL----AHLG-
--GASSLDELRG----FEGSAANAYFQALSATV--DDS-WGF------SRRVRQPP-
TDGINAMLSYGYTLLFYNIYSLLRSRGLNPQVGFLHALRQG-
HPALASDMMEEFRSIVVDAVVWKLAVNRQLTPD-DF-D----YPK-TAGEG----------
CFLKPHARQTFIKALEEKFN----SAIAHPVTGTALD-YRRCMEYQVQHLSQVIRGIDA-DYQAMVLR
>PF_ETX03376.1 hypothetical protein ETSY2_33635 [Candidatus Entotheonella sp. TSY2]
LYLMEQGAVLAKENERFVVR-----------------------------------KSGA---------
VLRKIPALKVDQILVFGN-VQITTPAMHFCLLEDIPIFLLS-SRGRYYGVVESSAT--DKVLLH-
RDQFARMAEPGFGLQIARELVRGKVANSRALLLRSAR--RRSHE-------GLRLAANALQQIQ----DRLH-
--EAASLETLRG----MEGTAAARYFAVWPELL--GTD-WPF------PGRKRRPA-
PDPVNALLSFGYTLLFYNTYALVHAQGLHPHVGVYHALQTG-
HAALVSDLMEEFRAPVIDATVLALLHRRQVRPD-DF-R----MPA-EAGMP----------
CRLTDEARKKVTQAFEKAFS----RRVTHPDAGGSCD-YRRAISLQAQRLVAVIKGEQT-RYQPFIRR
>PF_WP_024971209.1 CRISPR-associated endonuclease Cas1 [Microcystis aeruginosa]
LYLIEQGSYLKVKNQQFQVY-----------------------------------YQEA---------
LKISVPVNRVSHILIFGC-CNVSEGAVKISLQRRIPIMFLS-QKGYYFGRLQAEGM--AEIDYL-
SKQVELSQDPAFILEQARSIVMAKLRNSRILLLRLNR--QEKTE-------LATRAIGQLAEFL----EKVE-
--RGDSRESLLG----YEGQGARVYFQGLGSLL--KEP-FEL------TQRTRRPP-
TEPVSSLLSLGYTLLFQNLYSLVQSVGLHPHFGNLHVPSRS-
HPALVSDLIEEFRSPIVDSLVVYLVNSQILNLE-DF-T----PPD-ARG-G----------
VYLYPDALKKYLKHWQDRLQ----LEMTHPRTGYKVS-YHRCLELQVWEYIACLTGESE-VYHPLLLS
>W_CRISPR_associated_endonuclease_Cas_Arthrospira_platensis_(2) 479129286
LYVTDQGAYVKVKHQQFQVL-----------------------------------LGND---------
LKVSIPVNVVDYIILFGC-CNLSHGAIGLALRRRIPILFLS-DQGRYFGRLQTDGM--TRVDYL-
SRQVHCAEDETFVLRQAKVIVAGKLHNCRILLRRLNR--DRQIS-------QVIEAIEELGVWQ----EKIA-
--EVELLESLLG----YEGFGTRIYFQALRALV--QPP-FTF------EHRTRRPP-
TDPVNSLLSLGYTLLHQNIHSLILAVGLHPHYGNLHVPRSN-
HPALVSDLIEEFRAPVVDSLVIYLVNSGIFTPE-DF-T----PSD-ERG-G----------
VYIYSDALKKYLKHWHDKLS----LKTTHPHTGYKVS-YYRCLELQVWEYISCLMGERE-VYRPMLTK
>PF_WP_008049584.1 CRISPR-associated endonuclease Cas1 [Arthrospira sp. PCC 8005]
LYVTDQGAYVKVKHQQFQVL-----------------------------------LGND---------
LKVSIPVNVVDYIILFGC-CNLSHGAIGLALRRRIPILFLS-YQGRYFGRLQTDGM--TRVDYL-
SRQVHCAEDETFVLRQAKVIVAGKLHNCRILLRRLNR--DRQIS-------QVIEAIEELGVWQ----EKIA-
--EVELLESLLG----YEGFGTRIYFQALGALV--QPP-FTF------EHRTRRPP-
TDPVNSLLSLGYTLLHQNIHSLILAVGLHPHYGNLHVPRSN-

HPALVSDLIEEFRAPVVDSLVIYLVNSGIFTPE-DF-T----PSD-ERG-G---------
VYLYSDALKKYLKHWQDKLS----LKTTHPHTGYKVS-YYRCLELQVWEYISCLIGERE-VYRPMKLE
>PF_WP_006620498.1 CRISPR-associated endonuclease Cas1 [Arthrospira platensis]
LYVTDQGAYVKVKHQQFQVL-----------------------------------LGND---------
LKVSIPVNVVDYIILFGC-CNLSHGAIGLALRRRIPILFLS-YQGRYFGRLQTDGM--TRVDYL-
SRQVHSAEDETFVLRQAKVIVAGKLHNCRILLRRLNR--DRQIS-------QVIEAIEELGVWQ----EKIA-
--EVELLESLLG----YEGFGTRIYFQALGALV--QPP-FTF------EHRTRRPP-
TDPVNSLLSLGYTLLHQNIHSLILAVGLHPHYGNLHVPRSN-
HPALVSDLIEEFRAPVVDSLVIYLVNSGIFTPE-DF-T----PSD-ERG-G---------
VYLYSDALKKYLKHWQDKLS----LKTTHPHTGYKVS-YYRCLELQVWEYISCLMGERE-VYRPMKLE
>PF_WP_006669920.1_(2) CRISPR-associated endonuclease Cas1 [Arthrospira maxima]
LYVTDQGAYVKVKHQQFQVL-----------------------------------LGND---------
LKVSIPVNVVDYIILFGC-CNLSHGAIGLALRRRIPILFLS-YQGRYFGRLQTDGM--TRVDYL-
SRQVHCAEDETFVLRQAKVIVAGKLHNCRILLRRLNR--DRQIS-------QVIEAIEELGVWQ----EKIA-
--EVELLESLLG----YEGFGTRIYFQALGALV--QPP-FTF------EHRTRRPP-
TDPVNSLLSLGYTLLHQNIHSLILAVGLHPHYGNLHVPRSN-
HPALVSDLIEEFRAPVVDSLVIYLVNSGIFTPE-DF-T----PSD-ERG-G---------
VYLYSDALKKYLKHWQDKLS----LKTTHPHTGYKVS-YYRCLELQVWEYISCLMGERE-VYRPMKLE
>PF_KPQ33062.1 CRISPR-associated endonuclease Cas1 [Phormidesmis priestleyi Ana]
LYVTEQGAYLRVRHSQFQVF-----------------------------------HEQE---------
LRISIPANNITFVVMFGA-CTVSHGAVRLALQRRIPLLYLS-NKGRYFGRLQTTGQ--AKIEYL-
VAQVTKSQDEDFVRRQAMNVIVGKLHNSRKLLMRLNR--RRKTD-------LATKAVKEIAVLI----KQVQ-
--QAETVESMLG----YEGQGASLYFRAYGSLL--KGE-FGF------DKRTRRPP-
TDPTNSLLSLGYTLLSQNVHAMTEAAGLHTHFGNLHVPQSH-
RPSLVCDLVEEFRAMVVDSLVAYLINSNIYTPE-DF-T----PPD-GRG-G---------
VYLHTDAMKRFLKHWEEKLQ----SETVHPHTGYKVS-LRRCFELQVWEYAACISGERE-EYRPMVWQ
>PF_WP_008312855.1 CRISPR-associated endonuclease Cas1 [Leptolyngbya sp. PCC 6406]
LYVTDQGAYLRVQHQQFQVF-----------------------------------HQQE---------
LRCSVPASRISHVVLFGC-CNVSHGAVRLALQRRIPLLYLS-NKGRYFGRLQTTGQ--AKLDYL-
TQQVYKAQDPEFIRTQAASIIVGKLHNSRILLQRLNR--RRKTE-------LATQAIDTLAELM----QTVP-
--SVESVEAMLG----YEGTGASAYFQAYASLL--KGE-FEF------EKRTRRPP-
TDPINSLLSLGYTLLSQNVHAMVEGVGLHTHFGNLHVPREN-
RPSLVCDLVEEFRAVVVDSLVAYLINSNIFKAD-DF-T----PPD-ERG-G---------
VYLYPDAMKRFLKHWEEKLQ----HTVTHPHTGYKVS-YRRCFELQVWEYVACLTGEQS-VYRPMRME
>PF_WP_035991689.1 CRISPR-associated endonuclease Cas1 [Leptolyngbya sp. KIOST-1]
LYVTDQGAYLRVKQQQFQVF-----------------------------------HQQE---------
LRCSVPASQISHVVLFGA-CTVSHGAVRLALQRRIPLLYLS-NKGRYFGRLQTTGQ--AKLEYL-
TQQVYKSQDPDFIRTQAASTIVGKLHNSRILLLRLNR--RRKTE-------RATQAIATLAELM----ETVP-
--TADSVEAMLG----YEGTGANQYFQAYASLL--KGK-FEF------EKRTRRPP-
TDPVNSLLSLGYTLLSQNVHAMVEGVGLHTHFGNLHKPQAN-
RPSLVCDLVEEFRAIAVDSLVAYLINSNIFKPD-DF-T----PPD-ERG-G---------
VYLHPDAMKRFLKHWEEKLQ----QSVTHPHTGYKVS-YRRCLELQVWEYVACLMGEQP-VYRPMRME
>W_CRISPR_associated_endonuclease_Cas1_[Cyanothece_sp._PCC_7822]_2_(2) 307592471
LYITDQGSYLKAQGYQFHIF-----------------------------------YQRE---------
LRCKIPVNQVSHIVLFGC-CNITHGAVRLALTRRIPLLYLS-QRGRYFGRLETEGQ--AKVEYL-
SQQVKRAENPEFTRLQAENIVRAKLNNSRVLLMRLNR--RRSNS-------SVKQAIANLEKLR----DNLP-
--LANSMDELRG----YEGKAATVYFQALGSLF---QAPFTF------EKRSKRPP-
TDPVNSLLSLGYTLLSQNMHSFVEAMGLHTHFGNLHVPRNN-

HPALVSDLIEEFRAPLVDSLVVYLVNKKIFTPE-DF-T----PPDGRNG-----------
VYLHPDSLKKFLKHWEEKLH----SEMTHPYTQYKVS-MRRCLELQVREYIASLMGDTD-GYRPMLWT
>W_CRISPR_associated_endonuclease_Cas1_[Cyanothece_sp._PCC_7822]_1_(2)
307591462
LYITDQGSYLKAQGYQFHIF-----------------------------------YQRE---------
LRCKIPVNQVSHIVLFGC-CNITHGAVRLALTRRIPLLYLS-QRGRYFGRLETEGQ--AKVEYL-
SQQVKRAENSEFTRLQAENIVRAKLNNSRVLLMRLNR--RRSNS-------SVKQAIANLEKLR----DNLP-
--LANTMDELRG----YEGKAATVYFQALGSLF---QAPFTF------EKRSKRPP-
TDPVNSLLSLGYTLLSQNMHSFVEAMGLHTHFGNLHVPRNN-
HPALVSDLIEEFRAPLVDSLVVYLVNKKIFTPE-DF-T----PPDGRNG-----------
VYLHPDSLKKFLKHWEEKLH----SEITHPYTQYKVS-MRRCLELQVREYIACLMGDTD-GYRPMLWA
>W_CRISPR-associated_endonuclease_Cas1_[Microcoleus_sp._PCC_7113]_(2)
428314604
LYITDQGAYLKVEHQQFVVF-----------------------------------YQGE---------
LRCKVPVNRVSHIVLFGC-CNLSHGAVSLALRRRIPVLYLS-QKGRYFGRLQTEGQ--AEVEYL-
VRQVQCALDPDFTRRQAKSIVLGKLHNSRILLMRLNR--RVKSE-------KAAKAIDSLALLM----LDVP-
--DAESIEVLLG----YEGHGSHLYFQALGTLF--KEP-FVF------EKRTKRPP-
TDPVNSLLSLGYTLLSQNMYSMVQTVGLHPHFGNLHVPRNN-
HPALVSDLMEEFRALLVDSLVAYLVNSKIFVLE-DF-T----PPD-ERG-G---------
VYLFPDALKKYLKHWEERLQ----SKMTHPHTGYKVS-NRRCLELQVWEYIACLMGEQE-IYRPMTWK
>W_CRISPR_associated_endonuclease_Cas_Anabaenacylindric_(2) 440685177
LYITDQGAYLSAKNQQFQVY-----------------------------------YQQE---------
LRIKIPVVRVTNIVLFGC-CNVSHGAVSVALHRRIPVMYLS-QKGRYFGRLQTEGM--AKVEYL-
ARQVECSQNFEFTKKQAEAIVRAKLHNSRIMLMRLNR--RVKSE-------KATQAIAQIELFM----DKLP-
--FSEDVNMLRG----YEGIAATTYFQALGSLV---TGKFTF------EKRTKRPP-
TDPINSMLSLGYTLLSQNVHSLIQSVGLHTHFGNLHVPRDN-
HPALVSDLMEEFRALVVDSLVIYLVNRNLFTID-DFTS-----PDERNG-----------
VYFQPHALKKFLKHWEEKLQ----TEVTHPHTDCKLP-YRRCIELQVREYIACLMGEVE-VYRPMIWD
>W_CRISPR_associated_endonuclease_Cas_Calothrix_parietina_(2) 428297029
LYVTDQGAYLSMKNQQFQVF-----------------------------------YQGE---------
LKIKIPVVRVSSIVMFGC-CNVSHGAVSMALIRRIPIMYLS-QKGRYFGHTAVQGN--AKVEYL-
MKQVICCQNPVFTRQQAETIVAAKLHNSRILLMRLNR--RRETE-------IATQAIDFIEILI----DSLP-
--KAESMDALRG----YEGKAATIYFQALGSLF--TGF-FAF------DKRTKRPP-
TDPINSLMSLGYTLLSQQVFSFVQSVGLHTHFGNLHVPRDN-
HPALVSDLMEEFRAQIVDSFVSYIVNKKILTPE-DF-T----SPD-ERG-G---------
VYLQASALKKYLKHWEEKLQ----SETTHPNTGYKVA-YRRCIELQVREYVACLVGEVE-VYRPMTWK
>PF_WP_041039832.1 CRISPR-associated endonuclease Cas1 [Tolypothrix
campylonemoides]
LYVTDQGAYLSVKNQQFQVF-----------------------------------YQGE---------
LKIKVPATRVNNIVMFGC-CNVSHGAVSMALRRRIPIMYLS-QKGRYFGHTAVQGD--ARVEYL-
MQQVKCCENTQFTRQQAEAIVAAKLHNSRILLMRLNR--RRPTE-------IATQAIDLIEILI----DSLP-
--QAESLDALRG----YEGKAATVYFQALGSLF--TGF-FAF------DKRTKRPP-
TDPINSLMSLGYTLLSQQVFSFIQSVGLHTHFGNLHTPRDN-
HPALVSDLMEEFRAQIVDSFVAYLVNKKILTPE-DF-T----PPD-ERG-G---------
VYLQASALKKYLKHWEEKLQ----TETTHPHTGYKVA-YRRCIELQVREYIACLVGEVE-VYRPMVWK
>PF_WP_007355619.1 [Kamptonema]
LYVTDQGSYLSIYNQQFQVF-----------------------------------YQRE---------
LRIKVPASRVSHIILFGC-CNLSHGAVSLALHGRIPIMYLS-QRGRYFGRLQAEGM--AKVEYL-
ERQVIGSQSSKFVRQQAEVIVWTKLHNSRALLMKLNR--RRPSK-------IAAIAIEAILQLM----EDLH-
--QAASMDALRG----YEGQAASLYFQGLGSLF--TGS-FVF------EKRTKRPP-
TDPINSLLSLGYTLLSQNVHSFVQAVGLHTHFGNLHTPRDY-
HPALVSDLMEEFRAQVVDSLVAYLVNSKIFVLE-DF-T----PPD-ERG-G---------
VYLQPYALKKYLKHWEEKLL----SEVTHPHTGYQVS-LRRCMELQVREYIACLMGEVE-VYRPMLWK
>W_CRISPR-associated_endonuclease_Cas1_[Nostoc_sp._PCC_7107] 427708216

LYITDQGAYLSVKNQQFQVY-----------------------------------YQGQ---------
LRIKVPVSRVSNVVLFGC-CNVSHGAVSMALRRRISIMYLS-QKGRYFGRLQVSGD--AKVEYL-
MLQVERCQNPEFTRTLAEVIVTAKIHNSRILLMRL-K--RRKSSEFDDN--LVKEAIKDLDILM----DKLP-
--FAESMDALRG----YEGRAATVYFQALGSLF--SGA-FKF------EKRTKRPP-
TDPVNSLLSLGYTLLSHNIFSFIQAVGLHSHFGNLHVPRDN-
HPALVSDLMEEFRAQLVDSLVAYLINSNIFTED-DF-T----PPD-EKG-G----------
VYLQPHALKKFLKHWEEKLQ----SELTHPNTGYKVS-FRRCLELQVREYVACLTGEVK-IYRPMIWK
>PF_BAU08380.1 hypothetical protein FIS3754_43240 [Fischerella sp. NIES-3754]
LYVTDQGAYLSVKNQQFQVF-----------------------------------YQGE---------
LRIKIPVSRVSNVVLFGC-CNVSHGAASLALRRRIPIMYLS-QKGRYFGRLQVEGD--AKVEYL-
MRQVECCQNPEFTRRQAEAIVTAKIHNSRILLMRLKR--RKSSEFDDN---LVKEAIKDLDTLM----DKLP-
--FAESMDALRG----YEGRAATVYFQALGSLF---SSVFKF------EKRTKRPP-
TDPVNSLLSLGYTLLSQNVFSFIQAIGLHGHFGNLHVPRDN-HPALFS------------------------
----

>PF_WP_035139015.1 CRISPR-associated endonuclease Cas1 [Fischerella muscicola]
LYVTDQGAYLSVKNQQFQVF-----------------------------------YQGE---------
LRIKIPVSRVSNVVLFGC-CNVSHGAASLALRRRIPIMYLS-QKGRYFGRLQVEGD--AKVEYL-
MRQVECCQNPEFTRRQAEAIVTAKIHNSRILLMRLKR--RKSSEFDDN---LVKEAIKDLDTLM----DKLP-
--FAESMDALRG----YEGRAATVYFQALGSLF---SSVFKF------EKRTKRPP-
TDPVNSLLSLGYTLLSQNVFSFIQAIGLHGHFGNLHVPRDN-
HPALVSDLMEEFRAQLVDSLVAYLINSNIFTED-DF-T----PPDEKGG-----------
VYLQPHALKKFLKHWEEKLQ----SELTHPNTGYKVS-FRRCLELQVREYVACLMGEVK-IYRPMIWK
>W_CRISPR_associated_endonuclease_Cas_Calothrix_sp_(2) 427717966
LYITDQGAYLSVKNQQFQVY-----------------------------------YQGE---------
LRIKVPVSRVSNIVLFGS-CNVSHGAVSMALRRRIPIMYLS-QKGRYFGRLQAEGE--ARVEYL-
MLQVERCQNSEFTRRQAEAIVKAKIHNSRILLMRLNR--RQKSKNVDET--IIKKASNELEILM----SKLP-
--FADNMDVLRG----YEGRAATIYFQALGNLF---SGSFTF------EKRTKRPP-
TDPINSMLSLGYTLLSQNVYSFIQSVGLHTHFGNLHVPRDH-
HPALVSDLMEEWRAGLVDSLTVYLVNSEVFTID-DFTL-----PDERNG-----------
VYFQPHALKKFLKHWEEKLQ----SEVTHPHTGQKVV-YRRALELQVREYISCLKGEVE-VYRPMIWE
>PF_WP_033334699.1 CRISPR-associated endonuclease Cas1 [Scytonema hofmannii]
LYVTDQGAYLSVKNQQFQVF-----------------------------------YQGE---------
LRIKLPVSRVSNVVLFGC-CNVSHGAVSMALHRRIPIMYLS-QKGRYFGRLQVEGD--AKVEYL-
MRQVECCQNPDFTRRQAEAIVRAKLHNSRILLMRLNR--RRKSQDVDET--VIKTAAHDLEVLM----SKLP-
--FAENMDMLRG----YEGKAATIYFQALGSLF--SGS-FKF------EKRTKRPP-
TDPINSMFSLGYTLLSQNVFSFVQTIGLHTHFGNLHVPRDN-HPALVSDLVEEWRAS-
VDSLVAYLVNSQIFTID-DF-T----LPD-ERG-G----------VYFQPHALKKFLKHWEEKLQ----
SELTHPHTGQKVV-FRRAIELQVREYISCLKGEVE-VYRPMVWE
>PF_WP_044448019.1 CRISPR-associated endonuclease Cas1 [Mastigocladus laminosus]
LYVTDQGAYLSLKNQQFQVF-----------------------------------YQGQ---------
LRIKVPIVRVTNIVLFGC-CNVSHGAVSMALRRRIPIMYLS-QKGRYFGRLETEGQ--AKVEYL-
ARQVECSRDDKFTRQQAEVIVWAKLHNSRALLLKLNR--RRPSK-------IALSAINLIADLM----DNLP-
--QAESMDALRG----YEGKAATLYFQALGSLF--TGV-FAF------DKRTRRPP-
TDPINSMLSLGYTLLSQNVYSFIQAMGLHTHFGNLHVPRDN-
HPALVSDLVEQFRAQLVDSLIAYLVNSNIFTIE-DF-T----NPD-ERG-G----------
VYFQPHALKKFLKHWEEKLQ----SEITHPHTEHKVA-FRRAIELQVREYIACLMGEVE-VYRPMIWK
>W_CRISPR-associated_endonuclease_Cas1_[Nostoc_sp._PCC_7120]_(2) 17228961
LYITDQGAYLSVKNQQFQVF-----------------------------------HQGE---------
LRIKVPVMRVSNVVLFGC-CNVSHGAVSMALRRRIPIMYLS-QKGRYFGRLQTEGD--AKVEYL-

MLQVERCQNHEFTRKQAEAIVRAKLHNSRALLLKLNR--RHPSK-------IAATAISGIAELM----EKLS-
--LAESMDSLRG----YEGKAATLYFQGLGSLF--TGA-FVF------EKRTKRPP-
TDPVNSLLSLGYTLLSQNVFSFVQVIGLHTHFGNLHVPRDN-
HPALVSDLMEEFRAQLVDSLVAYLINSKIFTFE-DF-T----PPD-EKG-G----------
VYLQPHALKKFLKHWEEKLQ----SEVTHPHTGYQVS-LRRCLELQVREYITSLTGEVE-LYRPMIWK
>PF_WP_029630506.1 CRISPR-associated endonuclease Cas1 [[Scytonema hofmanni] UTEX B 1581]
LYVTDQGAYLSVKNQQFQVY-----------------------------------YQGE---------
LRIKVPATRVTNIVLFGC-CNVSHGAVSYALRRRIPIMYLS-QKGRYFGRLQTEGN--AKVEYL-
MRQVICSQNPEFTRKQAENIVWAKLHNSRALLLKFNR--RRPSK-------MASSAIELIADLM----DKLP-
--QAESMDALRG----YEGKAATLYFQALGSLF--TGV-FEF------DRRTKRPP-
TDPINSLMSLGYTLLSQNVFSFVETVGLHTHFGNLHVPRDN-
HPALVSDLMEEFRAQLVDSLVAYVVNSKILTLE-DF-T----PPD-EKG-G----------
VYLQPHALKKFLKHWEEKLQ----SELTHPHTGYQVS-FRRCVELQVREYVACLMGEVE-LYRPMIWK
>DS_NF_Cyanotheque_7424 WP_012599796
LYLMEQGTWVQKEQERLIIQ-----------------------------------VSKT---------
QKMEVLMREVERIMIFGN-VQLSTPAINACLKHNILVLFLN-QAGQYNGHLWSLGS--IHLNNE-
MVQIKRHQEHEFQVKISKAIVYGKLMNSKRLLMRLNR--KRQVP-------DMDKVIEGINSDI----LSLE-
--SVDNLDQLRG----YEGIAAARYFPAFGQLI--TNAAFSF------SLRNRQPP-
TDPVNSLLSFGYTLLFNNVLSLIISEGLSPYFGNFHYGERD-
KPYLAFDLMEEFRAIIVDGMVLRVINNGLLTLK-DF-E----PVA-SNG-G----------
VYLTDKGRRIFLKEFESRIN----KLISHPDIQSPVS-YRQTIQLQIRRYKQSLLSDV--SYQSFVRD
>DS_NF_Rivularia WP_015120903
IYLIEQGTNIYKDYQRFIIH-----------------------------------VSEK---------
PKLEVPIRDVQQIIVFGN-IQLSTPVIQACLKEQIPVVFLS-QTGTYHGHLWSEKS--IHLDNQ-
LVQAERRNDDLFRFSVSRAVVLGKLLNSKQLLMRFNR--RRKIG-------KVEEAIYGINQDI----DALN-
--YVDNLDTLRG----YEGIAAARYFPAFGNLI--TNPKFSF------SQRFRQPP-
TDEINSLLSFGYTLLFNNVLSFIITEGLSPYIGHFHYGDKQ-
KTYLAFDLMEEFRSPIVDSLVLKIINKSLFKPQ-DF-D----VVA-STG-G----------
VYLSQTSRRVFLKQFENRMN----EEISHPDLISQVT-YRHAIQLQVRRYKRCLLSDN--IYESFLRA
>DS_NF_crinalium WP_015201684
IYLLEPGSTLYKEHLRFVIW-----------------------------------VPDE---------
QKIEVPIREVERILVFGN-IQVTTQAINACLEQQILVLFLS-TTGQYKGHIWNWES--NHLGNE-
LIQIEKRQDPQFQFLVSQAIVRGKLANSRQLLMRLNR--KRKVA-------EVSQAIAGIGTDI----SALD-
--TVNNLDSLRG----YEGVGAARYFPAFGQLI--TNPEFSF------SLRYRQPP-
TDPVNSLLSFGYTLLFNNVLSLIVAEGLSPYFANLHYGEDK-
KPYLAFDLMEEFRSPIVDSLVLKLINSAVFKSR-DF-E----TVV-STG-G----------
VYLRKESRRIFLQHFEKRMN----EELSHPDLLSQVS-YRHAIQLQIRRYKRSLLHSI--AYEPFLRT
>PF_WP_017302244.1 CRISPR-associated endonuclease Cas1 [Nodosilinea nodulosa]
LYVVEQGAYLHKDQGRLVLK----------------------------------APRAK---------
DSLEIPLAEVERILLFGN-VQLTTAVIAACLQQQIPVIFLS-QLGDYKGHLWSAEI--TDLTAE-
AEQFARQHDEAFGCTTARAVVYGKLWNSKIFLLRQNR--KRQFA-------EVRTAIERLDGAL----
TTLAGD-APLSLEQIRG----YEGNGASEYFQTFGPLI--TNPGFSW------AGRNFHPP-
TDPVNSLLSFGYTLLFNNVFSLLLVEGLNPYLGHLHGAERQ-
KAYLAFDLMEEFRSPVVDALVMRLINQKIIRPT-DF-S----WPK-ENN-G----------
VYLTDPARRVFLRHFEQRIT----EKVTHPDVKEPVS-YRRVIQLQVKRYKRAVLGNQ--PYKAFERM
>PF_WP_045442561.1 CRISPR-associated endonuclease Cas1 [Synechococcus sp. NKBG042902]
LYLVQQGTTVKKEQGRFCVK-----------------------------------TPPE---------
HLFEMPVREVTQVLVFGN-IQLTTTVITECLERQIPVLFFS-QFGDYRGHLWSVEH--DDIRTE-
LLQYQRQGEAHFTQEVARAIVGAKLMNSRLLLLRLNR--KRKSD-------QVSRIIEALLEDV----DAVA-
--AAESLEQLRG----HEGAGAARYFQGLGQLI--VNEGFSF------TGRNRRPP-
KDPVNSLLSFGYTLLFNHVLSFILAAGLNPYLGHLHGSEKK-

KTFLAFDLMEEFRSPIVDSLVIRLINQKFVKPT-DF-T----WPN-EAG-G---------
IYLSDVARRPFLQQFEKRLS----LEVAHPDVAEPVS-YRRVIQLQVERYKQALINQT--PYEPYQRL
>PF_WP_006515493.1 CRISPR-associated endonuclease Cas1 [Leptolyngbya sp. PCC 7375]
LYLVQQGTTVHKEQGRFLIR-----------------------------------APQT---------
KTVEVPIREVEQILIFGN-CQLTTQVISVCLDQRITVVYLT-QMGDYKGHLWSAEA--EGMNAI-
MAQFERHQDDAFCLETARVIVARKLQNSKQLLLRLNR--KRKLE-------AVKISIEGLYTDL----DAVA-
--MATEINMLRG----HEGAAAARYFKALGKLI--NNPGFVF------NGRNRRPP-
TDPTNSLLSFGYTLLHNHVLSLILAEGLNPYLGNLHGSERK-
QTFLAFDLVEEFRSVIVDSLVMRLINQKAVRPT-DF-S----WPN-KQG-G---------
VYLQGTARRLFLKRFEERLS----LPVSHPDVSDTVS-YRRILQLQVRRYKQAVLNGV--IYEAYRKI
>PF_BAU44853.1 CRISPR-associated endonuclease Cas1 [Leptolyngbya sp. O-77]
LYLVRQGTTLRKDHGRFVIEGGRFETDEDESLTAQSPPSQPPTPKSKPQNPQSKTPEPF---------
PPLEIPIKEVGRILVLGN-VQISTSALSECLEHQIPVVFMS-RAGDYKGHLWSSEF--CDLPTE-
AAQFGRRHDPGFQVKMAQQILHGKLTNSRHLLLRLNR--KRKVE-------GLSAKIHRIDQHI----AALT-
--KTTDLDAMRG----HEGASARLYFQALGQLI--TNPGFSL------TERNRRPP-
KDPVNSLLSFGYTLLFNNVLSLILAEGLNPYLGNLHRSDRK-
EPHLAFDLMEEWRSPIVDSLVMWLINKKAIRPT-DF-T----FPN-AEG-G---------
VYLENTARRVFLKHFEDRIT----ETVTHPTVQQPVS-YRRAIQLQIQRYKKCLQDSQ--PYAPFIRS
>PF_WP_009625648.1 CRISPR-associated endonuclease Cas1 [Pseudanabaena biceps]
LYLVHQGATLRKEQGRFLVQ-----------------------------------PLKE---------
SALEIPIAEVELVLVFGN-IQLTTSAIAACLDAKIPVIFLT-QMGEYKGQLWNSEF--CDLPSE-
EAQWQRRLDVAFQLETARAIIWGKLMNSKQLLLRLNR--KRQLE-------DVTTAIAGITSDL----ESVE-
--TAETLESLRG----YEGIAANRYFVALGLLI--TNEGFSL------TGRTRRPP-
KDPVNSLLSFGYTLLYNNVLSLILAEGLNPYLGNLHRSDRK-
ETHLAFDLMEEFRSPVVDTLVMNLINKKILRPT-DF-T----YPD-KDG-G---------
IYLADAARRVFLKHFEERIS----LQIVYADLKEKIS-YRRVIQHQIWRYRSALLGEA--PYESFRRN
>PF_WP_051057839.1_(2) CRISPR-associated endonuclease Cas1 [Actinomyces cardiffensis]
IYVGRDGARVHVTKGRLIVD---------------------------------GTDGL---------
PQMSIPQRSVSRIVLTGS-VGLSAGARSWALYNDIDVLCLS-RRGTYLGQLAGPRS--
TVNARRLLAQAAFSDDEEQRLPLARSIVKAKLRNQVHVLNRIGR--RDTRT-------DVKSAAQELRNLA--
--EELT---HADSINEIMG----LEGAASTDYYTALSSLV---PEDVAF------QGRSRRPP-
RDLANAALSYCYAILLGECTGALFAAGLEPSLGILHSSTDK-
RPSLSLDLMEEFRPLLVDSTVMALLRTHRLRPE-HATS----APGDEDG-----------
VWLNREGKKIVVDGYEATMQ----RHVKGALPGFTGT-WRRHIHHEAQLLGRAIMEPDY-TWTGVAWR
>PF_WP_053587381.1 CRISPR-associated endonuclease Cas1 [Actinomyces sp. oral taxon 414]
VYVGRDGARVHVSKGRLIVS---------------------------------GADGL---------
PLVSIPRRAVSRIVLTGA-VGLSAGARSWALRNDVDVIFLS-RHGGYLGRLSAPRD--
TANARRLLTQAAFADDAEARLPLARAIVRAKMRHQVSVLHRTGR--RDGGA-------DVAGACALIRSQA--
--ASVA---DATDTDELMG----LEGAASSAYFDCLSRLV---PEDVAF------HGRSRRPP-
KDLANAAFSYAYAILLAECTGALLAAGLEPSLGVLHASTDK-
RPSLSLDLMEEFRPLLVDRTVMALLRSRRLRPEHATVS--------GDGEG----------
VWLDKDGKKVLVDGYEATLQ----RQVKGALPGFAGT-WRRHIHHQAQLLGRAIVEPDY-RWSGASWR
>PF_WP_009405645.1 CRISPR-associated endonuclease Cas1 [Actinomyces sp. oral taxon 175]
VYVGRDSARVHVSQNRLIVD---------------------------------GADGL---------
PQVSIPRRAVSRIVLTGA-VGLSSGARSWALYNDIDVIFLS-RHSGYLGQLAGPRS--
TASVRRLLTQASFATDDDARLPLARAIVRAKMRHQVSVLHRTGR--RSRGS-------GVETPCTTIRQLA--
--DDAK---QAADVDELMG----LEGAASTAYFGCLSGLV---PPDVAF------DGRSRRPP-
KDLANAALSYAYAILLAECTGALLAVGLEPSLGVLHASTDK-

RPSLSLDLMEEFRPLLVDRTVLSLLRSKRLRPEHATTS--------PDGEG----------
VWLNSKNKKTLVDGYEATLQ----RQVKGAMPGFSGT-WRRHIHHGAQLLGRAITEPGY-EWTGASWR
>PF_WP_021604855.1 CRISPR-associated endonuclease Cas1 [Actinomyces johnsonii]
VYVGRDGARVHVSQNRLIVD----------------------------------GADGL---------
PQVSIPRRAVSRIVLTGA-VGLSSGARSWALYNDIDVVFLS-RHGGYLGQLAGPRS--
TASARRLLTQASFATDDDARLPLARAIVRAKMRHQVSVLHRTGR--RSQGS-------DVETPCTTIRQLA--
--DDAE---QAADVDELMG----LEGAASTAYFGCLSGLV---PPEVAF------DGRSRRPP-
KDLANAALSYAYAILLAECTGALLAAGLEPSLGVLHASTDK-RPSLSLDLY----------------------
---------------AAG----------------PPGRHA--------------GGLGLPAAGVRFP------
---------AAAGAR----------
>PF_WP_021609237.1 hypothetical protein [Actinomyces johnsonii]
VYVGRDGARVHVSQNRLIVD----------------------------------GTDGL---------
SQVSIPRRAVSRIVLTGA-VGLSSGARS---

-
>PF_WP_006063846.1 CRISPR-associated endonuclease Cas1 [Corynebacterium durum]
LYLGAQTAHARISKGRLIVE-----------------------------------DRQK---
GGGKPTKLFDVPCGHVSRIVAFGS-VAISSGLRSWAMYNGVDIVLAS-RRGSYLGIMQGTKT--ETHSAR-
LIAQVEINDTPRQLEISCAIIRAKIHKQIVVLQRFSK--RESEE-------KVSNAITNMRKFM----TMLD-
--DTASIDEAMG----LEGAAAKEYFAAYGALL---PNELAF------TTRSRQPP-
LDLANASLSFLYTVLLGECVTALRAAGMDPGLGVLHNPQAK-
RPSLALDLLEEFRPLVADQVVLNAARRNRLTSK-HARK----D---PKGQG----------
ILLTKKGREEILKAYEERML----QVVKGSIPDFAGS-IRRHLYRQAQRLAGAIMHDDY-EWSGMSWR
>W_CRISPR-associated_endonuclease_Cas1_[Microlunatus_phosphovorus]_(2) 336116789
LYAGLQGGRVRVKQGRVIVE-----------------------------------TADD--------
VVALDVPTDHVSRVVCFGS-VGFSAGARSWAMSKDVDVVFVS-RRGTYQGQLLPASSP-TRAERL-
RGQLAFSDDPTRTVPLALAIVEAKIRKQIVLLQRFGR--RAHAE-------LVQEAVAEMRNVL----RLLP-
--DCTSTEEAMG----VEGAAARVYFPCLGQLF---PAELQF------TLRSRQPP-
MDIANSALSYLYTLLTAECVTALVAAGLDPAIGFLHADDKG-
RPSLALDLVEEFRPHVADQVVITAARNGELTAA-SGHT----EPG-KSG------------
VLLTKAARTSLLAGYELRML----RTTKGALPDFSGT-LRRHLYRQAQRLLATVTRGE--PWTGLSWR
>PF_WP_051209229.1 CRISPR-associated endonuclease Cas1 [Propionicicella superfundia]
LYAGLQGGRIRISQGRLLAQ----------------------------------TKDDE---------
TVLDVPKRHVGRIVCFGS-VGLSAGARSWALSHDVDVVFAS-RSGAYLGTMVSHEQ--RYRPAR-
LRAQLDAVGSAQALAIGRAIIAAKVRKQEVVLQRANH---------PDLVDDVRDVVRHLAGLR----RMLP-
--DAGSAAELMG----LEGAAAQAYFPMLGRLM---PEELRF------ALRSRRPP-
EDVPNAALSFLYTVLLGECVTALHAAGLDPSIGVLHADQGN-
RPSLALDLMEEFRPHIVDSVVLRVARRGGLTVA-SGRS--------EPGRG----------
VLLTKAGREAVLDAYETRML----TATRGALPGFAGS-LRRHVLRQAQRLRAAIMDRGQ-AWTGLSWR
>PF_WP_052396493.1 CRISPR-associated endonuclease Cas1 [Kutzneria sp. 744]
LYAGIQGSRARIEQGRVVVE-----------------------------------HQDA---------
EVLDVPAGHVARIVCSGA-VGVSSGLRNWALSGGVELVFCS-QRGRYLGQTVSGHL--GRVERL-
RRQLACADDPERHLPLARSIVDIKIRKQAVLLQRLTR--REQAR-------ELSEAVAAMRGYA----AMAP-
--QAMSRAEVMG----LEGAAARAYFQAWAAIV----PELGF------SGRNRRPP-
LAVVNSALSFGYAVLLSEAVTALAAAGMDSSVGFLHTEQDG-

RPSLALDLMEEFRPLVVDQVVLELVRRNRLGPQ-H--G----HRDEERGGV-----------
LLTRAGREALLDGYERRML----TVTRGALPGFAGS-LRRHLYRQAQVLAAWVDGVGP-GLVGLSWR
>W_hypothetical_protein_[Nakamurella_multipartita]_(2) 258651473
LYLGVQGSRARIMDGRVIVE------------------------------------SGD-------
DAELLDVPSGLIERIVTFGG-VGVSAGLRSWALANQVDLVFLS-RRGSYLGHAWAAAA---------------
----
--DHRVSRLR-----------------------
--------------------AQLAAAD----------DQAVWLPLNPPGVSGG--------------------
---CLKR--------------------

>PF_WP_052104813.1 CRISPR-associated endonuclease Cas1 [Cellulomonas bogoriensis]
MVVSEGGAVVRTRRGRVRVD-----------------------------------RDGE---------
TVASMSLSRVARIVVQGR-VGLTTPLLHEAAQRGIDVVMLS-RSGGYVGRLSRRRP--GDPSLR-
RAQARAYDSGADLERLTTAFVSGKITNMRVAVLRHQR--------GAGTSEEGARVAAQLAEAR----ARAS-
--VMRSVPSLMG----VEGAATRAYFGWLGSRV---GEEWGF------HGRARRPP-
PDPVNSMLSYGYVLLCAEGVSACEQAGLDPDMGFLHSDRWG-
RPSLALDLMEEWRPVIVDSTVLRIISNKRLKPS-DF------TFDAKQG------------
ARMTAHARQTFLREYEARML-----TLAGSDAGAGRQPYRRLIATQAMRLAEALRTPGG-PYRPYVWR
>PF_WP_052914180.1 CRISPR-associated endonuclease Cas1 [Frankia sp. BMG5.1]
VYVDRQGSLVRSRGDKLVVT-----------------------------------DGEE---------
SLLRLNLRRVRQVVCYGR-VGLTTAFLHQAAERGIEVVLLT-EQGVLGARLTAPTA--SDPQIR-
RVQYKAADDEQRGRTLAAAFVEGKVGNLRVAVLRSAR--REDDA-------EAFVAAELMRELA----GRLP-
--ECATLDEILG----IEGAASREYFQTLRRML---DPIWGF------EGRNRRPP-
PDPVNAMLSYGYTILTHEAIAAAEAAGLDPMVGFLHQHRWG-
RPALALDLMEEFRPITVDVAMWRAISSGQIRPE-QF------ENDPNQG------------
CRMAADARHAFVTAYEKRML----TLTTHQPSGRRVS-YRVALSLQAKALARALRDPGE-PYVPLRWK
>PF_KXK58998.1 hypothetical protein AWW66_26575 [Micromonospora rosaria]
VFVTTQGALLRVKGERLRVE-----------------------------------DGDR---------
LLANVNLKRVRQIVCFGR-VGVTSTLLQRIVERGIELAWLY-EDGRHAARVSGLDG--TDPEVR-
LAQYRAADDARQALRIARQLVAGKVTNMRVGLLRAAR--AQQAP-------ELADRQARLATAR----QSAL-
--IADSTAELMG----YEGSATRDYFAGLSQIL---GPEWGF------TTRQRRPP-
PDPVNAMLSFGYTLLTNEALTACQLAGLDPYLGMLHSPRRN-
RPSLALDLIEELRPVVVDATVIRLVRTGQVTPK-NF-T----LTD-DRG------------
CRLDDHGRRAFLDAYERRML----TLVHHPVEQRRIP-WRHVLLAQARTLAAVLSSRRP-EYRPVVWR
>PF_WP_017559367.1 CRISPR-associated endonuclease Cas1 [Nocardiopsis baichengensis]
MYITRPGALVRSRGDRVRVE-----------------------------------HNDT---------
ILLSVNLKRVRQVVGIGR-VGFSTPFLHRALRQGVELVLLD-DLGRFQGRLSAALG--GDVHVR-
AAQYEAALDGARAMELARVFVAGKIANLRTGLLRASR--QRGAR-----TLDIAPAAEHLAHAR----STAL-
--ETPGMNELLG----TEGAAAREYFGLLGRLL---PAEWGF------THRRRRPP-
PDPVNAMLSLGYTLLLNDAVVACHIAGLDPEGGFLHTLRRG-
RASLALDLIEEFRPLIVDAVVARLLLGAKLTPA-DFET-----PQAERG------------
CRMKPDALKTFLAAYETRML----TLARHPGLGRRVS-YRTALVAQARLLAAVIAGEEQ-AYRPLAWR
>PF_WP_020380191.1 hypothetical protein [Nocardiopsis potens]
VYVTTPGALMRSRGERVRIE-----------------------------------HGEK---------
VLLSSGLKRIRQVVGVGR-IGFSTPFLHRALRQGVELVLLD-DLGRFQGRPSRALG--GGVHVR-
AAQYEAALDGARAVGFARLFVASKPANLRTALLRASR------------------SVNAPEATA---------
--EDAARWPS-----------------

---AWPGPGLRL-----------------------
